# Pan-Cancer analysis of the expression and regulation of matrisome genes across 32 tumor types

**DOI:** 10.1016/j.mbplus.2019.04.001

**Published:** 2019-04-06

**Authors:** Valerio Izzi, Juho Lakkala, Raman Devarajan, Anni Kääriäinen, Jarkko Koivunen, Ritva Heljasvaara, Taina Pihlajaniemi

**Affiliations:** a Oulu Center for Cell-Matrix Research and Biocenter Oulu, Faculty of Biochemistry and Molecular Medicine, University of Oulu, P.O. Box 5000, FIN-90014 Oulu, Finland; b Centre for Cancer Biomarkers (CCBIO), Department of Biomedicine, University of Bergen, N-5009 Bergen, Norway

**Keywords:** Pan-Cancer, Tumor, Matrisome, Gene regulation, Prognostic, Therapy

## Abstract

The microenvironment plays a central role in cancer, and neoplastic cells actively shape it to their needs by complex arrays of extracellular matrix (ECM) proteins, enzymes, cytokines and growth factors collectively referred to as the matrisome. Studies on the cancer matrisome have been performed for single or few neoplasms, but a more systematic analysis is still missing. Here we present a Pan-Cancer study of matrisome gene expression in 10,487 patients across 32 tumor types, supplemented with transcription factors (TFs) and driver genes/pathways regulating each tumor's matrisome. We report on 919 TF-target pairs, either used specifically or shared across tumor types, and their prognostic significance, 40 master regulators, 31 overarching regulatory pathways and the potential for druggability with FDA-approved cancer drugs. These results provide a comprehensive transcriptional architecture of the cancer matrisome and suggest the need for development of specific matrisome-targeting approaches for future therapies.

## Highlights

• In-depth characterization of matrisome gene expression and regulation in 10,487 patients across 32 human tumor types.
• Identification of transcription factor (TF) and “master regulators” governing each cancer’s matrisome.
• Analysis unveils therapeutic possibilities and suggests new treatments by repurposing of FDA-approved cancer drugs.

## Introduction

The crucial role played by the microenvironment in cancer development, progression and dissemination has been extensively investigated [[1], [2], [3], [4], [5]] and evidence begin to accumulate for Pan-Cancer “microenvironmental signatures” impacting patients' prognosis and survival [[6], [7], [8]]. Cancer microenvironment is a complex entity though, populated by different cell types embedded in a mesh of structural and functional extracellular matrix (ECM) and ECM-associated molecules, ECM-remodeling enzymes, cytokines and growth factors collectively referred to as the matrisome [[9], [10], [11]]. Altered expression of matrisome proteins is intimately linked to cancer progression, as *e.g.* in the case of collagen I, II, III, V and IX deposition in a vast array of solid tumors [12,13] and impaired laminin adhesion in breast and lung cancer [14,15]. Likewise, ECM remodeling *via* metalloproteinases and impaired or ectopic interaction with ECM receptors such as CD44 are reportedly major Pan-Cancer drivers of dissemination and resistance to therapy [13,16,17].

On the other hand, understanding and manipulating the peritumoral matrisome composition shows promising therapeutic potential. In example, over-expressed matrisome proteins can be used as “beacons” for antibody-drug conjugate therapy to direct the chemotherapeutic cargo just next to the cancer cells, thus maximizing therapeutic effects while minimizing toxicity [18], and ECM-cancer interaction mechanisms can be manipulated to increase the efficacy of antimetastatic drugs [19,20]. The burgeoning of cancer “omics” data during the last years, led by The Cancer Genome Atlas (TCGA) Research Network and culminated in the Pan-Cancer Atlas (the most comprehensive collection of integrated data on gene expression, methylation, mutation and copy-number variation in human cancers available to date), has provided an incredible momentum to any field of cancer research [[21], [22], [23]]. Several “layers” of information from TCGA have obviously already entered the matrisome field, contributing to the identification of subtle patient phenotypes and molecular mechanisms [[24], [25], [26], [27]], but a comprehensive view of the whole “cancer matrisome”, systematically defining gene expression and its transcriptional regulatory circuitry, is still missing.

Considering the centrality of matrisome in cancer, we have attempted to fill this gap by compiling a systematic atlas of matrisome data from the TGCA Pan-Cancer cohort to discover similarities and differences between different tumors and to infer regulatory relationships driving matrisome organization in different tumors. We show here that the composition of each “cancer matrisome” is a combination of tumor-specific molecular phenotypes, tissue- and system-of-origin features and physiopathological mechanisms. We further show that different tumors share similar matrisome organization under similar master regulatory mechanisms, suggestive of common microenvironmental needs, and that matrisome expression has significant impact on Pan-Cancer and tumor-specific patient survival and offers possibilities for therapeutic interventions.

## Results and discussion

We analyzed the expression of 820 matrisome genes [10] across 32 cohorts of The Cancer Genome Atlas (TCGA) Pan-Cancer initiative [28]. After filtering out samples with missing clinical or genetic information, 10,487 patients from different cohorts were included (Suppl Table 1).

**Fig. 1:**
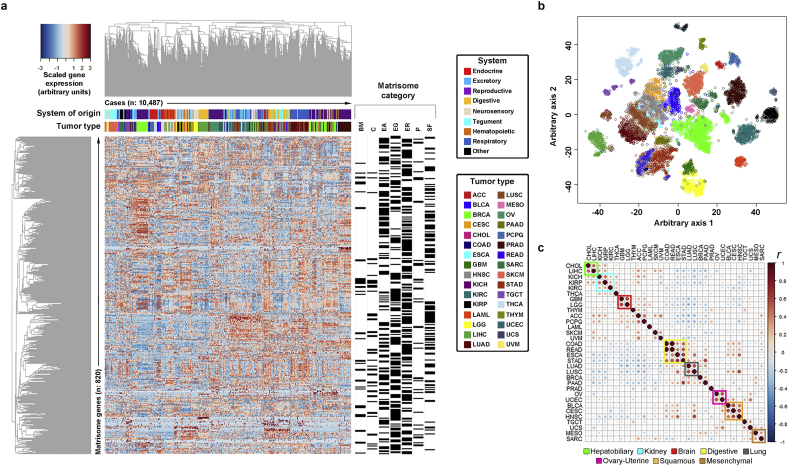
The cancer matrisome. (a) Heatmap of the expression of 820 matrisome genes in 10,487 cases from 32 tumor types. The matrisome category of each gene is given on the right as black bars, while tumor types and system of the body from which each tumor originates are color-coded and placed on top of the heatmap. The distance metrics for hierarchical clustering is 1-Pearson correlation (*r*). *Abbreviations*: ACC, adrenocortical cancer; BLCA, bladder urothelial carcinoma; BRCA, breast invasive carcinoma; CESC, cervical and endocervical cancer; CHOL, cholangiocarcinoma; COAD, colon adenocarcinoma; ESCA, esophageal carcinoma; GBM, glioblastoma multiforme; HNSC, head & neck squamous cell carcinoma; KICH, kidney chromophobe; KIRC, kidney clear cell carcinoma; KIRP, kidney papillary cell carcinoma; LAML, acute myeloid leukemia; LGG, brain lower grade glioma; LIHC, liver hepatocellular carcinoma; LUAD, lung adenocarcinoma; LUSC, lung squamous cell carcinoma; MESO, mesothelioma; OV, ovarian serous cystadenocarcinoma; PAAD, pancreatic adenocarcinoma; PCPG, pheochromocytoma & paraganglioma; PRAD, prostate adenocarcinoma; READ, rectum adenocarcinoma; SARC, sarcoma; SKCM, skin cutaneous melanoma; STAD, stomach adenocarcinoma; TGCT, testicular germ cell tumor; THCA, thyroid carcinoma; THYM, thymoma; UCEC, uterine corpus endometrioid carcinoma; UCS, uterine carcinosarcoma; UVM, uveal melanoma. BM, basement membranes; C, collagens; EA, ECM-affiliated; EG, ECM glycoproteins; ER, ECM regulators; P, proteoglycans; SF, secreted factors. (b) Multidimensional scaling of the data in (a) by T-distributed Stochastic Neighbor Embedding (t-SNE). (c) Inter-tumor correlation (Pearson correlation (*r*)) based on the 100 most-expressed matrisome genes in each tumor type. Clusters of highly correlated tumor types are reported as colored squares.

At global level, matrisome gene expression separated neatly each tumor (Fig. 1a, b) with an average recall accuracy per tumor of 95% by 3 independent automatic classification algorithms (χ-square test <0.0001). Tissue-of-origin effects were typical confounding factors as already reported [29], determining misclassification of e.g. rectal (READ) as colon (COAD) cancer in 25% of the cases, COAD as READ in 8% of the cases, lung squamous (LUSC) as adenomatous (LUAD) cancer in 7% of the cases, and uterine carcinosarcoma (UCS) as uterine endometrial carcinoma (UCEC) in 8% of the cases and as sarcoma (SARC) in 9% of the cases (Suppl Table 2, 3 and Suppl Fig. 1). Restricting the analysis to the most-expressed genes in each tumor type exacerbated tissue-of-origin effects and highlighted clusters of highly correlated tumors, in which tissue- and organ-of-origin effects as well as histopathological features such as squamosity converged (Fig. 1c and Suppl Figs. 2, 3). Notably, the overall similarity between tumors was much lower than between healthy samples from matching tissues (Suppl Fig. 4, 5), suggesting a strong matrisome specialization during cancer development [30]. We identified 29 tumor-specific matrisome signatures (see Suppl Table 4), which varied significantly in size and composition (χ-square test <0.001). With respect to global matrisome expression, the signatures had the benefit of being smaller and easier to further investigate and to concentrate on those matrisome genes which scored among the highest-ranking genes in each tumor type. Also, multi-tumor clustering could be performed with signatures, producing coherent high-ranking results in multiple cohorts and tests used for cross-validation (see Suppl Table 5 and Suppl Figs. 6–8).

**Fig. 2:**
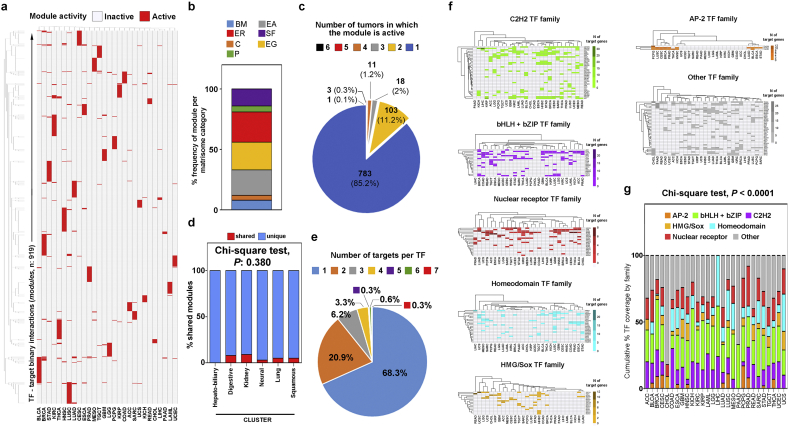
Modular transcriptional control of the matrisome signatures. (a) Heatmap of module activation in each tumor type. A module is defined as the binary interaction (positive Pearson correlation, significantly higher than in any other tumor type) between a transcription factor (TF) and a gene of the matrisome signature of the given cancer. (b) Frequency of modules regulating each matrisome category. (c) Usage of the same module between different tumors is infrequent, and (d) does not depend on clusters. “Shared” indicates a module used by at least 2 tumor types of the same cluster. (e) Number of matrisome targets per each TF identified. (f) Abundance of different members of TF families in each tumor. % TF coverage is the % of modules having the given TF in respect to the total of modules for the given cancer. (g) Cumulative coverage by family. Values sum all the individual TF coverage familywise for the given cancer.

Using a multi-source consensus approach, we then inferred a complete chart of TFs driving gene expression (each TF-target interaction is defined here as a “module”) for 28 tumor types. Samples in this and further analyses included almost only primary tumors (99.27% of total) while metastatic tumors accounted for 0.3% of total and recurrent tumors for 0.43% of total (see Suppl Fig. 9a). Before further modelling, we also evaluated global and tumor-specific patterns of matrisome expression in primary and metastatic or recurrent cancers, in light of suggested matrisome differences in the primary and metastatic niches [31,32]. Data in Suppl Fig. 9b, c show that, for what concerns matrisome expression, metastatic and recurrent tumors overlap with primary tumors, both at the global and tumor-specific level (BRCA, THCA, GBM and LGG are considered as examples), in line with similar recent findings by Naba et al. at proteomics level [33]. Next, we moved on to identify transcriptional modules differentially governing the matrisome components (Fig. 2a, b and Suppl Table 6); of these (919 unique modules in total), the majority (783 modules, 85.2%) was highly specific and only active in one tumor type, with the remainder (136 modules, 14.8%) active in two or more tumor types (Fig. 2c) independently of eventual clusters (Fig. 2d). In line with the expected sparsity of gene regulatory networks [34], approx. 89% of all identified TFs had only 1 or 2 matrisome targets and “hub” TFs with 3 or more connections were the minority (Fig. 2e and Suppl Fig. 10). The use of different TF families characterizes the different tumors (χ-square test <0.001) and aligns with the expected abundance of TFs from whole-genome binding studies and functional connections with the ECM [[35], [36], [37], [38], [39], [40], [41]]. *E.g.*, leaving aside the TFs which were not grouped into a specific subfamily (the “other” group), the C2H2 family of zinc-finger factors was the most represented through all tumors (on average 26% of all TFs), followed by leucine zipper/helix-loop-helix factors (bHLH+bZIP) and nuclear receptor TFs (14% and 13% of the total, respectively). Homeodomain-containing TFs were also abundant (10% of all TFs) while HMG/Sox factors and AP-2 were less represented (5% and 3% of the total, respectively), though their expression varied significantly between tumors. In fact, AP-2 reached approx. 10% in breast (BRCA), cervical/endocervical (CESC), lung squamous (LUSC) and prostate cancer (PRAD), and nearly 20% of all TFs in pheocromocytoma/paraganglioma (PCPG). HMG/Sox TF were more typically used by clusters of similar tumors, covering approx. 10% of total TFs in brain (GBM and LGG), colorectal (COAD and READ), uterine (UCEC and UCS) and head-and-neck neoplasms (HNSC). The abundant presence of the “other” group depends on the inclusion of several TFs which are crucial for multiple cancers, such as *PAX* and *NFKB* factors in the Rel subfamily, *RUNX1*, *2* and *3* in the Runt subfamily and *TEAD1*, *3* and *4* in the Tea subfamily [35,36,42] (Fig. 2f, g). In line with previous studies [43] and the proposed specialization of matrisome transcriptional programs during cancer development, we also notice that the TF-target correlation within a module for a given cancer is significantly higher (average approx. 8-fold) than the correlation between the same TF-target pair in the rest of the Pan-Cancer cohort (Suppl Fig. 11a). Furthermore, a sizeable proportion of the TFs and targets identified by our analysis stain at “high” or “medium” level (average 61 and 41%, respectively) in tumor samples from The Human Protein Atlas (Suppl Fig. 11b), facilitating future investigations.

We found 233 modules linked to differential patient survival (Suppl Table 7), with examples such as adrenocortical (ACC), bladder (BLCA), breast (BRCA), lung adenomatous cancer (LUAD), sarcomas (SARC) and kidney clear cell carcinomas (KIRC) showing approx. 30% or more modules with prognostic potential and extremes such as low-grade glioma (LGG) and pancreatic cancer (PAAD) showing >50% matrisome modules with significant prognostic value (Suppl Fig. 11c). Notably, in any case in which a TF controlled multiple targets through multiple prognostic modules, the “direction” of the prognostic association (towards either better or worse prognosis) was the same for all the modules, clearly defining noxious or favorable transcriptional circuits for each tumor (Suppl Table 7). Likewise, when the same matrisome target was under different TFs in multiple prognostic modules within the same tumor, the “direction” of the prognostic association was equal, marking noxious and favorable matrisome regulatory mechanisms as above (Suppl Table 7). In line with previous findings, we could not identify global differences in the module profiles of metastatic or recurrent tumors in respect to primary tumors, while rather only a few modules varied their expression to the plus or the minus side, again suggesting that matrisome differences in secondary tumors are limited and very specific (see Suppl Fig. 9d).

**Fig. 3:**
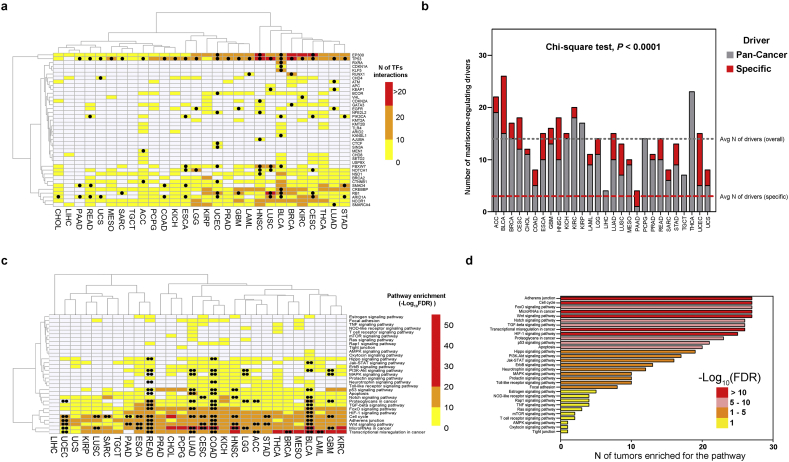
Master regulators of the cancer matrisome. Identification of driver genes and pathways regulating the matrisome signatures of any given cancer. (a) Heatmap of all driver genes identified as regulators in this study and the number of transcription factors (TF) they regulate. Dots indicate that the regulator is a cancer-specific driver for the given neoplasm. (b) Number of drivers found per cancer type, including general (Pan-Cancer) and tumor-specific (specific) cancer drivers. Average number of total and specific drivers across the study is reported. (c) KEGG enrichment for pathways regulating the matrisome (master regulators + TFs) in any given cancer. Double dots indicate pathways with oncogenic mutations in at least two cancer-specific drivers for the given neoplasm. Values are shown as antilog_10_ of false discovery rate (−Log_10_(FDR)). (d) Number of tumor types in which each matrisome-regulating pathway is enriched.

Analysis of master regulators identified 40 cancer-specific or Pan-Cancer driver genes [44] which physically interacted with the TFs presiding the various modules and explained the majority of each cancer matrisome (Fig. 3a, Suppl Fig. 12 and Suppl Table 8). On average, tumors expressed about 14 matrisome-controlling master regulators, 3 of which cancer-specific (Fig. 3b), though in some cases such as bladder and uterine corpus neoplasms (BLCA and UCEC) the number of cancer-specific regulators increased notably, in line with recent reports [44]. The master regulators identified were typically hub proteins (such as TP53 and P300), governing large networks of TFs as expected [45], and the number of drivers in a cancer type correlated linearly with the number of TFs they controlled (Suppl Fig. 13). Cancer drivers occurring in at least two cancer types (“common” regulators) governed, on average, a higher number of TFs than those occurring in a single tumor type (“unique” regulators, see Suppl Fig. 14a–c), possibly providing a further explanation to the observed overlap of matrisome profiles between different tumor types within the same cluster. Also, cancer-specific drivers governed generally more targets than drivers with no specific association (Pan-Cancer - only, Suppl Fig. 14d), suggesting that the cancer matrisome is globally mastered by a relatively small set of regulators which acquire oncogenic mutations in multiple tumors [7,46]. Combinations of regulators are not otherwise interchangeable and, *e.g.*, those characterizing the different clusters such as the digestive system (ESCA, STAD, COAD and READ), the brain (GBM and LGG) or the lung (LUAD and LUSC) show a clear ability to discriminate their cluster of origin (see Suppl Fig. 15). Mapping master regulators and TFs to biological pathways, finally, provided further insights into cancer matrisome regulation. In general, pathway activation by mutations (at least two cancer-specific master regulators carrying oncogenic mutations in the same pathway) was infrequent, though in some tumors (BLCA, COAD, LUAD, READ and UCEC) it accounted for the majority of active pathways. Unsurprisingly [[47], [48], [49], [50], [51], [52], [53], [54], [55]], cell-to-cell adhesion and transcriptional avenues (FoxO, Wnt and MicroRNAs) were the most common matrisome-controlling pathways (27/27, 100% tumor types), followed by other expected pathways such as *TP53* and apoptosis, Notch and TGF-β signaling, and hypoxia [[56], [57], [58], [59], [60], [61], [62], [63], [64], [65]] (Fig. 3c, d and Suppl Table 9).

Finally, we used the Drug Gene Interaction Database (DGIdb) [66] to assess the therapeutic implications of our findings. A wide array of drugs, small molecules, and more broadly-defined therapeutic approaches were found to potentially interact with the different levels of the cancer matrisome architecture (Suppl Fig. 16a). Among these, FDA-approved cancer drugs currently labelled for use in cancer show no inferable direct interaction with the matrisome and its circuitry, though many FDA-approved cancer drugs would seem to have such potential if used off-label in different cancers (Suppl Fig. 16b and Suppl Table 10). We will here provide an example on the use of these data for the prioritization of future drug analyses on the cancer matrisome. As these data are inferred from the intersection of multiple databases and high-throughput screenings, however, we stress that robust, low-throughput validation of the results at the experimental level will be needed before their further use. According to our findings, Axitinib (tyrosine kinase inhibitor) and Dactinomycin (Actinomycin-D) could be re-purposed towards brain and kidney tumors (GBM, LGG and KIRC) to suppress *CSF1* activity. This would be particularly beneficial since *CSF1* expression, due to 3 modules in KIRC and to 1 in GBM and LGG, associates with poorer prognosis in these cancers (Suppl Table 5 and Suppl Fig. 17) in concert with the upregulation of its receptor (*CSF1R*), as already shown [67,68].

The findings we report represent the first attempt to systematize and understand matrisome regulation in cancer, providing an initial platform for further integration with the many aspects of cancer inherently linked to the microenvironment, such as genomic instability [29] and the immune system [69]. Furthermore, our report unveils prognostic matrisome circuitries in almost every tumor type assessed and informs on therapeutic approaches and the possible re-purposing of FDA-approved drugs for targeting the cancer matrisome. With the increasing understanding of the crucial role played by the tumor microenvironment in prognosis and response to therapy [69,70], our analysis might help in the identification of novel markers providing several valid and testable hypotheses for next exploration in cancer biology.

## Experimental procedures

### Data sources

The Cancer Genome Atlas (TCGA) Pan-Cancer RNA-seq V2 gene expression data and clinical data, data from matching healthy donors (TCGA) or unrelated healthy tissues (Genotype-Tissue Expression (GTEx) database), all from Toil [71] recomputing (RSEM, batch-normalized, log2(x + 1)-transformed and upper quantile normalized), were downloaded from the UCSC Xena Browser (https://xenabrowser.net/). Textual transcription factor (TF)-target interactions were downloaded from TRRUST [72], ENCODE [73], Marbach [74] and MSigDB [75]. Cap analysis of gene expression (CAGE) data from FANTOM5 [76] were accessed and downloaded *via* the Semantic catalogue of Samples, Transcription initiation, And Regulations (SSTAR) [77]. FANTOM5 data were restricted to cancer cell samples matching, as much as possible, the tumor types from TCGA as reported in Suppl Table 11 and only the reads from the most-supported promoter (“p1”) were considered. Tumor-specific and Pan-Cancer drivers were from Bailey et al. [44]. Human protein-protein interaction list was downloaded from BioGRID [78]. Staining profiles for proteins in human tumor tissue based on immunohistochemisty were downloaded from The Human Protein Atlas (THPA) [79]. Druggable genes and drug-genes interactions were from the Drug Gene Interaction Database (DGIdb) [66]. FDA-approved cancer drugs were from Sun et al. [80]. The list of matrisome genes was from the Matrisome Project [10].

### Software

Majority of the analyses were performed in R x64 v3.3.2, with specific packages introduced next to relevant methods. Linear support vector machine for feature selection (LSVM) of master regulators at the cluster level was performed in IBM SPSS Modeler 18. Network analyses and gene onthology (GO) enrichment were performed in Cytoscape v3.3.0 with manual overlays. Kyoto Encyclopedia of Genes and Genomes (KEGG) pathway enrichment was performed in StringDB [81]. Majority of the graphics were produced with GraphPad Prism V5. Cross-validation of cluster signatures was performed in Oncomine [82]. R codes for inferring TF, cross-validating results in THPA, evaluating module association with survival, identifying master regulators and evaluating druggability (as well as the data associated with the codes) are available upon request to the Authors.

### Gene expression and Pan-Cancer classification

The initial data matrix from TCGA Pan-Cancer was intersected with the clinical data and with the list of matrisome genes to include only the genes present in all samples with complete clinical information. This filtering process resulted in a final database of 820 matrisome genes out of 1028 total (820/1028, 79.76%) and 10,487 samples out of 11,257 total, excluding healthy donors and samples with no annotation (10,487/11,257, 93.16%), spanning 32 different tumor types. The types of tumor and the number of samples per each type, as well as the system of origin of each neoplasm, are reported in Suppl Table 1. Heatmap was generated with the gplots package v3.0.1, using 1-Pearson correlation (*r*) as the distance metrics for hierarchical clustering and information about system of origin, tumor type and matrisome category of each gene as overlays. T-distributed Stochastic Neighbor Embedding (tSNE) using a Barnes-Hut implementation was calculated using the Rtsne package v0.13 and the same distance metrics as for the heatmap. Automated sample classification and cross-validation was performed using 3 different algorithms: Support Vector Machine (SVM), perceptron-based Neural Network (NN), and the C5.0 classification and regression algorithm (C5.0). SVM analysis was performed using the e1071 package v1.7-0, setting the analysis to C-classification, data scaling (standardization), radial basis function (RBF) kernel, RBF Gamma 0.1, Gamma 1/dimensions, and 100× cross-validation (80% training, 20% test) *via* random sample permutation. NN analysis was performed using the neuralnet package v1.33, data scaling (standardization), 250 as the maximum steps for the training and 10× cross-validation (80% training, 20% test) *via* random sample permutation. C5.0 analysis was performed using the C50 package v0.1.2, with default Quinlan's C5.0 algorithm and 10× cross-validation (80% training, 20% test) *via* random sample permutation. Results from each run of each algorithm were averaged and reported in Suppl Table 2. Total recall accuracy from the combination of the 3 models is reported in Suppl Table 3 and used to build cross-tumor similarities and misclassification tables. Contingency of cross-classified cases was analyzed using the Stats package v3.6.0, Chi-square test with Yates continuity correction.

For clustering based on correlation analysis, average expression was calculated per each gene in each tumor type and genes were ranked accordingly. Next, the 20, 50 and 100 most- and least-expressed genes were extracted from each tumor and two new Pan-Cancer lists were created. These lists were subjected to correlation (Pearson correlation (*r*)) analysis using the corrplot package v0.84. The same approaches to t-SNE and correlation-based clustering were followed for the healthy donor samples included in the original TCGA entry (727, after removing samples with missing information) and for GTEx (6456 samples). Matching GTEx-TCGA tissues are reported in Suppl Table 12. For GTEx only, the correlation analysis was repeated twice using both the whole “TCGA TARGET GTEx” cohort from the UCSC TOIL RNA-seq recompute pipeline and isolating GTEx samples, or the individual GTEx cohort from Xena. The two analyses were run in order to verify whether horizontal (cross-cohort) normalization (in the “TCGA TARGET GTEx” cohort) affects the results and the differences between TCGA and GTEx. We observe no differences between the comparisons of cross-normalized cohorts and individually-normalized TCGA and GTEx (not shown), and data are available upon request to the Authors.

To infer gene signatures, the expression of each gene in each tumor type was confronted against the same gene in healthy matching TCGA samples (if present) or in GTEx using the “TCGA TARGET GTEx” cohort. In parallel, each gene in each tumor type was also compared *vs.* the same gene in all other tumor types, excluding tumors belonging to the same cluster as identified by correlation analysis, if present. Note that uveal melanoma (UVM) was dropped from the cases further analyzed at this stage because of no corresponding healthy tissue identifiable. All analyses were performed using the Stats package v3.6.0, two-sided multiple pairwise *t*-test with Benjamini-Hochberg adjustment for *P* values. Only the genes up-regulated in tumors *vs.* both healthy and rest of Pan-Cancer at *P* < 0.05 were kept as signature members. Contingency of signature composition in terms of matrisome categories was analyzed using the Stats package v3.6.0, Chi-square test with Yates continuity correction, and binary heatmap was generated using the gplots package v3.0.1, using 1-Pearson correlation (*r*) as the distance metrics for hierarchical clustering. Signatures for digestive (ESCA, STAD, COAD, READ), lung (LUAD, LUSC), brain (GBM, LGG), ovary-uterine (OV, UCEC), hepatobiliary (LIHC, CHOL) and kidney (KICH, KIRC, KIRP) clusters were inferred by comparing the single tumors and retaining the genes common to all members. Quasi-proportional Venn diagrams were calculated using nVennR package v0.2.1. The hepatobiliary and kidney cluster signatures were not further investigated because of their small size (8 and 2 genes, respectively). The remaining signatures were cross-validated by evaluating the ranking of their components in several tests (tumor *vs.* matching healthy samples) in multiple cohorts from Oncomine (Suppl Table 13). A test was considered positive if the gene in tumor had higher ranking than in healthy (reported as red in the heatmap from Oncomine).

### Transcriptional regulation inference

Our approach leverages on a multistep consensus scheme to infer and cross-validate transcription factor (TF) - matrisome binary interactions (modules), upon which a “master regulator” layer is built based on known protein-protein interactions and oncogenicity features of master regulators. To define modules, in the first step a model was built by mining all TF interactions with the matrisome genes in each cancer signature from four databases (TRRUST, ENCODE, Marbach and MSigDB). In parallel, the resulting TFs were searched for occurrence in matching FANTOM5 samples and signature genes were investigated for over-representation of TF binding sites. Note that ovarian cancer (OV) and thymoma (THYM) were dropped from the cases further analyzed at this stage because of no corresponding FANTOM5 samples identifiable. Results were matched and only the modules present in two inference schemes (one of which is obligatory FANTOM5) were retained. TF-target Pearson correlation (*r*) was then calculated for each retained module in the cancer of interest and in the rest of the Pan-Cancer data, modules with negative correlation values were removed and correlation coefficients were z-transformed (zCor) using Fisher transformation to ensure their normal distribution for subsequent analyses. A zCor threshold to separate the cancer of interest from the rest of Pan-Cancer was then computed as the Youden Index [83] of the whole zCor values (confidence interval = 0.95), all modules below the threshold were removed and only the remaining modules for which zCor in the cancer of interest is >zCor in Pan-Cancer were retained. For each matrisome gene with >1 TF in the resulting model, then, gene expression values of any TF-target pair were regressed using adaptive lasso regression [84] and only the modules whose coefficients were not shrunk were kept (adaptive lasso pruning). The results of the above procedure (“model 1”) were then passed in parallel to two further inference schemes, namely sparse Bayesian network [85] (“model 2”) and mixed graphical model [86] (“model 3”), each with 10× cross-validation. Finally, results from the models were compared and only modules present in at least two models were retained and reported in the study (Suppl Table 6). The final consensus pipeline yielded networks that are, on average, 27% the size of the original models based on data mining only, thus eliminating >70% of all mined interactions (data not shown, available on request to the Authors). Cross-validation of results into THPA was possible for 22 types of tumors (Suppl Table 14). For these, TFs and targets from each module were checked against THPA staining profiles and each single protein was considered as cross-validated if Percentile Positive Staining (PPS, % of assayed samples that show “High” or “Medium” staining for a given TF or target) > 0. All heatmaps were generated using the pheatmap package v1.0.1, using Pearson correlation (*r*) as the distance metrics for hierarchical clustering.

For each tumor, a “master regulator” layer (cancer driver genes interacting with the TFs identified) was built upon the module structure as follows. Pan-Cancer drivers [44] were filtered so that, for each cancer, their frequency of mutation in the tumor of interest was > average frequency of mutation of the same driver in the whole Pan-Cancer cohort. Cancer-specific drivers [44] were filtered so that their frequency of mutation in the tumor of interest was >5%, well above the lower limit of intermediate-frequency drivers [44] and the Pan-Cancer frequency of mutation in drivers (4.16%). Next, Pan-Cancer and cancer-specific drivers passing the filtering stage were searched for physical interaction with the TFs identified in the given cancer against the BioGrid database, and only drivers interacting with >10% of all TFs (corresponding to the top 50% of the mined drivers) were considered true “master regulators” and retained (Suppl Table 8). This procedure yielded 380 unique protein-protein physical interactions covering all the 347 master regulator-TF interactions identified across the different tumors. Of these, 41.05% were Affinity Capture Western, 20.79% Reconstituted Complex, 11.84% Two-hybrid, 6.32% Biochemical Activity, 5.26% Affinity Capture MS, 3.95% Co-localization, 2.11% Protein-peptide, 1.84% Co-crystal Structure, 1.58% Affinity Capture Luminescence, 1.32% Co-fractionation, 0.79% Co-purification, 0.79% Far Western, 0.79% FRET, 0.79% Synthetic Lethality, 0.53% PCA and 0.26% Phenotypic Enhancement. Furthermore, at the end of the pipeline, the weighted average of TFs interacting with each master was 7.96 (ranging from 5.7 in the lower quartile to 36.7 in the upper), in line with the values reported by [45]. Contingency of Pan-Cancer and cancer-specific regulators per each tumor was analyzed using the Stats package v3.6.0, Chi-square test with Yates continuity correction. The overall regulatory infrastructure above matrisome genes (TFs and master regulators) was then enriched for KEGG pathways using StringDB. Total results are restricted to the pathways shown in Fig. 3 for clarity. To infer the most important regulators at the cluster level, the lists of master regulators from each member of the cluster were first intersected, entries not common to all cluster members discarded and linear support vector machine (LSVM) analysis was performed setting epsilon: 0.1, lambda: 0.1, L2 penalty function, predictor importance calculation (for feature extraction) and 10× cross-validation (80% training, 20% test) *via* random sample permutation. Gene expression of extracted predictors was used to classify digestive, lung and brain neoplasms into the appropriate cluster *vs.* rest of the 22 tumors and Area under the curve (AUC) of the Receiver Operating Characteristic (ROC) graph was performed in the ROCR package v1.0-7. All heatmaps were generated using the pheatmap package v1.0.1, using Pearson correlation (*r*) as the distance metrics for hierarchical clustering.

### Prognostic value and druggability of matrisome modules

For each cancer type for which module analysis was possible, we then evaluated the association of module activity with overall patient survival and the possibilities for therapeutic interventions. For survival, monovariate analysis (Kaplan-Meier method, Log-Rank test) was performed using the survminer package v0.4.3 and the survival package v2.42-6. To this aim, gene expression of TF and matrisome gene of each module were averaged together and all individuals of any cohort were dichotomized into “high” (above the average) or “low” (below the average) expression of any module identified. For each module in each cohort, finally, survival analysis was performed as above. To evaluate druggability, the whole matrisome architecture (proper matrisome genes + TFs + master regulators) of each cancer type was searched for drugs and therapeutic approaches against DGIdb and all entries without an associated mechanism were removed. From these lists, FDA-approved drugs [80] were deduced and divided into drugs targeting the master regulators, TFs or the matrisome proper, and whether their suggested use was on-(FDA-approved for the given cancer) or off-(not FDA-approved for the given cancer) label.

## Competing interests

The authors declare no conflict of interests.

## Appendix A Supplemental data

Supplemental material Image 1
