# Supplementary material for: Pan-Cancer analysis of the expression and regulation of matrisome genes across 32 tumor types
Source: Matrix Biol Plus. 2019 Apr 6;1:100004. doi: 10.1016/j.mbplus.2019.04.001 (PMC7852311; doi:10.1016/j.mbplus.2019.04.001)
Supplement: Supplementary file 1 — Supplemental material [file mmc1.pdf]

Pan-Cancer analysis of the expression and regulation of matrisome genes across 32 tumor types

Valerio Izzi, Juho Lakkala, Raman Devarajan, Anni Kääriäinen, Jarkko Koivunen, Ritva Heljasvaara and Taina Pihlajaniemi

SUPPLEMENTAL DATA

Supplemental data for this manuscript include 17 figures and 14 tables

Izzi et al Supplemental Figure 1

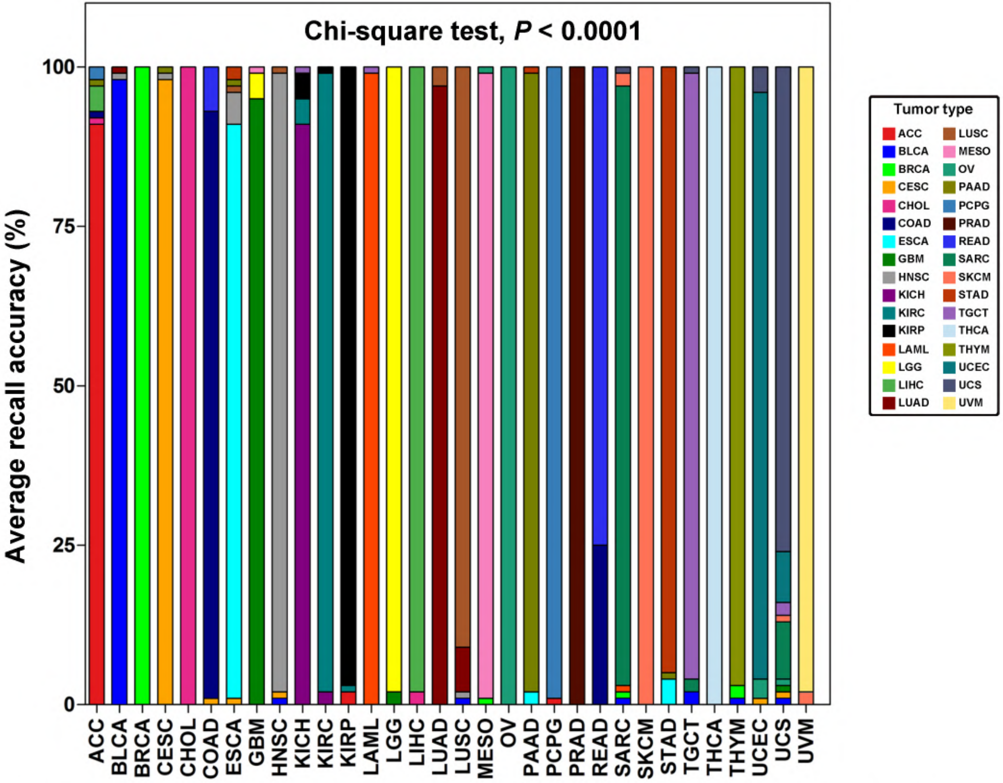

Supplemental Figure 1. Average recall accuracy of cross-tumor classification using matrisome gene expression. 3 independent classification algorithms (SVM, NN and C5.0) were used to classify each patient into one of the 32 possible tumor types, the assignement being repeated 100 times randomly per each algorithm. The recall accuracy (percentile of properly and missclassified cases) was then calculated as the average of all tests.

Izzi et al Supplemental Figure 2

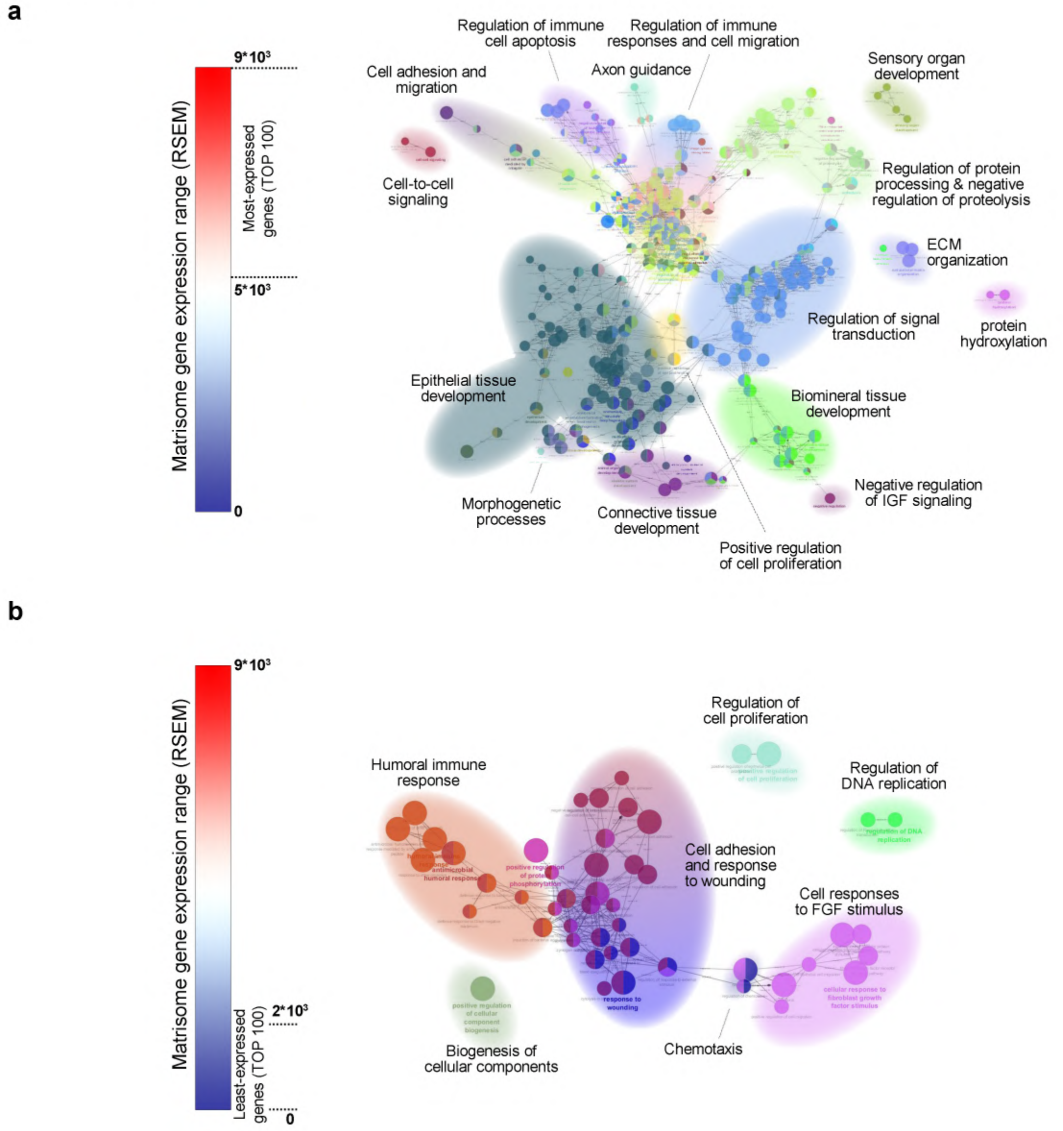

Supplemental Data Figure 2. Gene ontology enrichment of the most- and least-expressed matrisome genes. Network-based gene ontology (GO) enrichment for biological processes of the 100 most and least expressed matrisome genes for each tumor type. Results were calculated using only experimentally-validated evidence and plotted using the ClueGO plugin for Cytoscape. (**a**) Most expressed and (**b**) least expressed genes. The expression range for the genes in (a) and (b) is reported.

Izzi et al Supplemental Figure 3

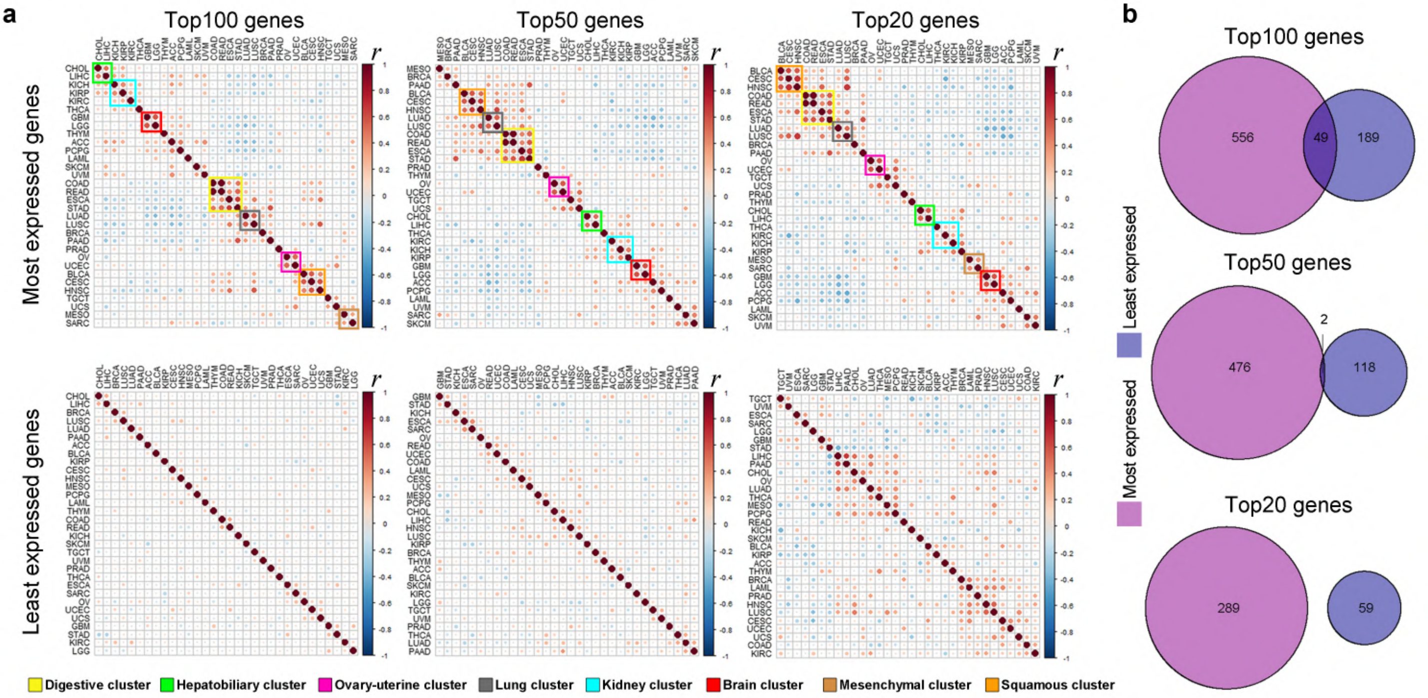

Supplemental Figure 3. Upregulated matrisome genes identify functional clusters of tumors. (**a**) Inter-tumor correlation (Pearson correlation (*r*)) based on the 100, 50 or 20 most- and least-expressed matrisome genes in each tumor type. Clusters of highly correlated tumor types, only observable by most expressed (upper panel) genes, are reported as colored squares. (**b**) Overlap between most and least expressed matrisome genes in different tumor types at different amount of top genes sampled.

Izzi et al Supplemental Figure 4

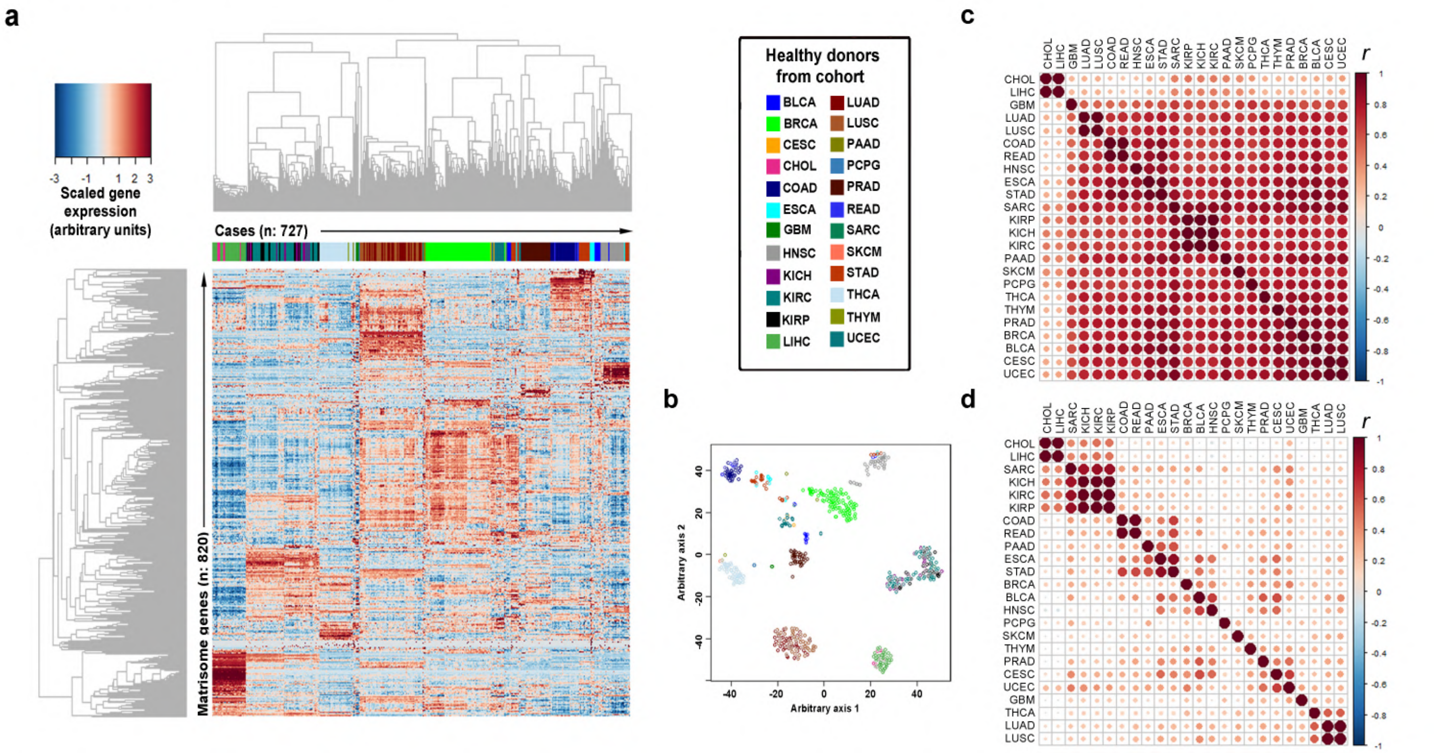

Supplemental Figure 4. Expression of matrisome genes in healthy donor tissues from The Cancer Genome Atlas (TCGA) shows overall higher inter-tissue similarities. Expression of the same matrisome genes as in the tumor samples in 727 cases from 24 of the 32 tumor cohorts analyzed. (**a**) Heatmap of gene expression and, (**b**) multidimensional scaling of the data according to t-SNE. (**c**,**d**) Correlation (Pearson correlation (*r*)) between the different sample types based on the 100 most (**c**) and least expressed (**d**) matrisome genes.

Izzi et al Supplemental Figure 5

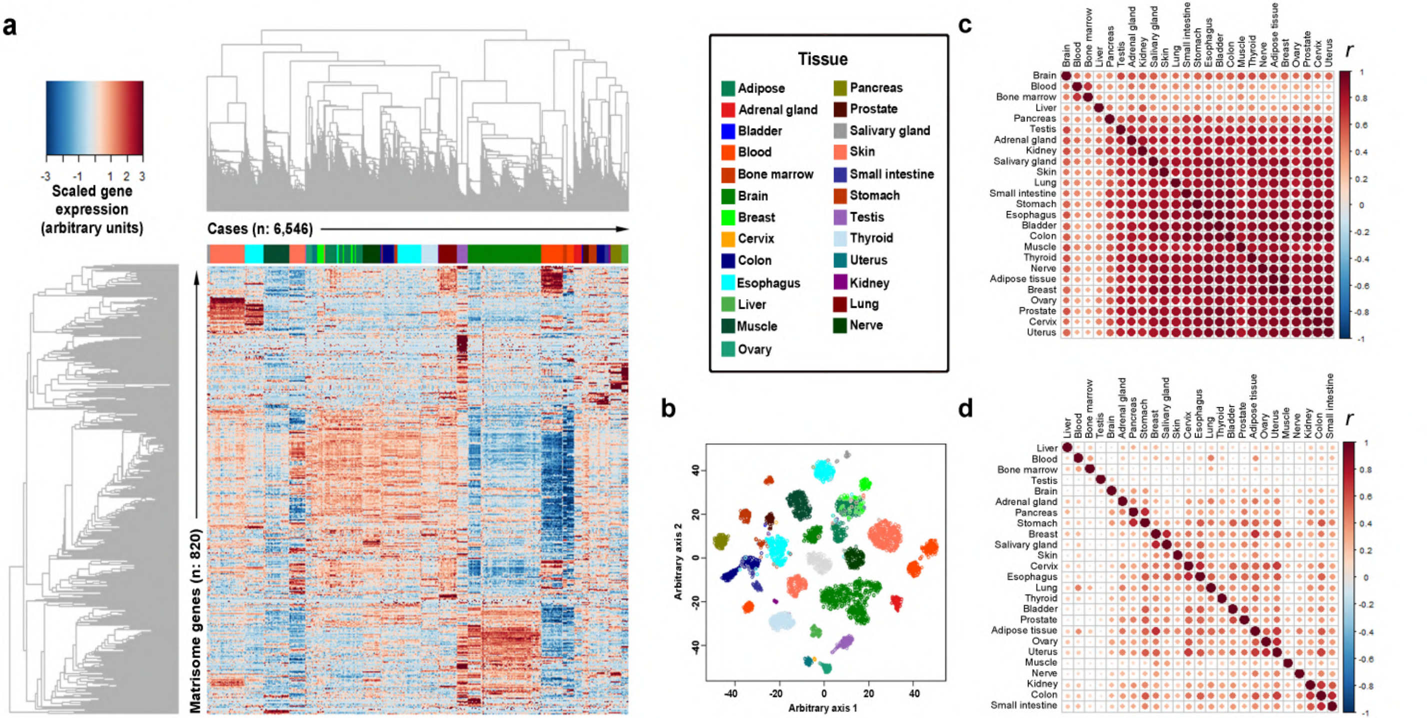

Supplemental Figure 5. Expression of matrisome genes in healthy donor tissues from the Genotype-Tissue Expression (GTEx) database shows overall higher inter-tissue similarities. Expression of the same matrisome genes as in the tumor samples in 6,546 cases from 25 healthy tissues, anatomically comparable to the tumor sites. (**a**) Heatmap of gene expression and, (**b**) multidimensional scaling of the data according to t-SNE. (**c**,**d**) Correlation (Pearson correlation (*r*)) between the different sample types based on the 100 most (**c**) and least expressed matrisome genes (**d**).

Izzi et al Supplemental Figure 6

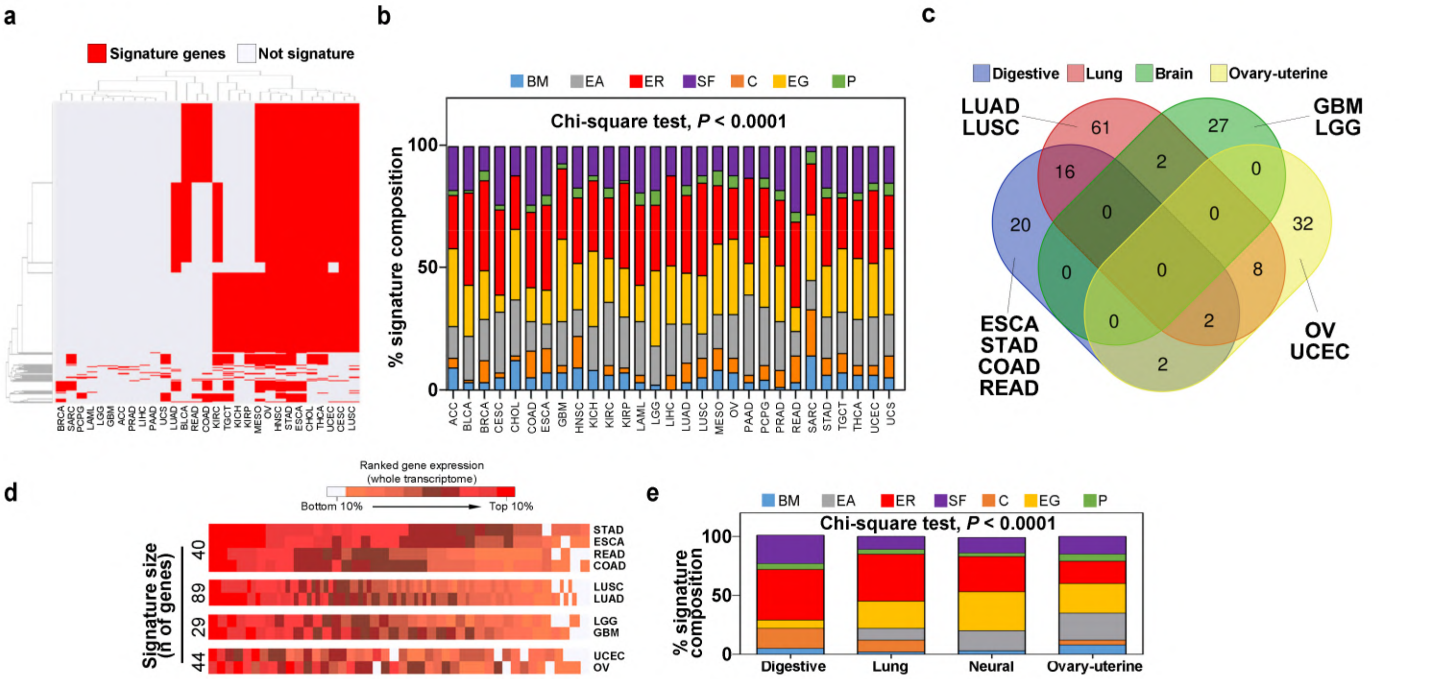

Supplemental Figure 6. Tumor matrisome signatures. (a) Binary heatmap of gene expression in each tumor matrisome signature. Red dots indicate signature genes (genes whose expression is significantly higher in the given tumor in respect to other tumors and comparable healthy tissue). (**b**) % composition of each signature in terms of matrisome categories. (**c**) Overlap between cluster signatures, each obtained by the intersection of the single signature belonging to it (see also Extended Data Figure 7). (**d**) Ranked gene expression (centiles) of signature genes in tumors from the same cluster and (**e**) % composition of each cluster signature in terms of matrisome categories. Abbreviations in (b) and (e): BM, basement membranes; C, collagens; EA, ECM-affiliated; EG, ECM glycoproteins; ER, ECM regulators; P, proteoglycans; SF, secreted factors.

Izzi et al Supplemental Figure 7

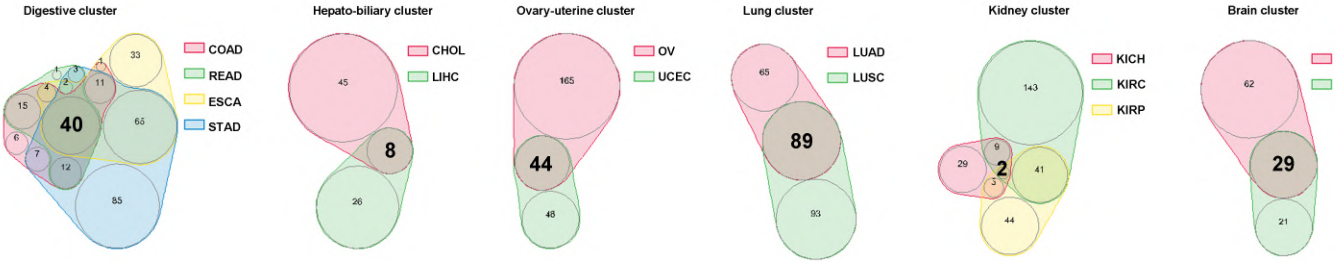

Supplemental Figure 7. Intersection of individual tumor matrisome signatures to generate cluster-level signatures. Each individual tumor matrisome signature was intersected with all other signatures from tumors in the same cluster. Genes common to all members of the cluster (bold) were considered as constituents of cluster-level signatures.

Izzi et al Supplemental Figure 8

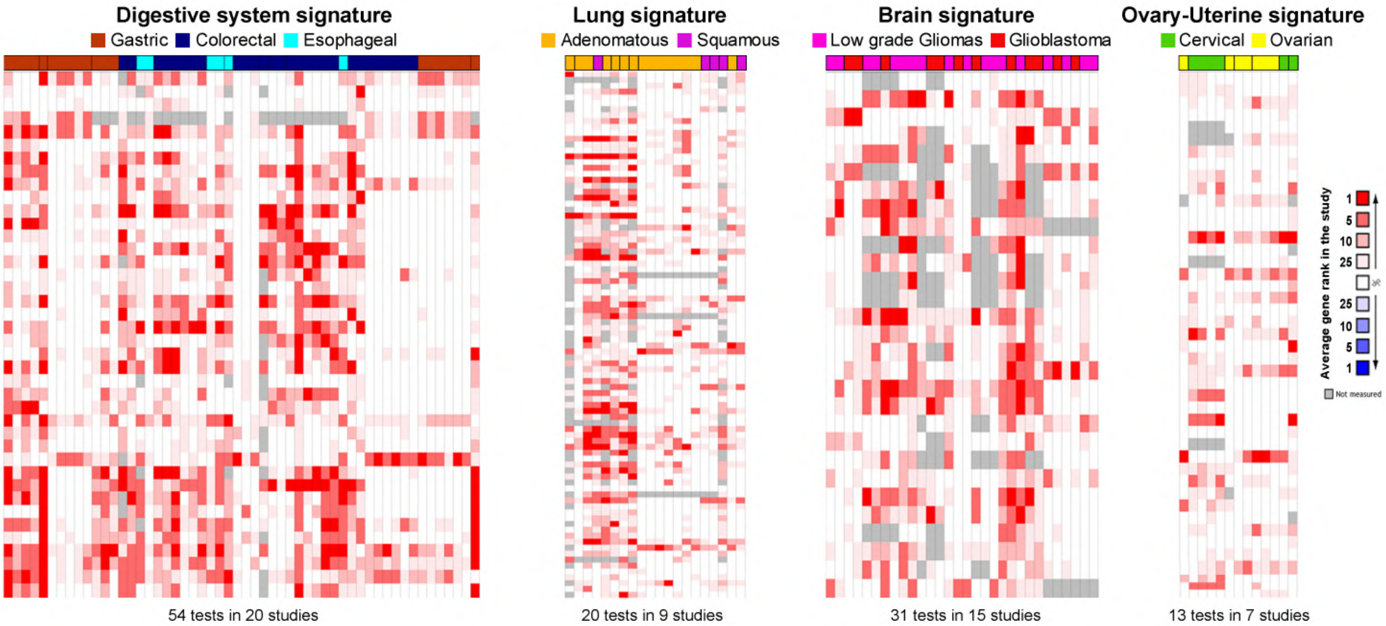

Supplemental Figure 8. Tumor cluster-level matrisome signatures. Cross-validation of cluster signatures by ranking of their components in different cohorts *vs.* respective healthy samples. Each column represents a test (tumor *vs.* healthy, order is as in Supplemental Table 13), and the row a gene (order is as in Supplemental Table 5). Red indicates that the given matrisome gene has higher ranking in tumor than in normal, blue that the given matrisome gene has lower ranking in tumor than in normal, white indicates no change and grey no values for the gene. Data are from *Oncomine* meta-analysis engine.

Izzi et al Supplemental Figure 9

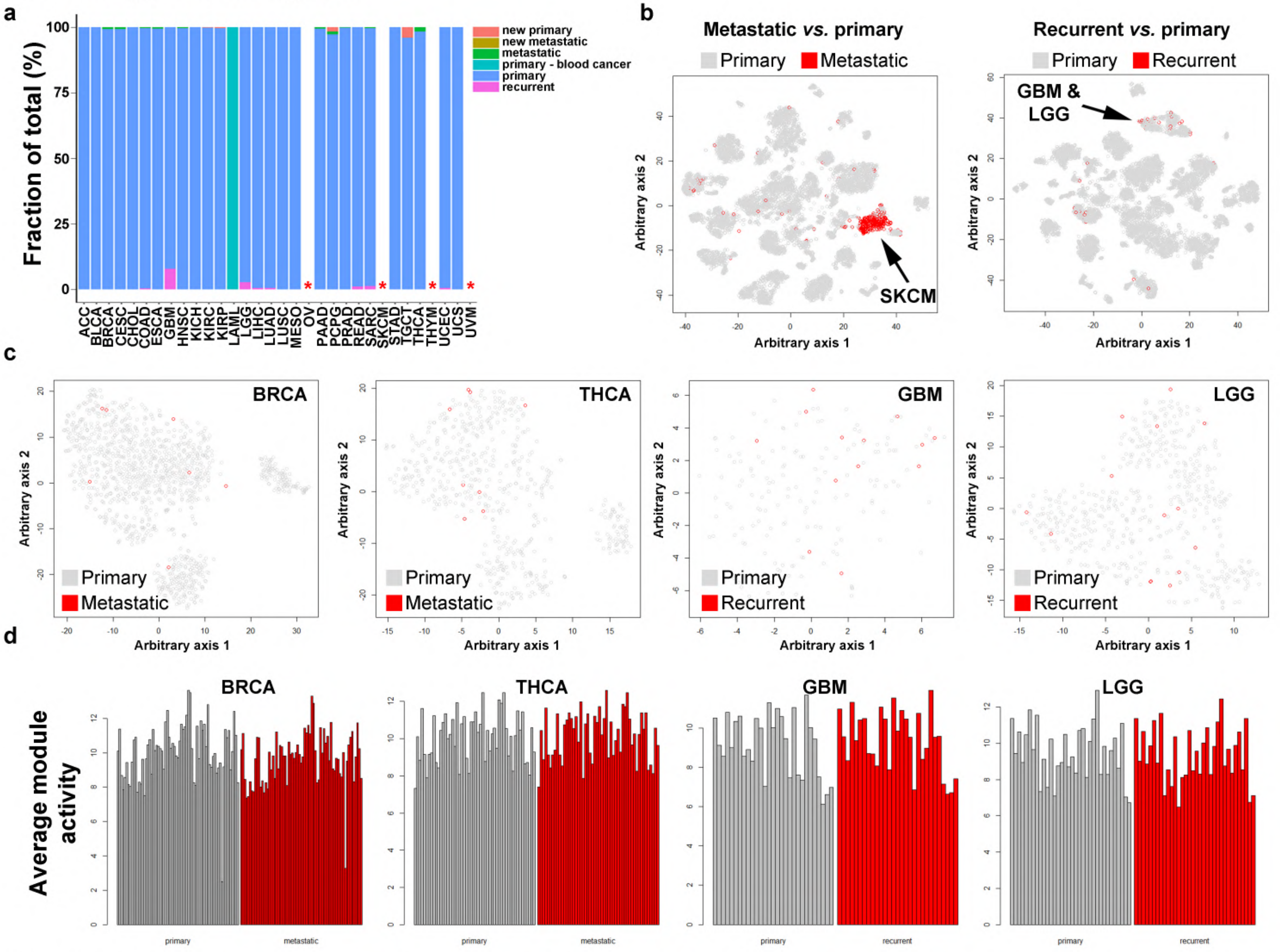

Supplemental Figure 9. Comparison of matrisome and modules' expression in primary and metastatic or recurrent tumors. (**a**) Frequency (%) of primary, metastatic and recurrent tumor samples per neoplasm. Neoplasms which were not modelled for transcriptional regulation are marked with a red asterisk. (**b**) t-SNE analysis of all patients shows that the majority of metastatic samples accumulate in SKCM (excluded from later transcriptional modelling) while recurrent samples enrich GBM and LGG (included in transcriptional analysis). Overall and (**c**) in selected examples, no metastatic or recurrent sample diverged notably from primary samples for matrisome gene expression. (**d**) Similar average module activity profiles for primary and metastatic BRCA and THCA samples or primary and recurrent GBM and LGG samples.

Izzi et al Supplemental Figure 10

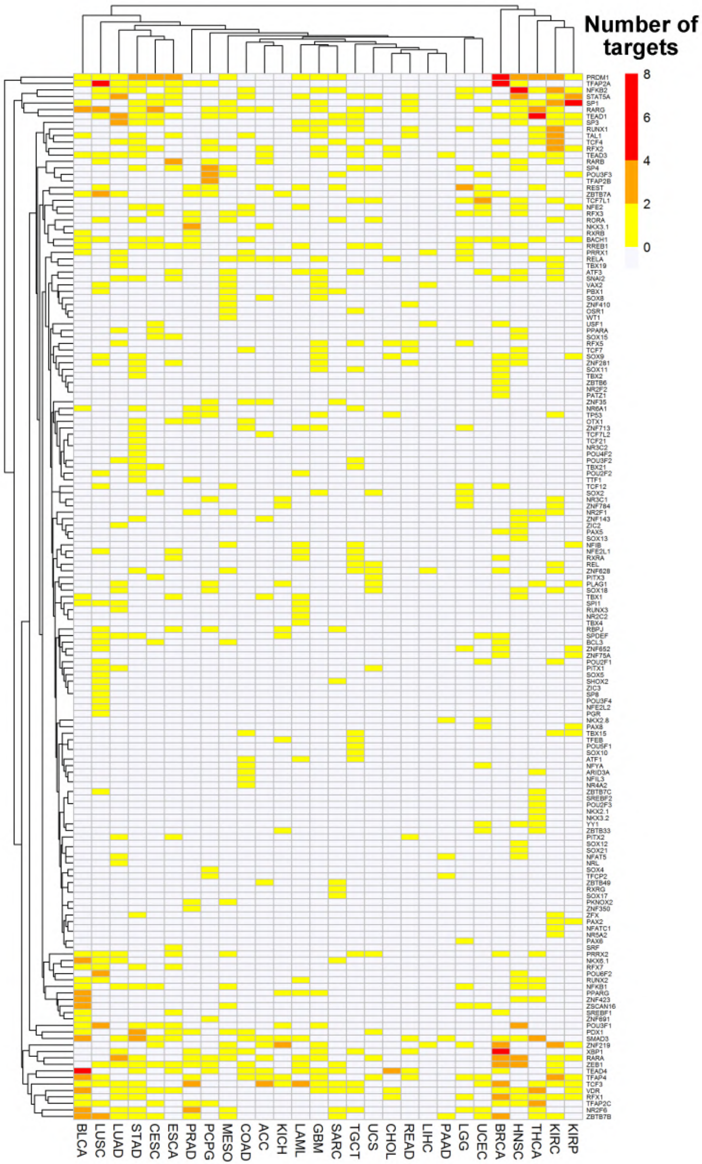

Supplemental Figure 10. Transcription factors in the matrisome modules. Heatmap of the number of trascriptional matrisome targets per each transcription factor (TF) in the modules identified in this study.

Izzi et al Supplemental Figure 11

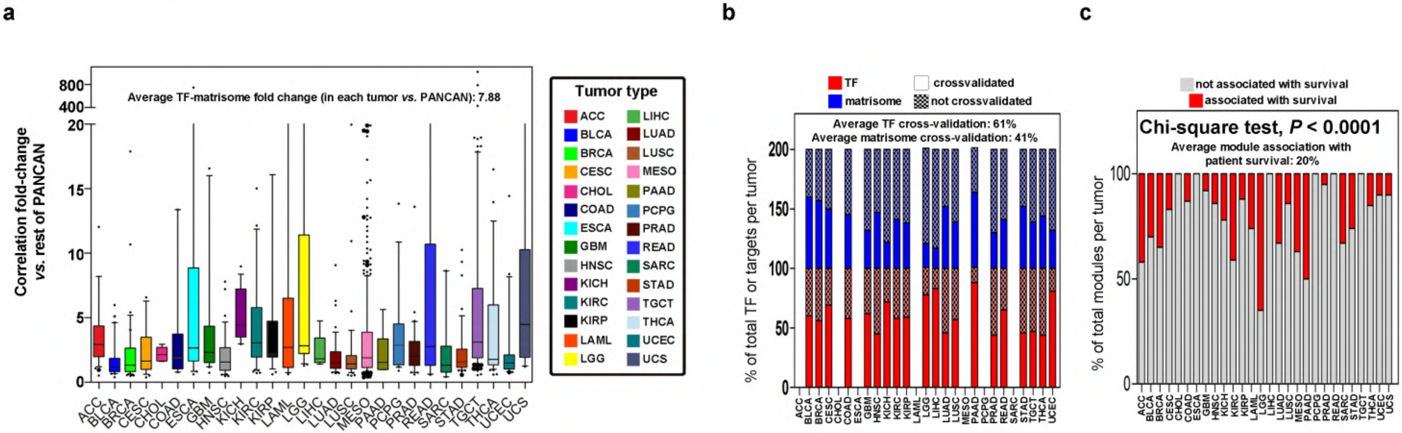

ESupplemental Figure 11. Features of the matrisome modules. (**a**) TF-matrisome correlation (Pearson *r*) in any given tumor's module *vs*. the same TF-matrisome pair in the rest of the PANCAN cohort. Values are expressed as fold-change in each tumor *vs*. the rest of the Pan-Cancer (PANCAN) cohort. (**b**) % Cross-validation of TF and targets from each module in matching samples from The Human Protein Atlas (% number of TF or matrisome genens with "High" or "Medium" staining), and (**c**) % frequency of modules associated with differential patient survival in any given tumor.

Izzi et al Supplemental Figure 12

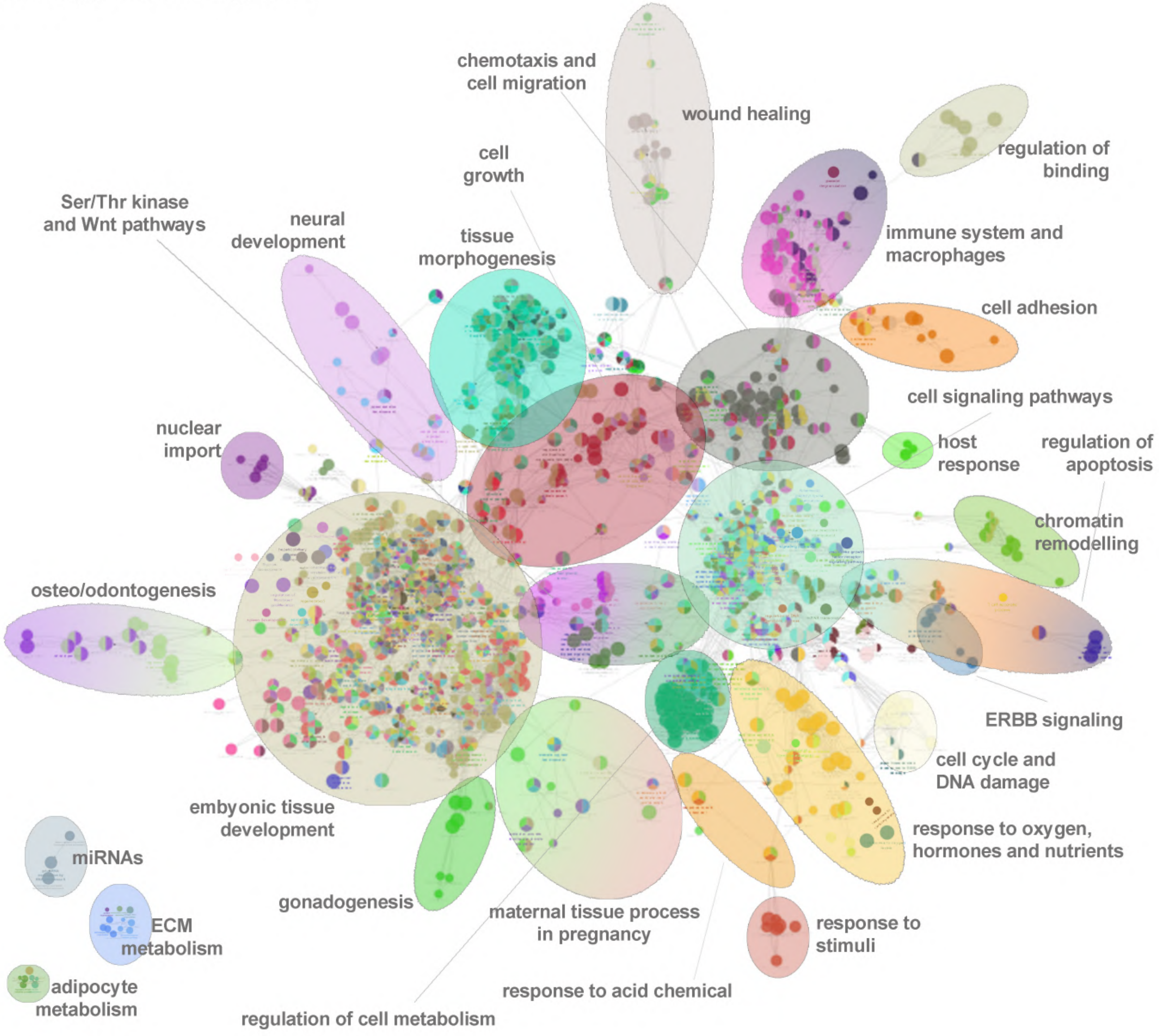

Supplemental Figure 12. Gene ontology enrichment of the whole matrisome modules and their master regulators. Network-based gene ontology (GO) enrichment for biological processes of the totality of modules and master regulators. Results were calculated and plotted using the ClueGO plugin for Cytoscape using only experimentally validated interactions, an enrichment threshold *P* < 0.05 evidence and GO term fusion. Onthological overlays are manually drawn and annotated.

Izzi et al Supplemental Figure 13

a

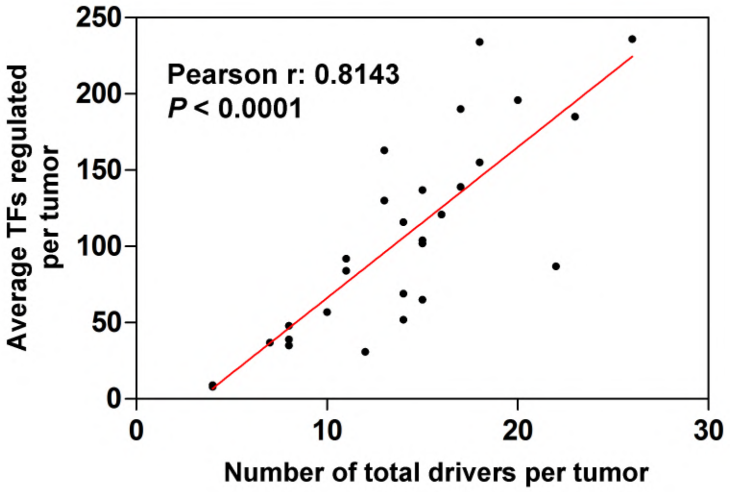

b

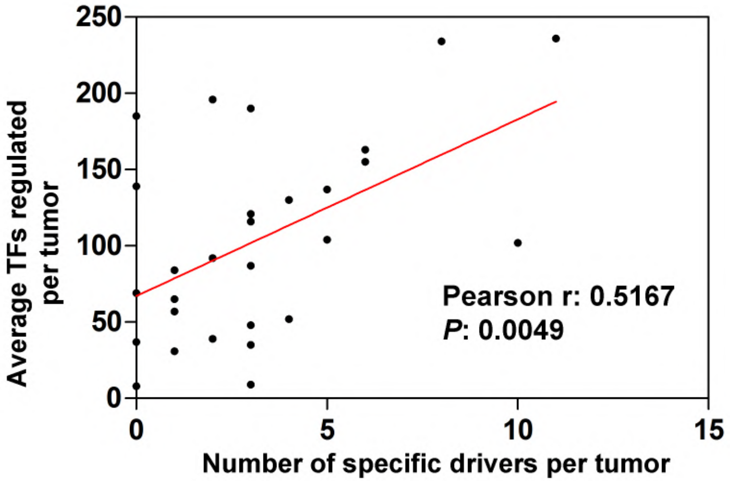

Supplemental Figure 13. Correlations between master regulators and matrisome-controlling transcription factors (TF). The number of cancer drivers in any given tumor correlated linearly with the number of matrisome-controlling TFs identified in the same tumor, either when the total number of drivers (**a**) or only the cancer-specific drivers (**b**) were considered.

Izzi et al Supplemental Figure 14

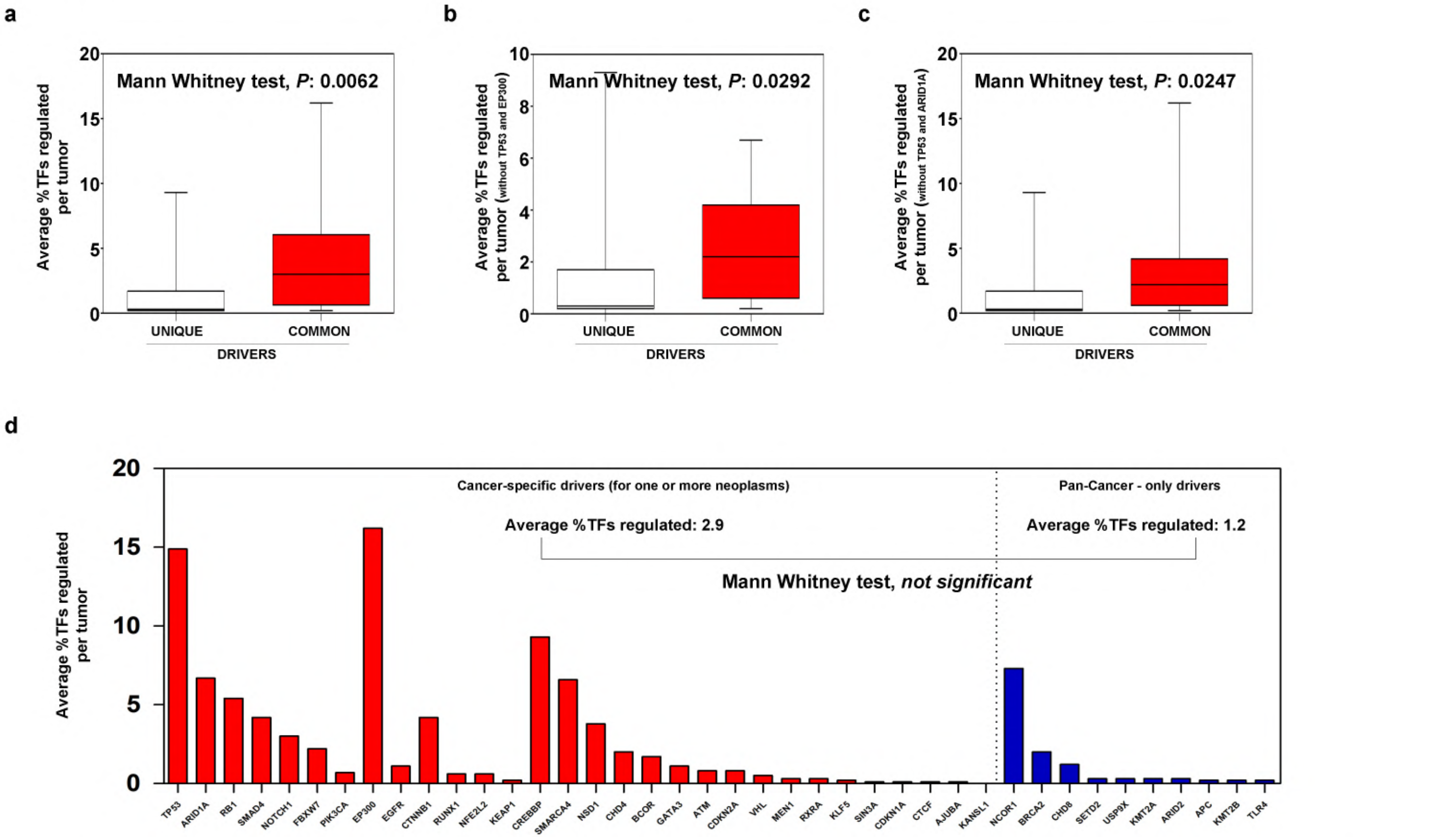

Supplemental Figure 14. Modular structure of the cancer matrisome at the master regulator level. Cancer driver genes shared by at least two tumor types (”common”) are (**a**) hubs in the cancer matrisome networks, regulating more TFs (in percentile) than the drivers active in one tumor type only (“unique”), even when (**b**) most common drivers (*TP53* and *EP300*) or (**c**) most recurrent cancer-specific drivers (*TP53* and *ARID1A*) were excluded from the analysis. (**d**) Cancer-specific drivers also regulated more TFs that Pan-Cancer - only drivers (those that are not found as specific drivers for any of the neoplasms studied).

Izzi et al Supplemental Figure 15

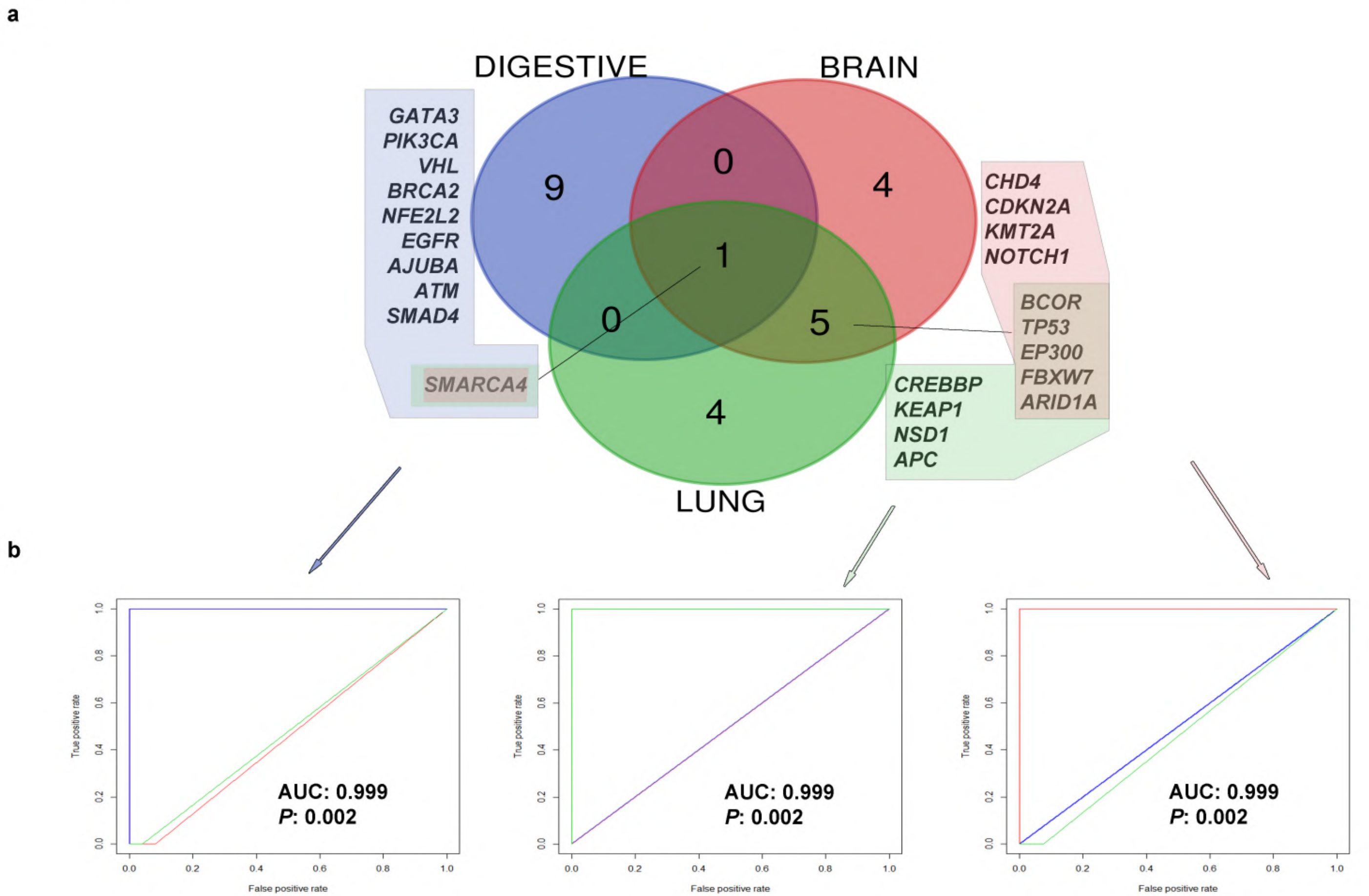

Supplemental Figure 15. Specificity of the master regulators at the cluster level. (**a**) Most important matrisome-regulating cancer drivers for digestive (ESCA, STAD, COAD and READ), brain (GBM and LGG) or lung (LUAD and LUSC) clusters extracted by Linear Support Vector Machine (LSVM) and their overlap. (**b**) Area under the curve (AUC) of the Receiver Operating Characteristic (ROC) graph for each group of master regulators, evaluating the ability of the given set of driver genes to correctly score the cluster of pertinence of the set. Note that colors follow the scheme in (a).

Izzi et al Supplemental Figure 16

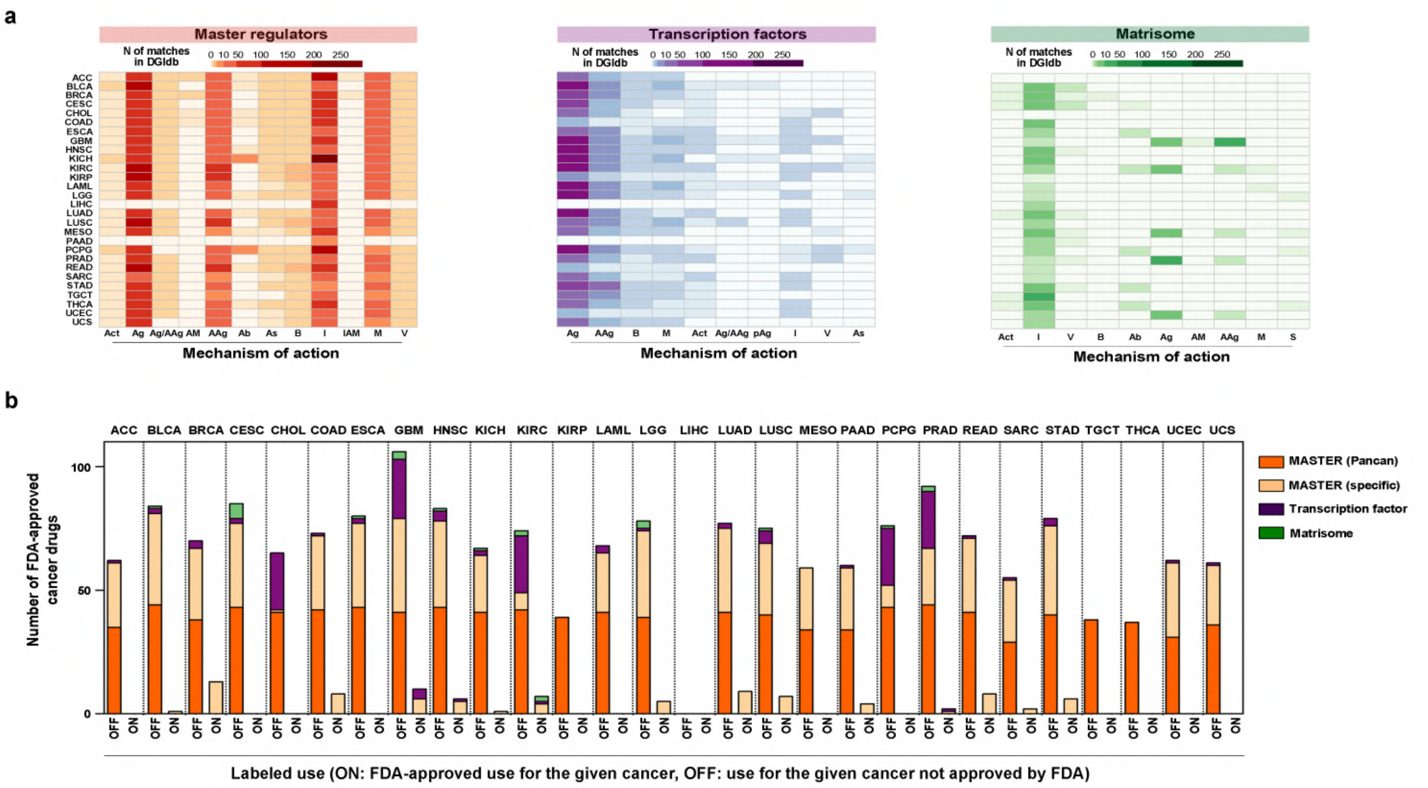

Supplemental Figure 16. Druggability of the cancer matrisome. (**a**) Identification of potential therapeutic approaches (e.g., drugs, small molecules, RNA interference, etc.) targeting the master regulators (left), transcription factors (middle) and the 28 matrisome signatures (right) identified in this study. Abbreviations: Act, activator; Ag, agonist; AAg, antagonist; Ag/AAg, agonist/antagonist; AM, allosteric modulator; Ab, antibody; As, antisense; B, binder; I, inhibitor; IAM, inhibitor/allosteric modulator; M, modulator; V, vaccine; pAg, partial agonist. (**b**) Number of FDA-approved cancer drugs with the potential to target master regulators, transcription factors and the matrisome proper, either used on-label (drug approved for the given cancer) or off-label (drug not approved for the given cancer).

Izzi et al Supplemental Figure 17

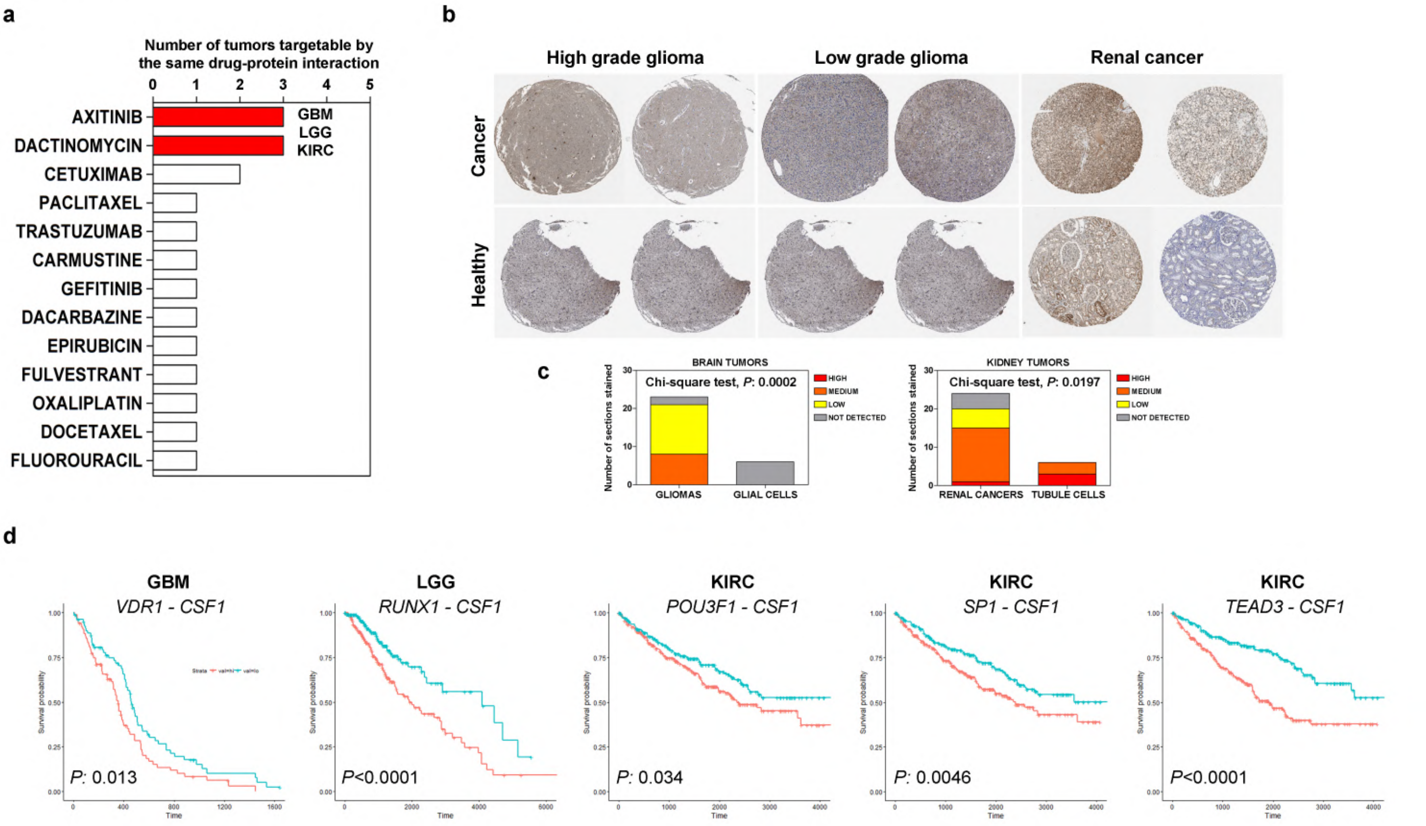

Supplemental Figure 17. Targeting the cancer matrisome: the example of macrophage colony-stimulating factor 1 (CSF1). Drugs with the same target and mechanism of action in different tumors indicate favorable multi-cancer drug targets. (**a**) Considering in example only the drugs acting on actual matrisome proteins, Axitinib and Dactinomycin are found in the lists of glioblastoma (GBM), lower-grade glioma (LGG) and kidney clear cell carcinoma (KIRC). (**b**) Upregulation of CSF1 receptor (CSF1R) in brain and kidney tumors from The Human Protein Atlas and, (**c**) increased expression of CSF1R in indicated tumors *vs.* matching healthy tissues. Note that antibody staining for CSF1 has not yet been performed for the same sections. (**d**) Overall survival (days) of patients with above ("high") or below ("low") the cohort's average expression of *TF-CSF1* modules.

Supplemental Table 1. Composition of the Pan-Cancer cohort

Tumor type	Abbreviation	System of origin	Number of patients
Adrenocortical cancer	ACC	Endocrine	77
Bladder urothelial carcinoma	BLCA	Excretory	426
Breast invasive carcinoma	BRCA	Reproductive	1212
Cervical and endocervical cancer	CESC	Reproductive	309
Cholangiocarcinoma	CHOL	Digestive	45
Colon adenocarcinoma	COAD	Digestive	331
Esophageal carcinoma	ESCA	Digestive	195
Glioblastoma multiforme	GBM	Neurosensory	171
Head and neck squamous cell carcinoma	HNSC	Tegument	564
Kidney chromophobe cell carcinoma	KICH	Excretory	91
Kidney clear cell carcinoma	KIRC	Excretory	603
Kidney papillary cell carcinoma	KIRP	Excretory	321
Acute myeloid leukemia	LAML	Hematopoietic	173
Brain lower grade glioma	LGG	Neurosensory	523
Liver hepatocellular carcinoma	LIHC	Digestive	421
Lung adenocarcinoma	LUAD	Respiratory	574
Lung squamous cell carcinoma	LUSC	Respiratory	548
Mesothelioma	MESO	Respiratory	87
Ovarian serous cystadenocarcinoma	OV	Reproductive	427
Pancreatic adenocarcinoma	PAAD	Digestive	183
Pheochromocytoma and paraganglioma	PCPG	Endocrine	185
Prostate adenocarcinoma	PRAD	Reproductive	548
Rectum adenocarcinoma	READ	Digestive	103
Sarcoma	SARC	Other	264
Skin cutaneous melanoma	SKCM	Tegument	470
Stomach adenocarcinoma	STAD	Digestive	450
Testicular germ cell tumor	TGCT	Reproductive	154
Thyroid carcinoma	THCA	Endocrine	571
Thymoma	THYM	Hematopoietic	121
Uterine corpus endometrioid carcinoma	UCEC	Reproductive	204
Uterine carcinosarcoma	UCS	Reproductive	57
Uveal melanoma	UVM	Neurosensory	79

Supplemental Table 2. Matrisome-based tumor classification by 3 independent algorithms

Support Vector Machine (SVM)

	ACC	BLCA	BRCA	CESC	CHOL	COAD	ESCA	GBM	HNSC	KICH	KIRC	KIRP	LAML	LGG	LIHC	LUAD	LUSC	MESO	OV	PAAD	PCPG	PRAD	READ	SARC	SKCM	STAD	TGCT	THCA	THYM	UCEC	UCS	UVM
ACC	100 %	0 %	0 %	0 %	0 %	0 %	0 %	0 %	0 %	0 %	0 %	0 %	0 %	0 %	0 %	0 %	0 %	0 %	0 %	0 %	0 %	0 %	0 %	0 %	0 %	0 %	0 %	0 %	0 %	0 %	0 %	0 %
BLCA	0 %	100 %	0 %	0 %	0 %	0 %	0 %	0 %	0 %	0 %	0 %	0 %	0 %	0 %	0 %	0 %	0 %	0 %	0 %	0 %	0 %	0 %	0 %	0 %	0 %	0 %	0 %	0 %	0 %	0 %	0 %	0 %
BRCA	0 %	0 %	100 %	0 %	0 %	0 %	0 %	0 %	0 %	0 %	0 %	0 %	0 %	0 %	0 %	0 %	0 %	0 %	0 %	0 %	0 %	0 %	0 %	0 %	0 %	0 %	0 %	0 %	0 %	0 %	0 %	0 %
CESC	0 %	0 %	0 %	100 %	0 %	0 %	0 %	0 %	0 %	0 %	0 %	0 %	0 %	0 %	0 %	0 %	0 %	0 %	0 %	0 %	0 %	0 %	0 %	0 %	0 %	0 %	0 %	0 %	0 %	0 %	0 %	0 %
CHOL	0 %	0 %	0 %	0 %	100 %	0 %	0 %	0 %	0 %	0 %	0 %	0 %	0 %	0 %	0 %	0 %	0 %	0 %	0 %	0 %	0 %	0 %	0 %	0 %	0 %	0 %	0 %	0 %	0 %	0 %	0 %	0 %
COAD	0 %	0 %	0 %	0 %	0 %	100 %	0 %	0 %	0 %	0 %	0 %	0 %	0 %	0 %	0 %	0 %	0 %	0 %	0 %	0 %	0 %	0 %	25 %	0 %	0 %	0 %	0 %	0 %	0 %	0 %	0 %	0 %
ESCA	0 %	0 %	0 %	0 %	0 %	0 %	100 %	0 %	0 %	0 %	0 %	0 %	0 %	0 %	0 %	0 %	0 %	0 %	0 %	0 %	0 %	0 %	0 %	0 %	0 %	0 %	0 %	0 %	0 %	0 %	0 %	0 %
GBM	0 %	0 %	0 %	0 %	0 %	0 %	0 %	100 %	0 %	0 %	0 %	0 %	0 %	0 %	0 %	0 %	0 %	0 %	0 %	0 %	0 %	0 %	0 %	0 %	0 %	0 %	0 %	0 %	0 %	0 %	0 %	0 %
HNSC	0 %	0 %	0 %	0 %	0 %	0 %	0 %	0 %	100 %	0 %	0 %	0 %	0 %	0 %	0 %	0 %	0 %	0 %	0 %	0 %	0 %	0 %	0 %	0 %	0 %	0 %	0 %	0 %	0 %	0 %	0 %	0 %
KICH	0 %	0 %	0 %	0 %	0 %	0 %	0 %	0 %	0 %	100 %	0 %	0 %	0 %	0 %	0 %	0 %	0 %	0 %	0 %	0 %	0 %	0 %	0 %	0 %	0 %	0 %	0 %	0 %	0 %	0 %	0 %	0 %
KIRC	0 %	0 %	0 %	0 %	0 %	0 %	0 %	0 %	0 %	0 %	100 %	0 %	0 %	0 %	0 %	0 %	0 %	0 %	0 %	0 %	0 %	0 %	0 %	0 %	0 %	0 %	0 %	0 %	0 %	0 %	0 %	0 %
KIRP	0 %	0 %	0 %	0 %	0 %	0 %	0 %	0 %	0 %	0 %	0 %	100 %	0 %	0 %	0 %	0 %	0 %	0 %	0 %	0 %	0 %	0 %	0 %	0 %	0 %	0 %	0 %	0 %	0 %	0 %	0 %	0 %
LAML	0 %	0 %	0 %	0 %	0 %	0 %	0 %	0 %	0 %	0 %	0 %	0 %	100 %	0 %	0 %	0 %	0 %	0 %	0 %	0 %	0 %	0 %	0 %	0 %	0 %	0 %	0 %	0 %	0 %	0 %	0 %	0 %
LGG	0 %	0 %	0 %	0 %	0 %	0 %	0 %	0 %	0 %	0 %	0 %	0 %	0 %	100 %	0 %	0 %	0 %	0 %	0 %	0 %	0 %	0 %	0 %	0 %	0 %	0 %	0 %	0 %	0 %	0 %	0 %	0 %
LIHC	0 %	0 %	0 %	0 %	0 %	0 %	0 %	0 %	0 %	0 %	0 %	0 %	0 %	0 %	100 %	0 %	0 %	0 %	0 %	0 %	0 %	0 %	0 %	0 %	0 %	0 %	0 %	0 %	0 %	0 %	0 %	0 %
LUAD	0 %	0 %	0 %	0 %	0 %	0 %	0 %	0 %	0 %	0 %	0 %	0 %	0 %	0 %	0 %	100 %	0 %	0 %	0 %	0 %	0 %	0 %	0 %	0 %	0 %	0 %	0 %	0 %	0 %	0 %	0 %	0 %
LUSC	0 %	0 %	0 %	0 %	0 %	0 %	0 %	0 %	0 %	0 %	0 %	0 %	0 %	0 %	0 %	0 %	100 %	0 %	0 %	0 %	0 %	0 %	0 %	0 %	0 %	0 %	0 %	0 %	0 %	0 %	0 %	0 %
MESO	0 %	0 %	0 %	0 %	0 %	0 %	0 %	0 %	0 %	0 %	0 %	0 %	0 %	0 %	0 %	0 %	0 %	100 %	0 %	0 %	0 %	0 %	0 %	0 %	0 %	0 %	0 %	0 %	0 %	0 %	0 %	0 %
OV	0 %	0 %	0 %	0 %	0 %	0 %	0 %	0 %	0 %	0 %	0 %	0 %	0 %	0 %	0 %	0 %	0 %	0 %	100 %	0 %	0 %	0 %	0 %	0 %	0 %	0 %	0 %	0 %	0 %	0 %	0 %	0 %
PAAD	0 %	0 %	0 %	0 %	0 %	0 %	0 %	0 %	0 %	0 %	0 %	0 %	0 %	0 %	0 %	0 %	0 %	0 %	0 %	100 %	0 %	0 %	0 %	0 %	0 %	0 %	0 %	0 %	0 %	0 %	0 %	0 %
PCPG	0 %	0 %	0 %	0 %	0 %	0 %	0 %	0 %	0 %	0 %	0 %	0 %	0 %	0 %	0 %	0 %	0 %	0 %	0 %	0 %	100 %	0 %	0 %	0 %	0 %	0 %	0 %	0 %	0 %	0 %	0 %	0 %
PRAD	0 %	0 %	0 %	0 %	0 %	0 %	0 %	0 %	0 %	0 %	0 %	0 %	0 %	0 %	0 %	0 %	0 %	0 %	0 %	0 %	0 %	100 %	0 %	0 %	0 %	0 %	0 %	0 %	0 %	0 %	0 %	0 %
READ	0 %	0 %	0 %	0 %	0 %	0 %	0 %	0 %	0 %	0 %	0 %	0 %	0 %	0 %	0 %	0 %	0 %	0 %	0 %	0 %	0 %	0 %	75 %	0 %	0 %	0 %	0 %	0 %	0 %	0 %	0 %	0 %
SARC	0 %	0 %	0 %	0 %	0 %	0 %	0 %	0 %	0 %	0 %	0 %	0 %	0 %	0 %	0 %	0 %	0 %	0 %	0 %	0 %	0 %	0 %	0 %	100 %	0 %	0 %	0 %	0 %	0 %	0 %	0 %	0 %
SKCM	0 %	0 %	0 %	0 %	0 %	0 %	0 %	0 %	0 %	0 %	0 %	0 %	0 %	0 %	0 %	0 %	0 %	0 %	0 %	0 %	0 %	0 %	0 %	0 %	100 %	0 %	0 %	0 %	0 %	0 %	0 %	0 %
STAD	0 %	0 %	0 %	0 %	0 %	0 %	0 %	0 %	0 %	0 %	0 %	0 %	0 %	0 %	0 %	0 %	0 %	0 %	0 %	0 %	0 %	0 %	0 %	0 %	0 %	100 %	0 %	0 %	0 %	0 %	0 %	0 %
TGCT	0 %	0 %	0 %	0 %	0 %	0 %	0 %	0 %	0 %	0 %	0 %	0 %	0 %	0 %	0 %	0 %	0 %	0 %	0 %	0 %	0 %	0 %	0 %	0 %	0 %	0 %	100 %	0 %	0 %	0 %	0 %	0 %
THCA	0 %	0 %	0 %	0 %	0 %	0 %	0 %	0 %	0 %	0 %	0 %	0 %	0 %	0 %	0 %	0 %	0 %	0 %	0 %	0 %	0 %	0 %	0 %	0 %	0 %	0 %	0 %	100 %	0 %	0 %	0 %	0 %
THYM	0 %	0 %	0 %	0 %	0 %	0 %	0 %	0 %	0 %	0 %	0 %	0 %	0 %	0 %	0 %	0 %	0 %	0 %	0 %	0 %	0 %	0 %	0 %	0 %	0 %	0 %	0 %	0 %	100 %	0 %	0 %	0 %
UCEC	0 %	0 %	0 %	0 %	0 %	0 %	0 %	0 %	0 %	0 %	0 %	0 %	0 %	0 %	0 %	0 %	0 %	0 %	0 %	0 %	0 %	0 %	0 %	0 %	0 %	0 %	0 %	0 %	0 %	100 %	0 %	0 %
UCS	0 %	0 %	0 %	0 %	0 %	0 %	0 %	0 %	0 %	0 %	0 %	0 %	0 %	0 %	0 %	0 %	0 %	0 %	0 %	0 %	0 %	0 %	0 %	0 %	0 %	0 %	0 %	0 %	0 %	0 %	100 %	0 %
UVM	0 %	0 %	0 %	0 %	0 %	0 %	0 %	0 %	0 %	0 %	0 %	0 %	0 %	0 %	0 %	0 %	0 %	0 %	0 %	0 %	0 %	0 %	0 %	0 %	0 %	0 %	0 %	0 %	0 %	0 %	0 %	100 %

Perceptron Neural Network (NN)

	ACC	BLCA	BRCA	CESC	CHOL	COAD	ESCA	GBM	HNSC	KICH	KIRC	KIRP	LAML	LGG	LIHC	LUAD	LUSC	MESO	OV	PAAD	PCPG	PRAD	READ	SARC	SKCM	STAD	TGCT	THCA	THYM	UCEC	UCS	UVM
**ACC**	74 %	0 %	0 %	0 %	failed	0 %	0 %	0 %	0 %	1 %	0 %	5 %	0 %	0 %	1 %	0 %	0 %	0 %	0 %	0 %	2 %	0 %	0 %	0 %	1 %	0 %	0 %	0 %	0 %	0 %	0 %	0 %
**BLCA**	0 %	89 %	0 %	0 %	failed	1 %	0 %	0 %	4 %	0 %	0 %	0 %	1 %	0 %	0 %	1 %	4 %	0 %	0 %	0 %	0 %	0 %	0 %	3 %	0 %	0 %	5 %	0 %	2 %	0 %	2 %	0 %
**BRCA**	0 %	0 %	99 %	1 %	failed	0 %	0 %	0 %	0 %	0 %	0 %	0 %	0 %	0 %	1 %	1 %	0 %	2 %	0 %	0 %	0 %	0 %	0 %	3 %	0 %	0 %	0 %	0 %	5 %	0 %	0 %	0 %
**CESC**	0 %	1 %	0 %	90 %	failed	2 %	4 %	0 %	2 %	0 %	0 %	0 %	0 %	0 %	0 %	1 %	1 %	0 %	0 %	1 %	0 %	0 %	0 %	0 %	0 %	0 %	0 %	0 %	1 %	3 %	4 %	0 %
**CHOL**	5 %	0 %	0 %	0 %	failed	0 %	0 %	0 %	0 %	0 %	0 %	0 %	0 %	0 %	6 %	0 %	0 %	0 %	0 %	1 %	0 %	0 %	0 %	0 %	0 %	0 %	0 %	0 %	0 %	0 %	0 %	0 %
**COAD**	2 %	0 %	0 %	0 %	failed	73 %	0 %	0 %	0 %	0 %	0 %	0 %	0 %	0 %	0 %	1 %	0 %	0 %	0 %	1 %	0 %	0 %	24 %	0 %	0 %	0 %	0 %	0 %	0 %	0 %	0 %	0 %
**ESCA**	0 %	1 %	0 %	0 %	failed	1 %	68 %	0 %	1 %	0 %	0 %	0 %	0 %	0 %	0 %	0 %	1 %	0 %	0 %	5 %	0 %	0 %	0 %	0 %	0 %	13 %	0 %	1 %	0 %	0 %	0 %	0 %
**GBM**	0 %	0 %	0 %	0 %	failed	0 %	0 %	84 %	0 %	0 %	0 %	0 %	0 %	7 %	0 %	0 %	0 %	0 %	0 %	0 %	0 %	0 %	0 %	1 %	0 %	0 %	0 %	0 %	1 %	0 %	2 %	0 %
**HNSC**	0 %	0 %	0 %	2 %	failed	0 %	15 %	0 %	89 %	0 %	0 %	0 %	0 %	0 %	1 %	0 %	4 %	0 %	0 %	0 %	0 %	0 %	0 %	0 %	0 %	0 %	1 %	0 %	1 %	0 %	0 %	0 %
**KICH**	0 %	0 %	0 %	0 %	failed	0 %	0 %	0 %	0 %	65 %	5 %	1 %	0 %	0 %	0 %	0 %	0 %	0 %	0 %	0 %	0 %	0 %	0 %	0 %	0 %	0 %	0 %	0 %	0 %	1 %	0 %	0 %
**KIRC**	0 %	0 %	0 %	0 %	failed	0 %	0 %	0 %	0 %	13 %	88 %	3 %	0 %	0 %	0 %	0 %	0 %	0 %	0 %	0 %	0 %	0 %	0 %	1 %	0 %	0 %	0 %	0 %	0 %	0 %	0 %	0 %
**KIRP**	0 %	1 %	0 %	0 %	failed	0 %	0 %	1 %	0 %	13 %	4 %	89 %	0 %	0 %	0 %	0 %	0 %	0 %	1 %	0 %	0 %	0 %	0 %	0 %	0 %	1 %	0 %	0 %	0 %	0 %	0 %	0 %
**LAML**	0 %	0 %	0 %	0 %	failed	0 %	0 %	0 %	0 %	0 %	0 %	0 %	96 %	0 %	0 %	0 %	0 %	0 %	0 %	0 %	0 %	0 %	0 %	3 %	0 %	0 %	0 %	0 %	0 %	0 %	0 %	0 %
**LGG**	0 %	0 %	0 %	0 %	failed	0 %	0 %	13 %	0 %	0 %	0 %	0 %	0 %	93 %	0 %	0 %	0 %	0 %	0 %	0 %	0 %	0 %	0 %	0 %	0 %	0 %	0 %	0 %	0 %	0 %	0 %	0 %
**LIHC**	12 %	0 %	0 %	0 %	failed	0 %	0 %	0 %	0 %	0 %	0 %	0 %	0 %	0 %	90 %	0 %	0 %	0 %	0 %	1 %	0 %	0 %	0 %	1 %	0 %	0 %	0 %	0 %	0 %	0 %	0 %	0 %
**LUAD**	0 %	2 %	0 %	0 %	failed	0 %	0 %	0 %	0 %	0 %	0 %	0 %	0 %	0 %	0 %	92 %	16 %	0 %	0 %	0 %	0 %	0 %	0 %	0 %	0 %	0 %	1 %	0 %	0 %	1 %	0 %	0 %
**LUSC**	0 %	1 %	0 %	1 %	failed	0 %	4 %	0 %	4 %	0 %	0 %	0 %	0 %	0 %	0 %	3 %	74 %	0 %	0 %	0 %	0 %	0 %	0 %	0 %	0 %	0 %	1 %	0 %	1 %	0 %	0 %	0 %
**MESO**	0 %	0 %	0 %	0 %	failed	0 %	0 %	3 %	0 %	0 %	1 %	0 %	0 %	0 %	0 %	0 %	0 %	96 %	1 %	0 %	1 %	0 %	0 %	1 %	0 %	0 %	0 %	0 %	0 %	0 %	0 %	0 %
**OV**	0 %	0 %	0 %	0 %	failed	0 %	0 %	0 %	0 %	0 %	0 %	0 %	0 %	0 %	0 %	0 %	0 %	2 %	96 %	0 %	0 %	0 %	0 %	0 %	0 %	0 %	0 %	0 %	0 %	8 %	4 %	0 %
**PAAD**	2 %	1 %	0 %	2 %	failed	0 %	2 %	0 %	0 %	0 %	0 %	0 %	0 %	0 %	1 %	0 %	0 %	0 %	0 %	87 %	0 %	0 %	0 %	0 %	0 %	2 %	0 %	0 %	0 %	0 %	0 %	0 %
**PCPG**	5 %	0 %	0 %	0 %	failed	0 %	0 %	0 %	0 %	0 %	0 %	0 %	0 %	0 %	0 %	0 %	0 %	0 %	0 %	0 %	96 %	0 %	0 %	0 %	0 %	0 %	0 %	0 %	0 %	0 %	0 %	0 %
**PRAD**	0 %	1 %	0 %	0 %	failed	0 %	0 %	0 %	0 %	0 %	0 %	0 %	0 %	0 %	0 %	0 %	0 %	0 %	0 %	0 %	0 %	100 %	0 %	0 %	0 %	1 %	0 %	0 %	0 %	0 %	0 %	0 %
**READ**	0 %	1 %	0 %	0 %	failed	21 %	0 %	0 %	0 %	0 %	0 %	0 %	0 %	0 %	0 %	0 %	0 %	0 %	0 %	0 %	0 %	0 %	76 %	0 %	0 %	0 %	0 %	0 %	0 %	0 %	0 %	0 %
**SARC**	0 %	0 %	0 %	0 %	failed	0 %	0 %	0 %	0 %	1 %	1 %	1 %	1 %	0 %	0 %	0 %	0 %	0 %	0 %	0 %	0 %	0 %	0 %	75 %	0 %	0 %	6 %	0 %	0 %	1 %	26 %	0 %
**SKCM**	0 %	0 %	0 %	1 %	failed	0 %	0 %	0 %	0 %	0 %	0 %	0 %	1 %	0 %	0 %	1 %	0 %	0 %	0 %	0 %	0 %	0 %	0 %	5 %	97 %	0 %	0 %	0 %	1 %	0 %	2 %	7 %
**STAD**	0 %	0 %	0 %	0 %	failed	1 %	7 %	0 %	0 %	0 %	0 %	0 %	0 %	0 %	0 %	0 %	0 %	0 %	0 %	4 %	1 %	0 %	0 %	0 %	0 %	82 %	0 %	0 %	0 %	0 %	0 %	0 %
**TGCT**	0 %	1 %	0 %	0 %	failed	0 %	0 %	0 %	0 %	4 %	0 %	0 %	2 %	0 %	0 %	0 %	0 %	0 %	0 %	0 %	0 %	0 %	0 %	1 %	0 %	0 %	85 %	1 %	0 %	1 %	6 %	0 %
**THCA**	0 %	0 %	0 %	0 %	failed	0 %	0 %	0 %	0 %	0 %	0 %	0 %	0 %	0 %	0 %	0 %	0 %	0 %	0 %	0 %	0 %	0 %	0 %	0 %	0 %	0 %	0 %	98 %	0 %	0 %	0 %	0 %
**THYM**	0 %	0 %	0 %	0 %	failed	0 %	0 %	0 %	0 %	0 %	0 %	0 %	0 %	0 %	0 %	0 %	0 %	0 %	0 %	0 %	0 %	0 %	0 %	0 %	0 %	0 %	0 %	0 %	89 %	0 %	0 %	0 %
**UCEC**	0 %	1 %	0 %	1 %	failed	1 %	1 %	0 %	0 %	1 %	0 %	0 %	0 %	0 %	0 %	0 %	0 %	0 %	1 %	0 %	0 %	0 %	0 %	0 %	0 %	0 %	1 %	0 %	0 %	74 %	24 %	0 %
**UCS**	0 %	0 %	0 %	0 %	failed	0 %	0 %	0 %	0 %	0 %	0 %	0 %	0 %	0 %	0 %	0 %	0 %	0 %	0 %	0 %	0 %	0 %	0 %	3 %	0 %	0 %	2 %	0 %	0 %	11 %	31 %	0 %
**UVM**	0 %	0 %	0 %	0 %	failed	0 %	0 %	0 %	0 %	0 %	0 %	0 %	0 %	0 %	0 %	0 %	0 %	0 %	0 %	0 %	0 %	0 %	0 %	0 %	0 %	0 %	0 %	0 %	0 %	0 %	0 %	93 %

C5.0 Classification and Regression Tress (C5.0)

	ACC	BLCA	BRCA	CESC	CHOL	COAD	ESCA	GBM	HNSC	KICH	KIRC	KIRP	LAML	LGG	LIHC	LUAD	LUSC	MESO	OV	PAAD	PCPG	PRAD	READ	SARC	SKCM	STAD	TGCT	THCA	THYM	UCEC	UCS	UVM
**ACC**	100 %	0 %	0 %	0 %	0 %	0 %	0 %	0 %	0 %	0 %	0 %	0 %	0 %	0 %	0 %	0 %	0 %	0 %	0 %	0 %	0 %	0 %	0 %	0 %	0 %	0 %	0 %	0 %	0 %	0 %	0 %	0 %
**BLCA**	0 %	100 %	0 %	0 %	0 %	0 %	0 %	0 %	0 %	0 %	0 %	0 %	0 %	0 %	0 %	0 %	0 %	0 %	0 %	0 %	0 %	0 %	0 %	0 %	0 %	0 %	0 %	0 %	0 %	0 %	0 %	0 %
**BRCA**	0 %	0 %	100 %	0 %	0 %	0 %	0 %	0 %	0 %	0 %	0 %	0 %	0 %	0 %	0 %	0 %	0 %	0 %	0 %	0 %	0 %	0 %	0 %	0 %	0 %	0 %	0 %	0 %	0 %	0 %	0 %	0 %
**CESC**	0 %	0 %	0 %	100 %	0 %	0 %	0 %	0 %	0 %	0 %	0 %	0 %	0 %	0 %	0 %	0 %	0 %	0 %	0 %	0 %	0 %	0 %	0 %	0 %	0 %	0 %	0 %	0 %	0 %	0 %	0 %	0 %
**CHOL**	0 %	0 %	0 %	0 %	100 %	0 %	0 %	0 %	0 %	0 %	0 %	0 %	0 %	0 %	0 %	0 %	0 %	0 %	0 %	0 %	0 %	0 %	0 %	0 %	0 %	0 %	0 %	0 %	0 %	0 %	0 %	0 %
**COAD**	0 %	0 %	0 %	0 %	0 %	100 %	0 %	0 %	0 %	0 %	0 %	0 %	0 %	0 %	0 %	0 %	0 %	0 %	0 %	0 %	0 %	0 %	25 %	0 %	0 %	0 %	0 %	0 %	0 %	0 %	0 %	0 %
**ESCA**	0 %	0 %	0 %	0 %	0 %	0 %	100 %	0 %	0 %	0 %	0 %	0 %	0 %	0 %	0 %	0 %	0 %	0 %	0 %	0 %	0 %	0 %	0 %	0 %	0 %	0 %	0 %	0 %	0 %	0 %	0 %	0 %
**GBM**	0 %	0 %	0 %	0 %	0 %	0 %	0 %	100 %	0 %	0 %	0 %	0 %	0 %	0 %	0 %	0 %	0 %	0 %	0 %	0 %	0 %	0 %	0 %	0 %	0 %	0 %	0 %	0 %	0 %	0 %	0 %	0 %
**HNSC**	0 %	0 %	0 %	0 %	0 %	0 %	0 %	0 %	100 %	0 %	0 %	0 %	0 %	0 %	0 %	0 %	0 %	0 %	0 %	0 %	0 %	0 %	0 %	0 %	0 %	0 %	0 %	0 %	0 %	0 %	0 %	0 %
**KICH**	0 %	0 %	0 %	0 %	0 %	0 %	0 %	0 %	0 %	100 %	0 %	0 %	0 %	0 %	0 %	0 %	0 %	0 %	0 %	0 %	0 %	0 %	0 %	0 %	0 %	0 %	0 %	0 %	0 %	0 %	0 %	0 %
**KIRC**	0 %	0 %	0 %	0 %	0 %	0 %	0 %	0 %	0 %	0 %	100 %	0 %	0 %	0 %	0 %	0 %	0 %	0 %	0 %	0 %	0 %	0 %	0 %	0 %	0 %	0 %	0 %	0 %	0 %	0 %	0 %	0 %
**KIRP**	0 %	0 %	0 %	0 %	0 %	0 %	0 %	0 %	0 %	0 %	0 %	100 %	0 %	0 %	0 %	0 %	0 %	0 %	0 %	0 %	0 %	0 %	0 %	0 %	0 %	0 %	0 %	0 %	0 %	0 %	0 %	0 %
**LAML**	0 %	0 %	0 %	0 %	0 %	0 %	0 %	0 %	0 %	0 %	0 %	0 %	100 %	0 %	0 %	0 %	0 %	0 %	0 %	0 %	0 %	0 %	0 %	0 %	0 %	0 %	0 %	0 %	0 %	0 %	0 %	0 %
**LGG**	0 %	0 %	0 %	0 %	0 %	0 %	0 %	0 %	0 %	0 %	0 %	0 %	0 %	100 %	0 %	0 %	0 %	0 %	0 %	0 %	0 %	0 %	0 %	0 %	0 %	0 %	0 %	0 %	0 %	0 %	0 %	0 %
**LIHC**	0 %	0 %	0 %	0 %	0 %	0 %	0 %	0 %	0 %	0 %	0 %	0 %	0 %	0 %	100 %	0 %	0 %	0 %	0 %	0 %	0 %	0 %	0 %	0 %	0 %	0 %	0 %	0 %	0 %	0 %	0 %	0 %
**LUAD**	0 %	0 %	0 %	0 %	0 %	0 %	0 %	0 %	0 %	0 %	0 %	0 %	0 %	0 %	0 %	100 %	0 %	0 %	0 %	0 %	0 %	0 %	0 %	0 %	0 %	0 %	0 %	0 %	0 %	0 %	0 %	0 %
**LUSC**	0 %	0 %	0 %	0 %	0 %	0 %	0 %	0 %	0 %	0 %	0 %	0 %	0 %	0 %	0 %	0 %	100 %	0 %	0 %	0 %	0 %	0 %	0 %	0 %	0 %	0 %	0 %	0 %	0 %	0 %	0 %	0 %
**MESO**	0 %	0 %	0 %	0 %	0 %	0 %	0 %	0 %	0 %	0 %	0 %	0 %	0 %	0 %	0 %	0 %	0 %	100 %	0 %	0 %	0 %	0 %	0 %	0 %	0 %	0 %	0 %	0 %	0 %	0 %	0 %	0 %
**OV**	0 %	0 %	0 %	0 %	0 %	0 %	0 %	0 %	0 %	0 %	0 %	0 %	0 %	0 %	0 %	0 %	0 %	0 %	100 %	0 %	0 %	0 %	0 %	0 %	0 %	0 %	0 %	0 %	0 %	0 %	0 %	0 %
**PAAD**	0 %	0 %	0 %	0 %	0 %	0 %	0 %	0 %	0 %	0 %	0 %	0 %	0 %	0 %	0 %	0 %	0 %	0 %	0 %	100 %	0 %	0 %	0 %	0 %	0 %	0 %	0 %	0 %	0 %	0 %	0 %	0 %
**PCPG**	0 %	0 %	0 %	0 %	0 %	0 %	0 %	0 %	0 %	0 %	0 %	0 %	0 %	0 %	0 %	0 %	0 %	0 %	0 %	0 %	100 %	0 %	0 %	0 %	0 %	0 %	0 %	0 %	0 %	0 %	0 %	0 %
**PRAD**	0 %	0 %	0 %	0 %	0 %	0 %	0 %	0 %	0 %	0 %	0 %	0 %	0 %	0 %	0 %	0 %	0 %	0 %	0 %	0 %	0 %	100 %	0 %	0 %	0 %	0 %	0 %	0 %	0 %	0 %	0 %	0 %
**READ**	0 %	0 %	0 %	0 %	0 %	0 %	0 %	0 %	0 %	0 %	0 %	0 %	0 %	0 %	0 %	0 %	0 %	0 %	0 %	0 %	0 %	0 %	75 %	0 %	0 %	0 %	0 %	0 %	0 %	0 %	0 %	0 %
**SARC**	0 %	0 %	0 %	0 %	0 %	0 %	0 %	0 %	0 %	0 %	0 %	0 %	0 %	0 %	0 %	0 %	0 %	0 %	0 %	0 %	0 %	0 %	0 %	100 %	0 %	0 %	0 %	0 %	0 %	0 %	0 %	0 %
**SKCM**	0 %	0 %	0 %	0 %	0 %	0 %	0 %	0 %	0 %	0 %	0 %	0 %	0 %	0 %	0 %	0 %	0 %	0 %	0 %	0 %	0 %	0 %	0 %	0 %	100 %	0 %	0 %	0 %	0 %	0 %	0 %	0 %
**STAD**	0 %	0 %	0 %	0 %	0 %	0 %	0 %	0 %	0 %	0 %	0 %	0 %	0 %	0 %	0 %	0 %	0 %	0 %	0 %	0 %	0 %	0 %	0 %	0 %	0 %	100 %	0 %	0 %	0 %	0 %	0 %	0 %
**TGCT**	0 %	0 %	0 %	0 %	0 %	0 %	0 %	0 %	0 %	0 %	0 %	0 %	0 %	0 %	0 %	0 %	0 %	0 %	0 %	0 %	0 %	0 %	0 %	0 %	0 %	0 %	100 %	0 %	0 %	0 %	0 %	0 %
**THCA**	0 %	0 %	0 %	0 %	0 %	0 %	0 %	0 %	0 %	0 %	0 %	0 %	0 %	0 %	0 %	0 %	0 %	0 %	0 %	0 %	0 %	0 %	0 %	0 %	0 %	0 %	0 %	100 %	0 %	0 %	0 %	0 %
**THYM**	0 %	0 %	0 %	0 %	0 %	0 %	0 %	0 %	0 %	0 %	0 %	0 %	0 %	0 %	0 %	0 %	0 %	0 %	0 %	0 %	0 %	0 %	0 %	0 %	0 %	0 %	0 %	0 %	100 %	0 %	0 %	0 %
**UCEC**	0 %	0 %	0 %	0 %	0 %	0 %	0 %	0 %	0 %	0 %	0 %	0 %	0 %	0 %	0 %	0 %	0 %	0 %	0 %	0 %	0 %	0 %	0 %	0 %	0 %	0 %	0 %	0 %	0 %	100 %	0 %	0 %
**UCS**	0 %	0 %	0 %	0 %	0 %	0 %	0 %	0 %	0 %	0 %	0 %	0 %	0 %	0 %	0 %	0 %	0 %	0 %	0 %	0 %	0 %	0 %	0 %	0 %	0 %	0 %	0 %	0 %	0 %	0 %	100 %	0 %
**UVM**	0 %	0 %	0 %	0 %	0 %	0 %	0 %	0 %	0 %	0 %	0 %	0 %	0 %	0 %	0 %	0 %	0 %	0 %	0 %	0 %	0 %	0 %	0 %	0 %	0 %	0 %	0 %	0 %	0 %	0 %	0 %	100 %

% of correctly classified cases (azure) per tumor type. Significant outliers (> 5% misclassified cases) are highlighted in orange.

Supplemental Table 3. Average recall accuracy

	ACC	BLCA	BRCA	CESC	CHOL	COAD	ESCA	GBM	HNSC	KICH	KIRC	KIRP	LAML	LGG	LIHC	LUAD	LUSC	MESO	OV	PAAD	PCPG	PRAD	READ	SARC	SKCM	STAD	TGCT	THCA	THYM	UCEC	UCS	UVM
**ACC**	91 %	0 %	0 %	0 %	0 %	0 %	0 %	0 %	0 %	0 %	0 %	2 %	0 %	0 %	0 %	0 %	0 %	0 %	0 %	0 %	1 %	0 %	0 %	0 %	0 %	0 %	0 %	0 %	0 %	0 %	0 %	0 %
**BLCA**	0 %	96 %	0 %	0 %	0 %	0 %	0 %	0 %	1 %	0 %	0 %	0 %	0 %	0 %	0 %	0 %	1 %	0 %	0 %	0 %	0 %	0 %	0 %	1 %	0 %	0 %	2 %	0 %	1 %	0 %	1 %	0 %
**BRCA**	0 %	0 %	100 %	0 %	0 %	0 %	0 %	0 %	0 %	0 %	0 %	0 %	0 %	0 %	0 %	0 %	0 %	1 %	0 %	0 %	0 %	0 %	0 %	1 %	0 %	0 %	0 %	0 %	2 %	0 %	0 %	0 %
**CESC**	0 %	0 %	0 %	97 %	0 %	1 %	1 %	0 %	1 %	0 %	0 %	0 %	0 %	0 %	0 %	0 %	0 %	0 %	0 %	0 %	0 %	0 %	0 %	0 %	0 %	0 %	0 %	0 %	0 %	1 %	1 %	0 %
**CHOL**	2 %	0 %	0 %	0 %	100 %	0 %	0 %	0 %	0 %	0 %	0 %	0 %	0 %	0 %	2 %	0 %	0 %	0 %	0 %	0 %	0 %	0 %	0 %	0 %	0 %	0 %	0 %	0 %	0 %	0 %	0 %	0 %
**COAD**	1 %	0 %	0 %	0 %	0 %	91 %	0 %	0 %	0 %	0 %	0 %	0 %	0 %	0 %	0 %	0 %	0 %	0 %	0 %	0 %	0 %	0 %	25 %	0 %	0 %	0 %	0 %	0 %	0 %	0 %	0 %	0 %
**ESCA**	0 %	0 %	0 %	0 %	0 %	0 %	89 %	0 %	0 %	0 %	0 %	0 %	0 %	0 %	0 %	0 %	0 %	0 %	0 %	2 %	0 %	0 %	0 %	0 %	0 %	4 %	0 %	0 %	0 %	0 %	0 %	0 %
**GBM**	0 %	0 %	0 %	0 %	0 %	0 %	0 %	95 %	0 %	0 %	0 %	0 %	0 %	2 %	0 %	0 %	0 %	0 %	0 %	0 %	0 %	0 %	0 %	0 %	0 %	0 %	0 %	0 %	0 %	0 %	1 %	0 %
**HNSC**	0 %	0 %	0 %	1 %	0 %	0 %	5 %	0 %	96 %	0 %	0 %	0 %	0 %	0 %	0 %	0 %	1 %	0 %	0 %	0 %	0 %	0 %	0 %	0 %	0 %	0 %	0 %	0 %	0 %	0 %	0 %	0 %
**KICH**	0 %	0 %	0 %	0 %	0 %	0 %	0 %	0 %	0 %	88 %	2 %	0 %	0 %	0 %	0 %	0 %	0 %	0 %	0 %	0 %	0 %	0 %	0 %	0 %	0 %	0 %	0 %	0 %	0 %	0 %	0 %	0 %
**KIRC**	0 %	0 %	0 %	0 %	0 %	0 %	0 %	0 %	0 %	4 %	96 %	1 %	0 %	0 %	0 %	0 %	0 %	0 %	0 %	0 %	0 %	0 %	0 %	0 %	0 %	0 %	0 %	0 %	0 %	0 %	0 %	0 %
**KIRP**	0 %	0 %	0 %	0 %	0 %	0 %	0 %	0 %	0 %	4 %	1 %	96 %	0 %	0 %	0 %	0 %	0 %	0 %	0 %	0 %	0 %	0 %	0 %	0 %	0 %	0 %	0 %	0 %	0 %	0 %	0 %	0 %
**LAML**	0 %	0 %	0 %	0 %	0 %	0 %	0 %	0 %	0 %	0 %	0 %	0 %	99 %	0 %	0 %	0 %	0 %	0 %	0 %	0 %	0 %	0 %	0 %	1 %	0 %	0 %	0 %	0 %	0 %	0 %	0 %	0 %
**LGG**	0 %	0 %	0 %	0 %	0 %	0 %	0 %	4 %	0 %	0 %	0 %	0 %	0 %	98 %	0 %	0 %	0 %	0 %	0 %	0 %	0 %	0 %	0 %	0 %	0 %	0 %	0 %	0 %	0 %	0 %	0 %	0 %
**LIHC**	4 %	0 %	0 %	0 %	0 %	0 %	0 %	0 %	0 %	0 %	0 %	0 %	0 %	0 %	97 %	0 %	0 %	0 %	0 %	0 %	0 %	0 %	0 %	0 %	0 %	0 %	0 %	0 %	0 %	0 %	0 %	0 %
**LUAD**	0 %	1 %	0 %	0 %	0 %	0 %	0 %	0 %	0 %	0 %	0 %	0 %	0 %	0 %	0 %	97 %	5 %	0 %	0 %	0 %	0 %	0 %	0 %	0 %	0 %	0 %	0 %	0 %	0 %	0 %	0 %	0 %
**LUSC**	0 %	0 %	0 %	0 %	0 %	0 %	1 %	0 %	1 %	0 %	0 %	0 %	0 %	0 %	0 %	1 %	91 %	0 %	0 %	0 %	0 %	0 %	0 %	0 %	0 %	0 %	0 %	0 %	0 %	0 %	0 %	0 %
**MESO**	0 %	0 %	0 %	0 %	0 %	0 %	0 %	1 %	0 %	0 %	0 %	0 %	0 %	0 %	0 %	0 %	0 %	99 %	0 %	0 %	0 %	0 %	0 %	0 %	0 %	0 %	0 %	0 %	0 %	0 %	0 %	0 %
**OV**	0 %	0 %	0 %	0 %	0 %	0 %	0 %	0 %	0 %	0 %	0 %	0 %	0 %	0 %	0 %	0 %	0 %	1 %	99 %	0 %	0 %	0 %	0 %	0 %	0 %	0 %	0 %	0 %	0 %	3 %	1 %	0 %
**PAAD**	1 %	0 %	0 %	1 %	0 %	0 %	1 %	0 %	0 %	0 %	0 %	0 %	0 %	0 %	0 %	0 %	0 %	0 %	0 %	96 %	0 %	0 %	0 %	0 %	0 %	1 %	0 %	0 %	0 %	0 %	0 %	0 %
**PCPG**	2 %	0 %	0 %	0 %	0 %	0 %	0 %	0 %	0 %	0 %	0 %	0 %	0 %	0 %	0 %	0 %	0 %	0 %	0 %	0 %	99 %	0 %	0 %	0 %	0 %	0 %	0 %	0 %	0 %	0 %	0 %	0 %
**PRAD**	0 %	0 %	0 %	0 %	0 %	0 %	0 %	0 %	0 %	0 %	0 %	0 %	0 %	0 %	0 %	0 %	0 %	0 %	0 %	0 %	0 %	100 %	0 %	0 %	0 %	0 %	0 %	0 %	0 %	0 %	0 %	0 %
**READ**	0 %	0 %	0 %	0 %	0 %	7 %	0 %	0 %	0 %	0 %	0 %	0 %	0 %	0 %	0 %	0 %	0 %	0 %	0 %	0 %	0 %	0 %	75 %	0 %	0 %	0 %	0 %	0 %	0 %	0 %	0 %	0 %
**SARC**	0 %	0 %	0 %	0 %	0 %	0 %	0 %	0 %	0 %	0 %	0 %	0 %	0 %	0 %	0 %	0 %	0 %	0 %	0 %	0 %	0 %	0 %	0 %	92 %	0 %	0 %	2 %	0 %	0 %	0 %	9 %	0 %
**SKCM**	0 %	0 %	0 %	0 %	0 %	0 %	0 %	0 %	0 %	0 %	0 %	0 %	0 %	0 %	0 %	0 %	0 %	0 %	0 %	0 %	0 %	0 %	0 %	2 %	99 %	0 %	0 %	0 %	0 %	0 %	1 %	2 %
**STAD**	0 %	0 %	0 %	0 %	0 %	0 %	2 %	0 %	0 %	0 %	0 %	0 %	0 %	0 %	0 %	0 %	0 %	0 %	0 %	1 %	0 %	0 %	0 %	0 %	0 %	94 %	0 %	0 %	0 %	0 %	0 %	0 %
**TGCT**	0 %	0 %	0 %	0 %	0 %	0 %	0 %	0 %	0 %	1 %	0 %	0 %	1 %	0 %	0 %	0 %	0 %	0 %	0 %	0 %	0 %	0 %	0 %	0 %	0 %	0 %	95 %	0 %	0 %	0 %	2 %	0 %
**THCA**	0 %	0 %	0 %	0 %	0 %	0 %	0 %	0 %	0 %	0 %	0 %	0 %	0 %	0 %	0 %	0 %	0 %	0 %	0 %	0 %	0 %	0 %	0 %	0 %	0 %	0 %	0 %	99 %	0 %	0 %	0 %	0 %
**THYM**	0 %	0 %	0 %	0 %	0 %	0 %	0 %	0 %	0 %	0 %	0 %	0 %	0 %	0 %	0 %	0 %	0 %	0 %	0 %	0 %	0 %	0 %	0 %	0 %	0 %	0 %	0 %	0 %	96 %	0 %	0 %	0 %
**UCEC**	0 %	0 %	0 %	0 %	0 %	0 %	0 %	0 %	0 %	0 %	0 %	0 %	0 %	0 %	0 %	0 %	0 %	0 %	0 %	0 %	0 %	0 %	0 %	0 %	0 %	0 %	0 %	0 %	0 %	91 %	8 %	0 %
**UCS**	0 %	0 %	0 %	0 %	0 %	0 %	0 %	0 %	0 %	0 %	0 %	0 %	0 %	0 %	0 %	0 %	0 %	0 %	0 %	0 %	0 %	0 %	0 %	1 %	0 %	0 %	1 %	0 %	0 %	4 %	77 %	0 %
**UVM**	0 %	0 %	0 %	0 %	0 %	0 %	0 %	0 %	0 %	0 %	0 %	0 %	0 %	0 %	0 %	0 %	0 %	0 %	0 %	0 %	0 %	0 %	0 %	0 %	0 %	0 %	0 %	0 %	0 %	0 %	0 %	98 %
**Recall accuracy**	91 %	96 %	100 %	97 %	100 %	91 %	89 %	95 %	96 %	88 %	96 %	96 %	99 %	98 %	97 %	97 %	91 %	99 %	99 %	96 %	99 %	100 %	75 %	92 %	99 %	94 %	95 %	99 %	96 %	91 %	77 %	98 %

% average of correctly classified cases (azure) by the 3 algorithms (SVM, NN and C5.0) per tumor type. Significant outliers (> 5% misclassified cases) are highlighted in orange. Per-tumor recall accuracy is given in red.

Supplemental Table 4. Matrisome signatures of different cancer types

Adrenocortical Cancer (ACC)

Signature genes	Expression in tumor	Expression in rest of Pan-Cancer	P-value	Expression in tumor	Expression in normal tissue	P-value	Matrisome category	Fold change vs. Pan-Cancer	Fold change vs. normal
**ADAM22**	8.815177922	7.815739107	2.83E-05	8.815177922	7.86075	1.85E-42	ECM_REGULATORS	1.127875151	1.121416903
**ADAM32**	5.555628571	4.623915648	2.94E-06	5.555628571	3.996519318	8.93E-07	ECM_REGULATORS	1.201498685	1.390116781
**ADAMTS3**	7.664607792	5.852472863	4.13E-17	7.664607792	2.823254545	4.85E-17	ECM_REGULATORS	1.309635768	2.714812876
**ADAMTS7**	9.98702987	8.051686859	2.44E-28	9.98702987	1.104234091	4.17E-98	ECM_REGULATORS	1.240364913	9.044304964
**ADAMTSL5**	6.998357143	6.224306234	0.000785007	6.998357143	0.935293182	2.40E-10	ECM_REGULATORS	1.124359387	7.482527702
**AMH**	6.71878961	4.836009039	1.02E-11	6.71878961	0.357515909	1.29E-66	SECRETED_FACTORS	1.38932528	18.79298079
**ANGPT2**	9.451393506	8.571205476	1.15E-06	9.451393506	0.017190909	2.17E-40	SECRETED_FACTORS	1.102691276	549.7902093
**ANGPTL1**	6.825520779	6.022709597	0.005449909	6.825520779	0.705911364	7.89E-61	SECRETED_FACTORS	1.133297342	9.669090386
**ANGPTL2**	11.04859091	10.4266921	0.002108585	11.04859091	10.47743068	4.83E-76	SECRETED_FACTORS	1.059644881	1.054513386
**ANGPTL5**	1.966454545	1.364826042	0.000481296	1.966454545	0.079918182	2.37E-51	SECRETED_FACTORS	1.44080966	24.60584689
**ANXA5**	14.28912857	13.37547354	9.97E-15	14.28912857	0.076728409	1.24E-75	ECM_AFFILIATED	1.068308239	186.2299602
**ANXA6**	13.20392987	12.24751976	1.23E-11	13.20392987	0.067611364	5.21E-56	ECM_AFFILIATED	1.078090106	195.2915776
**BDNF**	6.014312987	5.221790106	0.000965588	6.014312987	2.182115909	1.39E-31	SECRETED_FACTORS	1.151772259	2.756184015
**C1QTNF1**	10.79542597	9.826968473	6.44E-07	10.79542597	1.214376136	9.47E-65	ECM_AFFILIATED	1.098550993	8.889688829
**CCBE1**	8.591724675	4.721905331	1.39E-40	8.591724675	1.862502273	2.04E-50	SECRETED_FACTORS	1.819546152	4.613000908
**COL15A1**	11.08727922	10.47593997	0.015402876	11.08727922	7.035285227	2.07E-74	COLLAGENS	1.058356506	1.575953051
**COL15A1**	11.08727922	10.47593997	0.015402876	11.08727922	7.035285227	2.07E-74	BASEMENT_MEMBRANES	1.058356506	1.575953051
**COL27A1**	10.29321558	9.597191095	0.000647837	10.29321558	0.979778409	9.98E-17	COLLAGENS	1.072523771	10.50565668
**COL4A3**	6.379018182	5.703849318	0.04700141	6.379018182	4.126751136	3.46E-88	COLLAGENS	1.11837074	1.545772442
**COL4A3**	6.379018182	5.703849318	0.04700141	6.379018182	4.126751136	3.46E-88	BASEMENT_MEMBRANES	1.11837074	1.545772442
**CTSA**	15.10262597	12.79478354	2.68E-105	15.10262597	0.294693182	5.82E-80	ECM_REGULATORS	1.180373699	51.24864403
**CTSD**	15.58845844	15.15320206	0.00035932	15.58845844	8.252893182	2.89E-60	ECM_REGULATORS	1.028723723	1.888847717
**DDX26B**	9.465680519	8.163761681	9.16E-19	9.465680519	1.648797727	1.39E-92	ECM_GLYCOPROTEINS	1.159475361	5.740959223
**DHH**	4.00954026	3.148019068	6.15E-07	4.00954026	1.124777273	1.29E-40	SECRETED_FACTORS	1.273670894	3.564741533
**EGLN1**	11.63714156	11.20446073	5.08E-07	11.63714156	7.600998864	1.81E-97	ECM_REGULATORS	1.038616837	1.531001618
**EGLN2**	11.93181169	11.42586543	2.80E-11	11.93181169	9.702230682	6.66E-98	ECM_REGULATORS	1.044280782	1.229800865
**ELSPBP1**	2.825581818	0.449653093	2.32E-64	2.825581818	0.149809091	4.97E-54	ECM_GLYCOPROTEINS	6.283915003	18.86121731
**EMID1**	10.61436494	8.135856465	3.03E-31	10.61436494	8.202040909	9.78E-76	ECM_GLYCOPROTEINS	1.304640142	1.294112655
**EPO**	2.856264935	2.119340672	0.001616851	2.856264935	2.3963625	0.000121936	SECRETED_FACTORS	1.347713925	1.191916889
**ESM1**	9.877927273	7.684936378	2.81E-14	9.877927273	6.248171591	8.98E-40	PROTEOGLYCANS	1.285362271	1.580930858
**F10**	7.413505195	6.188117925	3.12E-05	7.413505195	0.041297727	1.59E-66	ECM_REGULATORS	1.198022611	179.5136363
**FCN2**	6.401418182	1.582387752	9.30E-90	6.401418182	0.146671591	0.020297684	ECM_AFFILIATED	4.045416917	43.64456772
**FGF11**	8.524801299	7.671139356	0.000690552	8.524801299	0.404869318	8.62E-58	SECRETED_FACTORS	1.111282288	21.05568616
**FGF12**	9.102087013	6.275401441	1.45E-25	9.102087013	2.663564773	0.000636392	SECRETED_FACTORS	1.450439004	3.417257619
**FNDC7**	1.345105195	0.778636964	2.58E-05	1.345105195	0	2.28E-67	ECM_GLYCOPROTEINS	1.727512636	Inf
**GPC5**	3.583584416	2.904613583	0.018573648	3.583584416	2.522506818	1.03E-88	ECM_AFFILIATED	1.233755993	1.420644095
**HYAL1**	9.114428571	7.567390845	1.02E-09	9.114428571	3.861846591	2.01E-19	ECM_REGULATORS	1.204434759	2.360121863
**HYAL2**	11.55970649	11.15881723	9.93E-06	11.55970649	1.068781818	1.01E-152	ECM_REGULATORS	1.035925784	10.81577764

**LAMB1**	13.00148182	12.22737797	4.22E-05	13.00148182	6.506263636	2.59E-63	BASEMENT_MEMBRANES	1.063309063	1.998302335
**LAMB1**	13.00148182	12.22737797	4.22E-05	13.00148182	6.506263636	2.59E-63	ECM_GLYCOPROTEINS	1.063309063	1.998302335
**LAMB2**	13.91894805	12.70728354	1.87E-17	13.91894805	6.199969318	1.95E-110	BASEMENT_MEMBRANES	1.095351969	2.245002731
**LAMB2**	13.91894805	12.70728354	1.87E-17	13.91894805	6.199969318	1.95E-110	ECM_GLYCOPROTEINS	1.095351969	2.245002731
**LGALS1**	13.43300519	12.68939432	4.32E-06	13.43300519	9.332195455	7.31E-148	ECM_AFFILIATED	1.058600974	1.439426045
**MATN3**	7.001937662	6.484673487	0.041961317	7.001937662	6.243238636	7.19E-83	ECM_GLYCOPROTEINS	1.079767189	1.12152331
**MGP**	12.48060909	11.8244419	0.029056308	12.48060909	9.700438636	7.47E-33	ECM_GLYCOPROTEINS	1.055492445	1.286602551
**MMRN2**	10.6691013	9.716885379	8.64E-09	10.6691013	4.095342045	7.55E-108	ECM_GLYCOPROTEINS	1.097996002	2.605179538
**NOV**	11.62837143	8.007560989	1.21E-58	11.62837143	8.679488636	1.55E-77	ECM_GLYCOPROTEINS	1.452173945	1.339753056
**NTN4**	10.30404805	9.744069894	0.019749201	10.30404805	0.432769318	5.65E-80	BASEMENT_MEMBRANES	1.057468611	23.80956232
**NTN4**	10.30404805	9.744069894	0.019749201	10.30404805	0.432769318	5.65E-80	ECM_GLYCOPROTEINS	1.057468611	23.80956232
**NTN5**	4.683575325	3.692497166	2.85E-08	4.683575325	2.335186364	2.34E-71	ECM_GLYCOPROTEINS	1.268403228	2.005653766
**NTN5**	4.683575325	3.692497166	2.85E-08	4.683575325	2.335186364	2.34E-71	BASEMENT_MEMBRANES	1.268403228	2.005653766
**NTNG2**	6.431781818	5.944061326	0.037233929	6.431781818	5.459398864	4.86E-78	ECM_GLYCOPROTEINS	1.082051726	1.178111726
**NTNG2**	6.431781818	5.944061326	0.037233929	6.431781818	5.459398864	4.86E-78	BASEMENT_MEMBRANES	1.082051726	1.178111726
**OGFOD1**	11.28945584	10.46202936	1.87E-48	11.28945584	2.750610227	6.86E-26	ECM_REGULATORS	1.079088527	4.104345913
**P4HTM**	10.94929091	10.30303633	1.32E-05	10.94929091	9.048806818	3.01E-113	ECM_REGULATORS	1.062724672	1.210025933
**PCOLCE**	11.30038312	10.58330325	0.000448722	11.30038312	0.029690909	0.017629848	ECM_GLYCOPROTEINS	1.067755771	380.6007786
**PCOLCE2**	8.264458442	6.546396638	1.32E-08	8.264458442	2.041115909	5.09E-44	ECM_GLYCOPROTEINS	1.262443891	4.048990263
**PLXNB3**	10.1234026	8.331585091	1.78E-09	10.1234026	1.239193182	5.01E-65	ECM_AFFILIATED	1.215063219	8.16934982
**PLXNC1**	10.76013377	9.654817887	2.67E-09	10.76013377	1.197148864	1.48E-85	ECM_AFFILIATED	1.114483348	8.988133467
**SDC3**	13.81238182	11.8710735	1.27E-28	13.81238182	10.3619375	1.30E-62	ECM_AFFILIATED	1.163532668	1.332992196
**SEMA3B**	10.56747143	9.546515927	8.92E-06	10.56747143	4.055819318	8.18E-32	ECM_AFFILIATED	1.106945352	2.605508431
**SEMA7A**	9.735885714	8.474149491	3.32E-11	9.735885714	7.163461364	1.76E-38	ECM_AFFILIATED	1.148892373	1.359103542
**SERPINA5**	15.0531026	6.399061671	1.95E-86	15.0531026	0.026001136	0.004495013	ECM_REGULATORS	2.352392174	578.9401812
**SERPINB6**	12.36965844	11.87641585	2.33E-06	12.36965844	1.951163636	7.96E-24	ECM_REGULATORS	1.041531266	6.339631495
**SERPINH1**	13.00680519	12.69555323	0.049037869	13.00680519	5.440107955	1.78E-47	ECM_REGULATORS	1.024516613	2.390909391
**SNED1**	10.49388182	9.159522046	9.15E-14	10.49388182	2.744521591	3.83E-106	ECM_GLYCOPROTEINS	1.145680066	3.823574153
**SPARC**	16.66842727	15.14837562	2.11E-16	16.66842727	6.178789773	8.38E-114	ECM_GLYCOPROTEINS	1.100344201	2.697684803
**SPOCK1**	11.90525714	9.333476119	5.73E-16	11.90525714	0.083861364	4.16E-70	PROTEOGLYCANS	1.275543751	141.963553
**SRPX**	9.338536364	8.165440701	7.06E-06	9.338536364	5.828085227	1.15E-63	ECM_GLYCOPROTEINS	1.143665934	1.602333528
**SSPO**	6.929906494	5.541056676	3.28E-09	6.929906494	2.583748864	1.31E-132	ECM_GLYCOPROTEINS	1.250647105	2.68211303
**TECTA**	6.116345455	5.164499789	9.04E-09	6.116345455	6.007329545	4.39E-24	ECM_GLYCOPROTEINS	1.18430549	1.01814715
**TIMP1**	14.64268961	13.05371847	1.56E-15	14.64268961	10.69748068	1.33E-79	ECM_REGULATORS	1.121725556	1.368797948
**TNXB**	12.63701039	9.455618722	2.78E-44	12.63701039	9.466342045	1.77E-30	ECM_GLYCOPROTEINS	1.336455155	1.33494124
**WFIKKN2**	3.120831169	2.384223074	0.00158006	3.120831169	1.607265909	1.78E-101	SECRETED_FACTORS	1.308950997	1.941701837
**WISP2**	10.49606104	6.064880317	1.45E-41	10.49606104	9.519772727	2.32E-93	ECM_GLYCOPROTEINS	1.730629541	1.102553742
**WNT11**	8.400097403	5.991537272	2.56E-18	8.400097403	2.8379625	3.97E-32	SECRETED_FACTORS	1.401993682	2.959904298
**WNT4**	8.500555844	6.949631508	2.22E-09	8.500555844	5.422438636	4.53E-50	SECRETED_FACTORS	1.223166413	1.567662894
**WNT6**	5.023546753	3.833595139	1.22E-06	5.023546753	3.036595455	2.15E-113	SECRETED_FACTORS	1.310400961	1.654335202
**VTN**	10.94211818	5.254219308	1.13E-44	10.94211818	5.340589773	4.52E-49	ECM_GLYCOPROTEINS	2.082539296	2.048859517
**VWA5B2**	11.05322208	4.448986647	6.11E-120	11.05322208	3.707904545	2.14E-50	ECM_GLYCOPROTEINS	2.484435885	2.980988842
**ZP1**	3.594903896	2.113987157	2.01E-13	3.594903896	0.2721625	1.63E-41	ECM_GLYCOPROTEINS	1.700532515	13.20866723
**ZP3**	8.210411688	7.793445658	0.014129186	8.210411688	6.596473864	1.25E-37	ECM_GLYCOPROTEINS	1.053502141	1.24466675

Bladder Urothelial Carcinoma (BLCA)

Signature genes	Expression in tumor	Expression in rest of Pan-Cancer	P-value	Expression in tumor	Expression in normal tissue	P-value	Matrisome category	Fold change vs. Pan-Cancer	Fold change vs. normal
**A2ML1**	8.298537793	3.790856356	1.49E-139	8.298537793	6.286505263	0.01757604	ECM_REGULATORS	2.189093179	1.320055809
**ADAM10**	12.84673052	12.02965498	4.95E-111	12.84673052	11.92630526	1.93E-05	ECM_REGULATORS	1.067921776	1.07717606
**ADAM15**	12.52005235	12.09285726	9.50E-19	12.52005235	11.92044211	0.001126945	ECM_REGULATORS	1.035326233	1.050301007
**ADAM17**	10.42462934	10.14128815	3.46E-07	10.42462934	9.780221053	0.010466552	ECM_REGULATORS	1.027939369	1.065888929
**ADAM8**	10.63341808	8.674758783	9.31E-115	10.63341808	8.756173684	3.19E-07	ECM_REGULATORS	1.225788329	1.21439095
**ADAMTS20**	2.968990376	1.652162821	3.44E-31	2.968990376	1.117489474	0.002129845	ECM_REGULATORS	1.797032555	2.65683968
**ADAMTS7**	9.163955399	8.032070766	6.03E-50	9.163955399	8.112342105	0.000658674	ECM_REGULATORS	1.14092065	1.12963128
**AGRN**	13.90175235	13.21509687	4.99E-26	13.90175235	12.11334737	1.66E-13	ECM_GLYCOPROTEINS	1.051959928	1.147639205
**AGRN**	13.90175235	13.21509687	4.99E-26	13.90175235	12.11334737	1.66E-13	BASEMENT_MEMBRANES	1.051959928	1.147639205
**AMH**	5.236961972	4.848428243	0.001209381	5.236961972	3.027705263	3.72E-05	SECRETED_FACTORS	1.080136017	1.729680242
**ANXA8**	11.65158709	4.785109393	8.50E-275	11.65158709	9.314215789	0.000516304	ECM_AFFILIATED	2.434967758	1.250946655
**ANXA8L1**	10.58020423	4.967643938	1.29E-236	10.58020423	8.980573684	0.008538186	ECM_AFFILIATED	2.129823385	1.178121198
**ARTN**	6.706534272	5.254881498	7.79E-45	6.706534272	5.380321053	0.000752408	SECRETED_FACTORS	1.276248432	1.246493324
**BGLAP**	4.35690939	4.108627296	0.000512104	4.35690939	3.638063158	0.015290344	ECM_GLYCOPROTEINS	1.060429451	1.197590366
**BMP8B**	8.295197183	7.764558468	7.86E-10	8.295197183	6.823	2.17E-05	SECRETED_FACTORS	1.068341132	1.215769776
**C1QTNF6**	10.31103357	9.306514889	8.50E-50	10.31103357	8.121321053	8.88E-11	ECM_AFFILIATED	1.107937148	1.269625163
**CILP2**	7.275610329	6.343231661	2.99E-13	7.275610329	6.138336842	0.012586779	ECM_GLYCOPROTEINS	1.146987958	1.18527388
**COL7A1**	11.97113592	8.794812919	2.10E-154	11.97113592	9.903873684	4.32E-06	COLLAGENS	1.361158677	1.208732694
**CRELD2**	10.47152183	10.36891182	0.01382132	10.47152183	9.878978947	6.36E-05	ECM_GLYCOPROTEINS	1.009895929	1.059980175
**CRLF3**	9.197746244	8.999731443	6.14E-06	9.197746244	8.808263158	0.009024251	SECRETED_FACTORS	1.022002301	1.044217921
**CSF2**	3.33911784	1.803928526	1.50E-50	3.33911784	1.822131579	0.003154082	SECRETED_FACTORS	1.851025576	1.832533873
**CST1**	7.005861268	4.974311134	3.28E-23	7.005861268	1.745789474	3.94E-10	ECM_REGULATORS	1.408408336	4.013004645
**CST4**	2.762000469	2.18324754	5.14E-06	2.762000469	0.305052632	4.39E-05	ECM_REGULATORS	1.265088094	9.054176832
**CST6**	7.884665023	4.590093753	1.06E-93	7.884665023	4.2427	6.03E-08	ECM_REGULATORS	1.717756858	1.858407388
**CSTB**	14.09110117	12.46795209	2.03E-186	14.09110117	13.17565789	0.00142052	ECM_REGULATORS	1.130185701	1.069479891
**CSTL1**	1.070446714	0.531965836	1.95E-28	1.070446714	0.284042105	0.005122323	ECM_REGULATORS	2.012247106	3.768619841
**CTSC**	12.55440023	12.06247845	1.00E-10	12.55440023	11.67459474	0.003377666	ECM_REGULATORS	1.040781153	1.075360689
**CTSV**	7.726790376	7.038075392	1.68E-08	7.726790376	4.742310526	1.06E-07	ECM_REGULATORS	1.097855585	1.62933033
**CXCL10**	8.583020892	8.216557989	0.005029977	8.583020892	6.800926316	0.007449772	SECRETED_FACTORS	1.044600538	1.262037036
**CXCL11**	6.708001408	6.317128309	0.00255089	6.708001408	4.597552632	0.002263282	SECRETED_FACTORS	1.061875124	1.459037437
**FBN2**	7.915163146	7.06951108	2.92E-11	7.915163146	5.910757895	0.001588643	ECM_GLYCOPROTEINS	1.119619597	1.339111377
**FGF11**	8.668219249	7.455610263	1.17E-29	8.668219249	6.440526316	9.36E-09	SECRETED_FACTORS	1.162643827	1.345886784
**FGF22**	2.83116831	2.463297225	5.50E-07	2.83116831	1.718405263	0.002829261	SECRETED_FACTORS	1.149340925	1.647555656
**GPC2**	6.45205446	5.528092229	2.36E-18	6.45205446	4.052568421	1.76E-07	ECM_AFFILIATED	1.167139438	1.592090198
**IBSP**	3.764347887	3.203609632	2.29E-05	3.764347887	0.972478947	1.48E-08	ECM_GLYCOPROTEINS	1.175033265	3.870878539
**IGFBP3**	14.281373	12.2994363	7.27E-78	14.281373	13.22118947	0.020110549	ECM_GLYCOPROTEINS	1.161140451	1.080188211
**KCP**	6.800561268	5.804015945	9.08E-18	6.800561268	5.564557895	0.002300065	ECM_GLYCOPROTEINS	1.171699274	1.222120678
**LAMC2**	11.65576643	9.673943753	1.09E-25	11.65576643	9.537847368	0.00011555	BASEMENT_MEMBRANES	1.204861919	1.2220542
**LAMC2**	11.65576643	9.673943753	1.09E-25	11.65576643	9.537847368	0.00011555	ECM_GLYCOPROTEINS	1.204861919	1.2220542
**MFAP2**	10.59084859	8.904379745	1.85E-35	10.59084859	9.144368421	0.000324495	ECM_GLYCOPROTEINS	1.189397678	1.158182622
**MMP1**	9.425970188	6.509346419	6.66E-53	9.425970188	6.772257895	0.000123623	ECM_REGULATORS	1.448067069	1.391850448
**MMP11**	11.07585939	9.817103461	7.49E-19	11.07585939	7.310157895	8.34E-11	ECM_REGULATORS	1.128220705	1.515132717

**MMP13**	7.082378404	3.879871474	1.21E-64	7.082378404	4.106521053	0.000156658	ECM_REGULATORS	1.825415726	1.724666284
**MMP14**	13.92918075	12.80056123	6.60E-39	13.92918075	13.11161579	0.000477349	ECM_REGULATORS	1.088169534	1.062354249
**MUC1**	11.91359061	10.78351914	1.52E-11	11.91359061	10.87316316	0.04013872	ECM_AFFILIATED	1.104796166	1.095687652
**NGLY1**	10.23802629	9.998852786	2.48E-17	10.23802629	9.861	0.01289566	ECM_REGULATORS	1.023920095	1.038234083
**OGFOD2**	9.251956808	9.057151916	5.19E-09	9.251956808	8.429194737	2.32E-09	ECM_REGULATORS	1.021508405	1.097608621
**PI3**	7.259658216	5.227828624	1.29E-23	7.259658216	4.734778947	0.01322405	ECM_REGULATORS	1.388656503	1.533262333
**PLAU**	11.96070164	10.34115838	5.43E-57	11.96070164	10.29175263	3.55E-05	ECM_REGULATORS	1.156611397	1.162163732
**PLXNA1**	12.07676033	11.39477878	8.98E-28	12.07676033	10.85537895	2.39E-09	ECM_AFFILIATED	1.059850355	1.112513933
**PLXNA3**	10.97967207	10.76621741	8.71E-05	10.97967207	10.06305263	3.96E-06	ECM_AFFILIATED	1.019826337	1.091087612
**PLXNB1**	12.23144671	11.86658829	3.93E-09	12.23144671	11.67024737	0.015825355	ECM_AFFILIATED	1.0307467	1.048088042
**PLXNB2**	13.88605469	13.65677356	2.09E-07	13.88605469	13.33291579	0.00062951	ECM_AFFILIATED	1.016788821	1.041486717
**PODNL1**	7.170835446	6.502546702	1.16E-09	7.170835446	6.005647368	0.005747984	PROTEOGLYCANS	1.102773386	1.1940154
**SDC1**	15.07955986	12.08857194	4.18E-127	15.07955986	13.05611053	3.91E-06	ECM_AFFILIATED	1.247422767	1.15498102
**SEMA3F**	12.19489437	10.42783493	3.47E-85	12.19489437	11.19094211	0.000843151	ECM_AFFILIATED	1.169456023	1.089711148
**SEMA4A**	10.42443779	9.607952449	2.77E-23	10.42443779	9.666847368	0.007532765	ECM_AFFILIATED	1.084980161	1.078369958
**SERPINB7**	3.849569014	2.707530387	9.10E-16	3.849569014	1.931789474	0.011601618	ECM_REGULATORS	1.421800853	1.992747691
**SERPINH1**	13.24035986	12.63826873	3.55E-18	13.24035986	12.50153158	0.000350843	ECM_REGULATORS	1.047640317	1.059099021
**ST14**	13.13931338	11.50465816	2.75E-36	13.13931338	11.02842105	1.24E-09	ECM_REGULATORS	1.142086379	1.191404764
**TGM1**	6.711450469	5.825268317	4.29E-15	6.711450469	5.356357895	0.02186771	ECM_REGULATORS	1.152127268	1.252987683
**TINAGL1**	13.15127606	10.43268998	2.33E-160	13.15127606	11.80057368	0.000259702	ECM_GLYCOPROTEINS	1.260583424	1.114460738
**WNT2**	6.729144366	5.484353537	1.46E-16	6.729144366	5.331673684	0.007961143	SECRETED_FACTORS	1.226971296	1.262107317
**WNT7B**	10.77480469	6.71497389	3.75E-117	10.77480469	8.947252632	0.000455452	SECRETED_FACTORS	1.604593684	1.204258462
**VWA9**	10.72438122	10.62042746	5.26E-06	10.72438122	10.28326316	5.17E-06	ECM_GLYCOPROTEINS	1.009788096	1.042896701
**XCL1**	5.347624648	4.009227797	2.64E-41	5.347624648	4.1363	0.015620247	SECRETED_FACTORS	1.333829086	1.292852222
**ZP1**	3.093282394	2.134271343	1.16E-27	3.093282394	2.069978947	0.024657011	ECM_GLYCOPROTEINS	1.44933886	1.494354519
**ZP3**	8.14135892	7.723563354	2.02E-08	8.14135892	6.9137	5.36E-05	ECM_GLYCOPROTEINS	1.054093628	1.177569018

Breast Invasive Carcinoma (BRCA)

Signature genes	Expression in tumor	Expression in rest of Pan-Cancer	P-value	Expression in tumor	Expression in normal tissue	P-value	Matrisome category	Fold change vs. Pan-Cancer	Fold change vs. normal
**ADAM12**	11.19590792	8.101521606	0	11.19590792	10.1097823	2.37E-13	ECM_REGULATORS	1.381951251	1.107433136
**ADAM15**	12.79624818	12.08449769	2.66E-130	12.79624818	12.21976726	8.01E-14	ECM_REGULATORS	1.058897814	1.047176097
**ADAM19**	9.231910561	9.03442828	7.44E-05	9.231910561	7.865879646	4.47E-31	ECM_REGULATORS	1.021858858	1.173665372
**ADAM8**	8.995519637	8.863581111	0.014808551	8.995519637	7.071266372	3.64E-46	ECM_REGULATORS	1.014885465	1.272122865
**ADAMDEC1**	6.726510809	6.040641488	4.02E-16	6.726510809	4.366595575	3.92E-25	ECM_REGULATORS	1.113542464	1.540447402
**ADAMTS12**	10.0917948	7.748959429	2.68E-247	10.0917948	9.522889381	9.89E-06	ECM_REGULATORS	1.302341933	1.059740841
**ADAMTS14**	7.025099917	6.658865499	1.06E-08	7.025099917	4.002469027	1.13E-67	ECM_REGULATORS	1.054999522	1.755191576
**ADAMTS19**	4.078201073	2.049047957	2.57E-144	4.078201073	3.520021239	0.022404181	ECM_REGULATORS	1.99029069	1.15857286
**ADAMTS2**	11.13285355	9.291421757	1.48E-195	11.13285355	10.8693115	0.033130686	ECM_REGULATORS	1.198186224	1.024246434
**ADAMTS4**	9.926613119	9.072732518	6.73E-50	9.926613119	8.837130088	1.84E-17	ECM_REGULATORS	1.094115042	1.123284711
**ADAMTS6**	6.533623845	5.670886954	2.09E-57	6.533623845	5.649369912	8.14E-14	ECM_REGULATORS	1.152134384	1.156522576
**AEBP1**	14.11790116	12.64730093	5.66E-137	14.11790116	13.32998496	3.52E-10	ECM_GLYCOPROTEINS	1.116277792	1.059108559
**ANXA9**	9.221688201	7.158920787	5.01E-250	9.221688201	7.721292035	6.92E-15	ECM_AFFILIATED	1.288139438	1.194319313
**ARTN**	6.027754455	5.44294193	9.81E-19	6.027754455	3.700948673	1.21E-35	SECRETED_FACTORS	1.107444197	1.628705229
**ASPN**	10.85226584	8.067378749	9.21e-310	10.85226584	9.606749558	6.14E-11	PROTEOGLYCANS	1.345203464	1.129650125
**ASTL**	2.739997277	1.835842965	6.14E-84	2.739997277	1.906823894	1.34E-09	ECM_REGULATORS	1.4925009	1.436943016
**BGN**	14.15985974	13.10687878	6.89E-68	14.15985974	12.11389115	5.61E-58	PROTEOGLYCANS	1.08033804	1.168894417
**BMP8A**	4.429501073	4.227535849	0.000477827	4.429501073	2.102295575	8.48E-49	SECRETED_FACTORS	1.047773746	2.106983016
**C1QTNF3**	8.956812211	7.80146041	1.55E-86	8.956812211	8.403404425	0.000246848	ECM_AFFILIATED	1.148094298	1.065855189
**C1QTNF5**	9.492984241	9.318128108	0.000454985	9.492984241	8.976244248	3.90E-06	ECM_AFFILIATED	1.018765157	1.057567506
**C1QTNF6**	10.15712583	9.294827073	4.40E-94	10.15712583	8.323367257	1.41E-56	ECM_AFFILIATED	1.092771898	1.220314509
**CBLN2**	4.329558086	2.674656668	4.53E-90	4.329558086	2.476847788	8.31E-09	SECRETED_FACTORS	1.618734149	1.748011366
**CCL11**	4.508605776	3.972758329	8.09E-10	4.508605776	1.525281416	9.88E-59	SECRETED_FACTORS	1.134880454	2.955917333
**CCL3L3**	5.792204868	5.477302221	8.97E-08	5.792204868	5.457149558	0.041997464	SECRETED_FACTORS	1.05749229	1.061397495
**CHRD**	8.816179455	7.498267817	1.22E-105	8.816179455	8.403622124	0.015938586	SECRETED_FACTORS	1.175762145	1.049092799
**CILP2**	8.208411386	6.093446911	2.58E-169	8.208411386	5.573426549	2.11E-53	ECM_GLYCOPROTEINS	1.347088357	1.472776453
**CLEC2D**	8.61132797	8.129157606	1.91E-34	8.61132797	8.396769027	0.019864075	ECM_AFFILIATED	1.059313694	1.02555256
**CLEC3A**	5.26575198	0.845965876	0	5.26575198	3.778562832	0.003093898	ECM_AFFILIATED	6.224544192	1.393585925
**CLEC4C**	1.534906353	1.075521466	2.80E-27	1.534906353	1.126680531	0.00789767	ECM_AFFILIATED	1.427127585	1.362326153
**CLEC5A**	6.987062129	6.123436604	1.50E-38	6.987062129	4.417784071	3.15E-50	ECM_AFFILIATED	1.141036085	1.581576197
**CLEC7A**	8.064476733	7.731135127	5.34E-10	8.064476733	7.32900354	5.52E-09	ECM_AFFILIATED	1.043116774	1.100351049
**COL10A1**	10.71988729	6.345894167	0	10.71988729	4.555057522	2.14E-97	COLLAGENS	1.689263485	2.353403276
**COL11A1**	10.98253185	6.949527019	5.10E-278	10.98253185	6.218397345	1.01E-62	COLLAGENS	1.580327959	1.766135427
**COL12A1**	13.67459901	10.98841381	2.24E-272	13.67459901	12.72750177	2.64E-11	COLLAGENS	1.244456138	1.074413444
**COL1A1**	17.03852649	14.43435341	6.27E-178	17.03852649	14.88682301	2.66E-37	COLLAGENS	1.180414945	1.144537453
**COL1A2**	16.69530239	14.2028013	1.11E-218	16.69530239	15.43880796	7.99E-17	COLLAGENS	1.175493625	1.081385456
**COL24A1**	6.21422632	5.215554943	9.82E-60	6.21422632	5.11490177	8.81E-23	COLLAGENS	1.191479409	1.214925838
**COL3A1**	16.56689051	13.96613871	2.83E-193	16.56689051	15.33594779	2.59E-15	COLLAGENS	1.186218386	1.080265187
**COL5A1**	13.41435545	11.60679847	1.30E-134	13.41435545	11.80284602	7.73E-30	COLLAGENS	1.155732606	1.136535665
**COL5A2**	13.52663259	11.24220768	2.07E-265	13.52663259	12.10333186	1.84E-23	COLLAGENS	1.20320074	1.117595779
**COL6A1**	14.25884546	13.08448322	2.69E-112	14.25884546	13.96310708	0.010616075	COLLAGENS	1.089752283	1.021179984
**COL6A1**	14.25884546	13.08448322	2.69E-112	14.25884546	13.96310708	0.010616075	BASEMENT_MEMBRANES	1.089752283	1.021179984

**COL6A3**	14.9025684	12.33135763	3.29E-219	14.9025684	14.35101062	3.24E-05	COLLAGENS	1.208509951	1.038433375
**COL6A3**	14.9025684	12.33135763	3.29E-219	14.9025684	14.35101062	3.24E-05	BASEMENT_MEMBRANES	1.208509951	1.038433375
**COL8A1**	11.25208771	9.600373445	1.42E-93	11.25208771	10.37454602	1.70E-09	COLLAGENS	1.172046876	1.084586033
**COL9A2**	8.980872937	8.720633941	6.77E-05	8.980872937	8.568457522	0.012601236	COLLAGENS	1.029841752	1.048131815
**COMP**	10.73491832	6.775507407	1.28E-271	10.73491832	6.659666372	1.14E-58	ECM_GLYCOPROTEINS	1.584371129	1.611930346
**CPN2**	2.953881353	2.172646889	4.72E-17	2.953881353	2.144440708	2.07E-07	ECM_REGULATORS	1.359577282	1.377460026
**CST1**	6.66099571	4.906725585	6.61E-45	6.66099571	1.230035398	4.82E-51	ECM_REGULATORS	1.357523586	5.415287819
**CST11**	0.390252888	0.184632884	5.70E-26	0.390252888	0.199652212	0.033106205	ECM_REGULATORS	2.113669457	1.954663478
**CST2**	4.861715842	3.441381013	2.21E-61	4.861715842	1.595419469	7.98E-39	ECM_REGULATORS	1.412722341	3.047296298
**CST4**	3.596259571	1.970130491	1.28E-99	3.596259571	0.398417699	3.58E-27	ECM_REGULATORS	1.825391561	9.026354951
**CST5**	2.551666337	1.531283838	8.85E-50	2.551666337	0.572423894	2.47E-10	ECM_REGULATORS	1.666357518	4.457651688
**CST6**	5.603691089	4.902758889	3.75E-12	5.603691089	4.184513274	3.75E-12	ECM_REGULATORS	1.142966892	1.339150033
**CST9L**	1.381228713	0.141443978	0	1.381228713	0.780349558	0.001502618	ECM_REGULATORS	9.765199821	1.77001281
**CTHRC1**	11.12223218	8.809608399	2.07E-203	11.12223218	8.909548673	6.80E-51	ECM_GLYCOPROTEINS	1.26251153	1.248349674
**CTSZ**	12.90454992	12.82921038	0.025801514	12.90454992	12.7363885	0.017109774	ECM_REGULATORS	1.0058725	1.013203226
**CXCL10**	9.270857096	8.278241186	8.30E-34	9.270857096	6.749662832	1.09E-34	SECRETED_FACTORS	1.119906619	1.373528919
**CXCL11**	7.374035479	6.360275914	1.22E-35	7.374035479	4.805169912	1.47E-32	SECRETED_FACTORS	1.159389243	1.534604523
**CXCL13**	7.47847302	6.199152873	1.64E-35	7.47847302	5.382593805	7.18E-12	SECRETED_FACTORS	1.206370156	1.389380899
**CXCL9**	10.04744134	8.820906361	3.01E-42	10.04744134	8.153427434	3.21E-16	SECRETED_FACTORS	1.139048633	1.232296653
**ECM1**	10.89193003	9.933208323	3.38E-57	10.89193003	9.980565487	8.28E-08	ECM_GLYCOPROTEINS	1.096516823	1.091313919
**EGLN2**	11.56296997	11.41214976	1.06E-13	11.56296997	11.02280973	3.34E-11	ECM_REGULATORS	1.013215758	1.049003861
**EGLN3**	9.838346617	9.588150307	0.000550771	9.838346617	8.81889646	2.20E-12	ECM_REGULATORS	1.026094325	1.115598381
**EMILIN1**	10.94591188	10.63250712	3.07E-08	10.94591188	10.58356814	0.004561431	ECM_GLYCOPROTEINS	1.029476093	1.034236444
**EPO**	2.382876815	2.091021251	2.90E-06	2.382876815	1.449641593	9.31E-07	SECRETED_FACTORS	1.139575609	1.643769623
**EPYC**	4.802066914	2.286165035	6.81E-199	4.802066914	1.204872566	1.46E-40	PROTEOGLYCANS	2.100490052	3.985539258
**F12**	7.398298927	7.213070739	0.014121616	7.398298927	4.965949558	1.62E-47	ECM_REGULATORS	1.025679519	1.489805493
**F7**	6.188980528	3.564751849	3.47E-189	6.188980528	4.734643363	5.94E-09	ECM_REGULATORS	1.736160269	1.307169316
**FBN2**	7.363150248	7.174418415	0.017468006	7.363150248	5.314756637	6.84E-28	ECM_GLYCOPROTEINS	1.02630622	1.385416257
**FN1**	16.61292409	14.97522479	2.13E-117	16.61292409	14.16118673	2.15E-50	ECM_GLYCOPROTEINS	1.109360582	1.173130784
**FNDC1**	10.55541436	7.880639849	5.32E-182	10.55541436	8.990941593	5.46E-20	ECM_GLYCOPROTEINS	1.339410829	1.174005442
**GPC4**	10.52031955	9.860518846	6.49E-23	10.52031955	9.99779469	6.28E-05	ECM_AFFILIATED	1.066913386	1.052264012
**GREM1**	10.02564703	7.748169747	1.19E-101	10.02564703	9.21980885	5.34E-06	ECM_AFFILIATED	1.293937453	1.087402916
**HPX**	7.685981353	4.633679116	5.00E-235	7.685981353	6.819457522	0.000210855	ECM_AFFILIATED	1.658721107	1.127066387
**HTRA3**	11.1885882	9.009517531	4.55E-198	11.1885882	10.80110354	0.00963821	ECM_REGULATORS	1.241863192	1.035874544
**HTRA4**	3.63617104	3.344919191	7.39E-07	3.63617104	2.610073451	1.87E-12	ECM_REGULATORS	1.08707291	1.393129775
**IBSP**	4.822415842	3.067219224	5.81E-107	4.822415842	0.582378761	2.49E-64	ECM_GLYCOPROTEINS	1.572243615	8.280548955
**IGFALS**	5.341866007	4.327864173	1.79E-40	5.341866007	3.742167257	5.38E-12	ECM_GLYCOPROTEINS	1.234296132	1.427479223
**ITIH6**	6.644765429	2.185747159	0	6.644765429	5.766893805	6.35E-05	ECM_REGULATORS	3.040043036	1.152226078
**KAZALD1**	8.170065347	7.313407137	8.21E-58	8.170065347	7.472739823	5.69E-07	ECM_REGULATORS	1.11713531	1.093315911
**LGALS8**	12.09642269	11.18617164	9.45E-261	12.09642269	11.81871504	0.000294775	ECM_AFFILIATED	1.081372884	1.023497279
**LMAN1**	12.64168894	12.34899995	5.67E-31	12.64168894	12.46526106	0.004513533	ECM_AFFILIATED	1.023701433	1.014153565
**LOXL1**	10.53321056	9.065306922	1.42E-135	10.53321056	9.360351327	6.94E-26	ECM_REGULATORS	1.161925421	1.125300771
**LOXL2**	10.98485809	10.06401344	1.56E-67	10.98485809	10.67768761	0.002987589	ECM_REGULATORS	1.091498749	1.028767509
**LRG1**	9.093024175	8.25288841	2.79E-20	9.093024175	8.268236283	0.001134478	ECM_GLYCOPROTEINS	1.101798997	1.099753788
**MATN3**	8.880825495	6.175853337	0	8.880825495	6.888699115	8.33E-31	ECM_GLYCOPROTEINS	1.437991644	1.289187602
**MEPE**	1.384993152	0.961013164	3.53E-20	1.384993152	0.745264602	2.30E-05	ECM_GLYCOPROTEINS	1.441180208	1.858391165
**MFAP2**	10.33492979	8.962394997	2.08E-62	10.33492979	8.470486726	2.38E-39	ECM_GLYCOPROTEINS	1.153143751	1.220110499

**MMP10**	5.356848185	4.605390232	1.25E-11	5.356848185	3.10069646	2.46E-20	ECM_REGULATORS	1.163169225	1.727627407
**MMP11**	12.44898465	9.670908582	6.63E-235	12.44898465	7.228453097	6.74E-98	ECM_REGULATORS	1.287261124	1.722219745
**MMP13**	7.764369967	3.946611903	1.22E-222	7.764369967	2.410757522	1.01E-65	ECM_REGULATORS	1.96735077	3.220717926
**MMP14**	13.5252085	12.86777279	8.58E-36	13.5252085	12.61523274	2.38E-17	ECM_REGULATORS	1.051091647	1.072133093
**MMP17**	7.250313531	6.509288442	3.34E-26	7.250313531	6.794683186	0.001449483	ECM_REGULATORS	1.113841182	1.067056893
**MMP25**	6.704895215	6.279096507	1.09E-20	6.704895215	6.148525664	0.000294572	ECM_REGULATORS	1.067812098	1.090488286
**MMP3**	7.705482096	4.169840539	5.49E-205	7.705482096	5.800159292	5.54E-18	ECM_REGULATORS	1.847908097	1.32849491
**MMP8**	2.153475743	1.971380733	0.008960725	2.153475743	1.63380177	0.006195267	ECM_REGULATORS	1.092369275	1.318076515
**MMP9**	10.48125355	9.226251439	3.54E-48	10.48125355	8.034178761	7.38E-30	ECM_REGULATORS	1.136025136	1.304583064
**MUC1**	13.19909579	10.57323003	1.40E-154	13.19909579	11.04934513	1.65E-23	ECM_AFFILIATED	1.248350387	1.194559101
**MUC19**	3.101980693	1.334942221	1.22E-201	3.101980693	2.287080531	0.002309737	ECM_AFFILIATED	2.323681613	1.356305845
**MUC5B**	8.942969472	5.717493283	3.02E-121	8.942969472	8.176951327	0.02555344	ECM_AFFILIATED	1.564141667	1.093680164
**MXRA5**	13.23535982	10.75513288	2.64E-235	13.23535982	12.54451327	5.01E-08	ECM_GLYCOPROTEINS	1.230608674	1.05507161
**NELL2**	8.656212211	7.734769057	5.46E-29	8.656212211	7.44449469	1.47E-07	ECM_GLYCOPROTEINS	1.119130015	1.162766927
**NID2**	10.24610561	9.071591817	2.69E-89	10.24610561	9.864915044	0.001710855	BASEMENT_MEMBRANES	1.129471632	1.038641039
**NID2**	10.24610561	9.071591817	2.69E-89	10.24610561	9.864915044	0.001710855	ECM_GLYCOPROTEINS	1.129471632	1.038641039
**NPNT**	11.77287764	10.01168541	3.34E-104	11.77287764	10.62509027	3.54E-11	ECM_GLYCOPROTEINS	1.17591366	1.108026129
**NPNT**	11.77287764	10.01168541	3.34E-104	11.77287764	10.62509027	3.54E-11	BASEMENT_MEMBRANES	1.17591366	1.108026129
**OVGP1**	6.545292822	6.288373477	3.82E-07	6.545292822	6.278546018	0.015834342	ECM_AFFILIATED	1.040856248	1.042485442
**P4HA1**	11.2767755	11.12019723	2.66E-07	11.2767755	10.62365929	1.40E-16	ECM_REGULATORS	1.01408053	1.061477518
**P4HA2**	10.8671066	10.6819488	2.33E-05	10.8671066	10.36848584	2.40E-13	ECM_REGULATORS	1.01733371	1.048090027
**P4HA3**	7.752185891	6.198792765	7.98E-158	7.752185891	5.441364602	4.49E-51	ECM_REGULATORS	1.250596073	1.424676797
**P4HTM**	10.39121955	10.29687822	0.017261499	10.39121955	9.792789381	3.37E-10	ECM_REGULATORS	1.00916213	1.061109266
**PLAU**	11.14789464	10.51843826	1.54E-22	11.14789464	10.34973274	2.06E-11	ECM_REGULATORS	1.05984314	1.077119083
**PLXNA3**	11.03704348	10.69795725	2.85E-24	11.03704348	10.14113717	6.08E-34	ECM_AFFILIATED	1.031696353	1.088343772
**PLXNC1**	10.24305536	9.587126836	3.52E-40	10.24305536	9.93779646	0.003134786	ECM_AFFILIATED	1.068417633	1.030716961
**PODNL1**	8.022631188	6.462424097	8.55E-123	8.022631188	7.432354867	1.63E-05	PROTEOGLYCANS	1.241427531	1.079419825
**POSTN**	14.51958952	10.38214502	0	14.51958952	12.87884602	8.10E-24	ECM_GLYCOPROTEINS	1.398515383	1.127398332
**PXDNL**	5.735522442	4.37468372	7.78E-109	5.735522442	5.006071681	0.000226227	ECM_GLYCOPROTEINS	1.311071339	1.145713207
**SDC1**	13.08792186	12.38498878	6.79E-19	13.08792186	11.1843	3.13E-34	ECM_AFFILIATED	1.056756861	1.170204829
**SDC4**	13.23332781	12.80626996	3.90E-16	13.23332781	13.01846195	0.016609306	ECM_AFFILIATED	1.033347559	1.016504704
**SEMA3F**	11.5286231	10.50643084	1.01E-76	11.5286231	10.68371062	3.28E-21	ECM_AFFILIATED	1.097292057	1.079084179
**SEMA4A**	10.26598795	9.652725305	3.88E-35	10.26598795	9.53340177	1.29E-13	ECM_AFFILIATED	1.063532591	1.076844153
**SEMA4C**	10.8784269	10.76954493	0.000115669	10.8784269	10.59589381	9.94E-05	ECM_AFFILIATED	1.010110174	1.026664394
**SERPINA12**	2.273342409	0.784366038	6.28E-197	2.273342409	1.763317699	0.003463727	ECM_REGULATORS	2.898318259	1.289241531
**SERPINA3**	13.40453251	8.72563917	4.71E-293	13.40453251	12.56724248	0.000112265	ECM_REGULATORS	1.536223565	1.066624801
**SERPINA9**	2.433109158	1.232506092	7.94E-103	2.433109158	1.019859292	1.54E-15	ECM_REGULATORS	1.974115321	2.385730245
**SERPINH1**	12.9210047	12.66867659	2.23E-09	12.9210047	12.24689381	1.03E-14	ECM_REGULATORS	1.01991748	1.055043418
**SPOCK1**	10.01728688	9.265470566	7.67E-19	10.01728688	9.688836283	0.025913676	PROTEOGLYCANS	1.08114173	1.033899902
**ST14**	12.17680759	11.66179909	5.94E-11	12.17680759	11.06404779	2.21E-23	ECM_REGULATORS	1.044162011	1.100574385
**SULF1**	12.71079884	10.817344	1.68E-145	12.71079884	11.42015575	1.60E-22	ECM_REGULATORS	1.175038794	1.113014491
**SULF2**	12.76129191	11.67624188	1.06E-103	12.76129191	12.12462301	1.12E-08	ECM_REGULATORS	1.09292802	1.052510408
**THBS2**	13.01113729	10.91948638	1.47E-203	13.01113729	12.29882124	4.49E-07	ECM_GLYCOPROTEINS	1.191552133	1.057917425
**TNFAIP6**	8.225396782	6.293037617	4.43E-144	8.225396782	7.612272566	3.77E-07	ECM_GLYCOPROTEINS	1.307063025	1.080544175
**TNR**	3.775593069	3.526543278	0.019248055	3.775593069	2.500474336	1.26E-12	ECM_GLYCOPROTEINS	1.070621504	1.509950738
**VCAN**	13.26468837	11.47016127	3.51E-142	13.26468837	12.02102655	2.37E-17	PROTEOGLYCANS	1.156451775	1.103457206
**WISP1**	8.78589604	7.146855148	3.90E-124	8.78589604	5.790788496	1.63E-66	ECM_GLYCOPROTEINS	1.22933736	1.517219295

**WNT2**	7.841377888	5.318455288	7.54E-174	7.841377888	7.12390885	6.33E-06	SECRETED_FACTORS	1.474371309	1.100712832
**WNT7B**	8.722412624	7.031001003	1.74E-52	8.722412624	6.962693805	8.76E-20	SECRETED_FACTORS	1.240564839	1.252735345
**WNT9A**	8.119590347	6.78354759	1.15E-92	8.119590347	7.795253097	0.006722567	SECRETED_FACTORS	1.196953399	1.04160702
**VWA9**	10.79560215	10.60224113	5.80E-44	10.79560215	10.5004469	1.74E-13	ECM_GLYCOPROTEINS	1.018237749	1.028108827
**ZP3**	8.164847855	7.748374706	3.71E-20	8.164847855	7.484613274	1.09E-10	ECM_GLYCOPROTEINS	1.053749743	1.090884399
**ZP4**	0.627632096	0.328370016	3.30E-21	0.627632096	0.053215044	1.56E-06	ECM_GLYCOPROTEINS	1.91135629	11.79426053
**ZPLD1**	3.564240264	2.122234415	2.85E-112	3.564240264	2.813462832	0.000335311	ECM_GLYCOPROTEINS	1.679475292	1.266851733

Cervical and endocervical cancer (CESC)

Signature genes	Expression in tumor	Expression in rest of Pan-Cancer	P-value	Expression in tumor	Expression in normal tissue	P-value	Matrisome category	Fold change vs. Pan-Cancer	Fold change vs. normal
A2ML1	9.514129126	3.790856356	3.82E-163	9.514129126	4.331966667	0.018090263	ECM_REGULATORS	2.509757224	2.196260927
ADAM17	10.49418641	10.14128815	5.50E-08	10.49418641	8.909466667	0.0128704	ECM_REGULATORS	1.034798169	1.177869204
ADAM8	10.43366667	8.674758783	1.90E-69	10.43366667	7.911233333	0.002841297	ECM_REGULATORS	1.20276159	1.318841984
ADAMDEC1	6.688965696	6.075688093	0.000148448	6.688965696	2.300133333	0.000776409	ECM_REGULATORS	1.100939613	2.90807737
AGRN	13.66198803	13.21509687	3.90E-09	13.66198803	11.835	0.000661704	ECM_GLYCOPROTEINS	1.033816715	1.154371612
AGRN	13.66198803	13.21509687	3.90E-09	13.66198803	11.835	0.000661704	BASEMENT_MEMBRANES	1.033816715	1.154371612
AMH	6.195443366	4.848428243	8.55E-22	6.195443366	2.708133333	0.007704271	SECRETED_FACTORS	1.277825113	2.287717259
ANXA1	14.4732835	12.33281074	4.07E-80	14.4732835	12.6937	0.040766622	ECM_AFFILIATED	1.173559199	1.14019423
ANXA2	15.80602945	14.32990725	4.13E-65	15.80602945	14.39736667	0.002895733	ECM_AFFILIATED	1.103009892	1.097841697
ANXA3	10.71969223	9.057695951	7.63E-26	10.71969223	8.7174	0.019638957	ECM_AFFILIATED	1.183489962	1.229689154
ANXA8	11.67643366	4.785109393	2.82E-204	11.67643366	4.564766667	0.000295087	ECM_AFFILIATED	2.440160234	2.557947538
ANXA8L1	10.66219126	4.967643938	1.72E-178	10.66219126	4.1037	0.000817142	ECM_AFFILIATED	2.146327594	2.598189746
ARTN	7.170338188	5.254881498	2.86E-56	7.170338188	4.701466667	0.0170519	SECRETED_FACTORS	1.364509969	1.525127943
BMP8B	8.575183495	7.764558468	6.45E-16	8.575183495	6.360633333	0.000113741	SECRETED_FACTORS	1.10440066	1.34816504
CCL18	7.500533333	6.932424042	0.003694628	7.500533333	2.386666667	0.000242165	SECRETED_FACTORS	1.081949588	3.142681564
CCL20	8.072576052	5.783442632	8.18E-31	8.072576052	4.917366667	0.031984335	SECRETED_FACTORS	1.395808097	1.641646149
CCL22	7.255526861	5.806754854	1.70E-26	7.255526861	4.419433333	0.003559671	SECRETED_FACTORS	1.249497704	1.641732393
CLEC7A	8.239785113	7.68305222	3.15E-08	8.239785113	5.961333333	0.044479298	ECM_AFFILIATED	1.072462464	1.382205063
COL17A1	10.73218964	7.260558424	1.71E-53	10.73218964	5.7598	0.007505702	COLLAGENS	1.47814934	1.863292066
COL7A1	12.93183301	8.794812919	6.81E-190	12.93183301	9.363666667	0.000491744	COLLAGENS	1.470393188	1.381065075
CRLF3	9.310138511	8.999731443	1.45E-09	9.310138511	8.211066667	0.003994024	SECRETED_FACTORS	1.034490703	1.133852505
CSF2	3.413646602	1.803928526	1.14E-41	3.413646602	0.7561	0.016659252	SECRETED_FACTORS	1.892340274	4.514808361
CSH2	2.149573463	0.354145385	8.59E-179	2.149573463	0	0.046514588	SECRETED_FACTORS	6.069748618	Inf
CSPG5	6.559236246	6.174499793	0.01079021	6.559236246	4.634366667	0.028647139	ECM_AFFILIATED	1.062310546	1.415346846
CST6	7.385954369	4.590093753	4.15E-51	7.385954369	3.7497	0.009790189	ECM_REGULATORS	1.609107519	1.969745411
CSTA	11.43617476	7.004722236	4.55E-186	11.43617476	4.915966667	0.000953433	ECM_REGULATORS	1.632637865	2.326332852
CSTB	14.16081489	12.46795209	1.21E-149	14.16081489	11.84826667	0.002293596	ECM_REGULATORS	1.135777134	1.195180298
CTSC	12.51150065	12.06247845	3.96E-07	12.51150065	10.89746667	0.007951758	ECM_REGULATORS	1.037224705	1.148110935
CTSV	10.61893883	7.038075392	1.32E-137	10.61893883	4.943066667	2.38E-11	ECM_REGULATORS	1.508784468	2.148249164
CXCL1	10.39414887	6.587876067	8.26E-92	10.39414887	6.949066667	0.015082424	SECRETED_FACTORS	1.577769339	1.495761858
CXCL10	10.13062816	8.216557989	3.17E-36	10.13062816	5.570766667	0.002130547	SECRETED_FACTORS	1.232952797	1.818533922
CXCL11	8.039445955	6.317128309	3.30E-30	8.039445955	4.231933333	0.014145033	SECRETED_FACTORS	1.272642499	1.899709972
CXCL13	7.595131715	6.126360231	6.26E-14	7.595131715	4.0281	0.024522538	SECRETED_FACTORS	1.239746184	1.885537031
CXCL8	9.849922654	7.774415085	7.59E-38	9.849922654	6.272466667	0.006527141	SECRETED_FACTORS	1.266966395	1.570342766
CXCL9	9.975141748	8.855705986	5.50E-11	9.975141748	5.6245	0.003535258	SECRETED_FACTORS	1.126408415	1.773516179
EGFL6	7.558454369	5.613663158	4.76E-28	7.558454369	5.244433333	0.019497922	SECRETED_FACTORS	1.346438886	1.441233759
EPGN	3.593149838	1.466454027	4.48E-71	3.593149838	0	0.02952027	SECRETED_FACTORS	2.450230128	Inf
F12	8.728497411	7.072578026	3.38E-30	8.728497411	3.194466667	7.86E-12	ECM_REGULATORS	1.23413236	2.732380182
FGF11	8.97988932	7.455610263	8.71E-34	8.97988932	6.3231	0.011927717	SECRETED_FACTORS	1.204447256	1.42017196
GPC1	12.49667476	11.38650372	1.62E-37	12.49667476	10.93086667	0.026321674	ECM_AFFILIATED	1.097498851	1.143246472
GPC2	6.396338511	5.528092229	1.68E-12	6.396338511	3.9654	0.013366729	ECM_AFFILIATED	1.157060745	1.613037401

**HAPLN1**	5.930613916	5.192622214	3.65E-05	5.930613916	0.212233333	5.53E-06	PROTEOGLYCANS	1.142123126	27.94383815
**HMSD**	6.003397411	2.728630736	3.59E-178	6.003397411	1.237566667	0.000130126	ECM_REGULATORS	2.200150182	4.850968899
**HPSE**	9.087242395	7.674142349	1.05E-59	9.087242395	7.656833333	0.029770211	ECM_REGULATORS	1.184137847	1.186814705
**KCP**	8.046039806	5.804015945	3.76E-60	8.046039806	4.7788	0.007103291	ECM_GLYCOPROTEINS	1.38628837	1.683694611
**LAMA5**	13.05631456	12.24232562	3.13E-22	13.05631456	12.05316667	0.042885483	BASEMENT_MEMBRANES	1.066489731	1.083226917
**LAMA5**	13.05631456	12.24232562	3.13E-22	13.05631456	12.05316667	0.042885483	ECM_GLYCOPROTEINS	1.066489731	1.083226917
**LAMB3**	13.57687573	10.41794833	8.09E-89	13.57687573	8.3254	6.97E-07	BASEMENT_MEMBRANES	1.303219722	1.630777588
**LAMB3**	13.57687573	10.41794833	8.09E-89	13.57687573	8.3254	6.97E-07	ECM_GLYCOPROTEINS	1.303219722	1.630777588
**LAMC2**	12.93690647	9.673943753	5.17E-49	12.93690647	8.3977	5.24E-05	BASEMENT_MEMBRANES	1.337293952	1.540529725
**LAMC2**	12.93690647	9.673943753	5.17E-49	12.93690647	8.3977	5.24E-05	ECM_GLYCOPROTEINS	1.337293952	1.540529725
**LGALS7**	7.793349838	2.063296659	1.17E-298	7.793349838	2.243566667	0.007311368	ECM_AFFILIATED	3.77713491	3.473643085
**LGALS9B**	5.092482848	2.503604669	4.17E-71	5.092482848	1.7004	0.017770363	ECM_AFFILIATED	2.034060293	2.99487347
**LGALS9C**	5.864445955	3.418625566	1.35E-66	5.864445955	2.354566667	0.008609733	ECM_AFFILIATED	1.715439682	2.490668894
**MMP1**	9.011963754	6.509346419	2.03E-29	9.011963754	4.3944	0.005724577	ECM_REGULATORS	1.384465225	2.050783669
**MMP11**	11.75408317	9.817103461	1.07E-31	11.75408317	9.383	0.029076794	ECM_REGULATORS	1.197306641	1.252699901
**MMP12**	8.778456634	5.248954386	2.79E-58	8.778456634	4.290566667	0.000830493	ECM_REGULATORS	1.672420065	2.045990033
**MMP3**	7.5063411	4.050837788	2.61E-60	7.5063411	2.703666667	0.004190837	ECM_REGULATORS	1.853034234	2.776355973
**MMP9**	10.2367754	9.218202699	4.04E-10	10.2367754	6.0924	0.000256134	ECM_REGULATORS	1.110495803	1.680253333
**MUC15**	5.837216828	4.400525729	1.47E-09	5.837216828	1.503933333	0.017843318	ECM_AFFILIATED	1.326481695	3.881300254
**MUC4**	11.87259126	6.398793851	4.39E-138	11.87259126	7.844533333	0.029328503	ECM_AFFILIATED	1.855442063	1.513485985
**OGFOD2**	9.232738188	9.057151916	6.31E-06	9.232738188	8.5622	0.024241545	ECM_REGULATORS	1.019386478	1.078313773
**PI3**	11.10691424	5.227828624	1.10E-134	11.10691424	6.0134	0.009358876	ECM_REGULATORS	2.124575046	1.847027346
**PLAU**	11.87068964	10.34115838	4.01E-38	11.87068964	9.018966667	0.00161957	ECM_REGULATORS	1.14790715	1.316191764
**PLXNA1**	12.20231456	11.39477878	2.30E-28	12.20231456	10.56816667	0.001336495	ECM_AFFILIATED	1.070868931	1.15462927
**PLXNB1**	12.06207217	11.86658829	0.006690689	12.06207217	10.93726667	0.008301909	ECM_AFFILIATED	1.016473469	1.102841554
**PLXNB2**	13.94196828	13.65677356	3.09E-08	13.94196828	13.2648	0.042473411	ECM_AFFILIATED	1.020883023	1.051050018
**PODNL1**	7.140645955	6.502546702	6.16E-07	7.140645955	4.5077	0.002742882	PROTEOGLYCANS	1.098130668	1.584099642
**PRSS3**	4.727119094	4.291396093	0.044699264	4.727119094	0.8885	0.042949547	ECM_REGULATORS	1.101534091	5.320336628
**SDC1**	14.73379029	12.08857194	1.10E-74	14.73379029	8.528033333	5.65E-14	ECM_AFFILIATED	1.218819755	1.727689107
**SDC4**	13.62304595	12.74728346	8.00E-18	13.62304595	12.4952	0.031706853	ECM_AFFILIATED	1.068701893	1.090262337
**SEMA3F**	12.14108803	10.42783493	9.07E-60	12.14108803	10.05543333	7.97E-05	ECM_AFFILIATED	1.164296147	1.207415695
**SEMA4A**	10.47042783	9.607952449	2.31E-19	10.47042783	8.0677	1.90E-05	ECM_AFFILIATED	1.089766825	1.297820672
**SEMA4B**	13.33532298	11.68045987	5.56E-124	13.33532298	10.7566	7.00E-05	ECM_AFFILIATED	1.141677907	1.239734022
**SERPINB13**	9.784079935	1.408272159	0	9.784079935	4.175566667	0.031561843	ECM_REGULATORS	6.947577477	2.343174164
**SERPINB2**	8.467252751	3.304195244	8.93E-162	8.467252751	2.5344	0.001774744	ECM_REGULATORS	2.562576399	3.340929905
**SERPINB3**	12.38856117	2.392725392	0	12.38856117	7.435733333	0.018968262	ECM_REGULATORS	5.177594223	1.66608465
**SERPINB4**	10.97515987	1.89524101	0	10.97515987	4.828933333	0.003782552	ECM_REGULATORS	5.790904593	2.272791756
**SERPINB5**	12.02747832	5.178847333	3.07E-146	12.02747832	5.156433333	2.66E-05	ECM_REGULATORS	2.322423803	2.332518921
**SERPINB7**	7.473047249	2.707530387	2.35E-177	7.473047249	2.642033333	0.00314979	ECM_REGULATORS	2.760097277	2.828521183
**SERPINB8**	9.739896117	8.658668862	4.14E-56	9.739896117	8.0066	0.005448461	ECM_REGULATORS	1.124872226	1.216483416
**ST14**	13.35476408	11.50465816	4.24E-34	13.35476408	8.803366667	3.50E-14	ECM_REGULATORS	1.160813636	1.517006457
**TGM1**	9.783284466	5.825268317	2.80E-187	9.783284466	5.4987	0.012739685	ECM_REGULATORS	1.679456453	1.779199532
**TGM5**	5.651428479	2.568398792	1.48E-109	5.651428479	1.7551	0.009272595	ECM_REGULATORS	2.200370323	3.220003692
**WNT10A**	7.613164078	5.674497355	8.02E-36	7.613164078	3.360133333	0.001001042	SECRETED_FACTORS	1.341645542	2.265732732
**WNT3A**	5.469840453	2.46023867	1.39E-99	5.469840453	1.621166667	0.004512346	SECRETED_FACTORS	2.223296674	3.374014878
**WNT7B**	10.31688641	6.71497389	6.05E-69	10.31688641	4.009733333	4.52E-10	SECRETED_FACTORS	1.536400078	2.572960731

**VWA9**	10.72309709	10.62042746	0.00011719	10.72309709	10.27616667	0.046842758	ECM_GLYCOPROTEINS	1.009667184	1.04349194

Cholangiocarcinoma (CHOL)

Signature genes	Expression in tumor	Expression in rest of Pan-Cancer	P-value	Expression in tumor	Expression in normal tissue	P-value	Matrisome category	Fold change vs. Pan-Cancer	Fold change vs. normal
**ADAM28**	8.717384444	8.144706616	0.042392565	8.717384444	5.929011111	0.000248605	ECM_REGULATORS	1.070312886	1.470293154
**ADAMTS10**	9.514424444	7.635274144	1.44E-16	9.514424444	8.412066667	0.020472007	ECM_REGULATORS	1.246114319	1.131044822
**ADAMTS16**	6.491073333	5.21190931	0.001028455	6.491073333	4.210088889	0.032711324	ECM_REGULATORS	1.245430983	1.541790092
**AGRN**	13.84950667	13.3278158	0.007281928	13.84950667	12.04275556	0.000152247	ECM_GLYCOPROTEINS	1.039143013	1.150028048
**AGRN**	13.84950667	13.3278158	0.007281928	13.84950667	12.04275556	0.000152247	BASEMENT_MEMBRANES	1.039143013	1.150028048
**AMH**	5.658886667	4.921354895	0.040457558	5.658886667	3.104122222	0.003168167	SECRETED_FACTORS	1.149863562	1.823023148
**ANXA11**	13.31701333	12.83159322	0.001705166	13.31701333	11.92021111	1.42E-05	ECM_AFFILIATED	1.037830073	1.117179319
**ANXA13**	10.99236	3.760670871	1.28E-50	10.99236	8.991088889	0.0417013	ECM_AFFILIATED	2.922978473	1.222583842
**ANXA3**	10.08162	9.322621984	0.048811243	10.08162	7.1658	0.00045096	ECM_AFFILIATED	1.081414651	1.406907812
**ANXA4**	14.41420444	11.92812769	1.44E-32	14.41420444	12.82972222	0.001451373	ECM_AFFILIATED	1.208421373	1.123500899
**BGLAP**	4.642831111	4.098312274	0.011510355	4.642831111	3.158611111	0.008513218	ECM_GLYCOPROTEINS	1.132864165	1.469896403
**CCL20**	8.991948889	5.85601887	4.79E-10	8.991948889	6.502677778	0.003775222	SECRETED_FACTORS	1.53550545	1.382807083
**CLCF1**	9.167851111	7.740658906	8.73E-08	9.167851111	7.187466667	0.000646807	SECRETED_FACTORS	1.184376062	1.275533027
**COL4A3**	7.023937778	5.757719739	0.004554614	7.023937778	5.698933333	0.042228547	COLLAGENS	1.219916581	1.232500429
**COL4A3**	7.023937778	5.757719739	0.004554614	7.023937778	5.698933333	0.042228547	BASEMENT_MEMBRANES	1.219916581	1.232500429
**CST2**	5.056413333	3.647997336	0.00092016	5.056413333	2.242733333	0.000891252	ECM_REGULATORS	1.386079229	2.254576261
**CTSC**	12.73839556	12.19572184	0.017464141	12.73839556	10.92235556	0.000382639	ECM_REGULATORS	1.044497055	1.166268164
**CTSH**	13.55254	12.11308902	1.21E-11	13.55254	12.36491111	0.008928358	ECM_REGULATORS	1.118834343	1.096048316
**CXCL1**	9.382997778	6.974505339	1.02E-06	9.382997778	7.437911111	0.022260708	SECRETED_FACTORS	1.345328066	1.261509803
**CXCL8**	10.60451111	8.042846702	1.33E-09	10.60451111	8.618811111	0.027582229	SECRETED_FACTORS	1.318502205	1.230391405
**EDA**	8.162688889	7.291974603	0.001283012	8.162688889	7.117877778	0.024419953	SECRETED_FACTORS	1.119407202	1.146786886
**FGF12**	7.493855556	6.303783664	0.000854937	7.493855556	5.173666667	0.004222516	SECRETED_FACTORS	1.188786918	1.448461225
**FRAS1**	10.15242	8.492037441	4.47E-06	10.15242	7.059688889	0.000828288	ECM_GLYCOPROTEINS	1.195522284	1.438083202
**IBSP**	4.182671111	3.321830985	0.029283794	4.182671111	0.321166667	0.000878345	ECM_GLYCOPROTEINS	1.259146275	13.0233662
**ITIH5**	12.30814	9.233053667	6.37E-21	12.30814	8.220522222	0.000167659	ECM_REGULATORS	1.333051929	1.497245512
**LAMA5**	13.08274222	12.38471859	0.000890017	13.08274222	10.99546667	1.74E-05	ECM_GLYCOPROTEINS	1.056361687	1.189830556
**LAMA5**	13.08274222	12.38471859	0.000890017	13.08274222	10.99546667	1.74E-05	BASEMENT_MEMBRANES	1.056361687	1.189830556
**LAMB1**	12.78626	12.27149268	0.037389473	12.78626	11.20578889	0.000303658	BASEMENT_MEMBRANES	1.041948224	1.141040593
**LAMB1**	12.78626	12.27149268	0.037389473	12.78626	11.20578889	0.000303658	ECM_GLYCOPROTEINS	1.041948224	1.141040593
**LAMB3**	12.18653556	10.88015143	0.002449804	12.18653556	9.040177778	4.28E-05	ECM_GLYCOPROTEINS	1.120070399	1.348041582
**LAMB3**	12.18653556	10.88015143	0.002449804	12.18653556	9.040177778	4.28E-05	BASEMENT_MEMBRANES	1.120070399	1.348041582
**LAMC1**	13.22067778	12.81834145	0.043114543	13.22067778	11.38597778	0.000133286	BASEMENT_MEMBRANES	1.031387549	1.161136798
**LAMC1**	13.22067778	12.81834145	0.043114543	13.22067778	11.38597778	0.000133286	ECM_GLYCOPROTEINS	1.031387549	1.161136798
**LAMC2**	11.83048222	10.30634999	0.007664373	11.83048222	7.253066667	1.91E-05	ECM_GLYCOPROTEINS	1.147882833	1.631100714
**LAMC2**	11.83048222	10.30634999	0.007664373	11.83048222	7.253066667	1.91E-05	BASEMENT_MEMBRANES	1.147882833	1.631100714
**LGALS9**	11.49061556	10.91498941	0.008790636	11.49061556	9.483155556	0.00024324	ECM_AFFILIATED	1.052737215	1.211686921
**LGALS9B**	3.888948889	2.802568376	0.006482108	3.888948889	1.934011111	0.017663085	ECM_AFFILIATED	1.387637469	2.010820345
**LOXL4**	10.50366667	7.762506616	1.36E-15	10.50366667	7.781388889	0.000630521	ECM_REGULATORS	1.353128208	1.349844715
**LTBP3**	12.67204444	12.18913514	0.00840958	12.67204444	11.97125556	0.01599177	ECM_GLYCOPROTEINS	1.039618013	1.058539297
**MMP24**	8.549486667	6.976836893	1.66E-05	8.549486667	5.785855556	0.000824987	ECM_REGULATORS	1.225410139	1.477652974
**MMP7**	10.99070222	8.338187526	1.31E-05	10.99070222	7.114788889	0.000967345	ECM_REGULATORS	1.31811646	1.544768565

**MUC13**	9.166308889	4.625577288	9.80E-12	9.166308889	4.631688889	0.000423468	ECM_AFFILIATED	1.981657276	1.979042442
**MUC20**	10.67625111	9.049174913	3.99E-05	10.67625111	8.639433333	0.004508428	ECM_AFFILIATED	1.179803818	1.235758261
**OTOG**	3.278717778	1.294315707	1.21E-12	3.278717778	0.231422222	0.00099601	ECM_GLYCOPROTEINS	2.533166955	14.16768773
**PLOD3**	12.26494444	11.66115053	0.000132368	12.26494444	11.3402	0.000224254	ECM_REGULATORS	1.051778245	1.081545691
**PLXNB2**	14.42507333	13.68274198	1.50E-08	14.42507333	13.75444444	0.010803833	ECM_AFFILIATED	1.054253113	1.04875725
**PLXNB3**	9.231353333	8.448288315	0.041319563	9.231353333	6.432088889	0.000804851	ECM_AFFILIATED	1.092689192	1.43520301
**REG1A**	5.260693333	2.828283195	0.000155934	5.260693333	1.0297	0.0054383	ECM_AFFILIATED	1.860030616	5.108957301
**SEMA3E**	7.496664444	5.290611356	5.42E-06	7.496664444	5.378366667	0.045244281	ECM_AFFILIATED	1.416975079	1.393855218
**SEMA6A**	11.29825111	9.633962968	2.14E-08	11.29825111	9.276755556	0.001035959	ECM_AFFILIATED	1.172752184	1.217909758
**SFTA2**	4.31276	3.218235206	0.043566529	4.31276	2.391	0.04905761	ECM_AFFILIATED	1.340100932	1.803747386
**SPP1**	14.55278667	12.02522585	5.44E-08	14.55278667	10.32803333	0.001032058	ECM_GLYCOPROTEINS	1.21018822	1.409056903
**TGM3**	5.379013333	3.865587137	0.001262149	5.379013333	2.945666667	0.015938644	ECM_REGULATORS	1.391512633	1.826076723
**THBS3**	10.20764667	9.886424149	0.035712411	10.20764667	8.956644444	0.00043155	ECM_GLYCOPROTEINS	1.032491274	1.139673092
**TIMP1**	14.01019111	13.07373821	0.000304222	14.01019111	12.67466667	0.003658915	ECM_REGULATORS	1.071628549	1.105369591
**TINAG**	3.055817778	1.888654027	0.018339637	3.055817778	0.153922222	0.006443728	ECM_GLYCOPROTEINS	1.617987061	19.85299935
**TINAGL1**	11.26729333	10.6401024	0.041314213	11.26729333	9.955811111	0.002947256	ECM_GLYCOPROTEINS	1.058945948	1.131730324
**VWA7**	8.161304444	6.571677298	5.96E-09	8.161304444	6.430344444	0.000374835	ECM_GLYCOPROTEINS	1.241890628	1.269186202
**VWDE**	5.589968889	4.370217663	0.003702368	5.589968889	3.770844444	0.030437918	ECM_GLYCOPROTEINS	1.279105372	1.482418321
**ZPLD1**	5.786535556	2.26459539	1.68E-29	5.786535556	3.9729	0.041046614	ECM_GLYCOPROTEINS	2.555218288	1.456501688

Colon Adenocarcinoma (COAD)

Signature genes	Expression in tumor	Expression in rest of Pan-Cancer	P-value	Expression in tumor	Expression in normal tissue	P-value	Matrisome category	Fold change vs. Pan-Cancer	Fold change vs. normal
**ACAN**	7.741981269	6.323319026	3.44E-26	7.741981269	4.509321951	7.89E-25	PROTEOGLYCANS	1.224354052	1.716883681
**ADAM19**	10.15683112	8.916404124	8.79E-43	10.15683112	9.618573171	0.002278981	ECM_REGULATORS	1.139117404	1.05596027
**ADAM9**	12.30679577	11.71561116	8.25E-17	12.30679577	11.95276829	0.004866329	ECM_REGULATORS	1.050461269	1.029618869
**ADAMTS12**	8.827316616	7.868232738	2.42E-13	8.827316616	5.957226829	2.96E-20	ECM_REGULATORS	1.121893176	1.481782861
**ADAMTS14**	7.859058912	6.534747141	8.30E-30	7.859058912	6.95734878	1.14E-07	ECM_REGULATORS	1.202656927	1.129605423
**ADAMTS2**	9.875450151	9.391779411	2.74E-05	9.875450151	8.178721951	4.13E-10	ECM_REGULATORS	1.051499372	1.207456398
**ADAMTS6**	5.915548943	5.681847736	0.019120835	5.915548943	4.083021951	6.27E-14	ECM_REGULATORS	1.041131198	1.448816346
**ADAMTSL5**	7.413130514	6.033847598	6.85E-36	7.413130514	6.445768293	1.02E-09	ECM_REGULATORS	1.228590944	1.150077101
**AGT**	9.023667372	8.416494983	0.002879375	9.023667372	7.708885366	9.64E-08	ECM_REGULATORS	1.072140765	1.1705541
**AMELX**	2.045275529	0.194335534	0	2.045275529	0.197573171	1.75E-11	ECM_GLYCOPROTEINS	10.52445475	10.35199021
**ANXA3**	11.30661813	8.926245599	7.35E-57	11.30661813	10.07511463	3.17E-15	ECM_AFFILIATED	1.266671189	1.122232207
**AREG**	10.77660091	6.670819537	1.18E-117	10.77660091	9.27405122	1.58E-07	SECRETED_FACTORS	1.615483802	1.16201654
**BMP4**	10.69677372	7.75824932	2.48E-165	10.69677372	9.416534146	5.06E-10	SECRETED_FACTORS	1.378761274	1.135956558
**BMP7**	8.863184894	7.932301201	1.25E-05	8.863184894	7.089192683	6.20E-06	SECRETED_FACTORS	1.117353548	1.250238961
**C1QTNF1**	10.20027432	9.766051711	6.53E-06	10.20027432	9.558597561	4.01E-05	ECM_AFFILIATED	1.044462452	1.067130848
**C1QTNF8**	0.563191239	0.303833429	2.30E-08	0.563191239	0.147185366	0.007540941	ECM_AFFILIATED	1.853618414	3.826407846
**CCL20**	10.02366163	5.551004688	9.28E-134	10.02366163	9.057821951	0.000198205	SECRETED_FACTORS	1.805738275	1.106630455
**CCL24**	8.513945921	2.305375744	0	8.513945921	7.465878049	0.000451279	SECRETED_FACTORS	3.693083847	1.14038106
**CCL26**	3.72293716	3.004064052	3.40E-11	3.72293716	2.553412195	3.33E-06	SECRETED_FACTORS	1.239300193	1.458024352
**CLEC6A**	1.592246828	1.388828189	0.018370693	1.592246828	0.915414634	0.003456681	ECM_AFFILIATED	1.146467821	1.739372267
**COL10A1**	7.564254985	6.694443984	2.55E-05	7.564254985	1.719041463	5.12E-23	COLLAGENS	1.129930283	4.400274889
**COL11A1**	8.839041994	7.317275999	1.66E-12	8.839041994	3.751768293	4.07E-26	COLLAGENS	1.207968921	2.355966921
**COL12A1**	12.4029423	11.1207018	6.56E-19	12.4029423	10.70771463	1.20E-10	COLLAGENS	1.115302121	1.158318345
**COL1A1**	15.54729154	14.56758602	1.43E-08	15.54729154	13.56163659	7.39E-12	COLLAGENS	1.067252427	1.14641706
**COL1A2**	15.13475861	14.3745589	4.38E-07	15.13475861	14.01686829	9.94E-06	COLLAGENS	1.052885081	1.079753216
**COL3A1**	15.26913142	14.11161066	3.51E-12	15.26913142	14.47818537	0.001215621	COLLAGENS	1.082026126	1.054630193
**COL4A1**	13.36630846	13.10463265	0.01485611	13.36630846	12.08233659	5.96E-11	COLLAGENS	1.019968191	1.106268507
**COL4A1**	13.36630846	13.10463265	0.01485611	13.36630846	12.08233659	5.96E-11	BASEMENT_MEMBRANES	1.019968191	1.106268507
**COL5A1**	12.62603625	11.66527755	2.28E-12	12.62603625	11.35673415	3.47E-08	COLLAGENS	1.082360552	1.111766472
**COL5A2**	12.07467795	11.39125581	4.58E-08	12.07467795	10.70444146	1.09E-08	COLLAGENS	1.059995328	1.12800635
**COL6A3**	13.54584381	12.4598605	1.79E-12	13.54584381	12.94646829	0.016257269	BASEMENT_MEMBRANES	1.087158544	1.046296449
**COL6A3**	13.54584381	12.4598605	1.79E-12	13.54584381	12.94646829	0.016257269	COLLAGENS	1.087158544	1.046296449
**COL9A3**	8.182997281	6.830510682	1.34E-21	8.182997281	6.703219512	7.63E-06	COLLAGENS	1.198006659	1.220756275
**CSF2**	2.841800604	1.948265976	2.95E-13	2.841800604	1.243990244	2.35E-08	SECRETED_FACTORS	1.458630721	2.284423546
**CSF3**	4.576332628	2.809586086	9.58E-29	4.576332628	3.166931707	0.000190332	SECRETED_FACTORS	1.628828051	1.445036727
**CST1**	7.820570393	4.675118846	5.03E-46	7.820570393	1.791290244	5.44E-26	ECM_REGULATORS	1.672806757	4.365886779
**CST4**	2.665591541	2.050409364	1.31E-05	2.665591541	0.15047561	5.80E-14	ECM_REGULATORS	1.300028954	17.71444253
**CSTL1**	1.168087915	0.510782866	5.51E-36	1.168087915	0.203880488	5.99E-07	ECM_REGULATORS	2.286858064	5.729277617
**CTSV**	8.429963142	7.231072045	4.07E-16	8.429963142	7.281407317	6.29E-07	ECM_REGULATORS	1.165797145	1.157738164
**CXCL1**	10.25534773	6.556732015	9.25E-96	10.25534773	7.884412195	8.99E-15	SECRETED_FACTORS	1.564094386	1.300711769
**CXCL11**	7.610099698	6.342739488	2.83E-17	7.610099698	5.055195122	3.00E-11	SECRETED_FACTORS	1.199812749	1.505401773

**CXCL2**	8.599125076	6.828612117	7.13E-36	8.599125076	6.915368293	1.59E-09	SECRETED_FACTORS	1.259278595	1.243480421
**CXCL3**	9.562309063	4.889910098	2.58E-275	9.562309063	7.143853659	6.46E-16	SECRETED_FACTORS	1.955518378	1.338536527
**CXCL5**	6.851458006	4.732781069	2.25E-32	6.851458006	3.568592683	4.19E-10	SECRETED_FACTORS	1.447660034	1.919932762
**CXCL6**	5.31704713	4.336360895	6.48E-08	5.31704713	3.923902439	2.09E-05	SECRETED_FACTORS	1.226154201	1.355040604
**CXCL8**	9.950111178	7.779989041	1.23E-44	9.950111178	6.867073171	1.50E-14	SECRETED_FACTORS	1.2789364	1.448959539
**EPYC**	3.160630514	2.536326265	6.88E-05	3.160630514	0.320021951	1.66E-10	PROTEOGLYCANS	1.246145087	9.876292865
**EREG**	9.240881873	3.905234524	2.70E-182	9.240881873	7.427312195	4.09E-05	SECRETED_FACTORS	2.366280902	1.244175771
**F12**	8.796860121	7.100190636	5.86E-34	8.796860121	7.947385366	5.23E-06	ECM_REGULATORS	1.238961117	1.106887324
**FGF18**	5.608446224	4.782199904	1.25E-10	5.608446224	4.12797561	3.13E-11	SECRETED_FACTORS	1.172775362	1.358643256
**FGF19**	3.81191571	1.643065402	5.40E-51	3.81191571	0.581517073	1.37E-11	SECRETED_FACTORS	2.320002421	6.555122602
**FGF20**	2.132281571	1.844686926	0.005489186	2.132281571	0.345326829	2.80E-06	SECRETED_FACTORS	1.155904311	6.174676829
**FGF23**	0.853424773	0.469638924	3.47E-10	0.853424773	0.18964878	0.006894498	SECRETED_FACTORS	1.817193442	4.500027742
**FGF3**	0.758840181	0.3792103	1.15E-07	0.758840181	0.061429268	0.00362738	SECRETED_FACTORS	2.001106462	12.35307212
**GPC4**	11.06736163	9.843267751	1.43E-22	11.06736163	10.66705854	0.004812882	ECM_AFFILIATED	1.124358487	1.037527036
**HTRA3**	10.08740272	9.118383418	1.46E-12	10.08740272	9.320917073	0.001038068	ECM_REGULATORS	1.106270954	1.082232858
**LAMB1**	12.69793565	12.17469109	3.04E-08	12.69793565	12.31526098	0.001056658	BASEMENT_MEMBRANES	1.042978056	1.031073209
**LAMB1**	12.69793565	12.17469109	3.04E-08	12.69793565	12.31526098	0.001056658	ECM_GLYCOPROTEINS	1.042978056	1.031073209
**LAMC2**	12.4503994	9.831347513	1.14E-32	12.4503994	11.65961463	3.70E-07	ECM_GLYCOPROTEINS	1.266398058	1.067822547
**LAMC2**	12.4503994	9.831347513	1.14E-32	12.4503994	11.65961463	3.70E-07	BASEMENT_MEMBRANES	1.266398058	1.067822547
**LOXL2**	10.62957855	10.07428463	1.70E-08	10.62957855	9.347885366	1.00E-11	ECM_REGULATORS	1.055119936	1.137110494
**MMP1**	9.955311178	6.627750723	6.42E-51	9.955311178	6.787319512	1.64E-13	ECM_REGULATORS	1.502064817	1.466751515
**MMP10**	5.406912387	4.533528019	2.08E-05	5.406912387	3.13967561	2.39E-09	ECM_REGULATORS	1.192650043	1.722124531
**MMP11**	11.20469879	9.858270961	5.55E-17	11.20469879	8.025609756	6.72E-22	ECM_REGULATORS	1.136578497	1.396118069
**MMP12**	9.839807855	5.270708918	2.90E-104	9.839807855	8.746031707	0.000668816	ECM_REGULATORS	1.866885083	1.125059705
**MMP20**	0.908372508	0.592134173	1.57E-06	0.908372508	0.294458537	0.002182214	ECM_REGULATORS	1.534065333	3.084891062
**MMP3**	8.497773414	4.142292602	8.26E-97	8.497773414	4.818139024	8.27E-15	ECM_REGULATORS	2.051466236	1.763704486
**MMP7**	8.937566767	8.135262776	0.000503342	8.937566767	2.8683	4.91E-27	ECM_REGULATORS	1.098620538	3.115980465
**MMP8**	2.568664048	1.926423533	5.31E-07	2.568664048	0.532968293	7.74E-09	ECM_REGULATORS	1.333384899	4.819543833
**MMP9**	10.01092961	9.293933642	8.68E-06	10.01092961	9.1798	0.004315545	ECM_REGULATORS	1.077146663	1.090538967
**MUC5AC**	6.66353716	2.53302771	1.23E-100	6.66353716	4.263578049	5.03E-05	ECM_AFFILIATED	2.63066098	1.562897896
**MXRA5**	11.20178882	10.90338756	0.037003251	11.20178882	10.55420732	0.00922954	ECM_GLYCOPROTEINS	1.027367757	1.061357664
**NID2**	9.39686284	9.115427105	0.009912174	9.39686284	8.794329268	0.000836664	BASEMENT_MEMBRANES	1.030874662	1.068513874
**NID2**	9.39686284	9.115427105	0.009912174	9.39686284	8.794329268	0.000836664	ECM_GLYCOPROTEINS	1.030874662	1.068513874
**OVGP1**	7.185370695	6.287084938	1.20E-21	7.185370695	5.874529268	4.55E-15	ECM_AFFILIATED	1.142877942	1.223139824
**PLOD3**	12.43899607	11.61495715	1.62E-45	12.43899607	11.62421707	6.86E-13	ECM_REGULATORS	1.070946359	1.070093237
**PODNL1**	7.261954985	6.538444441	5.79E-09	7.261954985	6.171687805	5.74E-09	PROTEOGLYCANS	1.110654843	1.176656243
**POSTN**	11.76367221	10.68835551	6.50E-08	11.76367221	11.15212195	0.038804147	ECM_GLYCOPROTEINS	1.100606375	1.05483712
**PRSS1**	2.28724139	1.947603848	0.046862798	2.28724139	0.540290244	2.58E-05	ECM_REGULATORS	1.174387385	4.233356822
**PRSS2**	4.922577644	2.974642102	5.17E-23	4.922577644	2.596470732	9.95E-05	ECM_REGULATORS	1.654847028	1.895872572
**REG1A**	8.510999396	2.150232005	6.82E-211	8.510999396	5.872134146	0.000587104	ECM_AFFILIATED	3.958177247	1.449387767
**REG1B**	5.172576133	0.604387787	3.35e-322	5.172576133	2.999946341	0.001892955	ECM_AFFILIATED	8.558373025	1.724222884
**REG3A**	6.572507553	0.691989679	0	6.572507553	4.018180488	0.0003001	ECM_AFFILIATED	9.497984945	1.635692466
**SDC4**	13.11104139	12.80251828	0.001697108	13.11104139	12.69398537	9.57E-05	ECM_AFFILIATED	1.024098627	1.032854617
**SERPINA4**	3.353470091	2.266563977	2.52E-08	3.353470091	0.267243902	4.51E-15	ECM_REGULATORS	1.479539128	12.54835025
**SERPINA7**	2.670365861	1.114846407	2.51E-23	2.670365861	1.594773171	0.006112327	ECM_REGULATORS	2.395276913	1.674448699
**SERPINB5**	9.648550151	5.535738191	1.08E-51	9.648550151	6.890763415	4.47E-12	ECM_REGULATORS	1.742956372	1.400214979

**SERPINE2**	11.14265921	10.74347755	0.002672357	11.14265921	10.10229756	2.84E-07	ECM_REGULATORS	1.037155722	1.102982678
**SFTA2**	4.350291239	2.87030541	6.62E-14	4.350291239	0.296768293	5.28E-21	ECM_AFFILIATED	1.515619635	14.65888151
**SPON2**	10.79167492	10.24663644	2.91E-05	10.79167492	9.972619512	5.78E-07	ECM_GLYCOPROTEINS	1.053191942	1.082130418
**SRPX2**	8.940777644	7.587607153	1.60E-25	8.940777644	6.808426829	3.25E-13	ECM_GLYCOPROTEINS	1.178339556	1.313192881
**SULF1**	12.01272296	10.89942983	6.75E-16	12.01272296	10.49467317	4.47E-07	ECM_REGULATORS	1.102142328	1.144649554
**TGFBI**	14.18545408	12.17238885	1.43E-71	14.18545408	11.77384634	4.14E-22	ECM_GLYCOPROTEINS	1.165379635	1.204827519
**TSPEAR**	4.051556193	2.483923895	5.12E-41	4.051556193	2.607041463	4.28E-05	ECM_GLYCOPROTEINS	1.631111244	1.554081993
**WISP1**	7.612747432	7.255730357	0.005976733	7.612747432	5.250046341	6.38E-16	ECM_GLYCOPROTEINS	1.049204843	1.450034331
**WNT11**	7.739306647	5.899689413	2.45E-43	7.739306647	6.9725	0.030773984	SECRETED_FACTORS	1.311815946	1.109975855
**WNT2**	7.172414804	5.44718666	1.35E-24	7.172414804	2.466446341	2.63E-32	SECRETED_FACTORS	1.316719116	2.907995476
**WNT8B**	2.009831722	1.719004804	0.000761904	2.009831722	0.780387805	1.30E-07	SECRETED_FACTORS	1.169183307	2.57542687
**VWA2**	8.487925076	5.360233535	5.86E-76	8.487925076	5.181985366	1.33E-24	ECM_GLYCOPROTEINS	1.583499118	1.637967782
**VWA9**	10.72670453	10.61265181	6.57E-06	10.72670453	10.5460878	0.001727008	ECM_GLYCOPROTEINS	1.010746864	1.01712642
**ZP3**	8.696715408	7.783635916	1.42E-28	8.696715408	7.458843902	6.69E-10	ECM_GLYCOPROTEINS	1.11730758	1.165960237

Esophageal Carcinoma (ESCA)

Signature genes	Expression in tumor	Expression in rest of Pan-Cancer	P-value	Expression in tumor	Expression in normal tissue	P-value	Matrisome category	Fold change vs. Pan-Cancer	Fold change vs. normal
**ACAN**	7.643793846	6.323319026	2.99E-14	7.643793846	5.261592308	1.84E-05	PROTEOGLYCANS	1.208826222	1.452752969
**ADAM10**	12.65238103	12.04475783	5.54E-30	12.65238103	12.09913846	0.004309001	ECM_REGULATORS	1.050447107	1.045725782
**ADAM12**	9.459065128	8.421758121	3.79E-08	9.459065128	6.0994	1.84E-08	ECM_REGULATORS	1.123169888	1.550818954
**ADAM17**	11.16374154	10.20838575	6.92E-30	11.16374154	10.15537692	0.000134765	ECM_REGULATORS	1.093585393	1.099293667
**ADAM8**	9.872030769	8.821557749	4.86E-16	9.872030769	8.428230769	0.000716041	ECM_REGULATORS	1.119080218	1.171305229
**ADAMDEC1**	7.070861026	5.850329943	3.14E-10	7.070861026	4.6466	3.98E-05	ECM_REGULATORS	1.208626025	1.521727936
**ADAMTS12**	9.607984615	7.868232738	1.45E-24	9.607984615	6.631392308	2.98E-08	ECM_REGULATORS	1.221110882	1.448863854
**ADAMTS14**	8.415809744	6.534747141	4.92E-35	8.415809744	6.572523077	1.59E-05	ECM_REGULATORS	1.287855454	1.280453434
**ADAMTS2**	10.79261026	9.391779411	8.54E-21	10.79261026	9.175669231	0.000593237	ECM_REGULATORS	1.149154999	1.176220501
**ADAMTS20**	3.727416923	1.790075893	2.38E-30	3.727416923	1.073938462	0.002775868	ECM_REGULATORS	2.082267538	3.470791909
**ADAMTS6**	6.928011282	5.681847736	6.29E-22	6.928011282	5.974030769	0.020367439	ECM_REGULATORS	1.219323643	1.159687914
**ADAMTS7**	9.147463077	8.00658547	3.75E-24	9.147463077	8.211084615	0.003296582	ECM_REGULATORS	1.142492403	1.11403834
**ADAMTSL5**	8.596593333	6.033847598	1.73E-70	8.596593333	7.807423077	0.04897076	ECM_REGULATORS	1.424728284	1.101079479
**AGRN**	14.51676205	13.28611182	1.98E-38	14.51676205	13.42239231	0.000229687	BASEMENT_MEMBRANES	1.092626816	1.081533136
**AGRN**	14.51676205	13.28611182	1.98E-38	14.51676205	13.42239231	0.000229687	ECM_GLYCOPROTEINS	1.092626816	1.081533136
**AMH**	6.30182	4.78537348	4.52E-18	6.30182	5.143623077	0.048902531	SECRETED_FACTORS	1.316891989	1.225171422
**ANGPT2**	9.245981538	8.572867687	7.52E-09	9.245981538	7.494123077	1.76E-07	SECRETED_FACTORS	1.078516767	1.233764304
**ANXA2**	15.50781077	14.42235591	4.19E-22	15.50781077	14.38373846	8.68E-05	ECM_AFFILIATED	1.075261966	1.078148828
**ANXA8**	9.253132308	5.867657451	1.82E-25	9.253132308	5.756746154	0.002675698	ECM_AFFILIATED	1.576972137	1.607354582
**ANXA8L1**	9.189085128	5.816157217	2.97E-32	9.189085128	6.7563	0.01558048	ECM_AFFILIATED	1.579923786	1.36007654
**ARTN**	6.935233333	5.510244494	8.63E-19	6.935233333	5.3514	0.020226771	SECRETED_FACTORS	1.258607189	1.295966165
**ASPN**	9.32468	8.261593676	4.83E-09	9.32468	8.150392308	0.014657761	PROTEOGLYCANS	1.128678118	1.144077444
**BGN**	13.61419897	13.22871855	0.008277135	13.61419897	11.42556154	1.01E-06	PROTEOGLYCANS	1.029139665	1.191556225
**BMP1**	11.37506872	10.62402635	3.19E-20	11.37506872	10.10366154	1.11E-05	ECM_REGULATORS	1.070692819	1.12583628
**BMP8A**	6.619213846	4.035349936	2.96E-87	6.619213846	5.049584615	0.000271714	SECRETED_FACTORS	1.640307272	1.310843238
**BMP8B**	8.798971795	7.691986097	9.82E-19	8.798971795	7.993153846	0.024670619	SECRETED_FACTORS	1.143914157	1.100813517
**CCL18**	8.447629744	6.923515264	4.76E-10	8.447629744	6.628276923	0.005119934	SECRETED_FACTORS	1.220135931	1.274483526
**CCL20**	9.324222051	5.551004688	6.37E-58	9.324222051	5.799869231	5.26E-06	SECRETED_FACTORS	1.679735935	1.607660739
**CCL22**	7.501091282	5.887581409	1.14E-20	7.501091282	5.984853846	0.002273717	SECRETED_FACTORS	1.274053089	1.253345775
**CCL24**	4.503682051	2.305375744	3.21E-48	4.503682051	3.019338462	0.007277726	SECRETED_FACTORS	1.953556622	1.491612189
**CCL26**	4.098959487	3.004064052	8.79E-15	4.098959487	1.924653846	7.50E-05	SECRETED_FACTORS	1.364471402	2.129712569
**CCL7**	2.627906667	2.256527657	0.013414109	2.627906667	0.983615385	0.003675837	SECRETED_FACTORS	1.164579862	2.671681134
**CD109**	11.27835179	9.848226658	2.26E-23	11.27835179	9.809415385	0.012645181	ECM_REGULATORS	1.145216513	1.1497476
**CLEC2B**	8.854177436	8.265322842	3.56E-07	8.854177436	7.797076923	0.034212678	ECM_AFFILIATED	1.071243992	1.135576515
**CLEC6A**	1.674436923	1.388828189	0.010584461	1.674436923	0.691453846	0.019673636	ECM_AFFILIATED	1.205647276	2.421617773
**CLEC7A**	8.254169231	7.78728395	0.000295486	8.254169231	6.634223077	0.000783286	ECM_AFFILIATED	1.059954829	1.244180236
**CNTF**	5.009567179	4.014342028	2.58E-24	5.009567179	4.416807692	0.028729978	SECRETED_FACTORS	1.247917378	1.13420541
**COCH**	7.885620513	6.377181409	2.23E-16	7.885620513	6.044215385	0.00418985	ECM_GLYCOPROTEINS	1.23653696	1.304655776
**COL10A1**	8.241527179	6.694443984	6.84E-09	8.241527179	3.686184615	2.75E-07	COLLAGENS	1.23109958	2.235787959
**COL12A1**	13.29082564	11.1207018	6.52E-31	13.29082564	11.90566154	0.000969624	COLLAGENS	1.195142706	1.116344993
**COL13A1**	7.078155897	6.342576509	1.41E-07	7.078155897	6.377892308	0.022021592	COLLAGENS	1.115974855	1.109795455
**COL18A1**	13.22397231	12.91779311	0.004805203	13.22397231	12.44866923	0.004415298	BASEMENT_MEMBRANES	1.023702129	1.062279997

**COL18A1**	13.22397231	12.91779311	0.004805203	13.22397231	12.44866923	0.004415298	COLLAGENS	1.023702129	1.062279997
**COL1A1**	16.57822103	14.56758602	3.95E-19	16.57822103	14.09875385	4.34E-06	COLLAGENS	1.138021152	1.175864279
**COL1A2**	15.62648359	14.3745589	1.60E-10	15.62648359	14.12480769	0.001849271	COLLAGENS	1.087093086	1.106314785
**COL24A1**	6.323890769	5.296083259	3.58E-12	6.323890769	4.541546154	0.000602171	COLLAGENS	1.194069364	1.392453265
**COL27A1**	11.07643077	9.554815529	1.41E-31	11.07643077	9.812953846	0.000444242	COLLAGENS	1.159251137	1.128756024
**COL3A1**	15.67227692	14.11161066	5.75E-13	15.67227692	14.07846923	0.001244422	COLLAGENS	1.110594481	1.113208877
**COL4A1**	14.2105159	13.10463265	2.59E-15	14.2105159	12.99077692	0.000446812	COLLAGENS	1.084388725	1.093892689
**COL4A1**	14.2105159	13.10463265	2.59E-15	14.2105159	12.99077692	0.000446812	BASEMENT_MEMBRANES	1.084388725	1.093892689
**COL4A2**	14.30296513	13.38834677	1.43E-11	14.30296513	13.54701538	0.024448104	COLLAGENS	1.068314511	1.05580194
**COL4A2**	14.30296513	13.38834677	1.43E-11	14.30296513	13.54701538	0.024448104	BASEMENT_MEMBRANES	1.068314511	1.05580194
**COL5A1**	13.31153949	11.66527755	2.70E-20	13.31153949	11.85597692	0.002323144	COLLAGENS	1.141124969	1.122770361
**COL5A2**	12.83734	11.39125581	5.96E-19	12.83734	11.10731538	9.28E-05	COLLAGENS	1.126946863	1.155755424
**COL5A3**	10.44755179	9.425908801	8.51E-14	10.44755179	8.729923077	8.62E-05	COLLAGENS	1.108386683	1.196751873
**COL6A3**	14.29269231	12.4598605	6.10E-20	14.29269231	13.12082308	0.010579034	COLLAGENS	1.147098903	1.089313698
**COL6A3**	14.29269231	12.4598605	6.10E-20	14.29269231	13.12082308	0.010579034	BASEMENT_MEMBRANES	1.147098903	1.089313698
**COL7A1**	12.11862769	9.209955931	6.14E-49	12.11862769	8.9184	3.21E-07	COLLAGENS	1.315818206	1.358834286
**COL8A1**	10.2832759	9.734282292	0.005138472	10.2832759	9.165130769	0.040399215	COLLAGENS	1.056397954	1.12199991
**CRLF3**	10.00320718	8.988405963	8.58E-57	10.00320718	9.129569231	3.26E-06	SECRETED_FACTORS	1.112901133	1.095693228
**CSF2**	3.875177949	1.948265976	1.74E-33	3.875177949	1.0002	5.76E-06	SECRETED_FACTORS	1.989039483	3.874403068
**CST1**	9.169130769	4.675118846	1.83E-55	9.169130769	3.910076923	2.96E-08	ECM_REGULATORS	1.961261536	2.345000098
**CST2**	4.52867641	3.474976626	2.73E-07	4.52867641	2.618392308	0.001778224	ECM_REGULATORS	1.303224999	1.729563747
**CST4**	2.929877436	2.050409364	1.63E-06	2.929877436	0.6525	0.000609483	ECM_REGULATORS	1.428923164	4.490233618
**CST6**	5.763257436	5.121371418	0.008089193	5.763257436	3.543061538	0.005731374	ECM_REGULATORS	1.125334791	1.626632045
**CSTL1**	1.590729231	0.510782866	4.92E-57	1.590729231	0.480353846	0.002325061	ECM_REGULATORS	3.114296383	3.311578003
**CTHRC1**	10.02414308	9.01501605	7.30E-08	10.02414308	6.8966	2.83E-09	ECM_GLYCOPROTEINS	1.111938461	1.453490572
**CTSA**	13.13848359	12.74033457	2.40E-09	13.13848359	12.61066154	0.015929984	ECM_REGULATORS	1.031251065	1.041855223
**CTSB**	15.56231949	15.24836909	0.000752586	15.56231949	14.597	0.001986708	ECM_REGULATORS	1.020589113	1.066131362
**CTSC**	12.99329077	12.11148266	8.86E-15	12.99329077	11.64567692	5.81E-05	ECM_REGULATORS	1.07280761	1.115717949
**CTSK**	10.85443128	10.28017202	0.000168625	10.85443128	10.0544	0.048746984	ECM_REGULATORS	1.055860861	1.079570266
**CTSV**	9.786259487	7.231072045	3.06E-40	9.786259487	7.323623077	2.83E-06	ECM_REGULATORS	1.353362188	1.336259306
**CXCL1**	10.37594872	6.556732015	2.38E-61	10.37594872	6.772238462	1.49E-07	SECRETED_FACTORS	1.582487845	1.532129853
**CXCL10**	8.794414872	8.345707122	0.023492332	8.794414872	5.888746154	3.01E-05	SECRETED_FACTORS	1.053765097	1.493427402
**CXCL11**	7.359250256	6.342739488	1.66E-07	7.359250256	4.918738462	0.000454182	SECRETED_FACTORS	1.16026368	1.496166205
**CXCL13**	7.768841026	6.191608355	1.56E-10	7.768841026	5.860576923	0.012527552	SECRETED_FACTORS	1.254737151	1.325610282
**CXCL3**	8.128645641	4.889910098	9.14E-83	8.128645641	6.661115385	0.037461614	SECRETED_FACTORS	1.662330284	1.220312991
**CXCL5**	7.430538462	4.732781069	1.99E-31	7.430538462	4.559953846	0.001561149	SECRETED_FACTORS	1.570015252	1.629520542
**CXCL6**	7.748362564	4.336360895	6.49E-47	7.748362564	4.690661538	1.02E-05	SECRETED_FACTORS	1.786835264	1.651869891
**CXCL8**	10.52959385	7.779989041	8.67E-43	10.52959385	6.636138462	7.46E-09	SECRETED_FACTORS	1.353420139	1.586704965
**CXCL9**	9.897547692	8.857239179	1.61E-06	9.897547692	7.860130769	0.003246439	SECRETED_FACTORS	1.11745291	1.259209036
**DMP1**	3.902303077	1.378079634	9.55E-116	3.902303077	1.283884615	9.10E-05	ECM_GLYCOPROTEINS	2.831696355	3.039449987
**DSPP**	3.108010256	0.492041358	0	3.108010256	0.758930769	0.000393852	ECM_GLYCOPROTEINS	6.316563035	4.095248714
**EGFL6**	8.462397436	5.7928	1.80E-32	8.462397436	6.717638462	1.59E-05	SECRETED_FACTORS	1.460847507	1.259728026
**ELFN1**	7.575115385	6.692110066	1.48E-10	7.575115385	6.558007692	0.007356338	ECM_AFFILIATED	1.13194722	1.155094007
**EREG**	8.442176923	3.905234524	2.47E-81	8.442176923	6.499692308	0.021713566	SECRETED_FACTORS	2.161759267	1.298857934
**F12**	8.602102564	7.100190636	1.31E-16	8.602102564	5.777684615	7.06E-10	ECM_REGULATORS	1.211531212	1.48884945
**FGF11**	8.306902051	7.804446418	0.001729826	8.306902051	6.633007692	0.004541305	SECRETED_FACTORS	1.064380688	1.252358272

**FGF19**	4.355058462	1.643065402	3.14E-47	4.355058462	1.608546154	0.009144709	SECRETED_FACTORS	2.650569148	2.70745011
**FNDC1**	9.045342564	8.021472757	5.97E-06	9.045342564	6.787546154	0.000427981	ECM_GLYCOPROTEINS	1.127641125	1.332638093
**GPC1**	12.90311949	11.63795591	3.29E-29	12.90311949	11.77865385	0.031875166	ECM_AFFILIATED	1.108710119	1.095466397
**HAPLN3**	8.90456359	8.519183557	0.003439171	8.90456359	7.391823077	9.90E-06	PROTEOGLYCANS	1.045236733	1.20465053
**HPSE**	9.290810769	7.671407813	1.75E-47	9.290810769	7.892838462	2.00E-05	ECM_REGULATORS	1.211095929	1.177119083
**HTRA3**	10.63171897	9.118383418	1.97E-17	10.63171897	9.672338462	0.033686128	ECM_REGULATORS	1.165965335	1.099188063
**IBSP**	4.902907179	3.204938584	8.29E-19	4.902907179	0.768992308	6.26E-10	ECM_GLYCOPROTEINS	1.529797545	6.375755818
**ITIH6**	4.151086154	2.729944452	3.62E-11	4.151086154	1.619492308	1.15E-05	ECM_REGULATORS	1.520575318	2.563202143
**LAMA3**	12.86893538	9.61444257	3.57E-63	12.86893538	11.86246923	0.033773012	BASEMENT_MEMBRANES	1.338500417	1.084844574
**LAMA3**	12.86893538	9.61444257	3.57E-63	12.86893538	11.86246923	0.033773012	ECM_GLYCOPROTEINS	1.338500417	1.084844574
**LAMA5**	14.04234051	12.30493433	7.92E-66	14.04234051	13.42252308	0.02035747	ECM_GLYCOPROTEINS	1.141195892	1.046177416
**LAMA5**	14.04234051	12.30493433	7.92E-66	14.04234051	13.42252308	0.02035747	BASEMENT_MEMBRANES	1.141195892	1.046177416
**LAMB3**	13.83676308	10.59206818	4.34E-54	13.83676308	11.90557692	0.000136215	ECM_GLYCOPROTEINS	1.306332517	1.162208532
**LAMB3**	13.83676308	10.59206818	4.34E-54	13.83676308	11.90557692	0.000136215	BASEMENT_MEMBRANES	1.306332517	1.162208532
**LAMC2**	13.7035041	9.831347513	2.12E-41	13.7035041	11.45524615	1.74E-05	ECM_GLYCOPROTEINS	1.393858175	1.196264482
**LAMC2**	13.7035041	9.831347513	2.12E-41	13.7035041	11.45524615	1.74E-05	BASEMENT_MEMBRANES	1.393858175	1.196264482
**LAMC3**	8.370059487	7.867710874	0.00082982	8.370059487	7.141546154	0.008564238	BASEMENT_MEMBRANES	1.063849399	1.17202344
**LAMC3**	8.370059487	7.867710874	0.00082982	8.370059487	7.141546154	0.008564238	ECM_GLYCOPROTEINS	1.063849399	1.17202344
**LGALS7**	6.538820513	2.682360013	5.61E-59	6.538820513	3.982892308	0.030853475	ECM_AFFILIATED	2.437711747	1.641726667
**LGALS8**	11.95496564	11.28158572	5.82E-25	11.95496564	11.44238462	0.005836635	ECM_AFFILIATED	1.05968841	1.044796696
**LOX**	10.65215179	9.477059811	4.20E-14	10.65215179	9.4727	0.006010146	ECM_REGULATORS	1.123993307	1.124510625
**LOXL2**	11.20392256	10.07428463	1.25E-18	11.20392256	9.537892308	2.00E-05	ECM_REGULATORS	1.112130834	1.174674887
**LUM**	13.26252718	11.42934962	1.70E-15	13.26252718	12.04977692	0.005189877	PROTEOGLYCANS	1.160392115	1.100645038
**MFAP2**	9.897047692	9.073903157	4.66E-05	9.897047692	7.241015385	2.79E-07	ECM_GLYCOPROTEINS	1.090715596	1.366803848
**MMP1**	11.52730256	6.627750723	9.65E-65	11.52730256	7.633846154	2.28E-07	ECM_REGULATORS	1.739248057	1.510025527
**MMP10**	7.774604615	4.533528019	1.12E-33	7.774604615	3.959538462	1.76E-06	ECM_REGULATORS	1.714912665	1.963512841
**MMP11**	11.27450103	9.858270961	1.25E-11	11.27450103	7.165853846	1.81E-09	ECM_REGULATORS	1.143659073	1.573364636
**MMP12**	9.635189744	5.270708918	1.28E-57	9.635189744	5.363907692	9.68E-10	ECM_REGULATORS	1.828063339	1.796300439
**MMP13**	6.751493846	4.357289753	1.04E-15	6.751493846	2.191946154	7.79E-06	ECM_REGULATORS	1.549470939	3.080136724
**MMP14**	13.57414615	12.90987096	2.51E-07	13.57414615	12.15228462	3.67E-06	ECM_REGULATORS	1.051454828	1.11700364
**MMP20**	1.108764615	0.592134173	1.54E-09	1.108764615	0.253861538	0.038620348	ECM_REGULATORS	1.872488814	4.367595903
**MMP25**	7.002941026	6.268618718	1.43E-11	7.002941026	6.280161538	0.037060066	ECM_REGULATORS	1.117142602	1.115089315
**MMP3**	9.10476359	4.142292602	1.66E-75	9.10476359	4.870730769	1.40E-07	ECM_REGULATORS	2.198001074	1.869280817
**MMP7**	9.356091795	8.135262776	4.46E-05	9.356091795	6.355438462	0.001330112	ECM_REGULATORS	1.150066329	1.472139468
**MMP8**	2.66470359	1.926423533	8.29E-06	2.66470359	0.760753846	0.001406696	ECM_REGULATORS	1.383238703	3.502714581
**MMP9**	9.930635385	9.293933642	0.002397483	9.930635385	7.024846154	2.28E-06	ECM_REGULATORS	1.06850724	1.413644536
**MUC16**	6.913934359	5.384185087	9.85E-07	6.913934359	3.801884615	0.001467491	ECM_AFFILIATED	1.284118998	1.818554496
**MUCL1**	3.480963077	2.692369792	0.001980121	3.480963077	0.808192308	0.001656486	ECM_AFFILIATED	1.292899322	4.307097511
**NID2**	10.12509231	9.115427105	1.32E-12	10.12509231	8.838846154	0.00323724	ECM_GLYCOPROTEINS	1.110764443	1.145521953
**NID2**	10.12509231	9.115427105	1.32E-12	10.12509231	8.838846154	0.00323724	BASEMENT_MEMBRANES	1.110764443	1.145521953
**OGFOD1**	10.75158615	10.45417434	2.50E-16	10.75158615	10.34435385	0.009790388	ECM_REGULATORS	1.028449096	1.039367593
**PI3**	11.93877795	5.438570844	1.08E-90	11.93877795	8.346023077	0.000658415	ECM_REGULATORS	2.195205007	1.43047507
**PLAU**	11.83188205	10.53550347	1.49E-16	11.83188205	9.151892308	2.57E-08	ECM_REGULATORS	1.123048565	1.292834493
**PLOD3**	12.04825077	11.61495715	1.03E-08	12.04825077	11.00083077	0.000509551	ECM_REGULATORS	1.037304797	1.095212809
**PLXDC1**	9.320161026	8.810221397	9.69E-07	9.320161026	8.401207692	0.003938519	ECM_AFFILIATED	1.057880456	1.10938348
**PLXNA1**	13.12843641	11.47403255	1.33E-69	13.12843641	12.34681538	0.012731139	ECM_AFFILIATED	1.144186785	1.063305476

**PLXNA3**	11.76699641	10.69586156	4.89E-41	11.76699641	11.09333846	0.009156754	ECM_AFFILIATED	1.100144793	1.06072635
**PODNL1**	7.979619487	6.538444441	6.45E-19	7.979619487	6.157630769	0.000119323	PROTEOGLYCANS	1.220415583	1.295891194
**PXDN**	11.84663179	11.25497626	1.10E-05	11.84663179	10.61263077	0.002393483	ECM_GLYCOPROTEINS	1.05256835	1.116276638
**SDC1**	14.2739241	12.36422045	6.46E-23	14.2739241	12.47496154	0.000207311	ECM_AFFILIATED	1.154454028	1.144205861
**SEMA4B**	13.53802	11.79546499	1.81E-78	13.53802	12.76428462	0.012051994	ECM_AFFILIATED	1.14773093	1.060617215
**SERPINB2**	8.234593846	3.854974139	2.55E-59	8.234593846	5.588176923	0.015719074	ECM_REGULATORS	2.136095743	1.473574291
**SERPINB3**	9.101648205	3.364342102	2.51E-66	9.101648205	6.041546154	0.024003919	ECM_REGULATORS	2.705327796	1.506509753
**SERPINB4**	7.289621538	2.728945079	4.75E-58	7.289621538	4.201607692	0.01293812	ECM_REGULATORS	2.671223249	1.734960061
**SERPINB5**	12.49761179	5.535738191	1.54E-85	12.49761179	8.564661538	7.67E-07	ECM_REGULATORS	2.257623349	1.459206734
**SERPINB7**	6.581345641	3.093882334	2.81E-51	6.581345641	4.2684	0.008356575	ECM_REGULATORS	2.127212651	1.541876497
**SERPINB9**	10.24224923	9.199020844	2.92E-27	10.24224923	8.871261538	0.000235061	ECM_REGULATORS	1.11340646	1.154542585
**SERPINE1**	12.31690205	10.74768977	1.22E-21	12.31690205	10.78537692	0.005734948	ECM_REGULATORS	1.146004612	1.142000149
**SERPINH1**	13.038	12.68452609	0.000590143	13.038	11.48388462	2.73E-06	ECM_REGULATORS	1.027866544	1.135330111
**SPON2**	10.69802564	10.24663644	0.007812454	10.69802564	9.478461538	0.000222126	ECM_GLYCOPROTEINS	1.044052427	1.128666883
**ST14**	13.42460256	11.48217639	2.02E-25	13.42460256	12.26475385	0.002816678	ECM_REGULATORS	1.169168815	1.094567631
**SULF1**	11.96557436	10.89942983	2.41E-09	11.96557436	10.37616154	0.001472433	ECM_REGULATORS	1.097816542	1.153179267
**SULF2**	13.03585846	11.71216418	1.86E-27	13.03585846	11.6123	0.0001348	ECM_REGULATORS	1.113018761	1.122590569
**TGFBI**	12.89621333	12.17238885	6.24E-07	12.89621333	11.17114615	0.000567278	ECM_GLYCOPROTEINS	1.059464456	1.154421682
**THBS2**	11.80440821	11.11429101	3.80E-05	11.80440821	10.50054615	0.013137334	ECM_GLYCOPROTEINS	1.062092777	1.12417088
**TINAG**	2.590997949	1.575004602	8.04E-06	2.590997949	0.832123077	0.022305541	ECM_GLYCOPROTEINS	1.645073255	3.113719617
**TNC**	13.37205436	11.82924378	1.32E-18	13.37205436	11.92383846	0.030670438	ECM_GLYCOPROTEINS	1.130423432	1.121455511
**TNFAIP6**	7.574409744	6.456752307	1.84E-09	7.574409744	6.102261538	0.003454241	ECM_GLYCOPROTEINS	1.173099011	1.241246331
**VCAN**	12.25474154	11.6322721	0.000343256	12.25474154	11.09766923	0.019360246	PROTEOGLYCANS	1.053512283	1.104262641
**WISP1**	8.068133846	7.255730357	1.45E-06	8.068133846	5.837430769	1.85E-05	ECM_GLYCOPROTEINS	1.111967155	1.382137821
**WNT10A**	8.032941538	5.868206431	1.04E-26	8.032941538	5.917769231	0.001659691	SECRETED_FACTORS	1.368892119	1.357427305
**WNT11**	6.676541538	5.899689413	6.30E-06	6.676541538	5.137553846	0.025053523	SECRETED_FACTORS	1.131676783	1.299556508
**WNT2**	6.58204	5.44718666	1.93E-07	6.58204	3.668392308	5.48E-07	SECRETED_FACTORS	1.208337516	1.794257388
**WNT3A**	4.873461538	2.892981229	2.60E-24	4.873461538	2.627853846	0.012568961	SECRETED_FACTORS	1.684581113	1.854540558
**WNT5A**	11.21177846	8.979624458	5.32E-40	11.21177846	9.713746154	0.001288968	SECRETED_FACTORS	1.248579884	1.154217774
**WNT7B**	9.563474872	7.379284375	1.23E-16	9.563474872	5.403084615	1.32E-05	SECRETED_FACTORS	1.295989473	1.770002795
**VWA9**	10.82967846	10.61265181	5.09E-11	10.82967846	10.46946923	0.015702004	ECM_GLYCOPROTEINS	1.020449804	1.034405682
**XCL1**	5.096330256	4.158361692	1.22E-10	5.096330256	3.825692308	0.01848279	SECRETED_FACTORS	1.225562044	1.332132813
**ZPLD1**	2.808174359	2.32646436	0.00187849	2.808174359	1.324253846	0.005266199	ECM_GLYCOPROTEINS	1.207056685	2.120571042

Glioblastoma Multiforme (GBM)

Signature genes	Expression in tumor	Expression in rest of Pan-Cancer	P-value	Expression in tumor	Expression in normal tissue	P-value	Matrisome category	Fold change vs. Pan-Cancer	Fold change vs. normal
**A2M**	14.92080409	14.08456856	3.08E-09	14.92080409	13.3281	0.000210522	ECM_REGULATORS	1.059372464	1.119499711
**ADAM12**	9.689019298	8.54835507	7.31E-09	9.689019298	7.1656	0.000100881	ECM_REGULATORS	1.133436693	1.352157432
**ADAM17**	10.76146491	10.17811342	4.41E-11	10.76146491	9.79774	2.56E-05	ECM_REGULATORS	1.057314305	1.09836196
**ADAM9**	12.52838187	11.81519477	1.81E-12	12.52838187	10.89746	1.90E-05	ECM_REGULATORS	1.060361857	1.149660735
**ADAMTS15**	8.277893567	7.764800786	0.005238777	8.277893567	6.15078	0.001365331	ECM_REGULATORS	1.066079323	1.345828264
**ADAMTS6**	7.47978538	5.613373369	1.89E-45	7.47978538	5.10728	1.52E-06	ECM_REGULATORS	1.33249383	1.464534034
**ADAMTS9**	10.04706784	8.96845603	2.24E-14	10.04706784	8.12938	0.00019715	ECM_REGULATORS	1.120267279	1.235895952
**AEBP1**	13.40413743	12.89517578	0.00057708	13.40413743	11.05854	0.000426762	ECM_GLYCOPROTEINS	1.039469152	1.212107333
**ANGPT2**	10.3335848	8.578557541	1.76E-47	10.3335848	6.56438	2.19E-10	SECRETED_FACTORS	1.204583025	1.574190525
**ANGPTL2**	11.48728538	10.30002002	3.45E-19	11.48728538	9.82668	0.000125598	SECRETED_FACTORS	1.115268257	1.168989463
**ANXA5**	14.29945439	13.38816387	3.62E-30	14.29945439	11.6682	1.81E-10	ECM_AFFILIATED	1.068066878	1.225506452
**C1QA**	12.78696901	11.44298469	7.51E-23	12.78696901	10.60316	0.000139674	ECM_AFFILIATED	1.117450503	1.205958319
**C1QB**	13.47323216	11.57063529	4.30E-40	13.47323216	11.26478	0.000105875	ECM_AFFILIATED	1.164433225	1.196049294
**C1QC**	13.22345439	11.40477196	1.57E-39	13.22345439	11.12644	0.00012245	ECM_AFFILIATED	1.159466795	1.18847128
**C1QL1**	11.10762456	5.470629542	1.55E-162	11.10762456	9.54586	0.04693223	ECM_AFFILIATED	2.030410664	1.163606481
**C1QTNF1**	10.78645556	9.803191698	1.14E-13	10.78645556	8.39614	9.91E-05	ECM_AFFILIATED	1.10030038	1.284692199
**C1QTNF5**	10.39453275	9.224824415	3.19E-21	10.39453275	8.31122	4.26E-06	ECM_AFFILIATED	1.126800065	1.250662688
**CCL25**	2.8051	2.245968906	0.006003885	2.8051	1.1001	0.007298855	SECRETED_FACTORS	1.248948724	2.549859104
**CELA1**	1.973848538	0.601802042	9.74E-74	1.973848538	0.3527	0.011197702	ECM_REGULATORS	3.279896709	5.596395061
**CLEC18B**	8.844843275	4.291425886	4.03E-107	8.844843275	6.60962	0.000650599	ECM_AFFILIATED	2.061049989	1.338177274
**CLEC18C**	5.896684795	3.336971755	6.26E-35	5.896684795	3.45574	0.0005729	ECM_AFFILIATED	1.767076627	1.706345036
**CLEC1A**	6.488012281	6.256580537	0.043885321	6.488012281	5.20304	0.001325695	ECM_AFFILIATED	1.036990133	1.246965674
**CLEC4A**	6.691049123	6.154100306	5.67E-08	6.691049123	5.20506	0.001450987	ECM_AFFILIATED	1.087250579	1.285489336
**CLEC4E**	5.952890058	5.397074257	0.000208283	5.952890058	2.30614	6.31E-06	ECM_AFFILIATED	1.10298465	2.581322061
**CLEC5A**	8.526384211	6.282516645	6.57E-43	8.526384211	5.01384	1.02E-05	ECM_AFFILIATED	1.357160624	1.700569665
**COL22A1**	8.85425731	6.480732707	9.98E-29	8.85425731	6.35596	0.002023541	COLLAGENS	1.366243249	1.393063724
**COL28A1**	8.27685731	5.407354682	1.81E-58	8.27685731	6.06172	0.016007492	COLLAGENS	1.530666619	1.36543049
**COL4A6**	6.94462807	6.147784938	0.000812358	6.94462807	4.95066	0.013248676	BASEMENT_MEMBRANES	1.129614673	1.40276813
**COL4A6**	6.94462807	6.147784938	0.000812358	6.94462807	4.95066	0.013248676	COLLAGENS	1.129614673	1.40276813
**CRISPLD1**	11.75843275	7.920171806	1.97E-108	11.75843275	8.618	1.43E-08	ECM_GLYCOPROTEINS	1.484618394	1.364403893
**CSF1**	11.4924	10.52971149	9.14E-23	11.4924	10.41772	0.01020349	SECRETED_FACTORS	1.091425915	1.103158849
**CSPG4**	12.00906433	10.12697844	3.26E-34	12.00906433	10.75842	0.011178378	ECM_AFFILIATED	1.18584871	1.116247955
**CTSL**	12.24415673	11.89488844	0.000350999	12.24415673	11.04692	0.001114008	ECM_REGULATORS	1.029362889	1.108377423
**CTSO**	10.88078129	10.41942741	7.66E-08	10.88078129	9.74212	0.000409833	ECM_REGULATORS	1.044278237	1.116880236
**DMP1**	2.393780702	1.437188155	3.40E-15	2.393780702	1.19868	0.026714204	ECM_GLYCOPROTEINS	1.665600077	1.997013967
**ECM2**	9.647707018	8.074004319	7.23E-31	9.647707018	7.4039	2.99E-05	ECM_GLYCOPROTEINS	1.194909816	1.303057445
**EFEMP1**	12.94942398	10.3545701	5.10E-45	12.94942398	10.28012	9.68E-05	ECM_GLYCOPROTEINS	1.250599866	1.259656889
**EFEMP2**	11.61823977	10.48730135	2.81E-23	11.61823977	9.78854	8.52E-05	ECM_GLYCOPROTEINS	1.107838841	1.186922643
**ELN**	12.09867135	9.960839426	1.91E-33	12.09867135	9.6098	4.18E-06	ECM_GLYCOPROTEINS	1.214623671	1.258993043
**EMILIN1**	11.02497251	10.71583015	0.03241808	11.02497251	9.33006	0.001857221	ECM_GLYCOPROTEINS	1.028849128	1.181661481
**EMILIN3**	9.496962573	4.774837108	2.76E-135	9.496962573	7.66324	0.047526473	ECM_GLYCOPROTEINS	1.988960536	1.239288157

**EPO**	2.917418129	2.129279741	8.99E-07	2.917418129	1.25194	0.00992709	SECRETED_FACTORS	1.370143186	2.33031785
**F13A1**	10.21198889	9.303279016	1.82E-08	10.21198889	7.60208	0.023026719	ECM_REGULATORS	1.0976763	1.343315104
**FAM20C**	12.3022807	10.64280416	6.96E-56	12.3022807	10.94456	0.008545014	ECM_REGULATORS	1.155924747	1.124054389
**FGF11**	10.07708421	7.485597978	9.50E-56	10.07708421	8.68298	0.001727878	SECRETED_FACTORS	1.346196288	1.160555962
**FREM2**	9.408122807	6.572103656	5.81E-26	9.408122807	7.61154	0.009432587	ECM_AFFILIATED	1.431523801	1.236034075
**GPC2**	8.460523977	5.339490218	3.68E-94	8.460523977	6.59618	0.009484983	ECM_AFFILIATED	1.584519052	1.282639949
**HPSE**	8.182471345	7.779119534	0.000808658	8.182471345	6.72414	0.005431225	ECM_REGULATORS	1.051850574	1.216879979
**HYAL2**	11.41889766	11.18846337	0.000155815	11.41889766	9.34486	3.84E-12	ECM_REGULATORS	1.020595705	1.22194422
**IBSP**	7.619619298	3.261130655	5.10E-104	7.619619298	3.27828	0.000281774	ECM_GLYCOPROTEINS	2.336496174	2.32427349
**IGFBP1**	5.678107018	2.828676024	2.24E-27	5.678107018	3.26974	0.000565389	ECM_GLYCOPROTEINS	2.007337344	1.73656224
**IGFBP2**	13.51584327	11.64802408	6.91E-26	13.51584327	8.24424	1.07E-11	ECM_GLYCOPROTEINS	1.160355025	1.639428653
**IGFBP5**	14.81711287	13.33117424	1.14E-16	14.81711287	13.27962	0.006107684	ECM_GLYCOPROTEINS	1.111463447	1.115778378
**IGFBP7**	14.40253801	13.40811877	1.93E-23	14.40253801	11.66528	8.51E-08	ECM_GLYCOPROTEINS	1.074165456	1.234650005
**LAMA2**	10.24906608	8.543781701	1.17E-25	10.24906608	9.1419	0.024603957	ECM_GLYCOPROTEINS	1.199593627	1.121108969
**LAMA2**	10.24906608	8.543781701	1.17E-25	10.24906608	9.1419	0.024603957	BASEMENT_MEMBRANES	1.199593627	1.121108969
**LAMA4**	11.26409357	10.94833087	0.019152065	11.26409357	9.20852	1.66E-05	ECM_GLYCOPROTEINS	1.028841172	1.223225184
**LAMA4**	11.26409357	10.94833087	0.019152065	11.26409357	9.20852	1.66E-05	BASEMENT_MEMBRANES	1.028841172	1.223225184
**LAMB4**	4.150412281	3.022570295	5.27E-16	4.150412281	1.17232	4.33E-08	ECM_GLYCOPROTEINS	1.373140035	3.540340761
**LAMB4**	4.150412281	3.022570295	5.27E-16	4.150412281	1.17232	4.33E-08	BASEMENT_MEMBRANES	1.373140035	3.540340761
**LAMC3**	9.413076608	7.734006658	3.29E-25	9.413076608	8.37412	0.023318582	ECM_GLYCOPROTEINS	1.217102212	1.124067557
**LAMC3**	9.413076608	7.734006658	3.29E-25	9.413076608	8.37412	0.023318582	BASEMENT_MEMBRANES	1.217102212	1.124067557
**LGALS1**	13.43515614	12.75693088	2.57E-10	13.43515614	11.47842	4.48E-05	ECM_AFFILIATED	1.053165238	1.170470861
**LOX**	10.00684795	9.666983029	0.030015082	10.00684795	6.5775	2.55E-05	ECM_REGULATORS	1.03515729	1.521375592
**LOXL3**	10.0967117	7.745675268	1.19E-97	10.0967117	7.88156	1.58E-11	ECM_REGULATORS	1.303528917	1.281054981
**MASP1**	10.83901813	6.007358593	3.27E-79	10.83901813	8.93504	0.007579586	ECM_REGULATORS	1.804290182	1.21309117
**MATN2**	11.78138187	10.21987741	1.22E-28	11.78138187	10.16568	0.000357027	ECM_GLYCOPROTEINS	1.152790919	1.15893692
**MATN3**	7.555430409	6.422352037	8.13E-11	7.555430409	6.55734	0.04471118	ECM_GLYCOPROTEINS	1.176427322	1.152209647
**MFAP3**	8.959746784	8.775480752	0.035689467	8.959746784	8.42072	0.045404131	ECM_GLYCOPROTEINS	1.020997828	1.064011959
**MMP25**	7.214401754	6.312897651	9.34E-15	7.214401754	4.7201	4.13E-05	ECM_REGULATORS	1.142803535	1.528442566
**MMRN1**	7.664406433	6.709784938	8.02E-08	7.664406433	5.85858	0.000337644	ECM_GLYCOPROTEINS	1.142273039	1.308236199
**NGLY1**	10.09700409	9.985555438	0.014202023	10.09700409	9.4496	4.04E-05	ECM_REGULATORS	1.011160987	1.06851127
**NID1**	11.75846433	11.42389893	0.005053943	11.75846433	8.65532	2.36E-09	BASEMENT_MEMBRANES	1.029286446	1.358524506
**NID1**	11.75846433	11.42389893	0.005053943	11.75846433	8.65532	2.36E-09	ECM_GLYCOPROTEINS	1.029286446	1.358524506
**NTN1**	11.32829766	8.219197304	2.15E-69	11.32829766	9.07214	6.07E-06	ECM_GLYCOPROTEINS	1.378272992	1.24869079
**NTN1**	11.32829766	8.219197304	2.15E-69	11.32829766	9.07214	6.07E-06	BASEMENT_MEMBRANES	1.378272992	1.24869079
**PLAT**	11.39158947	10.87718591	0.001089954	11.39158947	9.59414	0.000458037	ECM_REGULATORS	1.047291971	1.187348681
**PLOD2**	11.57145146	11.20386516	0.008848531	11.57145146	9.72598	4.70E-05	ECM_REGULATORS	1.032808883	1.189746582
**PLXNA3**	11.11822047	10.71939606	3.41E-06	11.11822047	10.17876	0.007993257	ECM_AFFILIATED	1.037205866	1.092296161
**PXDNL**	6.287045614	4.518930379	8.02E-29	6.287045614	4.24882	0.004668019	ECM_GLYCOPROTEINS	1.391268527	1.479715689
**SERPINA3**	14.45566374	9.066903829	3.10E-58	14.45566374	10.92132	7.31E-06	ECM_REGULATORS	1.594332974	1.323618733
**SERPINA5**	7.74141462	6.563903441	8.94E-05	7.74141462	4.65622	9.07E-05	ECM_REGULATORS	1.17939191	1.662596402
**SERPINB6**	12.22094795	11.89663158	4.42E-06	12.22094795	10.95826	4.51E-06	ECM_REGULATORS	1.027261193	1.115227048
**SERPINB8**	9.339094152	8.817767763	2.41E-08	9.339094152	8.17354	0.000325245	ECM_REGULATORS	1.059122264	1.142600899
**SERPINE1**	11.93378596	10.86046701	1.82E-10	11.93378596	8.37998	3.27E-05	ECM_REGULATORS	1.098828067	1.42408287
**SPARC**	16.74061637	15.03396626	8.73E-45	16.74061637	13.82872	1.18E-12	ECM_GLYCOPROTEINS	1.113519618	1.210568756
**SPP1**	16.22759181	11.86787706	1.85E-72	16.22759181	13.77804	0.001201029	ECM_GLYCOPROTEINS	1.367354223	1.177786667

**SRGN**	11.83501754	10.95284832	2.97E-14	11.83501754	9.99404	0.000992635	PROTEOGLYCANS	1.080542449	1.184207542
**SRPX**	11.16343392	8.003414041	1.91E-74	11.16343392	8.07598	1.18E-05	ECM_GLYCOPROTEINS	1.394833987	1.382300838
**SRPX2**	9.372615205	7.769879659	3.15E-20	9.372615205	4.73826	1.76E-08	ECM_GLYCOPROTEINS	1.206275466	1.978071107
**TGM5**	4.991847953	2.902514786	2.84E-24	4.991847953	1.55432	0.00024444	ECM_REGULATORS	1.719835495	3.211596038
**TIMP4**	11.04222105	4.989430644	1.98E-260	11.04222105	7.32874	3.44E-07	ECM_REGULATORS	2.213122466	1.506701159
**TNC**	14.00795497	11.78978764	7.62E-33	14.00795497	10.10318	7.00E-10	ECM_GLYCOPROTEINS	1.188143111	1.386489696
**TNFAIP6**	8.519797661	6.560521832	6.10E-24	8.519797661	6.29632	0.001107522	ECM_GLYCOPROTEINS	1.298646339	1.35313924
**VCAN**	12.93575263	11.54805434	1.86E-14	12.93575263	11.1978	1.04E-05	PROTEOGLYCANS	1.120167281	1.155204829
**WISP1**	7.917148538	7.472480874	0.009447198	7.917148538	5.7609	0.019244273	ECM_GLYCOPROTEINS	1.059507367	1.374290222
**WNT5A**	9.941411111	9.054258664	7.64E-07	9.941411111	8.03454	2.70E-05	SECRETED_FACTORS	1.097981787	1.237334198
**VWF**	12.51642164	12.22619669	0.015786384	12.51642164	11.3773	0.00838525	ECM_GLYCOPROTEINS	1.023737958	1.100122317

Head And Neck Squamous Cell Carcinoma (HNSC)

Signature genes	Expression in tumor	Expression in rest of Pan-Cancer	P-value	Expression in tumor	Expression in normal tissue	P-value	Matrisome category	Fold change vs. Pan-Cancer	Fold change vs. normal
**ADAM12**	9.948067376	8.350742022	1.05E-45	9.948067376	6.419404545	2.34E-28	ECM_REGULATORS	1.191279452	1.549686938
**ADAM17**	10.95340993	10.14128815	5.93E-62	10.95340993	10.377175	0.000453842	ECM_REGULATORS	1.080080732	1.055529075
**ADAM19**	9.921342199	8.982960699	1.25E-40	9.921342199	8.620761364	8.28E-09	ECM_REGULATORS	1.104462385	1.150866122
**ADAM23**	8.840080319	6.703816141	5.92E-74	8.840080319	7.994961364	0.012746523	ECM_REGULATORS	1.318663897	1.105706446
**ADAM8**	10.0261734	8.674758783	7.23E-74	10.0261734	8.335984091	1.59E-16	ECM_REGULATORS	1.155786997	1.202758222
**ADAM9**	12.10201826	11.80565268	1.84E-07	12.10201826	11.72331364	0.004994061	ECM_REGULATORS	1.025103702	1.032303548
**ADAMDEC1**	6.715496277	6.075688093	1.18E-07	6.715496277	4.908579545	3.64E-07	ECM_REGULATORS	1.105306292	1.368113976
**ADAMTS12**	8.898492199	8.012440085	4.44E-18	8.898492199	5.717672727	7.89E-24	ECM_REGULATORS	1.110584554	1.556313665
**ADAMTS14**	7.776204965	6.612327525	9.53E-37	7.776204965	5.892034091	1.66E-16	ECM_REGULATORS	1.176016302	1.319782752
**ADAMTS15**	8.207466489	7.84373793	0.000346353	8.207466489	7.277186364	0.003149876	ECM_REGULATORS	1.04637184	1.127835138
**ADAMTS2**	10.75396879	9.443182303	2.43E-48	10.75396879	7.706502273	9.48E-25	ECM_REGULATORS	1.138807708	1.395440942
**ADAMTS20**	3.076375	1.652162821	1.78E-46	3.076375	0.291618182	1.48E-12	ECM_REGULATORS	1.862028948	10.54932508
**ADAMTS4**	9.474466135	9.181597333	0.000378262	9.474466135	8.540547727	3.66E-05	ECM_REGULATORS	1.031897369	1.109351114
**AGRN**	13.96252376	13.21509687	1.69E-39	13.96252376	12.43534091	1.06E-22	ECM_GLYCOPROTEINS	1.056558563	1.12280989
**AGRN**	13.96252376	13.21509687	1.69E-39	13.96252376	12.43534091	1.06E-22	BASEMENT_MEMBRANES	1.056558563	1.12280989
**ANXA10**	2.54477234	2.193632586	0.015295502	2.54477234	1.33505	0.000767955	ECM_AFFILIATED	1.160072273	1.906125119
**ANXA8**	13.16645603	4.785109393	0	13.16645603	12.71096364	0.028187346	ECM_AFFILIATED	2.751547551	1.035834607
**ANXA8L1**	12.07652571	4.967643938	0	12.07652571	11.53127273	0.016651098	ECM_AFFILIATED	2.431036898	1.047284718
**ARTN**	7.862509752	5.254881498	1.00E-176	7.862509752	4.71635	3.59E-26	SECRETED_FACTORS	1.496229697	1.667075122
**ASPN**	9.140427837	8.403881748	1.73E-11	9.140427837	7.020922727	3.34E-11	PROTEOGLYCANS	1.087643557	1.301884124
**BGN**	13.43368511	13.2532102	0.038932474	13.43368511	11.83328182	4.62E-11	PROTEOGLYCANS	1.013617449	1.135245937
**BMP1**	11.46753635	10.57772517	4.05E-78	11.46753635	9.818897727	3.24E-28	ECM_REGULATORS	1.084121223	1.167904653
**BMP2**	9.608940426	7.888544515	2.91E-61	9.608940426	8.775618182	0.000180573	SECRETED_FACTORS	1.218087875	1.094958808
**C1QTNF1**	10.26840638	9.864086471	3.74E-08	10.26840638	9.524468182	0.000113333	ECM_AFFILIATED	1.040989088	1.078108109
**C1QTNF6**	10.02811401	9.306514889	5.48E-34	10.02811401	7.322515909	1.07E-28	ECM_AFFILIATED	1.077536986	1.369490231
**CCL11**	5.397409574	3.82493177	1.55E-37	5.397409574	2.269229545	1.57E-19	SECRETED_FACTORS	1.411112642	2.378520756
**CCL18**	9.035768972	6.932424042	4.52E-47	9.035768972	7.828165909	0.000196076	SECRETED_FACTORS	1.303406848	1.154263857
**CCL20**	8.126826596	5.783442632	1.80E-56	8.126826596	6.537947727	1.44E-05	SECRETED_FACTORS	1.405188417	1.243024101
**CCL26**	4.805993794	3.033188246	2.19E-97	4.805993794	3.874595455	0.002014565	SECRETED_FACTORS	1.584469346	1.240385958
**CCL3**	7.679767199	7.449845244	0.001895206	7.679767199	6.691304545	1.37E-05	SECRETED_FACTORS	1.030862648	1.147723459
**CCL4**	7.884945035	7.697607401	0.010304435	7.884945035	7.254159091	0.004721971	SECRETED_FACTORS	1.024337125	1.086955075
**CCL5**	10.41276259	9.278506976	2.45E-38	10.41276259	9.162879545	2.40E-06	SECRETED_FACTORS	1.122245488	1.136407233
**CCL7**	3.09194078	2.225775566	4.88E-22	3.09194078	1.5775	5.00E-08	SECRETED_FACTORS	1.38915209	1.960025851
**CD109**	12.0543422	9.638371256	1.22E-182	12.0543422	10.98536591	2.34E-11	ECM_REGULATORS	1.250661743	1.09730912
**CLCF1**	7.861660106	7.690002013	0.027969312	7.861660106	7.222940909	0.001436758	SECRETED_FACTORS	1.022322243	1.088429243
**CLEC12A**	5.475124823	5.085718143	2.83E-05	5.475124823	3.7949	8.28E-10	ECM_AFFILIATED	1.076568671	1.442758656
**CLEC12B**	2.127197695	1.684964628	8.52E-10	2.127197695	0.923445455	2.03E-06	ECM_AFFILIATED	1.262458369	2.303544497
**CLEC2B**	9.558077128	8.063107423	9.99E-115	9.558077128	9.0737	0.017736648	ECM_AFFILIATED	1.185408631	1.053382537
**CLEC4D**	2.632829965	2.265460601	4.06E-06	2.632829965	1.790034091	0.0001252	ECM_AFFILIATED	1.162161003	1.470826717
**CLEC6A**	1.689381915	1.437028124	0.000207915	1.689381915	0.695747727	7.37E-06	ECM_AFFILIATED	1.175608109	2.428152977
**CLEC7A**	9.047491844	7.68305222	9.55E-74	9.047491844	7.725547727	1.04E-08	ECM_AFFILIATED	1.17759083	1.17111332
**COL10A1**	8.292123582	6.767186112	3.39E-21	8.292123582	3.586397727	1.49E-26	COLLAGENS	1.225342919	2.312103735

**COL11A1**	8.577338475	7.421047464	3.83E-12	8.577338475	4.628520455	7.38E-16	COLLAGENS	1.155812372	1.853149091
**COL12A1**	12.89354734	11.16124339	2.86E-53	12.89354734	10.255075	1.51E-21	COLLAGENS	1.155207076	1.257284548
**COL13A1**	7.113368972	6.322005039	1.00E-21	7.113368972	4.840290909	6.40E-29	COLLAGENS	1.1251761	1.469616001
**COL16A1**	11.77041348	10.36012609	4.36E-61	11.77041348	10.17894091	1.64E-14	COLLAGENS	1.136126469	1.156349524
**COL17A1**	14.78535408	7.260558424	0	14.78535408	13.81915	0.001286475	COLLAGENS	2.036393513	1.069917765
**COL1A1**	16.26503759	14.59467244	2.55E-35	16.26503759	12.85541818	1.62E-23	COLLAGENS	1.114450335	1.265228199
**COL1A2**	15.49162748	14.42088372	3.59E-20	15.49162748	12.93503864	3.90E-16	COLLAGENS	1.074249525	1.197648335
**COL22A1**	7.618780851	6.534245331	1.35E-19	7.618780851	4.991179545	3.54E-10	COLLAGENS	1.165977166	1.526448965
**COL24A1**	5.590579787	5.304828298	0.001318744	5.590579787	4.103320455	1.28E-08	COLLAGENS	1.053866303	1.36245264
**COL27A1**	10.6764695	9.516521506	2.20E-50	10.6764695	8.75675	4.88E-23	COLLAGENS	1.121887813	1.219227396
**COL2A1**	4.706288121	4.212122029	0.000961517	4.706288121	2.003331818	9.09E-15	COLLAGENS	1.117319985	2.349230456
**COL3A1**	15.58749362	14.16122497	2.36E-28	15.58749362	12.74591364	8.53E-18	COLLAGENS	1.100716474	1.22294047
**COL4A1**	13.46562908	13.20038037	0.001550095	13.46562908	10.77245455	3.34E-31	COLLAGENS	1.02009402	1.250005653
**COL4A1**	13.46562908	13.20038037	0.001550095	13.46562908	10.77245455	3.34E-31	BASEMENT_MEMBRANES	1.02009402	1.250005653
**COL4A2**	13.7168477	13.46444425	0.001752434	13.7168477	11.2737	3.45E-29	COLLAGENS	1.018745923	1.216712144
**COL4A2**	13.7168477	13.46444425	0.001752434	13.7168477	11.2737	3.45E-29	BASEMENT_MEMBRANES	1.018745923	1.216712144
**COL4A5**	11.00873528	9.093607706	1.20E-78	11.00873528	9.027431818	1.80E-19	BASEMENT_MEMBRANES	1.210601517	1.219475871
**COL4A5**	11.00873528	9.093607706	1.20E-78	11.00873528	9.027431818	1.80E-19	COLLAGENS	1.210601517	1.219475871
**COL4A6**	9.309090603	5.843072094	7.72E-163	9.309090603	7.164411364	1.34E-08	COLLAGENS	1.593184279	1.299351772
**COL4A6**	9.309090603	5.843072094	7.72E-163	9.309090603	7.164411364	1.34E-08	BASEMENT_MEMBRANES	1.593184279	1.299351772
**COL5A1**	13.24905248	11.70819783	1.46E-47	13.24905248	10.263775	3.03E-23	COLLAGENS	1.131604767	1.290855702
**COL5A2**	12.72768936	11.42975977	5.35E-41	12.72768936	9.799897727	1.27E-23	COLLAGENS	1.113557031	1.298757366
**COL5A3**	11.02522979	9.373327384	1.83E-94	11.02522979	9.206318182	2.11E-15	COLLAGENS	1.176234365	1.197572099
**COL6A1**	13.86867465	13.21081672	1.27E-18	13.86867465	11.89552727	1.48E-15	COLLAGENS	1.049796916	1.165873049
**COL6A1**	13.86867465	13.21081672	1.27E-18	13.86867465	11.89552727	1.48E-15	BASEMENT_MEMBRANES	1.049796916	1.165873049
**COL6A2**	14.13536099	13.24389445	1.38E-23	14.13536099	12.363275	7.22E-13	COLLAGENS	1.067311511	1.143334674
**COL6A2**	14.13536099	13.24389445	1.38E-23	14.13536099	12.363275	7.22E-13	BASEMENT_MEMBRANES	1.067311511	1.143334674
**COL6A3**	13.75072589	12.54847187	1.63E-23	13.75072589	11.27796818	9.27E-17	BASEMENT_MEMBRANES	1.095808799	1.219255602
**COL6A3**	13.75072589	12.54847187	1.63E-23	13.75072589	11.27796818	9.27E-17	COLLAGENS	1.095808799	1.219255602
**COL7A1**	13.42070461	8.794812919	0	13.42070461	11.14698864	4.35E-26	COLLAGENS	1.525979544	1.203975804
**COL8A2**	9.681838652	8.872417403	1.37E-18	9.681838652	8.981990909	0.001004895	COLLAGENS	1.091228942	1.077916773
**CRELD2**	10.58404149	10.36891182	3.54E-09	10.58404149	10.11055	2.91E-06	ECM_GLYCOPROTEINS	1.020747565	1.046831428
**CRISPLD2**	10.38468511	10.21860656	0.029887909	10.38468511	9.622375	0.000251088	ECM_GLYCOPROTEINS	1.016252563	1.079222656
**CRLF3**	9.561390957	8.999731443	1.75E-48	9.561390957	9.312945455	0.013262946	SECRETED_FACTORS	1.062408475	1.026677436
**CSF2**	4.917521631	1.803928526	1.94E-247	4.917521631	1.779447727	1.20E-16	SECRETED_FACTORS	2.726006912	2.763510024
**CSH2**	0.50612961	0.354145385	0.000718265	0.50612961	0.027243182	0.005772246	SECRETED_FACTORS	1.429157716	18.57821209
**CSPG4**	11.51334344	10.21595992	8.57E-50	11.51334344	9.946181818	1.39E-07	ECM_AFFILIATED	1.126995753	1.157564144
**CST1**	6.577290957	4.974311134	3.28E-19	6.577290957	4.508120455	0.000403519	ECM_REGULATORS	1.32225162	1.458987404
**CSTL1**	0.795301773	0.531965836	3.24E-10	0.795301773	0.456052273	0.016950456	ECM_REGULATORS	1.495024153	1.743882929
**CTHRC1**	9.880125887	8.99135062	1.74E-15	9.880125887	6.681602273	3.89E-26	ECM_GLYCOPROTEINS	1.098847804	1.478706077
**CTSB**	15.5636984	15.22599296	9.42E-10	15.5636984	15.11861364	0.000245571	ECM_REGULATORS	1.022179535	1.029439523
**CTSC**	13.31164982	12.06247845	7.12E-79	13.31164982	12.156625	3.00E-13	ECM_REGULATORS	1.103558434	1.095011964
**CTSK**	11.05114326	10.316325	4.65E-16	11.05114326	9.913252273	2.69E-06	ECM_REGULATORS	1.071228685	1.114784831
**CTSV**	11.08001206	7.038075392	1.51E-301	11.08001206	9.122595455	2.65E-15	ECM_REGULATORS	1.574295733	1.21456795
**CXCL1**	10.01229291	6.587876067	1.75E-132	10.01229291	9.311431818	0.038088185	SECRETED_FACTORS	1.519805899	1.075268885
**CXCL10**	10.17109929	8.216557989	7.63E-65	10.17109929	7.718206818	8.83E-09	SECRETED_FACTORS	1.237878355	1.317806005

**CXCL11**	8.059074113	6.317128309	1.18E-52	8.059074113	5.387018182	3.57E-09	SECRETED_FACTORS	1.275749632	1.496017619
**CXCL13**	8.683501064	6.126360231	1.29E-68	8.683501064	5.522095455	4.54E-19	SECRETED_FACTORS	1.417399685	1.57250108
**CXCL3**	6.127595922	5.265943317	7.62E-14	6.127595922	5.365513636	0.008202595	SECRETED_FACTORS	1.163627398	1.142033426
**CXCL5**	6.144514184	5.007960057	1.50E-14	6.144514184	4.388020455	3.53E-05	SECRETED_FACTORS	1.226949519	1.400292968
**CXCL8**	10.49552943	7.774415085	1.31E-111	10.49552943	8.950822727	2.29E-06	SECRETED_FACTORS	1.350008884	1.172577064
**CXCL9**	10.44496365	8.855705986	2.27E-35	10.44496365	8.271879545	8.54E-08	SECRETED_FACTORS	1.179461431	1.262707417
**EGFL6**	8.145789539	5.613663158	7.79E-82	8.145789539	6.792277273	8.94E-09	SECRETED_FACTORS	1.451064895	1.199272234
**EGFLAM**	7.889874645	7.639742403	0.001291868	7.889874645	7.079447727	2.93E-05	ECM_GLYCOPROTEINS	1.032740926	1.114476008
**EGLN3**	10.46199149	9.469607858	8.70E-22	10.46199149	9.636084091	0.00184461	ECM_REGULATORS	1.104796698	1.085709858
**EPO**	2.394135993	2.125263975	0.002913864	2.394135993	1.706686364	0.013060045	SECRETED_FACTORS	1.126512293	1.402797868
**F12**	9.042606915	7.072578026	2.49E-74	9.042606915	8.526218182	0.004633166	ECM_REGULATORS	1.278544667	1.060564804
**FBN1**	11.47918032	11.2276634	0.001784653	11.47918032	10.79095682	0.012769299	ECM_GLYCOPROTEINS	1.022401537	1.063777801
**FBN2**	8.900113475	7.06951108	8.45E-61	8.900113475	6.250988636	1.92E-12	ECM_GLYCOPROTEINS	1.258943281	1.423792938
**FGF11**	9.828681028	7.455610263	1.09E-139	9.828681028	9.034220455	0.000184517	SECRETED_FACTORS	1.318293296	1.087939029
**FGF19**	2.173263298	1.87229003	0.010612808	2.173263298	0.557827273	0.000101682	SECRETED_FACTORS	1.160751413	3.895943071
**FNDC1**	8.842	8.254219536	1.36E-05	8.842	6.809625	6.12E-09	ECM_GLYCOPROTEINS	1.071209696	1.298456229
**GPC1**	13.63316241	11.38650372	3.91E-255	13.63316241	12.97210909	1.19E-06	ECM_AFFILIATED	1.197308914	1.050959587
**GREM1**	10.27995904	7.815446474	5.00E-58	10.27995904	7.713704545	4.19E-17	ECM_AFFILIATED	1.315338679	1.332687684
**HAPLN1**	5.888536348	5.192622214	1.54E-07	5.888536348	2.883011364	5.83E-25	PROTEOGLYCANS	1.134019789	2.042495018
**HAPLN3**	9.942170213	8.387811025	2.97E-92	9.942170213	9.083365909	1.21E-07	PROTEOGLYCANS	1.18531166	1.094546924
**HMSD**	4.224640426	2.728630736	1.40E-69	4.224640426	3.036027273	6.28E-05	ECM_REGULATORS	1.548263886	1.391502792
**HSPG2**	13.66084131	13.38147567	1.92E-05	13.66084131	12.84305909	5.53E-07	BASEMENT_MEMBRANES	1.020877043	1.063675034
**HSPG2**	13.66084131	13.38147567	1.92E-05	13.66084131	12.84305909	5.53E-07	PROTEOGLYCANS	1.020877043	1.063675034
**HTRA1**	12.09709929	11.83951613	0.001648197	12.09709929	10.749425	9.10E-11	ECM_REGULATORS	1.021756224	1.125371756
**HTRA3**	10.29356773	9.168616141	2.76E-26	10.29356773	8.240006818	3.13E-13	ECM_REGULATORS	1.122695898	1.249218351
**IBSP**	4.392891489	3.203609632	1.18E-24	4.392891489	1.263134091	1.03E-19	ECM_GLYCOPROTEINS	1.371231827	3.4777713
**IGFBP3**	12.74142465	12.2994363	1.22E-06	12.74142465	11.46306136	1.05E-07	ECM_GLYCOPROTEINS	1.035935659	1.111520234
**KCP**	6.914541844	5.804015945	3.46E-28	6.914541844	6.235527273	0.002152512	ECM_GLYCOPROTEINS	1.1913375	1.108894491
**LAMA1**	7.705062234	7.464246593	0.042844921	7.705062234	5.303031818	6.17E-10	BASEMENT_MEMBRANES	1.032262552	1.452954178
**LAMA1**	7.705062234	7.464246593	0.042844921	7.705062234	5.303031818	6.17E-10	ECM_GLYCOPROTEINS	1.032262552	1.452954178
**LAMA3**	13.33518741	9.549523324	3.57E-248	13.33518741	11.13958182	7.59E-16	BASEMENT_MEMBRANES	1.396424404	1.197099463
**LAMA3**	13.33518741	9.549523324	3.57E-248	13.33518741	11.13958182	7.59E-16	ECM_GLYCOPROTEINS	1.396424404	1.197099463
**LAMA5**	12.86174521	12.24232562	2.69E-23	12.86174521	12.09035909	1.31E-10	ECM_GLYCOPROTEINS	1.050596562	1.063801754
**LAMA5**	12.86174521	12.24232562	2.69E-23	12.86174521	12.09035909	1.31E-10	BASEMENT_MEMBRANES	1.050596562	1.063801754
**LAMB1**	12.51245621	12.20358609	2.34E-05	12.51245621	11.19618636	4.26E-17	ECM_GLYCOPROTEINS	1.025309783	1.117564124
**LAMB1**	12.51245621	12.20358609	2.34E-05	12.51245621	11.19618636	4.26E-17	BASEMENT_MEMBRANES	1.025309783	1.117564124
**LAMB3**	14.67629291	10.41794833	1.96E-277	14.67629291	13.03279773	2.30E-15	ECM_GLYCOPROTEINS	1.408750786	1.126104557
**LAMB3**	14.67629291	10.41794833	1.96E-277	14.67629291	13.03279773	2.30E-15	BASEMENT_MEMBRANES	1.408750786	1.126104557
**LAMC1**	12.95617128	12.82066114	0.019953475	12.95617128	11.89305682	1.51E-13	BASEMENT_MEMBRANES	1.010569668	1.089389505
**LAMC1**	12.95617128	12.82066114	0.019953475	12.95617128	11.89305682	1.51E-13	ECM_GLYCOPROTEINS	1.010569668	1.089389505
**LAMC2**	14.71451206	9.673943753	1.30E-198	14.71451206	11.42653636	3.18E-26	ECM_GLYCOPROTEINS	1.521045856	1.287749112
**LAMC2**	14.71451206	9.673943753	1.30E-198	14.71451206	11.42653636	3.18E-26	BASEMENT_MEMBRANES	1.521045856	1.287749112
**LGALS1**	13.23956294	12.66720456	9.28E-21	13.23956294	11.61540455	6.15E-14	ECM_AFFILIATED	1.045184269	1.139827967
**LOX**	10.29464043	9.47525591	5.32E-19	10.29464043	8.968677273	5.47E-08	ECM_REGULATORS	1.086476241	1.14784378
**LOXL1**	9.525580851	9.174167468	5.06E-05	9.525580851	8.799395455	0.000308855	ECM_REGULATORS	1.038304662	1.082526737
**LOXL2**	10.87377535	10.13900373	8.16E-22	10.87377535	7.924936364	1.03E-29	ECM_REGULATORS	1.072469805	1.372096236

**LTBP1**	12.13962039	10.73405085	2.57E-81	12.13962039	10.62178409	5.42E-11	ECM_GLYCOPROTEINS	1.13094493	1.142898433
**LTBP2**	12.01934007	11.21481983	2.12E-21	12.01934007	11.17380227	7.87E-06	ECM_GLYCOPROTEINS	1.071737242	1.075671448
**LUM**	12.83399504	11.49733654	3.87E-22	12.83399504	10.91654318	2.93E-13	PROTEOGLYCANS	1.1162581	1.175646431
**MFAP1**	10.56507872	10.35962327	6.98E-21	10.56507872	10.41875227	0.010646991	ECM_GLYCOPROTEINS	1.019832329	1.014044527
**MFAP2**	10.90941702	8.904379745	2.70E-64	10.90941702	7.977972727	4.73E-30	ECM_GLYCOPROTEINS	1.225174278	1.367442256
**MMP1**	12.68449504	6.509346419	9.05E-286	12.68449504	7.607865909	2.10E-26	ECM_REGULATORS	1.948658777	1.667286883
**MMP10**	10.23030355	4.176157259	0	10.23030355	6.923118182	1.51E-11	ECM_REGULATORS	2.449693082	1.477701706
**MMP11**	11.05668812	9.817103461	1.50E-23	11.05668812	6.673270455	6.14E-30	ECM_REGULATORS	1.126267861	1.656861983
**MMP12**	10.06848564	5.248954386	7.94E-187	10.06848564	6.373284091	6.33E-27	ECM_REGULATORS	1.918188823	1.579795517
**MMP13**	9.423479078	3.879871474	1.96E-236	9.423479078	4.072188636	7.78E-21	ECM_REGULATORS	2.428812176	2.314106717
**MMP14**	14.19266507	12.80056123	8.42E-76	14.19266507	12.78506591	8.69E-22	ECM_REGULATORS	1.108753344	1.110097138
**MMP17**	7.641632624	6.561373182	7.99E-27	7.641632624	6.440379545	6.47E-09	ECM_REGULATORS	1.164639232	1.186518989
**MMP19**	8.607979078	7.869768524	7.14E-23	8.607979078	7.177961364	2.95E-12	ECM_REGULATORS	1.093803338	1.199223379
**MMP2**	12.99935195	12.20524881	1.55E-19	12.99935195	11.67833182	2.58E-07	ECM_REGULATORS	1.065062429	1.113117195
**MMP25**	7.006467021	6.273813703	2.17E-29	7.006467021	6.292288636	0.000270202	ECM_REGULATORS	1.116779578	1.113500576
**MMP28**	9.381712943	6.980837886	1.72E-147	9.381712943	8.479990909	0.001574075	ECM_REGULATORS	1.343923623	1.10633526
**MMP3**	10.09752518	4.050837788	5.36E-304	10.09752518	6.433013636	7.50E-15	ECM_REGULATORS	2.49270045	1.569641501
**MMP7**	9.027723404	8.186261406	2.85E-06	9.027723404	7.051911364	1.28E-05	ECM_REGULATORS	1.102789534	1.280181066
**MMP8**	2.558814539	2.003374782	3.55E-08	2.558814539	0.998368182	5.69E-07	ECM_REGULATORS	1.277252046	2.562996884
**MMP9**	11.62381312	9.218202699	1.34E-85	11.62381312	7.799352273	4.13E-29	ECM_REGULATORS	1.260963064	1.490356213
**MXRA5**	11.9320406	10.96681191	6.89E-18	11.9320406	11.27	0.008792321	ECM_GLYCOPROTEINS	1.088013609	1.05874362
**NELL2**	10.07812039	7.786844362	5.96E-86	10.07812039	7.361497727	1.34E-13	ECM_GLYCOPROTEINS	1.294249624	1.369031244
**NPNT**	10.70802979	10.23714763	6.68E-05	10.70802979	9.329920455	3.13E-10	ECM_GLYCOPROTEINS	1.045997399	1.147708583
**NPNT**	10.70802979	10.23714763	6.68E-05	10.70802979	9.329920455	3.13E-10	BASEMENT_MEMBRANES	1.045997399	1.147708583
**P4HA2**	11.68479043	10.62915453	9.97E-63	11.68479043	10.56820455	1.18E-13	ECM_REGULATORS	1.099315133	1.105655211
**PAPLN**	9.446656738	9.232874075	0.005014819	9.446656738	8.482959091	8.09E-07	ECM_GLYCOPROTEINS	1.023154509	1.113603948
**PAPLN**	9.446656738	9.232874075	0.005014819	9.446656738	8.482959091	8.09E-07	BASEMENT_MEMBRANES	1.023154509	1.113603948
**PLAU**	12.92889716	10.34115838	5.05E-183	12.92889716	10.50826136	3.43E-25	ECM_REGULATORS	1.250236839	1.2303555
**PLXNA1**	12.77613812	11.39477878	6.11E-139	12.77613812	11.51009091	5.86E-23	ECM_AFFILIATED	1.121227395	1.109994545
**PLXNB2**	13.8183516	13.65677356	2.54E-05	13.8183516	13.61067273	0.029434193	ECM_AFFILIATED	1.011831347	1.015258531
**PLXNB3**	9.12092039	8.229032259	7.74E-15	9.12092039	8.265372727	1.16E-07	ECM_AFFILIATED	1.108383113	1.103509871
**PODNL1**	8.254925355	6.502546702	7.54E-75	8.254925355	7.558140909	0.000436448	PROTEOGLYCANS	1.269491129	1.092189925
**POSTN**	12.17274202	10.82205528	1.15E-18	12.17274202	9.266120455	1.64E-16	ECM_GLYCOPROTEINS	1.124808709	1.313682687
**SDC1**	15.40230408	12.08857194	1.92E-204	15.40230408	14.80989318	0.000106916	ECM_AFFILIATED	1.274121058	1.040001024
**SEMA3C**	11.11344663	9.935833391	3.90E-25	11.11344663	10.06454091	3.30E-05	ECM_AFFILIATED	1.118521838	1.10421794
**SEMA4B**	13.2726672	11.68045987	3.01E-203	13.2726672	12.86804318	0.004110259	ECM_AFFILIATED	1.136313753	1.031444098
**SEMA7A**	9.143028191	8.367978994	2.34E-27	9.143028191	8.04295	6.16E-11	ECM_AFFILIATED	1.092620834	1.136775461
**SERPINA9**	2.749595745	1.30724655	1.30E-72	2.749595745	0.999440909	3.83E-07	ECM_REGULATORS	2.103349016	2.751133879
**SERPINB7**	8.374102837	2.707530387	0	8.374102837	7.466768182	0.009968649	ECM_REGULATORS	3.092893389	1.121516382
**SERPINE1**	12.7405383	10.57539853	4.26E-112	12.7405383	9.908793182	3.18E-19	ECM_REGULATORS	1.204733634	1.285781029
**SERPINE2**	11.3924789	10.83624116	2.42E-08	11.3924789	10.39336591	0.000125248	ECM_REGULATORS	1.051331244	1.096129877
**SERPINH1**	13.20814628	12.63826873	8.31E-21	13.20814628	11.16637955	1.91E-27	ECM_REGULATORS	1.045091425	1.182849483
**SPON2**	11.01452234	10.30146379	1.29E-12	11.01452234	10.29451136	0.000431021	ECM_GLYCOPROTEINS	1.069219148	1.069941248
**SULF1**	11.33008014	11.06337253	0.0128019	11.33008014	9.711231818	4.44E-09	ECM_REGULATORS	1.024107261	1.166698556
**SULF2**	13.04080142	11.77359221	4.14E-73	13.04080142	12.10724773	6.80E-06	ECM_REGULATORS	1.107631485	1.077107012
**TGFBI**	14.05956844	12.19407224	3.39E-103	14.05956844	11.25018409	1.31E-21	ECM_GLYCOPROTEINS	1.152983857	1.249718967

**TGM4**	3.408146454	1.955925218	1.67E-44	3.408146454	2.311361364	1.60E-06	ECM_REGULATORS	1.742472781	1.47451909
**THBS2**	11.90535124	11.14111553	2.13E-14	11.90535124	10.28936136	1.42E-08	ECM_GLYCOPROTEINS	1.068595977	1.157054439
**TINAGL1**	11.080425	10.43268998	5.39E-14	11.080425	9.852768182	4.88E-08	ECM_GLYCOPROTEINS	1.062087058	1.124600193
**TNC**	14.30787305	11.74784416	9.39E-139	14.30787305	12.53642273	1.05E-09	ECM_GLYCOPROTEINS	1.217914782	1.141304291
**TNFAIP6**	7.613089894	6.48153929	8.80E-25	7.613089894	6.1573	2.38E-08	ECM_GLYCOPROTEINS	1.174580536	1.23643316
**TSKU**	11.90481312	10.73257483	2.85E-53	11.90481312	11.61267727	0.012422396	ECM_GLYCOPROTEINS	1.109222467	1.025156632
**WISP1**	8.281605142	7.293077917	7.09E-23	8.281605142	5.257627273	1.10E-25	ECM_GLYCOPROTEINS	1.135543215	1.575160184
**WISP3**	5.01121117	4.005596049	7.55E-29	5.01121117	3.625781818	0.000628306	ECM_GLYCOPROTEINS	1.251052555	1.382104997
**WNT10A**	8.857202837	5.674497355	1.15E-162	8.857202837	7.922647727	0.001863564	SECRETED_FACTORS	1.560878838	1.117959947
**WNT10B**	5.586805496	4.127468111	5.80E-69	5.586805496	5.049140909	0.031350554	SECRETED_FACTORS	1.35356721	1.10648635
**WNT2**	6.210688121	5.484353537	3.34E-08	6.210688121	3.074609091	3.50E-17	SECRETED_FACTORS	1.132437593	2.019992766
**WNT2B**	8.482610993	7.515716369	4.57E-40	8.482610993	7.712663636	0.002328686	SECRETED_FACTORS	1.128649696	1.099828982
**WNT3A**	7.17197766	2.46023867	0	7.17197766	6.539538636	0.012500959	SECRETED_FACTORS	2.915155244	1.096710037
**WNT7A**	7.420093972	3.42596503	1.87E-167	7.420093972	5.332127273	1.67E-07	SECRETED_FACTORS	2.165840546	1.391582307
**WNT7B**	11.18605248	6.71497389	3.44E-185	11.18605248	9.545902273	3.29E-14	SECRETED_FACTORS	1.665837078	1.171817201
**XCL1**	5.053192199	4.009227797	4.28E-34	5.053192199	3.732634091	1.13E-07	SECRETED_FACTORS	1.260390393	1.353787185
**ZP3**	8.373817553	7.723563354	1.13E-23	8.373817553	7.464363636	1.66E-07	ECM_GLYCOPROTEINS	1.084190958	1.121839444
**ZP4**	1.205608511	0.317821985	2.47E-87	1.205608511	0.260545455	3.44E-05	ECM_GLYCOPROTEINS	3.793345227	4.627248296

Kidney Chromophobe Cell Carcinoma (KICH)

Signature genes	Expression in tumor	Expression in rest of Pan-Cancer	P-value	Expression in tumor	Expression in normal tissue	P-value	Matrisome category	Fold change vs. Pan-Cancer	Fold change vs. normal
ADAM2	4.082815385	0.556721136	5.39E-105	4.082815385	1.313852	3.68E-05	ECM_REGULATORS	7.333681301	3.107515447
ADAMTSL1	10.45967363	6.85884034	1.05E-64	10.45967363	8.640016	0.001427815	ECM_REGULATORS	1.524991559	1.210608132
AMBN	0.737930769	0.363815372	0.000581757	0.737930769	0.225992	0.001785494	ECM_GLYCOPROTEINS	2.028311135	3.265295981
ANGPT1	10.58045165	7.036744816	1.62E-62	10.58045165	9.336088	0.000260589	SECRETED_FACTORS	1.503600304	1.133285338
CCBE1	9.417736264	4.770810695	3.07E-70	9.417736264	7.967592	0.000863116	SECRETED_FACTORS	1.974032689	1.182005337
COCH	7.982751648	6.597409565	1.59E-07	7.982751648	6.509388	0.008609444	ECM_GLYCOPROTEINS	1.209982732	1.22634442
CTSA	13.80329121	12.75228011	1.41E-26	13.80329121	13.024148	7.14E-06	ECM_REGULATORS	1.082417504	1.05982297
CTSD	17.0778033	15.04259047	6.83E-80	17.0778033	15.668436	2.48E-09	ECM_REGULATORS	1.135296699	1.089949456
EDA	9.3144	7.1486447	4.82E-29	9.3144	8.330912	0.000247674	SECRETED_FACTORS	1.302960266	1.118052861
EGLN2	11.86002967	11.46866805	2.22E-08	11.86002967	11.25576	2.19E-06	ECM_REGULATORS	1.034124418	1.053685373
ELSPBP1	1.735616484	0.400242473	2.00E-28	1.735616484	0.644264	0.022405719	ECM_GLYCOPROTEINS	4.336412556	2.693952298
ESM1	9.752767033	7.370375887	1.88E-23	9.752767033	8.584032	0.000567929	PROTEOGLYCANS	1.323238758	1.136152222
F7	5.671462637	3.953165836	9.00E-08	5.671462637	4.243908	0.00187656	ECM_REGULATORS	1.434663475	1.336377376
FBN3	9.929897802	4.399688545	7.07E-53	9.929897802	7.456724	0.000142475	ECM_GLYCOPROTEINS	2.256954714	1.331670289
FGF13	9.153572527	7.191609269	2.74E-16	9.153572527	8.092036	0.000326565	SECRETED_FACTORS	1.272812827	1.131182873
FGF14	8.339159341	5.464790762	3.30E-27	8.339159341	6.307288	0.0009575	SECRETED_FACTORS	1.525979622	1.322146593
GAS6	13.99842637	11.70844753	9.08E-50	13.99842637	13.270436	4.68E-05	ECM_GLYCOPROTEINS	1.195583474	1.05485806
GLDN	8.507069231	6.26903598	1.41E-21	8.507069231	5.056184	5.50E-06	ECM_GLYCOPROTEINS	1.356997991	1.682507842
ITIH1	3.240041758	2.262396326	0.005539042	3.240041758	1.097	4.62E-06	ECM_REGULATORS	1.43212828	2.953547637
LAMA5	12.92159341	12.27180755	2.20E-05	12.92159341	12.517688	0.024712695	ECM_GLYCOPROTEINS	1.052949482	1.032266774
LAMA5	12.92159341	12.27180755	2.20E-05	12.92159341	12.517688	0.024712695	BASEMENT_MEMBRANES	1.052949482	1.032266774
LAMB4	8.088335165	2.812860536	2.10E-200	8.088335165	6.927324	0.023507155	ECM_GLYCOPROTEINS	2.875483893	1.167598796
LAMB4	8.088335165	2.812860536	2.10E-200	8.088335165	6.927324	0.023507155	BASEMENT_MEMBRANES	2.875483893	1.167598796
LGALS3	14.31726374	12.37218833	7.24E-26	14.31726374	12.24306	1.75E-09	ECM_AFFILIATED	1.15721353	1.169418735
LMAN1	13.23705604	12.33211867	8.96E-26	13.23705604	12.767116	0.006457524	ECM_AFFILIATED	1.073380528	1.03680863
MEPE	1.693049451	1.017366121	3.39E-05	1.693049451	0.576976	0.000549147	ECM_GLYCOPROTEINS	1.664149626	2.93434987
MMP15	11.55500769	10.78826829	2.58E-06	11.55500769	10.702056	0.000259217	ECM_REGULATORS	1.071071592	1.079699797
MMP24	11.26327582	6.583126742	8.91E-89	11.26327582	9.643124	3.94E-05	ECM_REGULATORS	1.710931031	1.168011095
MMP26	2.326157143	0.445473057	8.39E-36	2.326157143	0.384288	0.000184698	ECM_REGULATORS	5.221768419	6.053161022
MUC20	14.11225495	8.917898849	2.07E-77	14.11225495	12.263312	0.00083432	ECM_AFFILIATED	1.582464119	1.150770277
MUCL1	3.848931868	2.809124356	0.004956504	3.848931868	1.76462	0.003099099	ECM_AFFILIATED	1.370153607	2.181167542
NTN1	11.17801758	8.63186912	2.03E-25	11.17801758	8.423124	5.74E-10	ECM_GLYCOPROTEINS	1.294970698	1.327063164
NTN1	11.17801758	8.63186912	2.03E-25	11.17801758	8.423124	5.74E-10	BASEMENT_MEMBRANES	1.294970698	1.327063164
NTN3	2.76332967	2.20448523	0.001396752	2.76332967	1.88634	0.014367241	ECM_GLYCOPROTEINS	1.253503372	1.464916012
NTN3	2.76332967	2.20448523	0.001396752	2.76332967	1.88634	0.014367241	BASEMENT_MEMBRANES	1.253503372	1.464916012
OGFOD2	9.536465934	9.056172382	6.57E-12	9.536465934	9.146448	0.000325649	ECM_REGULATORS	1.053034939	1.042641464
PRSS1	3.109471429	2.259461729	0.013080291	3.109471429	0.582472	6.17E-05	ECM_REGULATORS	1.376200087	5.338404985
SDC4	14.98805824	12.74528157	5.19E-34	14.98805824	14.201924	1.60E-05	ECM_AFFILIATED	1.175969174	1.055354066
SEMA3C	11.78956264	10.13217717	2.59E-09	11.78956264	10.273432	0.000409658	ECM_AFFILIATED	1.163576439	1.147577814
SEMA3F	11.99166923	10.55298595	1.50E-13	11.99166923	11.052212	0.00018665	ECM_AFFILIATED	1.136329499	1.085001738
SEMA6B	9.723021978	8.737020281	1.86E-10	9.723021978	8.978488	0.042820798	ECM_AFFILIATED	1.112853314	1.082924205
SERPINI1	10.6349	7.887681936	8.00E-58	10.6349	9.182568	3.81E-06	ECM_REGULATORS	1.348292196	1.158161856

**SFTPB**	8.603405495	4.253465498	1.17E-14	8.603405495	4.432088	4.40E-07	ECM_AFFILIATED	2.022681387	1.941163058
**SLIT2**	11.10335934	8.224947487	2.04E-35	11.10335934	8.929716	2.30E-05	ECM_GLYCOPROTEINS	1.349961122	1.24341685
**SMOC1**	9.778545055	7.171748427	3.49E-13	9.778545055	7.537304	0.000893658	ECM_GLYCOPROTEINS	1.363481326	1.297353146
**SPARCL1**	14.29788791	12.32108392	6.97E-17	14.29788791	12.616812	0.000179104	ECM_GLYCOPROTEINS	1.160440754	1.133240942
**ST14**	13.92157582	11.7281401	2.62E-15	13.92157582	12.955956	0.000117234	ECM_REGULATORS	1.187023322	1.074530959
**WFIKKN1**	6.769836264	4.942840678	4.07E-27	6.769836264	6.055988	0.019976603	SECRETED_FACTORS	1.369624616	1.117874782
**VWF**	12.76904176	12.07067299	2.41E-06	12.76904176	11.383168	4.30E-05	ECM_GLYCOPROTEINS	1.057856655	1.121747633

Kidney Clear Cell Carcinoma (KIRC)

Signature genes	Expression in tumor	Expression in rest of Pan-Cancer	P-value	Expression in tumor	Expression in normal tissue	P-value	Matrisome category	Fold change vs. Pan-Cancer	Fold change vs. normal
**A2M**	15.70100149	14.02514141	5.57E-110	15.70100149	15.48531111	0.035654791	ECM_REGULATORS	1.11948971	1.013928708
**ACAN**	9.72783466	6.316032264	1.80E-263	9.72783466	7.435811111	7.22E-21	PROTEOGLYCANS	1.540181281	1.308241228
**ADAM18**	3.200157877	0.293273311	0	3.200157877	0.136519444	1.52E-20	ECM_REGULATORS	10.91186194	23.44104087
**ADAM28**	9.036876285	7.966519172	1.62E-38	9.036876285	7.827138889	1.86E-16	ECM_REGULATORS	1.134356937	1.154556782
**ADAM9**	12.57898557	11.75748795	1.65E-51	12.57898557	12.29076806	0.00220817	ECM_REGULATORS	1.069870165	1.023449919
**ADAMTS10**	8.550409453	7.606247466	1.91E-49	8.550409453	6.801998611	7.70E-22	ECM_REGULATORS	1.124129801	1.257043693
**ADAMTS18**	5.619278441	3.755358034	1.45E-82	5.619278441	4.660552778	5.63E-07	ECM_REGULATORS	1.496336272	1.205710719
**ADAMTS4**	10.82078358	9.142641237	1.73E-106	10.82078358	8.412451389	9.53E-26	ECM_REGULATORS	1.183551153	1.286281856
**ADAMTS5**	10.12485904	7.573374989	6.80E-229	10.12485904	9.530538889	4.03E-06	ECM_REGULATORS	1.336901851	1.062359553
**ADAMTS7**	8.48764461	8.054007021	2.49E-11	8.48764461	6.316693056	3.87E-40	ECM_REGULATORS	1.053841223	1.343684826
**ADAMTSL4**	9.131905307	8.831679561	7.65E-05	9.131905307	7.438222222	3.23E-24	ECM_REGULATORS	1.033994185	1.227699984
**AGRN**	14.00731376	13.21223926	1.39E-48	14.00731376	13.79238889	0.02006401	BASEMENT_MEMBRANES	1.06017712	1.015582861
**AGRN**	14.00731376	13.21223926	1.39E-48	14.00731376	13.79238889	0.02006401	ECM_GLYCOPROTEINS	1.06017712	1.015582861
**AGT**	11.19882537	8.26131118	2.90E-86	11.19882537	9.989651389	1.11E-07	ECM_REGULATORS	1.355574815	1.121042661
**ANGPT2**	11.42097861	8.447859692	0	11.42097861	8.524740278	3.74E-39	SECRETED_FACTORS	1.351937535	1.339745052
**ANGPT4**	4.893195688	2.985718317	6.32E-122	4.893195688	4.170411111	9.07E-05	SECRETED_FACTORS	1.638867156	1.173312548
**ANGPTL2**	11.33397944	10.42380202	6.24E-35	11.33397944	10.09700556	1.85E-16	SECRETED_FACTORS	1.087317221	1.122508983
**ANGPTL4**	13.67519701	9.073770735	0	13.67519701	8.673445833	9.46E-49	SECRETED_FACTORS	1.50711291	1.576674055
**ANXA13**	8.106730182	3.573841375	2.63E-254	8.106730182	6.476006944	2.28E-06	ECM_AFFILIATED	2.268351987	1.251809989
**ANXA4**	14.43841128	11.74772896	0	14.43841128	12.88178333	1.29E-35	ECM_AFFILIATED	1.229038508	1.120839476
**ANXA5**	13.85351526	13.35401873	4.38E-30	13.85351526	13.39930139	3.69E-16	ECM_AFFILIATED	1.037404211	1.033898325
**ANXA6**	12.77003367	12.23624529	1.45E-24	12.77003367	12.48158194	6.93E-05	ECM_AFFILIATED	1.043623543	1.023110189
**BDNF**	6.437959701	5.149569943	1.30E-48	6.437959701	5.1248875	1.76E-09	SECRETED_FACTORS	1.250193661	1.256214834
**BGN**	14.6958325	13.16003454	1.95E-75	14.6958325	13.89452361	6.82E-07	PROTEOGLYCANS	1.116701666	1.057670843
**BMP2**	9.390164179	7.930017789	8.09E-47	9.390164179	8.684381944	1.13E-07	SECRETED_FACTORS	1.184129018	1.081270289
**C1QA**	12.37058905	11.42867589	1.21E-37	12.37058905	9.954880556	1.74E-36	ECM_AFFILIATED	1.082416649	1.242665744
**C1QB**	12.47623002	11.59366766	5.00E-30	12.47623002	9.824451389	1.57E-38	ECM_AFFILIATED	1.076124517	1.269916204
**C1QC**	12.22649685	11.43836338	5.25E-26	12.22649685	9.682797222	1.92E-37	ECM_AFFILIATED	1.068902643	1.262702974
**C1QL1**	7.913460697	5.832533995	1.32E-59	7.913460697	4.984013889	2.08E-15	ECM_AFFILIATED	1.356779181	1.587768588
**C1QL4**	5.976339801	3.416379244	9.45E-108	5.976339801	4.105866667	1.95E-08	ECM_AFFILIATED	1.749319784	1.4555611
**C1QTNF1**	10.57609784	9.796196717	1.38E-27	10.57609784	9.7274625	1.50E-07	ECM_AFFILIATED	1.079612645	1.087241184
**C1QTNF5**	10.41621177	9.229578758	2.06E-67	10.41621177	9.720984722	2.88E-09	ECM_AFFILIATED	1.128568491	1.071518171
**CBLN3**	7.894085738	6.674096505	4.25E-123	7.894085738	6.601611111	6.88E-22	SECRETED_FACTORS	1.182794665	1.195781697
**CBLN4**	6.144408955	3.16523712	2.52E-171	6.144408955	4.858958333	1.14E-07	SECRETED_FACTORS	1.941216004	1.264552716
**CCL15**	6.716117579	2.358480184	1.45E-219	6.716117579	5.998088889	0.009232839	SECRETED_FACTORS	2.847646389	1.119709578
**CCL20**	7.643207463	5.937788081	1.91E-32	7.643207463	5.1621125	9.42E-15	SECRETED_FACTORS	1.287214592	1.480635585
**CCL28**	9.394191045	7.846971484	3.40E-55	9.394191045	8.237198611	9.50E-09	SECRETED_FACTORS	1.197174103	1.140459456
**CCL3**	7.775709121	7.442840382	3.78E-06	7.775709121	6.108431944	2.47E-16	SECRETED_FACTORS	1.044723348	1.272946837
**CCL3L3**	5.880202819	5.502708425	2.98E-06	5.880202819	4.569527778	9.90E-08	SECRETED_FACTORS	1.068601562	1.286829429
**CCL4**	8.653224046	7.664904751	7.20E-44	8.653224046	6.309963889	3.95E-31	SECRETED_FACTORS	1.128940845	1.371358727
**CCL4L2**	7.182091874	6.474404508	4.96E-18	7.182091874	5.100302778	1.58E-16	SECRETED_FACTORS	1.109305399	1.408169708
**CCL5**	10.46199353	9.337918444	2.27E-39	10.46199353	7.666788889	7.64E-36	SECRETED_FACTORS	1.12037748	1.364586098

**CD109**	11.34672322	9.706693571	1.18E-86	11.34672322	10.44636389	4.11E-10	ECM_REGULATORS	1.16895863	1.086188777
**CD209**	7.545014096	6.79707709	6.37E-19	7.545014096	6.678206944	1.38E-06	ECM_AFFILIATED	1.110038035	1.12979639
**CDCP2**	1.508277612	0.839142293	4.33E-68	1.508277612	1.146961111	0.002896403	ECM_GLYCOPROTEINS	1.797403878	1.315020708
**CHRD**	8.579543947	7.613930184	1.11E-30	8.579543947	8.234683333	0.02320428	SECRETED_FACTORS	1.126821988	1.041879038
**CLEC10A**	7.000780763	6.170362648	1.44E-20	7.000780763	6.313555556	0.000240295	ECM_AFFILIATED	1.134581736	1.108849158
**CLEC12A**	6.440212438	5.032945365	7.25E-55	6.440212438	3.9739375	6.38E-32	ECM_AFFILIATED	1.279611037	1.620612412
**CLEC12B**	2.187917081	1.694381166	1.77E-12	2.187917081	0.521280556	9.28E-19	ECM_AFFILIATED	1.291277976	4.197196803
**CLEC14A**	11.20403881	8.897483098	0	11.20403881	10.16789028	2.92E-13	ECM_AFFILIATED	1.259236874	1.101903984
**CLEC17A**	3.092544942	2.763759048	9.46E-05	3.092544942	2.033172222	2.79E-07	ECM_AFFILIATED	1.118963299	1.521044262
**CLEC18A**	10.44670912	4.513542726	0	10.44670912	8.084451389	1.02E-14	ECM_AFFILIATED	2.314525364	1.292197654
**CLEC18B**	10.47349022	3.95675984	0	10.47349022	9.261241667	2.99E-06	ECM_AFFILIATED	2.646986585	1.130894819
**CLEC18C**	9.77235539	2.916698976	0	9.77235539	7.694377778	5.39E-10	ECM_AFFILIATED	3.350484733	1.270064412
**CLEC1A**	8.086524212	6.162316353	1.90E-226	8.086524212	7.058401389	6.94E-16	ECM_AFFILIATED	1.312253988	1.145659444
**CLEC1B**	1.946277944	1.415200739	2.52E-13	1.946277944	0.589652778	7.51E-21	ECM_AFFILIATED	1.375266342	3.300718689
**CLEC2B**	8.837280597	8.242477576	3.99E-19	8.837280597	6.944370833	6.47E-36	ECM_AFFILIATED	1.072163135	1.272581895
**CLEC2D**	8.659300829	8.181053125	1.06E-18	8.659300829	6.625101389	2.96E-33	ECM_AFFILIATED	1.058457963	1.307044273
**CLEC4A**	6.794940464	6.12701686	1.64E-36	6.794940464	5.136445833	1.99E-32	ECM_AFFILIATED	1.109012856	1.322887593
**CLEC4D**	2.904792371	2.252958499	2.53E-17	2.904792371	2.253077778	2.63E-05	ECM_AFFILIATED	1.289323515	1.289255258
**CLEC4E**	6.874488723	5.286163302	7.69E-87	6.874488723	5.905483333	2.01E-09	ECM_AFFILIATED	1.300468474	1.164085704
**CLEC4F**	3.911364179	3.249367684	4.22E-17	3.911364179	2.705805556	2.02E-09	ECM_AFFILIATED	1.203730867	1.44554518
**CLEC5A**	6.949000332	6.184372762	1.07E-16	6.949000332	4.924469444	1.81E-24	ECM_AFFILIATED	1.123638661	1.41111655
**CLEC7A**	8.473397015	7.752143338	2.13E-22	8.473397015	6.350652778	7.90E-32	ECM_AFFILIATED	1.09303926	1.33425607
**CLEC9A**	5.180434494	3.391493402	4.33E-102	5.180434494	4.116405556	1.84E-08	ECM_AFFILIATED	1.527478866	1.258484963
**COL15A1**	11.74691277	10.45141803	1.63E-44	11.74691277	10.40918194	2.00E-15	COLLAGENS	1.123953968	1.128514501
**COL15A1**	11.74691277	10.45141803	1.63E-44	11.74691277	10.40918194	2.00E-15	BASEMENT_MEMBRANES	1.123953968	1.128514501
**COL21A1**	8.031513433	6.099536518	9.35E-99	8.031513433	6.363894444	1.04E-13	COLLAGENS	1.316741593	1.262043785
**COL23A1**	11.58185871	6.192223163	0	11.58185871	6.686234722	2.48E-45	COLLAGENS	1.870387808	1.732194454
**COL27A1**	10.85414328	9.480106408	5.67E-78	10.85414328	9.429201389	8.62E-17	COLLAGENS	1.144938972	1.151120104
**COL4A1**	15.16350597	13.08107084	1.00E-151	15.16350597	13.50309444	3.25E-29	COLLAGENS	1.159194546	1.12296526
**COL4A1**	15.16350597	13.08107084	1.00E-151	15.16350597	13.50309444	3.25E-29	BASEMENT_MEMBRANES	1.159194546	1.12296526
**COL4A2**	15.0285131	13.3373902	1.26E-106	15.0285131	13.69891944	9.70E-25	COLLAGENS	1.126795638	1.097058287
**COL4A2**	15.0285131	13.3373902	1.26E-106	15.0285131	13.69891944	9.70E-25	BASEMENT_MEMBRANES	1.126795638	1.097058287
**COL5A3**	9.718981426	9.554075074	0.025700093	9.718981426	6.625777778	2.61E-47	COLLAGENS	1.017260316	1.466843856
**COL8A1**	11.37484776	9.731375528	1.20E-48	11.37484776	9.448852778	7.39E-24	COLLAGENS	1.168883857	1.203833738
**CRLF3**	9.199522056	9.065529698	0.000262257	9.199522056	8.397855556	4.87E-25	SECRETED_FACTORS	1.014780422	1.095460858
**CSF1**	11.19209022	10.56072784	4.84E-32	11.19209022	10.33311944	1.95E-16	SECRETED_FACTORS	1.059783983	1.083127924
**CSPG4**	11.92532305	10.2142164	1.41E-94	11.92532305	9.533372222	5.18E-36	ECM_AFFILIATED	1.167522068	1.25090291
**CST7**	8.298978939	6.979783879	5.99E-57	8.298978939	5.5285875	2.41E-35	ECM_REGULATORS	1.189002279	1.501102938
**CTSD**	15.87999917	15.04259047	7.74E-87	15.87999917	15.61078472	0.005256144	ECM_REGULATORS	1.055669182	1.017245414
**CTSL**	12.90206053	11.76380357	8.50E-108	12.90206053	12.70466528	0.002378692	ECM_REGULATORS	1.096759263	1.015537226
**CTSS**	12.03344925	11.4009428	2.12E-22	12.03344925	10.33526667	4.05E-29	ECM_REGULATORS	1.055478434	1.164309509
**CTSW**	8.191033831	6.880932485	1.19E-52	8.191033831	5.523063889	4.42E-34	ECM_REGULATORS	1.190395902	1.483059765
**CTSZ**	13.28780912	12.78600574	2.33E-27	13.28780912	12.23794306	1.24E-24	ECM_REGULATORS	1.039246297	1.085787788
**CX3CL1**	12.84839668	10.45128073	1.10E-169	12.84839668	12.05495	2.20E-13	SECRETED_FACTORS	1.229360976	1.06581916
**CXCL10**	9.247189552	8.42873144	3.74E-13	9.247189552	6.565777778	1.25E-28	SECRETED_FACTORS	1.097103356	1.4083921
**CXCL11**	7.082460199	6.55354109	1.95E-06	7.082460199	4.349088889	4.15E-28	SECRETED_FACTORS	1.080707377	1.628492859

**CXCL2**	7.289367993	7.062526151	0.035299681	7.289367993	6.398533333	6.57E-05	SECRETED_FACTORS	1.03211908	1.139224821
**CXCL9**	10.32818624	8.957809945	3.15E-28	10.32818624	7.325129167	6.46E-31	SECRETED_FACTORS	1.152981175	1.409966432
**DDX26B**	8.471691874	8.186677101	1.43E-07	8.471691874	7.020068056	1.46E-26	ECM_GLYCOPROTEINS	1.034814464	1.206782015
**DMP1**	1.851439303	1.433549018	2.10E-10	1.851439303	0.44505	1.48E-13	ECM_GLYCOPROTEINS	1.291507496	4.160070337
**EBI3**	7.037408292	5.996347445	1.73E-55	7.037408292	4.940815278	4.92E-34	SECRETED_FACTORS	1.173615831	1.42434151
**EDIL3**	11.62819337	9.137124229	8.40E-156	11.62819337	10.78246111	7.62E-07	ECM_GLYCOPROTEINS	1.272631637	1.07843592
**EFEMP2**	11.76636517	10.33536263	1.48E-118	11.76636517	10.96328056	2.74E-15	ECM_GLYCOPROTEINS	1.138456927	1.073252218
**EGFL7**	10.74446617	9.72576932	4.31E-74	10.74446617	9.981395833	1.11E-07	SECRETED_FACTORS	1.104742033	1.076449261
**EGFLAM**	8.492099668	7.633638376	8.41E-32	8.492099668	7.380697222	2.12E-12	ECM_GLYCOPROTEINS	1.112457684	1.150582311
**EGLN1**	12.24558972	11.13169949	2.26E-283	12.24558972	11.936975	2.88E-07	ECM_REGULATORS	1.100064705	1.025853679
**EGLN3**	13.47001874	9.356348406	0	13.47001874	9.729830556	2.75E-46	ECM_REGULATORS	1.43966622	1.384404247
**EPO**	4.640887894	1.997428822	3.08E-215	4.640887894	2.528947222	5.32E-07	SECRETED_FACTORS	2.323430924	1.835106661
**ESM1**	12.42546086	7.370375887	0	12.42546086	9.05735	5.60E-45	PROTEOGLYCANS	1.685865287	1.371864934
**F2**	3.856571144	2.012606461	2.16E-36	3.856571144	1.697127778	2.81E-08	ECM_REGULATORS	1.916207276	2.272410596
**FASLG**	5.689646269	4.107513714	1.06E-80	5.689646269	2.872016667	1.03E-33	SECRETED_FACTORS	1.385180103	1.98106311
**FBLN7**	7.526677944	7.268946263	0.000416672	7.526677944	5.9656875	1.97E-16	ECM_GLYCOPROTEINS	1.03545654	1.26166145
**FBN1**	11.54930315	11.18550923	4.21E-06	11.54930315	11.0740375	0.000417453	ECM_GLYCOPROTEINS	1.03252368	1.042917107
**FCN1**	8.221015257	6.275952259	2.65E-98	8.221015257	7.165208333	1.11E-10	ECM_AFFILIATED	1.309923167	1.147351881
**FCN3**	9.0575733	5.495804307	0	9.0575733	7.607698611	4.80E-11	ECM_AFFILIATED	1.648088759	1.190579933
**FGF11**	9.851438806	7.539408404	9.17E-139	9.851438806	7.818938889	2.04E-22	SECRETED_FACTORS	1.306659393	1.259945748
**FGF14**	6.73802272	5.464790762	1.24E-34	6.73802272	6.354980556	0.020849244	SECRETED_FACTORS	1.232988236	1.060274325
**FGF20**	2.359872968	1.855644531	4.17E-10	2.359872968	1.197641667	1.32E-12	SECRETED_FACTORS	1.271726847	1.970433256
**FGG**	5.71429005	3.216749229	1.22E-34	5.71429005	3.605713889	5.53E-05	ECM_GLYCOPROTEINS	1.776417632	1.584787431
**FN1**	15.47287214	15.22713282	0.012573832	15.47287214	13.98961528	3.05E-18	ECM_GLYCOPROTEINS	1.016138253	1.106025565
**GPC6**	10.87708308	8.242906746	8.06E-180	10.87708308	10.22113333	3.40E-05	ECM_AFFILIATED	1.319568863	1.064175834
**HAPLN1**	8.064862521	5.195900929	2.95E-119	8.064862521	4.250297222	2.42E-32	PROTEOGLYCANS	1.55215864	1.897482011
**HAPLN2**	3.421747595	2.633874831	7.36E-15	3.421747595	2.686426389	1.16E-05	PROTEOGLYCANS	1.299130678	1.273717236
**HAPLN4**	3.954277446	3.642159027	0.002661679	3.954277446	1.940705556	1.63E-15	PROTEOGLYCANS	1.085695989	2.037546311
**HMCN1**	9.900076783	8.678227302	5.03E-39	9.900076783	8.940238889	3.50E-10	BASEMENT_MEMBRANES	1.140794824	1.107361549
**HMCN1**	9.900076783	8.678227302	5.03E-39	9.900076783	8.940238889	3.50E-10	ECM_GLYCOPROTEINS	1.140794824	1.107361549
**HSPG2**	14.47044511	13.32827609	5.85E-76	14.47044511	13.65412222	1.41E-09	BASEMENT_MEMBRANES	1.08569518	1.059785819
**HSPG2**	14.47044511	13.32827609	5.85E-76	14.47044511	13.65412222	1.41E-09	PROTEOGLYCANS	1.08569518	1.059785819
**HTRA1**	13.02821211	11.75354396	3.26E-59	13.02821211	12.34995139	4.74E-09	ECM_REGULATORS	1.108449685	1.054920112
**HTRA4**	4.143048425	3.286020429	3.91E-27	4.143048425	1.068151389	5.69E-34	ECM_REGULATORS	1.260810307	3.878709018
**IGFBP1**	5.747076285	2.801140298	2.30E-95	5.747076285	4.9471875	0.033226399	ECM_GLYCOPROTEINS	2.051691695	1.161685561
**IGFBP3**	15.67271443	12.20539058	0	15.67271443	12.62461111	8.80E-41	ECM_GLYCOPROTEINS	1.284081351	1.241441363
**IGFBP4**	14.95431907	13.33754914	1.13E-106	14.95431907	14.21862361	6.57E-12	ECM_GLYCOPROTEINS	1.121219417	1.051741679
**LAMA4**	12.18136949	10.8735146	5.38E-74	12.18136949	9.671184722	8.06E-46	ECM_GLYCOPROTEINS	1.120278947	1.259552975
**LAMA4**	12.18136949	10.8735146	5.38E-74	12.18136949	9.671184722	8.06E-46	BASEMENT_MEMBRANES	1.120278947	1.259552975
**LAMA5**	12.72284179	12.27180755	6.43E-14	12.72284179	12.16372222	1.80E-07	ECM_GLYCOPROTEINS	1.036753693	1.045966157
**LAMA5**	12.72284179	12.27180755	6.43E-14	12.72284179	12.16372222	1.80E-07	BASEMENT_MEMBRANES	1.036753693	1.045966157
**LAMB2**	13.70115771	12.62592665	1.29E-93	13.70115771	13.4987125	0.00943834	ECM_GLYCOPROTEINS	1.085160566	1.014997372
**LAMB2**	13.70115771	12.62592665	1.29E-93	13.70115771	13.4987125	0.00943834	BASEMENT_MEMBRANES	1.085160566	1.014997372
**LAMC1**	13.51508557	12.74272957	2.79E-43	13.51508557	12.96420139	9.38E-11	BASEMENT_MEMBRANES	1.060611504	1.04249272
**LAMC1**	13.51508557	12.74272957	2.79E-43	13.51508557	12.96420139	9.38E-11	ECM_GLYCOPROTEINS	1.060611504	1.04249272
**LGALS12**	5.359220896	2.965744046	4.38E-133	5.359220896	2.600404167	2.07E-17	ECM_AFFILIATED	1.807040936	2.060918439

**LGALS2**	9.935327695	4.971912606	0	9.935327695	8.854563889	0.000145832	ECM_AFFILIATED	1.998290896	1.12205726
**LGALS3**	12.53880862	12.37218833	0.021097249	12.53880862	11.71294167	2.81E-10	ECM_AFFILIATED	1.013467326	1.070508928
**LGALS4**	6.815552239	5.61655076	1.03E-11	6.815552239	5.825394444	0.007070449	ECM_AFFILIATED	1.213476479	1.169972661
**LGALS8**	11.68758109	11.27448579	6.65E-28	11.68758109	11.47609028	1.00E-05	ECM_AFFILIATED	1.036639836	1.018428821
**LGI4**	9.742774793	6.763202956	8.69E-286	9.742774793	6.834722222	3.86E-22	ECM_GLYCOPROTEINS	1.440556325	1.425482189
**LOX**	12.20320498	9.413232496	1.37E-231	12.20320498	8.994881944	3.08E-29	ECM_REGULATORS	1.296388353	1.356683173
**LOXL2**	11.38397828	10.14151548	3.28E-65	11.38397828	8.782736111	1.02E-36	ECM_REGULATORS	1.122512538	1.296176742
**MASP1**	10.01143997	6.013994785	4.15E-169	10.01143997	8.433825	6.64E-12	ECM_REGULATORS	1.664690497	1.187058063
**MATN1**	2.705213101	2.397074958	3.79E-07	2.705213101	1.476141667	5.54E-16	ECM_GLYCOPROTEINS	1.128547563	1.832624309
**MMRN1**	8.317568325	6.661839073	1.22E-69	8.317568325	7.528338889	0.000756579	ECM_GLYCOPROTEINS	1.248539365	1.104834472
**MUC12**	5.264200663	4.96823485	0.020242154	5.264200663	2.091136111	3.78E-28	ECM_AFFILIATED	1.059571623	2.517387862
**MUC3A**	8.593650415	6.251572804	5.14E-57	8.593650415	5.777555556	1.94E-28	ECM_AFFILIATED	1.374638141	1.48741978
**NID1**	12.79713947	11.34216783	2.77E-115	12.79713947	11.5376125	1.25E-21	BASEMENT_MEMBRANES	1.128279854	1.109167037
**NID1**	12.79713947	11.34216783	2.77E-115	12.79713947	11.5376125	1.25E-21	ECM_GLYCOPROTEINS	1.128279854	1.109167037
**NID2**	10.62164826	9.157650623	1.42E-73	10.62164826	9.872540278	2.13E-07	BASEMENT_MEMBRANES	1.159866072	1.075877936
**NID2**	10.62164826	9.157650623	1.42E-73	10.62164826	9.872540278	2.13E-07	ECM_GLYCOPROTEINS	1.159866072	1.075877936
**OIT3**	5.799255556	3.156478009	3.52E-163	5.799255556	4.537640278	2.68E-07	ECM_GLYCOPROTEINS	1.837255175	1.278033339
**OTOG**	2.711248756	1.158646569	8.74E-89	2.711248756	1.556794444	5.54E-10	ECM_GLYCOPROTEINS	2.340013624	1.741558602
**P4HA1**	12.23105854	11.09278501	3.21E-168	12.23105854	10.76867639	3.56E-38	ECM_REGULATORS	1.102613864	1.135799619
**P4HA2**	11.81924876	10.63943168	8.32E-85	11.81924876	10.53157778	2.04E-24	ECM_REGULATORS	1.110890986	1.122267623
**P4HA3**	7.0343199	6.413283868	9.44E-15	7.0343199	4.990619444	2.70E-23	ECM_REGULATORS	1.096835887	1.409508374
**PAMR1**	8.908585738	7.58100814	2.36E-54	8.908585738	8.296740278	9.82E-06	ECM_REGULATORS	1.175118873	1.073745283
**PCSK5**	9.35573665	8.304611201	2.44E-48	9.35573665	8.997338889	0.005244554	ECM_REGULATORS	1.126571302	1.039833751
**PCSK6**	11.4643592	9.095109544	1.33E-139	11.4643592	8.997893056	7.37E-24	ECM_REGULATORS	1.2604971	1.274115966
**PLOD1**	13.10309718	12.50637365	4.45E-49	13.10309718	11.99509861	1.14E-23	ECM_REGULATORS	1.047713554	1.092370943
**PLOD2**	13.07497297	11.00814783	1.16E-166	13.07497297	11.69973472	3.32E-22	ECM_REGULATORS	1.187754123	1.117544396
**PLOD3**	11.87019652	11.66421617	3.72E-06	11.87019652	10.66856667	7.19E-38	ECM_REGULATORS	1.017659167	1.112632736
**PLXDC1**	10.26218242	8.791182675	1.08E-141	10.26218242	7.847458333	1.59E-39	ECM_AFFILIATED	1.167326718	1.307707793
**PLXDC2**	11.99356633	10.79286649	3.54E-71	11.99356633	11.61820278	0.00090517	ECM_AFFILIATED	1.111249393	1.032308229
**PLXNA2**	11.08723765	10.54093009	5.96E-24	11.08723765	10.37407083	2.85E-11	ECM_AFFILIATED	1.051827263	1.068745127
**PLXND1**	12.88422272	11.84135469	2.83E-108	12.88422272	11.50862083	4.99E-30	ECM_AFFILIATED	1.088069994	1.119527953
**PXDN**	11.85385108	11.2128316	4.57E-17	11.85385108	10.31253056	1.65E-24	ECM_GLYCOPROTEINS	1.057168386	1.149460941
**PZP**	3.459644279	2.539882844	6.35E-32	3.459644279	2.898555556	2.83E-05	ECM_REGULATORS	1.362127504	1.193575287
**REG1A**	7.856792206	2.469637468	2.10E-205	7.856792206	5.193655556	7.61E-09	ECM_AFFILIATED	3.181354473	1.512767283
**REG1B**	2.53455937	0.831605184	3.28E-60	2.53455937	0.928422222	2.80E-07	ECM_AFFILIATED	3.047791692	2.729964136
**SEMA3F**	11.76249718	10.55298595	2.03E-56	11.76249718	10.77075694	2.59E-19	ECM_AFFILIATED	1.114613176	1.092077116
**SEMA5B**	11.68887048	6.513178062	0	11.68887048	8.360398611	6.67E-41	ECM_AFFILIATED	1.794649305	1.398123585
**SEMA6A**	11.54077181	9.413355469	3.02E-146	11.54077181	9.825670833	3.58E-25	ECM_AFFILIATED	1.225999788	1.174553066
**SEMA6B**	9.86630995	8.737020281	7.56E-76	9.86630995	8.392631944	1.82E-24	ECM_AFFILIATED	1.129253411	1.175591878
**SEMA6C**	8.782595854	8.339472382	6.83E-16	8.782595854	8.354565278	2.86E-05	ECM_AFFILIATED	1.053135672	1.051233136
**SERPINA1**	14.95360498	11.61734368	1.15E-134	14.95360498	13.89255833	3.90E-07	ECM_REGULATORS	1.287179358	1.07637518
**SERPINB1**	11.68110746	11.54642448	0.032431084	11.68110746	11.03191667	3.74E-26	ECM_REGULATORS	1.011664475	1.058846601
**SERPINB6**	12.39492355	11.84919963	4.50E-45	12.39492355	11.90864444	6.72E-22	ECM_REGULATORS	1.046055762	1.040834127
**SERPINB9**	10.02796799	9.250079476	1.57E-44	10.02796799	8.781790278	5.15E-28	ECM_REGULATORS	1.084095333	1.141904746
**SERPINE1**	12.42297562	10.71683257	2.60E-75	12.42297562	10.64312083	6.34E-14	ECM_REGULATORS	1.159202175	1.167230535
**SERPINE2**	11.74547363	10.61310534	3.43E-32	11.74547363	10.97230556	4.54E-06	ECM_REGULATORS	1.106695284	1.070465416

**SERPINE3**	1.463690713	1.085625179	3.49E-15	1.463690713	0.815306944	6.06E-07	ECM_REGULATORS	1.348246836	1.795263395
**SERPINF2**	12.10498557	7.33069281	0	12.10498557	11.15714306	4.23E-05	ECM_REGULATORS	1.651274427	1.084953873
**SERPING1**	14.13853847	12.81372791	4.80E-70	14.13853847	13.73928333	0.004964138	ECM_REGULATORS	1.10338994	1.029059386
**SERPINH1**	12.85610647	12.70051871	0.00815546	12.85610647	11.50330972	1.10E-32	ECM_REGULATORS	1.012250504	1.117600654
**SERPINI1**	9.692102819	7.887681936	9.88E-156	9.692102819	9.254679167	0.000312622	ECM_REGULATORS	1.228764407	1.047265134
**SNED1**	9.818390713	9.178406915	9.54E-23	9.818390713	9.476165278	0.002546701	ECM_GLYCOPROTEINS	1.069727111	1.036114338
**SPARC**	16.23162471	15.12703417	2.49E-59	16.23162471	14.50161667	1.67E-36	ECM_GLYCOPROTEINS	1.073020959	1.119297599
**SPARCL1**	13.80186833	12.32108392	4.23E-57	13.80186833	12.48785972	2.98E-16	ECM_GLYCOPROTEINS	1.120182966	1.105222883
**SPON2**	10.63698773	10.24074302	2.76E-05	10.63698773	9.052461111	3.47E-14	ECM_GLYCOPROTEINS	1.038692965	1.175038213
**SRGN**	12.06489254	10.89604364	8.04E-79	12.06489254	11.43566111	4.67E-09	PROTEOGLYCANS	1.10727278	1.055023616
**SULF1**	11.57327761	11.0555136	5.13E-07	11.57327761	10.50840694	6.92E-10	ECM_REGULATORS	1.046833104	1.101335119
**SULF2**	12.26121012	11.74720074	1.64E-13	12.26121012	11.48369306	1.91E-08	ECM_REGULATORS	1.043755903	1.067706186
**TGFBI**	13.9308466	12.22635787	1.67E-91	13.9308466	11.42486806	2.10E-17	ECM_GLYCOPROTEINS	1.139410997	1.2193442
**TGM2**	13.77264046	11.97478609	1.83E-117	13.77264046	12.59867222	6.94E-15	ECM_REGULATORS	1.150136659	1.093181902
**TIMP1**	13.36011841	13.0633727	5.15E-05	13.36011841	11.4754875	1.42E-26	ECM_REGULATORS	1.022715857	1.16423101
**TLL1**	8.683251575	5.422040308	2.28E-277	8.683251575	7.693520833	2.50E-08	ECM_REGULATORS	1.601473077	1.128644708
**TNFAIP6**	9.24041675	6.325536518	7.85E-180	9.24041675	3.766816667	1.36E-47	ECM_GLYCOPROTEINS	1.460811541	2.453110296
**TNN**	5.074218408	4.777913081	0.001873261	5.074218408	4.333523611	0.000530431	ECM_GLYCOPROTEINS	1.062015638	1.170922063
**VCAN**	12.8368869	11.58204903	8.77E-38	12.8368869	11.09440417	2.74E-15	PROTEOGLYCANS	1.10834334	1.157059605
**WFIKKN1**	6.173001824	4.942840678	3.86E-73	6.173001824	5.469561111	0.000335116	SECRETED_FACTORS	1.248877362	1.128610084
**WNT2B**	7.899565174	7.570306894	3.12E-06	7.899565174	7.193263889	0.000178062	SECRETED_FACTORS	1.043493386	1.098189264
**VWA1**	12.19261376	11.52317063	2.82E-29	12.19261376	11.13334583	3.24E-16	ECM_GLYCOPROTEINS	1.058095394	1.095143719
**VWCE**	7.715001493	5.70891516	1.59E-112	7.715001493	5.624819444	2.37E-16	ECM_GLYCOPROTEINS	1.351395366	1.371599848
**VWF**	14.6267602	12.07067299	0	14.6267602	11.87630417	3.86E-37	ECM_GLYCOPROTEINS	1.211760124	1.231591916
**XCL1**	4.892048425	4.142711434	8.00E-19	4.892048425	3.118611111	6.85E-19	SECRETED_FACTORS	1.180880808	1.568662539
**XCL2**	4.622433167	3.443631978	1.40E-51	4.622433167	2.592145833	1.63E-23	SECRETED_FACTORS	1.342313347	1.783245799
**ZP1**	2.464280929	2.149809407	2.13E-05	2.464280929	0.528861111	3.20E-13	ECM_GLYCOPROTEINS	1.146278792	4.659599424

Kidney Papillary Cell Carcinoma (KIRP)

Signature genes	Expression in tumor	Expression in rest of Pan-Cancer	P-value	Expression in tumor	Expression in normal tissue	P-value	Matrisome category	Fold change vs. Pan-Cancer	Fold change vs. normal
**ADAM10**	12.59589221	12.00752005	3.53E-45	12.59589221	12.0682375	6.47E-05	ECM_REGULATORS	1.049000307	1.043722599
**ADAM18**	0.631162928	0.293273311	5.56E-12	0.631162928	0	0.001473833	ECM_REGULATORS	2.152132175	Inf
**ADAM28**	8.671511526	7.966519172	3.42E-10	8.671511526	7.778990625	0.000192492	ECM_REGULATORS	1.088494403	1.114734796
**ADAMTS10**	7.990943614	7.606247466	7.36E-06	7.990943614	7.45928125	0.031531271	ECM_REGULATORS	1.050576339	1.071275281
**ADAMTS13**	8.100636449	7.026120957	1.58E-45	8.100636449	7.452178125	0.002299593	ECM_REGULATORS	1.152931539	1.087015945
**ADAMTS3**	7.629615576	5.729295809	1.55E-70	7.629615576	6.7797625	0.000255731	ECM_REGULATORS	1.331684701	1.125351452
**ADAMTS9**	11.19361215	8.839440287	4.49E-119	11.19361215	10.38225	9.90E-07	ECM_REGULATORS	1.266325897	1.078148971
**ANXA1**	12.94412586	12.60711304	0.005055428	12.94412586	11.0567875	3.89E-15	ECM_AFFILIATED	1.026731958	1.170695001
**ANXA13**	8.637501558	3.573841375	1.41E-175	8.637501558	6.5670375	0.000211675	ECM_AFFILIATED	2.416867637	1.31528129
**ANXA4**	13.65363863	11.74772896	2.72E-158	13.65363863	12.71661563	1.87E-10	ECM_AFFILIATED	1.162236435	1.073684936
**BDNF**	5.430978816	5.149569943	0.018053609	5.430978816	4.694178125	0.043626365	SECRETED_FACTORS	1.054647063	1.156960531
**BMP1**	10.8820514	10.65086269	0.00019105	10.8820514	9.26795	8.69E-16	ECM_REGULATORS	1.021706102	1.174159485
**C1QTNF3**	9.394130218	7.836027745	6.59E-46	9.394130218	8.4200125	0.017124323	ECM_AFFILIATED	1.198838305	1.115690769
**C1QTNF5**	10.38591028	9.229578758	2.29E-35	10.38591028	9.816803125	0.005306626	ECM_AFFILIATED	1.125285406	1.057972758
**C1QTNF8**	0.452791589	0.335373638	0.016336358	0.452791589	0.118353125	0.02004039	ECM_AFFILIATED	1.350110854	3.825767919
**CCL18**	7.961552025	7.110148818	7.71E-06	7.961552025	2.835803125	3.83E-13	SECRETED_FACTORS	1.11974478	2.807512255
**CCL28**	8.923380062	7.846971484	1.35E-15	8.923380062	7.83704375	0.005176882	SECRETED_FACTORS	1.137175034	1.138615573
**CELA1**	0.900054517	0.6377678	3.92E-06	0.900054517	0.119390625	3.38E-06	ECM_REGULATORS	1.411257385	7.538736958
**CLCF1**	9.926199065	7.578864274	1.40E-119	9.926199065	8.69990625	1.94E-09	SECRETED_FACTORS	1.309721181	1.140954716
**CLEC17A**	3.426080374	2.763759048	6.84E-09	3.426080374	2.38930625	0.000736231	ECM_AFFILIATED	1.239645105	1.43392266
**CLEC4E**	6.376849221	5.286163302	1.99E-23	6.376849221	5.307953125	0.000738535	ECM_AFFILIATED	1.206328457	1.201376325
**CLEC5A**	6.545141433	6.184372762	0.003972951	6.545141433	4.384225	1.49E-13	ECM_AFFILIATED	1.058335531	1.492884474
**COL23A1**	9.439427726	6.192223163	6.59E-139	9.439427726	7.159628125	5.54E-10	COLLAGENS	1.524400442	1.318424304
**COL27A1**	11.32645202	9.480106408	5.94E-77	11.32645202	10.31993438	4.35E-07	COLLAGENS	1.19476001	1.0975314
**COLQ**	6.8583081	6.26206762	3.24E-16	6.8583081	6.102709375	4.87E-05	ECM_GLYCOPROTEINS	1.095214634	1.12381365
**COLQ**	6.8583081	6.26206762	3.24E-16	6.8583081	6.102709375	4.87E-05	BASEMENT_MEMBRANES	1.095214634	1.12381365
**CRELD1**	10.86847944	10.22277786	2.38E-36	10.86847944	10.68195625	0.046787122	ECM_GLYCOPROTEINS	1.063163025	1.01746152
**CST3**	13.87110093	13.57421519	3.50E-06	13.87110093	13.13172188	7.83E-08	ECM_REGULATORS	1.021871301	1.056304807
**CTSA**	13.79110561	12.75228011	4.53E-85	13.79110561	13.5067625	0.030104231	ECM_REGULATORS	1.081461942	1.021051907
**CTSB**	15.5677704	15.22879197	3.28E-06	15.5677704	15.15025938	0.019058605	ECM_REGULATORS	1.02225905	1.027558012
**CTSC**	14.46493894	12.03806892	1.54E-172	14.46493894	13.08364063	2.13E-12	ECM_REGULATORS	1.201599612	1.105574462
**CTSD**	16.61061745	15.04259047	7.56E-159	16.61061745	16.03045938	0.001042485	ECM_REGULATORS	1.104239159	1.036190982
**CTSL**	13.04041807	11.76380357	2.53E-73	13.04041807	12.75044375	0.02995159	ECM_REGULATORS	1.108520556	1.022742292
**CTSZ**	13.5529757	12.78600574	8.38E-34	13.5529757	12.34570938	3.75E-11	ECM_REGULATORS	1.05998511	1.097788332
**CX3CL1**	12.6440215	10.45128073	9.06E-79	12.6440215	12.257825	0.004039731	SECRETED_FACTORS	1.209805938	1.031506119
**CXCL1**	8.110678816	6.955120429	1.07E-09	8.110678816	6.52434375	0.000271251	SECRETED_FACTORS	1.166144986	1.243140939
**CXCL5**	5.802856386	5.078675021	0.000185536	5.802856386	2.851815625	1.58E-06	SECRETED_FACTORS	1.142592578	2.034793672
**CXCL6**	8.086219938	4.379255152	1.74E-90	8.086219938	5.762940625	0.000134894	SECRETED_FACTORS	1.846482942	1.403141289
**EBI3**	6.608440187	5.996347445	8.49E-12	6.608440187	5.2003375	2.73E-08	SECRETED_FACTORS	1.102077598	1.270771404
**EGFL8**	8.128786916	7.67385114	2.91E-09	8.128786916	7.409	0.010091308	SECRETED_FACTORS	1.059283894	1.097150346
**EGLN3**	10.22908037	9.356348406	1.87E-12	10.22908037	9.4548625	0.002235524	ECM_REGULATORS	1.093276985	1.081885683

**ELFN2**	2.185737072	1.091319468	1.30E-34	2.185737072	1.1796125	4.48E-05	ECM_AFFILIATED	2.00283889	1.852928035
**ELSPBP1**	1.682143302	0.400242473	1.72E-80	1.682143302	0.52133125	0.003433874	ECM_GLYCOPROTEINS	4.20281059	3.226630481
**F12**	7.884756386	7.30649261	3.45E-05	7.884756386	5.290778125	1.72E-13	ECM_REGULATORS	1.079143826	1.490282941
**FBLN7**	8.16224891	7.268946263	2.95E-19	8.16224891	6.211996875	2.50E-09	ECM_GLYCOPROTEINS	1.122893005	1.313949294
**FGF17**	3.428017445	3.165921094	0.038448031	3.428017445	2.502115625	0.002310742	SECRETED_FACTORS	1.082786761	1.370047575
**FGF20**	2.082680685	1.855644531	0.038811808	2.082680685	1.110853125	0.000159748	SECRETED_FACTORS	1.122348947	1.874847933
**GPC4**	12.2659757	9.796160452	1.76E-86	12.2659757	11.7863625	0.021807793	ECM_AFFILIATED	1.252120743	1.040692215
**HTRA4**	4.644349844	3.286020429	2.83E-36	4.644349844	1.256496875	1.05E-14	ECM_REGULATORS	1.41336609	3.69626852
**IGFBP6**	11.63734174	8.715188514	8.09E-117	11.63734174	9.177053125	4.58E-11	ECM_GLYCOPROTEINS	1.335294323	1.268091356
**KCP**	10.93225171	5.632865192	0	10.93225171	10.00592813	0.000148395	ECM_GLYCOPROTEINS	1.940797683	1.092577478
**LAMA3**	10.62209564	9.84153712	5.36E-07	10.62209564	8.7836625	8.34E-09	BASEMENT_MEMBRANES	1.079312663	1.209301432
**LAMA3**	10.62209564	9.84153712	5.36E-07	10.62209564	8.7836625	8.34E-09	ECM_GLYCOPROTEINS	1.079312663	1.209301432
**LAMA5**	13.19917819	12.27180755	1.23E-29	13.19917819	12.87041563	0.018267389	ECM_GLYCOPROTEINS	1.075569197	1.025544052
**LAMA5**	13.19917819	12.27180755	1.23E-29	13.19917819	12.87041563	0.018267389	BASEMENT_MEMBRANES	1.075569197	1.025544052
**LAMB1**	14.51635919	12.08447157	4.07E-149	14.51635919	13.75882188	2.87E-05	ECM_GLYCOPROTEINS	1.201240709	1.055058298
**LAMB1**	14.51635919	12.08447157	4.07E-149	14.51635919	13.75882188	2.87E-05	BASEMENT_MEMBRANES	1.201240709	1.055058298
**LAMC1**	13.20527134	12.74272957	1.37E-09	13.20527134	12.56580313	3.12E-05	BASEMENT_MEMBRANES	1.036298484	1.050889562
**LAMC1**	13.20527134	12.74272957	1.37E-09	13.20527134	12.56580313	3.12E-05	ECM_GLYCOPROTEINS	1.036298484	1.050889562
**LGALS12**	4.018092835	2.965744046	1.95E-16	4.018092835	2.83313125	0.000474026	ECM_AFFILIATED	1.354834663	1.418251567
**MASP1**	10.18852461	6.013994785	2.30E-101	10.18852461	8.741159375	1.65E-05	ECM_REGULATORS	1.694135924	1.165580465
**MMP15**	11.68384143	10.78826829	1.85E-24	11.68384143	10.88892188	0.000396395	ECM_REGULATORS	1.083013615	1.073002595
**MMP24**	11.01330125	6.583126742	6.20E-264	11.01330125	10.3208875	0.002894253	ECM_REGULATORS	1.672959017	1.067088586
**MMP7**	13.07205826	7.939855923	1.91E-109	13.07205826	11.9315375	0.003654131	ECM_REGULATORS	1.646384819	1.09558875
**MUC12**	6.546148287	4.96823485	1.95E-19	6.546148287	2.312725	2.65E-13	ECM_AFFILIATED	1.317600412	2.830491427
**MUC22**	1.248452648	0.955345006	0.002749573	1.248452648	0.549109375	0.005760463	ECM_AFFILIATED	1.306808158	2.27359558
**MUC3A**	9.093922741	6.251572804	2.52E-45	9.093922741	5.70645625	8.46E-13	ECM_AFFILIATED	1.454661575	1.593619988
**NTN5**	4.013013084	3.722585082	0.001091724	4.013013084	2.834084375	3.69E-06	ECM_GLYCOPROTEINS	1.078017828	1.41598222
**NTN5**	4.013013084	3.722585082	0.001091724	4.013013084	2.834084375	3.69E-06	BASEMENT_MEMBRANES	1.078017828	1.41598222
**OGFOD2**	9.463765421	9.056172382	2.31E-27	9.463765421	9.24090625	0.020626991	ECM_REGULATORS	1.045007209	1.024116593
**OTOG**	3.096923988	1.158646569	4.64E-75	3.096923988	1.9328125	0.000268218	ECM_GLYCOPROTEINS	2.672880644	1.602288886
**OVGP1**	7.822942991	6.27086932	9.61E-60	7.822942991	6.4070375	4.85E-09	ECM_AFFILIATED	1.247505344	1.220992228
**P4HTM**	11.36993333	10.26899876	1.64E-48	11.36993333	10.97552813	5.70E-05	ECM_REGULATORS	1.107209534	1.035934964
**PAPLN**	12.23612492	8.998343095	2.61E-261	12.23612492	11.3163125	0.000120046	BASEMENT_MEMBRANES	1.359819779	1.081281992
**PAPLN**	12.23612492	8.998343095	2.61E-261	12.23612492	11.3163125	0.000120046	ECM_GLYCOPROTEINS	1.359819779	1.081281992
**PLOD2**	11.96637072	11.00814783	1.42E-21	11.96637072	11.13271563	9.01E-07	ECM_REGULATORS	1.087046695	1.074883355
**PLOD3**	12.17843053	11.66421617	2.38E-17	12.17843053	11.0299875	4.35E-17	ECM_REGULATORS	1.044084776	1.104120066
**PLXDC2**	12.02954019	10.79286649	1.23E-41	12.02954019	11.33408125	2.39E-05	ECM_AFFILIATED	1.114582507	1.061359975
**PXDN**	12.14663178	11.2128316	3.33E-19	12.14663178	10.14875625	5.75E-15	ECM_GLYCOPROTEINS	1.083279604	1.19685915
**REG1B**	1.742262617	0.831605184	5.86E-11	1.742262617	0.757484375	0.016708556	ECM_AFFILIATED	2.095059832	2.300064099
**REG3G**	4.680821807	0.752465266	1.29E-247	4.680821807	3.0763375	0.004342932	ECM_AFFILIATED	6.220648338	1.521556658
**SEMA4F**	9.016138629	8.657883224	1.70E-09	9.016138629	8.58793125	0.000704471	ECM_AFFILIATED	1.041379099	1.049861529
**SEMA6A**	11.34110685	9.413355469	3.39E-67	11.34110685	10.11896563	6.81E-09	ECM_AFFILIATED	1.204788972	1.120777288
**SERPINE2**	13.28343832	10.61310534	1.40E-90	13.28343832	10.9273	1.87E-11	ECM_REGULATORS	1.25160713	1.215619441
**SLPI**	10.48679595	9.820370365	0.002815295	10.48679595	9.2418625	0.032658316	ECM_REGULATORS	1.067861553	1.134705905
**SPON2**	12.26753707	10.24074302	8.69E-55	12.26753707	9.468040625	2.06E-12	ECM_GLYCOPROTEINS	1.197914746	1.295678542
**SPP1**	17.23932056	11.59797863	4.07E-242	17.23932056	15.96070938	1.28E-06	ECM_GLYCOPROTEINS	1.486407339	1.080109922

**SULF2**	12.89173178	11.74720074	2.95E-33	12.89173178	11.66308125	5.48E-08	ECM_REGULATORS	1.097430108	1.105345277
**TGM2**	12.99723614	11.97478609	3.57E-22	12.99723614	12.37251563	0.022808045	ECM_REGULATORS	1.085383575	1.050492602
**THBS3**	10.28046885	9.860752133	6.09E-13	10.28046885	9.2702	1.42E-10	ECM_GLYCOPROTEINS	1.042564371	1.108980264
**TIMP1**	13.27896885	13.0633727	0.029914409	13.27896885	11.24769063	1.89E-18	ECM_REGULATORS	1.016503866	1.180595136
**TIMP2**	13.60509938	13.32464488	0.000461695	13.60509938	13.13305938	0.006965581	ECM_REGULATORS	1.021047803	1.035942882
**TINAGL1**	13.33965794	10.38895489	8.82E-149	13.33965794	12.59830938	8.26E-06	ECM_GLYCOPROTEINS	1.284023089	1.058845084
**TLL2**	5.640549533	4.952078748	2.45E-11	5.640549533	4.697865625	0.000619081	ECM_REGULATORS	1.139026623	1.200662169
**TNFAIP6**	7.853962928	6.325536518	7.09E-30	7.853962928	3.591609375	2.15E-14	ECM_GLYCOPROTEINS	1.241627948	2.186753098
**VCAN**	13.02272741	11.58204903	3.08E-27	13.02272741	10.35019375	4.99E-11	PROTEOGLYCANS	1.124388904	1.258210979
**WNT2B**	7.807886916	7.570306894	0.012792755	7.807886916	7.264059375	0.038082634	SECRETED_FACTORS	1.031383143	1.074865514
**WNT5A**	9.663325545	9.166653262	0.000142986	9.663325545	8.2600125	1.46E-06	SECRETED_FACTORS	1.05418251	1.169892363
**VWDE**	4.841403427	4.296767061	0.000793592	4.841403427	3.898359375	0.035043347	ECM_GLYCOPROTEINS	1.12675492	1.241907931

Acute Myeloid Leukemia (LAML)

Signature genes	Expression in tumor	Expression in rest of Pan-Cancer	P-value	Expression in tumor	Expression in normal tissue	P-value	Matrisome category	Fold change vs. Pan-Cancer	Fold change vs. normal
**ADAM10**	12.9438526	12.04652004	1.47E-57	12.9438526	7.340137247	0	ECM_REGULATORS	1.074488944	1.763434683
**ADAM11**	6.272948555	4.996735932	2.39E-18	6.272948555	5.243065182	5.57E-161	ECM_REGULATORS	1.255409259	1.196427726
**ADAM17**	11.50581503	10.18508517	1.01E-52	11.50581503	3.687994332	2.06E-195	ECM_REGULATORS	1.129672933	3.119802796
**ADAM20**	4.248837572	1.684467879	2.68E-206	4.248837572	2.451197976	7.27E-67	ECM_REGULATORS	2.522361884	1.733371851
**ADAM28**	11.17229538	7.985893853	3.65E-102	11.17229538	0.117832389	2.43E-31	ECM_REGULATORS	1.399003741	94.81514805
**ADAM8**	10.93917803	8.844270584	3.33E-54	10.93917803	2.167739676	1.47E-145	ECM_REGULATORS	1.236866051	5.04635227
**ADAMTS10**	8.554260694	7.648778621	8.98E-15	8.554260694	3.57282753	1.39E-112	ECM_REGULATORS	1.11838257	2.394255144
**ADAMTS6**	6.716960694	5.754721194	1.65E-12	6.716960694	0.021462348	3.64E-81	ECM_REGULATORS	1.167208709	312.9648554
**ADAMTSL4**	11.09090289	8.801570118	5.56E-63	11.09090289	5.204299595	6.68E-14	ECM_REGULATORS	1.260105043	2.131103847
**AMH**	5.666390173	4.836136843	7.71E-06	5.666390173	1.710121457	1.22E-28	SECRETED_FACTORS	1.171676972	3.313443118
**ANGPT1**	10.60055145	7.076903742	9.05E-114	10.60055145	1.965484615	3.04E-30	SECRETED_FACTORS	1.497908101	5.393352541
**ANGPTL3**	4.175817919	2.358567355	9.72E-14	4.175817919	1.401635628	8.80E-12	SECRETED_FACTORS	1.770489153	2.979246415
**ANXA1**	14.14795318	12.5695958	1.88E-23	14.14795318	0.036306883	2.31E-82	ECM_AFFILIATED	1.125569462	389.6768924
**AREG**	7.550857225	7.014058251	0.030869553	7.550857225	0.227155061	2.04E-05	SECRETED_FACTORS	1.076531867	33.24098174
**ARTN**	6.9857	5.485786184	1.70E-19	6.9857	0.261886235	8.60E-24	SECRETED_FACTORS	1.273418206	26.67455968
**ASTL**	2.554580347	1.930034691	1.17E-07	2.554580347	1.904823482	7.94E-47	ECM_REGULATORS	1.323592969	1.341111327
**BMP8B**	8.121893064	7.790625965	0.011475905	8.121893064	3.383938057	9.33E-70	SECRETED_FACTORS	1.042521243	2.400130537
**CCL1**	2.355576879	0.606005333	3.02E-111	2.355576879	0.705804049	1.36E-208	SECRETED_FACTORS	3.887056354	3.337437471
**CCL3L3**	5.933883237	5.506648109	0.003870467	5.933883237	4.902368421	8.72E-05	SECRETED_FACTORS	1.077585333	1.210411525
**CLEC11A**	11.83482486	8.470163845	1.36E-205	11.83482486	6.799356275	5.13E-09	ECM_AFFILIATED	1.397236827	1.740580193
**CLEC12A**	11.59656416	4.993967122	0	11.59656416	3.961307692	2.69E-53	ECM_AFFILIATED	2.322114639	2.927458572
**CLEC12B**	8.040116185	1.599652792	0	8.040116185	1.945121862	2.07E-22	ECM_AFFILIATED	5.026163317	4.133476848
**CLEC17A**	5.650477457	2.74487387	1.16E-81	5.650477457	1.722210526	1.03E-06	ECM_AFFILIATED	2.058556321	3.280944676
**CLEC18A**	5.865421387	4.952130182	8.40E-07	5.865421387	4.756684211	5.02E-99	ECM_AFFILIATED	1.184423909	1.233090348
**CLEC2B**	10.06702486	8.191380948	1.46E-53	10.06702486	6.539072065	3.99E-163	ECM_AFFILIATED	1.228977741	1.539518873
**CLEC2D**	10.98961965	8.137838094	4.33E-189	10.98961965	7.754911336	2.19E-157	ECM_AFFILIATED	1.350434787	1.417117382
**CLEC4A**	7.171749133	6.130756971	4.78E-27	7.171749133	0.113618219	1.17E-05	ECM_AFFILIATED	1.169798308	63.12147136
**CLEC4D**	5.328216763	2.227688491	5.67E-112	5.328216763	0.172690688	7.01E-59	ECM_AFFILIATED	2.391814109	30.85410578
**CLEC4G**	4.23326185	2.525975005	1.63E-27	4.23326185	2.918590283	8.42E-06	ECM_AFFILIATED	1.675892217	1.450447455
**CLEC5A**	8.68282948	6.181991885	7.35E-51	8.68282948	3.459968016	1.26E-50	ECM_AFFILIATED	1.404535891	2.509511487
**CLEC7A**	9.081417341	7.747657446	3.71E-23	9.081417341	9.055188664	1.22E-37	ECM_AFFILIATED	1.172150086	1.002896536
**COCH**	8.79724104	6.435326401	8.30E-35	8.79724104	1.409151012	1.31E-10	ECM_GLYCOPROTEINS	1.367023286	6.242937034
**COL19A1**	4.549876879	2.541607475	2.36E-30	4.549876879	2.529996761	0.008396548	COLLAGENS	1.790157183	1.79837261
**COL23A1**	7.52	6.575577972	3.12E-06	7.52	5.123671255	0.023257637	COLLAGENS	1.143625706	1.467697599
**COL24A1**	10.43625665	5.245340508	8.28E-261	10.43625665	5.248983401	1.68E-21	COLLAGENS	1.989624245	1.988243408
**COLQ**	7.852090173	6.248510287	2.40E-61	7.852090173	6.721824291	2.20E-15	ECM_GLYCOPROTEINS	1.256633952	1.16814868
**COLQ**	7.852090173	6.248510287	2.40E-61	7.852090173	6.721824291	2.20E-15	BASEMENT_MEMBRANES	1.256633952	1.16814868
**CRLF3**	11.53849075	9.005339422	0	11.53849075	10.00208988	1.47E-183	SECRETED_FACTORS	1.281294376	1.153607985
**CST7**	11.15283642	6.963638869	6.84E-175	11.15283642	3.935171255	0.004626887	ECM_REGULATORS	1.601581677	2.834142581
**CSTA**	9.417616185	7.583918024	4.44E-15	9.417616185	0.360797571	2.50E-36	ECM_REGULATORS	1.241787709	26.10221616
**CTSA**	13.34331792	12.80281218	4.70E-14	13.34331792	0.218745344	1.72E-221	ECM_REGULATORS	1.042217736	60.99932308
**CTSC**	12.89961503	12.15051554	1.49E-10	12.89961503	2.008253846	1.29E-256	ECM_REGULATORS	1.061651663	6.423299053

**CTSG**	11.20455087	3.707475567	2.35E-303	11.20455087	10.10622955	6.73E-07	ECM_REGULATORS	3.022150966	1.108677653
**CTSS**	13.30250173	11.38905414	7.47E-59	13.30250173	1.435295142	8.42E-259	ECM_REGULATORS	1.168007595	9.268129841
**CTSW**	11.61744509	6.857769217	5.97E-213	11.61744509	7.856733198	1.75E-39	ECM_REGULATORS	1.694056	1.478661015
**CXCL2**	7.757123699	7.036650853	0.000243546	7.757123699	5.453459919	7.18E-11	SECRETED_FACTORS	1.102388602	1.422422428
**CXCL3**	6.194993642	5.329789034	1.94E-05	6.194993642	0.921595951	0.00035187	SECRETED_FACTORS	1.162333744	6.722027839
**CXCL8**	9.700831792	8.007138705	5.05E-15	9.700831792	5.072441296	0.014612053	SECRETED_FACTORS	1.211522886	1.912458169
**DDX26B**	11.44502312	8.11844362	1.16E-262	11.44502312	4.050267206	8.67E-188	ECM_GLYCOPROTEINS	1.409755817	2.825745201
**ECM2**	8.428275145	8.124389432	0.024761478	8.428275145	2.296951417	2.16E-12	ECM_GLYCOPROTEINS	1.037404129	3.669331046
**EGFL8**	8.340536416	7.64745797	2.24E-11	8.340536416	5.877795547	1.22E-50	SECRETED_FACTORS	1.090628605	1.418990564
**ELANE**	10.87367861	1.630488899	0	10.87367861	6.553738462	6.00E-25	ECM_REGULATORS	6.668968199	1.659156629
**EMID1**	10.17125607	8.120219566	7.02E-47	10.17125607	4.51798502	1.33E-95	ECM_GLYCOPROTEINS	1.252583873	2.251281495
**EMILIN2**	12.27820751	9.281946122	1.15E-152	12.27820751	2.904724696	2.12E-66	ECM_GLYCOPROTEINS	1.322805299	4.226978044
**EPGN**	5.804923121	1.832324753	1.29E-97	5.804923121	1.894318623	9.60E-39	SECRETED_FACTORS	3.168064565	3.064385816
**EREG**	8.497576301	4.250374762	3.12E-58	8.497576301	1.47559919	3.84E-56	SECRETED_FACTORS	1.999253425	5.758729306
**EYS**	7.171964162	4.208640295	9.68E-166	7.171964162	4.568393522	3.27E-16	ECM_GLYCOPROTEINS	1.704104808	1.56990945
**F13A1**	11.73250925	9.187595734	7.77E-56	11.73250925	6.308240486	7.65E-06	ECM_REGULATORS	1.276994503	1.85987032
**FAM20B**	11.47866358	11.29822391	0.000205542	11.47866358	1.862123077	1.14E-11	ECM_REGULATORS	1.015970623	6.164288347
**FCN1**	11.33375202	6.31953918	4.57E-202	11.33375202	2.474690688	2.06E-10	ECM_AFFILIATED	1.793445962	4.579866113
**FGF17**	5.616921387	3.078817093	8.03E-52	5.616921387	1.323577328	1.57E-11	SECRETED_FACTORS	1.824376446	4.243742522
**FNDC7**	1.784879769	0.765987948	1.02E-29	1.784879769	0.012794737	1.96E-74	ECM_GLYCOPROTEINS	2.330166907	139.5010926
**GRIFIN**	2.493548555	0.151489393	0	2.493548555	1.624798381	7.27E-76	ECM_AFFILIATED	16.4602188	1.534681832
**HPSE**	8.447778613	7.768240973	9.63E-09	8.447778613	5.664127126	6.99E-25	ECM_REGULATORS	1.087476385	1.491452862
**HYAL3**	9.00286185	7.078630153	4.49E-139	9.00286185	3.313623482	6.34E-257	ECM_REGULATORS	1.271836733	2.716923603
**IMPG2**	6.310452601	3.81454489	9.76E-94	6.310452601	1.175668421	9.90E-122	PROTEOGLYCANS	1.654313367	5.367544529
**KNG1**	3.337751445	2.198945676	0.000122203	3.337751445	2.913063968	9.02E-148	ECM_REGULATORS	1.517887177	1.145787213
**KY**	4.549816763	3.145937086	2.14E-16	4.549816763	1.514387449	1.43E-101	ECM_REGULATORS	1.44625167	3.00439413
**LGALS12**	7.602139306	3.057418955	6.35E-146	7.602139306	0.560155466	3.62E-13	ECM_AFFILIATED	2.486456524	13.57148109
**LGALS8**	12.51443121	11.27085609	1.24E-75	12.51443121	6.346947368	8.52E-198	ECM_AFFILIATED	1.110335463	1.971724435
**LGALS9**	12.76034104	10.85436987	3.43E-65	12.76034104	5.830256275	1.27E-200	ECM_AFFILIATED	1.175594825	2.188641534
**LGALS9C**	4.491741618	3.677051018	4.48E-05	4.491741618	4.138217814	2.88E-31	ECM_AFFILIATED	1.221560864	1.085428999
**LOXL3**	9.226018497	7.824301891	9.83E-36	9.226018497	1.654054656	1.07E-258	ECM_REGULATORS	1.179149095	5.577819611
**MASP2**	8.973761272	4.799598129	8.40E-137	8.973761272	7.579575304	5.92E-117	ECM_REGULATORS	1.869690135	1.183939853
**MFAP3**	9.452482081	8.756427002	8.49E-16	9.452482081	0.008181377	5.59E-175	ECM_GLYCOPROTEINS	1.079490765	1155.365733
**MMP21**	3.063899422	1.247988908	4.75E-114	3.063899422	2.095264777	9.07E-132	ECM_REGULATORS	2.455069433	1.462297011
**MMP25**	7.784336416	6.303884322	2.53E-38	7.784336416	0.021526316	5.18E-71	ECM_REGULATORS	1.234847598	361.6195401
**MMP8**	6.255147399	1.920925771	1.25E-139	6.255147399	2.016360729	3.74E-54	ECM_REGULATORS	3.256319163	3.102196601
**MMRN1**	10.12605318	6.643129145	5.34E-90	10.12605318	7.531660324	6.49E-32	ECM_GLYCOPROTEINS	1.524289677	1.344464931
**MUC19**	6.412321965	1.457422726	5.81E-254	6.412321965	0.566216599	6.88E-72	ECM_AFFILIATED	4.399768063	11.32485691
**MUC6**	6.790424855	4.110726537	1.19E-21	6.790424855	5.225644534	0.007986686	ECM_AFFILIATED	1.65187949	1.299442549
**NGLY1**	11.53614046	9.946942942	2.77E-296	11.53614046	2.755565587	0	ECM_REGULATORS	1.159767431	4.186487346
**NTN3**	3.513287283	2.087444454	1.20E-29	3.513287283	2.342884211	3.36E-88	ECM_GLYCOPROTEINS	1.683056656	1.499556516
**NTN3**	3.513287283	2.087444454	1.20E-29	3.513287283	2.342884211	3.36E-88	BASEMENT_MEMBRANES	1.683056656	1.499556516
**NTN5**	5.130250289	3.67578025	3.65E-34	5.130250289	0.968868421	5.54E-53	ECM_GLYCOPROTEINS	1.39569015	5.295094956
**NTN5**	5.130250289	3.67578025	3.65E-34	5.130250289	0.968868421	5.54E-53	BASEMENT_MEMBRANES	1.39569015	5.295094956
**NTNG2**	10.28887746	5.874825461	6.78E-181	10.28887746	1.466815789	8.87E-14	BASEMENT_MEMBRANES	1.751350321	7.014430531
**NTNG2**	10.28887746	5.874825461	6.78E-181	10.28887746	1.466815789	8.87E-14	ECM_GLYCOPROTEINS	1.751350321	7.014430531

**OGFOD1**	10.66913353	10.46473275	7.98E-08	10.66913353	1.7273	1.33E-45	ECM_REGULATORS	1.019532345	6.17676925
**OGFOD2**	9.175483815	9.066398041	0.032076855	9.175483815	6.328080162	2.18E-38	ECM_REGULATORS	1.012031876	1.449963272
**OGN**	7.705776879	6.172076895	8.59E-10	7.705776879	1.990348178	1.08E-79	PROTEOGLYCANS	1.248490096	3.871572302
**PLXDC2**	11.2131815	10.891156	0.008922507	11.2131815	0.137824291	9.43E-72	ECM_AFFILIATED	1.029567614	81.35852817
**PLXNC1**	11.2651474	9.636059143	2.27E-39	11.2651474	0.794257085	8.42E-122	ECM_AFFILIATED	1.169061671	14.18325075
**PLXND1**	13.51380058	11.83996109	2.03E-82	13.51380058	2.477731579	0.027130201	ECM_AFFILIATED	1.141372043	5.454101926
**PRG2**	8.108887861	1.85115956	0	8.108887861	1.200249798	2.72E-83	PROTEOGLYCANS	4.380437018	6.756000191
**PRG4**	5.660745665	5.028648119	0.008054864	5.660745665	0.170848178	1.44E-106	PROTEOGLYCANS	1.125699299	33.13319303
**SEMA4A**	11.21776012	9.698539083	2.89E-34	11.21776012	0.775150607	6.49E-29	ECM_AFFILIATED	1.156644317	14.47171686
**SEMA4D**	12.7009896	10.40048786	3.09E-161	12.7009896	2.606353846	2.66E-172	ECM_AFFILIATED	1.221191714	4.873087211
**SERPINB1**	14.10931734	11.50648687	1.80E-120	14.10931734	2.378901619	1.41E-116	ECM_REGULATORS	1.226205487	5.93102179
**SERPINB10**	8.213713295	1.092046665	0	8.213713295	5.445735628	2.52E-05	ECM_REGULATORS	7.521394058	1.508283519
**SERPINB2**	7.440518497	3.891332742	5.82E-36	7.440518497	1.472166397	4.49E-54	ECM_REGULATORS	1.9120746	5.054128741
**SERPINB8**	10.98026127	8.745631918	1.94E-131	10.98026127	4.302958704	1.25E-57	ECM_REGULATORS	1.255513767	2.551793318
**SERPINB9**	9.929163584	9.257366473	3.95E-11	9.929163584	0.864357895	0	ECM_REGULATORS	1.072568923	11.48732908
**SERPIND1**	6.521765318	3.246120719	5.30E-37	6.521765318	1.690732794	2.50E-90	ECM_REGULATORS	2.009095127	3.857360159
**SERPINI2**	6.863645087	1.522773366	6.42E-242	6.863645087	5.413568421	2.33E-10	ECM_REGULATORS	4.507331976	1.267859673
**SLIT1**	6.431248555	5.417977274	8.68E-06	6.431248555	5.280717004	1.96E-40	ECM_GLYCOPROTEINS	1.187020216	1.217874116
**SRGN**	15.38833121	10.8746825	0	15.38833121	3.283348988	9.66E-138	PROTEOGLYCANS	1.415060276	4.686779039
**SSPO**	7.86233237	5.512489752	6.89E-51	7.86233237	0.724367206	2.38E-149	ECM_GLYCOPROTEINS	1.426276097	10.85407001
**THBS3**	10.0452815	9.856948885	0.016535824	10.0452815	4.043587045	1.13E-134	ECM_GLYCOPROTEINS	1.019106584	2.484250096
**VCAN**	12.71811908	11.66010408	4.16E-09	12.71811908	0.147120243	2.69E-06	PROTEOGLYCANS	1.09073804	86.44710492
**WFIKKN1**	6.477812717	5.008217452	3.35E-32	6.477812717	0.183051012	8.35E-61	SECRETED_FACTORS	1.293436792	35.38801911
**WNT1**	1.349380925	0.687615969	5.05E-19	1.349380925	0.352096356	4.90E-117	SECRETED_FACTORS	1.962404869	3.832419452
**WNT16**	4.675616763	2.550006147	1.14E-47	4.675616763	2.565715385	7.01E-27	SECRETED_FACTORS	1.833570781	1.822344283
**WNT2B**	8.933253757	7.55502292	1.05E-26	8.933253757	8.476351012	3.12E-92	SECRETED_FACTORS	1.182425765	1.053903236
**VWCE**	7.879437572	5.798354334	6.84E-37	7.879437572	5.376004049	7.98E-55	ECM_GLYCOPROTEINS	1.35890929	1.465668087
**XCL2**	3.796474566	3.486271553	0.029322267	3.796474566	0.041340891	1.29E-42	SECRETED_FACTORS	1.088978443	91.83340038
**ZFP91**	12.38289595	11.64241937	7.35E-53	12.38289595	0.60864332	2.54E-275	SECRETED_FACTORS	1.063601607	20.34507823

Brain Lower Grade Glioma (LGG)

Signature genes	Expression in tumor	Expression in rest of Pan-Cancer	P-value	Expression in tumor	Expression in normal tissue	P-value	Matrisome category	Fold change vs. Pan-Cancer	Fold change vs. normal
**A2M**	14.2534413	14.08456856	0.036741604	14.2534413	13.3281	0.011803153	ECM_REGULATORS	1.011989912	1.069427848
**ABI3BP**	9.620694073	8.639476095	1.94E-20	9.620694073	7.7298	0.009356049	ECM_GLYCOPROTEINS	1.113573782	1.244623932
**ADAM17**	10.56405201	10.17811342	4.00E-14	10.56405201	9.79774	0.014642725	ECM_REGULATORS	1.03791848	1.07821314
**ADAMTS20**	4.615450669	1.601717176	4.98E-191	4.615450669	1.6891	0.012039795	ECM_REGULATORS	2.881564074	2.732491072
**ADAMTS6**	8.155678585	5.613373369	3.95E-235	8.155678585	5.10728	1.88E-09	ECM_REGULATORS	1.4529015	1.596873206
**ADAMTS9**	9.466436902	8.96845603	9.01E-10	9.466436902	8.12938	0.009172046	ECM_REGULATORS	1.05552582	1.164472186
**AMH**	8.026862333	4.640634749	2.78E-225	8.026862333	5.77342	0.005131941	SECRETED_FACTORS	1.729690606	1.390313252
**ANGPTL2**	12.54337361	10.30002002	1.18E-181	12.54337361	9.82668	1.16E-06	SECRETED_FACTORS	1.217800896	1.276460983
**BCAN**	16.70403002	3.738930879	0	16.70403002	13.6481	1.01E-07	PROTEOGLYCANS	4.467595299	1.223908824
**BMP2**	10.59951128	7.892823374	3.41E-142	10.59951128	7.57198	3.06E-05	SECRETED_FACTORS	1.342930252	1.399833502
**BMP7**	12.12375296	7.749604289	3.28E-154	12.12375296	11.11576	0.002296236	SECRETED_FACTORS	1.5644351	1.090681426
**C1QL1**	12.17040229	5.470629542	0	12.17040229	9.54586	0.000391298	ECM_AFFILIATED	2.224680396	1.274940371
**C1QTNF1**	10.10105048	9.803191698	9.68E-05	10.10105048	8.39614	0.003464395	ECM_AFFILIATED	1.030383858	1.203058843
**C1QTNF5**	11.11847897	9.224824415	1.74E-151	11.11847897	8.31122	2.97E-10	ECM_AFFILIATED	1.205278113	1.337767376
**CLEC4F**	5.119072658	3.191926539	2.01E-119	5.119072658	1.03112	1.46E-06	ECM_AFFILIATED	1.603756413	4.964575081
**CRISPLD1**	10.58353767	7.920171806	4.92E-156	10.58353767	8.618	5.46E-05	ECM_GLYCOPROTEINS	1.336276274	1.228073528
**CSF1**	11.42384723	10.52971149	9.45E-56	11.42384723	10.41772	0.019917551	SECRETED_FACTORS	1.084915502	1.096578448
**CTSO**	11.21049962	10.41942741	5.14E-57	11.21049962	9.74212	5.77E-06	ECM_REGULATORS	1.07592281	1.150724854
**EFEMP1**	11.74695679	10.3545701	1.95E-39	11.74695679	10.28012	0.023888391	ECM_GLYCOPROTEINS	1.134470738	1.142686738
**EGFL8**	8.986040918	7.577419851	2.23E-121	8.986040918	8.23844	0.034974029	SECRETED_FACTORS	1.185897191	1.090745447
**ELN**	11.06343327	9.960839426	2.15E-27	11.06343327	9.6098	0.002055739	ECM_GLYCOPROTEINS	1.110692864	1.151265715
**FBN2**	7.914928107	7.156657102	8.43E-11	7.914928107	5.37108	0.00049353	ECM_GLYCOPROTEINS	1.10595324	1.473619478
**FGF11**	10.48437476	7.485597978	1.22E-213	10.48437476	8.68298	5.34E-06	SECRETED_FACTORS	1.400606176	1.207462733
**FGF2**	10.94636119	7.605882212	2.72E-305	10.94636119	10.30794	0.045054253	SECRETED_FACTORS	1.43919678	1.061934895
**GPC2**	9.041081071	5.339490218	0	9.041081071	6.59618	8.94E-07	ECM_AFFILIATED	1.693247989	1.370654086
**IGFBP1**	5.188856788	2.828676024	1.29E-54	5.188856788	3.26974	0.020135081	ECM_GLYCOPROTEINS	1.834376487	1.586932535
**LOXL3**	9.017237667	7.745675268	1.83E-86	9.017237667	7.88156	0.00470528	ECM_REGULATORS	1.164164176	1.144093005
**LTBP3**	13.08680287	12.10880309	6.61E-72	13.08680287	12.31454	0.004088892	ECM_GLYCOPROTEINS	1.080767667	1.062711467
**MASP1**	11.94411759	6.007358593	0	11.94411759	8.93504	6.86E-08	ECM_REGULATORS	1.988247814	1.336772705
**MATN2**	11.53295315	10.21987741	1.24E-58	11.53295315	10.16568	0.008627595	ECM_GLYCOPROTEINS	1.128482534	1.134498937
**MATN3**	7.377682792	6.422352037	1.27E-21	7.377682792	6.55734	0.03658978	ECM_GLYCOPROTEINS	1.148750917	1.125102983
**MEP1A**	3.418512046	2.593111222	2.68E-10	3.418512046	1.95394	0.033023487	ECM_REGULATORS	1.318305214	1.749548116
**MFAP1**	10.52323289	10.36659452	5.42E-12	10.52323289	10.24414	0.030378151	ECM_GLYCOPROTEINS	1.015109916	1.02724415
**MMP16**	11.47538623	6.534650148	0	11.47538623	10.08548	0.004060414	ECM_REGULATORS	1.756082724	1.137812601
**NTN1**	11.71538489	8.219197304	4.68E-251	11.71538489	9.07214	1.26E-09	BASEMENT_MEMBRANES	1.425368495	1.291358477
**NTN1**	11.71538489	8.219197304	4.68E-251	11.71538489	9.07214	1.26E-09	ECM_GLYCOPROTEINS	1.425368495	1.291358477
**PLXNA3**	10.94491185	10.71939606	4.70E-06	10.94491185	10.17876	0.004209866	ECM_AFFILIATED	1.021038107	1.075269665
**PODN**	9.46768738	8.912276391	4.21E-08	9.46768738	7.98692	0.008747869	PROTEOGLYCANS	1.062319767	1.18539905
**SEMA5A**	12.04332581	9.578621883	1.04E-168	12.04332581	10.95534	0.033454814	ECM_AFFILIATED	1.257313	1.099311004
**SEMA6A**	12.28387591	9.411305514	1.42E-238	12.28387591	11.59464	0.031211903	ECM_AFFILIATED	1.305225496	1.059444356
**SMOC1**	13.92400956	6.587153773	0	13.92400956	11.10822	0.000628423	ECM_GLYCOPROTEINS	2.113812739	1.253487018
**SPARC**	16.99384723	15.03396626	1.57E-166	16.99384723	13.82872	1.57E-09	ECM_GLYCOPROTEINS	1.130363534	1.228880708

**SRPX**	10.39182428	8.003414041	9.14E-126	10.39182428	8.07598	0.000191407	ECM_GLYCOPROTEINS	1.298423926	1.28675706
**SULF2**	13.31131549	11.71489504	4.48E-103	13.31131549	11.30822	1.79E-05	ECM_REGULATORS	1.136272706	1.177136233
**TGM5**	3.389331358	2.902514786	3.69E-05	3.389331358	1.55432	0.018243093	ECM_REGULATORS	1.16772234	2.180587883
**TIMP3**	14.11998967	13.16736585	1.83E-30	14.11998967	12.42296	1.52E-05	ECM_REGULATORS	1.072347334	1.136604294
**TIMP4**	11.13904493	4.989430644	0	11.13904493	7.32874	8.34E-09	ECM_REGULATORS	2.232528264	1.519912691
**TNC**	12.42727763	11.78978764	2.39E-09	12.42727763	10.10318	0.000689646	ECM_GLYCOPROTEINS	1.054071371	1.230036249
**VCAN**	13.69108642	11.54805434	9.45E-93	13.69108642	11.1978	1.27E-07	PROTEOGLYCANS	1.185575164	1.222658596
**WNT5A**	9.341081644	9.054258664	0.005387261	9.341081644	8.03454	0.012285011	SECRETED_FACTORS	1.03167824	1.162615613
**VWCE**	8.119607839	5.683643368	4.77E-145	8.119607839	6.86424	0.006739663	ECM_GLYCOPROTEINS	1.428592069	1.18288519

Liver Hepatocellular Carcinoma (LIHC)

Signature genes	Expression in tumor	Expression in rest of Pan-Cancer	P-value	Expression in tumor	Expression in normal tissue	P-value	Matrisome category	Fold change vs. Pan-Cancer	Fold change vs. normal
**ADAMTS10**	8.142894299	7.635274144	1.91E-11	8.142894299	7.486658	0.00195358	ECM_REGULATORS	1.066483553	1.087654104
**AMBN**	0.853220428	0.3279475	2.72E-26	0.853220428	0	0.000890941	ECM_GLYCOPROTEINS	2.60169822	Inf
**C17orf58**	9.232926603	8.744455833	4.14E-28	9.232926603	8.561548	1.33E-10	ECM_REGULATORS	1.055860625	1.078417899
**CBLN1**	5.568139905	3.936572458	5.82E-34	5.568139905	4.419758	0.000348208	SECRETED_FACTORS	1.41446397	1.259829137
**CCL15**	9.854640143	2.415644656	0	9.854640143	9.202952	0.00882769	SECRETED_FACTORS	4.079507355	1.070812946
**CCL20**	8.750070546	5.85601887	1.96E-66	8.750070546	7.15508	0.000230199	SECRETED_FACTORS	1.494201221	1.222917221
**CCL25**	4.60824228	2.165042421	1.35E-81	4.60824228	2.379118	1.82E-06	SECRETED_FACTORS	2.128476669	1.936954065
**CELA3A**	0.91937981	0.528904151	1.97E-05	0.91937981	0.072372	0.000561595	ECM_REGULATORS	1.738273008	12.70352913
**CLEC14A**	9.376042518	9.003399132	2.86E-07	9.376042518	9.030356	0.014919114	ECM_AFFILIATED	1.041389189	1.038280497
**COL27A1**	10.84546865	9.541330536	1.89E-49	10.84546865	10.50045	0.018941356	COLLAGENS	1.136683045	1.03285751
**COL5A3**	10.38562732	9.412519309	3.19E-26	10.38562732	9.283058	2.24E-05	COLLAGENS	1.103384437	1.11877221
**CRELD1**	10.71393492	10.2293722	3.59E-28	10.71393492	10.300148	6.21E-07	ECM_GLYCOPROTEINS	1.047369742	1.04017291
**CRELD2**	10.99834703	10.3681307	6.53E-53	10.99834703	10.296032	3.70E-09	ECM_GLYCOPROTEINS	1.060783988	1.068212204
**CSTL1**	0.699043705	0.580929847	0.016614064	0.699043705	0.374988	0.044393987	ECM_REGULATORS	1.203318626	1.864176202
**CTSA**	13.69355297	12.77392136	4.06E-88	13.69355297	12.823326	1.07E-14	ECM_REGULATORS	1.071992898	1.067862813
**EGLN1**	11.94505012	11.17259565	7.09E-97	11.94505012	11.758498	0.03237828	ECM_REGULATORS	1.069138318	1.015865302
**EGLN2**	11.68868717	11.41882167	3.43E-16	11.68868717	11.209264	1.96E-07	ECM_REGULATORS	1.023633393	1.042770263
**FBLN7**	8.720601425	7.233815028	6.72E-66	8.720601425	8.106112	0.000731723	ECM_GLYCOPROTEINS	1.205532819	1.075805691
**GPC3**	12.46773349	7.863516386	1.36E-237	12.46773349	8.105304	7.88E-18	ECM_AFFILIATED	1.585516311	1.538219108
**LGALS8**	11.78049335	11.269759	5.34E-31	11.78049335	11.555006	0.023552646	ECM_AFFILIATED	1.045319013	1.019514256
**MEP1A**	4.136321378	2.571939098	1.57E-27	4.136321378	1.647292	1.00E-06	ECM_REGULATORS	1.608250126	2.510982496
**MUC13**	8.896255582	4.625577288	6.70E-82	8.896255582	5.724394	1.93E-08	ECM_AFFILIATED	1.923274659	1.554095609
**OGFOD2**	9.753190261	9.036881988	8.12E-107	9.753190261	9.440706	0.000163832	ECM_REGULATORS	1.07926498	1.033099671
**PLOD1**	13.20997387	12.49407436	4.49E-50	13.20997387	12.938524	0.010653468	ECM_REGULATORS	1.057299123	1.020979972
**PLOD3**	12.29088551	11.66115053	1.89E-33	12.29088551	11.326502	2.45E-19	ECM_REGULATORS	1.054002817	1.085143984
**REG1A**	3.881072922	2.828283195	8.92E-07	3.881072922	1.105046	7.88E-06	ECM_AFFILIATED	1.372236319	3.51213698
**REG3A**	4.71291924	1.146505079	3.31E-112	4.71291924	1.272898	5.19E-06	ECM_AFFILIATED	4.110683262	3.702511309
**SEMA6C**	9.14252114	8.341219629	2.00E-35	9.14252114	7.947982	3.09E-11	ECM_AFFILIATED	1.096065269	1.150294646
**SPON2**	12.61736295	10.21770282	1.10E-101	12.61736295	11.768112	4.41E-07	ECM_GLYCOPROTEINS	1.234853192	1.072165437
**TGM3**	6.971352257	3.865587137	4.23E-86	6.971352257	3.389808	4.32E-14	ECM_REGULATORS	1.803439428	2.056562571
**TINAG**	3.103591924	1.888654027	2.28E-13	3.103591924	0.508714	8.02E-07	ECM_GLYCOPROTEINS	1.643282401	6.100858093
**TMPRSS15**	1.453154394	0.984701297	7.30E-08	1.453154394	0.074748	0.000410202	ECM_REGULATORS	1.475731167	19.44071272
**TSPEAR**	3.505656295	2.613054575	3.86E-17	3.505656295	2.752282	0.004042314	ECM_GLYCOPROTEINS	1.341593217	1.273727145
**VWA7**	6.914300713	6.571677298	0.000144315	6.914300713	6.32681	0.002140942	ECM_GLYCOPROTEINS	1.052136372	1.092857335

Lung Adenocarcinoma (LUAD)

Signature genes	Expression in tumor	Expression in rest of Pan-Cancer	P-value	Expression in tumor	Expression in normal tissue	P-value	Matrisome category	Fold change vs. Pan-Cancer	Fold change vs. normal
**ADAM12**	8.848474042	8.348884303	6.73E-06	8.848474042	6.561020339	5.45E-19	ECM_REGULATORS	1.059839102	1.348642983
**ADAM15**	12.71221254	12.12676564	9.63E-44	12.71221254	11.90235424	3.47E-14	ECM_REGULATORS	1.048277251	1.068041859
**ADAM19**	9.638549652	8.974371885	3.75E-21	9.638549652	9.040608475	5.89E-05	ECM_REGULATORS	1.074008273	1.066139484
**ADAM2**	0.721807666	0.558411265	0.01336353	0.721807666	0.058111864	0.000674348	ECM_REGULATORS	1.292609427	12.4210034
**ADAM28**	9.412993728	7.91717527	4.35E-70	9.412993728	7.675532203	5.25E-19	ECM_REGULATORS	1.188933352	1.226363655
**ADAM8**	10.54010592	8.731807571	4.33E-126	10.54010592	8.872135593	3.07E-20	ECM_REGULATORS	1.207093244	1.188000996
**ADAM9**	12.65690906	11.71973271	5.64E-65	12.65690906	12.35555424	0.023919962	ECM_REGULATORS	1.079965676	1.024390231
**ADAMDEC1**	7.424546864	5.936989589	3.77E-36	7.424546864	5.417447458	5.00E-12	ECM_REGULATORS	1.250557501	1.370488025
**ADAMTS12**	9.264763763	7.851592077	1.04E-44	9.264763763	7.958935593	1.02E-11	ECM_REGULATORS	1.179985368	1.164070704
**ADAMTS14**	7.163511672	6.635430198	7.08E-09	7.163511672	4.933784746	1.32E-25	ECM_REGULATORS	1.079585115	1.451930322
**ADAMTS16**	6.612009059	5.034281014	8.16E-44	6.612009059	4.162805085	3.47E-18	ECM_REGULATORS	1.313396896	1.588354229
**ADAMTS2**	9.8477777	9.441430027	5.87E-06	9.8477777	9.341244068	0.002237282	ECM_REGULATORS	1.043038785	1.0542255
**ADAMTSL5**	6.40815122	6.177318889	0.008268301	6.40815122	5.471638983	4.99E-06	ECM_REGULATORS	1.037367721	1.171157534
**AGRN**	13.64358275	13.26079766	1.12E-11	13.64358275	13.23345085	0.005757839	BASEMENT_MEMBRANES	1.028865917	1.03099206
**AGRN**	13.64358275	13.26079766	1.12E-11	13.64358275	13.23345085	0.005757839	ECM_GLYCOPROTEINS	1.028865917	1.03099206
**ANGPTL4**	9.622597213	9.294404527	0.001747439	9.622597213	8.419622034	7.09E-06	SECRETED_FACTORS	1.035310781	1.142877575
**ANXA10**	2.794986237	2.250012568	0.000233315	2.794986237	0.802064407	6.88E-07	ECM_AFFILIATED	1.242209167	3.484740394
**ASPN**	9.445250871	8.283267336	1.33E-26	9.445250871	9.046740678	0.045178635	PROTEOGLYCANS	1.14028082	1.04405014
**BGN**	14.02716411	13.14403239	2.40E-24	14.02716411	13.68819153	0.015882831	PROTEOGLYCANS	1.067188797	1.024763869
**BMP8A**	4.402228049	4.233057469	0.03982155	4.402228049	2.359913559	4.19E-20	SECRETED_FACTORS	1.039964158	1.865419194
**BMP8B**	7.97126446	7.785403332	0.012707897	7.97126446	6.983752542	2.30E-11	SECRETED_FACTORS	1.023873025	1.141401333
**C1QL2**	2.574679617	1.56304504	1.32E-23	2.574679617	1.275869492	0.000567784	ECM_AFFILIATED	1.647220362	2.017980392
**C1QTNF6**	9.506119512	9.358422232	0.013501192	9.506119512	7.607322034	2.29E-26	ECM_AFFILIATED	1.015782284	1.249601301
**CBLN3**	7.112201045	6.731921741	1.72E-12	7.112201045	6.722327119	0.000879314	SECRETED_FACTORS	1.056488967	1.057996869
**CCL1**	1.206354704	0.585415387	2.29E-45	1.206354704	0.541835593	9.67E-05	SECRETED_FACTORS	2.060681578	2.226422035
**CCL19**	8.519009756	6.00968536	3.90E-75	8.519009756	7.427488136	7.92E-05	SECRETED_FACTORS	1.417546718	1.146957033
**CCL20**	8.601482578	5.6792485	8.52E-91	8.601482578	7.528376271	0.000426218	SECRETED_FACTORS	1.514545908	1.142541535
**CCL22**	8.14944007	5.80571685	3.20E-119	8.14944007	7.683220339	0.025103088	SECRETED_FACTORS	1.403692305	1.06068025
**CCL26**	3.287485889	2.992511927	0.000326706	3.287485889	2.41969661	0.000112071	SECRETED_FACTORS	1.098570689	1.358635572
**CCL7**	3.910518467	2.122847421	6.23E-93	3.910518467	2.284505085	5.86E-09	SECRETED_FACTORS	1.842110002	1.711757392
**CHAD**	5.165752091	4.90545464	0.019617279	5.165752091	4.662118644	0.049298488	PROTEOGLYCANS	1.05306286	1.108026733
**CHRD**	8.006191115	7.648675334	4.13E-05	8.006191115	7.406262712	0.003901321	SECRETED_FACTORS	1.046742183	1.081002852
**CHRDL2**	5.85492108	4.721666086	1.36E-19	5.85492108	5.017574576	0.00065872	SECRETED_FACTORS	1.240011677	1.166882722
**CILP**	8.026508537	6.874250443	4.75E-18	8.026508537	6.427998305	4.28E-08	ECM_GLYCOPROTEINS	1.167619452	1.248679318
**CILP2**	7.548761847	6.195282958	6.08E-35	7.548761847	4.452372881	3.73E-27	ECM_GLYCOPROTEINS	1.218469261	1.695446911
**CLCF1**	8.780628746	7.644861121	1.19E-48	8.780628746	7.854884746	9.94E-08	SECRETED_FACTORS	1.148566155	1.117855835
**CLEC17A**	3.594714111	2.697537608	1.65E-25	3.594714111	2.689708475	7.82E-05	ECM_AFFILIATED	1.332590916	1.336469787
**CLEC2D**	8.674361498	8.138486802	1.56E-21	8.674361498	8.176230508	0.000419804	ECM_AFFILIATED	1.065844513	1.060924284
**COCH**	7.039310627	6.334502499	3.49E-11	7.039310627	4.6993	3.97E-15	ECM_GLYCOPROTEINS	1.111264954	1.497948764
**COL10A1**	9.639965331	6.53605465	6.25E-86	9.639965331	5.402659322	4.07E-37	COLLAGENS	1.474890564	1.784300056
**COL11A1**	8.63662892	7.216588393	6.26E-18	8.63662892	3.937052542	3.25E-26	COLLAGENS	1.196774494	2.193678857
**COL17A1**	8.589133275	7.609714255	3.79E-08	8.589133275	5.95710339	6.13E-12	COLLAGENS	1.128706412	1.441830486

**COL1A1**	15.6819561	14.60099167	4.03E-16	15.6819561	13.11496102	6.72E-25	COLLAGENS	1.074033631	1.195730287
**COL1A2**	15.33991185	14.3746144	4.94E-17	15.33991185	14.19507627	8.07E-10	COLLAGENS	1.067152928	1.080650188
**COL24A1**	6.400852091	5.194934608	1.22E-43	6.400852091	4.852837288	8.09E-20	COLLAGENS	1.232133333	1.318991697
**COL28A1**	6.814599826	5.440382605	8.64E-42	6.814599826	6.16759322	0.014323458	COLLAGENS	1.252595694	1.104904228
**COL3A1**	15.35310087	14.10683089	9.19E-23	15.35310087	13.16751695	1.27E-21	COLLAGENS	1.088345142	1.165982997
**COL5A1**	12.73341794	11.68922889	3.43E-23	12.73341794	11.09924068	2.45E-15	COLLAGENS	1.089329164	1.147233249
**COL5A2**	12.07339077	11.40793507	3.70E-12	12.07339077	10.38746949	1.56E-16	COLLAGENS	1.058332704	1.162303367
**COL6A3**	14.10555906	12.4519153	1.42E-44	14.10555906	13.17073898	4.26E-08	BASEMENT_MEMBRANES	1.132802362	1.070977041
**COL6A3**	14.10555906	12.4519153	1.42E-44	14.10555906	13.17073898	4.26E-08	COLLAGENS	1.132802362	1.070977041
**COL9A2**	8.976551045	8.737339028	0.010108377	8.976551045	7.855925424	9.54E-07	COLLAGENS	1.027378132	1.142647182
**COMP**	8.963234843	7.08737953	6.36E-30	8.963234843	6.497016949	2.78E-13	ECM_GLYCOPROTEINS	1.264675442	1.379592344
**CRELD2**	10.67204808	10.36259582	6.48E-18	10.67204808	9.756508475	2.29E-14	ECM_GLYCOPROTEINS	1.029862427	1.093838858
**CRLF1**	9.252050174	6.917476241	2.32E-142	9.252050174	7.502684746	3.96E-06	SECRETED_FACTORS	1.337489259	1.233165259
**CST1**	6.996181882	4.863208286	7.57E-34	6.996181882	1.573347458	2.38E-28	ECM_REGULATORS	1.438593922	4.446685853
**CST2**	5.005652265	3.491917704	5.71E-35	5.005652265	3.155718644	1.92E-09	ECM_REGULATORS	1.433496631	1.586216273
**CST4**	2.511594774	2.116840844	0.000322177	2.511594774	0.19489661	4.02E-13	ECM_REGULATORS	1.186482574	12.88680584
**CSTB**	13.12585784	12.62397843	1.45E-19	13.12585784	12.86257966	0.04338464	ECM_REGULATORS	1.039756041	1.020468536
**CSTL1**	0.852194425	0.539763865	9.06E-14	0.852194425	0.082801695	2.52E-06	ECM_REGULATORS	1.578828224	10.29199252
**CTHRC1**	10.32879547	8.906081078	1.29E-38	10.32879547	6.903474576	1.76E-36	ECM_GLYCOPROTEINS	1.1597464	1.496173464
**CTSA**	13.15958031	12.798058	7.07E-19	13.15958031	12.58433729	1.02E-10	ECM_REGULATORS	1.028248216	1.04571103
**CTSK**	10.83321341	10.28276675	8.02E-10	10.83321341	10.12259661	5.65E-05	ECM_REGULATORS	1.053530988	1.070201039
**CXCL10**	9.284108188	8.267030059	2.27E-18	9.284108188	8.647608475	0.009759792	SECRETED_FACTORS	1.123028237	1.073604132
**CXCL13**	8.705944599	6.042532066	2.96E-77	8.705944599	5.421954237	2.44E-20	SECRETED_FACTORS	1.440777559	1.605683895
**CXCL9**	10.56689251	8.780968916	1.82E-44	10.56689251	9.821430508	0.007066611	SECRETED_FACTORS	1.203385709	1.075901571
**ECM1**	10.37943258	10.00734346	1.72E-05	10.37943258	9.675513559	0.000750012	ECM_GLYCOPROTEINS	1.037181608	1.072752626
**EGF**	7.34174547	5.35711905	4.21E-58	7.34174547	5.913491525	1.83E-08	SECRETED_FACTORS	1.370465245	1.241524646
**EGLN3**	9.967910453	9.514325809	9.01E-06	9.967910453	7.926094915	5.60E-16	ECM_REGULATORS	1.047673861	1.257606748
**EPYC**	3.026194599	2.527657405	4.03E-05	3.026194599	0.858572881	3.65E-11	PROTEOGLYCANS	1.197232898	3.524679925
**EYS**	5.352576481	4.18147638	1.37E-81	5.352576481	4.084064407	1.39E-08	ECM_GLYCOPROTEINS	1.280068568	1.310600409
**FGB**	5.894201742	2.873140865	3.60E-51	5.894201742	2.491511864	1.46E-07	ECM_GLYCOPROTEINS	2.051483731	2.365712894
**FGF19**	2.094317944	1.779890069	0.005757324	2.094317944	0.086684746	1.68E-08	SECRETED_FACTORS	1.176655784	24.16016712
**FGL1**	6.417988153	2.661060758	2.18E-126	6.417988153	2.872713559	2.11E-12	ECM_GLYCOPROTEINS	2.411815714	2.234120465
**FNDC1**	9.255183275	8.057008489	6.92E-19	9.255183275	7.543559322	9.72E-12	ECM_GLYCOPROTEINS	1.148712117	1.22689872
**GREM1**	9.412007491	7.803377768	3.55E-26	9.412007491	5.534950847	5.70E-27	ECM_AFFILIATED	1.206145309	1.700468125
**HABP2**	7.927193554	4.248317993	6.39E-104	7.927193554	6.662459322	0.000659886	ECM_REGULATORS	1.865960497	1.189829937
**HAPLN3**	8.891849826	8.457367485	1.76E-08	8.891849826	7.872386441	2.07E-10	PROTEOGLYCANS	1.051373237	1.129498646
**HPSE**	8.395077352	7.687341837	2.24E-26	8.395077352	8.063440678	0.008636253	ECM_REGULATORS	1.092065051	1.041128432
**HTRA3**	10.59483031	9.087765894	4.68E-47	10.59483031	9.970138983	0.000913911	ECM_REGULATORS	1.165834424	1.062656231
**IBSP**	3.83315993	3.191351938	2.44E-08	3.83315993	1.325101695	1.41E-13	ECM_GLYCOPROTEINS	1.201108497	2.892728871
**IGFBP3**	12.77350436	12.34110359	2.30E-06	12.77350436	10.98132203	7.36E-16	ECM_GLYCOPROTEINS	1.035037447	1.163202783
**IGFBPL1**	4.897288328	3.014307923	6.85E-76	4.897288328	3.627474576	5.73E-07	ECM_GLYCOPROTEINS	1.62468084	1.350054487
**KAZALD1**	7.843300348	7.408597309	1.53E-08	7.843300348	7.289110169	0.000226166	ECM_REGULATORS	1.058675485	1.076029881
**LAMB3**	13.21871568	10.50950965	7.47E-112	13.21871568	12.65810508	0.004757765	BASEMENT_MEMBRANES	1.257786149	1.044288667
**LAMB3**	13.21871568	10.50950965	7.47E-112	13.21871568	12.65810508	0.004757765	ECM_GLYCOPROTEINS	1.257786149	1.044288667
**LGI2**	7.865033624	6.633540331	1.03E-50	7.865033624	5.990388136	2.44E-16	ECM_GLYCOPROTEINS	1.185646462	1.312942241
**LMAN1**	12.61828815	12.35524882	3.09E-13	12.61828815	12.12246271	4.02E-06	ECM_AFFILIATED	1.021289683	1.040901379

**LOXL1**	9.530434495	9.17058456	2.73E-05	9.530434495	8.644011864	7.94E-09	ECM_REGULATORS	1.039239585	1.102547595
**MEP1A**	3.116994774	2.603808457	5.53E-05	3.116994774	2.22670678	7.15E-05	ECM_REGULATORS	1.197090656	1.399822735
**MEPE**	1.644044077	0.96349417	2.69E-25	1.644044077	0.800840678	0.000299754	ECM_GLYCOPROTEINS	1.706335262	2.052897813
**MFAP2**	9.8171223	8.982083524	1.33E-12	9.8171223	8.560520339	3.45E-12	ECM_GLYCOPROTEINS	1.092967158	1.146790371
**MMP1**	9.346113763	6.655851201	4.78E-56	9.346113763	5.255598305	1.57E-24	ECM_REGULATORS	1.404195118	1.778315849
**MMP10**	5.364680314	4.403483631	1.57E-10	5.364680314	3.274566102	3.78E-09	ECM_REGULATORS	1.218280971	1.638287378
**MMP11**	10.25023467	9.902623033	0.004714791	10.25023467	5.693869492	1.11E-36	ECM_REGULATORS	1.035102986	1.800222974
**MMP12**	8.371388153	5.288873839	3.27E-81	8.371388153	4.774123729	1.23E-21	ECM_REGULATORS	1.582829995	1.753492081
**MMP13**	6.980947213	4.027926599	1.93E-68	6.980947213	2.404969492	6.67E-24	ECM_REGULATORS	1.73313665	2.902717576
**MMP14**	13.08398066	12.88654952	0.008714024	13.08398066	12.22577458	7.53E-10	ECM_REGULATORS	1.015320714	1.070196459
**MMP15**	11.38420157	10.79933345	7.00E-19	11.38420157	10.61801017	9.65E-07	ECM_REGULATORS	1.054157797	1.072159603
**MMP17**	7.08890331	6.563556188	1.78E-07	7.08890331	5.245347458	6.51E-15	ECM_REGULATORS	1.080040013	1.351464963
**MMP7**	10.23692892	8.028083566	1.23E-35	10.23692892	9.245591525	0.001139171	ECM_REGULATORS	1.275139806	1.107222712
**MMP8**	2.907500871	1.844659915	8.93E-28	2.907500871	2.022523729	0.000642026	ECM_REGULATORS	1.576171764	1.437560821
**MMP9**	10.63465418	9.186752173	1.50E-32	10.63465418	8.634183051	1.08E-14	ECM_REGULATORS	1.157607605	1.231691999
**MUC13**	6.978524042	4.715810945	1.69E-30	6.978524042	3.868523729	2.66E-09	ECM_AFFILIATED	1.479814209	1.803924321
**MUC16**	8.577284146	5.006450731	1.74E-84	8.577284146	6.194062712	3.47E-07	ECM_AFFILIATED	1.713246491	1.38475901
**MUC20**	10.88399268	8.842053604	2.42E-73	10.88399268	9.301166102	5.47E-11	ECM_AFFILIATED	1.230934936	1.170175069
**MUC21**	9.306322648	2.022117619	0	9.306322648	7.5981	0.000102819	ECM_AFFILIATED	4.602265744	1.224822343
**MUC22**	3.45166777	0.777409738	1.189e-319	3.45166777	2.673716949	0.008153389	ECM_AFFILIATED	4.439959521	1.290962295
**MUC3A**	8.787510279	6.385170582	1.99E-55	8.787510279	6.213061017	3.47E-12	ECM_AFFILIATED	1.376237356	1.414360853
**MUC4**	11.03745767	6.23558142	1.29E-198	11.03745767	9.481749153	7.35E-06	ECM_AFFILIATED	1.770076745	1.164074
**MUC5AC**	7.14116324	2.897735045	6.61E-126	7.14116324	5.402264407	0.004306179	ECM_AFFILIATED	2.464394822	1.321883326
**MUC5B**	11.57477108	5.629958548	5.55E-208	11.57477108	9.071264407	1.38E-06	ECM_AFFILIATED	2.055924743	1.275982108
**MXRA5**	11.52951516	10.94950214	1.59E-07	11.52951516	10.36780339	1.36E-08	ECM_GLYCOPROTEINS	1.052971634	1.112049942
**OVGP1**	6.652351916	6.354716658	3.31E-05	6.652351916	5.599425424	1.68E-09	ECM_AFFILIATED	1.046836905	1.188041882
**P4HA1**	11.6889507	11.05711718	8.91E-51	11.6889507	10.97171186	5.90E-11	ECM_REGULATORS	1.057142699	1.065371643
**P4HA3**	7.281101394	6.291833294	3.96E-32	7.281101394	5.333608475	8.40E-23	ECM_REGULATORS	1.157230501	1.365136085
**PAPLN**	9.518667944	9.165869525	3.41E-06	9.518667944	9.10520678	0.005643014	ECM_GLYCOPROTEINS	1.038490447	1.045409311
**PAPLN**	9.518667944	9.165869525	3.41E-06	9.518667944	9.10520678	0.005643014	BASEMENT_MEMBRANES	1.038490447	1.045409311
**PCOLCE**	11.0133331	10.53977435	1.17E-09	11.0133331	10.67757797	0.029833938	ECM_GLYCOPROTEINS	1.044930635	1.031444878
**PLAT**	11.03202143	10.73300273	0.000841737	11.03202143	10.45898475	0.015789123	ECM_REGULATORS	1.027859743	1.05478894
**PLAU**	11.60701829	10.42690117	2.77E-39	11.60701829	9.921516949	4.90E-13	ECM_REGULATORS	1.113180043	1.169883431
**PLOD2**	11.30051063	11.10990963	0.014903702	11.30051063	9.97949661	7.16E-12	ECM_REGULATORS	1.017155945	1.132372811
**PLXNA3**	10.91470401	10.72869754	9.29E-05	10.91470401	10.08320339	1.44E-09	ECM_AFFILIATED	1.017337283	1.082463934
**PLXNB3**	8.855291463	8.257915569	1.43E-07	8.855291463	6.314579661	1.29E-18	ECM_AFFILIATED	1.072339792	1.402356442
**PODNL1**	7.933080662	6.498366151	8.01E-52	7.933080662	6.008613559	1.02E-15	PROTEOGLYCANS	1.220780805	1.320284719
**POSTN**	12.40337927	10.65579905	9.98E-31	12.40337927	10.94893051	1.52E-11	ECM_GLYCOPROTEINS	1.164002738	1.132839345
**PRSS1**	2.587647038	2.036197918	5.67E-05	2.587647038	0.859279661	2.59E-06	ECM_REGULATORS	1.270822947	3.011414276
**PRSS2**	4.882862021	2.997010112	9.46E-34	4.882862021	2.269169492	2.20E-08	ECM_REGULATORS	1.629244426	2.151827812
**PXDN**	11.67322056	11.2170192	6.52E-09	11.67322056	11.13572712	0.00115538	ECM_GLYCOPROTEINS	1.040670462	1.048267476
**SDC1**	14.15729843	12.22606245	3.77E-68	14.15729843	13.52462712	2.33E-07	ECM_AFFILIATED	1.157960586	1.046779206
**SEMA3A**	8.497528397	6.75711843	1.74E-69	8.497528397	7.798659322	0.004310548	ECM_AFFILIATED	1.257566888	1.089614002
**SEMA4B**	12.38422683	11.75074548	7.05E-33	12.38422683	11.02136102	4.51E-18	ECM_AFFILIATED	1.053909886	1.123656762
**SEMA4C**	10.98127491	10.75306554	1.08E-08	10.98127491	10.15887288	2.03E-12	ECM_AFFILIATED	1.021222727	1.080954063
**SEMA4D**	10.6511507	10.41271202	1.18E-06	10.6511507	10.36877797	0.008306744	ECM_AFFILIATED	1.022898806	1.027232981

**SEMA7A**	8.862530314	8.435009856	3.92E-09	8.862530314	8.313266102	0.000756518	ECM_AFFILIATED	1.05068405	1.066070808
**SERPINB4**	2.874089024	2.46078551	0.009576698	2.874089024	1.176555932	1.92E-05	ECM_REGULATORS	1.167955928	2.442798464
**SERPINF1**	11.7852716	11.2102417	4.88E-10	11.7852716	11.41286441	0.029084951	ECM_REGULATORS	1.05129505	1.032630476
**SERPINH1**	13.05448188	12.6377388	3.83E-12	13.05448188	12.06749492	1.45E-14	ECM_REGULATORS	1.032976079	1.081788886
**SPP1**	12.71463206	11.89340338	1.19E-09	12.71463206	8.47020339	2.22E-31	ECM_GLYCOPROTEINS	1.069049089	1.501101151
**SRPX2**	9.109969686	7.532673305	6.73E-58	9.109969686	7.412852542	2.75E-15	ECM_GLYCOPROTEINS	1.209393972	1.22894252
**ST14**	13.08375679	11.56402765	1.00E-41	13.08375679	11.8177322	3.87E-18	ECM_REGULATORS	1.131418671	1.107129233
**SULF1**	11.70801376	10.9295462	2.66E-13	11.70801376	9.709867797	4.30E-19	ECM_REGULATORS	1.071225973	1.205785085
**THBS2**	11.89290052	11.05818659	5.06E-17	11.89290052	9.532767797	2.39E-24	ECM_GLYCOPROTEINS	1.075483799	1.247581057
**TIMP1**	13.65900174	13.02316839	7.13E-17	13.65900174	12.24449831	4.53E-18	ECM_REGULATORS	1.048823246	1.115521551
**TNC**	12.4145378	11.73852488	3.88E-11	12.4145378	11.63503559	0.001687767	ECM_GLYCOPROTEINS	1.057589257	1.066996117
**TNFAIP6**	7.036201742	6.401114212	5.69E-09	7.036201742	6.654264407	0.033903126	ECM_GLYCOPROTEINS	1.099215154	1.057397379
**VCAN**	12.39823589	11.59335711	3.94E-15	12.39823589	11.20094746	6.05E-10	PROTEOGLYCANS	1.06942586	1.10689171
**WISP1**	8.288399477	7.190848115	1.05E-28	8.288399477	6.434661017	4.96E-20	ECM_GLYCOPROTEINS	1.152631699	1.288086421
**WNT1**	0.908371603	0.686474853	1.05E-07	0.908371603	0.610986441	0.039426808	SECRETED_FACTORS	1.3232409	1.486729561
**WNT10A**	6.541629791	5.866096775	1.66E-08	6.541629791	5.545247458	6.12E-05	SECRETED_FACTORS	1.11515886	1.179682213
**WNT10B**	4.371216551	4.158620673	0.00933399	4.371216551	3.514228814	9.03E-06	SECRETED_FACTORS	1.051121729	1.243862248
**WNT16**	3.005846864	2.471660619	3.12E-11	3.005846864	2.36250678	0.016777571	SECRETED_FACTORS	1.216124431	1.272312482
**WNT3**	8.03024338	6.901643086	3.42E-53	8.03024338	6.367279661	3.70E-25	SECRETED_FACTORS	1.16352632	1.261173344
**WNT5B**	7.792643031	7.450610945	4.48E-05	7.792643031	7.360567797	0.028023667	SECRETED_FACTORS	1.045906583	1.058701346
**WNT8B**	2.032936934	1.776435163	0.000154055	2.032936934	1.357922034	0.000144436	SECRETED_FACTORS	1.144391293	1.497094003
**VWA1**	12.18069303	11.50385576	5.45E-28	12.18069303	11.51391356	1.25E-06	ECM_GLYCOPROTEINS	1.058835688	1.057910759
**VWDE**	5.687624913	4.057989055	1.99E-41	5.687624913	4.637950847	0.000191182	ECM_GLYCOPROTEINS	1.401587051	1.226322809
**ZPLD1**	2.920290244	2.231629151	6.39E-14	2.920290244	0.722018644	1.39E-15	ECM_GLYCOPROTEINS	1.308591189	4.044618886

Lung Squamous Cell Carcinoma (LUAD)

Signature genes	Expression in tumor	Expression in rest of Pan-Cancer	P-value	Expression in tumor	Expression in normal tissue	P-value	Matrisome category	Fold change vs. Pan-Cancer	Fold change vs. normal
**A2ML1**	8.646600547	4.481047987	3.88E-115	8.646600547	2.402734	8.99E-29	ECM_REGULATORS	1.929593384	3.598650765
**ADAM12**	9.935634489	8.348884303	2.76E-44	9.935634489	7.253842	2.82E-25	ECM_REGULATORS	1.190055357	1.369706493
**ADAM17**	11.59019434	10.0946329	1.89E-202	11.59019434	11.251512	0.001617325	ECM_REGULATORS	1.148154119	1.030101052
**ADAM20**	2.160307482	1.720521954	2.67E-19	2.160307482	1.830912	0.041962926	ECM_REGULATORS	1.255611692	1.179907872
**ADAM23**	9.8278	6.705551778	6.90E-152	9.8278	6.908458	6.61E-12	ECM_REGULATORS	1.465621372	1.422575052
**ADAM28**	8.67137135	7.91717527	1.44E-18	8.67137135	7.974716	0.000318447	ECM_REGULATORS	1.095260753	1.087358014
**ADAMDEC1**	7.879349453	5.936989589	1.08E-57	7.879349453	5.746754	1.80E-12	ECM_REGULATORS	1.327162417	1.371095657
**ADAMTS12**	9.588904745	7.851592077	1.96E-63	9.588904745	8.539328	8.99E-07	ECM_REGULATORS	1.22126884	1.12291093
**ADAMTS14**	7.340764781	6.635430198	3.71E-14	7.340764781	5.62757	2.48E-15	ECM_REGULATORS	1.106298245	1.304428871
**ADAMTS17**	7.221102007	6.498386492	6.70E-24	7.221102007	6.727954	0.020029304	ECM_REGULATORS	1.111214609	1.073298362
**ADAMTS18**	4.100185401	3.840909856	0.011868452	4.100185401	2.623042	6.14E-06	ECM_REGULATORS	1.067503679	1.563141346
**ADAMTS20**	4.408810401	1.624489002	4.31E-169	4.408810401	0.568792	3.28E-24	ECM_REGULATORS	2.713967529	7.751182157
**ADAMTSL5**	6.943487591	6.177318889	1.09E-17	6.943487591	5.74168	3.49E-08	ECM_REGULATORS	1.124029327	1.209312883
**AGRN**	13.54030584	13.26079766	1.16E-06	13.54030584	13.21525	0.033302707	BASEMENT_MEMBRANES	1.02107778	1.024597025
**AGRN**	13.54030584	13.26079766	1.16E-06	13.54030584	13.21525	0.033302707	ECM_GLYCOPROTEINS	1.02107778	1.024597025
**ANXA10**	3.382676095	2.250012568	7.10E-14	3.382676095	1.124568	7.47E-08	ECM_AFFILIATED	1.5034032	3.007978259
**ANXA8**	11.19839507	5.352544442	4.82E-199	11.19839507	9.918826	0.000108298	ECM_AFFILIATED	2.092162932	1.129004085
**ARTN**	8.079219891	5.385328083	6.18E-179	8.079219891	3.81504	1.08E-36	SECRETED_FACTORS	1.500227983	2.11772875
**ASTL**	2.460307299	1.92820977	6.55E-15	2.460307299	1.79538	0.001139337	ECM_REGULATORS	1.275954171	1.370354632
**BMP1**	10.78444763	10.64880498	0.005389351	10.78444763	10.46007	0.012991429	ECM_REGULATORS	1.012737829	1.031011038
**BMP7**	10.44977956	7.991588991	1.01E-49	10.44977956	7.370448	2.62E-19	SECRETED_FACTORS	1.307597222	1.417794354
**C1QTNF6**	9.893833577	9.358422232	1.69E-18	9.893833577	8.017602	2.90E-25	ECM_AFFILIATED	1.057211711	1.234014058
**CCL26**	5.073585219	2.992511927	1.42E-130	5.073585219	2.649104	9.91E-19	SECRETED_FACTORS	1.6954269	1.915208017
**CCL7**	3.754296533	2.122847421	7.44E-76	3.754296533	2.688636	4.29E-05	SECRETED_FACTORS	1.768519252	1.39635731
**CD109**	11.66947117	9.685100993	2.12E-114	11.66947117	10.475196	2.81E-09	ECM_REGULATORS	1.20488895	1.114009816
**CILP**	7.870831204	6.874250443	2.33E-13	7.870831204	7.15791	0.021615322	ECM_GLYCOPROTEINS	1.144973008	1.099599074
**CILP2**	7.506387774	6.195282958	8.13E-32	7.506387774	5.045382	1.78E-19	ECM_GLYCOPROTEINS	1.211629529	1.487773924
**CLEC2A**	0.643396533	0.358863609	2.43E-08	0.643396533	0.043618	0.00644145	ECM_AFFILIATED	1.792872045	14.75071147
**CLEC2L**	4.673113321	2.600579466	3.43E-74	4.673113321	1.674224	7.01E-12	ECM_AFFILIATED	1.796950788	2.791211523
**COCH**	8.27134854	6.334502499	2.90E-69	8.27134854	5.474966	1.03E-14	ECM_GLYCOPROTEINS	1.305761351	1.510757974
**COL10A1**	9.31967135	6.53605465	4.69E-67	9.31967135	6.248992	4.67E-22	COLLAGENS	1.425886387	1.491387947
**COL11A1**	9.538168613	7.216588393	1.78E-43	9.538168613	4.435304	1.38E-33	COLLAGENS	1.321700517	2.150510678
**COL16A1**	11.03827135	10.44124955	1.24E-11	11.03827135	10.446768	0.004580538	COLLAGENS	1.057179153	1.056620703
**COL17A1**	11.5984708	7.609714255	9.34E-104	11.5984708	6.779046	7.83E-27	COLLAGENS	1.524166403	1.71092965
**COL19A1**	3.078537956	2.565919327	5.57E-07	3.078537956	2.354778	0.040761256	COLLAGENS	1.199779714	1.307358042
**COL1A1**	16.03940182	14.60099167	4.07E-26	16.03940182	14.015766	1.48E-13	COLLAGENS	1.098514552	1.14438282
**COL1A2**	15.58816661	14.3746144	9.01E-25	15.58816661	14.877108	0.001906581	COLLAGENS	1.084423287	1.047795486
**COL24A1**	6.535150912	5.194934608	3.44E-51	6.535150912	5.455178	1.20E-09	COLLAGENS	1.257985212	1.197972076
**COL27A1**	9.998671168	9.600441399	5.14E-07	9.998671168	9.153602	3.48E-05	COLLAGENS	1.04148036	1.092320943
**COL2A1**	4.731746533	4.216924634	0.000611432	4.731746533	2.873426	1.36E-06	COLLAGENS	1.122084681	1.646726428
**COL3A1**	15.86105219	14.10683089	2.39E-41	15.86105219	14.058312	8.50E-12	COLLAGENS	1.124352614	1.128233047

**COL4A5**	11.00072245	9.233981548	1.55E-64	11.00072245	10.011668	2.46E-05	COLLAGENS	1.191330347	1.098790176
**COL4A5**	11.00072245	9.233981548	1.55E-64	11.00072245	10.011668	2.46E-05	BASEMENT_MEMBRANES	1.191330347	1.098790176
**COL4A6**	9.060562409	6.084243043	8.62E-111	9.060562409	7.154312	5.60E-08	BASEMENT_MEMBRANES	1.489184825	1.26644776
**COL4A6**	9.060562409	6.084243043	8.62E-111	9.060562409	7.154312	5.60E-08	COLLAGENS	1.489184825	1.26644776
**COL5A1**	13.01577391	11.68922889	9.39E-35	13.01577391	11.810644	1.46E-07	COLLAGENS	1.11348439	1.102037612
**COL5A2**	12.5918188	11.40793507	1.64E-33	12.5918188	11.118954	1.10E-11	COLLAGENS	1.103777215	1.13246433
**COL7A1**	12.0410292	9.192337939	6.19E-131	12.0410292	7.040214	2.94E-40	COLLAGENS	1.309898448	1.710321476
**CRELD2**	10.64335967	10.36259582	8.62E-15	10.64335967	10.004696	4.03E-10	ECM_GLYCOPROTEINS	1.027093969	1.06383639
**CRISPLD1**	8.665226095	8.102181612	3.73E-08	8.665226095	7.840674	0.000131764	ECM_GLYCOPROTEINS	1.069492948	1.10516342
**CRNN**	2.342176642	1.271700427	1.01E-16	2.342176642	0.924678	0.000714504	SECRETED_FACTORS	1.841767599	2.532964602
**CST1**	7.341701825	4.863208286	2.75E-43	7.341701825	2.290916	1.38E-23	ECM_REGULATORS	1.509641659	3.204701449
**CST11**	0.338239234	0.206593668	4.18E-06	0.338239234	0	0.0028002	ECM_REGULATORS	1.637219751	Inf
**CST2**	4.080574453	3.491917704	2.38E-06	4.080574453	3.254296	0.007377502	ECM_REGULATORS	1.168576925	1.253903902
**CST4**	2.492257299	2.116840844	0.000793745	2.492257299	0.094936	2.51E-13	ECM_REGULATORS	1.177347511	26.2519729
**CSTA**	11.59151241	7.376330763	1.38E-217	11.59151241	9.299326	8.50E-14	ECM_REGULATORS	1.57144694	1.246489521
**CSTB**	13.73386807	12.62397843	1.58E-83	13.73386807	13.07272	1.74E-05	ECM_REGULATORS	1.087919164	1.050574637
**CSTL1**	1.054700547	0.539763865	1.00E-33	1.054700547	0.0424	3.28E-10	ECM_REGULATORS	1.954003621	24.87501291
**CTHRC1**	10.68446241	8.906081078	3.84E-57	10.68446241	7.69606	8.52E-34	ECM_GLYCOPROTEINS	1.199681691	1.388302899
**CTSK**	11.14175584	10.28276675	8.75E-21	11.14175584	10.655276	0.015407042	ECM_REGULATORS	1.083536767	1.04565624
**CTSV**	9.500482117	7.28437811	2.23E-81	9.500482117	5.84878	1.55E-38	ECM_REGULATORS	1.304226932	1.624352791
**CXCL10**	9.611589781	8.267030059	1.89E-29	9.611589781	9.058222	0.048275056	SECRETED_FACTORS	1.162641204	1.06109011
**CXCL13**	9.079429197	6.042532066	3.70E-95	9.079429197	6.130736	9.74E-15	SECRETED_FACTORS	1.502586846	1.480968875
**CXCL14**	10.76109197	9.895338473	1.12E-07	10.76109197	7.702888	3.00E-16	SECRETED_FACTORS	1.087491044	1.397020438
**CXCL6**	7.429093248	4.310898089	4.68E-106	7.429093248	6.05043	0.000433487	SECRETED_FACTORS	1.723328433	1.227862028
**DDX26B**	8.40835	8.158415761	1.37E-05	8.40835	8.067562	0.013442348	ECM_GLYCOPROTEINS	1.030635144	1.042241758
**DMP1**	2.225825365	1.386894842	3.67E-34	2.225825365	0.640736	6.07E-16	ECM_GLYCOPROTEINS	1.604898437	3.473857197
**DSPP**	0.90179854	0.544958676	1.32E-15	0.90179854	0.10041	4.00E-09	ECM_GLYCOPROTEINS	1.654801694	8.981162635
**EGLN3**	11.0053438	9.514325809	5.89E-46	11.0053438	8.391256	3.01E-23	ECM_REGULATORS	1.156712942	1.311525211
**EMILIN3**	6.201634307	4.974561559	1.33E-27	6.201634307	4.257484	2.03E-07	ECM_GLYCOPROTEINS	1.246669527	1.456643009
**EPYC**	2.94842792	2.527657405	0.000663543	2.94842792	0.993914	7.35E-10	PROTEOGLYCANS	1.166466592	2.966481929
**F12**	7.722950365	7.239870699	1.17E-05	7.722950365	4.653612	2.47E-37	ECM_REGULATORS	1.066724902	1.659560437
**FAM20B**	11.70546496	11.28599471	1.33E-50	11.70546496	11.462064	0.009034253	ECM_REGULATORS	1.037167326	1.021235352
**FBN2**	8.654026825	7.214706321	1.33E-36	8.654026825	5.714504	1.28E-10	ECM_GLYCOPROTEINS	1.199498142	1.514396844
**FGF11**	9.319413869	7.581973561	4.79E-72	9.319413869	5.181768	9.32E-45	SECRETED_FACTORS	1.229154097	1.798500795
**FGF12**	7.214895985	6.297278665	1.87E-18	7.214895985	6.22049	0.000505748	SECRETED_FACTORS	1.145716486	1.159859751
**FGF19**	2.867705474	1.779890069	3.14E-20	2.867705474	0.319134	3.97E-08	SECRETED_FACTORS	1.611169995	8.985897693
**FGF3**	0.655310766	0.441621676	0.000522181	0.655310766	0.066276	0.026133206	SECRETED_FACTORS	1.483873644	9.887602849
**FNDC1**	9.342585949	8.057008489	1.36E-20	9.342585949	8.008618	1.52E-06	ECM_GLYCOPROTEINS	1.159560147	1.16656656
**FNDC8**	1.346417336	1.185251575	0.000270973	1.346417336	0.902056	0.002342554	ECM_GLYCOPROTEINS	1.135975994	1.492609479
**GPC1**	13.20194161	11.54649143	9.85E-132	13.20194161	10.789932	2.96E-26	ECM_AFFILIATED	1.143372573	1.223542614
**GPC2**	5.882286679	5.581202819	0.001243922	5.882286679	3.24719	2.59E-26	ECM_AFFILIATED	1.053946052	1.811500614
**GREM1**	10.09896624	7.803377768	1.65E-49	10.09896624	6.264874	3.18E-29	ECM_AFFILIATED	1.294178821	1.611998301
**HAPLN3**	9.618239234	8.457367485	9.39E-49	9.618239234	8.514438	1.15E-11	PROTEOGLYCANS	1.137261595	1.129638766
**HPSE**	8.708708759	7.687341837	2.44E-50	8.708708759	8.345318	0.015031287	ECM_REGULATORS	1.132863471	1.043544267
**HRG**	2.512062774	1.764826866	2.15E-06	2.512062774	0.461036	3.99E-11	ECM_REGULATORS	1.423404654	5.448734532
**HTRA3**	10.83117482	9.087765894	1.10E-59	10.83117482	10.447054	0.040750311	ECM_REGULATORS	1.191841311	1.036768338

**IBSP**	4.025513869	3.191351938	7.54E-13	4.025513869	1.25143	4.80E-24	ECM_GLYCOPROTEINS	1.261381993	3.216731154
**IGFBP2**	13.49237664	11.45141622	4.70E-87	13.49237664	11.0501	1.39E-16	ECM_GLYCOPROTEINS	1.178227774	1.22101851
**IGFBP3**	13.43426624	12.34110359	2.89E-31	13.43426624	11.424918	7.50E-16	ECM_GLYCOPROTEINS	1.088579003	1.175874194
**IGFBP5**	13.89259982	13.31700634	1.90E-08	13.89259982	13.174706	0.000860734	ECM_GLYCOPROTEINS	1.043222438	1.05449031
**ITIH6**	3.166669891	2.714329856	0.000482406	3.166669891	0.795968	4.04E-21	ECM_REGULATORS	1.166648882	3.978388441
**KCP**	6.424840511	5.963599477	9.35E-06	6.424840511	4.890912	3.54E-10	ECM_GLYCOPROTEINS	1.077342725	1.313628319
**LAMA1**	8.62599635	7.364141068	6.39E-25	8.62599635	6.515078	2.02E-09	ECM_GLYCOPROTEINS	1.171351319	1.324005077
**LAMA1**	8.62599635	7.364141068	6.39E-25	8.62599635	6.515078	2.02E-09	BASEMENT_MEMBRANES	1.171351319	1.324005077
**LAMA5**	12.91348102	12.29558791	8.90E-23	12.91348102	12.479082	0.008005131	BASEMENT_MEMBRANES	1.050253238	1.034810174
**LAMA5**	12.91348102	12.29558791	8.90E-23	12.91348102	12.479082	0.008005131	ECM_GLYCOPROTEINS	1.050253238	1.034810174
**LAMB3**	13.66504434	10.50950965	1.73E-143	13.66504434	12.649714	1.43E-06	BASEMENT_MEMBRANES	1.300255178	1.080265083
**LAMB3**	13.66504434	10.50950965	1.73E-143	13.66504434	12.649714	1.43E-06	ECM_GLYCOPROTEINS	1.300255178	1.080265083
**LAMC2**	13.53287135	9.777165179	2.84E-106	13.53287135	12.485904	4.39E-05	BASEMENT_MEMBRANES	1.38413038	1.083851946
**LAMC2**	13.53287135	9.777165179	2.84E-106	13.53287135	12.485904	4.39E-05	ECM_GLYCOPROTEINS	1.38413038	1.083851946
**LGALS7**	6.138242883	2.521967037	1.53E-141	6.138242883	1.662822	6.19E-21	ECM_AFFILIATED	2.43391083	3.691461193
**LGI2**	7.271919526	6.633540331	2.13E-14	7.271919526	6.685724	0.011557024	ECM_GLYCOPROTEINS	1.096235066	1.087678691
**LMAN1**	12.60747974	12.35524882	4.30E-12	12.60747974	12.224118	7.03E-06	ECM_AFFILIATED	1.02041488	1.031361097
**LOXL1**	10.02550931	9.17058456	2.25E-22	10.02550931	9.165316	1.10E-07	ECM_REGULATORS	1.093224673	1.093853099
**LOXL2**	10.49420985	10.1490868	8.36E-06	10.49420985	9.479968	4.40E-07	ECM_REGULATORS	1.034005331	1.106987898
**MFAP1**	10.44435566	10.38744451	0.008601781	10.44435566	10.228088	0.000835841	ECM_GLYCOPROTEINS	1.00547884	1.021144485
**MFAP2**	10.76625566	8.982083524	2.94E-49	10.76625566	9.0782	2.74E-18	ECM_GLYCOPROTEINS	1.198636778	1.185946075
**MMP1**	11.06820146	6.655851201	7.54E-139	11.06820146	6.488356	2.61E-28	ECM_REGULATORS	1.66292802	1.705856069
**MMP10**	8.922525	4.403483631	1.16E-178	8.922525	4.411314	3.00E-18	ECM_REGULATORS	2.026242346	2.022645633
**MMP11**	11.24844361	9.902623033	8.78E-27	11.24844361	6.389998	1.59E-41	ECM_REGULATORS	1.135905464	1.760320365
**MMP12**	10.36185438	5.288873839	1.58E-202	10.36185438	5.287452	8.00E-41	ECM_REGULATORS	1.959179722	1.959706562
**MMP13**	7.822345803	4.027926599	2.21E-105	7.822345803	3.633838	9.08E-16	ECM_REGULATORS	1.942027892	2.152640212
**MMP14**	13.77446004	12.88654952	1.18E-30	13.77446004	12.540866	2.52E-17	ECM_REGULATORS	1.068902115	1.098365937
**MMP20**	1.20065146	0.621304132	1.78E-27	1.20065146	0.07673	3.00E-06	ECM_REGULATORS	1.93246978	15.64774482
**MMP27**	1.014506569	0.790822424	0.000173395	1.014506569	0.583164	0.023556332	ECM_REGULATORS	1.282850029	1.739659117
**MMP3**	6.709239964	4.47830094	2.70E-38	6.709239964	2.545218	6.30E-22	ECM_REGULATORS	1.498166393	2.636017804
**MMP7**	10.10642318	8.028083566	1.43E-30	10.10642318	9.45505	0.020689762	ECM_REGULATORS	1.258883654	1.068891563
**MMP8**	3.559166606	1.844659915	4.70E-66	3.559166606	2.581158	0.000465321	ECM_REGULATORS	1.929443242	1.378903037
**MMP9**	11.20170766	9.186752173	1.56E-58	11.20170766	9.37426	5.33E-12	ECM_REGULATORS	1.219332736	1.194943138
**MUC20**	10.1498531	8.842053604	7.33E-30	10.1498531	9.471318	0.022205436	ECM_AFFILIATED	1.14790676	1.071641043
**MUCL1**	3.799071168	2.585652963	2.49E-15	3.799071168	1.998758	1.44E-06	ECM_AFFILIATED	1.469288888	1.900715929
**MXRA5**	12.10781825	10.94950214	1.69E-24	12.10781825	10.90326	5.89E-08	ECM_GLYCOPROTEINS	1.105787103	1.110476889
**NELL1**	5.049544708	3.183604624	9.32E-40	5.049544708	3.582784	0.002912466	ECM_GLYCOPROTEINS	1.586109239	1.40939133
**NELL2**	9.280001642	7.784212985	2.64E-35	9.280001642	8.542114	0.005751805	ECM_GLYCOPROTEINS	1.192156697	1.086382322
**NTN1**	9.740960584	8.373452995	2.99E-38	9.740960584	9.244002	0.029825567	ECM_GLYCOPROTEINS	1.163314655	1.053760112
**NTN1**	9.740960584	8.373452995	2.99E-38	9.740960584	9.244002	0.029825567	BASEMENT_MEMBRANES	1.163314655	1.053760112
**NTN3**	2.346226277	2.122345115	0.002222824	2.346226277	1.454284	2.89E-05	ECM_GLYCOPROTEINS	1.105487633	1.61332056
**NTN3**	2.346226277	2.122345115	0.002222824	2.346226277	1.454284	2.89E-05	BASEMENT_MEMBRANES	1.105487633	1.61332056
**OGFOD1**	10.59556606	10.4677929	6.54E-09	10.59556606	10.249286	4.13E-08	ECM_REGULATORS	1.012206313	1.033785774
**OTOG**	1.545167701	1.321034351	0.007569108	1.545167701	0.082344	6.90E-09	ECM_GLYCOPROTEINS	1.169665043	18.76478797
**P4HA1**	11.94876113	11.05711718	4.85E-94	11.94876113	11.087436	9.81E-13	ECM_REGULATORS	1.080639821	1.077684789
**P4HA3**	6.910732117	6.291833294	4.17E-13	6.910732117	6.022376	5.78E-06	ECM_REGULATORS	1.09836542	1.147509242

**PI3**	10.01309726	5.739437608	1.91E-99	10.01309726	6.41362	3.61E-13	ECM_REGULATORS	1.744612965	1.561223968
**PLAT**	11.46175347	10.73300273	1.36E-15	11.46175347	10.873996	0.008476133	ECM_REGULATORS	1.067898122	1.054051654
**PLAU**	12.33468102	10.42690117	3.23E-95	12.33468102	10.292114	2.73E-20	ECM_REGULATORS	1.182967098	1.198459425
**PLOD1**	12.8454146	12.50815465	4.02E-15	12.8454146	12.052784	1.73E-10	ECM_REGULATORS	1.026963206	1.065763279
**PLOD2**	11.87069945	11.10990963	1.42E-21	11.87069945	10.217694	1.83E-21	ECM_REGULATORS	1.068478489	1.16177872
**PLXNA1**	12.58902974	11.44635716	2.85E-91	12.58902974	11.957206	2.17E-06	ECM_AFFILIATED	1.099828493	1.052840416
**PLXNB3**	9.293770438	8.257915569	2.56E-19	9.293770438	6.656892	1.07E-29	ECM_AFFILIATED	1.125437813	1.396112546
**PODNL1**	7.758440146	6.498366151	2.61E-39	7.758440146	6.501072	9.55E-10	PROTEOGLYCANS	1.193906278	1.193409356
**POSTN**	12.73912372	10.65579905	5.97E-41	12.73912372	11.457848	2.50E-07	ECM_GLYCOPROTEINS	1.195510882	1.111825163
**PRSS1**	3.104124453	2.036197918	2.03E-14	3.104124453	1.168906	8.35E-08	ECM_REGULATORS	1.52447089	2.655580904
**PRSS2**	5.38712646	2.997010112	3.48E-51	5.38712646	3.314658	7.61E-06	ECM_REGULATORS	1.797500261	1.625243527
**PXDNL**	4.993241788	4.497337266	4.58E-08	4.993241788	4.575702	0.029967658	ECM_GLYCOPROTEINS	1.110266251	1.091251526
**SDC1**	14.79921186	12.22606245	1.15E-112	14.79921186	13.461974	4.47E-16	ECM_AFFILIATED	1.210464279	1.099334456
**SEMA3F**	11.50432518	10.60397557	1.53E-28	11.50432518	10.607284	5.99E-12	ECM_AFFILIATED	1.084906798	1.084568414
**SEMA4B**	13.13464562	11.75074548	4.95E-139	13.13464562	11.28714	6.24E-23	ECM_AFFILIATED	1.117771264	1.163682352
**SEMA4C**	11.07020438	10.75306554	6.45E-15	11.07020438	10.403734	1.12E-08	ECM_AFFILIATED	1.029492877	1.06406069
**SEMA4F**	9.450489599	8.601750593	1.22E-79	9.450489599	8.960048	3.64E-05	ECM_AFFILIATED	1.098670497	1.054736492
**SERPINB11**	4.250412774	1.213348105	2.05E-156	4.250412774	2.500328	0.00012945	ECM_REGULATORS	3.503044804	1.699942077
**SERPINB12**	3.030064964	0.6472548	2.00E-199	3.030064964	0.3851	1.28E-10	ECM_REGULATORS	4.681409801	7.868254904
**SERPINB13**	9.081497993	2.003399968	0	9.081497993	2.821118	6.89E-26	ECM_REGULATORS	4.533042896	3.219113129
**SERPINB2**	7.883534672	3.769084495	4.03E-140	7.883534672	5.37378	2.01E-08	ECM_REGULATORS	2.091631186	1.467037108
**SERPINB3**	9.617741058	2.953548105	2.16E-252	9.617741058	5.719938	3.86E-13	ECM_REGULATORS	3.256334658	1.681441487
**SERPINB4**	7.508500547	2.46078551	2.21E-197	7.508500547	2.282844	1.61E-21	ECM_REGULATORS	3.051261688	3.289099276
**SERPINB5**	11.44991825	5.779809963	4.03E-149	11.44991825	4.77262	6.36E-47	ECM_REGULATORS	1.981019847	2.399084412
**SERPINB7**	6.524130657	3.028885542	3.06E-138	6.524130657	2.85953	1.88E-17	ECM_REGULATORS	2.153970682	2.281539504
**SERPINE2**	11.08869818	10.80134688	0.005466568	11.08869818	8.999292	9.82E-19	ECM_REGULATORS	1.026603284	1.232174506
**SERPINH1**	13.35134416	12.6377388	2.91E-31	13.35134416	12.423648	1.66E-13	ECM_REGULATORS	1.056466221	1.0746718
**SERPINI1**	8.411880109	8.004189151	3.44E-08	8.411880109	7.758726	0.001055219	ECM_REGULATORS	1.050934698	1.084183165
**SPOCK3**	5.51814854	3.721253049	2.77E-29	5.51814854	4.162816	0.000720377	PROTEOGLYCANS	1.482873771	1.325580698
**SPP1**	13.54572299	11.89340338	5.53E-33	13.54572299	9.487244	1.73E-28	ECM_GLYCOPROTEINS	1.1389274	1.427782715
**SRPX2**	9.098075547	7.532673305	3.55E-55	9.098075547	8.074988	3.15E-08	ECM_GLYCOPROTEINS	1.207814965	1.126698337
**ST14**	12.98226661	11.56402765	3.65E-35	12.98226661	11.851808	6.59E-19	ECM_REGULATORS	1.122642301	1.095382798
**SULF1**	12.15466004	10.9295462	2.23E-29	12.15466004	10.294112	3.74E-17	ECM_REGULATORS	1.112091921	1.180739051
**TGM3**	5.620668978	3.957551628	1.54E-31	5.620668978	4.407694	0.006882783	ECM_REGULATORS	1.420238952	1.275194916
**TGM4**	2.787642701	2.008516903	1.16E-13	2.787642701	2.08783	0.005902149	ECM_REGULATORS	1.387910998	1.33518663
**TGM5**	4.813358577	2.888203759	1.23E-61	4.813358577	1.353268	1.01E-20	ECM_REGULATORS	1.666557826	3.556840609
**TGM6**	0.798854927	0.259174704	3.54E-36	0.798854927	0.013962	0.000206453	ECM_REGULATORS	3.082302847	57.21636778
**THBS2**	12.15564288	11.05818659	4.30E-27	12.15564288	10.365326	9.17E-14	ECM_GLYCOPROTEINS	1.099243785	1.172721715
**TLL2**	5.185689416	4.938260406	0.001887203	5.185689416	2.881134	3.68E-20	ECM_REGULATORS	1.050104488	1.799877901
**TMPRSS15**	1.564481387	0.974273241	2.36E-14	1.564481387	0.342546	1.58E-06	ECM_REGULATORS	1.605793243	4.56721546
**TNC**	13.31201953	11.73852488	1.35E-50	13.31201953	12.211386	7.66E-05	ECM_GLYCOPROTEINS	1.134045347	1.090131745
**TSKU**	11.7310615	10.79481481	3.26E-34	11.7310615	9.818012	3.00E-30	ECM_GLYCOPROTEINS	1.086731148	1.194851004
**VCAN**	12.36162664	11.59335711	2.17E-13	12.36162664	11.758308	0.002691562	PROTEOGLYCANS	1.066268082	1.051309988
**WISP1**	8.824367883	7.190848115	2.09E-58	8.824367883	7.348638	2.98E-11	ECM_GLYCOPROTEINS	1.227166496	1.200816789
**WNT10A**	7.135885401	5.866096775	5.16E-25	7.135885401	5.978018	7.09E-05	SECRETED_FACTORS	1.21646227	1.193687507
**WNT10B**	5.424705109	4.158620673	4.33E-50	5.424705109	3.643778	1.21E-10	SECRETED_FACTORS	1.304448166	1.488758401

**WNT16**	4.082458577	2.471660619	2.44E-83	4.082458577	2.480258	3.01E-07	SECRETED_FACTORS	1.651706769	1.645981417
**WNT3**	7.473125547	6.901643086	2.21E-14	7.473125547	6.459614	1.21E-09	SECRETED_FACTORS	1.082803827	1.156899708
**WNT5A**	11.1535219	8.931545478	4.00E-107	11.1535219	9.820158	6.40E-08	SECRETED_FACTORS	1.248778493	1.135778253
**WNT5B**	8.44740438	7.450610945	4.82E-31	8.44740438	7.477104	9.76E-06	SECRETED_FACTORS	1.133786805	1.129769544
**WNT7B**	10.98979416	6.848191223	5.09E-149	10.98979416	9.483946	6.04E-10	SECRETED_FACTORS	1.604773261	1.158778652
**VWA9**	10.83498193	10.620444	5.59E-28	10.83498193	10.373318	5.93E-12	ECM_GLYCOPROTEINS	1.020200467	1.044504944
**VWDE**	6.359392518	4.057989055	1.44E-76	6.359392518	4.935474	6.46E-06	ECM_GLYCOPROTEINS	1.567129046	1.288506943
**XCL1**	6.157655474	3.996191618	2.88E-133	6.157655474	5.536504	0.000305562	SECRETED_FACTORS	1.540880935	1.112192003
**ZFP91**	11.88391241	11.64715431	1.34E-17	11.88391241	11.69422	0.001915432	SECRETED_FACTORS	1.020327549	1.01622104
**ZP3**	8.355566971	7.77258977	4.58E-19	8.355566971	6.171748	3.03E-27	ECM_GLYCOPROTEINS	1.075004242	1.353841241
**ZP4**	0.917193066	0.347357779	1.17E-34	0.917193066	0	0.000579378	ECM_GLYCOPROTEINS	2.640485175	Inf
**ZPLD1**	2.606076277	2.231629151	5.86E-05	2.606076277	1.097334	5.00E-09	ECM_GLYCOPROTEINS	1.167790928	2.374916185

Mesothelioma (MESO)

Signature genes	Expression in tumor	Expression in rest of Pan-Cancer	P-value	Expression in tumor	Expression in normal tissue	P-value	Matrisome category	Fold change vs. Pan-Cancer	Fold change vs. normal
**ADAM15**	12.71979885	12.1620167	1.20E-07	12.71979885	0.83795	3.56E-108	ECM_REGULATORS	1.045862636	15.17966329
**ADAM19**	9.450868966	9.009334678	0.010872967	9.450868966	4.969286364	4.37E-122	ECM_REGULATORS	1.049008534	1.901856378
**ADAM33**	8.609277011	6.07140516	1.88E-24	8.609277011	2.576570455	2.26E-11	ECM_REGULATORS	1.418004034	3.341370695
**ADAM8**	9.494810345	8.89876033	0.001737574	9.494810345	1.253972727	2.87E-35	ECM_REGULATORS	1.066981242	7.57178377
**ADAM9**	12.64007816	11.80909606	2.64E-09	12.64007816	4.8283375	1.43E-29	ECM_REGULATORS	1.070367968	2.617894495
**ADAMTS17**	7.317745977	6.470935793	1.27E-06	7.317745977	1.917002273	4.62E-100	ECM_REGULATORS	1.130863636	3.817286021
**ADAMTS2**	11.86501264	9.426232784	5.02E-29	11.86501264	7.958089773	9.91E-06	ECM_REGULATORS	1.258722643	1.49093727
**ADAMTS3**	9.860437931	5.811481354	4.18E-91	9.860437931	2.823254545	0.014977928	ECM_REGULATORS	1.69671678	3.492578431
**ADAMTS4**	9.555248276	9.125549438	0.034234549	9.555248276	0	0.012047317	ECM_REGULATORS	1.047087448	Inf
**ADAMTS7**	9.998666667	7.996091801	2.33E-35	9.998666667	1.104234091	5.26E-107	ECM_REGULATORS	1.250444206	9.054843306
**ADAMTSL1**	8.364321839	6.790864128	3.23E-13	8.364321839	1.164730682	2.32E-23	ECM_REGULATORS	1.231702134	7.181335539
**ADAMTSL4**	11.30261839	8.780658761	6.16E-40	11.30261839	0.429810227	7.70E-14	ECM_REGULATORS	1.287217588	26.29676465
**AEBP1**	14.00148276	12.78698765	7.08E-09	14.00148276	11.84688977	7.85E-153	ECM_GLYCOPROTEINS	1.094978985	1.181869928
**AGRN**	14.64297931	13.31695538	1.15E-21	14.64297931	5.316565909	1.63E-38	ECM_GLYCOPROTEINS	1.099574107	2.754217583
**AGRN**	14.64297931	13.31695538	1.15E-21	14.64297931	5.316565909	1.63E-38	BASEMENT_MEMBRANES	1.099574107	2.754217583
**ANGPTL1**	7.123282759	5.966124566	1.51E-05	7.123282759	0.705911364	6.83E-19	SECRETED_FACTORS	1.193954749	10.09090252
**ANGPTL2**	10.76874598	10.36563627	0.03009494	10.76874598	10.47743068	3.14E-71	SECRETED_FACTORS	1.038889046	1.027804078
**ANGPTL7**	3.823086207	2.6243956	2.35E-05	3.823086207	0.365079545	1.94E-93	SECRETED_FACTORS	1.456749206	10.47192661
**ANXA1**	13.83164138	12.57241633	1.79E-08	13.83164138	0.224142045	1.25E-43	ECM_AFFILIATED	1.10015776	61.70926722
**ANXA11**	13.52005057	12.81794992	2.47E-10	13.52005057	13.39536591	2.90E-05	ECM_AFFILIATED	1.054774801	1.009308045
**ANXA2**	16.29526437	14.47993711	1.55E-28	16.29526437	0.084206818	2.68E-109	ECM_AFFILIATED	1.125368449	193.5147856
**ANXA3**	12.32706667	9.217102772	6.43E-28	12.32706667	3.499820455	0.00044701	ECM_AFFILIATED	1.337412305	3.522199732
**ANXA4**	12.81105517	11.97875792	3.85E-08	12.81105517	4.551970455	4.37E-86	ECM_AFFILIATED	1.069481098	2.814397699
**ANXA5**	13.88917816	13.35678082	1.65E-06	13.88917816	0.076728409	8.46E-67	ECM_AFFILIATED	1.039859705	181.0174136
**ANXA8**	13.834	5.723261602	8.13E-65	13.834	8.312913636	1.41E-71	ECM_AFFILIATED	2.417153183	1.664157792
**ANXA8L1**	12.90776552	5.756162687	2.29E-66	12.90776552	11.30016023	1.95E-87	ECM_AFFILIATED	2.242425417	1.142263938
**ANXA9**	8.140203448	7.45056614	0.001683406	8.140203448	0.125560227	4.13E-104	ECM_AFFILIATED	1.092561732	64.83106653
**BDNF**	8.071434483	5.172303512	1.87E-38	8.071434483	2.182115909	0.000229552	SECRETED_FACTORS	1.560510605	3.698902725
**BGN**	15.54251954	13.17120109	7.92E-29	15.54251954	5.793145455	7.14E-49	PROTEOGLYCANS	1.180038133	2.682915467
**BMP1**	12.32157241	10.58460187	2.37E-51	12.32157241	9.4256625	1.30E-85	ECM_REGULATORS	1.164103531	1.30723675
**BMP3**	4.499948276	3.732376391	0.027513127	4.499948276	2.530544318	4.35E-64	SECRETED_FACTORS	1.205652326	1.778253099
**C1QA**	13.32276437	11.41637434	7.63E-25	13.32276437	9.521038636	2.63E-25	ECM_AFFILIATED	1.166987344	1.399297375
**C1QB**	13.32633678	11.58387838	7.86E-19	13.32633678	11.57160795	1.51E-29	ECM_AFFILIATED	1.150420985	1.151640881
**C1QC**	13.15924598	11.42031566	4.33E-20	13.15924598	6.343613636	7.00E-23	ECM_AFFILIATED	1.152266397	2.074408489
**C1QTNF1**	11.90692874	9.803957695	6.78E-31	11.90692874	1.214376136	4.59E-49	ECM_AFFILIATED	1.214502256	9.804975888
**CCBE1**	6.332402299	4.706428443	2.11E-09	6.332402299	1.862502273	5.34E-07	SECRETED_FACTORS	1.345479353	3.399943394
**CCL1**	0.976931034	0.637295008	0.002297226	0.976931034	0.614193182	8.17E-149	SECRETED_FACTORS	1.532933763	1.590592445
**CCL11**	5.831643678	4.038724862	5.62E-09	5.831643678	0.786252273	1.29E-43	SECRETED_FACTORS	1.443931904	7.417013445
**CCL13**	5.074767816	4.445460024	0.026335718	5.074767816	2.6249875	6.48E-86	SECRETED_FACTORS	1.141561906	1.933254088
**CCL14**	7.567655172	6.746117157	0.004187745	7.567655172	3.622790909	5.85E-32	SECRETED_FACTORS	1.121779388	2.088901999
**CCL2**	12.26028506	9.299596113	6.79E-44	12.26028506	11.58556477	6.97E-05	SECRETED_FACTORS	1.318367476	1.058238014

**CCL21**	8.128348276	6.936625582	0.002239826	8.128348276	5.462685227	1.07E-06	SECRETED_FACTORS	1.171801502	1.487976689
**CCL3**	8.845716092	7.421448589	1.29E-14	8.845716092	6.340792045	2.23E-76	SECRETED_FACTORS	1.191912332	1.395049077
**CCL4L2**	7.513467816	6.497752723	1.32E-06	7.513467816	7.208472727	3.80E-82	SECRETED_FACTORS	1.156317905	1.042310639
**CCL5**	10.42524598	9.371459816	1.38E-06	10.42524598	9.316761364	9.06E-33	SECRETED_FACTORS	1.112446319	1.118977461
**CD209**	7.3384	6.724245077	0.003663645	7.3384	4.470389773	0.005624516	ECM_AFFILIATED	1.091334405	1.641557084
**CILP**	9.709352874	6.909578285	1.24E-17	9.709352874	0.229773864	9.06E-46	ECM_GLYCOPROTEINS	1.405201949	42.256124
**CILP2**	9.42836092	6.291889828	5.25E-31	9.42836092	3.601670455	1.67E-48	ECM_GLYCOPROTEINS	1.49849428	2.617774457
**CLEC12B**	2.289172414	1.709411602	0.001166542	2.289172414	1.214805682	4.66E-15	ECM_AFFILIATED	1.339158112	1.884393898
**CLEC4A**	6.9565	6.127433465	9.38E-10	6.9565	0.693794318	2.85E-24	ECM_AFFILIATED	1.135304045	10.02674686
**COL10A1**	8.368842529	6.885885606	0.000183149	8.368842529	4.158871591	7.79E-26	COLLAGENS	1.21536183	2.01228683
**COL11A1**	11.38752299	7.376679647	8.49E-23	11.38752299	4.648151136	5.67E-100	COLLAGENS	1.543719334	2.449903769
**COL11A2**	6.44463908	5.977837806	0.04487258	6.44463908	3.157593182	4.22E-109	COLLAGENS	1.078088648	2.040997275
**COL12A1**	12.97835977	11.23249695	2.39E-10	12.97835977	6.947123864	2.76E-112	COLLAGENS	1.155429628	1.868163002
**COL13A1**	7.132173563	6.351330091	9.34E-05	7.132173563	4.500606818	0.001629923	COLLAGENS	1.122941724	1.584713762
**COL14A1**	11.99126322	9.908622593	4.46E-19	11.99126322	0.121203409	1.68E-63	COLLAGENS	1.210184676	98.93503251
**COL15A1**	11.0362977	10.43055367	0.010617429	11.0362977	7.035285227	3.76E-72	COLLAGENS	1.058074005	1.568706505
**COL15A1**	11.0362977	10.43055367	0.010617429	11.0362977	7.035285227	3.76E-72	BASEMENT_MEMBRANES	1.058074005	1.568706505
**COL16A1**	11.69893563	10.40352338	6.03E-10	11.69893563	2.086480682	1.74E-107	COLLAGENS	1.124516686	5.607018428
**COL18A1**	14.02407701	12.86523985	3.82E-13	14.02407701	4.852423864	4.68E-70	COLLAGENS	1.090075053	2.890117889
**COL18A1**	14.02407701	12.86523985	3.82E-13	14.02407701	4.852423864	4.68E-70	BASEMENT_MEMBRANES	1.090075053	2.890117889
**COL1A1**	18.11817471	14.62013411	5.34E-27	18.11817471	5.928756818	2.31E-145	COLLAGENS	1.239261869	3.0559821
**COL1A2**	17.09245057	14.38451312	4.69E-22	17.09245057	7.728581818	7.33E-73	COLLAGENS	1.188253675	2.211589523
**COL21A1**	6.592688506	6.098003966	0.036863303	6.592688506	5.823370455	8.86E-59	COLLAGENS	1.081122371	1.132108726
**COL3A1**	17.24964253	14.15126581	2.51E-23	17.24964253	4.069794318	3.19E-21	COLLAGENS	1.218946966	4.238455602
**COL4A2**	13.92467816	13.39872327	0.007080911	13.92467816	5.313918182	5.05E-11	COLLAGENS	1.039254105	2.620416364
**COL4A2**	13.92467816	13.39872327	0.007080911	13.92467816	5.313918182	5.05E-11	BASEMENT_MEMBRANES	1.039254105	2.620416364
**COL4A5**	11.00118851	9.299093587	9.38E-12	11.00118851	6.1588875	8.94E-25	COLLAGENS	1.183038799	1.786229819
**COL4A5**	11.00118851	9.299093587	9.38E-12	11.00118851	6.1588875	8.94E-25	BASEMENT_MEMBRANES	1.183038799	1.786229819
**COL4A6**	8.278477011	6.169130328	6.40E-11	8.278477011	4.933819318	2.11E-13	COLLAGENS	1.341919618	1.677904373
**COL4A6**	8.278477011	6.169130328	6.40E-11	8.278477011	4.933819318	2.11E-13	BASEMENT_MEMBRANES	1.341919618	1.677904373
**COL5A1**	14.43998736	11.71228787	2.38E-26	14.43998736	12.67630568	8.01E-47	COLLAGENS	1.232892115	1.139132151
**COL5A2**	13.6010931	11.41711507	1.21E-20	13.6010931	6.819744318	2.56E-95	COLLAGENS	1.191289833	1.994369945
**COL5A3**	10.30551494	9.395735251	3.53E-06	10.30551494	5.696418182	3.91E-39	COLLAGENS	1.096829005	1.80912191
**COL6A1**	15.76334943	13.11534005	4.42E-50	15.76334943	14.33658523	5.87E-75	BASEMENT_MEMBRANES	1.201901695	1.09951911
**COL6A1**	15.76334943	13.11534005	4.42E-50	15.76334943	14.33658523	5.87E-75	COLLAGENS	1.201901695	1.09951911
**COL6A2**	15.75881034	13.17268439	7.11E-34	15.75881034	11.82396818	4.08E-92	COLLAGENS	1.1963249	1.332785246
**COL6A2**	15.75881034	13.17268439	7.11E-34	15.75881034	11.82396818	4.08E-92	BASEMENT_MEMBRANES	1.1963249	1.332785246
**COL6A3**	14.27122414	12.53777336	2.69E-09	14.27122414	12.88514318	2.37E-55	COLLAGENS	1.138258264	1.107572026
**COL6A3**	14.27122414	12.53777336	2.69E-09	14.27122414	12.88514318	2.37E-55	BASEMENT_MEMBRANES	1.138258264	1.107572026
**COL8A1**	11.92575287	9.753171152	2.99E-14	11.92575287	10.4492	3.67E-49	COLLAGENS	1.222756444	1.141307744
**COMP**	9.762024138	7.228315785	4.43E-10	9.762024138	8.0084125	2.41E-95	ECM_GLYCOPROTEINS	1.35052541	1.218971193
**CRELD1**	10.73991609	10.24479204	1.97E-07	10.73991609	6.756992045	3.03E-67	ECM_GLYCOPROTEINS	1.048329342	1.589452233
**CRIM1**	13.66764713	11.35632036	1.01E-55	13.66764713	0.195836364	7.59E-190	ECM_GLYCOPROTEINS	1.203527788	69.79116071
**CRISPLD1**	9.351166667	8.091082074	3.19E-07	9.351166667	6.08975	2.12E-11	ECM_GLYCOPROTEINS	1.155737463	1.535558384
**CRLF1**	7.6224	7.088001401	0.01984563	7.6224	6.768884091	2.11E-92	SECRETED_FACTORS	1.075394821	1.126094035
**CSF1**	12.28516092	10.54940643	5.40E-38	12.28516092	1.706475	2.73E-05	SECRETED_FACTORS	1.164535749	7.199144974

**CST7**	7.777955172	7.028249852	0.000407107	7.777955172	5.448784091	1.44E-105	ECM_REGULATORS	1.106670272	1.427466209
**CTGF**	12.65613793	11.95918097	0.000838164	12.65613793	0.140071591	3.56E-19	ECM_GLYCOPROTEINS	1.058277984	90.35478107
**CTHRC1**	10.84582989	9.016966032	2.02E-11	10.84582989	7.952111364	1.29E-88	ECM_GLYCOPROTEINS	1.202824747	1.363893108
**CTSF**	11.42059425	10.89944704	0.001726588	11.42059425	4.0361375	6.26E-103	ECM_REGULATORS	1.047814097	2.829585031
**CTSK**	11.2007908	10.30976458	4.69E-05	11.2007908	1.001173864	4.17E-16	ECM_REGULATORS	1.086425467	11.18765802
**CTSL**	12.86513793	11.83880246	1.28E-14	12.86513793	0.687885227	2.70E-22	ECM_REGULATORS	1.086692508	18.70244835
**CTSZ**	13.53369425	12.82270553	1.95E-09	13.53369425	6.65405	5.59E-139	ECM_REGULATORS	1.055447637	2.0339033
**CXCL6**	6.151895402	4.550276884	5.00E-06	6.151895402	0.363067045	1.38E-50	SECRETED_FACTORS	1.35198265	16.94424068
**CYR61**	13.13840805	11.49950018	2.23E-14	13.13840805	10.858025	4.72E-27	ECM_GLYCOPROTEINS	1.142519922	1.210018217
**DCN**	14.29005747	11.96756789	2.19E-16	14.29005747	8.570943182	4.79E-125	PROTEOGLYCANS	1.194065294	1.667267787
**DDX26B**	9.298098851	8.155626825	2.01E-16	9.298098851	1.648797727	3.52E-93	ECM_GLYCOPROTEINS	1.140083902	5.639320516
**DPT**	8.139389655	6.713021369	6.08E-05	8.139389655	7.7318875	1.24E-70	ECM_GLYCOPROTEINS	1.212477841	1.052704098
**EBI3**	7.126397701	6.0419632	1.51E-10	7.126397701	1.269811364	9.26E-07	SECRETED_FACTORS	1.179483798	5.612170363
**ECM2**	9.315887356	8.08043748	5.10E-11	9.315887356	7.371207955	4.82E-100	ECM_GLYCOPROTEINS	1.152893934	1.263820993
**EDIL3**	10.83222414	9.241769376	3.95E-11	10.83222414	9.7754875	7.38E-14	ECM_GLYCOPROTEINS	1.172094184	1.108100659
**EFEMP1**	15.31970575	10.41146057	2.00E-83	15.31970575	7.888103409	3.94E-144	ECM_GLYCOPROTEINS	1.47142715	1.942127905
**EFEMP2**	12.85555402	10.38304768	3.93E-55	12.85555402	3.0062375	1.25E-81	ECM_GLYCOPROTEINS	1.238129152	4.276293547
**EGFLAM**	8.055070115	7.591237816	0.014406632	8.055070115	6.383727273	0.01132365	ECM_GLYCOPROTEINS	1.06110101	1.261813008
**EGLN1**	11.41983908	11.21746294	0.012667456	11.41983908	7.600998864	2.19E-90	ECM_REGULATORS	1.018041168	1.502412944
**EGLN2**	12.07905747	11.41629852	1.71E-20	12.07905747	9.702230682	1.64E-23	ECM_REGULATORS	1.058053751	1.244977353
**ELN**	11.20726782	9.982076421	3.58E-07	11.20726782	10.14081705	5.99E-71	ECM_GLYCOPROTEINS	1.122739132	1.105164186
**EPYC**	5.831597701	2.525688802	2.20E-28	5.831597701	1.913973864	5.18E-73	PROTEOGLYCANS	2.308913789	3.046853362
**F13A1**	11.38796437	9.151014296	1.38E-23	11.38796437	10.69367727	7.65E-47	ECM_REGULATORS	1.24444832	1.064925009
**F7**	4.745442529	3.88701325	0.007710956	4.745442529	0.098905682	3.29E-81	ECM_REGULATORS	1.220845473	47.97947339
**FAM20A**	10.9235092	8.5285566	4.73E-25	10.9235092	5.639498864	2.30E-40	ECM_REGULATORS	1.2808157	1.936964518
**FAM20B**	11.75446437	11.29748278	2.19E-11	11.75446437	1.810592045	7.04E-14	ECM_REGULATORS	1.040449859	6.492055677
**FAM20C**	11.55032069	10.6391928	2.78E-10	11.55032069	2.013855682	1.46E-114	ECM_REGULATORS	1.085638818	5.735426224
**FBLN1**	13.02265287	11.54336361	5.27E-09	13.02265287	5.178522727	1.61E-08	ECM_GLYCOPROTEINS	1.128150625	2.514742825
**FBLN5**	10.33012069	9.870161158	0.013194561	10.33012069	8.263398864	3.70E-130	ECM_GLYCOPROTEINS	1.046601015	1.250105539
**FCN1**	7.720111494	6.375246369	1.27E-08	7.720111494	4.508817045	2.34E-74	ECM_AFFILIATED	1.210951083	1.712225494
**FGF1**	7.973445977	7.14805734	0.002752051	7.973445977	4.498592045	7.26E-85	SECRETED_FACTORS	1.115470344	1.77243144
**FGF2**	9.142921839	7.775464473	8.54E-10	9.142921839	0.318039773	3.31E-93	SECRETED_FACTORS	1.175868255	28.74773102
**FGL2**	10.1488069	9.575530002	0.001971321	10.1488069	0.014686364	2.81E-35	ECM_GLYCOPROTEINS	1.059868947	691.0360623
**FMOD**	12.42850575	10.31520491	5.77E-17	12.42850575	6.794548864	2.10E-36	PROTEOGLYCANS	1.204872405	1.829187779
**FN1**	16.35575517	15.10008369	6.85E-07	16.35575517	3.210990909	5.44E-27	ECM_GLYCOPROTEINS	1.083156591	5.093678442
**FNDC1**	11.02737931	8.123307567	2.42E-18	11.02737931	0.142098864	1.70E-83	ECM_GLYCOPROTEINS	1.357498681	77.6035714
**FRAS1**	11.67537471	8.373052279	4.29E-35	11.67537471	8.400604545	9.67E-97	ECM_GLYCOPROTEINS	1.394398879	1.389825536
**GAS6**	13.57455517	11.69314578	1.55E-34	13.57455517	7.357082955	2.46E-184	ECM_GLYCOPROTEINS	1.160898481	1.845100192
**GPC6**	9.426437931	8.393325049	1.56E-05	9.426437931	2.636935227	6.17E-11	ECM_AFFILIATED	1.123087439	3.574770375
**HAPLN3**	9.346833333	8.507890854	1.20E-05	9.346833333	8.379421591	1.21E-05	PROTEOGLYCANS	1.098607574	1.115450897
**HSPG2**	13.77207356	13.33531073	0.005933004	13.77207356	3.715952273	6.69E-54	PROTEOGLYCANS	1.032752355	3.706203027
**HSPG2**	13.77207356	13.33531073	0.005933004	13.77207356	3.715952273	6.69E-54	BASEMENT_MEMBRANES	1.032752355	3.706203027
**HTRA1**	13.81848851	11.76162011	4.73E-25	13.81848851	4.689118182	1.65E-72	ECM_REGULATORS	1.174879683	2.9469269
**HTRA4**	5.671916092	3.38490515	1.70E-28	5.671916092	1.250257955	1.17E-62	ECM_REGULATORS	1.675649934	4.536596685
**IBSP**	4.599722989	3.259130357	1.93E-06	4.599722989	3.661544318	3.75E-09	ECM_GLYCOPROTEINS	1.411334462	1.256224857
**IGFBP2**	12.52543563	11.58790818	0.000202354	12.52543563	3.308595455	1.84E-36	ECM_GLYCOPROTEINS	1.080905668	3.785725938

**IGFBP4**	14.73150575	13.41177671	3.66E-12	14.73150575	12.13285568	2.39E-167	ECM_GLYCOPROTEINS	1.098400761	1.214182888
**IGFBP5**	14.08746782	13.2982347	0.001315802	14.08746782	0.933857955	2.22E-85	ECM_GLYCOPROTEINS	1.059348713	15.08523619
**ITIH3**	5.991982759	5.105618104	0.00363797	5.991982759	0.263054545	3.96E-46	ECM_REGULATORS	1.173605749	22.77848021
**ITLN1**	14.82886667	2.786346429	4.49E-293	14.82886667	10.01529432	4.30E-77	ECM_AFFILIATED	5.321975227	1.480622156
**ITLN2**	9.374154023	1.992356215	2.40E-184	9.374154023	2.077495455	3.55E-85	ECM_AFFILIATED	4.705059241	4.512238042
**LAMA4**	12.55048506	10.84677364	8.63E-21	12.55048506	4.796089773	2.04E-16	ECM_GLYCOPROTEINS	1.157070801	2.616816126
**LAMA4**	12.55048506	10.84677364	8.63E-21	12.55048506	4.796089773	2.04E-16	BASEMENT_MEMBRANES	1.157070801	2.616816126
**LAMA5**	13.50438276	12.33664821	1.76E-14	13.50438276	5.271243182	1.29E-63	BASEMENT_MEMBRANES	1.094655738	2.561897126
**LAMA5**	13.50438276	12.33664821	1.76E-14	13.50438276	5.271243182	1.29E-63	ECM_GLYCOPROTEINS	1.094655738	2.561897126
**LAMB1**	13.53588966	12.18350672	2.15E-14	13.53588966	6.506263636	1.60E-48	ECM_GLYCOPROTEINS	1.111001124	2.080439775
**LAMB1**	13.53588966	12.18350672	2.15E-14	13.53588966	6.506263636	1.60E-48	BASEMENT_MEMBRANES	1.111001124	2.080439775
**LAMB2**	13.49917586	12.68118825	9.29E-10	13.49917586	6.199969318	1.06E-42	BASEMENT_MEMBRANES	1.064504019	2.177297204
**LAMB2**	13.49917586	12.68118825	9.29E-10	13.49917586	6.199969318	1.06E-42	ECM_GLYCOPROTEINS	1.064504019	2.177297204
**LAMC1**	13.68217931	12.75481789	5.24E-11	13.68217931	1.8319125	1.98E-90	ECM_GLYCOPROTEINS	1.072706755	7.468795213
**LAMC1**	13.68217931	12.75481789	5.24E-11	13.68217931	1.8319125	1.98E-90	BASEMENT_MEMBRANES	1.072706755	7.468795213
**LAMC2**	11.86013908	10.1747368	6.32E-05	11.86013908	3.909002273	2.78E-39	ECM_GLYCOPROTEINS	1.165645787	3.034057863
**LAMC2**	11.86013908	10.1747368	6.32E-05	11.86013908	3.909002273	2.78E-39	BASEMENT_MEMBRANES	1.165645787	3.034057863
**LGALS1**	14.11412644	12.62419549	6.71E-24	14.11412644	9.332195455	1.17E-116	ECM_AFFILIATED	1.118021853	1.512412219
**LGALS12**	6.720454023	3.081854844	8.68E-48	6.720454023	0.125365909	8.96E-71	ECM_AFFILIATED	2.180652355	53.60671072
**LOX**	12.35414943	9.449353137	1.85E-38	12.35414943	1.688184091	1.35E-130	ECM_REGULATORS	1.307406893	7.318010809
**LOXL1**	11.35404023	9.147083573	1.45E-26	11.35404023	9.193948864	3.39E-151	ECM_REGULATORS	1.241274351	1.234947072
**LOXL2**	11.55769195	10.10354265	3.64E-15	11.55769195	1.558902273	7.57E-136	ECM_REGULATORS	1.143924696	7.413993909
**LOXL4**	8.863649425	7.786948204	1.68E-05	8.863649425	2.124920455	4.24E-207	ECM_REGULATORS	1.138269986	4.171285286
**LTBP2**	12.68084943	11.22602588	2.10E-12	12.68084943	6.822564773	7.59E-188	ECM_GLYCOPROTEINS	1.129593817	1.858663105
**LTBP3**	13.21348276	12.14308736	4.03E-16	13.21348276	9.146761364	2.53E-102	ECM_GLYCOPROTEINS	1.088148538	1.444607794
**LTBP4**	12.51565632	11.52975097	8.69E-10	12.51565632	1.134201136	1.97E-28	ECM_GLYCOPROTEINS	1.085509683	11.03477674
**LUM**	13.90188621	11.57994423	3.72E-12	13.90188621	10.18142727	2.77E-129	PROTEOGLYCANS	1.20051409	1.365416246
**MATN2**	11.84732069	10.27346922	7.09E-16	11.84732069	3.5017	1.74E-214	ECM_GLYCOPROTEINS	1.153195716	3.383305449
**MFAP2**	11.51305862	9.030276431	1.06E-17	11.51305862	1.199613636	4.29E-46	ECM_GLYCOPROTEINS	1.274939777	9.597305559
**MFAP4**	11.16551954	9.690583159	1.88E-08	11.16551954	5.583082955	1.16E-18	ECM_GLYCOPROTEINS	1.152203057	1.999884227
**MFAP5**	9.809078161	6.666390677	3.74E-19	9.809078161	7.282652273	2.10E-72	ECM_GLYCOPROTEINS	1.471422639	1.346910136
**MGP**	13.09517586	11.79634638	4.71E-06	13.09517586	9.700438636	4.87E-12	ECM_GLYCOPROTEINS	1.110104387	1.349957085
**MMP14**	14.65203678	12.86918534	1.56E-22	14.65203678	4.339971591	2.77E-66	ECM_REGULATORS	1.138536465	3.376067441
**MMP15**	11.65534713	10.81978352	2.77E-07	11.65534713	4.612923864	7.05E-103	ECM_REGULATORS	1.077225538	2.526672339
**MMP16**	7.988442529	6.777905624	1.41E-05	7.988442529	0.242132955	5.95E-79	ECM_REGULATORS	1.178600437	32.99196734
**MMP19**	9.386417241	7.922748925	1.65E-15	9.386417241	9.199939773	0.001024833	ECM_REGULATORS	1.184742484	1.020269423
**MMP2**	13.74835632	12.20003146	2.90E-13	13.74835632	11.88949318	2.24E-123	ECM_REGULATORS	1.126911546	1.156345028
**MMP23B**	8.9408	5.775458603	3.14E-63	8.9408	2.105089773	8.84E-05	ECM_REGULATORS	1.548067541	4.247229793
**MMP24**	11.48173793	6.957151529	1.13E-67	11.48173793	0.241529545	1.52E-119	ECM_REGULATORS	1.650350418	47.53761247
**MMRN2**	10.34814483	9.701514246	3.47E-05	10.34814483	4.095342045	2.55E-76	ECM_GLYCOPROTEINS	1.066652542	2.526808436
**MUC12**	6.228290805	5.014356679	0.000241394	6.228290805	3.365229545	6.76E-115	ECM_AFFILIATED	1.242091699	1.850777405
**MUC16**	9.814417241	5.366460468	2.95E-22	9.814417241	3.793965909	5.11E-12	ECM_AFFILIATED	1.828843667	2.586849085
**MXRA5**	13.37446552	10.98854985	1.62E-18	13.37446552	2.138310227	2.32E-22	ECM_GLYCOPROTEINS	1.217127437	6.254689028
**NGLY1**	10.21227471	9.973720018	0.000142784	10.21227471	7.918786364	5.30E-62	ECM_REGULATORS	1.023918327	1.289626244
**NID2**	9.585683908	9.147420373	0.033383755	9.585683908	7.775701136	5.06E-144	ECM_GLYCOPROTEINS	1.047911162	1.232774221
**NID2**	9.585683908	9.147420373	0.033383755	9.585683908	7.775701136	5.06E-144	BASEMENT_MEMBRANES	1.047911162	1.232774221

**NPNT**	12.00386552	10.22070788	7.51E-10	12.00386552	7.904952273	1.10E-14	BASEMENT_MEMBRANES	1.174465179	1.51852473
**NPNT**	12.00386552	10.22070788	7.51E-10	12.00386552	7.904952273	1.10E-14	ECM_GLYCOPROTEINS	1.174465179	1.51852473
**NTN1**	9.499257471	8.409457429	1.84E-05	9.499257471	9.140754545	1.38E-34	ECM_GLYCOPROTEINS	1.129592194	1.039220277
**NTN1**	9.499257471	8.409457429	1.84E-05	9.499257471	9.140754545	1.38E-34	BASEMENT_MEMBRANES	1.129592194	1.039220277
**NTNG1**	8.503358621	4.205837165	2.19E-41	8.503358621	2.505509091	1.48E-115	ECM_GLYCOPROTEINS	2.021799297	3.393864605
**NTNG1**	8.503358621	4.205837165	2.19E-41	8.503358621	2.505509091	1.48E-115	BASEMENT_MEMBRANES	2.021799297	3.393864605
**OGFOD1**	10.75006782	10.46929272	1.45E-07	10.75006782	2.750610227	4.89E-21	ECM_REGULATORS	1.026818917	3.908248326
**OGN**	10.37584138	6.087930929	1.94E-35	10.37584138	7.816123864	8.43E-22	PROTEOGLYCANS	1.704329681	1.327491933
**OIT3**	3.852302299	3.291471675	0.026687521	3.852302299	0.97745	6.62E-22	ECM_GLYCOPROTEINS	1.170389017	3.941175813
**P4HA1**	12.04572644	11.1256326	6.33E-18	12.04572644	11.69698409	9.18E-156	ECM_REGULATORS	1.082700362	1.029814723
**P4HTM**	10.83038621	10.31171631	0.000229574	10.83038621	9.048806818	8.07E-59	ECM_REGULATORS	1.050299085	1.196885559
**PCOLCE**	13.57034598	10.49021485	5.69E-63	13.57034598	0.029690909	0.00134824	ECM_GLYCOPROTEINS	1.293619451	457.0539061
**PLAU**	11.70922989	10.56399114	4.76E-07	11.70922989	11.31137727	5.73E-15	ECM_REGULATORS	1.108409665	1.035172782
**PLOD1**	12.85816782	12.48732833	0.000293399	12.85816782	11.82298409	4.19E-33	ECM_REGULATORS	1.029697264	1.087556891
**PLOD2**	13.60498276	11.12241915	1.63E-37	13.60498276	12.19945	4.93E-38	ECM_REGULATORS	1.223203565	1.115212797
**PLXDC2**	13.07348621	10.86537647	1.14E-37	13.07348621	0.061589773	2.40E-119	ECM_AFFILIATED	1.203224411	212.2671611
**PLXNA1**	12.09596322	11.50490271	1.83E-05	12.09596322	5.021021591	8.22E-18	ECM_AFFILIATED	1.051374663	2.409064171
**PLXNA2**	11.24911149	10.52806114	2.63E-07	11.24911149	3.247742045	2.87E-18	ECM_AFFILIATED	1.068488428	3.463671479
**PODN**	11.19254023	8.873205929	3.01E-22	11.19254023	8.389872727	1.45E-182	PROTEOGLYCANS	1.261386281	1.334053637
**PODNL1**	8.048456322	6.619169051	1.34E-09	8.048456322	4.977126136	2.30E-75	PROTEOGLYCANS	1.215931526	1.617089079
**POMZP3**	9.273391954	8.760404765	3.23E-07	9.273391954	1.451835227	3.05E-135	ECM_GLYCOPROTEINS	1.058557476	6.387358414
**POSTN**	12.90072414	10.78360275	1.59E-08	12.90072414	8.4119375	1.89E-79	ECM_GLYCOPROTEINS	1.196327835	1.533621016
**PRG2**	2.845818391	1.924254963	8.07E-06	2.845818391	0.027786364	5.15E-19	PROTEOGLYCANS	1.478919606	102.4178057
**PRG4**	13.71763908	4.920917463	2.07E-161	13.71763908	0.0715	2.05E-54	PROTEOGLYCANS	2.7876182	191.855092
**PXDN**	12.94000575	11.20015499	2.27E-19	12.94000575	0.502647727	3.58E-195	ECM_GLYCOPROTEINS	1.155341668	25.74368697
**RSPO1**	10.59541379	3.309434964	3.23E-137	10.59541379	1.812540909	9.86E-51	ECM_GLYCOPROTEINS	3.201577885	5.845613602
**RSPO2**	3.275208046	2.629827457	0.026511173	3.275208046	2.923955682	2.56E-08	ECM_GLYCOPROTEINS	1.245407959	1.120129168
**RSPO4**	5.333187356	4.21821029	0.000163598	5.333187356	1.403763636	3.21E-67	ECM_GLYCOPROTEINS	1.264324676	3.79920609
**SBSPON**	10.08631494	7.103059767	3.53E-37	10.08631494	8.265506818	2.85E-32	ECM_GLYCOPROTEINS	1.419995787	1.220289955
**SEMA3B**	12.44383678	9.540702269	1.95E-41	12.44383678	4.055819318	8.92E-50	ECM_AFFILIATED	1.304289394	3.068143772
**SEMA3C**	13.34428621	10.02105688	4.38E-33	13.34428621	0.316580682	1.87E-119	ECM_AFFILIATED	1.331624635	42.15129657
**SEMA3D**	7.090629885	6.29984337	0.004607781	7.090629885	1.392293182	3.55E-63	ECM_AFFILIATED	1.12552479	5.092770673
**SEMA4C**	11.23955517	10.76626554	2.03E-06	11.23955517	7.172706818	0.001276973	ECM_AFFILIATED	1.043960427	1.566989347
**SEMA4D**	10.94853908	10.46967319	5.50E-05	10.94853908	1.698551136	3.16E-103	ECM_AFFILIATED	1.04573838	6.445810695
**SEMA4F**	8.949625287	8.683718804	0.013778805	8.949625287	8.810028409	7.64E-97	ECM_AFFILIATED	1.030621268	1.015845225
**SEMA5A**	11.54457241	9.713686415	3.94E-17	11.54457241	6.311929545	1.41E-154	ECM_AFFILIATED	1.188485187	1.82900844
**SEMA6D**	8.458024138	7.753381778	0.002122948	8.458024138	2.064331818	1.30E-73	ECM_AFFILIATED	1.090881938	4.097221224
**SERPINB1**	12.05479195	11.57368539	0.002432743	12.05479195	6.3805125	6.64E-103	ECM_REGULATORS	1.041569003	1.889314057
**SERPINB6**	12.13586552	11.87827023	0.009026437	12.13586552	1.951163636	1.41E-21	ECM_REGULATORS	1.021686262	6.21980919
**SERPINB7**	7.31267931	3.192043903	2.89E-33	7.31267931	0.551915909	6.73E-05	ECM_REGULATORS	2.290908124	13.24962588
**SERPINB8**	9.453072414	8.783433268	2.86E-07	9.453072414	6.214178409	9.21E-13	ECM_REGULATORS	1.076238884	1.521210334
**SERPINB9**	10.92015747	9.2790309	5.37E-31	10.92015747	6.230551136	5.93E-26	ECM_REGULATORS	1.176864005	1.752679214
**SERPINE1**	12.62652414	10.72367076	2.38E-15	12.62652414	4.090814773	6.38E-53	ECM_REGULATORS	1.177444219	3.08655484
**SERPINE2**	11.42031609	10.72609572	0.005717899	11.42031609	8.017265909	2.67E-53	ECM_REGULATORS	1.06472256	1.424465176
**SERPING1**	14.91494138	12.87465213	6.11E-26	14.91494138	10.41882273	3.33E-87	ECM_REGULATORS	1.15847335	1.43153807
**SERPINH1**	14.72437011	12.62998523	3.13E-47	14.72437011	5.440107955	7.48E-80	ECM_REGULATORS	1.165826392	2.706631971

**SLIT2**	10.58404138	8.224777841	3.30E-24	10.58404138	3.686559091	6.52E-12	ECM_GLYCOPROTEINS	1.2868483	2.870981074
**SLIT3**	12.37143678	9.665665647	6.19E-28	12.37143678	1.751048864	0.001159739	ECM_GLYCOPROTEINS	1.279936347	7.06515794
**SMOC2**	11.02795057	9.329549546	3.65E-12	11.02795057	7.158918182	6.19E-90	ECM_GLYCOPROTEINS	1.182045341	1.54044931
**SPARC**	16.96448851	15.0976259	1.40E-27	16.96448851	6.178789773	3.13E-132	ECM_GLYCOPROTEINS	1.123652727	2.745600535
**SPOCK1**	10.04520345	9.29634631	0.012344158	10.04520345	0.083861364	3.11E-59	PROTEOGLYCANS	1.08055392	119.7834499
**SPOCK2**	14.0195977	10.6692275	3.92E-48	14.0195977	0.104305682	3.68E-40	PROTEOGLYCANS	1.314021817	134.4087633
**SPON1**	10.11785977	9.49423014	0.042295002	10.11785977	5.324669318	4.84E-59	ECM_GLYCOPROTEINS	1.065685118	1.900185564
**SPON2**	11.07854368	10.28166109	0.001107953	11.07854368	5.291855682	1.30E-11	ECM_GLYCOPROTEINS	1.077505238	2.093508278
**SRGN**	11.88916782	10.93118557	1.81E-09	11.88916782	3.626101136	3.61E-07	PROTEOGLYCANS	1.087637543	3.278774466
**SRPX**	9.232768966	8.084828522	1.83E-06	9.232768966	5.828085227	3.19E-23	ECM_GLYCOPROTEINS	1.141986987	1.584185647
**SRPX2**	8.36175977	7.652218054	0.003614801	8.36175977	0.447057955	5.98E-12	ECM_GLYCOPROTEINS	1.092723667	18.70397268
**SSPO**	7.347136782	5.560898905	5.40E-16	7.347136782	2.583748864	2.09E-36	ECM_GLYCOPROTEINS	1.321213873	2.843595554
**SULF1**	14.45171034	10.97815528	8.92E-40	14.45171034	7.992345455	1.65E-12	ECM_REGULATORS	1.316406079	1.808193906
**SULF2**	12.93065977	11.7754912	1.03E-10	12.93065977	8.803554545	1.11E-15	ECM_REGULATORS	1.098099396	1.46879987
**SVEP1**	10.48504483	8.154217453	4.15E-25	10.48504483	7.204344318	1.02E-16	ECM_GLYCOPROTEINS	1.285843171	1.455378084
**TGFBI**	14.87025057	12.24063272	1.07E-34	14.87025057	0.331004545	7.14E-82	ECM_GLYCOPROTEINS	1.214826956	44.9246114
**TGM1**	11.5793046	6.321878562	5.88E-69	11.5793046	11.41854091	0.000450036	ECM_REGULATORS	1.831624016	1.01407918
**THBS2**	14.00004483	11.08485729	1.10E-32	14.00004483	2.990764773	3.60E-190	ECM_GLYCOPROTEINS	1.262988279	4.681091925
**THBS3**	12.23914483	9.812401105	2.07E-113	12.23914483	5.484704545	2.01E-73	ECM_GLYCOPROTEINS	1.247313955	2.231504856
**TIMP1**	15.54036667	13.0121327	6.18E-42	15.54036667	10.69748068	4.13E-49	ECM_REGULATORS	1.194298201	1.452712758
**TIMP2**	15.08115057	13.25271185	1.20E-36	15.08115057	7.864988636	9.28E-89	ECM_REGULATORS	1.137967138	1.917504433
**TIMP3**	14.05314368	13.18876945	1.37E-05	14.05314368	10.76498864	1.72E-09	ECM_REGULATORS	1.065538657	1.305449003
**TNXB**	13.48152529	9.390344732	4.53E-87	13.48152529	9.466342045	9.41E-85	ECM_GLYCOPROTEINS	1.435679485	1.424153619
**VCAN**	13.09112414	11.61817348	5.05E-09	13.09112414	0.205842045	3.90E-13	PROTEOGLYCANS	1.126779882	63.59791125
**WNT10A**	9.30503908	6.003330702	3.90E-29	9.30503908	6.650735227	3.13E-16	SECRETED_FACTORS	1.549979427	1.399099312
**WNT2B**	11.87002759	7.527508613	1.18E-132	11.87002759	9.662820455	1.63E-14	SECRETED_FACTORS	1.576886616	1.228422658
**WNT3**	8.876863218	6.97752722	3.35E-25	8.876863218	0.864421591	4.05E-61	SECRETED_FACTORS	1.272207609	10.26913639
**WNT4**	8.857716092	7.011031117	1.12E-14	8.857716092	5.422438636	1.23E-83	SECRETED_FACTORS	1.263397059	1.633529983
**WNT7B**	8.170763218	7.311771892	0.026959202	8.170763218	1.833001136	3.92E-13	SECRETED_FACTORS	1.117480597	4.457587645
**WNT9A**	9.501265517	6.908381876	1.02E-28	9.501265517	1.376176136	1.21E-60	SECRETED_FACTORS	1.375324307	6.904105707
**VTN**	11.91651954	5.223164966	2.63E-68	11.91651954	5.340589773	3.01E-17	ECM_GLYCOPROTEINS	2.281474856	2.231311531
**XCL2**	5.018388506	3.469792591	6.87E-15	5.018388506	0.949342045	5.17E-08	SECRETED_FACTORS	1.44630792	5.286175336
**ZP3**	8.422656322	7.805067729	0.000105855	8.422656322	6.596473864	2.11E-11	ECM_GLYCOPROTEINS	1.079126615	1.276842218
**ZPLD1**	3.164602299	2.273493173	9.35E-05	3.164602299	1.234010227	8.26E-118	ECM_GLYCOPROTEINS	1.391955928	2.564486281

Ovarian serous cystadenocarcinoma (OV)

Signature genes	Expression in tumor	Expression in rest of Pan-Cancer	P-value	Expression in tumor	Expression in normal tissue	P-value	Matrisome category	Fold change vs. Pan-Cancer	Fold change vs. normal
**ADAM15**	12.48514379	12.14891079	2.95E-12	12.48514379	0.83795	7.50E-187	ECM_REGULATORS	1.02767598	14.89962861
**ADAM28**	8.990647073	7.972306027	3.06E-26	8.990647073	0.294375	4.94E-145	ECM_REGULATORS	1.127734816	30.54147626
**ADAM32**	6.490762061	4.516453379	7.39E-121	6.490762061	3.996519318	7.40E-19	ECM_REGULATORS	1.437136956	1.624103762
**ADAM33**	7.501688993	6.098849371	3.55E-33	7.501688993	2.576570455	5.03E-45	ECM_REGULATORS	1.230017096	2.911501597
**ADAM8**	9.895855738	8.835672108	1.31E-33	9.895855738	1.253972727	1.04E-36	ECM_REGULATORS	1.119989019	7.891603639
**ADAMTS17**	7.097877986	6.469939052	5.79E-15	7.097877986	1.917002273	3.35E-15	ECM_REGULATORS	1.097054845	3.702592369
**ADAMTS3**	6.319719438	5.853245323	5.66E-07	6.319719438	2.823254545	2.66E-39	ECM_REGULATORS	1.079694954	2.238451878
**ADAMTS6**	5.994201405	5.745067502	0.004412245	5.994201405	0	5.32E-07	ECM_REGULATORS	1.043364835	Inf
**ADAMTS7**	8.4660363	8.034459517	1.29E-08	8.4660363	1.104234091	5.44E-205	ECM_REGULATORS	1.053715721	7.666885463
**ADAMTS9**	9.294612412	8.977638991	0.000394246	9.294612412	5.987080682	0.017371379	ECM_REGULATORS	1.035306991	1.552444823
**AGRN**	14.71028829	13.22559015	3.64E-120	14.71028829	5.316565909	6.31E-220	BASEMENT_MEMBRANES	1.1122595	2.76687782
**AGRN**	14.71028829	13.22559015	3.64E-120	14.71028829	5.316565909	6.31E-220	ECM_GLYCOPROTEINS	1.1122595	2.76687782
**AMH**	5.300237471	4.810524026	4.11E-05	5.300237471	0.357515909	7.70E-110	SECRETED_FACTORS	1.101800436	14.82517934
**ANXA1**	12.8387733	12.58123836	0.012056242	12.8387733	0.224142045	4.86E-06	ECM_AFFILIATED	1.020469761	57.27962942
**ANXA11**	13.72866932	12.7730452	3.64E-80	13.72866932	13.39536591	3.51E-93	ECM_AFFILIATED	1.074815685	1.024881994
**ANXA13**	4.642310773	3.967160166	5.07E-05	4.642310773	0.0342625	7.55E-284	ECM_AFFILIATED	1.170184862	135.4924706
**ANXA3**	10.84480375	9.076169795	1.75E-40	10.84480375	3.499820455	3.36E-16	ECM_AFFILIATED	1.194865675	3.098674314
**ANXA9**	7.895020609	7.391378034	7.39E-07	7.895020609	0.125560227	1.30E-113	ECM_AFFILIATED	1.068139199	62.87835551
**ARTN**	7.537910539	5.393294866	2.99E-90	7.537910539	0.296636364	1.18E-136	SECRETED_FACTORS	1.397644803	25.41128285
**ASTL**	4.293822482	1.822395333	1.50E-245	4.293822482	3.903072727	1.64e-318	ECM_REGULATORS	2.356142164	1.100113368
**BGN**	13.58052763	13.22805289	0.000364603	13.58052763	5.793145455	4.31E-116	PROTEOGLYCANS	1.026646003	2.344240748
**BMP1**	10.92875902	10.62568557	3.27E-08	10.92875902	9.4256625	4.66E-194	ECM_REGULATORS	1.028522719	1.159468527
**BMP3**	4.07574918	3.637397748	0.005978949	4.07574918	2.530544318	2.76E-137	SECRETED_FACTORS	1.120512373	1.610621537
**BMP7**	10.76211382	7.891825122	2.08E-54	10.76211382	8.190473864	3.19E-187	SECRETED_FACTORS	1.363704042	1.313979386
**BTC**	8.428602342	6.360064255	8.78E-72	8.428602342	1.7589625	3.00E-56	SECRETED_FACTORS	1.325238552	4.791803317
**C1QA**	11.7279555	11.46953003	0.002884938	11.7279555	9.521038636	6.10E-87	ECM_AFFILIATED	1.022531479	1.23179371
**C1QB**	12.45649087	11.60875435	1.54E-20	12.45649087	11.57160795	7.85E-13	ECM_AFFILIATED	1.073025623	1.076470177
**C1QC**	12.39126581	11.44195078	3.62E-27	12.39126581	6.343613636	1.04E-59	ECM_AFFILIATED	1.082967935	1.953344973
**C1QTNF3**	8.530044262	7.913812125	1.45E-10	8.530044262	2.171843182	1.58E-17	ECM_AFFILIATED	1.077867926	3.927559933
**C1QTNF9**	2.91544637	2.234241751	2.19E-17	2.91544637	2.696727273	6.52E-45	ECM_AFFILIATED	1.304892977	1.081105383
**CCBE1**	5.069017564	4.744636242	0.009695747	5.069017564	1.862502273	1.82E-45	SECRETED_FACTORS	1.068368007	2.721616848
**CCL11**	5.451006557	3.992449848	5.45E-25	5.451006557	0.786252273	2.56E-65	SECRETED_FACTORS	1.365328749	6.93289768
**CCL20**	6.977606792	5.934170637	6.14E-10	6.977606792	5.353228409	5.87E-76	SECRETED_FACTORS	1.175835212	1.30343902
**CCL28**	10.06684098	7.860008797	9.50E-80	10.06684098	9.537213636	7.34E-209	SECRETED_FACTORS	1.28076714	1.055532713
**CCL3**	7.773451756	7.42891946	5.43E-05	7.773451756	6.340792045	1.97E-199	SECRETED_FACTORS	1.046377175	1.225943337
**CILP2**	9.421063934	6.188143638	3.87E-150	9.421063934	3.601670455	6.14E-106	ECM_GLYCOPROTEINS	1.522437824	2.615748457
**CLCF1**	10.0505096	7.586128267	7.16E-179	10.0505096	7.252020455	1.09E-71	SECRETED_FACTORS	1.324853634	1.385890962
**CLEC11A**	10.35459321	8.441903297	2.47E-158	10.35459321	4.235623864	3.84E-84	ECM_AFFILIATED	1.226570934	2.444644176
**CLEC18C**	4.881362295	3.382231341	7.51E-30	4.881362295	4.195159091	3.03E-115	ECM_AFFILIATED	1.44323726	1.163570246
**CLEC4E**	5.711822482	5.393684253	0.000880143	5.711822482	4.371936364	2.58E-42	ECM_AFFILIATED	1.058983473	1.306474296
**COL10A1**	8.56577096	6.825529769	1.19E-21	8.56577096	4.158871591	6.09E-79	COLLAGENS	1.254960604	2.059638239
**COL11A1**	9.036805855	7.396753967	3.54E-18	9.036805855	4.648151136	1.75E-181	COLLAGENS	1.221725894	1.944172121

**COL11A2**	6.672815691	5.946932295	1.28E-11	6.672815691	3.157593182	1.52E-164	COLLAGENS	1.122060141	2.113260102
**COL12A1**	12.09792014	11.25151982	3.06E-11	12.09792014	6.947123864	5.02E-277	COLLAGENS	1.075225422	1.741428594
**COL13A1**	7.876257143	6.311790513	1.84E-64	7.876257143	4.500606818	2.56E-17	COLLAGENS	1.247864156	1.750043374
**COL18A1**	13.93399906	12.81815479	3.27E-53	13.93399906	4.852423864	4.53E-101	COLLAGENS	1.087051865	2.871554393
**COL18A1**	13.93399906	12.81815479	3.27E-53	13.93399906	4.852423864	4.53E-101	BASEMENT_MEMBRANES	1.087051865	2.871554393
**COL1A1**	16.64154965	14.65339649	2.67E-39	16.64154965	5.928756818	1.19E-213	COLLAGENS	1.135678657	2.806920601
**COL1A2**	15.60984754	14.44426474	7.72E-19	15.60984754	7.728581818	2.30E-108	COLLAGENS	1.080695198	2.019755747
**COL25A1**	5.066418735	3.819856331	9.31E-27	5.066418735	3.960978409	1.30E-81	COLLAGENS	1.32633751	1.279082644
**COL3A1**	14.88629157	14.24504373	1.09E-05	14.88629157	4.069794318	0.000299101	COLLAGENS	1.045015505	3.657750344
**COL4A5**	9.838145667	9.243899087	3.77E-07	9.838145667	6.1588875	1.10E-42	BASEMENT_MEMBRANES	1.064285273	1.597390059
**COL4A5**	9.838145667	9.243899087	3.77E-07	9.838145667	6.1588875	1.10E-42	COLLAGENS	1.064285273	1.597390059
**COL6A2**	13.69983934	13.26451559	1.80E-05	13.69983934	11.82396818	5.83E-199	COLLAGENS	1.032818669	1.158649882
**COL6A2**	13.69983934	13.26451559	1.80E-05	13.69983934	11.82396818	5.83E-199	BASEMENT_MEMBRANES	1.032818669	1.158649882
**COL6A3**	13.04769977	12.61917087	0.001570708	13.04769977	12.88514318	7.01E-138	COLLAGENS	1.033958562	1.012615815
**COL6A3**	13.04769977	12.61917087	0.001570708	13.04769977	12.88514318	7.01E-138	BASEMENT_MEMBRANES	1.033958562	1.012615815
**COL9A2**	10.4104548	8.640044349	2.13E-64	10.4104548	9.830579545	2.42E-35	COLLAGENS	1.204907565	1.058986884
**COMP**	8.877429274	7.174488322	1.04E-19	8.877429274	8.0084125	1.85E-241	ECM_GLYCOPROTEINS	1.237360614	1.108512989
**CPAMD8**	9.748430445	7.514284355	1.44E-85	9.748430445	3.203618182	2.18E-212	ECM_REGULATORS	1.297319876	3.042943913
**CRELD1**	10.35396136	10.23590371	0.007239613	10.35396136	6.756992045	2.00E-139	ECM_GLYCOPROTEINS	1.011533681	1.532332921
**CSF1**	11.84504098	10.53760739	2.40E-97	11.84504098	1.706475	0.0190492	SECRETED_FACTORS	1.124073098	6.941233234
**CSH1**	0.407759953	0.227973823	2.10E-05	0.407759953	0.2683125	1.92E-68	SECRETED_FACTORS	1.788626201	1.519720301
**CST7**	7.279537237	7.026111891	0.009672354	7.279537237	5.448784091	6.40E-233	ECM_REGULATORS	1.036069073	1.335992969
**CSTL1**	1.240477986	0.550957741	4.73E-46	1.240477986	1.133457955	2.39E-200	ECM_REGULATORS	2.251493885	1.094419057
**CTGF**	12.25030351	11.970925	0.003653573	12.25030351	0.140071591	1.66E-06	ECM_GLYCOPROTEINS	1.023338089	87.45744539
**CTHRC1**	10.50398337	9.014697656	2.90E-32	10.50398337	7.952111364	5.82E-133	ECM_GLYCOPROTEINS	1.165206397	1.320904964
**CTSA**	13.02873607	12.80799154	1.93E-06	13.02873607	0.294693182	5.18E-86	ECM_REGULATORS	1.017234906	44.21118936
**CTSB**	15.42844309	15.22466902	0.001114148	15.42844309	10.11695114	5.66E-219	ECM_REGULATORS	1.013384466	1.525009154
**CTSF**	11.82877073	10.87263265	1.64E-36	11.82877073	4.0361375	7.97E-232	ECM_REGULATORS	1.087939886	2.930715499
**CXCL3**	5.973495785	5.303557102	3.00E-07	5.973495785	4.897975	0.010572939	SECRETED_FACTORS	1.126318746	1.219584784
**CXCL6**	5.054540281	4.524416213	0.001015238	5.054540281	0.363067045	4.95E-134	SECRETED_FACTORS	1.117169607	13.92178206
**CXCL8**	8.522983138	8.017859619	0.000315103	8.522983138	7.288136364	1.38E-72	SECRETED_FACTORS	1.062999796	1.169432447
**CYR61**	11.85251686	11.52534416	0.000940609	11.85251686	10.858025	4.26E-91	ECM_GLYCOPROTEINS	1.02838724	1.091590493
**DCN**	12.79317119	12.01249609	3.77E-09	12.79317119	8.570943182	3.72E-290	PROTEOGLYCANS	1.064988583	1.492621165
**DDX26B**	8.360422717	8.162516518	0.001945433	8.360422717	1.648797727	5.87E-103	ECM_GLYCOPROTEINS	1.024245733	5.070617565
**EFEMP2**	11.7654459	10.38452637	1.84E-79	11.7654459	3.0062375	3.64E-110	ECM_GLYCOPROTEINS	1.132978576	3.913678111
**EGFL8**	7.777307728	7.638513687	0.038435086	7.777307728	7.413967045	5.62E-41	SECRETED_FACTORS	1.018170294	1.049007593
**EGFLAM**	8.494968618	7.595239631	8.18E-25	8.494968618	6.383727273	4.08E-23	ECM_GLYCOPROTEINS	1.118459592	1.330722359
**EGLN1**	11.35399461	11.20512424	6.46E-05	11.35399461	7.600998864	2.53E-66	ECM_REGULATORS	1.013285919	1.493750337
**EMID1**	10.46482108	7.99631111	9.13E-171	10.46482108	8.202040909	1.74E-161	ECM_GLYCOPROTEINS	1.308706094	1.275880137
**EPYC**	5.904547775	2.419148752	4.47E-144	5.904547775	1.913973864	7.57E-109	PROTEOGLYCANS	2.440754323	3.084967819
**ESM1**	8.079577752	7.674007447	0.001203474	8.079577752	6.248171591	1.16E-127	PROTEOGLYCANS	1.052849871	1.293110734
**FAM20A**	10.39689578	8.4744243	2.12E-73	10.39689578	5.639498864	1.07E-126	ECM_REGULATORS	1.226855703	1.843585048
**FAM20C**	10.86873934	10.67585045	0.003956992	10.86873934	2.013855682	3.19E-166	ECM_REGULATORS	1.018067778	5.396980252
**FBLN1**	12.29278103	11.54563504	1.60E-10	12.29278103	5.178522727	2.30E-68	ECM_GLYCOPROTEINS	1.06471242	2.37380073
**FBLN5**	10.73922108	9.85804377	6.85E-25	10.73922108	8.263398864	5.86E-274	ECM_GLYCOPROTEINS	1.08938663	1.299613059
**FBN3**	8.621191335	4.011001157	1.09E-167	8.621191335	5.654632955	2.92E-119	ECM_GLYCOPROTEINS	2.1493864	1.524624393

**FCN1**	6.726763232	6.401334771	0.002908352	6.726763232	4.508817045	2.33E-222	ECM_AFFILIATED	1.050837594	1.491913104
**FGF12**	7.513144965	6.254173793	5.78E-27	7.513144965	2.663564773	5.42E-143	SECRETED_FACTORS	1.201300957	2.820710441
**FGF17**	4.41496534	3.041329129	4.98E-37	4.41496534	3.023728409	8.10E-11	SECRETED_FACTORS	1.451656546	1.460106446
**FGF3**	1.912965105	0.352845323	1.40E-128	1.912965105	0.704777273	1.38E-245	SECRETED_FACTORS	5.421540212	2.714283192
**FMOD**	11.88809859	10.27177107	1.37E-43	11.88809859	6.794548864	3.77E-75	PROTEOGLYCANS	1.157356264	1.749652381
**FNDC1**	9.600284778	8.140779241	2.93E-21	9.600284778	0.142098864	4.48E-211	ECM_GLYCOPROTEINS	1.179283272	67.56060205
**FRAS1**	9.537160422	8.321466934	5.05E-23	9.537160422	8.400604545	1.42E-169	ECM_GLYCOPROTEINS	1.146091248	1.135294534
**GAS6**	13.41380141	11.6797751	3.07E-136	13.41380141	7.357082955	0	ECM_GLYCOPROTEINS	1.148464015	1.823249988
**GPC4**	10.34589438	9.904581849	5.16E-05	10.34589438	0.904875	8.01E-57	ECM_AFFILIATED	1.044556402	11.4335067
**GPC5**	4.01459274	2.882405702	1.18E-19	4.01459274	2.522506818	2.78E-293	ECM_AFFILIATED	1.392792395	1.591509173
**HAPLN1**	6.266915457	5.175917735	2.16E-13	6.266915457	0.216446591	7.07E-48	PROTEOGLYCANS	1.210783435	28.95363438
**HAPLN3**	9.951475176	8.43607989	6.45E-69	9.951475176	8.379421591	6.96E-14	PROTEOGLYCANS	1.179632638	1.187608842
**HSPG2**	13.8345363	13.3520807	5.08E-11	13.8345363	3.715952273	1.25E-96	BASEMENT_MEMBRANES	1.036133364	3.723012376
**HSPG2**	13.8345363	13.3520807	5.08E-11	13.8345363	3.715952273	1.25E-96	PROTEOGLYCANS	1.036133364	3.723012376
**HYAL2**	11.67794941	11.12761848	2.94E-45	11.67794941	1.068781818	1.87E-110	ECM_REGULATORS	1.049456309	10.926411
**HYAL3**	8.296868852	7.050463809	1.83E-140	8.296868852	0.793639773	3.69E-168	ECM_REGULATORS	1.176783411	10.45419992
**IBSP**	5.127900703	3.200376563	2.15E-49	5.127900703	3.661544318	3.34E-22	ECM_GLYCOPROTEINS	1.60228042	1.400474843
**IGFBP2**	14.77849578	11.38359689	6.42E-197	14.77849578	3.308595455	2.26E-125	ECM_GLYCOPROTEINS	1.298227259	4.466697723
**IGFBP7**	13.84322201	13.38459881	4.92E-13	13.84322201	3.753894318	8.33E-66	ECM_GLYCOPROTEINS	1.034264994	3.687696254
**IGSF10**	8.022576347	5.921321195	3.41E-62	8.022576347	6.493828409	2.43E-51	ECM_GLYCOPROTEINS	1.354862552	1.235415512
**IMPG2**	7.325644496	3.689035166	0	7.325644496	0.720606818	3.48E-94	PROTEOGLYCANS	1.98578874	10.16593836
**ITIH5**	9.616670492	9.139512622	1.88E-05	9.616670492	6.149601136	1.49E-89	ECM_REGULATORS	1.05220824	1.56378768
**ITIH6**	3.217039344	2.710970495	0.000450531	3.217039344	2.437770455	2.48E-220	ECM_REGULATORS	1.186674422	1.319664589
**KAZALD1**	8.061175644	7.345047088	7.37E-17	8.061175644	4.251143182	8.24E-50	ECM_REGULATORS	1.097498157	1.896237153
**KY**	3.736312178	3.150563931	1.14E-07	3.736312178	2.282142045	3.45E-210	ECM_REGULATORS	1.18591854	1.637195277
**LAMA5**	14.21761265	12.23349818	2.30E-181	14.21761265	5.271243182	1.32E-117	BASEMENT_MEMBRANES	1.162187008	2.697202947
**LAMA5**	14.21761265	12.23349818	2.30E-181	14.21761265	5.271243182	1.32E-117	ECM_GLYCOPROTEINS	1.162187008	2.697202947
**LAMB2**	13.05291335	12.69586931	8.37E-09	13.05291335	6.199969318	3.90E-109	ECM_GLYCOPROTEINS	1.028122851	2.105319023
**LAMB2**	13.05291335	12.69586931	8.37E-09	13.05291335	6.199969318	3.90E-109	BASEMENT_MEMBRANES	1.028122851	2.105319023
**LAMB3**	11.2414377	10.80988009	0.002379016	11.2414377	5.342407955	0.021209709	BASEMENT_MEMBRANES	1.039922516	2.104189309
**LAMB3**	11.2414377	10.80988009	0.002379016	11.2414377	5.342407955	0.021209709	ECM_GLYCOPROTEINS	1.039922516	2.104189309
**LAMC1**	13.46229813	12.75514809	2.62E-27	13.46229813	1.8319125	1.93E-155	BASEMENT_MEMBRANES	1.055440362	7.348767
**LAMC1**	13.46229813	12.75514809	2.62E-27	13.46229813	1.8319125	1.93E-155	ECM_GLYCOPROTEINS	1.055440362	7.348767
**LAMC2**	12.14237049	9.999060248	1.48E-28	12.14237049	3.909002273	4.81E-64	ECM_GLYCOPROTEINS	1.214351168	3.106258233
**LAMC2**	12.14237049	9.999060248	1.48E-28	12.14237049	3.909002273	4.81E-64	BASEMENT_MEMBRANES	1.214351168	3.106258233
**LAMC3**	8.385036066	7.744194998	3.52E-10	8.385036066	5.444	5.32E-10	ECM_GLYCOPROTEINS	1.082751153	1.540234399
**LAMC3**	8.385036066	7.744194998	3.52E-10	8.385036066	5.444	5.32E-10	BASEMENT_MEMBRANES	1.082751153	1.540234399
**LGALS1**	13.08060422	12.6650971	2.89E-09	13.08060422	9.332195455	4.32E-208	ECM_AFFILIATED	1.032807259	1.401664194
**LGALS9**	11.9534096	10.8248943	4.06E-55	11.9534096	8.188831818	2.77E-32	ECM_AFFILIATED	1.104251854	1.459720979
**LOX**	9.938874941	9.511695292	4.31E-05	9.938874941	1.688184091	1.16e-315	ECM_REGULATORS	1.04491099	5.887317026
**LOXL1**	10.98252319	9.145221246	5.24E-80	10.98252319	9.193948864	8.00e-314	ECM_REGULATORS	1.200902951	1.194538206
**LOXL4**	8.863853396	7.771169947	1.96E-21	8.863853396	2.124920455	7.34E-248	ECM_REGULATORS	1.140607329	4.171381275
**LRG1**	8.839179625	8.301315179	0.00027813	8.839179625	0.161159091	5.04E-128	ECM_GLYCOPROTEINS	1.064792679	54.84753963
**LTBP2**	12.37901124	11.20731689	8.13E-35	12.37901124	6.822564773	7.90e-323	ECM_GLYCOPROTEINS	1.104547267	1.814421944
**LTBP3**	13.44267728	12.11826726	2.81E-108	13.44267728	9.146761364	1.37E-161	ECM_GLYCOPROTEINS	1.109290379	1.469665245
**LTBP4**	12.11983513	11.5422021	6.88E-15	12.11983513	1.134201136	2.58E-06	ECM_GLYCOPROTEINS	1.050045305	10.68578997

**MATN2**	10.86944379	10.28373041	7.84E-11	10.86944379	3.5017	0	ECM_GLYCOPROTEINS	1.056955342	3.104047689
**MFAP2**	11.74334918	8.950824838	3.18E-99	11.74334918	1.199613636	2.37E-56	ECM_GLYCOPROTEINS	1.31198514	9.789276167
**MFAP5**	9.402893208	6.656987033	9.81E-63	9.402893208	7.282652273	1.26E-216	ECM_GLYCOPROTEINS	1.412484832	1.291135819
**MFGE8**	13.30270937	11.69517655	8.41E-111	13.30270937	9.285703409	5.37E-268	ECM_GLYCOPROTEINS	1.137452633	1.432601148
**MMP11**	11.54939953	9.873782752	6.74E-33	11.54939953	1.070275	1.03E-17	ECM_REGULATORS	1.169703631	10.79105794
**MMP15**	11.29674473	10.75782345	8.90E-13	11.29674473	4.612923864	3.96E-206	ECM_REGULATORS	1.050095755	2.448933706
**MMP2**	12.66078244	12.24848089	3.70E-05	12.66078244	11.88949318	0	ECM_REGULATORS	1.033661443	1.0648715
**MMP23B**	8.574217564	5.699775852	1.28E-243	8.574217564	2.105089773	4.17E-11	ECM_REGULATORS	1.504307851	4.073088794
**MMP24**	8.38033185	6.904115077	1.18E-34	8.38033185	0.241529545	5.77E-284	ECM_REGULATORS	1.213816942	34.69692221
**MMP7**	11.56676323	8.038498569	2.52E-70	11.56676323	11.35431364	1.54E-207	ECM_REGULATORS	1.438920855	1.018710915
**MUC1**	14.39931756	10.67021314	1.38E-118	14.39931756	4.323046591	1.54E-108	ECM_AFFILIATED	1.349487342	3.330826366
**MUC15**	6.271611007	4.596114418	9.74E-17	6.271611007	4.302838636	3.57E-25	ECM_AFFILIATED	1.364546318	1.457551988
**MUC16**	14.49132834	4.797925548	0	14.49132834	3.793965909	1.13E-33	ECM_AFFILIATED	3.020332057	3.819572628
**MUC20**	11.46670867	8.894625619	2.39E-88	11.46670867	5.420503409	2.26E-278	ECM_AFFILIATED	1.289172716	2.115432424
**MUC4**	8.354538642	6.646361627	3.60E-19	8.354538642	6.610771591	1.49E-51	ECM_AFFILIATED	1.25700934	1.26377663
**MUC5AC**	3.784044731	3.154161932	0.002186994	3.784044731	3.622044318	0.001068024	ECM_AFFILIATED	1.199698941	1.044726237
**MUC5B**	8.774392037	5.923638474	7.67E-37	8.774392037	0.0289625	1.32E-58	ECM_AFFILIATED	1.48125043	302.9569974
**MXRA5**	12.50287166	10.98856956	2.74E-33	12.50287166	2.138310227	1.17E-29	ECM_GLYCOPROTEINS	1.137807027	5.847080327
**NGLY1**	10.28084169	9.957309619	2.13E-29	10.28084169	7.918786364	2.14E-109	ECM_REGULATORS	1.032491916	1.298285017
**NOV**	9.136040749	7.974512064	7.89E-33	9.136040749	8.679488636	1.79E-138	ECM_GLYCOPROTEINS	1.145655142	1.052601269
**NTN1**	10.05322623	8.348553653	5.94E-48	10.05322623	9.140754545	1.16E-06	BASEMENT_MEMBRANES	1.204187773	1.099824547
**NTN1**	10.05322623	8.348553653	5.94E-48	10.05322623	9.140754545	1.16E-06	ECM_GLYCOPROTEINS	1.204187773	1.099824547
**NTN4**	10.05788361	9.74494795	0.002660868	10.05788361	0.432769318	1.71E-66	BASEMENT_MEMBRANES	1.032112604	23.24075017
**NTN4**	10.05788361	9.74494795	0.002660868	10.05788361	0.432769318	1.71E-66	ECM_GLYCOPROTEINS	1.032112604	23.24075017
**NTN5**	4.996707963	3.628190138	1.67E-71	4.996707963	2.335186364	3.88E-17	ECM_GLYCOPROTEINS	1.377190217	2.139746977
**NTN5**	4.996707963	3.628190138	1.67E-71	4.996707963	2.335186364	3.88E-17	BASEMENT_MEMBRANES	1.377190217	2.139746977
**NTNG1**	5.248816628	4.263854495	3.47E-11	5.248816628	2.505509091	2.39E-207	BASEMENT_MEMBRANES	1.231002754	2.094910231
**NTNG1**	5.248816628	4.263854495	3.47E-11	5.248816628	2.505509091	2.39E-207	ECM_GLYCOPROTEINS	1.231002754	2.094910231
**OGFOD1**	10.5195548	10.46721055	0.033215536	10.5195548	2.750610227	1.54E-90	ECM_REGULATORS	1.005000783	3.824444008
**OVGP1**	7.427050117	6.236974432	6.63E-52	7.427050117	6.919146591	5.56E-304	ECM_AFFILIATED	1.190809775	1.073405516
**PLAT**	11.42578103	10.77696345	2.18E-10	11.42578103	10.77925227	2.07E-80	ECM_REGULATORS	1.060204118	1.059978999
**PLXDC2**	11.69932436	10.88319747	4.40E-25	11.69932436	0.061589773	2.52E-272	ECM_AFFILIATED	1.074989624	189.9556345
**PLXNA1**	11.93970703	11.50542314	7.38E-12	11.93970703	5.021021591	5.84E-11	ECM_AFFILIATED	1.037746016	2.377943773
**PLXNA3**	11.90166721	10.68557464	2.67E-113	11.90166721	8.4293625	3.41E-21	ECM_AFFILIATED	1.113806941	1.411929694
**PLXNB2**	14.00893934	13.66575592	2.00E-15	14.00893934	6.501055682	1.60E-84	ECM_AFFILIATED	1.025112656	2.154871459
**PODNL1**	8.589643091	6.57862844	2.37E-77	8.589643091	4.977126136	3.87E-148	PROTEOGLYCANS	1.305689046	1.72582387
**POMZP3**	9.178620609	8.756252394	5.56E-20	9.178620609	1.451835227	7.35E-216	ECM_GLYCOPROTEINS	1.048236185	6.322081485
**PXDN**	12.30886628	11.23052421	2.04E-33	12.30886628	0.502647727	0	ECM_GLYCOPROTEINS	1.096018854	24.48805716
**PXDNL**	5.193834426	4.528682305	3.65E-11	5.193834426	2.552178409	4.11E-139	ECM_GLYCOPROTEINS	1.146875421	2.035059308
**RSPO1**	8.564930445	3.104684801	0	8.564930445	1.812540909	7.62E-103	ECM_GLYCOPROTEINS	2.758711751	4.725372212
**RSPO3**	7.648325761	5.651119978	5.89E-59	7.648325761	7.129190909	1.40E-33	ECM_GLYCOPROTEINS	1.353417693	1.0728182
**RSPO4**	7.476433724	4.043109953	1.12E-146	7.476433724	1.403763636	1.05E-105	ECM_GLYCOPROTEINS	1.849178927	5.325991876
**SBSPON**	9.176945199	7.029450954	9.91E-87	9.176945199	8.265506818	2.90E-24	ECM_GLYCOPROTEINS	1.30549957	1.110270114
**SDC3**	12.44861593	11.85881039	9.51E-15	12.44861593	10.3619375	2.73E-118	ECM_AFFILIATED	1.049735641	1.201379175
**SEMA3B**	11.47289344	9.45391739	5.03E-93	11.47289344	4.055819318	1.65E-107	ECM_AFFILIATED	1.213559731	2.828748655
**SEMA3C**	10.50545831	10.05694137	0.000473095	10.50545831	0.316580682	7.78E-87	ECM_AFFILIATED	1.044597749	33.18414204

**SEMA3E**	6.613915457	5.110393121	1.06E-20	6.613915457	1.547186364	7.54E-66	ECM_AFFILIATED	1.294208743	4.274802061
**SEMA3F**	11.979774	10.55742698	2.94E-56	11.979774	5.262152273	1.11E-165	ECM_AFFILIATED	1.13472478	2.276592045
**SEMA4A**	10.02389391	9.707560846	9.19E-05	10.02389391	5.029613636	9.62E-24	ECM_AFFILIATED	1.032586256	1.992974935
**SEMA4C**	11.45498876	10.742427	2.46E-55	11.45498876	7.172706818	0.001062539	ECM_AFFILIATED	1.066331543	1.597024533
**SEMA4D**	10.58986745	10.42550944	0.003379259	10.58986745	1.698551136	3.40E-145	ECM_AFFILIATED	1.015764986	6.234647412
**SEMA4F**	8.842200468	8.659497007	0.00032161	8.842200468	8.810028409	3.22E-238	ECM_AFFILIATED	1.021098623	1.003651754
**SEMA5B**	8.764908431	6.812674584	4.39E-62	8.764908431	1.245409091	1.27E-66	ECM_AFFILIATED	1.286559093	7.037774571
**SEMA6D**	9.093357377	7.733087804	9.48E-39	9.093357377	2.064331818	8.61E-99	ECM_AFFILIATED	1.175902512	4.404988237
**SERPINA5**	9.75081897	6.262222677	1.79E-75	9.75081897	0.026001136	3.99E-05	ECM_REGULATORS	1.557085954	375.0151083
**SERPINB2**	6.052825761	3.895233046	6.79E-32	6.052825761	3.463939773	2.80E-66	ECM_REGULATORS	1.553905938	1.747381929
**SERPINB9**	9.808486417	9.255728713	3.59E-17	9.808486417	6.230551136	9.11E-19	ECM_REGULATORS	1.059720603	1.574256627
**SERPING1**	14.44445925	12.83354895	1.85E-73	14.44445925	10.41882273	1.21E-233	ECM_REGULATORS	1.125523369	1.386381132
**SERPINH1**	13.75354707	12.63424212	9.96E-61	13.75354707	5.440107955	7.11E-172	ECM_REGULATORS	1.088592964	2.528175394
**SLIT2**	9.078046838	8.231706209	4.75E-15	9.078046838	3.686559091	3.86E-74	ECM_GLYCOPROTEINS	1.102814727	2.462471539
**SLIT3**	10.3958918	9.712318679	2.49E-09	10.3958918	1.751048864	2.25E-13	ECM_GLYCOPROTEINS	1.070382073	5.936951286
**SLPI**	14.4749377	9.350146459	4.35E-156	14.4749377	6.950823864	2.02E-09	ECM_REGULATORS	1.54809743	2.08247799
**SMOC2**	9.715422951	9.331990381	0.000694413	9.715422951	7.158918182	1.34E-184	ECM_GLYCOPROTEINS	1.041087973	1.357107695
**SNED1**	9.375377752	9.168028551	0.007314321	9.375377752	2.744521591	1.24E-277	ECM_GLYCOPROTEINS	1.022616553	3.41603352
**SPOCK2**	12.80686909	10.58218176	4.45E-99	12.80686909	0.104305682	0.001727572	PROTEOGLYCANS	1.210229552	122.782085
**SPON1**	14.56326745	9.212814702	0	14.56326745	5.324669318	3.90E-102	ECM_GLYCOPROTEINS	1.580762006	2.735055752
**SRPX2**	7.945938407	7.697606859	0.028750269	7.945938407	0.447057955	5.88E-22	ECM_GLYCOPROTEINS	1.032260877	17.77384414
**SSPO**	7.035513583	5.474774412	1.74E-53	7.035513583	2.583748864	3.24E-110	ECM_GLYCOPROTEINS	1.285078262	2.722986619
**ST14**	13.31902553	11.62216987	1.56E-40	13.31902553	7.75235	6.41E-135	ECM_REGULATORS	1.146001623	1.718062978
**SULF1**	11.54973583	11.01650899	1.22E-05	11.54973583	7.992345455	2.75E-72	ECM_REGULATORS	1.048402524	1.445099677
**THBS3**	11.11961569	9.793048874	1.95E-157	11.11961569	5.484704545	5.47E-56	ECM_GLYCOPROTEINS	1.135460043	2.027386452
**THSD4**	12.47778759	9.59101531	2.35E-148	12.47778759	0.623610227	0.000289088	ECM_GLYCOPROTEINS	1.300987141	20.00895278
**TIMP1**	14.12874778	13.01716283	5.34E-38	14.12874778	10.69748068	2.90E-45	ECM_REGULATORS	1.085393795	1.320754689
**TSPEAR**	3.154500937	2.618330875	3.50E-07	3.154500937	0.246639773	2.09E-158	ECM_GLYCOPROTEINS	1.204775518	12.78991179
**WFIKKN1**	5.423455269	5.017183685	4.63E-07	5.423455269	0.677475	0.00598092	SECRETED_FACTORS	1.080976024	8.005395431
**WFIKKN2**	3.403708899	2.330780469	6.92E-27	3.403708899	1.607265909	1.46E-237	SECRETED_FACTORS	1.46033011	2.117701172
**WISP3**	7.103237237	3.981010653	2.71E-202	7.103237237	2.821670455	2.42E-18	ECM_GLYCOPROTEINS	1.784279886	2.517387254
**WNT10A**	10.31926628	5.772225994	1.44E-256	10.31926628	6.650735227	1.19E-96	SECRETED_FACTORS	1.787744674	1.551597819
**WNT11**	8.695292506	5.859420272	4.20E-129	8.695292506	2.8379625	6.74E-40	SECRETED_FACTORS	1.483985122	3.063920861
**WNT2**	6.356818501	5.529834111	2.21E-08	6.356818501	4.862244318	1.95E-05	SECRETED_FACTORS	1.149549584	1.307383604
**WNT6**	7.818470492	3.640180793	0	7.818470492	3.036595455	1.35E-186	SECRETED_FACTORS	2.147824775	2.574748796
**WNT9A**	7.697771194	6.88970344	4.97E-14	7.697771194	1.376176136	6.00E-124	SECRETED_FACTORS	1.11728629	5.593594447
**VWA1**	12.79596159	11.46398755	3.10E-81	12.79596159	9.995514773	4.20E-177	ECM_GLYCOPROTEINS	1.116187673	1.280170345
**VWA2**	6.978196721	5.53905211	2.49E-21	6.978196721	3.028419318	8.01E-88	ECM_GLYCOPROTEINS	1.25981785	2.30423729
**VWA3A**	5.838096956	2.750141213	2.22E-175	5.838096956	1.233936364	3.11E-33	ECM_GLYCOPROTEINS	2.122835339	4.731278798
**VWA7**	7.94507822	6.533085856	1.54E-56	7.94507822	2.859077273	1.55E-18	ECM_GLYCOPROTEINS	1.216129467	2.778895938
**VWA9**	10.7910993	10.61640365	1.01E-14	10.7910993	0	3.42E-08	ECM_GLYCOPROTEINS	1.016455256	Inf
**VWCE**	8.201612881	5.711136161	1.02E-124	8.201612881	7.819661364	1.29E-58	ECM_GLYCOPROTEINS	1.436073778	1.04884502
**VWDE**	6.007568852	4.160367421	5.18E-40	6.007568852	0.554190909	2.82E-65	ECM_GLYCOPROTEINS	1.443999591	10.84025153
**XCL2**	4.22536089	3.451260562	3.25E-17	4.22536089	0.949342045	2.95E-07	SECRETED_FACTORS	1.224294954	4.450830878
**ZP3**	9.301655738	7.719755915	4.79E-105	9.301655738	6.596473864	1.69E-11	ECM_GLYCOPROTEINS	1.204915782	1.41009514

Pancreatic Adenocarcinoma (PAAD)

Signature genes	Expression in tumor	Expression in rest of Pan-Cancer	P-value	Expression in tumor	Expression in normal tissue	P-value	Matrisome category	Fold change vs. Pan-Cancer	Fold change vs. normal
**ADAM15**	12.47848361	12.16121957	1.22E-05	12.47848361	11.6925	0.021874399	ECM_REGULATORS	1.026088176	1.067221177
**ANXA10**	10.25956066	2.19835954	3.56E-229	10.25956066	6.01345	0.005978883	ECM_AFFILIATED	4.666916612	1.706102263
**ANXA13**	7.964411475	3.905186782	8.71E-60	7.964411475	4.871275	0.00754829	ECM_AFFILIATED	2.039444442	1.634974719
**AREG**	9.449830055	6.979811355	1.37E-24	9.449830055	6.7669	0.002946447	SECRETED_FACTORS	1.353880438	1.396478455
**BTC**	7.506886885	6.44669637	1.33E-09	7.506886885	6.16245	0.004663966	SECRETED_FACTORS	1.164454855	1.218165971
**CCL15**	5.696214754	2.678599631	7.29E-33	5.696214754	3.000075	0.020529667	SECRETED_FACTORS	2.126564451	1.898690784
**COL17A1**	11.72529727	7.803316101	1.73E-36	11.72529727	7.194725	0.00271202	COLLAGENS	1.502604421	1.629707497
**CST4**	5.105444809	2.105719187	3.75E-57	5.105444809	2.9555	0.046154684	ECM_REGULATORS	2.424561091	1.727438609
**CTSE**	13.51801257	4.862236995	1.02E-152	13.51801257	10.150975	0.035408968	ECM_REGULATORS	2.780204375	1.331695977
**DMBT1**	11.3447694	5.089390596	6.77E-87	11.3447694	7.027675	0.010792403	ECM_GLYCOPROTEINS	2.229101733	1.614299096
**EREG**	6.938223497	4.273947118	4.80E-25	6.938223497	3.007625	0.00387164	SECRETED_FACTORS	1.623376075	2.306877851
**F12**	7.891927322	7.222801504	0.000281678	7.891927322	5.991525	0.005490922	ECM_REGULATORS	1.09264076	1.31718174
**LAMA3**	12.65140328	9.789260025	2.76E-47	12.65140328	10.421375	0.004360101	BASEMENT_MEMBRANES	1.292375853	1.213985993
**LAMA3**	12.65140328	9.789260025	2.76E-47	12.65140328	10.421375	0.004360101	ECM_GLYCOPROTEINS	1.292375853	1.213985993
**MMP28**	10.03943825	7.18999178	1.55E-66	10.03943825	8.128275	0.007583901	ECM_REGULATORS	1.396307334	1.235125319
**MMP3**	6.17977377	4.550021031	1.64E-08	6.17977377	3.7135	0.021260895	ECM_REGULATORS	1.35818576	1.664137275
**MUC1**	14.19635902	10.81774834	2.95E-43	14.19635902	11.121575	0.002960313	ECM_AFFILIATED	1.312321064	1.276470196
**MUC13**	11.98540492	4.689187539	4.03E-106	11.98540492	8.1559	0.000651207	ECM_AFFILIATED	2.555966214	1.469537993
**MUC17**	8.533389071	1.449220468	3.23E-169	8.533389071	4.063075	0.031406015	ECM_AFFILIATED	5.88826149	2.100229277
**MUC4**	10.1772306	6.66719145	1.38E-34	10.1772306	6.927825	0.019157022	ECM_AFFILIATED	1.526464431	1.469036906
**MUC5AC**	11.6032153	3.069593129	2.55E-173	11.6032153	5.1649	0.002076761	ECM_AFFILIATED	3.780049933	2.246551782
**MUC5B**	12.25629235	5.980757735	5.79E-77	12.25629235	8.151925	0.007962225	ECM_AFFILIATED	2.049287547	1.503484435
**NDNF**	7.559861202	5.602530134	5.48E-18	7.559861202	5.53135	0.032435725	ECM_GLYCOPROTEINS	1.349365558	1.366729858
**P4HA2**	11.43687541	10.69032027	2.73E-12	11.43687541	10.6788	0.021159716	ECM_REGULATORS	1.069834684	1.07098882
**PI3**	8.870198907	5.892774864	1.59E-18	8.870198907	6.222275	0.042465057	ECM_REGULATORS	1.505266892	1.42555559
**PLXNA2**	11.42138689	10.51896031	9.05E-21	11.42138689	9.95755	0.000128787	ECM_AFFILIATED	1.085790473	1.147007736
**PRSS12**	9.131220765	6.801189645	1.18E-33	9.131220765	7.724125	0.036134263	ECM_REGULATORS	1.3425917	1.182168953
**PRSS3**	10.67896612	4.432121497	1.01E-111	10.67896612	7.252875	0.008467159	ECM_REGULATORS	2.409447965	1.472376971
**REG4**	10.5203	2.644548525	1.16E-176	10.5203	5.901775	0.026885294	ECM_AFFILIATED	3.978108135	1.782565415
**SDC1**	13.45909508	12.44859451	1.78E-07	13.45909508	11.597575	0.005448054	ECM_AFFILIATED	1.081173869	1.160509424
**SEMA4G**	10.28006339	8.468645953	1.94E-36	10.28006339	8.635625	0.007155921	ECM_AFFILIATED	1.213896938	1.190424942
**SERPINB3**	6.098840437	3.342312908	4.81E-16	6.098840437	2.614575	0.048445434	ECM_REGULATORS	1.824736524	2.332631666
**SERPINB4**	4.591914208	2.714413956	7.76E-11	4.591914208	1.7921	0.038341617	ECM_REGULATORS	1.69167794	2.562309139
**SERPINB5**	10.00606831	5.996560472	2.04E-27	10.00606831	4.419725	7.75E-05	ECM_REGULATORS	1.668634604	2.263957216
**SFTA2**	8.554688525	3.041460899	6.77E-96	8.554688525	5.757675	0.030760845	ECM_AFFILIATED	2.812690615	1.485788712
**SLPI**	12.15946612	9.593511694	4.43E-18	12.15946612	9.547225	0.004292805	ECM_REGULATORS	1.267467691	1.273612607
**TINAG**	4.450571585	1.897891246	7.82E-25	4.450571585	1.072075	0.007613601	ECM_GLYCOPROTEINS	2.345008753	4.151362157
**WNT7B**	9.150920219	7.192302019	6.03E-13	9.150920219	6.636525	0.006511436	SECRETED_FACTORS	1.272321462	1.378872259
**VWA3B**	4.756512022	3.223021254	2.51E-19	4.756512022	2.997125	0.014354663	ECM_GLYCOPROTEINS	1.475792943	1.587024906

Pheochromocytoma and paraganglioma (PCPG)

Signature genes	Expression in tumor	Expression in rest of Pan-Cancer	P-value	Expression in tumor	Expression in normal tissue	P-value	Matrisome category	Fold change vs. Pan-Cancer	Fold change vs. normal
**ADAM23**	10.15726919	6.750581499	3.40E-65	10.15726919	7.9768	0.001439183	ECM_REGULATORS	1.504650998	1.273351368
**ADAM29**	2.209852432	0.994209406	6.25E-30	2.209852432	0	0.026336233	ECM_REGULATORS	2.222723321	Inf
**ADAMTS13**	7.369545405	7.01204008	0.000323348	7.369545405	5.7199	0.001653674	ECM_REGULATORS	1.050984495	1.288404588
**ADAMTS19**	8.063711351	2.179762464	1.17E-207	8.063711351	3.587333333	0.001795953	ECM_REGULATORS	3.699353249	2.247828847
**ADAMTS9**	9.734160541	8.997890245	3.98E-08	9.734160541	8.578733333	0.044308842	ECM_REGULATORS	1.081826992	1.134685059
**AMH**	6.411084865	4.821796758	7.88E-19	6.411084865	2.881466667	0.00096161	SECRETED_FACTORS	1.329604956	2.224938063
**ANGPT2**	9.136489189	8.567633062	1.25E-06	9.136489189	4.7242	2.22E-05	SECRETED_FACTORS	1.066395949	1.933975951
**ANXA6**	13.59219568	12.23052098	1.84E-50	13.59219568	12.90676667	0.007946362	ECM_AFFILIATED	1.111334153	1.053106175
**BCAN**	9.391909189	4.482181285	1.91E-56	9.391909189	6.0429	0.016060788	PROTEOGLYCANS	2.095388069	1.554205628
**BMP7**	12.03806703	7.957072209	2.50E-49	12.03806703	6.358666667	2.90E-05	SECRETED_FACTORS	1.512876434	1.893174726
**BMP8B**	9.906523784	7.758192273	3.05E-65	9.906523784	5.561533333	8.85E-06	SECRETED_FACTORS	1.276911352	1.781257648
**BMPER**	6.454091351	5.058993768	1.86E-16	6.454091351	4.6716	0.043513461	ECM_GLYCOPROTEINS	1.275765824	1.38155907
**C1QL1**	11.22214757	5.801038827	5.38E-130	11.22214757	6.225066667	0.001613579	ECM_AFFILIATED	1.934506543	1.802735323
**C1QL4**	4.840688108	3.566869113	7.43E-10	4.840688108	1.721166667	0.012588041	ECM_AFFILIATED	1.357125242	2.812445884
**C1QTNF9**	2.57108973	2.270456455	0.013058798	2.57108973	0.7714	0.024787353	ECM_AFFILIATED	1.132410941	3.333017539
**CBLN1**	8.552329189	3.918867463	6.05E-121	8.552329189	4.221	0.002228238	SECRETED_FACTORS	2.182347137	2.026138164
**CLEC4F**	3.626871892	3.280324267	0.012425641	3.626871892	0.716166667	0.008184551	ECM_AFFILIATED	1.105644319	5.064284699
**COL11A2**	6.850741081	5.97384306	4.67E-08	6.850741081	4.613066667	0.008344912	COLLAGENS	1.146789598	1.485073071
**COL21A1**	6.988367027	6.140055465	2.63E-07	6.988367027	5.0952	0.043785709	COLLAGENS	1.138160244	1.371558924
**COL22A1**	11.1239173	6.463970763	2.41E-117	11.1239173	5.7366	2.67E-05	COLLAGENS	1.720910831	1.93911329
**COL4A1**	13.74791946	13.18093568	5.59E-05	13.74791946	12.03913333	0.025130509	COLLAGENS	1.043015442	1.141935975
**COL4A1**	13.74791946	13.18093568	5.59E-05	13.74791946	12.03913333	0.025130509	BASEMENT_MEMBRANES	1.043015442	1.141935975
**COL9A1**	4.643000541	3.953880654	0.001198299	4.643000541	2.156766667	0.024410971	COLLAGENS	1.174289501	2.152759783
**COLQ**	7.247843243	6.257493273	8.00E-26	7.247843243	5.992666667	0.043031221	BASEMENT_MEMBRANES	1.158266246	1.209452093
**COLQ**	7.247843243	6.257493273	8.00E-26	7.247843243	5.992666667	0.043031221	ECM_GLYCOPROTEINS	1.158266246	1.209452093
**CRELD1**	11.62089405	10.22388406	2.03E-102	11.62089405	10.69396667	0.011900322	ECM_GLYCOPROTEINS	1.136641807	1.086677602
**CSPG5**	7.322000541	6.030864978	9.68E-12	7.322000541	3.8906	5.94E-07	ECM_AFFILIATED	1.214087957	1.881972071
**EGFLAM**	11.88806162	7.551577655	4.60E-254	11.88806162	8.518166667	0.001140655	ECM_GLYCOPROTEINS	1.574248742	1.395612705
**ELN**	11.19355027	10.03016328	5.01E-12	11.19355027	9.377833333	0.034166501	ECM_GLYCOPROTEINS	1.115988839	1.193617958
**ESM1**	9.778595676	7.66373014	9.23E-30	9.778595676	7.1215	0.031357189	PROTEOGLYCANS	1.275957725	1.37310899
**FGF1**	9.605981622	7.082217501	1.08E-40	9.605981622	5.486666667	0.000279715	SECRETED_FACTORS	1.356352247	1.750786444
**FGF14**	10.42451081	5.495114065	1.92E-164	10.42451081	5.5986	5.85E-09	SECRETED_FACTORS	1.89705085	1.861985284
**FGF2**	9.134366486	7.79258444	2.05E-18	9.134366486	7.6043	0.015685606	SECRETED_FACTORS	1.17218704	1.201210695
**FRAS1**	8.939234595	8.390863269	0.002989113	8.939234595	6.105133333	0.003690267	ECM_GLYCOPROTEINS	1.065353386	1.464216112
**GAS6**	12.86997784	11.71689571	3.26E-27	12.86997784	11.86096667	0.004534005	ECM_GLYCOPROTEINS	1.098411914	1.085069894
**HMCN2**	7.629457297	6.346645117	1.35E-13	7.629457297	4.795066667	0.008732534	ECM_GLYCOPROTEINS	1.202124454	1.591105573
**HTRA1**	12.72659568	11.80214542	1.87E-11	12.72659568	11.42356667	0.031086826	ECM_REGULATORS	1.078329001	1.114064989
**IGFBPL1**	7.894916757	3.089620899	3.90E-169	7.894916757	2.154766667	2.75E-10	ECM_GLYCOPROTEINS	2.555302743	3.663931171
**LOXL3**	10.43425297	7.800972054	2.24E-132	10.43425297	8.027633333	0.001143281	ECM_REGULATORS	1.337558051	1.299791924
**MMP15**	11.6785427	10.77564726	1.13E-15	11.6785427	9.0148	4.34E-09	ECM_REGULATORS	1.083790367	1.295485502
**MMP16**	8.547111892	6.812869103	2.31E-19	8.547111892	5.834133333	0.001287375	ECM_REGULATORS	1.254553957	1.465018265
**MMP17**	7.673587027	6.575559765	1.12E-10	7.673587027	4.764433333	0.007934962	ECM_REGULATORS	1.166986128	1.610598048

**MMP24**	9.015028649	6.919515463	1.66E-31	9.015028649	5.904566667	2.68E-06	ECM_REGULATORS	1.302841029	1.526789205
**MUC12**	5.636061081	4.994126509	0.004612188	5.636061081	1.6878	0.010214098	ECM_AFFILIATED	1.128537908	3.339294396
**NELL1**	5.143024865	3.295989652	1.78E-14	5.143024865	1.549133333	0.047639032	ECM_GLYCOPROTEINS	1.560388656	3.319936867
**NELL2**	9.887304865	7.804519588	2.59E-25	9.887304865	6.0374	0.011320177	ECM_GLYCOPROTEINS	1.26686912	1.637675964
**NTN3**	2.813151351	2.098356416	5.05E-09	2.813151351	0.677933333	0.010389194	ECM_GLYCOPROTEINS	1.34064515	4.149598807
**NTN3**	2.813151351	2.098356416	5.05E-09	2.813151351	0.677933333	0.010389194	BASEMENT_MEMBRANES	1.34064515	4.149598807
**NTNG1**	8.858544865	4.220461551	3.66E-98	8.858544865	3.365066667	0.000235649	BASEMENT_MEMBRANES	2.09895168	2.63250204
**NTNG1**	8.858544865	4.220461551	3.66E-98	8.858544865	3.365066667	0.000235649	ECM_GLYCOPROTEINS	2.09895168	2.63250204
**P4HA2**	11.47875568	10.68942327	1.07E-13	11.47875568	8.288166667	8.03E-09	ECM_REGULATORS	1.073842376	1.384957149
**P4HTM**	12.76905459	10.26358265	2.13E-154	12.76905459	11.442	0.000262902	ECM_REGULATORS	1.244112804	1.115980999
**PARM1**	14.17438595	10.00073266	4.20E-138	14.17438595	11.3275	1.86E-05	ECM_AFFILIATED	1.417334752	1.251325177
**PLOD3**	11.96138757	11.68413105	0.000385585	11.96138757	10.75793333	0.000129464	ECM_REGULATORS	1.023729323	1.111866675
**PLXDC1**	9.237015676	8.821530489	7.93E-05	9.237015676	7.1762	0.005854972	ECM_AFFILIATED	1.047098991	1.287173668
**PLXNA2**	11.29858811	10.52099029	5.84E-16	11.29858811	7.984566667	3.97E-07	ECM_AFFILIATED	1.073909185	1.415053387
**PLXNA3**	12.38694108	10.70751952	6.05E-97	12.38694108	9.941233333	2.37E-09	ECM_AFFILIATED	1.156845062	1.246016532
**PLXNA4**	9.218675676	6.903955669	8.57E-35	9.218675676	6.601233333	0.032718968	ECM_AFFILIATED	1.33527446	1.396508078
**PLXNC1**	11.77124324	9.625073238	5.95E-72	11.77124324	8.9879	0.000252737	ECM_AFFILIATED	1.222977005	1.309676703
**POMZP3**	8.984627568	8.770460571	0.00201205	8.984627568	8.0697	0.017954048	ECM_GLYCOPROTEINS	1.024419128	1.113378139
**PRSS3**	10.25086703	4.438596418	1.05E-97	10.25086703	4.5678	2.23E-05	ECM_REGULATORS	2.309483914	2.244158463
**PXDNL**	4.879395135	4.525718948	0.019049931	4.879395135	1.191	0.000143555	ECM_GLYCOPROTEINS	1.078148067	4.096889282
**RSPO4**	7.830937838	4.132178548	7.23E-75	7.830937838	3.5728	0.011418238	ECM_GLYCOPROTEINS	1.895111198	2.191820935
**SDC2**	12.40801514	11.08943676	8.31E-19	12.40801514	9.746033333	1.73E-05	ECM_AFFILIATED	1.118903999	1.273134896
**SEMA3G**	9.77027027	8.245383178	3.24E-36	9.77027027	7.9428	0.012954564	ECM_AFFILIATED	1.184938293	1.230078848
**SEMA4F**	9.897177838	8.64873478	1.56E-61	9.897177838	7.4915	7.67E-06	ECM_AFFILIATED	1.144349791	1.321120982
**SEMA5B**	7.747103243	6.875239711	8.04E-07	7.747103243	6.275633333	0.039707245	ECM_AFFILIATED	1.126812092	1.234473531
**SEMA6C**	10.22548216	8.340432867	1.32E-86	10.22548216	7.028133333	1.89E-09	ECM_AFFILIATED	1.226013365	1.45493571
**SEMA6D**	9.26345027	7.734931101	3.03E-22	9.26345027	6.0081	1.60E-06	ECM_AFFILIATED	1.197612513	1.541826912
**SERPINE2**	11.39018649	10.72928134	0.000131694	11.39018649	8.789033333	0.013229428	ECM_REGULATORS	1.061598268	1.295954408
**SERPINI1**	10.84008162	7.954987905	1.71E-125	10.84008162	7.8606	2.79E-06	ECM_REGULATORS	1.362677323	1.379039974
**SFTPC**	4.442125405	2.577904349	4.26E-10	4.442125405	2.310866667	0.039079345	ECM_AFFILIATED	1.723153696	1.92227681
**SFTPD**	6.90176	5.259608979	8.59E-14	6.90176	2.418933333	3.92E-11	ECM_AFFILIATED	1.312219222	2.853224562
**SLIT3**	13.37917622	9.668866259	2.86E-106	13.37917622	11.43516667	0.007124135	ECM_GLYCOPROTEINS	1.383737851	1.17000273
**SPOCK2**	14.14896054	10.60871472	1.01E-112	14.14896054	10.583	0.000102788	PROTEOGLYCANS	1.333711097	1.336951766
**SPOCK3**	11.90596919	3.707118016	1.65E-216	11.90596919	6.974433333	0.000318986	PROTEOGLYCANS	3.211650975	1.707087676
**TECTA**	7.924528108	5.122050447	6.88E-155	7.924528108	6.03	8.13E-06	ECM_GLYCOPROTEINS	1.547139801	1.314183766
**THBS3**	10.26385081	9.852804514	6.32E-08	10.26385081	8.5171	4.49E-06	ECM_GLYCOPROTEINS	1.04171871	1.205087508
**TLL2**	6.556991892	4.882329344	5.76E-36	6.556991892	1.8899	6.51E-05	ECM_REGULATORS	1.34300483	3.46949145
**TNR**	10.0256827	3.439133799	7.72E-148	10.0256827	5.1753	0.000275786	ECM_GLYCOPROTEINS	2.915176695	1.937217688
**WFIKKN1**	5.993617838	5.01520065	4.90E-16	5.993617838	3.023966667	1.31E-05	SECRETED_FACTORS	1.195090337	1.982038329
**WNT10A**	6.717854054	5.955984663	0.000201986	6.717854054	0.9246	0.000316304	SECRETED_FACTORS	1.127916614	7.265686842
**WNT6**	6.908831351	3.787265065	3.09E-87	6.908831351	3.946366667	0.008433014	SECRETED_FACTORS	1.824227043	1.75068156
**WNT8B**	3.780857297	1.750456843	6.69E-70	3.780857297	1.090333333	0.022372075	SECRETED_FACTORS	2.159926028	3.467615987
**VWA3A**	3.748077838	2.928454397	1.95E-06	3.748077838	1.369266667	0.027707901	ECM_GLYCOPROTEINS	1.279882603	2.737288455
**VWA5B1**	5.173526486	1.851240827	2.30E-91	5.173526486	0.762466667	0.000686614	ECM_GLYCOPROTEINS	2.794626399	6.785249392
**VWA7**	8.472305946	6.558491642	2.39E-46	8.472305946	3.6214	1.14E-06	ECM_GLYCOPROTEINS	1.291807081	2.339511224
**VWDE**	6.871858919	4.220677286	1.19E-36	6.871858919	2.9824	0.007764324	ECM_GLYCOPROTEINS	1.62814128	2.304137245

**VWF**	13.35014973	12.18628817	1.73E-24	13.35014973	10.47066667	0.00078012	ECM_GLYCOPROTEINS	1.09550583	1.275004749

Prostate Adenocarcinoma (PRAD)

Signature genes	Expression in tumor	Expression in rest of Pan-Cancer	P-value	Expression in tumor	Expression in normal tissue	P-value	Matrisome category	Fold change vs. Pan-Cancer	Fold change vs. normal
**ADAM15**	12.56629069	12.14472699	4.29E-23	12.56629069	12.10831538	5.83E-10	ECM_REGULATORS	1.034711665	1.037823206
**ADAM2**	4.134672993	0.362217648	0	4.134672993	1.482911538	4.51E-12	ECM_REGULATORS	11.41488555	2.78821284
**ADAM21**	3.768488504	2.877626653	2.41E-43	3.768488504	2.338698077	1.19E-09	ECM_REGULATORS	1.309582152	1.611361698
**ADAM7**	4.150734854	0.313866194	0	4.150734854	3.165875	0.020582095	ECM_REGULATORS	13.22453624	1.311086146
**ADAMTS13**	7.540296533	6.989568397	6.54E-21	7.540296533	7.038532692	3.25E-05	ECM_REGULATORS	1.078792867	1.071288131
**AMELY**	1.977266058	0.168306882	0	1.977266058	1.52765	0.010309323	ECM_GLYCOPROTEINS	11.74798104	1.294318763
**ANGPTL3**	3.583137409	2.322680441	1.76E-19	3.583137409	1.687676923	8.37E-09	SECRETED_FACTORS	1.542673433	2.123118092
**ANXA3**	11.4790792	9.036710645	7.77E-98	11.4790792	11.05941346	0.002989247	ECM_AFFILIATED	1.270271855	1.037946473
**ANXA5**	13.67889854	13.36582211	4.81E-12	13.67889854	13.50140192	0.006753936	ECM_AFFILIATED	1.023423656	1.013146532
**BGLAP**	4.259316423	4.091953305	0.008138452	4.259316423	3.490090385	6.72E-05	ECM_GLYCOPROTEINS	1.040900544	1.220402899
**BMP6**	7.630523905	6.598832126	7.14E-42	7.630523905	6.952392308	0.000753369	SECRETED_FACTORS	1.156344602	1.097539317
**BMP8A**	4.591429015	4.232100533	1.52E-05	4.591429015	3.990276923	0.000348302	SECRETED_FACTORS	1.084905469	1.150654229
**C17orf58**	9.141172263	8.740792132	1.65E-24	9.141172263	8.603959615	1.04E-07	ECM_REGULATORS	1.045805932	1.062437839
**C1QTNF9**	3.549381752	2.205537056	8.72E-80	3.549381752	3.056867308	0.006489066	ECM_AFFILIATED	1.609304973	1.161117378
**C1QTNF9B**	4.549483394	2.16900492	1.07E-245	4.549483394	3.625119231	0.000765292	SECRETED_FACTORS	2.09749796	1.254988624
**CBLN3**	7.151815876	6.728865872	6.54E-15	7.151815876	6.919782692	0.014634176	SECRETED_FACTORS	1.062856061	1.03353186
**CHRD**	8.505031204	7.603469806	6.79E-25	8.505031204	7.650155769	2.87E-07	SECRETED_FACTORS	1.118572365	1.111746148
**CLEC19A**	0.479589051	0.233499255	5.87E-21	0.479589051	0.218098077	0.004490824	ECM_AFFILIATED	2.053921115	2.198960476
**COL12A1**	11.61680091	11.28133064	0.002908034	11.61680091	11.17878462	0.032141244	COLLAGENS	1.029736765	1.039182819
**COL26A1**	8.418630109	4.616069192	1.53E-214	8.418630109	7.593005769	6.46E-05	COLLAGENS	1.823766014	1.10873485
**COL28A1**	7.639440876	5.460073659	4.27E-100	7.639440876	6.907826923	0.000477008	COLLAGENS	1.39914612	1.105910869
**COL2A1**	7.128061861	4.01038687	3.66E-99	7.128061861	5.250392308	0.000418909	COLLAGENS	1.77740006	1.35762462
**COL8A1**	10.06752664	9.77603274	0.012559713	10.06752664	9.683448077	0.032960878	COLLAGENS	1.029817198	1.039663409
**COL9A2**	11.81276861	8.581879525	2.55E-275	11.81276861	10.63039808	1.16E-05	COLLAGENS	1.376478029	1.111225424
**COLEC12**	11.44653285	8.34171468	1.08E-218	11.44653285	10.26007115	1.45E-13	ECM_AFFILIATED	1.372203832	1.115638739
**COMP**	9.557698358	7.104933444	1.04E-49	9.557698358	7.236878846	5.44E-12	ECM_GLYCOPROTEINS	1.34521997	1.320693432
**CRELD1**	11.41088631	10.18444042	2.54E-229	11.41088631	11.17338077	0.000253054	ECM_GLYCOPROTEINS	1.120423494	1.021256373
**CRELD2**	11.05154106	10.35796178	5.20E-82	11.05154106	10.53314423	8.37E-08	ECM_GLYCOPROTEINS	1.066960981	1.049215772
**CSPG5**	6.38787281	6.035213482	0.001676539	6.38787281	5.442238462	2.46E-09	ECM_AFFILIATED	1.058433613	1.173758345
**CST2**	6.21092354	3.461879706	4.79E-111	6.21092354	3.768309615	6.92E-11	ECM_REGULATORS	1.794089936	1.648198841
**CXCL11**	7.332879562	6.430271868	1.31E-14	7.332879562	5.970665385	3.42E-08	SECRETED_FACTORS	1.140368512	1.228151151
**EGF**	7.954211131	5.399798551	4.98E-94	7.954211131	7.319434615	0.004546676	SECRETED_FACTORS	1.473057014	1.086724802
**ELFN2**	1.661190876	1.092207828	7.22E-17	1.661190876	0.613257692	8.90E-09	ECM_AFFILIATED	1.520947601	2.708797455
**FAM20C**	10.87980985	10.66803229	0.000357648	10.87980985	10.54674038	0.000186006	ECM_REGULATORS	1.019851605	1.031580323
**FBLN7**	7.832843066	7.265773589	1.60E-13	7.832843066	7.60685	0.045782132	ECM_GLYCOPROTEINS	1.078046676	1.029709152
**FGF13**	10.25233339	7.112633575	7.04E-241	10.25233339	9.548055769	5.04E-09	SECRETED_FACTORS	1.441425779	1.073761365
**FGF17**	3.781531569	3.084250689	4.51E-13	3.781531569	3.041359615	0.000711838	SECRETED_FACTORS	1.226077887	1.243368772
**FGF22**	4.087429197	2.386718292	1.00E-158	4.087429197	3.379028846	1.40E-05	SECRETED_FACTORS	1.712572955	1.209646139
**FGL1**	4.632033394	2.752218875	3.45E-32	4.632033394	2.796446154	1.01E-07	ECM_GLYCOPROTEINS	1.683017814	1.656399995
**FMOD**	12.54958923	10.22524725	7.62E-114	12.54958923	12.08655	0.025292538	PROTEOGLYCANS	1.227314013	1.03831029
**FNDC8**	1.531914416	1.155008381	9.93E-18	1.531914416	1.012915385	3.90E-05	ECM_GLYCOPROTEINS	1.326323203	1.512381428
**GRIFIN**	0.356318978	0.180962139	3.34E-13	0.356318978	0.113598077	0.007913077	ECM_AFFILIATED	1.969025013	3.136663822

**HPX**	6.061289234	4.92717544	1.36E-16	6.061289234	4.853032692	6.20E-13	ECM_AFFILIATED	1.230175241	1.24896938
**HYAL3**	7.789852007	7.072909508	5.04E-59	7.789852007	7.439005769	0.000124884	ECM_REGULATORS	1.10136458	1.047163055
**IGFBP2**	13.1865135	11.47178885	6.96E-63	13.1865135	12.61118654	8.09E-06	ECM_GLYCOPROTEINS	1.149473171	1.045620368
**IMPG1**	2.899341058	2.702249432	0.003208785	2.899341058	2.289076923	0.001186588	PROTEOGLYCANS	1.072936134	1.266598352
**MMP10**	6.957133942	4.567359392	2.91E-51	6.957133942	5.136382692	6.33E-10	ECM_REGULATORS	1.523228926	1.354481229
**MMP26**	4.537673905	0.223018302	0	4.537673905	1.963844231	1.98E-17	ECM_REGULATORS	20.34664362	2.310607855
**MUC2**	5.850437044	2.835298702	2.88E-70	5.850437044	4.459536538	6.60E-05	ECM_AFFILIATED	2.063428816	1.31189351
**NGLY1**	10.34824635	9.952478398	8.01E-55	10.34824635	10.10415192	5.02E-06	ECM_REGULATORS	1.039765769	1.024157834
**NTN5**	4.677058577	3.6458902	1.12E-51	4.677058577	4.105321154	0.001417172	BASEMENT_MEMBRANES	1.282830343	1.139267405
**NTN5**	4.677058577	3.6458902	1.12E-51	4.677058577	4.105321154	0.001417172	ECM_GLYCOPROTEINS	1.282830343	1.139267405
**OGFOD2**	9.273159124	9.05689676	1.06E-13	9.273159124	8.968367308	2.85E-05	ECM_REGULATORS	1.023878197	1.033985207
**OVGP1**	7.824262956	6.235019901	2.80E-108	7.824262956	7.207419231	8.65E-05	ECM_AFFILIATED	1.254889813	1.085584549
**P4HA3**	7.429637044	6.320355277	2.09E-39	7.429637044	6.980713462	0.012567498	ECM_REGULATORS	1.175509401	1.064309126
**P4HTM**	11.41305967	10.24684042	3.83E-95	11.41305967	10.85587885	4.05E-15	ECM_REGULATORS	1.113812571	1.051325262
**PCSK6**	9.762374818	9.09198687	5.03E-11	9.762374818	8.078165385	1.48E-21	ECM_REGULATORS	1.073733933	1.2084891
**PLXNA3**	11.13156971	10.71539893	4.02E-18	11.13156971	10.58356923	1.81E-10	ECM_AFFILIATED	1.03883857	1.051778419
**PLXNB2**	14.09927755	13.66023837	1.24E-30	14.09927755	13.95608269	0.022355217	ECM_AFFILIATED	1.032139936	1.010260391
**PLXNB3**	10.58575438	8.221180139	6.01E-97	10.58575438	9.696528846	4.68E-14	ECM_AFFILIATED	1.287619806	1.091705552
**PXDN**	11.87006752	11.23308719	1.17E-15	11.87006752	11.20007308	2.62E-07	ECM_GLYCOPROTEINS	1.056705723	1.059820542
**REG4**	6.733936131	2.564085512	2.19E-142	6.733936131	5.875882692	0.005435371	ECM_AFFILIATED	2.626252557	1.146029709
**SEMA4A**	11.20312664	9.642025475	3.93E-108	11.20312664	10.9995	0.007196581	ECM_AFFILIATED	1.161905937	1.018512354
**SERPINA11**	5.804103832	2.231528997	2.81E-126	5.804103832	3.795336538	1.58E-07	ECM_REGULATORS	2.600953804	1.529272509
**SERPINB6**	13.22095328	11.80610427	2.39E-290	13.22095328	12.768975	2.97E-07	ECM_REGULATORS	1.119840464	1.035396599
**SFTPA2**	10.35024635	3.910794839	1.37E-232	10.35024635	8.824530769	1.04E-06	ECM_AFFILIATED	2.646583822	1.172894811
**SLIT1**	8.22922354	5.280612647	6.49E-116	8.22922354	5.680530769	2.56E-18	ECM_GLYCOPROTEINS	1.558384243	1.44867159
**SMOC2**	11.8738542	9.225192917	1.61E-159	11.8738542	11.49413654	0.002439051	ECM_GLYCOPROTEINS	1.28711175	1.033035771
**SPOCK1**	13.64525201	9.115664866	5.92e-323	13.64525201	12.75562885	7.36E-07	PROTEOGLYCANS	1.496901456	1.069743575
**SPON2**	14.29966898	10.10096099	0	14.29966898	12.26757115	2.79E-10	ECM_GLYCOPROTEINS	1.415674112	1.165647935
**SSPO**	7.194232847	5.460666385	6.48E-84	7.194232847	5.804007692	7.04E-12	ECM_GLYCOPROTEINS	1.317464269	1.239528482
**ST14**	13.29507026	11.63454864	3.03E-49	13.29507026	12.62860577	3.55E-14	ECM_REGULATORS	1.142723338	1.052774194
**TECTB**	1.510285401	0.862595251	7.07E-24	1.510285401	0.963417308	0.016485497	ECM_GLYCOPROTEINS	1.75086218	1.567633661
**TGM2**	13.40505931	12.0307264	2.41E-63	13.40505931	13.08545385	0.049005623	ECM_REGULATORS	1.114235239	1.024424484
**TGM3**	7.792613504	3.787472724	9.14E-186	7.792613504	5.371325	1.06E-16	ECM_REGULATORS	2.057470528	1.450780488
**TGM6**	0.391103467	0.268784918	0.003624613	0.391103467	0.150478846	0.017392387	ECM_REGULATORS	1.45507966	2.59905945
**THBS4**	10.49291095	7.79211316	1.75E-109	10.49291095	8.660148077	3.61E-12	ECM_GLYCOPROTEINS	1.346606592	1.211631817
**TLL2**	6.334877555	4.833412567	4.45E-81	6.334877555	5.997759615	0.029956531	ECM_REGULATORS	1.310642836	1.056207311
**WFIKKN1**	5.670917883	4.997258618	3.41E-21	5.670917883	4.839794231	1.36E-06	SECRETED_FACTORS	1.134805764	1.171727064
**WNT10B**	4.504440876	4.221638565	0.000795608	4.504440876	3.694890385	7.26E-07	SECRETED_FACTORS	1.066988755	1.219100002
**WNT7B**	9.522419708	7.099890572	3.58E-52	9.522419708	9.185626923	0.029049445	SECRETED_FACTORS	1.341206545	1.036665193
**WNT8B**	3.562463321	1.688342399	4.35E-171	3.562463321	3.150292308	0.01341573	SECRETED_FACTORS	2.110036047	1.1308358
**ZP1**	4.488258029	1.994551615	1.25E-239	4.488258029	3.036090385	1.34E-08	ECM_GLYCOPROTEINS	2.250259154	1.478301849
**ZP3**	8.536071898	7.755730315	3.12E-33	8.536071898	7.248788462	3.40E-14	ECM_GLYCOPROTEINS	1.100614842	1.177586012

Rectum Adenocarcinoma (READ)

Signature genes	Expression in tumor	Expression in rest of Pan-Cancer	P-value	Expression in tumor	Expression in normal tissue	P-value	Matrisome category	Fold change vs. Pan-Cancer	Fold change vs. normal
**ACAN**	8.121383495	6.323319026	4.39E-14	8.121383495	4.5152	5.36E-10	PROTEOGLYCANS	1.284354539	1.798676359
**ADAMTS12**	8.976704854	7.868232738	1.90E-06	8.976704854	6.25624	1.72E-06	ECM_REGULATORS	1.140879427	1.434840232
**ADAMTS14**	7.777338835	6.534747141	2.43E-09	7.777338835	6.35842	0.000155491	ECM_REGULATORS	1.190151458	1.223155884
**ADAMTS2**	9.875135922	9.391779411	0.018401857	9.875135922	7.97738	0.000365197	ECM_REGULATORS	1.051465914	1.23789213
**ADAMTSL5**	7.255732039	6.033847598	5.07E-10	7.255732039	6.23908	0.000935168	ECM_REGULATORS	1.202505022	1.162949031
**AGT**	9.451233981	8.416494983	0.004526609	9.451233981	7.08583	6.47E-06	ECM_REGULATORS	1.122941795	1.333821723
**AMELX**	2.64708835	0.194335534	6.29E-252	2.64708835	0.11755	2.29E-05	ECM_GLYCOPROTEINS	13.62122665	22.518829
**ANXA3**	11.47964563	8.926245599	1.13E-21	11.47964563	10.30756	4.52E-06	ECM_AFFILIATED	1.286055319	1.11371126
**ANXA4**	13.23804854	11.82699493	2.91E-25	13.23804854	12.90817	0.033980463	ECM_AFFILIATED	1.119307873	1.025555795
**AREG**	11.25790291	6.670819537	6.37E-48	11.25790291	10.0434	0.026168835	SECRETED_FACTORS	1.687634158	1.120925475
**BMP4**	10.89172427	7.75824932	9.47E-62	10.89172427	9.50031	0.002082775	SECRETED_FACTORS	1.403889437	1.146459881
**BMP7**	9.837092233	7.932301201	5.28E-07	9.837092233	8.0239	0.002984458	SECRETED_FACTORS	1.240130951	1.225973932
**CCL20**	10.43490388	5.551004688	4.54E-52	10.43490388	8.93931	0.00264275	SECRETED_FACTORS	1.879822567	1.167305294
**CCL24**	8.813801942	2.305375744	3.47E-210	8.813801942	7.60971	0.027168822	SECRETED_FACTORS	3.823152024	1.158230989
**COL10A1**	7.721826214	6.694443984	0.005019957	7.721826214	2.16437	9.59E-07	COLLAGENS	1.153467896	3.567701555
**COL11A1**	8.975939806	7.317275999	1.52E-05	8.975939806	4.44583	1.60E-07	COLLAGENS	1.22667777	2.018957046
**COL12A1**	12.51784175	11.1207018	5.55E-08	12.51784175	11.27396	0.006744425	COLLAGENS	1.125634153	1.110332283
**COL1A1**	15.60103883	14.56758602	0.000803474	15.60103883	13.49192	0.000170636	COLLAGENS	1.07094194	1.156324588
**COL1A2**	15.23048738	14.3745589	0.001435371	15.23048738	14.13734	0.024545588	COLLAGENS	1.059544678	1.077323413
**COL4A1**	13.68104563	13.10463265	0.002643182	13.68104563	12.41511	0.000737651	COLLAGENS	1.043985436	1.101967331
**COL4A1**	13.68104563	13.10463265	0.002643182	13.68104563	12.41511	0.000737651	BASEMENT_MEMBRANES	1.043985436	1.101967331
**COL5A1**	12.7487699	11.66527755	9.19E-06	12.7487699	11.41928	0.002752162	COLLAGENS	1.092881832	1.11642502
**COL5A2**	12.24646117	11.39125581	0.000123375	12.24646117	10.88569	0.001757219	COLLAGENS	1.075075599	1.125005504
**COL9A3**	8.705231068	6.830510682	9.21E-14	8.705231068	6.76355	0.00176223	COLLAGENS	1.274462697	1.287080168
**CSF2**	3.044507767	1.948265976	4.79E-07	3.044507767	1.07041	0.000920983	SECRETED_FACTORS	1.562675633	2.844244511
**CSF3**	4.945138835	2.809586086	3.72E-14	4.945138835	3.2849	0.026547626	SECRETED_FACTORS	1.760095147	1.505415335
**CST1**	8.289738835	4.675118846	2.47E-20	8.289738835	2.1563	1.70E-08	ECM_REGULATORS	1.773161091	3.844427415
**CST2**	4.538435922	3.474976626	0.000155135	4.538435922	1.00058	9.63E-07	ECM_REGULATORS	1.306033511	4.535805155
**CST4**	2.991103883	2.050409364	0.000179375	2.991103883	0.37357	0.000220894	ECM_REGULATORS	1.458783761	8.006809657
**CSTL1**	1.114260194	0.510782866	4.90E-11	1.114260194	0	0.001501164	ECM_REGULATORS	2.181475279	Inf
**CTSV**	9.022127184	7.231072045	9.79E-12	9.022127184	7.6191	0.00094036	ECM_REGULATORS	1.247688742	1.184146052
**CXCL1**	10.30440291	6.556732015	1.62E-32	10.30440291	8.08621	6.20E-05	SECRETED_FACTORS	1.571576036	1.274317995
**CXCL11**	7.212341748	6.342739488	0.001063379	7.212341748	4.78896	8.75E-05	SECRETED_FACTORS	1.137101999	1.506035078
**CXCL2**	8.884470874	6.828612117	3.19E-16	8.884470874	7.1593	0.000476769	SECRETED_FACTORS	1.301065388	1.240969211
**CXCL3**	9.6049	4.889910098	6.54E-94	9.6049	7.42962	1.45E-05	SECRETED_FACTORS	1.964228341	1.292784826
**CXCL5**	7.498083495	4.732781069	2.21E-18	7.498083495	4.50304	0.003513402	SECRETED_FACTORS	1.584286994	1.665115898
**CXCL6**	5.950331068	4.336360895	6.17E-07	5.950331068	4.65486	0.049647524	SECRETED_FACTORS	1.372194615	1.278305055
**CXCL8**	9.977666019	7.779989041	1.05E-15	9.977666019	7.32096	0.000264803	SECRETED_FACTORS	1.282478159	1.362890389
**EPYC**	3.355069903	2.536326265	0.003234883	3.355069903	0.37402	0.001558463	PROTEOGLYCANS	1.322806908	8.970295447
**F12**	8.619692233	7.100190636	1.05E-09	8.619692233	7.77414	0.024367298	ECM_REGULATORS	1.214008563	1.108764729
**F2**	3.100887379	2.08297719	0.003869562	3.100887379	0.84363	0.005895919	ECM_REGULATORS	1.48868043	3.675648541
**F7**	4.950301942	3.873236448	0.000364096	4.950301942	2.71598	0.00172469	ECM_REGULATORS	1.278078942	1.82265773

**FGF18**	5.941838835	4.782199904	4.22E-07	5.941838835	3.92801	7.18E-06	SECRETED_FACTORS	1.242490685	1.512684243
**FGF19**	4.928401942	1.643065402	4.15E-38	4.928401942	0.7498	1.63E-05	SECRETED_FACTORS	2.999516596	6.572955377
**FGF20**	2.666450485	1.844686926	6.89E-06	2.666450485	0.66708	0.029385049	SECRETED_FACTORS	1.445475895	3.997197466
**FGF23**	1.0403	0.469638924	9.46E-08	1.0403	0.07043	0.048989808	SECRETED_FACTORS	2.215106002	14.77069431
**FGF4**	1.201363107	0.406171312	4.61E-08	1.201363107	0.24826	0.011443495	SECRETED_FACTORS	2.957774398	4.839132791
**LAMC2**	12.39525922	9.831347513	6.97E-11	12.39525922	11.55936	0.005519794	ECM_GLYCOPROTEINS	1.26078945	1.072313625
**LAMC2**	12.39525922	9.831347513	6.97E-11	12.39525922	11.55936	0.005519794	BASEMENT_MEMBRANES	1.26078945	1.072313625
**LOXL2**	10.8055301	10.07428463	3.11E-05	10.8055301	9.27297	6.24E-05	ECM_REGULATORS	1.072585349	1.165271763
**MMP1**	9.842854369	6.627750723	3.27E-16	9.842854369	6.36902	8.99E-05	ECM_REGULATORS	1.48509725	1.545426827
**MMP10**	5.890628155	4.533528019	0.000210496	5.890628155	3.1467	0.000606095	ECM_REGULATORS	1.299347469	1.872001829
**MMP11**	11.32738252	9.858270961	2.86E-07	11.32738252	7.73634	2.58E-08	ECM_REGULATORS	1.149023249	1.464178478
**MMP20**	1.184235922	0.592134173	3.78E-07	1.184235922	0.19067	0.018847858	ECM_REGULATORS	1.999945243	6.210918982
**MMP3**	9.020161165	4.142292602	6.47E-40	9.020161165	4.97275	2.72E-05	ECM_REGULATORS	2.177577016	1.813918087
**MMP7**	9.135129126	8.135262776	0.01469889	9.135129126	3.79349	1.09E-08	ECM_REGULATORS	1.122905231	2.408106816
**MMP8**	2.446820388	1.926423533	0.021713963	2.446820388	0.32454	0.000617017	ECM_REGULATORS	1.270136264	7.539349197
**MMP9**	10.14470097	9.293933642	0.003084157	10.14470097	9.02294	0.038340081	ECM_REGULATORS	1.091540069	1.124323222
**OVGP1**	7.375123301	6.287084938	8.34E-11	7.375123301	6.13266	0.000145768	ECM_AFFILIATED	1.173059275	1.20259778
**PLOD3**	12.61662621	11.61495715	3.72E-22	12.61662621	11.3423	8.06E-08	ECM_REGULATORS	1.086239583	1.112351658
**PODNL1**	7.089570874	6.538444441	0.013002392	7.089570874	5.7042	0.000324086	PROTEOGLYCANS	1.084290145	1.242868566
**PRSS2**	4.21358835	2.974642102	0.000368052	4.21358835	1.02447	0.003754459	ECM_REGULATORS	1.416502626	4.112944595
**REG1A**	8.111842718	2.150232005	1.82E-63	8.111842718	2.76355	0.000375585	ECM_AFFILIATED	3.77254301	2.935297975
**REG1B**	5.031982524	0.604387787	1.16E-111	5.031982524	1.36788	0.005630639	ECM_AFFILIATED	8.3257515	3.678672489
**REG3A**	6.186950485	0.691989679	1.39E-119	6.186950485	1.66069	0.001777828	ECM_AFFILIATED	8.940813242	3.725530042
**SDC4**	13.36613495	12.80251828	0.001357438	13.36613495	12.6837	0.003653586	ECM_AFFILIATED	1.044023891	1.053804091
**SERPINA4**	3.886332039	2.266563977	3.19E-06	3.886332039	0.25052	1.30E-05	ECM_REGULATORS	1.714635932	15.51306099
**SERPINB5**	9.847217476	5.535738191	5.12E-19	9.847217476	8.4504	0.026939751	ECM_REGULATORS	1.778844507	1.165296019
**SERPINE2**	11.52373301	10.74347755	0.001017733	11.52373301	10.41633	0.003728025	ECM_REGULATORS	1.072625968	1.106314125
**SFTA2**	4.850459223	2.87030541	1.75E-08	4.850459223	0.84392	9.01E-07	ECM_AFFILIATED	1.689875651	5.747534391
**SPON2**	10.77283689	10.24663644	0.024005815	10.77283689	9.83802	0.004386635	ECM_GLYCOPROTEINS	1.051353482	1.095020837
**SRPX2**	9.234133981	7.587607153	8.55E-13	9.234133981	6.94982	1.97E-05	ECM_GLYCOPROTEINS	1.217002119	1.328686783
**SULF2**	12.57878058	11.71216418	1.99E-07	12.57878058	11.95181	0.021577686	ECM_REGULATORS	1.07399285	1.052458212
**TGFBI**	14.52063592	12.17238885	5.21E-32	14.52063592	11.6497	2.44E-09	ECM_GLYCOPROTEINS	1.192915877	1.246438614
**TSPEAR**	4.275168932	2.483923895	3.45E-18	4.275168932	2.73338	0.018454626	ECM_GLYCOPROTEINS	1.721135233	1.564059491
**WNT11**	8.472060194	5.899689413	1.01E-27	8.472060194	7.02067	0.024158487	SECRETED_FACTORS	1.436018	1.206731009
**WNT2**	7.47143301	5.44718666	1.39E-11	7.47143301	2.96796	5.57E-10	SECRETED_FACTORS	1.371613179	2.517363108
**VWA2**	8.569839806	5.360233535	1.40E-26	8.569839806	4.93176	4.92E-09	ECM_GLYCOPROTEINS	1.598781051	1.737683871
**ZP3**	8.856968932	7.783635916	1.93E-13	8.856968932	7.40735	1.90E-05	ECM_GLYCOPROTEINS	1.137896097	1.195700073

Sarcoma (SARC)

Signature genes	Expression in tumor	Expression in rest of Pan-Cancer	P-value	Expression in tumor	Expression in normal tissue	P-value	Matrisome category	Fold change vs. Pan-Cancer	Fold change vs. normal
**ADAM12**	10.89949015	8.386105051	3.56E-56	10.89949015	7.22335	0.032551242	ECM_REGULATORS	1.299708278	1.50892455
**ADAMTS6**	7.747486742	5.717910971	3.51E-76	7.747486742	5.252	0.030436854	ECM_REGULATORS	1.354950572	1.475149799
**ADAMTS7**	10.10905682	7.996091801	2.51E-111	10.10905682	7.2841	0.004434144	ECM_REGULATORS	1.26424972	1.387825101
**AMH**	6.441102652	4.809554736	2.85E-27	6.441102652	2.6229	0.019732308	SECRETED_FACTORS	1.339230554	2.455717965
**ANXA5**	14.19035417	13.35678082	1.95E-38	14.19035417	13.12465	0.033891719	ECM_AFFILIATED	1.062408252	1.081198673
**BGLAP**	4.464993561	4.085882626	2.50E-05	4.464993561	2.1235	0.028093098	ECM_GLYCOPROTEINS	1.092785567	2.102657669
**BMP1**	12.16980303	10.58460187	4.13E-122	12.16980303	10.2758	0.019172888	ECM_REGULATORS	1.149764835	1.184316844
**COL1A1**	18.04303598	14.62013411	1.35E-73	18.04303598	14.808	0.037129863	COLLAGENS	1.234122468	1.218465423
**COL1A2**	17.71674394	14.38451312	1.04E-92	17.71674394	15.02	0.04974141	COLLAGENS	1.231654057	1.179543538
**COL5A1**	14.92131023	11.71228787	4.24E-102	14.92131023	11.7459	0.017398671	COLLAGENS	1.273987661	1.270342011
**COL5A2**	14.23705114	11.41711507	2.59E-95	14.23705114	11.2083	0.004779968	COLLAGENS	1.246991999	1.270223953
**COL5A3**	11.24105492	9.395735251	3.29E-58	11.24105492	8.33285	0.041100551	COLLAGENS	1.196399709	1.349004833
**COL6A1**	16.40835038	13.11534005	8.46E-218	16.40835038	14.443	0.015269077	COLLAGENS	1.25108082	1.136076326
**COL6A1**	16.40835038	13.11534005	8.46E-218	16.40835038	14.443	0.015269077	BASEMENT_MEMBRANES	1.25108082	1.136076326
**COL6A2**	16.65895303	13.17268439	4.64E-172	16.65895303	14.4729	0.008329954	COLLAGENS	1.264658936	1.151044575
**COL6A2**	16.65895303	13.17268439	4.64E-172	16.65895303	14.4729	0.008329954	BASEMENT_MEMBRANES	1.264658936	1.151044575
**COL6A3**	15.57116515	12.53777336	4.14E-72	15.57116515	13.26485	0.044087104	BASEMENT_MEMBRANES	1.241940231	1.173866659
**COL6A3**	15.57116515	12.53777336	4.14E-72	15.57116515	13.26485	0.044087104	COLLAGENS	1.241940231	1.173866659
**CTHRC1**	10.79435	9.016966032	2.63E-29	10.79435	7.6714	0.047271785	ECM_GLYCOPROTEINS	1.197115522	1.407089971
**FBLN7**	9.52141553	7.237631285	1.00E-98	9.52141553	6.77405	0.023423373	ECM_GLYCOPROTEINS	1.315543049	1.405572077
**FBN1**	13.91629318	11.06802929	4.49E-134	13.91629318	11.3717	0.042504217	ECM_GLYCOPROTEINS	1.257341557	1.223765416
**FBN2**	10.41097576	7.095961652	3.40E-95	10.41097576	6.4834	0.022199914	ECM_GLYCOPROTEINS	1.467169112	1.605789517
**FN1**	17.24498068	15.10008369	1.02E-48	17.24498068	14.47775	0.013909119	ECM_GLYCOPROTEINS	1.142045371	1.191136791
**GPC2**	6.51081553	5.548603374	2.53E-13	6.51081553	3.4904	0.006192393	ECM_AFFILIATED	1.173415199	1.865349396
**HAPLN3**	9.578815152	8.507890854	5.42E-22	9.578815152	7.12765	0.02616298	PROTEOGLYCANS	1.12587424	1.343895274
**LAMA4**	13.19065265	10.84677364	5.35E-108	13.19065265	11.42485	0.048149884	ECM_GLYCOPROTEINS	1.216089972	1.15455806
**LAMA4**	13.19065265	10.84677364	5.35E-108	13.19065265	11.42485	0.048149884	BASEMENT_MEMBRANES	1.216089972	1.15455806
**LAMC1**	14.19221288	12.75481789	9.61E-69	14.19221288	12.84435	0.047868988	ECM_GLYCOPROTEINS	1.112694278	1.104938193
**LAMC1**	14.19221288	12.75481789	9.61E-69	14.19221288	12.84435	0.047868988	BASEMENT_MEMBRANES	1.112694278	1.104938193
**LGALS1**	14.94000682	12.62419549	1.07E-157	14.94000682	13.10245	0.015439831	ECM_AFFILIATED	1.18344229	1.140245284
**LOXL2**	12.28161061	10.10354265	4.11E-91	12.28161061	9.8336	0.028835334	ECM_REGULATORS	1.215574678	1.24894348
**MFAP2**	11.8167822	9.030276431	8.70E-62	11.8167822	9.1491	0.044804588	ECM_GLYCOPROTEINS	1.308573695	1.291578647
**MMP14**	15.24377083	12.86918534	3.17E-110	15.24377083	12.4847	0.002533468	ECM_REGULATORS	1.184517157	1.220996166
**NTN3**	2.861985985	2.082597484	3.01E-14	2.861985985	0	0.024550644	BASEMENT_MEMBRANES	1.374238664	Inf
**NTN3**	2.861985985	2.082597484	3.01E-14	2.861985985	0	0.024550644	ECM_GLYCOPROTEINS	1.374238664	Inf
**PLOD3**	12.31404697	11.6684407	8.07E-23	12.31404697	11.1919	0.040302748	ECM_REGULATORS	1.055329267	1.100264206
**PLXNA1**	11.93235492	11.50490271	7.92E-08	11.93235492	9.99535	0.003418437	ECM_AFFILIATED	1.037153918	1.193790605
**PLXND1**	12.96534848	11.83478347	5.30E-57	12.96534848	11.57105	0.023035923	ECM_AFFILIATED	1.095528998	1.120498873
**PXDN**	13.25719356	11.20015499	3.20E-75	13.25719356	11.33015	0.029560636	ECM_GLYCOPROTEINS	1.183661616	1.170081028
**SERPINH1**	14.63516136	12.62998523	1.80E-124	14.63516136	12.57145	0.00503397	ECM_REGULATORS	1.158763142	1.164158579
**TIMP2**	15.46104886	13.25271185	9.76E-151	15.46104886	14.0228	0.023940154	ECM_REGULATORS	1.16663284	1.102565027
**VCAN**	13.49171932	11.61817348	1.13E-37	13.49171932	10.5325	0.027976219	PROTEOGLYCANS	1.16125993	1.280960771

Stomach Adenocarcinoma (STAD)

Signature genes	Expression in tumor	Expression in rest of Pan-Cancer	P-value	Expression in tumor	Expression in normal tissue	P-value	Matrisome category	Fold change vs. Pan-Cancer	Fold change vs. normal
**ACAN**	8.278879556	6.323319026	8.86E-64	8.278879556	4.600433333	9.35E-27	PROTEOGLYCANS	1.309261722	1.799586899
**ADAM10**	12.388144	12.04475783	5.17E-22	12.388144	11.46264444	5.49E-14	ECM_REGULATORS	1.02850918	1.080740492
**ADAM12**	8.847993556	8.421758121	0.000639662	8.847993556	5.682261111	2.50E-21	ECM_REGULATORS	1.050611218	1.557125479
**ADAM30**	0.736926889	0.122202466	7.03E-121	0.736926889	0.242536111	0.040059193	ECM_REGULATORS	6.03037658	3.038421312
**ADAM8**	10.00042489	8.821557749	8.80E-43	10.00042489	8.363797222	4.78E-12	ECM_REGULATORS	1.133634804	1.195679979
**ADAM9**	13.004724	11.71561116	2.79E-96	13.004724	12.33549167	7.09E-05	ECM_REGULATORS	1.110033768	1.054252587
**ADAMDEC1**	8.29156	5.850329943	3.60E-79	8.29156	5.992755556	3.81E-10	ECM_REGULATORS	1.417280748	1.383597232
**ADAMTS12**	9.685602222	7.868232738	3.47E-58	9.685602222	6.703233333	2.55E-23	ECM_REGULATORS	1.230975563	1.444914975
**ADAMTS14**	8.340000444	6.534747141	3.46E-71	8.340000444	6.090702778	1.21E-20	ECM_REGULATORS	1.2762545	1.369300186
**ADAMTS18**	4.399408889	3.841406452	7.46E-07	4.399408889	1.812333333	5.52E-14	ECM_REGULATORS	1.145259931	2.427483293
**ADAMTS2**	10.93916133	9.391779411	2.39E-54	10.93916133	8.483675	4.98E-19	ECM_REGULATORS	1.164759185	1.289436634
**ADAMTS4**	10.26293044	9.106498629	2.26E-36	10.26293044	9.804319444	0.030943719	ECM_REGULATORS	1.126989731	1.046776424
**ADAMTS6**	6.972587333	5.681847736	8.00E-51	6.972587333	5.693872222	4.09E-08	ECM_REGULATORS	1.227168988	1.224577416
**ADAMTS7**	9.007179778	8.00658547	6.33E-41	9.007179778	7.857366667	2.44E-09	ECM_REGULATORS	1.124971414	1.146335682
**ADAMTS9**	9.756672667	8.971298469	7.25E-19	9.756672667	8.892827778	1.82E-05	ECM_REGULATORS	1.087542979	1.097139505
**ADAMTSL2**	8.415504	8.196535852	0.009711787	8.415504	7.401125	3.46E-06	ECM_REGULATORS	1.026714718	1.137057407
**AEBP1**	13.21165	12.81990476	4.19E-05	13.21165	12.73315833	0.046740666	ECM_GLYCOPROTEINS	1.030557578	1.037578396
**AGRN**	13.67810778	13.28611182	4.52E-10	13.67810778	12.150275	2.95E-16	BASEMENT_MEMBRANES	1.029504189	1.125744708
**AGRN**	13.67810778	13.28611182	4.52E-10	13.67810778	12.150275	2.95E-16	ECM_GLYCOPROTEINS	1.029504189	1.125744708
**AGT**	10.06237956	8.416494983	7.73E-21	10.06237956	8.559566667	1.57E-05	ECM_REGULATORS	1.195554631	1.175571141
**AMELX**	1.047598889	0.194335534	6.80E-124	1.047598889	0.123505556	0.000113839	ECM_GLYCOPROTEINS	5.390670813	8.482200531
**AMH**	5.983361333	4.78537348	1.11E-24	5.983361333	3.845486111	4.06E-08	SECRETED_FACTORS	1.250343648	1.555944076
**ANGPT2**	8.988419333	8.572867687	7.50E-08	8.988419333	7.270194444	2.98E-16	SECRETED_FACTORS	1.048472887	1.23633823
**ANXA13**	8.118077111	3.513948788	6.63E-201	8.118077111	6.114466667	0.000117569	ECM_AFFILIATED	2.310243433	1.327683599
**ANXA2**	15.21144378	14.42235591	2.45E-26	15.21144378	14.64078889	0.000602821	ECM_AFFILIATED	1.054712827	1.038977059
**ANXA4**	13.40527578	11.82699493	3.37E-124	13.40527578	12.352425	9.78E-09	ECM_AFFILIATED	1.133447326	1.085234339
**ANXA9**	7.681538667	7.394649341	0.004400053	7.681538667	6.813055556	0.001951112	ECM_AFFILIATED	1.03879688	1.127473364
**ASPN**	10.49579844	8.261593676	1.41E-75	10.49579844	9.095838889	1.20E-05	PROTEOGLYCANS	1.270432662	1.153912088
**ASTL**	2.499896667	1.883944398	1.82E-16	2.499896667	1.901575	0.022822351	ECM_REGULATORS	1.32694822	1.314645316
**BDNF**	5.546703111	5.227870663	0.001870225	5.546703111	5.011527778	0.019961389	SECRETED_FACTORS	1.060987057	1.106788859
**BGN**	13.84422956	13.22871855	2.06E-10	13.84422956	11.46079167	8.30E-20	PROTEOGLYCANS	1.046528392	1.207964507
**BMP1**	10.882524	10.62402635	1.59E-06	10.882524	9.590019444	2.81E-18	ECM_REGULATORS	1.02433142	1.13477601
**BMP8A**	7.135248222	4.035349936	5.70E-269	7.135248222	5.140386111	1.31E-14	SECRETED_FACTORS	1.768185742	1.388076317
**BMP8B**	8.574716222	7.691986097	2.31E-26	8.574716222	8.096230556	0.02546795	SECRETED_FACTORS	1.114759714	1.059099807
**C1QB**	12.45423289	11.63344026	4.62E-20	12.45423289	11.96248889	0.043090101	ECM_AFFILIATED	1.070554591	1.041107165
**C1QC**	12.16901911	11.46874185	5.16E-16	12.16901911	11.65993333	0.029118777	ECM_AFFILIATED	1.061059641	1.043661123
**C1QTNF5**	9.951939556	9.320140306	2.89E-15	9.951939556	9.348008333	0.00247881	ECM_AFFILIATED	1.067788599	1.064605336
**C1QTNF8**	0.637034	0.303833429	1.83E-16	0.637034	0.184544444	0.013611429	ECM_AFFILIATED	2.096655401	3.451927268
**CCL15**	7.275021778	2.223574734	1.49E-244	7.275021778	4.281536111	2.07E-10	SECRETED_FACTORS	3.271768502	1.699161607
**CCL18**	9.123665556	6.923515264	1.12E-41	9.123665556	7.521652778	3.98E-05	SECRETED_FACTORS	1.317779366	1.212986803
**CCL20**	9.637511111	5.551004688	5.41E-148	9.637511111	7.533080556	3.32E-06	SECRETED_FACTORS	1.736174198	1.279358563
**CCL22**	7.707134889	5.887581409	2.34E-56	7.707134889	6.867469444	0.003011668	SECRETED_FACTORS	1.309049396	1.122267081

**CCL26**	4.449234889	3.004064052	7.58E-53	4.449234889	3.557566667	0.0055161	SECRETED_FACTORS	1.481071912	1.250639919
**CCL3L3**	5.978905556	5.520878763	8.70E-07	5.978905556	4.094302778	2.60E-08	SECRETED_FACTORS	1.082962661	1.46029883
**CCL4**	8.670872889	7.670621992	7.83E-34	8.670872889	7.705441667	0.000166742	SECRETED_FACTORS	1.130400233	1.125292133
**CCL4L2**	7.397370889	6.472151775	1.37E-22	7.397370889	5.884366667	4.55E-06	SECRETED_FACTORS	1.142953865	1.257122696
**CCL7**	2.993413111	2.256527657	2.15E-13	2.993413111	0.602138889	2.98E-11	SECRETED_FACTORS	1.326557244	4.971300088
**CLCF1**	8.466583333	7.683445344	3.46E-19	8.466583333	7.924052778	0.004851927	SECRETED_FACTORS	1.101925367	1.068466298
**CLEC12A**	5.365190889	5.125828423	0.021386655	5.365190889	4.759672222	0.021714527	ECM_AFFILIATED	1.046697323	1.127218564
**CLEC19A**	0.755869333	0.215233557	3.02E-83	0.755869333	0.34615	0.0234625	ECM_AFFILIATED	3.511856355	2.183646781
**CLEC4A**	6.372682	6.157361533	0.000444025	6.372682	5.747713889	0.001288989	ECM_AFFILIATED	1.0349696	1.108733337
**CLEC4C**	1.358348	1.146013977	0.001811117	1.358348	0.820880556	0.024102345	ECM_AFFILIATED	1.185280482	1.654745006
**CLEC4E**	6.200654667	5.399081813	9.80E-18	6.200654667	5.540661111	0.021048406	ECM_AFFILIATED	1.148464661	1.119118196
**CLEC6A**	2.161366889	1.388828189	6.59E-25	2.161366889	1.268141667	0.001362805	ECM_AFFILIATED	1.556252175	1.704357601
**CLEC7A**	8.015342222	7.78728395	0.007319763	8.015342222	7.28485	0.000402668	ECM_AFFILIATED	1.029285984	1.100275534
**CNTF**	4.351806889	4.014342028	1.95E-07	4.351806889	3.767913889	0.00138598	SECRETED_FACTORS	1.0840648	1.154964529
**COCH**	7.461280222	6.377181409	6.96E-19	7.461280222	6.107552778	0.000284567	ECM_GLYCOPROTEINS	1.169996546	1.221648096
**COL10A1**	8.806952222	6.694443984	1.68E-32	8.806952222	2.843163889	1.56E-26	COLLAGENS	1.315561418	3.097588661
**COL11A1**	7.916312889	7.317275999	0.001265841	7.916312889	2.879758333	1.71E-19	COLLAGENS	1.081866106	2.748950423
**COL12A1**	13.06926	11.1207018	2.63E-55	13.06926	11.40725278	4.92E-11	COLLAGENS	1.175218996	1.145697413
**COL15A1**	12.49456422	10.32914512	5.53E-91	12.49456422	11.86424722	0.003117688	COLLAGENS	1.209641657	1.053127433
**COL15A1**	12.49456422	10.32914512	5.53E-91	12.49456422	11.86424722	0.003117688	BASEMENT_MEMBRANES	1.209641657	1.053127433
**COL1A1**	16.64813133	14.56758602	2.66E-44	16.64813133	13.83975278	2.43E-20	COLLAGENS	1.142820184	1.20292115
**COL1A2**	15.78739756	14.3745589	1.04E-27	15.78739756	13.98214444	2.27E-12	COLLAGENS	1.098287444	1.129111319
**COL27A1**	10.41805889	9.554815529	1.51E-23	10.41805889	9.362458333	5.29E-06	COLLAGENS	1.090346418	1.112748225
**COL3A1**	15.90301956	14.11161066	7.60E-36	15.90301956	13.97098889	6.14E-13	COLLAGENS	1.126945743	1.138288756
**COL4A1**	14.312298	13.10463265	8.31E-39	14.312298	12.67856389	3.70E-15	BASEMENT_MEMBRANES	1.092155605	1.128857978
**COL4A1**	14.312298	13.10463265	8.31E-39	14.312298	12.67856389	3.70E-15	COLLAGENS	1.092155605	1.128857978
**COL4A2**	14.33370444	13.38834677	5.52E-26	14.33370444	13.50583611	3.76E-05	BASEMENT_MEMBRANES	1.070610486	1.061297081
**COL4A2**	14.33370444	13.38834677	5.52E-26	14.33370444	13.50583611	3.76E-05	COLLAGENS	1.070610486	1.061297081
**COL5A1**	13.50273756	11.66527755	1.64E-54	13.50273756	12.01277222	6.80E-10	COLLAGENS	1.157515327	1.124031764
**COL5A2**	12.74541111	11.39125581	2.49E-36	12.74541111	10.85533333	1.39E-16	COLLAGENS	1.118876735	1.17411513
**COL5A3**	9.881122667	9.425908801	4.62E-07	9.881122667	8.778491667	1.01E-07	COLLAGENS	1.048293897	1.125605974
**COL6A3**	14.51064333	12.4598605	8.50E-54	14.51064333	13.19758056	7.39E-08	COLLAGENS	1.164591155	1.099492689
**COL6A3**	14.51064333	12.4598605	8.50E-54	14.51064333	13.19758056	7.39E-08	BASEMENT_MEMBRANES	1.164591155	1.099492689
**COL7A1**	9.744857111	9.209955931	3.87E-05	9.744857111	7.794313889	2.76E-10	COLLAGENS	1.058078582	1.250252075
**COL8A1**	10.79210911	9.734282292	4.42E-16	10.79210911	8.962394444	3.22E-08	COLLAGENS	1.108670243	1.204154668
**COL9A1**	6.450487111	3.736297098	1.38E-87	6.450487111	5.324941667	0.004205413	COLLAGENS	1.726438488	1.211372352
**CRLF3**	9.634607778	8.988405963	8.11E-53	9.634607778	9.116372222	3.03E-07	SECRETED_FACTORS	1.071892816	1.056846687
**CSF2**	3.302427111	1.948265976	4.62E-37	3.302427111	0.944919444	1.38E-10	SECRETED_FACTORS	1.695059685	3.494929785
**CSH2**	0.594325778	0.421791061	0.00155685	0.594325778	0	0.005088807	SECRETED_FACTORS	1.409052569	Inf
**CST1**	9.709013556	4.675118846	2.64E-150	9.709013556	2.778716667	5.69E-29	ECM_REGULATORS	2.076741549	3.494063886
**CST2**	5.754231778	3.474976626	5.65E-62	5.754231778	2.942344444	1.26E-11	ECM_REGULATORS	1.655905175	1.955662189
**CST4**	3.510344444	2.050409364	1.58E-32	3.510344444	0.336883333	6.17E-13	ECM_REGULATORS	1.71202127	10.4200597
**CSTL1**	1.122179556	0.510782866	2.11E-40	1.122179556	0.398558333	0.001680396	ECM_REGULATORS	2.196979639	2.815596769
**CTHRC1**	9.938579333	9.01501605	1.02E-13	9.938579333	6.815844444	4.43E-24	ECM_GLYCOPROTEINS	1.102447215	1.45815818
**CTSA**	13.68244667	12.74033457	7.01E-98	13.68244667	12.935775	1.10E-07	ECM_REGULATORS	1.073947203	1.057721448
**CTSC**	12.74138356	12.11148266	4.42E-17	12.74138356	11.80356389	4.84E-10	ECM_REGULATORS	1.05200857	1.079452246

**CTSD**	15.30379733	15.17742801	0.015206619	15.30379733	14.86923333	0.001033208	ECM_REGULATORS	1.008326136	1.029225717
**CTSH**	12.65691889	12.1334887	3.32E-14	12.65691889	11.99398889	0.000511006	ECM_REGULATORS	1.043139298	1.055271854
**CTSK**	11.20556733	10.28017202	4.85E-20	11.20556733	10.68377222	0.010118662	ECM_REGULATORS	1.090017493	1.04883997
**CTSS**	12.70831622	11.2836481	5.68E-83	12.70831622	11.78918056	1.83E-06	ECM_REGULATORS	1.126259532	1.077964339
**CTSV**	8.511077778	7.231072045	8.96E-24	8.511077778	6.710436111	6.55E-08	ECM_REGULATORS	1.177014656	1.268334522
**CXCL1**	10.35122733	6.556732015	1.83E-133	10.35122733	7.379027778	1.01E-13	SECRETED_FACTORS	1.578717463	1.40279013
**CXCL10**	9.251250444	8.345707122	5.19E-12	9.251250444	7.073405556	6.91E-10	SECRETED_FACTORS	1.108504086	1.307891987
**CXCL11**	7.910064444	6.342739488	6.47E-34	7.910064444	5.419202778	2.20E-11	SECRETED_FACTORS	1.247105365	1.459636181
**CXCL13**	8.765910889	6.191608355	2.11E-55	8.765910889	7.303997222	0.001084193	SECRETED_FACTORS	1.415772831	1.20015255
**CXCL2**	9.360149778	6.828612117	1.53E-94	9.360149778	8.613730556	0.014581648	SECRETED_FACTORS	1.370725063	1.086654582
**CXCL3**	9.554197556	4.889910098	0	9.554197556	7.287705556	2.74E-09	SECRETED_FACTORS	1.953859553	1.311002137
**CXCL5**	9.53692	4.732781069	1.67E-200	9.53692	7.111666667	0.000316455	SECRETED_FACTORS	2.015077364	1.341024607
**CXCL6**	6.366538222	4.336360895	4.18E-38	6.366538222	3.777161111	5.06E-09	SECRETED_FACTORS	1.468175361	1.685535256
**CXCL8**	10.433952	7.779989041	2.64E-87	10.433952	7.613883333	7.65E-12	SECRETED_FACTORS	1.341126825	1.370385064
**CXCL9**	10.74646378	8.857239179	2.95E-39	10.74646378	8.282894444	6.14E-11	SECRETED_FACTORS	1.213297232	1.297428556
**DMBT1**	11.99001533	4.525549118	0	11.99001533	10.16876944	0.012122999	ECM_GLYCOPROTEINS	2.649405635	1.179101896
**DMP1**	2.035884889	1.378079634	1.36E-19	2.035884889	0.480102778	8.27E-07	ECM_GLYCOPROTEINS	1.477334719	4.24051887
**DSPP**	1.123386667	0.492041358	3.27E-49	1.123386667	0.292394444	0.000736743	ECM_GLYCOPROTEINS	2.28311431	3.842024662
**EGFL6**	6.883437333	5.7928	2.16E-13	6.883437333	4.733863889	1.99E-11	SECRETED_FACTORS	1.18827464	1.454084337
**ELFN1**	7.415106889	6.692110066	1.82E-15	7.415106889	6.562619444	1.25E-06	ECM_AFFILIATED	1.108037198	1.129900484
**EMILIN2**	9.602762444	9.320936395	0.000151632	9.602762444	8.726152778	2.61E-07	ECM_GLYCOPROTEINS	1.030235809	1.100457749
**EPYC**	2.809829111	2.536326265	0.042743585	2.809829111	0.821438889	8.07E-06	PROTEOGLYCANS	1.107834252	3.420618562
**EYS**	5.382029111	4.186296216	1.29E-67	5.382029111	4.80965	0.001866105	ECM_GLYCOPROTEINS	1.285630264	1.119006396
**F12**	7.983059778	7.100190636	2.32E-13	7.983059778	6.4057	6.84E-07	ECM_REGULATORS	1.124344428	1.246243155
**F2**	2.588225333	2.08297719	0.002941795	2.588225333	1.707352778	0.028329502	ECM_REGULATORS	1.242560574	1.51592885
**FASLG**	5.173983778	4.123316518	1.53E-27	5.173983778	4.484719444	0.012744792	SECRETED_FACTORS	1.254811207	1.15369174
**FBN1**	12.23087289	11.07984782	1.87E-36	12.23087289	11.70960833	0.032306184	ECM_GLYCOPROTEINS	1.103884556	1.044515968
**FBN2**	7.556540444	7.144258567	0.001216384	7.556540444	6.754675	0.002738049	ECM_GLYCOPROTEINS	1.057708141	1.118712661
**FGF18**	6.339259556	4.782199904	1.43E-44	6.339259556	5.788811111	0.044051313	SECRETED_FACTORS	1.325594848	1.095088341
**FGF19**	3.034992	1.643065402	4.96E-29	3.034992	0.393547222	8.85E-08	SECRETED_FACTORS	1.847152278	7.711887745
**FGF20**	2.316928444	1.844686926	1.48E-07	2.316928444	1.284263889	0.0120597	SECRETED_FACTORS	1.256000903	1.804090627
**FGF23**	1.570236889	0.469638924	8.85E-89	1.570236889	0.893605556	0.044880985	SECRETED_FACTORS	3.34349818	1.757192398
**FGF3**	0.985761556	0.3792103	1.18E-21	0.985761556	0.169688889	0.014830767	SECRETED_FACTORS	2.599511554	5.809228654
**FGF4**	1.301984889	0.406171312	4.31E-36	1.301984889	0.777297222	0.046871838	SECRETED_FACTORS	3.205506769	1.675015492
**FNDC1**	10.21330578	8.021472757	8.65E-48	10.21330578	6.933008333	1.44E-14	ECM_GLYCOPROTEINS	1.27324571	1.473142002
**FNDC8**	1.37334	1.161960077	1.24E-05	1.37334	0.842011111	0.005747283	ECM_GLYCOPROTEINS	1.181916683	1.631023608
**HABP2**	8.261086889	4.336069366	1.56E-95	8.261086889	6.897988889	0.019524205	ECM_REGULATORS	1.905201737	1.197608031
**HAPLN3**	9.011324	8.519183557	1.74E-08	9.011324	7.544372222	5.29E-12	PROTEOGLYCANS	1.057768499	1.194443187
**HMCN1**	9.091628444	8.682149989	0.000178737	9.091628444	8.467694444	0.033291012	ECM_GLYCOPROTEINS	1.047163255	1.073684048
**HMCN1**	9.091628444	8.682149989	0.000178737	9.091628444	8.467694444	0.033291012	BASEMENT_MEMBRANES	1.047163255	1.073684048
**HPSE**	8.955951333	7.671407813	9.42E-67	8.955951333	7.741355556	6.83E-09	ECM_REGULATORS	1.167445605	1.156897041
**HSPG2**	14.97598978	13.25809189	2.48E-130	14.97598978	14.51063056	0.019386653	PROTEOGLYCANS	1.129573539	1.032070227
**HSPG2**	14.97598978	13.25809189	2.48E-130	14.97598978	14.51063056	0.019386653	BASEMENT_MEMBRANES	1.129573539	1.032070227
**HTRA3**	10.85178089	9.118383418	9.67E-49	10.85178089	10.22499167	0.013140961	ECM_REGULATORS	1.190099208	1.061299729
**HTRA4**	3.934204222	3.38454054	4.68E-09	3.934204222	1.358152778	2.83E-18	ECM_REGULATORS	1.162404225	2.896731713
**HYAL4**	1.894647556	1.270187372	2.67E-13	1.894647556	1.178613889	0.012489327	ECM_REGULATORS	1.491628398	1.60752183

**IBSP**	3.688607111	3.204938584	0.000153379	3.688607111	0.204741667	1.69E-15	ECM_GLYCOPROTEINS	1.150913509	18.01590839
**IGFBP1**	3.393523111	3.017202849	0.023932315	3.393523111	0.921875	1.84E-07	ECM_GLYCOPROTEINS	1.12472488	3.681109815
**IGFBP3**	12.84558	12.42794167	6.25E-05	12.84558	11.58416944	8.76E-09	ECM_GLYCOPROTEINS	1.033604787	1.108890893
**IGFBP7**	14.015228	13.4033764	8.28E-23	14.015228	13.37991944	0.000902061	ECM_GLYCOPROTEINS	1.045649065	1.047482241
**IMPG1**	3.517167333	2.643124979	1.26E-32	3.517167333	2.164197222	5.70E-09	PROTEOGLYCANS	1.330685216	1.625160266
**KCP**	6.559173333	5.94710912	9.85E-08	6.559173333	5.566794444	0.000110822	ECM_GLYCOPROTEINS	1.102917939	1.178267565
**LAMA3**	11.85473089	9.61444257	3.10E-68	11.85473089	10.91919444	0.000760023	ECM_GLYCOPROTEINS	1.233012814	1.085678156
**LAMA3**	11.85473089	9.61444257	3.10E-68	11.85473089	10.91919444	0.000760023	BASEMENT_MEMBRANES	1.233012814	1.085678156
**LAMA5**	13.15700356	12.30493433	1.31E-36	13.15700356	12.47625278	0.002165853	ECM_GLYCOPROTEINS	1.069246141	1.054563721
**LAMA5**	13.15700356	12.30493433	1.31E-36	13.15700356	12.47625278	0.002165853	BASEMENT_MEMBRANES	1.069246141	1.054563721
**LAMB1**	12.69907889	12.17469109	1.11E-10	12.69907889	11.81490833	9.44E-09	ECM_GLYCOPROTEINS	1.043071959	1.07483516
**LAMB1**	12.69907889	12.17469109	1.11E-10	12.69907889	11.81490833	9.44E-09	BASEMENT_MEMBRANES	1.043071959	1.07483516
**LAMB3**	12.75482422	10.59206818	4.29E-55	12.75482422	11.19830556	4.78E-08	ECM_GLYCOPROTEINS	1.204186379	1.13899591
**LAMB3**	12.75482422	10.59206818	4.29E-55	12.75482422	11.19830556	4.78E-08	BASEMENT_MEMBRANES	1.204186379	1.13899591
**LAMC1**	13.27702556	12.78046788	1.71E-14	13.27702556	12.90646389	0.041783618	BASEMENT_MEMBRANES	1.038852856	1.028711324
**LAMC1**	13.27702556	12.78046788	1.71E-14	13.27702556	12.90646389	0.041783618	ECM_GLYCOPROTEINS	1.038852856	1.028711324
**LAMC2**	12.40610222	9.831347513	4.91E-42	12.40610222	10.18286111	5.52E-14	BASEMENT_MEMBRANES	1.261892351	1.218331674
**LAMC2**	12.40610222	9.831347513	4.91E-42	12.40610222	10.18286111	5.52E-14	ECM_GLYCOPROTEINS	1.261892351	1.218331674
**LGALS2**	8.024652222	5.195708291	9.57E-98	8.024652222	6.975825	0.015234767	ECM_AFFILIATED	1.544477052	1.150351711
**LGALS4**	13.34649756	4.772168591	0	13.34649756	11.476125	0.000123982	ECM_AFFILIATED	2.79673639	1.162979451
**LGALS8**	11.67006911	11.28158572	2.06E-19	11.67006911	11.18103056	7.80E-07	ECM_AFFILIATED	1.034435176	1.043738236
**LGALS9**	12.45254667	10.7310062	2.93E-136	12.45254667	11.70815	0.000319805	ECM_AFFILIATED	1.160426752	1.063579359
**LOX**	10.39019089	9.477059811	6.34E-19	10.39019089	9.126238889	3.76E-09	ECM_REGULATORS	1.096351727	1.138496484
**LOXL1**	10.46247156	9.197010938	1.18E-39	10.46247156	9.898422222	0.003246313	ECM_REGULATORS	1.137594771	1.056983762
**LOXL2**	11.24972867	10.07428463	1.46E-43	11.24972867	9.805772222	3.32E-13	ECM_REGULATORS	1.116677668	1.14725576
**LTBP2**	11.99744933	11.2331707	2.87E-16	11.99744933	11.34945556	0.003094461	ECM_GLYCOPROTEINS	1.068037659	1.057094702
**LUM**	13.825864	11.42934962	1.43E-55	13.825864	12.93931944	0.000267376	PROTEOGLYCANS	1.209680731	1.068515547
**MATN3**	7.007406667	6.493302349	2.07E-06	7.007406667	5.030638889	1.55E-09	ECM_GLYCOPROTEINS	1.079174554	1.392945672
**MFAP2**	9.757172	9.073903157	3.13E-07	9.757172	7.861247222	8.36E-12	ECM_GLYCOPROTEINS	1.075300434	1.241173534
**MMP1**	10.780812	6.627750723	7.17E-104	10.780812	7.7176	1.44E-10	ECM_REGULATORS	1.626617	1.396912512
**MMP10**	5.874649333	4.533528019	3.21E-14	5.874649333	3.301372222	4.46E-10	ECM_REGULATORS	1.29582288	1.779456825
**MMP11**	11.03384422	9.858270961	2.06E-17	11.03384422	7.622777778	1.60E-20	ECM_REGULATORS	1.119247408	1.44748339
**MMP12**	9.484455778	5.270708918	4.38E-118	9.484455778	6.371233333	4.41E-10	ECM_REGULATORS	1.799464916	1.48863733
**MMP14**	13.32404622	12.90987096	1.11E-06	13.32404622	12.34501944	1.11E-09	ECM_REGULATORS	1.032082061	1.079305406
**MMP15**	12.28964956	10.64786643	2.23E-113	12.28964956	11.66650833	0.001560212	ECM_REGULATORS	1.154188929	1.05341283
**MMP19**	8.931064444	7.890906824	3.67E-35	8.931064444	8.414872222	0.007063884	ECM_REGULATORS	1.131817248	1.061342847
**MMP3**	7.836351111	4.142292602	2.57E-93	7.836351111	4.33435	8.51E-10	ECM_REGULATORS	1.891790818	1.807964542
**MMP7**	9.637948222	8.135262776	3.92E-14	9.637948222	6.045947222	2.73E-10	ECM_REGULATORS	1.184712589	1.594117161
**MMP8**	2.553130667	1.926423533	1.32E-08	2.553130667	0.400688889	1.90E-09	ECM_REGULATORS	1.325321573	6.37185292
**MMP9**	10.09875089	9.293933642	6.57E-09	10.09875089	7.727013889	3.43E-12	ECM_REGULATORS	1.086595975	1.306940952
**MUC1**	13.74801689	10.66424837	1.82E-82	13.74801689	12.69809444	0.00287361	ECM_AFFILIATED	1.289168857	1.082683465
**MUC12**	8.543143556	4.426734354	1.46E-258	8.543143556	6.098691667	7.50E-06	ECM_AFFILIATED	1.929897498	1.400815785
**MUC13**	13.34371733	3.871153997	0	13.34371733	10.29103333	4.26E-10	ECM_AFFILIATED	3.446961125	1.296635323
**MUC17**	11.16074044	0.657567474	0	11.16074044	9.240544444	0.007719749	ECM_AFFILIATED	16.97276839	1.207801176
**MUC3A**	12.06082533	5.869215593	0	12.06082533	9.356169444	1.73E-07	ECM_AFFILIATED	2.054929682	1.289077267
**MUC5B**	10.54449444	5.562827041	4.52E-123	10.54449444	8.696797222	0.006640958	ECM_AFFILIATED	1.895528005	1.212457204

**MXRA5**	13.03528978	10.90338756	1.34E-66	13.03528978	11.52289722	1.84E-10	ECM_GLYCOPROTEINS	1.195526592	1.131251067
**NGLY1**	10.07889067	9.959465731	2.38E-05	10.07889067	9.881375	0.025173668	ECM_REGULATORS	1.011991099	1.019988682
**NID2**	10.52273889	9.115427105	3.38E-50	10.52273889	9.176313889	3.99E-09	ECM_GLYCOPROTEINS	1.154387915	1.146728307
**NID2**	10.52273889	9.115427105	3.38E-50	10.52273889	9.176313889	3.99E-09	BASEMENT_MEMBRANES	1.154387915	1.146728307
**NTNG2**	6.394661778	5.95492044	1.25E-05	6.394661778	5.967911111	0.0361571	BASEMENT_MEMBRANES	1.07384504	1.071507544
**NTNG2**	6.394661778	5.95492044	1.25E-05	6.394661778	5.967911111	0.0361571	ECM_GLYCOPROTEINS	1.07384504	1.071507544
**OGFOD1**	10.62352556	10.45417434	1.94E-12	10.62352556	10.36175278	0.001127894	ECM_REGULATORS	1.016199387	1.025263368
**OIT3**	4.744170222	3.162342453	3.20E-44	4.744170222	3.593138889	0.000161971	ECM_GLYCOPROTEINS	1.500207613	1.320341453
**OTOG**	1.654464667	1.291497704	8.42E-05	1.654464667	0.418186111	4.02E-05	ECM_GLYCOPROTEINS	1.281043444	3.956287937
**PCOLCE**	11.01975444	10.58468347	6.65E-07	11.01975444	10.57139167	0.032211444	ECM_GLYCOPROTEINS	1.041103825	1.042412843
**PLAU**	11.13289267	10.53550347	7.81E-09	11.13289267	9.424855556	1.45E-14	ECM_REGULATORS	1.056702483	1.181226874
**PLOD3**	12.31784178	11.61495715	4.79E-44	12.31784178	10.86550556	1.97E-17	ECM_REGULATORS	1.060515473	1.133664855
**PLXDC1**	9.546927333	8.810221397	1.42E-26	9.546927333	8.699297222	8.15E-07	ECM_AFFILIATED	1.083619458	1.097436619
**PLXNA1**	12.011132	11.47403255	2.64E-18	12.011132	10.83508056	1.93E-14	ECM_AFFILIATED	1.046809999	1.108541089
**PLXNA3**	11.22321533	10.69586156	2.48E-23	11.22321533	10.233925	1.22E-09	ECM_AFFILIATED	1.049304468	1.096667733
**PLXNB1**	11.99301667	11.84458584	0.013241308	11.99301667	11.44999722	0.000518602	ECM_AFFILIATED	1.012531534	1.04742529
**PLXNB2**	14.38407889	13.6445309	5.78E-70	14.38407889	14.07644167	0.015299843	ECM_AFFILIATED	1.054201056	1.021854758
**PLXND1**	12.19908733	11.89022243	1.91E-08	12.19908733	11.29818333	1.12E-07	ECM_AFFILIATED	1.025976377	1.079738837
**PODNL1**	7.686152222	6.538444441	9.91E-27	7.686152222	6.407372222	8.55E-08	PROTEOGLYCANS	1.175532237	1.199579477
**POSTN**	12.85119822	10.68835551	1.23E-36	12.85119822	12.23900278	0.017231599	ECM_GLYCOPROTEINS	1.20235505	1.050020043
**PRSS3**	10.78949444	3.83222702	0	10.78949444	10.00613611	0.018032004	ECM_REGULATORS	2.815463277	1.078287795
**PXDN**	11.63657644	11.25497626	1.74E-05	11.63657644	10.57374722	1.58E-07	ECM_GLYCOPROTEINS	1.033905019	1.100515853
**REG4**	11.38451	1.936207302	0	11.38451	9.385597222	0.006036422	ECM_AFFILIATED	5.879799124	1.212976621
**SDC1**	13.06162622	12.36422045	4.77E-08	13.06162622	12.29052222	0.006301894	ECM_AFFILIATED	1.056405155	1.062739726
**SDC4**	13.43246667	12.80251828	1.11E-13	13.43246667	12.87870833	0.001041967	ECM_AFFILIATED	1.049205037	1.042997971
**SEMA4D**	10.76442178	10.40990058	1.56E-10	10.76442178	10.40602778	0.010007144	ECM_AFFILIATED	1.034056156	1.034441
**SEMA4G**	11.26400133	8.233080251	7.46E-261	11.26400133	9.982061111	4.39E-07	ECM_AFFILIATED	1.368139383	1.128424401
**SEMA5A**	9.894536667	9.681341964	0.031243534	9.894536667	9.446891667	0.037005266	ECM_AFFILIATED	1.022021193	1.047385427
**SEMA6B**	9.286624889	8.793950223	7.45E-12	9.286624889	8.677583333	0.000576141	ECM_AFFILIATED	1.056024273	1.070185619
**SEMA7A**	8.918727556	8.475128954	5.19E-08	8.918727556	8.173902778	0.00071903	ECM_AFFILIATED	1.052341221	1.091122295
**SERPINA1**	13.24141467	11.79513816	1.08E-19	13.24141467	11.41300833	1.60E-05	ECM_REGULATORS	1.122616326	1.160203715
**SERPINA4**	7.145585778	2.266563977	5.91E-176	7.145585778	5.688380556	0.010469421	ECM_REGULATORS	3.15260714	1.256172246
**SERPINB5**	10.87661533	5.535738191	6.80E-114	10.87661533	8.913827778	3.41E-05	ECM_REGULATORS	1.964799446	1.220195813
**SERPINB9**	10.28189844	9.199020844	7.86E-64	10.28189844	9.604122222	0.000315752	ECM_REGULATORS	1.117716616	1.070571387
**SERPINE1**	11.28036733	10.74768977	8.95E-07	11.28036733	9.449688889	1.17E-10	ECM_REGULATORS	1.049562052	1.193728965
**SERPINH1**	12.93389489	12.68452609	0.000241792	12.93389489	11.48178333	2.92E-19	ECM_REGULATORS	1.019659291	1.126470907
**SPON2**	11.24789489	10.24663644	4.49E-19	11.24789489	10.39352778	3.58E-06	ECM_GLYCOPROTEINS	1.097715817	1.08220184
**SRPX2**	8.381263333	7.587607153	7.22E-13	8.381263333	7.791197222	0.007089993	ECM_GLYCOPROTEINS	1.104599008	1.075734973
**ST14**	13.45612822	11.48217639	1.93E-57	13.45612822	11.97865556	2.56E-09	ECM_REGULATORS	1.171914432	1.123342111
**SULF1**	12.53068556	10.89942983	7.41E-43	12.53068556	10.20347778	5.49E-14	ECM_REGULATORS	1.149664318	1.228079859
**SULF2**	12.58987444	11.71216418	1.57E-27	12.58987444	11.63905556	2.74E-06	ECM_REGULATORS	1.074940058	1.0816921
**TGFBI**	12.73359622	12.17238885	4.56E-09	12.73359622	12.04849722	0.001356618	ECM_GLYCOPROTEINS	1.046104949	1.05686178
**TGM2**	13.53918311	12.01897952	3.01E-62	13.53918311	12.86103889	0.002707094	ECM_REGULATORS	1.126483583	1.052728573
**THBS1**	13.651726	12.14261519	1.69E-44	13.651726	13.06033889	0.019802709	ECM_GLYCOPROTEINS	1.12428219	1.045281146
**THBS2**	11.84119689	11.11429101	6.90E-11	11.84119689	9.501916667	4.49E-12	ECM_GLYCOPROTEINS	1.065402812	1.246190353
**TIMP1**	14.20954578	13.01940428	3.94E-44	14.20954578	12.64195	5.20E-14	ECM_REGULATORS	1.091412899	1.123999524

**TINAG**	4.209975333	1.575004602	1.56E-66	4.209975333	2.8214	0.008594082	ECM_GLYCOPROTEINS	2.672992401	1.492158267
**TINAGL1**	12.17423156	10.44053464	1.33E-69	12.17423156	11.448325	3.95E-06	ECM_GLYCOPROTEINS	1.166054419	1.063407228
**TNFAIP6**	7.438055333	6.456752307	1.44E-15	7.438055333	6.621619444	0.002266519	ECM_GLYCOPROTEINS	1.1519809	1.123298522
**TSPEAR**	4.212367556	2.483923895	6.05E-66	4.212367556	3.134252778	0.001647062	ECM_GLYCOPROTEINS	1.695852101	1.343978248
**VCAN**	12.61271044	11.6322721	1.53E-17	12.61271044	10.87264167	1.47E-11	PROTEOGLYCANS	1.084286057	1.160041031
**WISP1**	8.431896222	7.255730357	7.08E-26	8.431896222	6.486747222	4.82E-11	ECM_GLYCOPROTEINS	1.16210165	1.299865084
**WISP3**	5.613243333	4.021015402	6.87E-56	5.613243333	4.527647222	0.00216102	ECM_GLYCOPROTEINS	1.395976581	1.239770472
**WNT2**	7.018146667	5.44718666	2.64E-27	7.018146667	2.896480556	1.10E-23	SECRETED_FACTORS	1.288398416	2.42299112
**WNT5A**	10.14453956	8.979624458	9.63E-26	10.14453956	9.235061111	5.89E-05	SECRETED_FACTORS	1.12972871	1.098481042
**WNT8B**	2.700165111	1.719004804	5.38E-39	2.700165111	1.233094444	8.72E-08	SECRETED_FACTORS	1.570772289	2.189747203
**VWA1**	12.42854844	11.49001339	1.06E-41	12.42854844	12.0278	0.020532188	ECM_GLYCOPROTEINS	1.081682677	1.033318516
**VWA3B**	4.741314444	3.140936735	1.10E-46	4.741314444	4.147102778	0.03969392	ECM_GLYCOPROTEINS	1.509522428	1.143283564
**VWA9**	10.68596133	10.61265181	0.000920595	10.68596133	10.06440278	5.35E-12	ECM_GLYCOPROTEINS	1.006907748	1.061758116
**ZFP91**	12.10707911	11.61588412	7.68E-59	12.10707911	11.845475	0.001936904	SECRETED_FACTORS	1.042286492	1.022084729

Testicular germ cell tumor (TGCT)

Signature genes	Expression in tumor	Expression in rest of Pan-Cancer	P-value	Expression in tumor	Expression in normal tissue	P-value	Matrisome category	Fold change vs. Pan-Cancer	Fold change vs. normal
**A2M**	14.48974416	14.10091615	0.00758076	14.48974416	13.23631932	4.83E-45	ECM_REGULATORS	1.027574662	1.094695875
**ADAM18**	3.024779221	0.43062132	4.62E-148	3.024779221	2.958575	1.83E-05	ECM_REGULATORS	7.024220771	1.022377064
**ADAM19**	10.25900455	9.039341063	2.94E-20	10.25900455	4.969286364	2.22E-156	ECM_REGULATORS	1.134928362	2.064482462
**ADAM20**	1.945568831	1.723510404	0.014221359	1.945568831	1.8749125	2.85E-108	ECM_REGULATORS	1.128840782	1.037685135
**ADAM32**	5.908311688	4.611716375	4.31E-20	5.908311688	3.996519318	4.18E-48	ECM_REGULATORS	1.28115244	1.478364351
**ADAM33**	8.422316234	6.14895825	4.30E-32	8.422316234	2.576570455	5.79E-22	ECM_REGULATORS	1.369714331	3.268808823
**ADAM8**	9.723261039	8.866244305	2.52E-09	9.723261039	1.253972727	1.89E-19	ECM_REGULATORS	1.096660627	7.75396532
**ADAMDEC1**	9.192250649	6.074119259	2.64E-44	9.192250649	8.603455682	8.74E-125	ECM_REGULATORS	1.513347081	1.068437032
**ADAMTS3**	7.165530519	5.8464072	6.38E-18	7.165530519	2.823254545	0.000575951	ECM_REGULATORS	1.225629737	2.538039133
**ADAMTS6**	6.497740909	5.759757718	3.19E-07	6.497740909	0	1.36E-13	ECM_REGULATORS	1.128127471	Inf
**ADAMTS7**	9.56698961	8.043525123	1.62E-34	9.56698961	1.104234091	8.94E-99	ECM_REGULATORS	1.189402589	8.663914372
**ADAMTSL4**	10.14736299	8.819841914	6.84E-20	10.14736299	0.429810227	1.21E-14	ECM_REGULATORS	1.150515291	23.60893795
**ADAMTSL5**	6.944474026	6.219341179	9.28E-06	6.944474026	0.935293182	1.14E-81	ECM_REGULATORS	1.116593193	7.424916765
**AMH**	7.377821429	4.812156818	3.25E-39	7.377821429	0.357515909	2.03E-50	SECRETED_FACTORS	1.533163134	20.6363444
**ANGPT1**	8.72602987	7.111320236	2.66E-22	8.72602987	2.541519318	4.23E-12	SECRETED_FACTORS	1.227061865	3.433391125
**ANGPTL5**	1.757477922	1.363457321	0.001276508	1.757477922	0.079918182	3.05E-93	SECRETED_FACTORS	1.288986384	21.99096479
**ANXA3**	9.805933766	9.154774867	0.002751325	9.805933766	3.499820455	0.015956503	ECM_AFFILIATED	1.071127789	2.801839092
**ANXA6**	13.35774416	12.23810033	4.16E-29	13.35774416	0.067611364	2.12E-73	ECM_AFFILIATED	1.091488368	197.5665545
**BCAN**	7.665983117	4.52263364	2.84E-20	7.665983117	1.5822	6.80E-97	PROTEOGLYCANS	1.695026334	4.845141649
**BMP7**	9.124014286	8.012745776	0.00025648	9.124014286	8.190473864	8.68E-83	SECRETED_FACTORS	1.138687604	1.113978805
**BMP8A**	4.776299351	4.243046569	0.00052084	4.776299351	1.973632955	3.18E-05	SECRETED_FACTORS	1.125676863	2.420054519
**C1QA**	12.68443312	11.45343086	4.46E-18	12.68443312	9.521038636	2.60E-36	ECM_AFFILIATED	1.107478909	1.332253087
**C1QB**	12.62134675	11.61465161	1.88E-11	12.62134675	11.57160795	3.03E-13	ECM_AFFILIATED	1.086674588	1.090716761
**C1QC**	12.45674091	11.45251726	3.57E-12	12.45674091	6.343613636	3.19E-30	ECM_AFFILIATED	1.087685845	1.963666393
**C1QTNF9**	2.757572727	2.268579096	0.000224449	2.757572727	2.696727273	1.50E-06	ECM_AFFILIATED	1.21555062	1.022562702
**CCL1**	1.805602597	0.617419046	3.26E-46	1.805602597	0.614193182	1.14E-147	SECRETED_FACTORS	2.924436183	2.939795899
**CCL11**	4.696264935	4.024827146	0.00380168	4.696264935	0.786252273	2.22E-98	SECRETED_FACTORS	1.166824006	5.972974703
**CCL13**	5.433473377	4.47403663	8.39E-06	5.433473377	2.6249875	1.38E-122	SECRETED_FACTORS	1.214445438	2.069904476
**CCL14**	7.354714286	6.785299197	0.00858529	7.354714286	3.622790909	1.40E-08	SECRETED_FACTORS	1.083918936	2.030123866
**CCL17**	4.19380974	3.627146966	0.000855451	4.19380974	3.565842045	6.18E-70	SECRETED_FACTORS	1.156228237	1.176106425
**CCL18**	8.031631169	7.071676086	0.000405161	8.031631169	5.106510227	2.71E-16	SECRETED_FACTORS	1.135746472	1.57282191
**CCL19**	9.996823377	6.208006281	9.31E-49	9.996823377	9.1442125	0.011567302	SECRETED_FACTORS	1.610311415	1.093240492
**CCL21**	8.31929026	6.894106697	1.30E-06	8.31929026	5.462685227	0.000736408	SECRETED_FACTORS	1.206724907	1.522930558
**CCL24**	4.441853247	2.725102874	3.11E-17	4.441853247	0.097161364	1.98E-106	SECRETED_FACTORS	1.629976354	45.71625058
**CCL3**	8.740666883	7.417444943	2.71E-21	8.740666883	6.340792045	4.14E-108	SECRETED_FACTORS	1.178393227	1.378481871
**CCL5**	10.88066818	9.357343501	2.56E-20	10.88066818	9.316761364	4.49E-25	SECRETED_FACTORS	1.162794567	1.167859491
**CILP2**	8.859505844	6.300295248	1.49E-35	8.859505844	3.601670455	4.78E-72	ECM_GLYCOPROTEINS	1.406204867	2.459832446
**CLEC11A**	9.082371429	8.517372438	2.05E-06	9.082371429	4.235623864	1.35E-37	ECM_AFFILIATED	1.066334893	2.144281863
**CLEC12A**	6.706621429	5.078986045	6.95E-21	6.706621429	3.044089773	1.97E-05	ECM_AFFILIATED	1.32046463	2.203161513
**CLEC12B**	3.705198052	1.676101665	4.61E-52	3.705198052	1.214805682	2.53E-13	ECM_AFFILIATED	2.210604601	3.050033522
**CLEC17A**	3.622554545	2.780440172	2.03E-07	3.622554545	2.599748864	1.27E-158	ECM_AFFILIATED	1.302870884	1.393424802

**CLEC18C**	4.618565584	3.427894406	3.83E-08	4.618565584	4.195159091	1.32E-37	ECM_AFFILIATED	1.347347683	1.100927399
**CLEC1A**	7.807029221	6.223547285	6.73E-41	7.807029221	1.579334091	0.035132149	ECM_AFFILIATED	1.254433985	4.94324112
**CLEC4A**	6.925666883	6.136338653	1.35E-14	6.925666883	0.693794318	3.01E-14	ECM_AFFILIATED	1.128631791	9.982305565
**CLEC4E**	7.142075974	5.377278622	1.99E-29	7.142075974	4.371936364	2.42E-06	ECM_AFFILIATED	1.328195259	1.633618466
**CLEC9A**	6.874375325	3.469250285	2.65E-100	6.874375325	1.9509875	1.74E-84	ECM_AFFILIATED	1.981516108	3.523536324
**COCH**	8.265304545	6.447597242	4.29E-19	8.265304545	4.182827273	1.92E-114	ECM_GLYCOPROTEINS	1.281920107	1.97600905
**COL11A2**	6.844648701	5.976564638	7.76E-07	6.844648701	3.157593182	2.99E-106	COLLAGENS	1.145248001	2.167679086
**COL13A1**	7.537797403	6.362314633	1.08E-14	7.537797403	4.500606818	2.26E-05	COLLAGENS	1.184757095	1.67484024
**COL14A1**	10.38097013	9.990020255	0.030178546	10.38097013	0.121203409	2.58E-27	COLLAGENS	1.039134042	85.6491596
**COL15A1**	10.85463117	10.47485168	0.033959411	10.85463117	7.035285227	1.66E-13	COLLAGENS	1.036256312	1.542884306
**COL15A1**	10.85463117	10.47485168	0.033959411	10.85463117	7.035285227	1.66E-13	BASEMENT_MEMBRANES	1.036256312	1.542884306
**COL17A1**	10.92392403	7.826266738	6.43E-20	10.92392403	3.388855682	0.012760561	COLLAGENS	1.395802672	3.223484578
**COL1A1**	15.55602792	14.72309046	0.00078358	15.55602792	5.928756818	1.29E-135	COLLAGENS	1.056573548	2.623826276
**COL1A2**	15.54163117	14.47520346	6.84E-07	15.54163117	7.728581818	1.89E-42	COLLAGENS	1.073672726	2.010929241
**COL21A1**	7.019368831	6.14213844	1.15E-06	7.019368831	5.823370455	5.76E-111	COLLAGENS	1.142821657	1.205379065
**COL25A1**	4.638569481	3.886852269	9.66E-05	4.638569481	3.960978409	2.96E-42	COLLAGENS	1.193399995	1.17106659
**COL3A1**	14.95189286	14.25650017	0.00347231	14.95189286	4.069794318	1.91E-05	COLLAGENS	1.048777237	3.673869412
**COL4A1**	13.79657662	13.18191152	6.57E-05	13.79657662	8.029746591	2.51E-34	COLLAGENS	1.046629437	1.718183316
**COL4A1**	13.79657662	13.18191152	6.57E-05	13.79657662	8.029746591	2.51E-34	BASEMENT_MEMBRANES	1.046629437	1.718183316
**COL4A2**	14.20705195	13.44157649	2.50E-07	14.20705195	5.313918182	0.038483001	BASEMENT_MEMBRANES	1.056948339	2.673554892
**COL4A2**	14.20705195	13.44157649	2.50E-07	14.20705195	5.313918182	0.038483001	COLLAGENS	1.056948339	2.673554892
**COL4A5**	9.769585065	9.278673773	0.010480589	9.769585065	6.1588875	6.11E-11	COLLAGENS	1.052907485	1.586258081
**COL4A5**	9.769585065	9.278673773	0.010480589	9.769585065	6.1588875	6.11E-11	BASEMENT_MEMBRANES	1.052907485	1.586258081
**COL4A6**	8.706823377	6.14074324	1.57E-25	8.706823377	4.933819318	2.50E-27	BASEMENT_MEMBRANES	1.417877777	1.764722787
**COL4A6**	8.706823377	6.14074324	1.57E-25	8.706823377	4.933819318	2.50E-27	COLLAGENS	1.417877777	1.764722787
**COL5A3**	9.952461039	9.44224444	0.000677925	9.952461039	5.696418182	1.12E-07	COLLAGENS	1.054035521	1.747143682
**COL6A2**	14.32705909	13.26632556	1.55E-10	14.32705909	11.82396818	2.30E-125	COLLAGENS	1.079956845	1.21169635
**COL6A2**	14.32705909	13.26632556	1.55E-10	14.32705909	11.82396818	2.30E-125	BASEMENT_MEMBRANES	1.079956845	1.21169635
**COL6A3**	13.28428247	12.61874339	0.002642124	13.28428247	12.88514318	4.74E-74	BASEMENT_MEMBRANES	1.052742104	1.030976706
**COL6A3**	13.28428247	12.61874339	0.002642124	13.28428247	12.88514318	4.74E-74	COLLAGENS	1.052742104	1.030976706
**COMP**	8.168780519	7.219158037	0.002025102	8.168780519	8.0084125	1.24E-113	ECM_GLYCOPROTEINS	1.131541999	1.020024945
**CPAMD8**	8.765424675	7.626237433	1.81E-09	8.765424675	3.203618182	9.88E-116	ECM_REGULATORS	1.149377364	2.736101551
**CRELD2**	10.55531429	10.39180375	0.015376245	10.55531429	0.708672727	8.59E-09	ECM_GLYCOPROTEINS	1.015734567	14.89448356
**CRISPLD1**	9.315064935	8.097706832	7.13E-11	9.315064935	6.08975	0.020098427	ECM_GLYCOPROTEINS	1.150333684	1.529630106
**CRLF1**	9.099877922	7.059624756	9.68E-32	9.099877922	6.768884091	2.74E-69	SECRETED_FACTORS	1.289003061	1.344368998
**CSF2**	2.905978571	2.068880074	3.33E-06	2.905978571	0.905110227	1.73E-19	SECRETED_FACTORS	1.404614317	3.210634997
**CSH1**	3.801808442	0.184485919	0	3.801808442	0.2683125	4.55E-65	SECRETED_FACTORS	20.60758059	14.16933032
**CST7**	8.23276039	7.014861792	2.81E-14	8.23276039	5.448784091	7.55E-89	ECM_REGULATORS	1.173616906	1.51093533
**CST9L**	1.132381818	0.272094774	2.55E-29	1.132381818	0.871779545	3.02E-275	ECM_REGULATORS	4.161718365	1.298931392
**CTHRC1**	9.485818182	9.070787506	0.044383233	9.485818182	7.952111364	2.02E-98	ECM_GLYCOPROTEINS	1.045754646	1.192867875
**CTSC**	12.69650065	12.15492012	1.23E-05	12.69650065	9.98955	5.08E-117	ECM_REGULATORS	1.044556486	1.270978237
**CTSL**	12.64565844	11.86333242	9.53E-15	12.64565844	0.687885227	8.83E-34	ECM_REGULATORS	1.065944879	18.38338423
**CTSS**	11.73688571	11.41590601	0.010865798	11.73688571	0.254202273	5.16E-157	ECM_REGULATORS	1.028116884	46.17144288
**CXCL13**	8.162095455	6.319954466	1.81E-11	8.162095455	6.927760227	5.75E-05	SECRETED_FACTORS	1.291480105	1.178172337
**CXCL9**	11.64381364	8.922699903	7.07E-30	11.64381364	3.184668182	5.85E-10	SECRETED_FACTORS	1.304965287	3.656209367
**DDX26B**	8.856307143	8.163141895	3.36E-11	8.856307143	1.648797727	9.32E-53	ECM_GLYCOPROTEINS	1.084914027	5.371372726

**EBI3**	7.230278571	6.050194039	4.08E-20	7.230278571	1.269811364	5.67E-19	SECRETED_FACTORS	1.195049039	5.693978475
**EFEMP1**	11.6112474	10.44925799	1.77E-09	11.6112474	7.888103409	1.57E-101	ECM_GLYCOPROTEINS	1.111203055	1.471994826
**EGFL7**	11.32200909	9.73113783	1.59E-47	11.32200909	8.208879545	1.48E-42	SECRETED_FACTORS	1.163482553	1.379239277
**EGFL8**	8.803781818	7.641828307	2.72E-26	8.803781818	7.413967045	3.46E-22	SECRETED_FACTORS	1.152051769	1.18745899
**EGFLAM**	10.06765455	7.591718339	7.24E-68	10.06765455	6.383727273	9.38E-08	ECM_GLYCOPROTEINS	1.326136468	1.57708093
**EGLN2**	11.93895325	11.42198875	8.45E-22	11.93895325	9.702230682	1.04E-68	ECM_REGULATORS	1.045260462	1.230536939
**ELSPBP1**	0.941251299	0.46003153	1.56E-06	0.941251299	0.149809091	4.71E-69	ECM_GLYCOPROTEINS	2.046058231	6.28300521
**EMID1**	8.763759091	8.14496787	4.54E-05	8.763759091	8.202040909	1.22E-104	ECM_GLYCOPROTEINS	1.075972211	1.068485172
**F10**	7.130981169	6.183197174	5.67E-06	7.130981169	0.041297727	1.06E-56	ECM_REGULATORS	1.153283806	172.6724844
**F2**	5.467025974	2.072911129	5.60E-34	5.467025974	5.179459091	1.32E-08	ECM_REGULATORS	2.637366309	1.05552064
**FAM20A**	9.6255	8.560326662	1.24E-09	9.6255	5.639498864	2.88E-18	ECM_REGULATORS	1.124431389	1.706800592
**FBLN1**	13.75817857	11.59315023	2.79E-29	13.75817857	5.178522727	1.34E-88	ECM_GLYCOPROTEINS	1.18675065	2.656776709
**FBLN2**	11.31948182	10.23178675	1.18E-10	11.31948182	10.38573636	2.27E-42	ECM_GLYCOPROTEINS	1.106305486	1.089906524
**FBLN5**	12.06672922	9.873761879	2.16E-55	12.06672922	8.263398864	3.59E-147	ECM_GLYCOPROTEINS	1.222100489	1.460262226
**FBN3**	11.11442727	4.14700932	1.49E-140	11.11442727	5.654632955	7.67E-48	ECM_GLYCOPROTEINS	2.680106654	1.96554354
**FCN1**	8.661434416	6.368586596	7.00E-38	8.661434416	4.508817045	3.15E-27	ECM_AFFILIATED	1.360024596	1.920999306
**FGF11**	8.47735974	7.665485048	5.41E-06	8.47735974	0.404869318	2.66E-22	SECRETED_FACTORS	1.105913023	20.9385087
**FGF13**	10.00445195	7.236045505	4.79E-54	10.00445195	2.916180682	1.27E-17	SECRETED_FACTORS	1.382585549	3.43066944
**FGF16**	3.343573377	1.048529498	1.43E-74	3.343573377	2.116503409	1.39E-133	SECRETED_FACTORS	3.188821472	1.579762812
**FGF17**	6.759304545	3.066458337	4.00E-97	6.759304545	3.023728409	1.48E-12	SECRETED_FACTORS	2.204270791	2.235420524
**FGF19**	6.859064935	1.779349231	1.34E-122	6.859064935	5.228060227	8.57E-44	SECRETED_FACTORS	3.854816591	1.311971293
**FGF2**	8.417274675	7.807297242	0.000282275	8.417274675	0.318039773	8.55E-71	SECRETED_FACTORS	1.078129142	26.46610706
**FGF3**	0.865861688	0.433148021	0.000102411	0.865861688	0.704777273	1.89E-166	SECRETED_FACTORS	1.998997217	1.228560741
**FGF4**	5.919713636	0.414717188	0	5.919713636	1.228957955	6.81E-158	SECRETED_FACTORS	14.27409766	4.816856113
**FGL2**	10.14908701	9.58491772	5.58E-05	10.14908701	0.014686364	1.15E-24	ECM_GLYCOPROTEINS	1.058860108	691.0551355
**FNDC7**	2.672161039	0.754637675	3.07E-91	2.672161039	0	1.58E-39	ECM_GLYCOPROTEINS	3.540985464	Inf
**FRAS1**	9.750134416	8.380423023	1.19E-11	9.750134416	8.400604545	4.99E-144	ECM_GLYCOPROTEINS	1.163441796	1.160646756
**FREM1**	7.053040909	5.985597532	1.92E-07	7.053040909	0.013803409	8.08E-162	ECM_AFFILIATED	1.178335308	510.9636947
**HAPLN1**	6.638294805	5.239506823	1.06E-08	6.638294805	0.216446591	1.02E-24	PROTEOGLYCANS	1.266969398	30.66943571
**HPX**	6.620682468	4.962082464	6.50E-11	6.620682468	4.147871591	5.26E-25	ECM_AFFILIATED	1.334254825	1.596163797
**HTRA4**	5.40094026	3.348438982	1.25E-39	5.40094026	1.250257955	3.08E-82	ECM_REGULATORS	1.612972579	4.319860746
**HYAL2**	11.65225065	11.15445061	1.01E-14	11.65225065	1.068781818	3.21E-92	ECM_REGULATORS	1.044627929	10.90236609
**HYAL4**	8.815507792	1.19545825	0	8.815507792	3.035072727	1.91E-05	ECM_REGULATORS	7.374166175	2.904545816
**IGFBP2**	12.5405	11.54679975	1.87E-07	12.5405	3.308595455	6.41E-95	ECM_GLYCOPROTEINS	1.086058499	3.790279039
**IGSF10**	6.967480519	5.994378825	3.00E-06	6.967480519	6.493828409	3.88E-47	ECM_GLYCOPROTEINS	1.162335702	1.072938809
**ITLN2**	6.132425325	1.964228414	7.34E-103	6.132425325	2.077495455	3.82E-86	ECM_AFFILIATED	3.122053057	2.951835736
**KNG1**	5.000430519	2.176259595	2.04E-19	5.000430519	3.533275	8.59E-151	ECM_REGULATORS	2.29771785	1.41523955
**LAMA4**	11.47398247	10.91165478	5.88E-05	11.47398247	4.796089773	4.31E-32	ECM_GLYCOPROTEINS	1.051534593	2.392361905
**LAMA4**	11.47398247	10.91165478	5.88E-05	11.47398247	4.796089773	4.31E-32	BASEMENT_MEMBRANES	1.051534593	2.392361905
**LAMA5**	12.66366429	12.32682005	0.003552334	12.66366429	5.271243182	4.66E-86	ECM_GLYCOPROTEINS	1.027326126	2.402405628
**LAMA5**	12.66366429	12.32682005	0.003552334	12.66366429	5.271243182	4.66E-86	BASEMENT_MEMBRANES	1.027326126	2.402405628
**LAMB1**	12.60562078	12.22750926	0.004833951	12.60562078	6.506263636	3.64E-82	BASEMENT_MEMBRANES	1.03092302	1.937459268
**LAMB1**	12.60562078	12.22750926	0.004833951	12.60562078	6.506263636	3.64E-82	ECM_GLYCOPROTEINS	1.03092302	1.937459268
**LAMC1**	13.81678506	12.78352298	6.02E-22	13.81678506	1.8319125	1.12E-102	ECM_GLYCOPROTEINS	1.080827647	7.542273479
**LAMC1**	13.81678506	12.78352298	6.02E-22	13.81678506	1.8319125	1.12E-102	BASEMENT_MEMBRANES	1.080827647	7.542273479
**LAMC3**	10.27522338	7.751949405	1.30E-51	10.27522338	5.444	1.33E-26	BASEMENT_MEMBRANES	1.325501863	1.887440003

**LAMC3**	10.27522338	7.751949405	1.30E-51	10.27522338	5.444	1.33E-26	ECM_GLYCOPROTEINS	1.325501863	1.887440003
**LGALS12**	4.050072078	3.118714613	9.41E-07	4.050072078	0.125365909	1.86E-66	ECM_AFFILIATED	1.29863504	32.30600813
**LGALS16**	1.215059091	0.026903571	0	1.215059091	0.795284091	1.94E-118	ECM_AFFILIATED	45.1634873	1.527830249
**LGALS7**	3.47269026	2.673128898	0.002431626	3.47269026	1.140015909	2.69E-64	ECM_AFFILIATED	1.299110665	3.046177016
**LOXL1**	9.994736364	9.223631424	1.37E-06	9.994736364	9.193948864	2.71E-171	ECM_REGULATORS	1.083601014	1.087099408
**LOXL2**	12.41042857	10.13705281	2.10E-58	12.41042857	1.558902273	8.71E-180	ECM_REGULATORS	1.224263975	7.961004861
**LTBP4**	12.09105779	11.5508213	1.07E-05	12.09105779	1.134201136	1.22E-55	ECM_GLYCOPROTEINS	1.046770396	10.66041763
**MATN3**	7.07896039	6.479670986	0.000897942	7.07896039	6.243238636	1.37E-126	ECM_GLYCOPROTEINS	1.092487629	1.133860293
**MFAP1**	10.82840519	10.3685813	2.44E-29	10.82840519	2.215597727	5.97E-122	ECM_GLYCOPROTEINS	1.044347812	4.887351644
**MFAP2**	11.00239416	9.09298169	3.80E-18	11.00239416	1.199613636	1.33E-68	ECM_GLYCOPROTEINS	1.209987497	9.171614779
**MFAP5**	7.930794805	6.773161405	1.93E-05	7.930794805	7.282652273	4.38E-67	ECM_GLYCOPROTEINS	1.170914781	1.088998144
**MMP15**	13.1271013	10.75676714	1.66E-83	13.1271013	4.612923864	1.26E-113	ECM_REGULATORS	1.220357485	2.845722515
**MMP16**	7.66576039	6.831207403	7.81E-05	7.66576039	0.242132955	5.48E-79	ECM_REGULATORS	1.122167713	31.65930224
**MMP2**	13.48239091	12.25474887	5.92E-14	13.48239091	11.88949318	6.34E-175	ECM_REGULATORS	1.100176842	1.133975242
**MMP23B**	6.814503247	5.812182203	4.65E-12	6.814503247	2.105089773	1.22E-22	ECM_REGULATORS	1.172451759	3.237155648
**MMP24**	10.41815325	6.904890448	3.41E-72	10.41815325	0.241529545	7.32E-128	ECM_REGULATORS	1.508807899	43.13407383
**MMP9**	12.71395909	9.321476019	8.67E-50	12.71395909	10.95604773	5.54E-11	ECM_REGULATORS	1.363942692	1.160451233
**MUC12**	5.687809091	4.995281138	0.005228636	5.687809091	3.365229545	2.32E-94	ECM_AFFILIATED	1.138636432	1.690169724
**MUC16**	6.727663636	5.291437598	3.29E-05	6.727663636	3.793965909	4.68E-37	ECM_AFFILIATED	1.271424544	1.773253581
**MUC3A**	8.727307792	6.431331046	8.43E-16	8.727307792	0.487304545	1.73E-78	ECM_AFFILIATED	1.356998688	17.90935027
**MUC4**	8.680755195	6.699345553	2.24E-10	8.680755195	6.610771591	8.08E-25	ECM_AFFILIATED	1.295761672	1.313122844
**NCAN**	6.512582468	2.406321746	3.62E-47	6.512582468	5.494847727	1.96E-168	PROTEOGLYCANS	2.706447082	1.185216186
**NELL1**	5.282794805	3.299447847	5.43E-14	5.282794805	0.758869318	7.33E-38	ECM_GLYCOPROTEINS	1.601114808	6.961402548
**NGLY1**	10.1256513	9.970886625	0.001045832	10.1256513	7.918786364	2.20E-37	ECM_REGULATORS	1.015521656	1.278687268
**NID2**	9.877287013	9.197347518	1.53E-05	9.877287013	7.775701136	2.82E-51	BASEMENT_MEMBRANES	1.073927781	1.270276061
**NID2**	9.877287013	9.197347518	1.53E-05	9.877287013	7.775701136	2.82E-51	ECM_GLYCOPROTEINS	1.073927781	1.270276061
**NTNG1**	7.672241558	4.252056566	4.56E-45	7.672241558	2.505509091	1.09E-118	ECM_GLYCOPROTEINS	1.804360182	3.062148761
**NTNG1**	7.672241558	4.252056566	4.56E-45	7.672241558	2.505509091	1.09E-118	BASEMENT_MEMBRANES	1.804360182	3.062148761
**OGFOD1**	10.80253701	10.46312039	3.58E-17	10.80253701	2.750610227	6.56E-44	ECM_REGULATORS	1.032439331	3.927323801
**OIT3**	4.305724675	3.287965518	8.75E-08	4.305724675	0.97745	2.02E-27	ECM_GLYCOPROTEINS	1.309540703	4.40505875
**PCOLCE**	11.30457013	10.57789727	5.37E-07	11.30457013	0.029690909	7.99E-06	ECM_GLYCOPROTEINS	1.068697288	380.7417986
**PCOLCE2**	8.764474675	6.526141798	1.45E-25	8.764474675	2.041115909	9.88E-67	ECM_GLYCOPROTEINS	1.342979504	4.293962257
**PCSK5**	10.40851364	8.323331966	1.02E-50	10.40851364	4.949498864	4.07E-87	ECM_REGULATORS	1.250522469	2.102942929
**PLOD1**	13.32065325	12.5094691	6.61E-25	13.32065325	11.82298409	5.28E-83	ECM_REGULATORS	1.064845609	1.126674378
**PLXNA1**	11.9026974	11.51487167	0.000179702	11.9026974	5.021021591	7.01E-60	ECM_AFFILIATED	1.033680421	2.370572838
**PLXNA2**	10.8827974	10.52951998	0.000793112	10.8827974	3.247742045	6.37E-19	ECM_AFFILIATED	1.033551142	3.350881089
**POMZP3**	9.193207143	8.767994484	2.07E-08	9.193207143	1.451835227	1.46E-153	ECM_GLYCOPROTEINS	1.048496	6.332128447
**PXDN**	12.77679805	11.24386177	1.85E-25	12.77679805	0.502647727	4.36E-133	ECM_GLYCOPROTEINS	1.13633539	25.41899099
**PXDNL**	6.034985714	4.509557428	2.09E-20	6.034985714	2.552178409	3.09E-26	ECM_GLYCOPROTEINS	1.338265631	2.364641003
**RELN**	7.042130519	5.782373909	1.90E-07	7.042130519	0.370898864	1.57E-11	ECM_GLYCOPROTEINS	1.217861493	18.98665973
**RSPO1**	4.11025974	3.350703639	0.000678619	4.11025974	1.812540909	1.57E-56	ECM_GLYCOPROTEINS	1.226685551	2.267678329
**RSPO2**	3.894609091	2.661749598	2.49E-08	3.894609091	2.923955682	0.006760204	ECM_GLYCOPROTEINS	1.463176361	1.331965842
**RSPO3**	7.631338961	5.728923168	1.33E-20	7.631338961	7.129190909	0.032197485	ECM_GLYCOPROTEINS	1.332072143	1.07043549
**RSPO4**	6.305003247	4.166017265	6.69E-22	6.305003247	1.403763636	1.43E-68	ECM_GLYCOPROTEINS	1.513436658	4.491499198
**SDC2**	11.69209156	11.10406254	0.000314522	11.69209156	9.546525	5.15E-18	ECM_AFFILIATED	1.052956206	1.224748436
**SEMA4A**	10.55910909	9.711148921	1.39E-10	10.55910909	5.029613636	4.93E-48	ECM_AFFILIATED	1.087318213	2.099387717

**SEMA4C**	12.31272922	10.75931697	5.13E-97	12.31272922	7.172706818	0.014044704	ECM_AFFILIATED	1.144378333	1.716608462
**SEMA4D**	11.41986688	10.42381143	1.10E-27	11.41986688	1.698551136	4.82E-102	ECM_AFFILIATED	1.095555782	6.723298839
**SEMA4F**	8.99652987	8.665903223	6.87E-05	8.99652987	8.810028409	1.23E-161	ECM_AFFILIATED	1.038152589	1.021169224
**SEMA4G**	8.994888961	8.492883635	0.001383029	8.994888961	3.130627273	9.88E-135	ECM_AFFILIATED	1.059108937	2.873190635
**SEMA5B**	7.800862987	6.877054166	1.78E-06	7.800862987	1.245409091	1.38E-54	ECM_AFFILIATED	1.134332055	6.263695234
**SEMA7A**	10.02117143	8.460495403	5.07E-31	10.02117143	7.163461364	3.47E-58	ECM_AFFILIATED	1.18446627	1.398928663
**SERPINA5**	9.115425974	6.423066418	1.01E-17	9.115425974	0.026001136	3.74E-13	ECM_REGULATORS	1.419170437	350.5779842
**SERPINB9**	11.366	9.237187564	1.99E-88	11.366	6.230551136	3.88E-06	ECM_REGULATORS	1.230461103	1.824236693
**SERPINE2**	11.56234351	10.72869835	1.05E-05	11.56234351	8.017265909	6.50E-108	ECM_REGULATORS	1.077702358	1.442180369
**SERPINH1**	14.13176494	12.67646775	9.69E-39	14.13176494	5.440107955	1.07E-122	ECM_REGULATORS	1.114803052	2.597699357
**SLIT1**	7.076403896	5.41022524	4.80E-12	7.076403896	1.756978409	4.17E-31	ECM_GLYCOPROTEINS	1.307968445	4.027598666
**SMOC1**	8.250266234	6.990809262	4.70E-06	8.250266234	0.795704545	1.16E-14	ECM_GLYCOPROTEINS	1.180158967	10.36850459
**SPAM1**	2.753737662	0.152275283	0	2.753737662	2.439968182	9.54E-57	ECM_REGULATORS	18.08394381	1.128595726
**SPOCK2**	11.09782597	10.66480901	0.012711783	11.09782597	0.104305682	1.37E-45	PROTEOGLYCANS	1.040602412	106.3971375
**SVEP1**	9.567695455	8.193189306	1.06E-15	9.567695455	7.204344318	1.11E-37	ECM_GLYCOPROTEINS	1.16776204	1.328045278
**TECTB**	1.718621429	0.884186867	2.42E-12	1.718621429	1.395998864	2.53E-93	ECM_GLYCOPROTEINS	1.943731005	1.231105177
**THBS4**	9.164805844	7.914888977	3.80E-08	9.164805844	4.319764773	3.31E-09	ECM_GLYCOPROTEINS	1.157919697	2.121598357
**TLL2**	7.246832468	4.877072312	3.08E-59	7.246832468	7.080390909	1.82E-06	ECM_REGULATORS	1.485898097	1.023507397
**TSPEAR**	5.686031818	2.603217952	5.58E-72	5.686031818	0.246639773	2.77E-34	ECM_GLYCOPROTEINS	2.184231948	23.05399391
**WFIKKN2**	4.037857792	2.365066883	3.99E-24	4.037857792	1.607265909	1.18E-171	SECRETED_FACTORS	1.707291164	2.512252496
**WNT1**	1.516121429	0.68634775	4.57E-26	1.516121429	1.228228409	1.45E-170	SECRETED_FACTORS	2.208969766	1.234396972
**WNT11**	6.864704545	5.99647208	9.52E-06	6.864704545	2.8379625	1.45E-36	SECRETED_FACTORS	1.144790546	2.418884867
**WNT2B**	10.22443766	7.538313742	2.02E-87	10.22443766	9.662820455	8.13E-63	SECRETED_FACTORS	1.356329547	1.058121457
**WNT3**	10.65651558	6.938683499	7.24E-166	10.65651558	0.864421591	1.37E-07	SECRETED_FACTORS	1.535812317	12.32791464
**WNT6**	5.314022078	3.820398635	8.63E-18	5.314022078	3.036595455	6.91E-43	SECRETED_FACTORS	1.390960103	1.749993424
**WNT8A**	4.022231818	0.2552979	0	4.022231818	1.177968182	9.25E-64	SECRETED_FACTORS	15.75505251	3.414550478
**WNT9A**	8.621424675	6.912866351	1.86E-22	8.621424675	1.376176136	2.30E-51	SECRETED_FACTORS	1.247156279	6.26476833
**VTN**	7.441898052	5.264000174	4.70E-14	7.441898052	5.340589773	3.58E-06	ECM_GLYCOPROTEINS	1.413734386	1.393459968
**VWA5B2**	6.378844805	4.469438401	6.34E-21	6.378844805	3.707904545	1.86E-12	ECM_GLYCOPROTEINS	1.427213943	1.720336844
**VWA9**	10.71350325	10.62326302	0.015060945	10.71350325	0	4.24E-23	ECM_GLYCOPROTEINS	1.008494586	Inf
**VWDE**	5.568891558	4.248050131	9.51E-09	5.568891558	0.554190909	2.81E-22	ECM_GLYCOPROTEINS	1.31092887	10.04868804
**VWF**	12.77471104	12.19835603	3.98E-06	12.77471104	0.1099125	1.99E-107	ECM_GLYCOPROTEINS	1.04724858	116.2261894
**XCL2**	5.398020779	3.46297297	5.37E-38	5.398020779	0.949342045	5.81E-29	SECRETED_FACTORS	1.558782245	5.686065212
**ZP4**	0.952246104	0.354173628	1.24E-12	0.952246104	0.320561364	2.82E-50	ECM_GLYCOPROTEINS	2.688642034	2.97055794

Thyroid Carcinoma (THCA)

Signature genes	Expression in tumor	Expression in rest of Pan-Cancer	P-value	Expression in tumor	Expression in normal tissue	P-value	Matrisome category	Fold change vs. Pan-Cancer	Fold change vs. normal
ADAMTS10	7.94207373	7.647687142	7.26E-06	7.94207373	7.292179661	2.03E-08	ECM_REGULATORS	1.038493545	1.089122059
ADAMTS18	4.627740806	3.805770956	1.58E-16	4.627740806	2.993918644	4.00E-12	ECM_REGULATORS	1.215979852	1.545713613
ADAMTS9	10.58527356	8.920219221	9.30E-104	10.58527356	8.627013559	1.50E-21	ECM_REGULATORS	1.186660697	1.226991645
ADAMTSL2	9.23889282	8.130247237	6.65E-51	9.23889282	8.764137288	0.000348856	ECM_REGULATORS	1.136360624	1.054170253
AGRN	13.90995289	13.26102169	3.25E-31	13.90995289	13.01676949	7.37E-25	BASEMENT_MEMBRANES	1.048935234	1.068617901
AGRN	13.90995289	13.26102169	3.25E-31	13.90995289	13.01676949	7.37E-25	ECM_GLYCOPROTEINS	1.048935234	1.068617901
ANGPT2	8.726709632	8.569085831	0.02065568	8.726709632	8.275672881	0.000635881	SECRETED_FACTORS	1.018394471	1.05450152
ANGPTL4	10.23180981	9.264555022	6.60E-21	10.23180981	9.310684746	3.88E-07	SECRETED_FACTORS	1.104403804	1.098932043
ANXA1	14.84928196	12.46585992	1.44E-163	14.84928196	13.61248475	9.41E-12	ECM_AFFILIATED	1.191195959	1.090857565
ANXA5	13.76640333	13.36005709	5.50E-20	13.76640333	13.47913051	3.21E-07	ECM_AFFILIATED	1.030415008	1.021312415
BMP1	11.40903748	10.5945713	1.23E-66	11.40903748	10.22187797	6.12E-31	ECM_REGULATORS	1.076875803	1.116139081
BRINP2	4.537763398	3.054232069	1.32E-26	4.537763398	2.061559322	1.32E-13	SECRETED_FACTORS	1.485729733	2.201131614
BTC	8.721982312	6.33524282	7.48E-127	8.721982312	8.51199322	0.010564049	SECRETED_FACTORS	1.376740018	1.024669791
C17orf58	8.887916462	8.754446864	0.000529672	8.887916462	8.638189831	5.18E-07	ECM_REGULATORS	1.01524592	1.028909602
C1QL2	3.474726445	1.509790369	4.34E-87	3.474726445	2.686062712	0.029043844	ECM_AFFILIATED	2.301462849	1.293613299
C1QL4	6.383875131	3.428420754	1.46E-137	6.383875131	5.589967797	0.000437004	ECM_AFFILIATED	1.862045411	1.142023597
CBLN1	7.631945009	3.791499889	1.13E-251	7.631945009	5.57979322	4.55E-10	SECRETED_FACTORS	2.0129092	1.367782767
CBLN4	5.488711909	3.231295835	1.20E-93	5.488711909	4.010559322	4.25E-08	SECRETED_FACTORS	1.698610152	1.368565197
CCL13	5.636687916	4.421987354	1.51E-26	5.636687916	3.876461017	6.47E-08	SECRETED_FACTORS	1.274695621	1.454080898
CCL17	4.868334501	3.564475323	6.70E-48	4.868334501	3.157930508	1.38E-06	SECRETED_FACTORS	1.365792735	1.541621796
CCL18	8.113516112	7.02659162	3.98E-14	8.113516112	7.059054237	0.004483402	SECRETED_FACTORS	1.154687301	1.149377217
CILP2	7.857487741	6.250372045	2.51E-49	7.857487741	7.453645763	0.005172667	ECM_GLYCOPROTEINS	1.257123205	1.054180463
CLCF1	8.822992995	7.649952713	1.96E-53	8.822992995	8.445545763	0.011664945	SECRETED_FACTORS	1.153339547	1.04469187
CLEC14A	10.28760648	8.945370674	1.02E-103	10.28760648	9.479788136	2.11E-13	ECM_AFFILIATED	1.150048092	1.08521481
CLEC5A	6.740581436	6.193457221	5.91E-09	6.740581436	5.995201695	0.000626978	ECM_AFFILIATED	1.088339064	1.124329385
COL13A1	8.257457793	6.271441055	1.40E-137	8.257457793	5.498889831	2.76E-33	COLLAGENS	1.316676298	1.501659071
COL15A1	11.12306182	10.44342349	7.71E-13	11.12306182	10.46128136	3.62E-07	BASEMENT_MEMBRANES	1.065078117	1.063259982
COL15A1	11.12306182	10.44342349	7.71E-13	11.12306182	10.46128136	3.62E-07	COLLAGENS	1.065078117	1.063259982
COL19A1	3.904593695	2.498159056	1.93E-46	3.904593695	2.236208475	2.44E-07	COLLAGENS	1.562988428	1.746077676
COL21A1	6.928415412	6.110485488	1.08E-17	6.928415412	6.423055932	0.002882797	COLLAGENS	1.133856782	1.078678978
COL8A1	13.05528459	9.603310458	1.50E-208	13.05528459	11.85196271	5.17E-17	COLLAGENS	1.359456684	1.101529334
COL8A2	11.2203718	8.781896622	9.95E-166	11.2203718	10.21841864	1.31E-05	COLLAGENS	1.277670677	1.098053642
COMP	8.388894571	7.166548346	6.26E-14	8.388894571	5.376869492	8.30E-13	ECM_GLYCOPROTEINS	1.170562754	1.560181921
CRLF1	9.517294571	6.949789088	8.60E-176	9.517294571	7.395266102	1.14E-08	SECRETED_FACTORS	1.369436461	1.286944167
CSF2	3.803716462	1.981982291	1.53E-82	3.803716462	0.647801695	8.10E-23	SECRETED_FACTORS	1.919147552	5.871729716
CST2	4.164488792	3.573344635	1.21E-06	4.164488792	0.932377966	1.13E-14	ECM_REGULATORS	1.16543161	4.466524246
CST5	3.207081611	1.559503187	5.18E-65	3.207081611	0.418108475	1.07E-16	ECM_REGULATORS	2.056476472	7.670453498
CST6	10.27843923	4.678879942	0	10.27843923	8.280054237	1.16E-07	ECM_REGULATORS	2.19677345	1.241349264
CTSB	18.1948993	15.06274782	0	18.1948993	17.32132712	6.42E-14	ECM_REGULATORS	1.207940245	1.050433329
CTSC	12.69760438	12.13208134	6.55E-18	12.69760438	11.24594746	3.29E-15	ECM_REGULATORS	1.046613851	1.12908267
CTSH	13.09107671	12.08135141	5.22E-63	13.09107671	11.19838644	6.78E-21	ECM_REGULATORS	1.083577181	1.169014552

**CTSS**	11.77595587	11.40015797	1.82E-08	11.77595587	10.69974068	1.75E-11	ECM_REGULATORS	1.032964271	1.100583296
**CX3CL1**	11.68674781	10.6105555	4.48E-33	11.68674781	10.5807661	1.33E-21	SECRETED_FACTORS	1.101426576	1.104527564
**CXCL14**	10.45124326	9.898353691	0.00046876	10.45124326	9.258169492	0.013666581	SECRETED_FACTORS	1.05585672	1.128867134
**DHH**	3.998536778	3.105733017	3.00E-43	3.998536778	3.636674576	0.009547067	SECRETED_FACTORS	1.287469578	1.099503597
**DMP1**	1.797028021	1.402916095	3.84E-09	1.797028021	0.583476271	6.37E-08	ECM_GLYCOPROTEINS	1.280923376	3.079864786
**EBI3**	6.46606007	6.044574183	6.33E-10	6.46606007	4.865845763	3.53E-11	SECRETED_FACTORS	1.069729624	1.328866632
**ECM1**	10.22317023	10.03369264	0.026251335	10.22317023	7.827081356	6.52E-14	ECM_GLYCOPROTEINS	1.018884133	1.306128014
**EGFL7**	10.61128424	9.705162697	1.15E-54	10.61128424	10.0956	1.72E-05	SECRETED_FACTORS	1.0933649	1.051080098
**EMILIN2**	10.41825201	9.268787848	4.33E-71	10.41825201	9.040023729	1.39E-18	ECM_GLYCOPROTEINS	1.124014508	1.152458481
**EMILIN3**	5.347065499	4.925947338	0.000128167	5.347065499	3.923171186	1.19E-08	ECM_GLYCOPROTEINS	1.085489781	1.362944731
**ESM1**	8.377387916	7.662091529	4.33E-11	8.377387916	6.454432203	7.73E-16	PROTEOGLYCANS	1.093355239	1.297927943
**FAM20A**	10.70894291	8.45314401	1.29E-133	10.70894291	8.812972881	6.00E-12	ECM_REGULATORS	1.266859159	1.215133991
**FAM20C**	10.81234483	10.67142596	0.015464432	10.81234483	10.06893729	1.05E-08	ECM_REGULATORS	1.013205253	1.073831778
**FBN3**	9.126610158	3.96847264	2.07E-280	9.126610158	8.056616949	5.14E-06	ECM_GLYCOPROTEINS	2.29977903	1.132809244
**FCN3**	6.738771979	5.649724334	1.27E-30	6.738771979	5.534820339	7.55E-13	ECM_AFFILIATED	1.1927612	1.217523165
**FGF1**	8.654111909	7.038787152	3.11E-49	8.654111909	7.4468	7.34E-19	SECRETED_FACTORS	1.229489076	1.162124927
**FGF2**	8.440933975	7.78028332	1.12E-13	8.440933975	7.847550847	8.61E-06	SECRETED_FACTORS	1.084913444	1.075613798
**FGF20**	3.889862522	1.770485085	3.21E-152	3.889862522	2.954781356	8.79E-06	SECRETED_FACTORS	2.197060317	1.316463742
**FN1**	17.00291454	15.05863349	2.69E-83	17.00291454	12.95730339	6.45E-23	ECM_GLYCOPROTEINS	1.129114042	1.312226319
**GAS6**	13.11575306	11.65785705	3.43E-125	13.11575306	12.61871695	9.76E-09	ECM_GLYCOPROTEINS	1.125056947	1.0393888
**GLDN**	6.474608932	6.212625777	0.005760986	6.474608932	4.721613559	1.30E-06	ECM_GLYCOPROTEINS	1.042169473	1.371270404
**GPC4**	10.24726918	9.918893596	0.000512691	10.24726918	9.987601695	0.0026541	ECM_AFFILIATED	1.033106069	1.025998983
**HSPG2**	13.69084273	13.35272191	1.07E-07	13.69084273	13.26746102	1.06E-05	PROTEOGLYCANS	1.025322239	1.031911284
**HSPG2**	13.69084273	13.35272191	1.07E-07	13.69084273	13.26746102	1.06E-05	BASEMENT_MEMBRANES	1.025322239	1.031911284
**HYAL2**	11.86111734	11.12148919	2.31E-106	11.86111734	11.16034068	1.90E-19	ECM_REGULATORS	1.066504416	1.062791691
**IGFALS**	6.073010858	4.351310266	3.48E-58	6.073010858	5.670486441	0.048261151	ECM_GLYCOPROTEINS	1.395674058	1.070985871
**IGFBP4**	14.48250438	13.39599055	7.08E-47	14.48250438	14.17847966	0.001279866	ECM_GLYCOPROTEINS	1.08110739	1.021442688
**IGFBP6**	11.40438722	8.669294353	3.34E-186	11.40438722	9.949230508	3.08E-07	ECM_GLYCOPROTEINS	1.315491983	1.146258216
**KCP**	7.204837478	5.899193556	9.26E-39	7.204837478	5.181405085	1.76E-18	ECM_GLYCOPROTEINS	1.221325832	1.390518085
**LAMA5**	12.9925373	12.29371693	2.57E-30	12.9925373	12.53715593	4.16E-09	BASEMENT_MEMBRANES	1.056843701	1.036322542
**LAMA5**	12.9925373	12.29371693	2.57E-30	12.9925373	12.53715593	4.16E-09	ECM_GLYCOPROTEINS	1.056843701	1.036322542
**LAMB3**	11.15101961	10.80378306	0.004821096	11.15101961	7.82520678	6.20E-21	ECM_GLYCOPROTEINS	1.032140275	1.425012773
**LAMB3**	11.15101961	10.80378306	0.004821096	11.15101961	7.82520678	6.20E-21	BASEMENT_MEMBRANES	1.032140275	1.425012773
**LAMC1**	13.32744431	12.76824901	8.28E-23	13.32744431	12.8864661	3.26E-10	ECM_GLYCOPROTEINS	1.04379577	1.034220259
**LAMC1**	13.32744431	12.76824901	8.28E-23	13.32744431	12.8864661	3.26E-10	BASEMENT_MEMBRANES	1.04379577	1.034220259
**LAMC2**	12.66008511	9.975507463	3.11E-58	12.66008511	11.73135085	1.10E-14	BASEMENT_MEMBRANES	1.269116901	1.079166865
**LAMC2**	12.66008511	9.975507463	3.11E-58	12.66008511	11.73135085	1.10E-14	ECM_GLYCOPROTEINS	1.269116901	1.079166865
**LGALS13**	0.862830473	0.057909036	1.03E-243	0.862830473	0.226916949	0.004495667	ECM_AFFILIATED	14.89975544	3.802406458
**LGALS3**	12.93674974	12.37384743	2.09E-14	12.93674974	11.01799831	9.44E-18	ECM_AFFILIATED	1.045491292	1.174147007
**LTBP3**	13.75566252	12.08049001	9.39E-234	13.75566252	13.4429322	0.001531067	ECM_GLYCOPROTEINS	1.138667597	1.023263549
**MFGE8**	12.71976305	11.72169367	1.07E-56	12.71976305	11.0242	1.94E-23	ECM_GLYCOPROTEINS	1.085147199	1.153803727
**MMP16**	8.287093695	6.760333068	1.20E-42	8.287093695	6.325005085	1.70E-13	ECM_REGULATORS	1.225841037	1.310211389
**MMRN2**	10.57418144	9.674913271	1.09E-47	10.57418144	10.13646102	4.98E-06	ECM_GLYCOPROTEINS	1.092948447	1.043182766
**MUC20**	9.319869002	9.005013927	0.005395907	9.319869002	8.561118644	2.07E-06	ECM_AFFILIATED	1.034964418	1.088627478
**MUC21**	6.660997198	2.328207352	1.52E-175	6.660997198	1.749169492	6.14E-22	ECM_AFFILIATED	2.860998267	3.808091343
**MXRA5**	12.12006077	10.97968525	1.50E-25	12.12006077	11.6121322	0.000403392	ECM_GLYCOPROTEINS	1.103862315	1.043741197

**NELL2**	11.48074711	7.631686729	1.74E-252	11.48074711	10.05358814	2.91E-06	ECM_GLYCOPROTEINS	1.504352513	1.141955186
**OIT3**	5.302952014	3.187741398	1.23E-99	5.302952014	2.403528814	2.44E-31	ECM_GLYCOPROTEINS	1.663545235	2.206319302
**P4HA2**	11.36333363	10.66534336	7.63E-30	11.36333363	9.724889831	2.89E-39	ECM_REGULATORS	1.065444706	1.16847942
**P4HTM**	11.48474991	10.2400072	9.70E-113	11.48474991	11.22337966	3.53E-06	ECM_REGULATORS	1.12155682	1.023288017
**PAPLN**	11.21737338	9.088080436	7.59E-185	11.21737338	10.51503051	3.07E-06	ECM_GLYCOPROTEINS	1.234295125	1.066794183
**PAPLN**	11.21737338	9.088080436	7.59E-185	11.21737338	10.51503051	3.07E-06	BASEMENT_MEMBRANES	1.234295125	1.066794183
**PAPPA**	7.111150263	6.717022509	3.77E-05	7.111150263	6.617530508	0.015921445	ECM_REGULATORS	1.058675961	1.074592743
**PCSK5**	9.363401401	8.295824738	2.17E-47	9.363401401	9.068545763	0.03129846	ECM_REGULATORS	1.12868843	1.032514104
**PLAU**	11.38469037	10.54549264	2.46E-20	11.38469037	9.066301695	3.96E-18	ECM_REGULATORS	1.079578808	1.255714927
**PLOD2**	12.9541697	11.05678794	2.71E-136	12.9541697	12.77863898	0.011054703	ECM_REGULATORS	1.171603342	1.013736261
**PLXNA2**	11.29700946	10.49081166	1.02E-47	11.29700946	11.0224339	0.00210332	ECM_AFFILIATED	1.076847991	1.024910611
**PLXNA3**	11.01710315	10.72102504	3.19E-10	11.01710315	10.18190508	2.37E-19	ECM_AFFILIATED	1.027616586	1.082027682
**PLXNA4**	8.834649037	6.83596427	1.47E-75	8.834649037	7.467186441	3.27E-10	ECM_AFFILIATED	1.292377884	1.183129564
**PLXNB1**	12.56708564	11.82785667	1.82E-45	12.56708564	12.18867627	5.14E-07	ECM_AFFILIATED	1.06249898	1.031045977
**PLXNB2**	14.42938441	13.64021125	3.62E-100	14.42938441	13.77036949	1.13E-14	ECM_AFFILIATED	1.057856374	1.047857461
**PLXNC1**	10.6593613	9.605555587	7.80E-52	10.6593613	8.853322034	1.55E-16	ECM_AFFILIATED	1.109707939	1.203995659
**PLXND1**	12.41944623	11.83579492	1.49E-32	12.41944623	11.11547119	2.86E-35	ECM_AFFILIATED	1.049312389	1.11731172
**PRSS1**	4.343289142	1.994286507	1.05E-67	4.343289142	0.812313559	3.85E-17	ECM_REGULATORS	2.177866183	5.346813545
**PRSS2**	5.757888091	3.079281333	3.46E-66	5.757888091	1.628315254	1.69E-18	ECM_REGULATORS	1.869880491	3.536101548
**RSPO4**	8.08866725	3.97335598	2.49E-282	8.08866725	6.570837288	1.82E-07	ECM_GLYCOPROTEINS	2.035726799	1.230994909
**SDC4**	14.36031331	12.76898026	8.59E-105	14.36031331	12.49631186	7.14E-23	ECM_AFFILIATED	1.124624912	1.149164127
**SEMA3E**	6.557032925	5.122243505	1.96E-24	6.557032925	5.888342373	0.016006297	ECM_AFFILIATED	1.280109569	1.113561765
**SEMA3F**	11.46077268	10.57641549	9.89E-30	11.46077268	10.2562322	1.11E-29	ECM_AFFILIATED	1.083615965	1.117444735
**SERPINA1**	14.47703713	11.7294482	2.14E-87	14.47703713	11.0922322	8.99E-21	ECM_REGULATORS	1.234247074	1.305150926
**SERPINA2**	0.17968704	0.137456101	0.031459902	0.17968704	0	0.000317357	ECM_REGULATORS	1.30723219	Inf
**SERPINB8**	8.889187215	8.776352047	0.03048604	8.889187215	8.597933898	0.004159338	ECM_REGULATORS	1.012856728	1.033874803
**SFTA2**	4.751644133	3.044728953	1.83E-28	4.751644133	3.344011864	1.55E-08	ECM_AFFILIATED	1.56061318	1.420941171
**SFTA3**	12.81639124	1.586582745	0	12.81639124	12.27791017	8.65E-07	ECM_AFFILIATED	8.077984765	1.043857714
**SFTPA1**	4.847377233	2.429937404	9.88E-36	4.847377233	1.790011864	2.82E-09	ECM_AFFILIATED	1.994856832	2.708014025
**SFTPB**	12.27154378	3.639111718	0	12.27154378	9.326594915	1.44E-08	ECM_AFFILIATED	3.372126148	1.315758205
**SLIT1**	9.332044483	5.210268879	1.22E-239	9.332044483	4.686608475	1.46E-28	ECM_GLYCOPROTEINS	1.791086929	1.99121487
**SLPI**	9.963256567	9.619575161	0.044624172	9.963256567	8.101537288	6.85E-08	ECM_REGULATORS	1.035727296	1.229798273
**SPOCK1**	9.647773555	9.335348175	0.009055464	9.647773555	8.057715254	2.32E-09	PROTEOGLYCANS	1.033466923	1.197333642
**SPOCK2**	12.30760893	10.57693546	4.97E-80	12.30760893	10.64100847	3.68E-10	PROTEOGLYCANS	1.163627119	1.156620537
**ST14**	12.98721629	11.64842446	9.89E-34	12.98721629	12.35942034	6.86E-14	ECM_REGULATORS	1.114933297	1.050794935
**TGM1**	6.916828722	6.286314502	1.48E-07	6.916828722	5.839150847	3.15E-07	ECM_REGULATORS	1.100299503	1.184560718
**TIMP1**	13.64643275	13.03192651	2.51E-16	13.64643275	11.38380508	4.25E-19	ECM_REGULATORS	1.047153906	1.198758469
**TINAGL1**	11.81558949	10.54044506	1.42E-48	11.81558949	11.49211695	0.000516717	ECM_GLYCOPROTEINS	1.120976337	1.028147342
**WNT10A**	6.524623818	5.937454296	7.88E-07	6.524623818	4.509754237	1.75E-07	SECRETED_FACTORS	1.09889247	1.446780351
**WNT3**	7.802292995	6.946693294	7.66E-32	7.802292995	6.606927119	4.95E-14	SECRETED_FACTORS	1.123166471	1.180926148
**WNT5A**	9.616122417	9.052331202	9.49E-09	9.616122417	8.976315254	1.60E-05	SECRETED_FACTORS	1.062281329	1.071277261
**WNT8A**	0.486871103	0.300465258	5.60E-08	0.486871103	0.270888136	0.010431094	SECRETED_FACTORS	1.620390678	1.797314239
**WNT9B**	2.561282312	1.97402734	5.59E-16	2.561282312	2.046164407	0.002450592	SECRETED_FACTORS	1.2974908	1.251748053
**VWA1**	11.64718249	11.52490643	0.045242187	11.64718249	10.55708305	2.53E-19	ECM_GLYCOPROTEINS	1.010609722	1.103257636
**VWA5A**	10.30417128	9.624493223	1.40E-31	10.30417128	10.04003559	0.000598025	ECM_GLYCOPROTEINS	1.07061962	1.026308242
**VWA5B2**	5.427716462	4.443911154	7.55E-20	5.427716462	4.663959322	1.03E-05	ECM_GLYCOPROTEINS	1.221382758	1.163757247

**VWA7**	8.128580035	6.503785629	3.15E-98	8.128580035	7.673132203	0.000101397	ECM_GLYCOPROTEINS	1.249822872	1.059356182
**VWF**	12.68139159	12.17949211	3.38E-14	12.68139159	12.32339492	0.012134172	ECM_GLYCOPROTEINS	1.041208572	1.029050167

Thymoma (THYM)

Signature genes	Expression in tumor	Expression in rest of Pan-Cancer	P-value	Expression in tumor	Expression in normal tissue	P-value	Matrisome category	Fold change vs. Pan-Cancer	Fold change vs. normal
**IGFBP2**	13.22729587	11.54194636	4.04E-15	13.22729587	11.0395	0.023480987	ECM_GLYCOPROTEINS	1.146019523	1.198178891

Uterine corpus endometrioid carcinoma (UCEC)

Signature genes	Expression in tumor	Expression in rest of Pan-Cancer	P-value	Expression in tumor	Expression in normal tissue	P-value	Matrisome category	Fold change vs. Pan-Cancer	Fold change vs. normal
ADAM2	1.25977451	0.555867918	9.12E-11	1.25977451	0.176886957	0.007768883	ECM_REGULATORS	2.266319874	7.121918623
ADAMTS14	7.009025	6.637393689	0.012339467	7.009025	5.380973913	1.95E-05	ECM_REGULATORS	1.055990548	1.302556956
ADAMTS15	9.175752941	7.674470536	1.67E-19	9.175752941	8.121178261	0.017450027	ECM_REGULATORS	1.195620323	1.129854886
AGRN	13.75570882	13.22559015	3.79E-09	13.75570882	12.39993913	2.23E-09	BASEMENT_MEMBRANES	1.040082799	1.109336802
AGRN	13.75570882	13.22559015	3.79E-09	13.75570882	12.39993913	2.23E-09	ECM_GLYCOPROTEINS	1.040082799	1.109336802
AMBN	1.137540686	0.341289915	6.00E-29	1.137540686	0.070569565	0.04312775	ECM_GLYCOPROTEINS	3.333062704	16.11942319
AMH	5.806248529	4.810524026	5.68E-09	5.806248529	3.61996087	5.78E-05	SECRETED_FACTORS	1.206988781	1.603953396
ANXA3	9.906577451	9.076169795	1.36E-05	9.906577451	9.286073913	0.027298608	ECM_AFFILIATED	1.091493182	1.06682087
ARTN	6.930994118	5.393294866	6.97E-24	6.930994118	5.172126087	1.42E-09	SECRETED_FACTORS	1.285113143	1.340066735
BMP3	5.436103431	3.637397748	5.29E-15	5.436103431	4.091126087	0.031072481	SECRETED_FACTORS	1.494503436	1.328754801
C1QL4	5.277604902	3.43054719	1.20E-21	5.277604902	2.624256522	1.55E-06	ECM_AFFILIATED	1.538414897	2.011085753
C1QTNF6	9.882488725	9.372857133	2.09E-07	9.882488725	9.2978	0.027416453	ECM_AFFILIATED	1.054373131	1.062884631
CCL25	2.945980882	2.153530357	1.26E-05	2.945980882	1.777291304	0.004965522	SECRETED_FACTORS	1.367977411	1.657567825
CCL28	8.281962255	7.860008797	0.011459314	8.281962255	7.433013043	0.017822473	SECRETED_FACTORS	1.053683586	1.114213335
CCL7	2.698642157	2.221031615	0.000923369	2.698642157	0.983704348	2.93E-05	SECRETED_FACTORS	1.215039956	2.743346782
CELA3B	0.994448039	0.503059487	1.47E-05	0.994448039	0.277021739	0.010281359	ECM_REGULATORS	1.976800092	3.589783395
CILP2	7.118513235	6.188143638	1.32E-07	7.118513235	4.789313043	7.82E-07	ECM_GLYCOPROTEINS	1.150347124	1.486332835
CLEC18B	5.340330882	4.364986404	3.05E-07	5.340330882	3.29926087	9.56E-06	ECM_AFFILIATED	1.223447312	1.618644628
COCH	6.96695049	6.48871872	0.007368587	6.96695049	5.475704348	0.000370407	ECM_GLYCOPROTEINS	1.073702034	1.272338689
COL18A1	14.20038775	12.81815479	2.75E-40	14.20038775	13.63617826	0.005368289	BASEMENT_MEMBRANES	1.107834004	1.041375925
COL18A1	14.20038775	12.81815479	2.75E-40	14.20038775	13.63617826	0.005368289	COLLAGENS	1.107834004	1.041375925
COL26A1	8.344038235	4.61903543	3.93E-81	8.344038235	5.604513043	1.79E-05	COLLAGENS	1.806446034	1.488806997
COL9A1	5.775592157	3.818673772	5.54E-23	5.775592157	3.733295652	0.007373962	COLLAGENS	1.512460216	1.547049228
COL9A2	10.62331618	8.640044349	4.41E-40	10.62331618	9.057813043	9.92E-05	COLLAGENS	1.229544172	1.17283456
CRELD2	10.90774951	10.41534795	1.15E-17	10.90774951	10.19903478	0.000323651	ECM_GLYCOPROTEINS	1.047276535	1.069488412
CSPG5	7.208827941	5.934973549	1.70E-12	7.208827941	4.663552174	8.94E-10	ECM_AFFILIATED	1.214635227	1.545780485
CST6	5.639136765	4.891841782	0.001542159	5.639136765	3.41056087	2.35E-05	ECM_REGULATORS	1.152763523	1.653433843
CSTB	12.95240686	12.69972501	0.00659123	12.95240686	11.57508696	4.48E-11	ECM_REGULATORS	1.01989664	1.118990027
CSTL1	0.794610294	0.550957741	0.000374308	0.794610294	0.070821739	0.006068432	ECM_REGULATORS	1.442234557	11.21986418
CTSV	9.830607353	7.273498235	6.91E-43	9.830607353	6.566747826	5.36E-10	ECM_REGULATORS	1.351565235	1.497028303
CXCL3	5.983512255	5.303557102	0.000316713	5.983512255	4.622030435	0.008136191	SECRETED_FACTORS	1.128207378	1.294563577
CXCL5	5.951929412	5.052951755	0.000211918	5.951929412	3.418186957	0.000292262	SECRETED_FACTORS	1.177911387	1.741253327
EGFL6	7.937783333	5.700566873	2.38E-26	7.937783333	6.408521739	0.001014967	SECRETED_FACTORS	1.392455086	1.238629384
EGLN3	10.64701225	9.586904109	3.93E-10	10.64701225	9.265617391	7.59E-05	ECM_REGULATORS	1.110578778	1.14908827
EMID1	10.93848529	7.99631111	2.84E-118	10.93848529	10.09030435	0.039632563	ECM_GLYCOPROTEINS	1.367941435	1.084059005
ESM1	8.214661765	7.674007447	0.002872182	8.214661765	5.764843478	1.58E-08	PROTEOGLYCANS	1.07045267	1.424958335
FBN2	9.191601471	7.118162206	1.68E-29	9.191601471	7.685291304	0.012510179	ECM_GLYCOPROTEINS	1.291288566	1.195999098
FGF18	9.066966176	4.587133837	2.48E-222	9.066966176	6.553856522	8.90E-08	SECRETED_FACTORS	1.976608161	1.383455092
FGF3	1.542066667	0.352845323	4.85E-41	1.542066667	0.113369565	0.009915844	SECRETED_FACTORS	4.370375821	13.60212208
FGF4	1.205833333	0.464769136	2.66E-12	1.205833333	0.084634783	0.02339458	SECRETED_FACTORS	2.594478078	14.24749135
FRAS1	9.84159902	8.321466934	9.45E-18	9.84159902	8.825286957	0.007026122	ECM_GLYCOPROTEINS	1.182675975	1.115159095

**GPC2**	6.881244118	5.516158391	7.90E-20	6.881244118	5.452595652	8.98E-05	ECM_AFFILIATED	1.247470364	1.262012545
**HAPLN1**	7.217173039	5.175917735	2.25E-21	7.217173039	2.752743478	1.40E-14	PROTEOGLYCANS	1.394375531	2.621810966
**HAPLN3**	10.69941225	8.43607989	1.27E-73	10.69941225	8.978226087	6.33E-07	PROTEOGLYCANS	1.268291955	1.191706708
**HYAL3**	7.521334314	7.050463809	1.33E-11	7.521334314	6.614234783	8.31E-06	ECM_REGULATORS	1.066785749	1.137143534
**IGFBP2**	13.41750098	11.38359689	1.16E-36	13.41750098	12.29811739	0.004892505	ECM_GLYCOPROTEINS	1.178669722	1.091020727
**KAZALD1**	9.309138725	7.345047088	4.17E-57	9.309138725	8.446686957	0.026069037	ECM_REGULATORS	1.267403546	1.102105331
**KCP**	6.880545098	5.905436181	4.34E-09	6.880545098	5.01146087	0.000248199	ECM_GLYCOPROTEINS	1.165120558	1.372961952
**LAMA1**	9.202537745	7.303030205	2.95E-22	9.202537745	7.163873913	0.000226808	ECM_GLYCOPROTEINS	1.260098546	1.284575616
**LAMA1**	9.202537745	7.303030205	2.95E-22	9.202537745	7.163873913	0.000226808	BASEMENT_MEMBRANES	1.260098546	1.284575616
**LAMA5**	13.13214314	12.23349818	2.12E-20	13.13214314	12.27125652	0.000135433	BASEMENT_MEMBRANES	1.073457726	1.070154724
**LAMA5**	13.13214314	12.23349818	2.12E-20	13.13214314	12.27125652	0.000135433	ECM_GLYCOPROTEINS	1.073457726	1.070154724
**LAMB4**	3.686614706	3.049299148	5.68E-07	3.686614706	2.650082609	0.004067901	BASEMENT_MEMBRANES	1.209003947	1.391131995
**LAMB4**	3.686614706	3.049299148	5.68E-07	3.686614706	2.650082609	0.004067901	ECM_GLYCOPROTEINS	1.209003947	1.391131995
**LAMC2**	11.81622843	9.999060248	7.58E-11	11.81622843	10.3531	0.00065014	ECM_GLYCOPROTEINS	1.181733897	1.141322737
**LAMC2**	11.81622843	9.999060248	7.58E-11	11.81622843	10.3531	0.00065014	BASEMENT_MEMBRANES	1.181733897	1.141322737
**LGALS14**	0.891853431	0.363141427	6.47E-15	0.891853431	0.179104348	0.030346367	ECM_AFFILIATED	2.455939659	4.9795186
**LGALS7**	3.358035784	2.66306872	0.002785256	3.358035784	1.202713043	6.77E-05	ECM_AFFILIATED	1.260964751	2.792050691
**LGALS9**	11.59434167	10.8248943	8.47E-14	11.59434167	10.73176957	0.001530684	ECM_AFFILIATED	1.071081282	1.080375571
**LOXL1**	9.91242598	9.145221246	2.97E-08	9.91242598	9.456017391	0.040403901	ECM_REGULATORS	1.083891326	1.048266471
**LRG1**	9.677417647	8.301315179	1.07E-10	9.677417647	8.565013043	0.020659627	ECM_GLYCOPROTEINS	1.165769211	1.129877748
**MATN4**	3.362613725	2.522882143	1.89E-11	3.362613725	1.969578261	0.000936568	ECM_GLYCOPROTEINS	1.33284614	1.707276016
**MFAP2**	11.8549451	8.950824838	2.39E-53	11.8549451	10.9845	0.020607537	ECM_GLYCOPROTEINS	1.324452809	1.079243033
**MMP10**	6.72962451	4.640318172	6.32E-16	6.72962451	4.30173913	0.001390364	ECM_REGULATORS	1.450250664	1.564396237
**MMP11**	12.44240245	9.873782752	1.82E-37	12.44240245	11.39246087	0.010579988	ECM_REGULATORS	1.260145454	1.092161087
**MMP12**	7.007539706	5.718196073	3.39E-06	7.007539706	2.976891304	3.52E-11	ECM_REGULATORS	1.225480836	2.35397903
**MMP15**	11.36485588	10.75782345	2.07E-08	11.36485588	9.910326087	1.04E-09	ECM_REGULATORS	1.056427068	1.146769116
**MUC1**	13.4798201	10.67021314	3.67E-34	13.4798201	11.26607391	4.44E-07	ECM_AFFILIATED	1.263313106	1.196496686
**MUC16**	10.96241912	4.797925548	1.06E-114	10.96241912	9.406586957	0.008556031	ECM_AFFILIATED	2.284824766	1.165398159
**MUC20**	10.0669049	8.894625619	2.35E-10	10.0669049	8.74333913	0.002719038	ECM_AFFILIATED	1.13179636	1.151379896
**MUC4**	7.290420098	6.646361627	0.019074082	7.290420098	5.7899	0.012720882	ECM_AFFILIATED	1.096903916	1.25916166
**MUC5AC**	5.143475	3.154161932	2.00E-11	5.143475	1.70486087	1.71E-06	ECM_AFFILIATED	1.630694654	3.016947067
**NGLY1**	10.09489608	9.957309619	0.000680384	10.09489608	9.664343478	4.26E-05	ECM_REGULATORS	1.013817634	1.044550631
**OGFOD2**	9.398629412	9.065212338	9.54E-13	9.398629412	8.88106087	0.000633873	ECM_REGULATORS	1.036779842	1.058277783
**OTOG**	2.449189706	1.231771002	2.49E-20	2.449189706	0.231069565	1.02E-06	ECM_GLYCOPROTEINS	1.988348241	10.59936086
**P4HA1**	11.55693529	11.10732871	1.57E-10	11.55693529	10.88210435	4.28E-05	ECM_REGULATORS	1.040478372	1.062012909
**PI3**	7.432301471	5.777735146	3.08E-07	7.432301471	2.92613913	4.94E-09	ECM_REGULATORS	1.286369361	2.539968586
**PLOD1**	13.48194216	12.50256311	2.64E-46	13.48194216	12.6600087	6.24E-06	ECM_REGULATORS	1.078334262	1.06492361
**PLXNB1**	12.54746667	11.80080558	9.76E-19	12.54746667	11.80721304	0.000182058	ECM_AFFILIATED	1.063272044	1.062695034
**PLXNB2**	13.84316618	13.66575592	0.003596769	13.84316618	13.46838696	0.010400115	ECM_AFFILIATED	1.012982104	1.027826585
**PRSS1**	3.392787745	1.860342269	3.56E-13	3.392787745	0.573565217	8.33E-05	ECM_REGULATORS	1.823743836	5.915260623
**PRSS2**	5.185112255	2.955715686	3.46E-19	5.185112255	1.281695652	5.30E-08	ECM_REGULATORS	1.754266244	4.045509748
**RSPO4**	4.789696078	4.043109953	7.59E-05	4.789696078	3.29873913	0.024422621	ECM_GLYCOPROTEINS	1.184656399	1.451977828
**SEMA3F**	11.03173235	10.55742698	0.000230002	11.03173235	10.24145652	0.000326563	ECM_AFFILIATED	1.044926228	1.077164398
**SEMA4D**	10.74612059	10.42550944	6.80E-05	10.74612059	10.0256913	2.92E-06	ECM_AFFILIATED	1.030752565	1.071858315
**SEMA4F**	8.855987745	8.659497007	0.007053614	8.855987745	8.48786087	0.021818293	ECM_AFFILIATED	1.02269078	1.043370984
**SFTA2**	5.009406863	3.122960166	1.89E-13	5.009406863	3.440447826	0.021089012	ECM_AFFILIATED	1.604057239	1.456033376

**SLPI**	13.43571029	9.350146459	7.97E-50	13.43571029	10.82352609	8.95E-06	ECM_REGULATORS	1.436951854	1.24134318
**SPP1**	12.42730392	11.94316859	0.030259761	12.42730392	9.076826087	1.69E-11	ECM_GLYCOPROTEINS	1.04053659	1.369124384
**ST14**	13.16738873	11.62216987	4.03E-17	13.16738873	10.1137087	6.47E-15	ECM_REGULATORS	1.13295442	1.301934742
**TGM3**	5.601064216	3.9904153	2.29E-12	5.601064216	3.809708696	0.002393461	ECM_REGULATORS	1.403629395	1.470208004
**TGM5**	4.021237255	2.949439448	1.27E-08	4.021237255	1.308186957	2.63E-07	ECM_REGULATORS	1.363390341	3.073901047
**TGM7**	3.607103922	0.533153805	1.47E-304	3.607103922	1.219634783	1.16E-05	ECM_REGULATORS	6.765597261	2.95752792
**WNT10A**	6.392014216	5.772225994	0.000871628	6.392014216	4.16966087	0.000180249	SECRETED_FACTORS	1.107374213	1.532981798
**WNT3A**	3.423218137	2.756148488	0.000405517	3.423218137	2.221704348	0.008000136	SECRETED_FACTORS	1.242029648	1.540807237
**WNT7A**	8.431003431	3.478866092	4.96E-105	8.431003431	5.876117391	0.000917971	SECRETED_FACTORS	2.423491796	1.434791525
**VWA1**	12.1498848	11.46398755	5.26E-12	12.1498848	11.65517391	0.015342231	ECM_GLYCOPROTEINS	1.0598306	1.042445604
**ZP3**	8.354165196	7.719755915	8.58E-10	8.354165196	7.57853913	0.005926778	ECM_GLYCOPROTEINS	1.082179966	1.102345063

Uterine Carcinosarcoma (UCS)

Signature genes	Expression in tumor	Expression in rest of Pan-Cancer	P-value	Expression in tumor	Expression in normal tissue	P-value	Matrisome category	Fold change vs. Pan-Cancer	Fold change vs. normal
**ACAN**	7.857208772	6.486774535	1.84E-05	7.857208772	5.695634783	9.38E-05	PROTEOGLYCANS	1.211265896	1.379514149
**ADAM12**	9.442970175	8.45376836	0.003707233	9.442970175	7.305026087	0.000101758	ECM_REGULATORS	1.11701312	1.29266755
**ADAM18**	0.923198246	0.466232387	0.006022229	0.923198246	0.033043478	0.003379328	ECM_REGULATORS	1.980124656	27.93889428
**ADAM19**	9.859257895	9.052868667	0.00019911	9.859257895	8.919530435	0.000189325	ECM_REGULATORS	1.089075547	1.105356158
**ADAM21**	4.239205263	2.916992167	1.53E-11	4.239205263	2.62113913	2.95E-05	ECM_REGULATORS	1.453279618	1.617314096
**ADAM23**	7.990363158	6.804231515	0.000988143	7.990363158	6.880869565	0.025151768	ECM_REGULATORS	1.174322646	1.161243224
**ADAMTS12**	8.879340351	8.015026999	0.005520197	8.879340351	8.016113043	0.01042488	ECM_REGULATORS	1.107836611	1.107686519
**ADAMTS14**	7.927517544	6.694489942	9.52E-06	7.927517544	5.380973913	3.94E-08	ECM_REGULATORS	1.184185444	1.473249577
**ADAMTS15**	9.476315789	7.768265369	4.68E-08	9.476315789	8.121178261	0.002199085	ECM_REGULATORS	1.219875395	1.166864645
**ADAMTS17**	8.175587719	6.483051256	5.60E-15	8.175587719	7.078534783	0.004868359	ECM_REGULATORS	1.261070967	1.154983054
**ADAMTS20**	2.683473684	1.750250412	0.002409239	2.683473684	0.429730435	8.21E-06	ECM_REGULATORS	1.533194145	6.244551158
**ADAMTS7**	10.60375439	8.052027565	4.14E-36	10.60375439	8.634178261	1.33E-08	ECM_REGULATORS	1.316904879	1.228113906
**ADAMTSL2**	8.75067193	8.187550441	0.014007598	8.75067193	7.361986957	3.78E-05	ECM_REGULATORS	1.068777773	1.1886291
**AMBN**	1.647994737	0.341967584	4.58E-23	1.647994737	0.070569565	0.000103876	ECM_GLYCOPROTEINS	4.819154839	23.35276874
**AMELX**	1.581361404	0.312183586	7.52E-26	1.581361404	0	0.000313026	ECM_GLYCOPROTEINS	5.065485424	Inf
**AMH**	6.18262807	4.842549482	3.08E-05	6.18262807	3.61996087	7.34E-08	SECRETED_FACTORS	1.276729973	1.707926769
**ANGPTL6**	4.477691228	3.967031074	0.016379767	4.477691228	2.269	7.88E-07	SECRETED_FACTORS	1.128726028	1.97342055
**ARTN**	6.427557895	5.505518121	0.001378862	6.427557895	5.172126087	0.000293303	SECRETED_FACTORS	1.167475568	1.242730318
**ASTL**	3.431582456	1.932187919	2.04E-13	3.431582456	2.129182609	0.001499684	ECM_REGULATORS	1.776008649	1.611690065
**BDNF**	6.504277193	5.220632148	4.13E-06	6.504277193	3.41316087	4.25E-11	SECRETED_FACTORS	1.245879236	1.905646245
**BGN**	14.20404211	13.2232426	0.000212393	14.20404211	12.8896	0.000106175	PROTEOGLYCANS	1.07417239	1.101976951
**BMP1**	11.57102982	10.63382365	1.65E-10	11.57102982	10.78095652	0.001204345	ECM_REGULATORS	1.088134447	1.073284156
**BMP3**	5.417921053	3.680793931	5.08E-05	5.417921053	4.091126087	0.038653378	SECRETED_FACTORS	1.471943595	1.324310456
**BMP5**	6.84017193	3.524598715	1.75E-16	6.84017193	0.718186957	7.69E-11	SECRETED_FACTORS	1.940695234	9.524221886
**BMP7**	10.94862632	8.013109166	3.51E-09	10.94862632	7.91606087	1.14E-06	SECRETED_FACTORS	1.366339343	1.383090213
**C1QL1**	7.730636842	5.886649377	5.44E-06	7.730636842	4.705247826	2.98E-09	ECM_AFFILIATED	1.313249074	1.642981864
**C1QL2**	2.239747368	1.613373394	0.042913158	2.239747368	0.330543478	3.32E-06	ECM_AFFILIATED	1.388238691	6.775953893
**C1QL4**	7.521966667	3.567848591	1.09E-26	7.521966667	2.624256522	2.87E-15	ECM_AFFILIATED	2.108263979	2.866322939
**C1QTNF3**	9.453675439	7.926686692	2.80E-09	9.453675439	8.301356522	0.002913023	ECM_AFFILIATED	1.192638968	1.138810918
**C1QTNF6**	10.25313509	9.389791841	2.69E-06	10.25313509	9.2978	0.001117695	ECM_AFFILIATED	1.091944876	1.102748509
**CCL25**	3.101917544	2.257443643	0.01395723	3.101917544	1.777291304	0.004020765	SECRETED_FACTORS	1.374084156	1.745306206
**CCL7**	3.455363158	2.299664669	2.56E-05	3.455363158	0.983704348	1.12E-07	SECRETED_FACTORS	1.502550874	3.51260332
**CELA3A**	1.789129825	0.54098906	3.31E-07	1.789129825	0.305256522	0.008552786	ECM_REGULATORS	3.307146032	5.861069943
**CELA3B**	1.03767193	0.521781812	0.014663176	1.03767193	0.277021739	0.044945948	ECM_REGULATORS	1.98870851	3.745814076
**CILP2**	9.80345614	6.318937459	4.40E-25	9.80345614	4.789313043	3.56E-21	ECM_GLYCOPROTEINS	1.551440603	2.046944113
**CLEC18A**	5.695898246	4.963214036	0.022607412	5.695898246	4.195034783	0.003358875	ECM_AFFILIATED	1.147622932	1.357771399
**CLEC18B**	5.856829825	4.468262128	0.000116954	5.856829825	3.29926087	1.66E-06	ECM_AFFILIATED	1.310762363	1.775194523
**CLEC18C**	4.153394737	3.441509923	0.044624202	4.153394737	2.504004348	0.004341649	ECM_AFFILIATED	1.206852466	1.658701088
**CLEC4F**	5.618907018	3.273690719	2.87E-21	5.618907018	4.591465217	0.005283449	ECM_AFFILIATED	1.716382976	1.223772097
**COCH**	8.167147368	6.465038523	3.32E-07	8.167147368	5.475704348	3.89E-09	ECM_GLYCOPROTEINS	1.263278995	1.491524533
**COL11A1**	9.379336842	7.404896405	9.85E-05	9.379336842	4.596656522	4.76E-08	COLLAGENS	1.266639846	2.040469371

**COL11A2**	10.02298947	5.967268255	1.40E-45	10.02298947	5.935082609	2.87E-11	COLLAGENS	1.679661286	1.688770003
**COL13A1**	8.551903509	6.36770464	1.59E-18	8.551903509	6.571269565	1.14E-05	COLLAGENS	1.343011963	1.301408111
**COL19A1**	3.872833333	2.56764302	1.84E-05	3.872833333	1.078930435	3.05E-05	COLLAGENS	1.508322342	3.58951162
**COL1A1**	16.86772982	14.7236684	1.25E-07	16.86772982	15.53188261	0.002901817	COLLAGENS	1.145620057	1.08600678
**COL22A1**	8.319963158	6.536482608	1.15E-06	8.319963158	6.779047826	0.021960001	COLLAGENS	1.272850194	1.22730557
**COL23A1**	9.58142807	6.574815896	9.54E-18	9.58142807	7.756191304	9.91E-06	COLLAGENS	1.457292222	1.235326424
**COL24A1**	7.054342105	5.321554832	9.83E-11	7.054342105	5.235378261	2.70E-05	COLLAGENS	1.325616728	1.347436948
**COL25A1**	8.235794737	3.874184458	7.71E-44	8.235794737	4.969104348	2.61E-09	COLLAGENS	2.125813788	1.65740024
**COL26A1**	11.21732281	4.779782704	2.37E-66	11.21732281	5.604513043	3.25E-22	COLLAGENS	2.346826938	2.001480364
**COL2A1**	11.63325263	4.132532848	7.36E-63	11.63325263	4.445930435	6.87E-16	COLLAGENS	2.815041782	2.616606985
**COL4A1**	14.07948246	13.18608188	0.000391508	14.07948246	13.23313913	0.00317155	BASEMENT_MEMBRANES	1.067753301	1.063956354
**COL4A1**	14.07948246	13.18608188	0.000391508	14.07948246	13.23313913	0.00317155	COLLAGENS	1.067753301	1.063956354
**COL5A2**	13.3406386	11.49619736	3.26E-10	13.3406386	11.48836957	3.66E-06	COLLAGENS	1.160439246	1.161229931
**COL8A2**	11.04227719	8.903040211	1.69E-14	11.04227719	8.581756522	2.01E-08	COLLAGENS	1.240281626	1.286715274
**COL9A1**	7.178663158	3.948480326	2.02E-17	7.178663158	3.733295652	1.60E-05	COLLAGENS	1.818082544	1.922875611
**COL9A2**	11.34673158	8.736522924	3.42E-20	11.34673158	9.057813043	1.14E-07	COLLAGENS	1.298769737	1.252701013
**COL9A3**	11.05883509	6.89508861	1.28E-35	11.05883509	6.691252174	2.99E-13	COLLAGENS	1.603871352	1.652730281
**CRELD2**	10.68861754	10.39259592	0.007323398	10.68861754	10.19903478	0.004449481	ECM_GLYCOPROTEINS	1.028483897	1.048002852
**CRISPLD1**	10.52219123	8.102431429	2.32E-15	10.52219123	9.166808696	6.60E-05	ECM_GLYCOPROTEINS	1.298646131	1.147857622
**CRLF1**	8.930954386	7.079522474	8.71E-11	8.930954386	6.027856522	1.71E-08	SECRETED_FACTORS	1.261519321	1.481613631
**CSH1**	0.664073684	0.235275101	0.000162124	0.664073684	0.033043478	0.032308489	SECRETED_FACTORS	2.822541282	20.09696676
**CSH2**	0.897173684	0.41880372	0.001273547	0.897173684	0.036713043	0.003079961	SECRETED_FACTORS	2.142229501	24.43746416
**CSPG5**	7.002987719	6.048453576	0.004957318	7.002987719	4.663552174	1.19E-11	ECM_AFFILIATED	1.157814577	1.501642409
**CTHRC1**	11.28251228	9.064828389	4.95E-11	11.28251228	7.590926087	3.38E-16	ECM_GLYCOPROTEINS	1.244647091	1.486315655
**CTSV**	8.884829825	7.380766347	1.59E-05	8.884829825	6.566747826	2.78E-09	ECM_REGULATORS	1.203781478	1.353003048
**DSPP**	0.88294386	0.554155829	0.013777276	0.88294386	0.315917391	0.022669162	ECM_GLYCOPROTEINS	1.593313312	2.794856769
**EGFL6**	9.561731579	5.883036999	6.34E-20	9.561731579	6.408521739	9.96E-08	SECRETED_FACTORS	1.625305362	1.492033884
**EGFLAM**	9.150650877	7.619756213	6.22E-11	9.150650877	8.22733913	0.013855457	ECM_GLYCOPROTEINS	1.200911239	1.112224831
**EGLN2**	11.92390526	11.42687881	1.80E-08	11.92390526	11.64072174	0.035876436	ECM_REGULATORS	1.043496257	1.024326973
**ELN**	13.04074386	10.03434578	1.89E-23	13.04074386	11.17933043	9.20E-05	ECM_GLYCOPROTEINS	1.299610771	1.166504912
**ELSPBP1**	1.233740351	0.462908466	2.56E-06	1.233740351	0.042082609	0.006816481	ECM_GLYCOPROTEINS	2.665192887	29.31710721
**EMILIN3**	9.947429825	4.921559415	4.16E-50	9.947429825	8.721282609	0.030799971	ECM_GLYCOPROTEINS	2.021194704	1.140592533
**EPYC**	5.2868	2.56212255	2.07E-13	5.2868	2.319434783	2.44E-05	PROTEOGLYCANS	2.063445404	2.279348417
**ESM1**	9.577270175	7.690784631	1.78E-08	9.577270175	5.764843478	8.25E-15	PROTEOGLYCANS	1.245291688	1.66132354
**FAM20C**	11.29262982	10.67574581	0.000592315	11.29262982	10.43004783	0.000712512	ECM_REGULATORS	1.057783693	1.082701634
**FBLN2**	12.08138596	10.23773861	2.47E-11	12.08138596	10.76645652	0.000334305	ECM_GLYCOPROTEINS	1.180083456	1.122132053
**FBN2**	12.5241614	7.167113298	7.04E-55	12.5241614	7.685291304	1.73E-20	ECM_GLYCOPROTEINS	1.747448503	1.629627415
**FBN3**	9.5832	4.220175139	8.44E-32	9.5832	5.403508696	1.70E-10	ECM_GLYCOPROTEINS	2.270806231	1.773514311
**FGF11**	9.166450877	7.669269674	2.94E-07	9.166450877	6.362547826	2.65E-10	SECRETED_FACTORS	1.195218224	1.440688719
**FGF18**	8.400885965	4.873462493	1.53E-31	8.400885965	6.553856522	3.83E-05	SECRETED_FACTORS	1.723802323	1.281823295
**FGF21**	1.462912281	0.77722977	0.011005119	1.462912281	0.037621739	0.000371925	SECRETED_FACTORS	1.882213391	38.88475957
**FGF23**	2.501312281	0.533258389	4.11E-36	2.501312281	0.319456522	9.54E-05	SECRETED_FACTORS	4.690619653	7.829898939
**FGF3**	1.104749123	0.435866779	0.000242216	1.104749123	0.113369565	0.001399844	SECRETED_FACTORS	2.534602721	9.744671074
**FGF4**	2.005589474	0.48730489	2.96E-14	2.005589474	0.084634783	0.000281819	SECRETED_FACTORS	4.115676891	23.69698854
**FRAS1**	10.52181754	8.388944219	1.09E-10	10.52181754	8.825286957	1.37E-05	ECM_GLYCOPROTEINS	1.254248123	1.192235176
**FREM2**	9.3833	6.670190038	3.07E-09	9.3833	8.082026087	0.008886599	ECM_AFFILIATED	1.406751524	1.161008378

**GPC1**	12.62100175	11.58144586	5.38E-07	12.62100175	11.00271739	1.38E-09	ECM_AFFILIATED	1.089760459	1.147080426
**GPC2**	9.43875614	5.553868993	3.30E-44	9.43875614	5.452595652	5.86E-23	ECM_AFFILIATED	1.699492039	1.731057416
**GPC3**	11.62242982	8.029928351	5.03E-21	11.62242982	8.608508696	1.32E-07	ECM_AFFILIATED	1.447388982	1.350109553
**GPC4**	11.39765789	9.928789367	4.73E-07	11.39765789	10.3643087	0.001642851	ECM_AFFILIATED	1.147940345	1.099702665
**GPC5**	3.718975439	2.905175628	0.015104549	3.718975439	1.530782609	2.83E-06	ECM_AFFILIATED	1.280120693	2.42946021
**HAPLN1**	7.911817544	5.245555877	2.62E-11	7.911817544	2.752743478	8.23E-14	PROTEOGLYCANS	1.508289632	2.87415722
**HAPLN3**	10.02856491	8.533685062	2.64E-10	10.02856491	8.978226087	0.001823549	PROTEOGLYCANS	1.175174012	1.116987344
**HAPLN4**	6.984492982	3.58996976	2.22E-25	6.984492982	5.753495652	0.014236955	PROTEOGLYCANS	1.945557609	1.21395642
**HMCN1**	9.692008772	8.672879108	0.000625867	9.692008772	8.312547826	8.52E-05	BASEMENT_MEMBRANES	1.117507652	1.165949234
**HMCN1**	9.692008772	8.672879108	0.000625867	9.692008772	8.312547826	8.52E-05	ECM_GLYCOPROTEINS	1.117507652	1.165949234
**HYAL3**	7.424075439	7.10865908	0.019266728	7.424075439	6.614234783	0.000597397	ECM_REGULATORS	1.044370725	1.122439055
**IGFBP2**	14.45163509	11.54559689	1.07E-20	14.45163509	12.29811739	2.81E-11	ECM_GLYCOPROTEINS	1.251700992	1.175109541
**IGFBPL1**	4.356285965	3.167931524	0.000169225	4.356285965	2.789078261	0.000420934	ECM_GLYCOPROTEINS	1.37511999	1.561908831
**KAZALD1**	10.26487018	7.396823854	8.09E-35	10.26487018	8.446686957	2.44E-05	ECM_REGULATORS	1.387740249	1.215254008
**KCP**	7.840698246	5.960061908	1.38E-09	7.840698246	5.01146087	3.90E-08	ECM_GLYCOPROTEINS	1.315539732	1.564553421
**KERA**	5.632940351	1.810448523	7.54E-45	5.632940351	3.165634783	0.001546419	PROTEOGLYCANS	3.111350739	1.779403102
**LAMA1**	9.717559649	7.358067872	1.05E-10	9.717559649	7.163873913	3.31E-07	ECM_GLYCOPROTEINS	1.320667303	1.356467153
**LAMA1**	9.717559649	7.358067872	1.05E-10	9.717559649	7.163873913	3.31E-07	BASEMENT_MEMBRANES	1.320667303	1.356467153
**LAMB1**	13.6392807	12.22537678	1.15E-10	13.6392807	12.82277391	0.000349834	ECM_GLYCOPROTEINS	1.115653198	1.063676299
**LAMB1**	13.6392807	12.22537678	1.15E-10	13.6392807	12.82277391	0.000349834	BASEMENT_MEMBRANES	1.115653198	1.063676299
**LAMB4**	3.924826316	3.03011628	0.000159716	3.924826316	2.650082609	0.003028	ECM_GLYCOPROTEINS	1.295272509	1.481020366
**LAMB4**	3.924826316	3.03011628	0.000159716	3.924826316	2.650082609	0.003028	BASEMENT_MEMBRANES	1.295272509	1.481020366
**LAMC1**	13.53805263	12.7946557	2.37E-05	13.53805263	13.0882	0.022559955	ECM_GLYCOPROTEINS	1.058102145	1.034370856
**LAMC1**	13.53805263	12.7946557	2.37E-05	13.53805263	13.0882	0.022559955	BASEMENT_MEMBRANES	1.058102145	1.034370856
**LAMC3**	10.18789123	7.775893365	1.37E-18	10.18789123	8.601456522	7.07E-05	ECM_GLYCOPROTEINS	1.310189164	1.184437915
**LAMC3**	10.18789123	7.775893365	1.37E-18	10.18789123	8.601456522	7.07E-05	BASEMENT_MEMBRANES	1.310189164	1.184437915
**LGALS12**	4.006689474	3.127613413	0.004685601	4.006689474	2.579469565	0.001226089	ECM_AFFILIATED	1.281069283	1.553299767
**LGALS14**	1.104980702	0.450484171	7.45E-06	1.104980702	0.179104348	0.008532951	ECM_AFFILIATED	2.452873539	6.169480055
**LGALS7**	4.074945614	2.677273567	0.001197882	4.074945614	1.202713043	3.91E-08	ECM_AFFILIATED	1.522050516	3.388127896
**LGI1**	4.974812281	2.0159821	4.91E-17	4.974812281	3.152165217	0.006082818	ECM_GLYCOPROTEINS	2.467686733	1.578220663
**LOX**	10.75694211	9.513439971	8.63E-06	10.75694211	9.47846087	2.99E-05	ECM_REGULATORS	1.130710042	1.134882789
**LOXL1**	11.15344561	9.224470422	1.46E-13	11.15344561	9.456017391	5.92E-08	ECM_REGULATORS	1.209115006	1.179507731
**LOXL2**	11.82927544	10.16137143	6.49E-13	11.82927544	9.781604348	2.60E-09	ECM_REGULATORS	1.164141624	1.209338981
**LOXL3**	9.574342105	7.837987862	5.29E-19	9.574342105	8.189065217	7.16E-08	ECM_REGULATORS	1.221530611	1.169161785
**MATN3**	7.748415789	6.481585858	1.78E-05	7.748415789	5.832856522	1.74E-05	ECM_GLYCOPROTEINS	1.195450613	1.328408433
**MATN4**	5.881617544	2.576361151	1.57E-44	5.881617544	1.969578261	2.01E-07	ECM_GLYCOPROTEINS	2.282916563	2.986231957
**MFAP2**	13.34495965	9.097937277	2.90E-32	13.34495965	10.9845	5.79E-10	ECM_GLYCOPROTEINS	1.466811569	1.21489004
**MMP10**	6.455108772	4.682603375	0.000240637	6.455108772	4.30173913	0.004234195	ECM_REGULATORS	1.378529902	1.500581178
**MMP14**	14.00286316	12.93796569	3.53E-06	14.00286316	13.44623913	0.021650972	ECM_REGULATORS	1.082307953	1.041396261
**MMP9**	10.79390351	9.363519549	0.000142026	10.79390351	6.786030435	8.31E-12	ECM_REGULATORS	1.152761358	1.59060641
**MXRA5**	11.79075614	11.03768365	0.025742054	11.79075614	10.25813913	0.000188782	ECM_GLYCOPROTEINS	1.068227403	1.149404974
**NCAN**	3.561282456	2.460639233	0.018678711	3.561282456	0.897195652	2.17E-06	PROTEOGLYCANS	1.447299713	3.969348767
**NELL1**	5.942403509	3.314288399	1.14E-09	5.942403509	4.485578261	0.029882264	ECM_GLYCOPROTEINS	1.792965124	1.324779808
**NELL2**	9.188915789	7.833896836	0.000163949	9.188915789	7.346891304	0.000493055	ECM_GLYCOPROTEINS	1.1729687	1.250721619
**NID2**	10.27370351	9.201504602	3.07E-05	10.27370351	9.310926087	0.030002765	BASEMENT_MEMBRANES	1.116524303	1.103402971
**NID2**	10.27370351	9.201504602	3.07E-05	10.27370351	9.310926087	0.030002765	ECM_GLYCOPROTEINS	1.116524303	1.103402971

**NTN3**	4.326480702	2.098858236	2.16E-24	4.326480702	2.4246	1.20E-05	BASEMENT_MEMBRANES	2.061349656	1.784410089
**NTN3**	4.326480702	2.098858236	2.16E-24	4.326480702	2.4246	1.20E-05	ECM_GLYCOPROTEINS	2.061349656	1.784410089
**NTNG1**	7.342021053	4.285669271	1.73E-14	7.342021053	5.066695652	3.65E-05	BASEMENT_MEMBRANES	1.71315624	1.449074813
**NTNG1**	7.342021053	4.285669271	1.73E-14	7.342021053	5.066695652	3.65E-05	ECM_GLYCOPROTEINS	1.71315624	1.449074813
**OGFOD2**	9.278614035	9.067047661	0.016410923	9.278614035	8.88106087	0.003678405	ECM_REGULATORS	1.023333546	1.044764153
**OTOG**	4.2852	1.285782167	3.26E-33	4.2852	0.231069565	5.16E-15	ECM_GLYCOPROTEINS	3.332757376	18.54506454
**P4HA1**	12.14595439	11.13278637	1.81E-14	12.14595439	10.88210435	1.78E-10	ECM_REGULATORS	1.091007587	1.116140224
**P4HA2**	11.13148246	10.70100803	0.023755406	11.13148246	10.62626957	0.040650546	ECM_REGULATORS	1.040227465	1.047543768
**P4HA3**	7.25905614	6.373507766	0.000558647	7.25905614	5.673213043	0.00019525	ECM_REGULATORS	1.138942072	1.279531737
**PCOLCE**	13.6178614	10.57201325	5.43E-38	13.6178614	12.19323478	0.000123535	ECM_GLYCOPROTEINS	1.288104837	1.116837463
**PI3**	7.125812281	5.938276826	0.049579035	7.125812281	2.92613913	1.17E-09	ECM_REGULATORS	1.199979807	2.435226749
**PLOD1**	13.87145088	12.51400308	5.48E-26	13.87145088	12.6600087	1.09E-09	ECM_REGULATORS	1.108474306	1.09569047
**PLOD2**	11.74594211	11.15689563	0.013752664	11.74594211	10.61473478	1.92E-06	ECM_REGULATORS	1.052796629	1.106569532
**PLOD3**	11.99037193	11.68737523	0.030293077	11.99037193	11.29513913	8.39E-06	ECM_REGULATORS	1.025925128	1.061551504
**PLXNA1**	12.25405439	11.51655832	1.33E-05	12.25405439	10.75556522	1.12E-13	ECM_AFFILIATED	1.064037887	1.139322215
**PLXNA3**	11.19935614	10.73462003	0.00138999	11.19935614	10.34557826	5.73E-07	ECM_AFFILIATED	1.043293206	1.082525873
**PLXNA4**	8.678084211	6.935316922	2.49E-07	8.678084211	7.595526087	0.010902067	ECM_AFFILIATED	1.251288774	1.142525759
**PROL1**	1.191154386	0.619904631	0.015733932	1.191154386	0.102834783	0.007288506	ECM_AFFILIATED	1.921512321	11.58318573
**PXDN**	12.92707018	11.25729699	4.13E-12	12.92707018	11.6447087	0.000939572	ECM_GLYCOPROTEINS	1.148328074	1.110123964
**REG3G**	1.877796491	0.956257526	0.001418898	1.877796491	0.569430435	0.012908459	ECM_AFFILIATED	1.963693293	3.297674969
**RSPO2**	4.289392982	2.671057814	7.78E-06	4.289392982	1.904308696	6.84E-05	ECM_GLYCOPROTEINS	1.605878001	2.252467256
**RSPO4**	7.816745614	4.17764836	1.47E-23	7.816745614	3.29873913	2.09E-12	ECM_GLYCOPROTEINS	1.871087497	2.369616179
**SEMA3A**	9.466963158	6.917994008	5.95E-17	9.466963158	7.314426087	1.91E-07	ECM_AFFILIATED	1.368454952	1.29428653
**SEMA3F**	11.55513684	10.61948173	0.000107924	11.55513684	10.24145652	1.65E-07	ECM_AFFILIATED	1.088107418	1.128270849
**SEMA4F**	9.490584211	8.666278073	1.28E-09	9.490584211	8.48786087	1.76E-06	ECM_AFFILIATED	1.095116511	1.118136166
**SEMA4G**	9.053933333	8.497229655	0.030194132	9.053933333	7.531878261	3.53E-08	ECM_AFFILIATED	1.065515904	1.202081741
**SEMA5B**	7.854226316	6.885354046	0.002202062	7.854226316	7.134808696	0.039862658	ECM_AFFILIATED	1.140714953	1.100832083
**SEMA6A**	10.98689649	9.584409012	1.22E-07	10.98689649	9.945443478	0.000425377	ECM_AFFILIATED	1.146330095	1.104716599
**SEMA6C**	10.98434386	8.359419559	9.78E-53	10.98434386	9.628904348	4.27E-05	ECM_AFFILIATED	1.314007962	1.140767782
**SEMA7A**	9.148052632	8.479781438	0.002508096	9.148052632	7.6902	3.26E-09	ECM_AFFILIATED	1.078807596	1.189572785
**SERPINE2**	11.35175965	10.73760217	0.047278163	11.35175965	10.41669565	0.00592546	ECM_REGULATORS	1.057196893	1.089765894
**SERPINH1**	14.72549649	12.68675741	8.49E-29	14.72549649	13.41794348	4.94E-08	ECM_REGULATORS	1.1606982	1.097448094
**SLIT1**	7.813319298	5.421693615	1.34E-09	7.813319298	4.235826087	1.39E-11	ECM_GLYCOPROTEINS	1.441121512	1.844579815
**TECTA**	6.280112281	5.16543001	6.75E-09	6.280112281	5.562973913	0.00132338	ECM_GLYCOPROTEINS	1.215796607	1.128912768
**TGFBI**	13.42976842	12.29033827	1.90E-05	13.42976842	11.8961	8.96E-05	ECM_GLYCOPROTEINS	1.092709421	1.128921951
**THBS3**	11.15468772	9.852980566	9.50E-22	11.15468772	10.62878261	0.020141688	ECM_GLYCOPROTEINS	1.132113034	1.049479336
**TLL2**	7.168792982	4.899537795	3.06E-21	7.168792982	5.41983913	7.05E-06	ECM_REGULATORS	1.463156992	1.32269479
**TMPRSS15**	3.112919298	0.988431333	4.66E-20	3.112919298	0.354991304	1.81E-06	ECM_REGULATORS	3.149353117	8.769001551
**TNN**	5.674526316	4.71568373	0.001548125	5.674526316	3.413686957	5.39E-05	ECM_GLYCOPROTEINS	1.203330554	1.662286668
**VCAN**	13.16881228	11.66940803	1.53E-06	13.16881228	11.1748087	7.96E-06	PROTEOGLYCANS	1.128490172	1.17843738
**WIF1**	8.586461404	3.310739837	1.73E-31	8.586461404	6.707052174	0.024231345	SECRETED_FACTORS	2.593517409	1.280213897
**WNT1**	1.009615789	0.69683278	0.015227243	1.009615789	0.480969565	0.038646589	SECRETED_FACTORS	1.448863799	2.099126145
**WNT10A**	8.242982456	5.956999712	4.61E-10	8.242982456	4.16966087	5.17E-12	SECRETED_FACTORS	1.383747332	1.976895175
**WNT10B**	5.61484386	4.228883337	5.53E-08	5.61484386	3.3326	1.49E-07	SECRETED_FACTORS	1.32773676	1.684823819
**WNT11**	9.542180702	5.989914305	1.25E-28	9.542180702	7.272926087	6.05E-09	SECRETED_FACTORS	1.593041272	1.31201398
**WNT16**	4.596542105	2.57407883	2.05E-15	4.596542105	2.654178261	0.001484397	SECRETED_FACTORS	1.785703705	1.731813636

**WNT3A**	3.784238596	2.81083373	0.005595722	3.784238596	2.221704348	0.000467444	SECRETED_FACTORS	1.346304677	1.703304312
**WNT6**	6.889745614	3.82567814	4.14E-27	6.889745614	5.785473913	0.002574769	SECRETED_FACTORS	1.800921395	1.190869705
**VWA5B1**	2.958445614	1.904118313	0.000372085	2.958445614	1.954904348	0.038024275	ECM_GLYCOPROTEINS	1.553708924	1.513345457
**VWCE**	8.284540351	5.819285762	4.78E-18	8.284540351	6.976134783	0.00098745	ECM_GLYCOPROTEINS	1.423635252	1.187554514
**ZP3**	8.238331579	7.794092627	0.024350326	8.238331579	7.57853913	0.008767957	ECM_GLYCOPROTEINS	1.056996879	1.087060638
**ZP4**	0.940426316	0.359800355	2.55E-05	0.940426316	0.032817391	0.001991142	ECM_GLYCOPROTEINS	2.613744826	28.65633978
**ZPLD1**	3.399714035	2.282818763	7.36E-05	3.399714035	1.582713043	0.000831966	ECM_GLYCOPROTEINS	1.489261474	2.148029328

Supplemental Table 5. Matrisome signatures of different clusters

Digestive cluster		Lung cluster		Brain cluster		Ovary-uterine cluster		Hepato-biliary		Kidney cluster	
Gene	Combined fold change	Gene	Combined fold change	Gene	Combined fold change	Gene	Combined fold change	Gene	Combined fold change	Gene	Combined fold change
**CST4**	5,816412831	CST4	10,37565221	TGM5	2,069935439	LRG1	14,55199482	TINAG	7,303781726	CTSD	1,073098482
**FGF19**	4,170582035	CSTL1	9,67495932	TIMP4	1,868066145	HAPLN1	8,545151079	REG1A	2,963340304	LAMA5	1,044841559
**MMP8**	3,443192746	FGF19	8,983472648	IGFBP1	1,791302152	ARTN	7,358526882	MUC13	1,859517497		
**CSTL1**	2,977192129	CST1	2,649905721	C1QL1	1,673409478	FGF3	6,527080327	TGM3	1,769397839		
**CST1**	2,691668639	SERPINB4	2,487778839	MASP1	1,585600468	AMH	4,684480489	CCL20	1,408857744		
**CSF2**	2,400425804	ANXA10	2,309582755	GPC2	1,482765269	CSTL1	4,00200292	VWA7	1,164017634		
**COL10A1**	2,26642653	ZPLD1	2,223979297	ADAMTS6	1,461700642	MMP11	3,578267027	ADAMTS10	1,1328242		
**MMP3**	1,946712885	EPYC	2,213715336	CRISPLD1	1,353343022	HYAL3	3,458728155	PLOD3	1,068117684		
**WNT2**	1,853459415	MMP13	2,182630583	SRPX	1,340578953	MFAP2	3,376239287				
**CXCL5**	1,646579182	IBSP	2,142987629	NTN1	1,335922689	MUC16	2,572531902				
**MMP7**	1,643331075	PRSS1	2,115572254	FGF11	1,27870529	RSPO4	2,452951258				
**SERPINB5**	1,623642153	MMP12	1,81380209	C1QTNF5	1,230127061	SEMA4D	2,33825582				
**MMP10**	1,604978635	PRSS2	1,800954007	LOXL3	1,22321027	IGFBP2	2,008653858				
**CXCL3**	1,58732163	MMP10	1,726364082	TNC	1,214685107	MUC1	1,785030875				
**MMP1**	1,534017938	COL11A1	1,715666136	EFEMP1	1,196853558	MUC5AC	1,723016725				
**CCL20**	1,532803253	CCL7	1,679685989	ANGPTL2	1,1946299	CILP2	1,69371656				
**CXCL6**	1,478013781	MMP1	1,637823764	ELN	1,183893823	LAMC2	1,660916509				
**CXCL1**	1,475853185	MMP8	1,580519716	VCAN	1,170901467	ANXA3	1,61296351				
**ACAN**	1,474337055	COL10A1	1,544116239	SPARC	1,170833154	SLPI	1,577217614				
**CXCL8**	1,378112685	CCL26	1,516960294	C1QTNF1	1,15460882	COL18A1	1,526954047				
**CXCL11**	1,338940378	CXCL13	1,507504294	MATN3	1,150622717	AGRN	1,50713923				
**ADAMTS12**	1,315657621	GREM1	1,453197639	ADAMTS9	1,144040309	LAMA5	1,500750601				
**MMP11**	1,3037066	COL17A1	1,451408238	MATN2	1,143677327	WNT10A	1,494924626				
**ADAMTS14**	1,244929158	WNT16	1,446531275	WNT5A	1,13240246	MMP15	1,425556411				
**CTSV**	1,236292597	MMP11	1,432887947	CTSO	1,096951535	MUC20	1,421945349				
**F12**	1,217448747	CILP2	1,403329906	CSF1	1,094019679	BMP3	1,388598037				
**LAMC2**	1,217208795	VWDE	1,370886463	ADAM17	1,067951971	SEMA3F	1,383351863				
**PODNL1**	1,188236036	CST2	1,360548433	A2M	1,065072484	KAZALD1	1,340811047				
**ADAMTS2**	1,165985642	COCH	1,356433261	PLXNA3	1,056452449	ST14	1,324738441				
**MMP9**	1,157404703	ADAMDEC1	1,3298259			PLXNB2	1,305198201				
**COL12A1**	1,142748876	CTHRC1	1,310976114			EMID1	1,259146668				
**TGFBI**	1,140801814	SPP1	1,284215089			MUC4	1,219212887				
**COL1A1**	1,137570347	WNT10B	1,272047636			ESM1	1,210342903				
**LOXL2**	1,122603336	COL24A1	1,251770579			HAPLN3	1,206810036				
**COL5A2**	1,120472117	PLXNB3	1,249061648			ZP3	1,199883988				
**COL5A1**	1,118609537	ADAM12	1,242060984			CXCL3	1,192168622				
**COL1A2**	1,086289128	ADAMTS14	1,235560638			LGALS9	1,178857422				
**COL4A1**	1,083935558	PODNL1	1,232095289			COL9A2	1,166568295				
**PLOD3**	1,083291096	WISP1	1,217175351			FRAS1	1,144805213				

**SPON2**	1,079291706	MMP9	1,20089387			LOXL1	1,131899739				
		EGLN3	1,19337969			CCL28	1,126049193				
		SRPX2	1,193212449			VWA1	1,124658555				
		P4HA3	1,192060312			NGLY1	1,097286299				
		MMP7	1,177534434			SEMA4F	1,022703035				
		WNT10A	1,176247712								
		FNDC1	1,175434386								
		ADAMTS12	1,17205896								
		LAMB3	1,170648769								
		LGI2	1,170625615								
		PLAU	1,166122499								
		WNT3	1,1661008								
		CILP	1,165217713								
		MFAP2	1,156085096								
		MUC20	1,155164452								
		POSTN	1,151044532								
		ADAM28	1,149478943								
		THBS2	1,148757589								
		SULF1	1,142460507								
		C1QTNF6	1,139152338								
		ADAMTSL5	1,135466866								
		SDC1	1,128634632								
		COL1A1	1,128165322								
		COL3A1	1,12672845								
		IGFBP3	1,115673357								
		SEMA4B	1,114755066								
		HTRA3	1,114275076								
		COL5A2	1,114219404								
		ST14	1,114143251								
		COL5A1	1,113021104								
		HAPLN3	1,111943061								
		CXCL10	1,105090921								
		MXRA5	1,095321392								
		PLOD2	1,094946491								
		WNT5B	1,092041069								
		TNC	1,087190617								
		LOXL1	1,082216238								
		HPSE	1,077400305								
		VCAN	1,07347391								
		P4HA1	1,070209738								
		COL1A2	1,070005472								
		CTSK	1,063231259								
		MMP14	1,063196306								
		SERPINH1	1,061475747								
		CRELD2	1,053657911								
		PLAT	1,051149615								

		CSTB	1,049679595								
		SEMA4C	1,048932589								
		LMAN1	1,02849176								
		AGRN	1,026383196								

Combined fold change is the average of total fold changes in each cohort. Each total fold change is the *per-gene* average of the fold change difference in the tumor of interest *vs.* respective healthy donors and rest of the Pan-Cancer cohort.

Supplemental Table 6. Modules of matrisome expression

Module	ACC	BLCA	BRCA	CESC	CHOL	COAD	ESCA	GBM	HNSC	KICH	KIRC	KIRP	LAML	LGG	LIHC	LUAD	LUSC	MESO	PAAD	PCPG	PRAD	READ	SARC	STAD	TGCT	THCA	UCEC	UCS
ARID3A::AGT	0	0	0	0	0	1	0	0	0	0	0	0	0	0	0	0	0	0	0	0	0	0	0	0	0	0	0	0
ARID3A::WNT5A	0	0	0	0	0	0	0	0	0	0	0	0	0	0	0	0	0	0	0	0	0	0	0	0	0	1	0	0
ATF1::ANGPT1	0	0	0	0	0	0	0	0	0	0	0	0	0	0	0	0	0	0	0	0	0	0	0	0	1	0	0	0
ATF1::POSTN	0	0	0	0	0	1	0	0	0	0	0	0	0	0	0	0	0	0	0	0	0	0	0	0	0	0	0	0
ATF1::PRG4	0	0	0	0	0	0	0	0	0	0	0	0	1	0	0	0	0	0	0	0	0	0	0	0	0	0	0	0
ATF3::CCL3	0	0	0	0	0	0	0	0	0	0	0	0	0	0	0	0	0	1	0	0	0	0	0	0	1	0	0	0
ATF3::CXCL2	0	0	0	0	0	0	0	0	1	0	1	0	1	0	0	0	0	0	0	0	0	0	0	0	0	0	1	0
ATF3::SERPINE1	0	0	0	0	0	0	1	1	0	0	1	0	0	0	0	0	0	0	0	0	0	0	0	0	0	0	0	0
BACH1::A2M	0	0	0	0	0	0	0	0	0	0	0	0	0	1	0	0	0	0	0	0	0	0	0	0	0	0	0	0
BACH1::COL8A2	0	0	0	0	0	0	0	0	0	0	0	0	0	0	0	0	0	0	0	0	0	0	0	0	0	1	0	0
BACH1::COLEC12	0	0	0	0	0	0	0	0	0	0	0	0	0	0	0	0	0	0	0	0	1	0	0	0	0	0	0	0
BACH1::CRLF3	0	1	0	0	0	0	0	0	0	0	0	0	0	0	0	0	0	0	0	0	0	0	0	0	0	0	0	0
BACH1::CXCL5	0	0	0	0	0	0	0	0	0	0	0	0	0	0	0	0	0	0	0	0	0	0	0	0	0	0	1	0
BACH1::CXCL8	0	0	0	1	0	0	0	0	0	0	0	0	0	0	0	0	0	0	0	0	0	0	0	0	0	0	0	0
BACH1::EGF	0	0	0	0	0	0	0	0	0	0	0	0	0	0	0	0	0	0	0	0	1	0	0	0	0	0	0	0
BACH1::EGLN2	0	0	1	0	0	0	0	0	0	0	0	0	0	0	0	0	0	0	0	0	0	0	0	0	0	0	0	0
BACH1::LAMC1	0	0	0	0	0	0	0	0	0	0	0	1	0	0	0	0	0	0	0	0	0	0	0	0	0	0	0	0
BACH1::LAMC2	0	0	0	0	0	1	0	0	0	0	0	0	0	0	0	0	1	0	0	0	0	0	0	1	0	0	0	0
BACH1::SERPINB8	0	0	0	0	0	0	0	0	0	0	0	0	0	0	0	0	0	0	0	0	0	0	0	1	0	0	0	0
BCL3::CCL2	0	0	0	0	0	0	0	0	0	0	0	0	0	0	0	0	0	1	0	0	0	0	0	0	0	0	0	0
BCL3::SEMA4B	0	0	0	1	0	0	0	0	0	0	0	0	0	0	0	0	0	0	0	0	0	0	0	0	0	0	0	0
BCL3::WNT7B	0	0	1	0	0	0	0	0	0	0	0	0	0	0	0	0	1	0	0	0	0	0	0	0	0	0	0	0
NFAT5::CLEC2D	0	0	0	0	0	0	0	0	0	0	0	0	0	0	0	1	0	0	0	0	0	0	0	0	0	0	0	0
NFAT5::LAMA3	0	0	0	0	0	0	0	0	0	0	0	0	0	0	0	0	0	0	1	0	0	0	0	0	0	0	0	0
NFAT5::SEMA3C	0	0	0	0	0	0	0	0	1	0	0	0	0	0	0	0	0	0	0	0	0	0	0	0	0	0	0	0
NFATC1::SRGN	0	0	0	0	0	0	0	0	0	0	1	0	0	0	0	0	0	0	0	0	0	0	0	0	0	0	0	0
NFE2::ADAMTS14	0	0	0	0	0	0	0	0	0	0	0	0	0	0	0	0	0	0	0	0	0	0	0	1	0	0	1	0
NFE2::BMP4	0	0	0	0	0	1	0	0	0	0	0	0	0	0	0	0	0	0	0	0	0	0	0	0	0	0	0	0
NFE2::CXCL1	0	0	0	1	0	0	0	0	1	0	0	0	0	0	0	0	0	0	0	0	0	0	0	0	0	0	0	0
NFE2::LAMA3	0	0	0	0	0	0	0	0	0	0	0	1	0	0	0	0	0	0	0	0	0	0	0	0	0	0	0	0
NFE2::LAMA5	0	0	0	1	0	0	0	0	0	0	0	0	0	0	0	0	0	0	0	0	0	0	0	0	0	0	0	0
NFE2::OGFOD2	0	0	0	0	0	0	0	0	0	0	0	0	1	0	0	0	0	0	0	0	0	0	0	0	0	0	0	0
NFE2::PODNL1	0	0	0	0	0	0	0	0	1	0	0	0	0	0	0	0	0	0	0	0	0	0	0	0	0	0	0	0
NFE2::SEMA3F	0	0	0	0	0	0	0	0	0	0	0	0	0	0	0	0	0	0	0	0	0	0	0	0	0	0	1	0
NFE2L1::GPC1	0	0	0	0	0	0	1	0	0	0	0	0	0	0	0	0	0	0	0	0	0	0	0	0	0	0	0	0
NFE2L1::SEMA4C	0	0	0	0	0	0	0	0	0	0	0	0	0	0	0	0	0	0	0	0	0	0	0	0	1	0	0	0
NFE2L1::SEMA4F	0	0	0	0	0	0	0	0	0	0	0	0	0	0	0	0	1	0	0	0	0	0	0	0	0	0	0	0
NFE2L1::THBS3	0	0	0	0	0	0	0	0	0	0	0	0	1	0	0	0	0	0	0	0	0	0	0	0	0	0	0	0
NFE2L2::HPSE	0	0	0	0	0	0	0	0	0	0	0	0	0	0	0	0	1	0	0	0	0	0	0	0	0	0	0	0
NFIB::C1QTNF5	0	0	0	0	0	0	0	0	0	0	0	1	0	0	0	0	0	0	0	0	0	0	0	0	0	0	0	0
NFIB::EGFLAM	0	0	0	0	0	0	0	0	0	0	0	0	0	0	0	0	0	1	0	0	0	0	0	0	0	0	0	0

**NFIB::FBLN5**	0	0	0	0	0	0	0	0	0	0	0	0	0	0	0	0	0	0	0	0	0	0	0	0	1	0	0	0
**NFIB::LTBP4**	0	0	0	0	0	0	0	0	0	0	0	0	0	0	0	0	0	0	0	0	0	0	0	0	1	0	0	0
**NFIB::PRG4**	0	0	0	0	0	0	0	0	0	0	0	0	1	0	0	0	0	0	0	0	0	0	0	0	0	0	0	0
**NFIL3::CSF3**	0	0	0	0	0	1	0	0	0	0	0	0	0	0	0	0	0	0	0	0	0	0	0	0	0	0	0	0
**NFKB1::CCL2**	0	0	0	0	0	0	0	0	0	0	0	0	0	0	0	0	0	1	0	0	0	0	0	0	0	0	0	0
**NFKB1::CSF1**	0	0	0	0	0	0	0	0	0	0	0	0	0	1	0	0	0	0	0	0	0	0	0	0	0	0	0	0
**NFKB1::HPSE**	0	0	0	1	0	0	0	0	0	0	0	0	0	0	0	0	0	0	0	0	0	0	0	0	0	0	0	0
**NFKB1::LTBP3**	0	0	0	0	0	0	0	0	0	0	0	0	0	1	0	0	0	0	0	0	0	0	0	0	0	1	0	0
**NFKB1::MMP13**	0	1	0	0	0	0	0	0	0	0	0	0	0	0	0	0	0	0	0	0	0	0	0	0	0	0	0	0
**NFKB1::MMP14**	0	1	0	0	0	0	0	0	0	0	0	0	0	0	0	0	0	0	0	0	0	0	0	0	0	0	0	0
**NFKB1::PLXNB2**	0	0	0	0	0	0	0	0	0	0	0	0	0	0	0	0	0	0	0	0	0	0	0	1	0	0	0	0
**NFKB1::SEMA4B**	0	0	0	0	0	0	0	0	1	0	0	0	0	0	0	0	0	0	0	0	0	0	0	0	0	0	0	0
**NFKB1::SERPINB8**	0	0	0	0	0	0	0	0	0	0	0	0	0	0	0	0	0	1	0	0	0	0	0	0	0	1	0	0
**NFKB1::TNC**	0	0	0	0	0	0	0	0	1	0	0	0	0	0	0	1	0	0	0	0	0	0	0	0	0	0	0	0
**NFKB2::ANGPTL4**	0	0	0	0	0	0	0	0	0	0	1	0	0	0	0	0	0	0	0	0	0	0	0	0	0	0	0	0
**NFKB2::C1QTNF1**	0	0	0	0	0	0	0	0	1	0	0	0	0	0	0	0	0	0	0	0	0	0	0	0	0	0	0	0
**NFKB2::CCL20**	0	0	0	0	0	0	0	0	1	0	0	0	0	0	0	0	0	0	0	0	0	0	0	0	0	0	0	0
**NFKB2::CCL5**	0	0	0	0	0	0	0	0	1	0	0	0	0	0	0	0	0	0	0	0	0	0	0	0	0	0	0	0
**NFKB2::CCL7**	0	0	0	0	0	0	0	0	0	0	0	0	0	0	0	0	0	0	0	0	0	0	0	0	0	0	1	0
**NFKB2::CSF1**	0	0	0	0	0	0	0	1	0	0	0	0	0	1	0	0	0	0	0	0	0	0	0	0	0	0	0	0
**NFKB2::CSTB**	0	0	0	0	0	0	0	0	0	0	0	0	0	0	0	1	0	0	0	0	0	0	0	0	0	0	1	0
**NFKB2::EBI3**	0	0	0	0	0	0	0	0	0	0	1	1	0	0	0	0	0	0	0	0	0	0	0	0	0	1	0	0
**NFKB2::HAPLN3**	0	0	0	0	0	0	1	0	1	0	0	0	0	0	0	1	1	0	0	0	0	0	1	0	0	0	0	0
**NFKB2::LAMA5**	0	0	0	0	0	0	0	0	1	0	0	0	0	0	0	0	0	0	0	0	0	0	0	0	0	0	0	0
**NFKB2::LAMC2**	0	0	0	0	0	1	0	0	0	0	0	0	0	0	0	0	0	0	0	0	0	0	0	0	0	0	0	0
**NFKB2::LGALS1**	0	0	0	0	0	0	0	0	1	0	0	0	0	0	0	0	0	0	0	0	0	0	0	0	0	0	0	0
**NFKB2::MMP14**	0	0	0	0	0	0	0	0	0	0	0	0	0	0	0	0	0	0	0	0	0	0	0	1	0	0	0	0
**NFKB2::MMP19**	0	0	0	0	0	0	0	0	1	0	0	0	0	0	0	0	0	0	0	0	0	0	0	0	0	0	0	0
**NFKB2::P4HA1**	0	0	0	0	0	0	0	0	0	0	1	0	0	0	0	0	0	0	0	0	0	0	0	0	0	0	0	0
**NFYA::CTSV**	0	0	0	0	0	1	0	0	0	0	0	0	0	0	0	0	0	0	0	0	0	0	0	0	0	0	0	0
**NFYA::HAPLN1**	0	0	0	0	0	0	0	0	0	0	0	0	0	0	0	0	0	0	0	0	0	0	0	0	0	0	1	0
**NKX2-1::COL13A1**	0	0	0	0	0	0	0	0	0	0	0	0	0	0	0	0	0	0	0	0	0	0	0	0	0	1	0	0
**NKX2-8::EREG**	0	0	0	0	0	0	0	0	0	0	0	0	0	0	0	0	0	0	1	0	0	0	0	0	0	0	0	0
**NKX2-8::LAMA5**	0	0	0	0	0	0	0	0	0	0	0	0	0	0	0	0	0	0	0	0	0	0	0	0	0	0	1	0
**NKX2-8::P4HA2**	0	0	0	0	0	0	0	0	0	0	0	0	0	0	0	0	0	0	1	0	0	0	0	0	0	0	0	0
**NKX3-1::ADAM32**	1	0	0	0	0	0	0	0	0	0	0	0	0	0	0	0	0	0	0	0	0	0	0	0	0	0	0	0
**NKX3-1::ANXA3**	0	0	0	0	0	0	0	0	0	0	0	0	0	0	0	0	0	0	0	0	1	0	0	0	0	0	0	0
**NKX3-1::COLEC12**	0	0	0	0	0	0	0	0	0	0	0	0	0	0	0	0	0	0	0	0	1	0	0	0	0	0	0	0
**NKX3-1::SPOCK1**	0	0	0	0	0	0	0	0	0	0	0	0	0	0	0	0	0	0	0	0	1	0	0	0	0	0	0	0
**NKX3-2::GAS6**	0	0	0	0	0	0	0	0	0	0	0	0	0	0	0	0	0	0	0	0	0	0	0	0	0	1	0	0
**NKX6-1::FBN2**	0	1	0	0	0	0	0	0	0	0	0	0	0	0	0	0	0	0	0	0	0	0	0	0	0	0	0	0
**NKX6-1::LOXL2**	0	0	0	0	0	0	0	0	0	0	0	0	0	0	0	0	0	0	0	0	0	0	1	0	0	0	0	0
**NKX6-1::MFAP2**	0	1	0	0	0	0	0	0	0	0	0	0	0	0	0	1	0	0	0	0	0	0	0	0	0	0	0	0
**NKX6-1::OGFOD1**	0	0	0	0	0	0	0	0	0	0	0	0	0	0	0	0	1	0	0	0	0	0	0	0	0	0	0	0
**NKX6-1::WNT5A**	0	0	0	0	0	0	0	0	0	0	0	0	0	0	0	0	1	0	0	0	0	0	0	0	0	0	0	0
**NKX6-1::XCL1**	0	1	0	0	0	0	0	0	0	0	0	0	0	0	0	0	0	0	0	0	0	0	0	0	0	0	0	0

**NR2C2::DDX26B**	0	0	0	0	0	0	0	0	0	0	0	0	1	0	0	0	0	0	0	0	0	0	0	0	0	0	0	0
**NR2F1::BGN**	0	0	0	0	0	0	0	0	1	0	1	0	0	0	0	0	0	0	0	0	0	0	0	0	0	0	0	0
**NR2F1::COL8A1**	0	0	0	0	0	0	0	0	0	0	0	0	0	0	0	0	0	0	0	0	1	0	0	0	0	1	0	0
**NR2F1::CXCL6**	0	0	0	0	0	0	0	0	0	0	0	0	0	0	0	0	0	1	0	0	0	0	0	0	0	0	0	0
**NR2F1::PLAU**	0	0	0	0	0	0	0	0	0	0	0	0	0	0	0	0	0	0	0	0	0	0	0	0	0	1	0	0
**NR2F2::LRG1**	0	0	1	0	0	0	0	0	0	0	0	0	0	0	0	0	0	0	0	0	0	0	0	0	0	0	0	0
**NR2F6::BGLAP**	0	0	0	0	0	0	0	0	0	0	0	0	0	0	0	0	0	0	0	0	1	0	0	0	0	0	0	0
**NR2F6::CILP2**	0	0	0	0	0	0	0	0	0	0	0	0	0	0	0	0	0	0	0	0	0	0	0	0	0	0	1	0
**NR2F6::CRELD2**	0	0	0	0	0	0	0	0	0	0	0	0	0	0	0	0	0	0	0	0	1	0	0	0	0	0	0	0
**NR2F6::HAPLN3**	0	0	0	0	0	0	0	0	0	0	0	0	0	0	0	0	0	0	0	0	0	0	0	0	0	0	1	0
**NR2F6::KAZALD1**	0	0	0	0	0	0	0	0	0	0	0	0	0	0	0	1	0	0	0	0	0	0	0	0	0	0	0	0
**NR2F6::LTBP3**	0	0	0	0	0	0	0	0	0	0	0	0	0	1	0	0	0	0	0	0	0	0	0	0	0	0	0	0
**NR2F6::MMP15**	0	0	0	0	0	0	0	0	0	0	0	0	0	0	0	0	0	0	0	0	0	0	0	1	0	0	0	0
**NR2F6::OGFOD2**	0	1	0	0	0	0	0	0	0	0	0	0	0	0	0	0	0	0	0	0	1	0	0	0	0	0	0	0
**NR2F6::PLXNB2**	0	1	0	0	0	0	0	0	0	0	0	0	0	0	0	0	0	0	0	0	0	0	0	0	0	0	0	0
**NR2F6::SEMA4C**	0	0	0	0	0	0	0	0	0	0	0	0	0	0	0	0	0	0	0	0	0	0	0	0	1	0	0	0
**NR2F6::SERPINH1**	0	0	0	0	0	0	0	0	0	0	0	0	0	0	0	0	0	1	0	0	0	0	1	0	0	0	0	0
**NR2F6::ST14**	0	1	0	0	0	0	0	0	0	0	0	0	0	0	0	1	0	0	0	0	1	0	0	0	0	1	0	0
**NR3C1::FGF2**	0	0	0	0	0	0	0	0	0	0	0	0	0	1	0	0	0	0	0	0	0	0	0	0	0	0	0	0
**NR3C1::SERPINE2**	0	0	0	0	0	0	0	0	0	0	0	0	0	0	0	0	0	0	0	1	0	0	0	0	0	0	0	0
**NR3C1::SMOC1**	0	0	0	0	0	0	0	0	0	1	0	0	0	0	0	0	0	0	0	0	0	0	0	0	0	0	0	0
**NR3C1::VCAN**	0	0	0	0	0	0	0	0	0	0	1	0	0	1	0	0	0	0	0	0	0	0	0	0	0	0	0	0
**NR3C2::MUC3A**	0	0	0	0	0	0	0	0	0	0	0	0	0	0	0	0	0	0	0	0	0	0	0	1	0	0	0	0
**NR4A2::CSF3**	0	0	0	0	0	1	0	0	0	0	0	0	0	0	0	0	0	0	0	0	0	0	0	0	0	0	0	0
**NR5A2::ANGPT2**	0	0	0	0	0	0	0	0	0	0	1	0	0	0	0	0	0	0	0	0	0	0	0	0	0	0	0	0
**NR6A1::ADAMTS14**	0	0	0	0	0	0	0	0	0	0	0	0	0	0	0	0	0	0	0	0	0	0	0	1	0	0	0	0
**NR6A1::ANXA6**	0	0	0	0	0	0	0	0	0	0	0	0	0	0	0	0	0	0	0	1	0	0	0	0	0	0	0	0
**NR6A1::BGLAP**	0	1	0	0	0	0	0	0	0	0	0	0	0	0	0	0	0	0	0	0	1	0	0	0	0	0	0	0
**NR6A1::MMP15**	0	0	0	0	0	0	0	0	0	0	0	0	0	0	0	0	0	0	0	0	0	0	0	0	1	0	0	0
**NR6A1::PRSS3**	0	0	0	0	0	0	0	0	0	0	0	0	0	0	0	0	0	0	0	1	0	0	0	0	0	0	0	0
**NRL::CSTB**	0	0	0	0	0	0	0	0	0	0	0	0	0	0	0	1	0	0	0	0	0	0	0	0	0	0	0	0
**OSR1::F10**	0	0	0	0	0	0	0	0	0	0	0	0	0	0	0	0	0	0	0	0	0	0	0	0	1	0	0	0
**OSR1::SEMA5A**	0	0	0	0	0	0	0	0	0	0	0	0	0	0	0	0	0	1	0	0	0	0	0	0	0	0	0	0
**OSR1::SVEP1**	0	0	0	0	0	0	0	0	0	0	0	0	0	0	0	0	0	1	0	0	0	0	0	0	0	0	0	0
**OTX1::ADAMTSL5**	0	0	0	0	0	1	0	0	0	0	0	0	0	0	0	0	0	0	0	0	0	0	0	0	0	0	0	0
**OTX1::CTSV**	0	0	0	0	0	0	0	0	0	0	0	0	0	0	0	0	0	0	0	0	0	0	0	1	0	0	0	0
**OTX1::MMP11**	0	0	0	0	0	0	1	0	0	0	0	0	0	0	0	0	0	0	0	0	0	0	0	0	0	0	0	0
**OTX1::MMP7**	0	0	0	0	0	1	0	0	0	0	0	0	0	0	0	0	0	0	0	0	0	0	0	0	0	0	0	0
**OTX1::SPON2**	0	0	0	0	0	0	0	0	0	0	0	0	0	0	0	0	0	0	0	0	1	0	0	0	0	0	0	0
**PATZ1::ADAM15**	0	0	1	0	0	0	0	0	0	0	0	0	0	0	0	0	0	0	0	0	0	0	0	0	0	0	0	0
**PAX2::CX3CL1**	0	0	0	0	0	0	0	0	0	0	1	1	0	0	0	0	0	0	0	0	0	0	0	0	0	0	0	0
**PAX5::C1QTNF6**	0	0	0	0	0	0	0	0	1	0	0	0	0	0	0	0	0	0	0	0	0	0	0	0	0	0	0	0
**PAX5::HTRA4**	0	0	1	0	0	0	0	0	0	0	0	0	0	0	0	0	0	0	0	0	0	0	0	0	0	0	0	0
**PAX5::SERPINA9**	0	0	0	0	0	0	0	0	1	0	0	0	0	0	0	0	0	0	0	0	0	0	0	0	0	0	0	0
**PAX6::MASP1**	0	0	0	0	0	0	0	0	0	0	0	0	0	1	0	0	0	0	0	0	0	0	0	0	0	0	0	0
**PAX8::ANXA3**	0	0	0	0	0	0	0	0	0	0	0	0	0	0	0	0	0	0	0	0	0	0	0	0	0	0	1	0

**PAX8::COL27A1**	0	0	0	0	0	0	0	0	0	0	0	1	0	0	0	0	0	0	0	0	0	0	0	0	0	0	0	0
**PAX8::FGF18**	0	0	0	0	0	0	0	0	0	0	0	0	0	0	0	0	0	0	0	0	0	0	0	0	0	0	1	0
**PBX1::COL4A6**	0	0	0	0	0	0	0	1	0	0	0	0	0	0	0	0	0	0	0	0	0	0	0	0	0	0	0	0
**PBX1::CTHRC1**	0	0	0	0	0	0	0	0	0	0	0	0	0	0	0	0	0	0	0	0	0	0	1	0	0	0	0	0
**PBX1::LAMA4**	0	0	0	0	0	0	0	0	0	0	0	0	0	0	0	0	0	1	0	0	0	0	1	0	0	0	0	0
**PBX1::WNT2B**	0	0	0	0	0	0	0	0	0	0	0	0	0	0	0	0	0	1	0	0	0	0	0	0	0	0	0	0
**PBX1::XCL1**	0	0	0	0	0	0	0	0	0	0	0	0	0	0	0	0	1	0	0	0	0	0	0	0	0	0	0	0
**PDX1::ANXA4**	0	0	0	0	0	0	0	0	0	0	0	0	0	0	0	0	0	0	0	0	0	0	0	1	0	0	0	0
**PDX1::BMP4**	0	0	0	0	0	1	0	0	0	0	0	0	0	0	0	0	0	0	0	0	0	0	0	0	0	0	0	0
**PDX1::COL11A2**	0	0	0	0	0	0	0	0	0	0	0	0	0	0	0	0	0	1	0	0	0	0	0	0	0	0	0	0
**PDX1::CXCL3**	0	0	0	0	0	0	1	0	0	0	0	0	0	0	0	0	0	0	0	0	0	0	0	0	0	0	0	0
**PDX1::CXCL5**	0	0	0	0	0	0	0	0	0	0	0	0	0	0	0	0	0	0	0	0	0	0	0	1	0	0	0	0
**PDX1::FGF13**	0	0	0	0	0	0	0	0	0	0	0	0	0	0	0	0	0	0	0	0	1	0	0	0	0	0	0	0
**PDX1::MMP15**	0	0	0	0	0	0	0	0	0	0	0	0	0	0	0	0	0	0	0	0	0	0	0	1	0	0	0	0
**PDX1::MUC3A**	0	0	0	0	0	0	0	0	0	0	0	0	0	0	0	0	0	0	0	0	0	0	0	1	0	0	0	0
**PDX1::NTNG1**	0	0	0	0	0	0	0	0	0	0	0	0	0	0	0	0	0	0	0	0	0	0	0	0	0	0	0	1
**PDX1::ST14**	0	0	0	0	0	0	1	0	0	0	0	0	0	0	0	0	0	0	0	0	0	0	0	0	0	0	0	0
**PDX1::VWA9**	0	1	0	0	0	0	0	0	0	0	0	0	0	0	0	0	0	0	0	0	0	0	0	0	0	0	0	0
**PGR::VCAN**	0	0	0	0	0	0	0	0	0	0	0	0	0	0	0	0	1	0	0	0	0	0	0	0	0	0	0	0
**PITX1::ANXA10**	0	0	0	0	0	0	0	0	0	0	0	0	0	0	0	1	1	0	0	0	0	0	0	0	0	0	0	0
**PITX1::P4HA2**	0	0	0	0	0	0	0	0	0	0	0	0	0	0	0	0	0	0	0	0	0	0	0	0	0	0	0	1
**PITX2::CTSV**	0	0	0	0	0	0	0	0	0	0	0	0	0	0	0	0	0	0	0	0	0	1	0	0	0	0	0	0
**PITX2::FGB**	0	0	0	0	0	0	0	0	0	0	0	0	0	0	0	1	0	0	0	0	0	0	0	0	0	0	0	0
**PITX2::WNT3A**	0	0	0	0	0	0	1	0	0	0	0	0	0	0	0	0	0	0	0	0	0	0	0	0	0	0	0	0
**PITX3::COL19A1**	0	0	0	0	0	0	0	0	0	0	0	0	0	0	0	0	0	0	0	0	0	0	0	0	0	0	0	1
**PITX3::MMP9**	0	0	0	1	0	0	0	0	0	0	0	0	0	0	0	0	0	0	0	0	0	0	0	0	0	0	0	0
**PITX3::SEMA6C**	0	0	0	0	0	0	0	0	0	0	0	0	0	0	0	0	0	0	0	0	0	0	0	0	0	0	0	1
**PKNOX2::COL11A2**	0	0	0	0	0	0	0	0	0	0	0	0	0	0	0	0	0	1	0	0	0	0	0	0	0	0	0	0
**PKNOX2::FMOD**	0	0	0	0	0	0	0	0	0	0	0	0	0	0	0	0	0	0	0	0	1	0	0	0	0	0	0	0
**PLAG1::ADAMTS19**	0	0	0	0	0	0	0	0	0	0	0	0	0	0	0	0	0	0	0	1	0	0	0	0	0	0	0	0
**PLAG1::CRLF1**	0	0	0	0	0	0	0	0	0	0	0	0	0	0	0	1	0	0	0	0	0	0	0	0	0	0	0	0
**PLAG1::GAS6**	0	0	0	0	0	0	0	0	0	1	0	0	0	0	0	0	0	0	0	0	0	0	0	0	0	0	0	0
**PLAG1::LOXL3**	0	0	0	0	0	0	0	0	0	0	0	0	0	0	0	0	0	0	0	0	0	0	0	0	0	0	0	1
**PLAG1::SLPI**	0	0	0	0	0	0	0	0	0	0	0	1	0	0	0	0	0	0	0	0	0	0	0	0	0	0	0	0
**PLAG1::WNT5A**	0	0	0	0	0	0	0	0	0	0	0	0	0	0	0	0	0	0	0	0	0	0	0	0	0	1	0	0
**POU2F1::FAM20B**	0	0	0	0	0	0	0	0	0	0	0	0	0	0	0	0	1	0	0	0	0	0	0	0	0	0	0	0
**POU2F1::LGALS8**	0	0	0	0	0	0	0	0	0	0	1	0	0	0	0	0	0	0	0	0	0	0	0	0	0	0	0	0
**POU2F1::SEMA4D**	0	0	0	0	0	0	0	0	0	0	0	0	0	0	0	0	0	0	0	0	0	0	0	0	0	0	1	0
**POU2F2::CCL22**	0	0	0	0	0	0	0	0	0	0	0	0	0	0	0	0	0	0	0	0	0	0	0	1	0	0	0	0
**POU2F2::DDX26B**	0	0	0	0	0	0	0	0	0	0	0	0	0	0	0	0	1	0	0	0	0	0	0	0	0	0	0	0
**POU2F2::NTNG2**	0	0	0	0	0	0	0	0	0	0	0	0	0	0	0	0	0	0	0	0	0	0	0	1	0	0	0	0
**POU2F2::SERPINB8**	0	0	0	0	0	0	0	0	0	0	0	0	1	0	0	0	0	0	0	0	0	0	0	0	0	0	0	0
**POU2F3::VWA5A**	0	0	0	0	0	0	0	0	0	0	0	0	0	0	0	0	0	0	0	0	0	0	0	0	0	1	0	0
**POU3F1::C1QL1**	0	0	0	0	0	0	0	1	0	0	0	0	0	0	0	0	0	0	0	0	0	0	0	0	0	0	0	0
**POU3F1::CLEC7A**	0	0	0	1	0	0	1	0	1	0	0	0	0	0	0	0	0	0	0	0	0	0	0	1	0	0	0	0
**POU3F1::CRLF3**	0	0	0	0	0	0	0	0	0	1	0	0	0	0	0	0	0	0	0	0	0	0	0	0	0	0	0	0

**POU3F1::CSF1**	0	0	0	0	0	0	0	0	0	1	0	0	0	0	0	0	0	0	0	0	0	0	0	0	0	0	0	0
**POU3F1::CSTA**	0	0	0	1	0	0	0	0	0	0	0	0	0	0	0	0	0	0	0	0	0	0	0	0	0	0	0	0
**POU3F1::SDC1**	0	1	0	0	0	0	0	0	0	0	0	0	0	0	0	0	0	0	0	0	0	0	0	0	0	0	0	0
**POU3F1::SERPINA9**	0	0	0	0	0	0	0	0	1	0	0	0	0	0	0	0	0	0	0	0	0	0	0	0	0	0	0	0
**POU3F1::SERPINB9**	0	0	0	0	0	0	0	0	0	0	0	0	0	0	0	0	0	0	0	0	0	0	0	1	0	0	0	0
**POU3F1::SERPINE2**	0	0	0	0	0	0	0	0	0	0	0	0	0	0	0	0	1	0	0	0	0	0	0	0	0	0	0	0
**POU3F1::THBS2**	0	0	0	0	0	0	0	0	1	0	0	0	0	0	0	0	0	0	0	0	0	0	0	0	0	0	0	0
**POU3F1::TLL2**	0	0	0	0	0	0	0	0	0	0	0	0	0	0	0	0	1	0	0	0	0	0	0	0	0	0	0	0
**POU3F1::TNC**	0	0	0	0	0	0	0	0	1	0	0	0	0	0	0	0	1	0	0	0	0	0	0	0	0	0	0	0
**POU3F2::FGF13**	0	0	0	0	0	0	0	0	0	0	0	0	0	0	0	0	0	0	0	0	0	0	0	0	1	0	0	0
**POU3F2::FGF20**	0	0	0	0	0	0	0	0	0	0	0	0	0	0	0	0	0	0	0	0	0	0	0	1	0	0	0	0
**POU3F2::SEMA3A**	0	0	0	0	0	0	0	0	0	0	0	0	0	0	0	1	0	0	0	0	0	0	0	0	0	0	0	0
**POU3F3::CX3CL1**	0	0	0	0	0	0	0	0	0	0	0	1	0	0	0	0	0	0	0	0	0	0	0	0	0	0	0	0
**POU3F3::FGF1**	0	0	0	0	0	0	0	0	0	0	0	0	0	0	0	0	0	0	0	1	0	0	0	0	0	0	0	0
**POU3F3::IGFBP2**	0	0	0	0	0	0	0	0	0	0	0	0	0	0	0	0	0	0	0	0	0	0	0	0	0	0	1	0
**POU3F3::PLXNC1**	0	0	0	0	0	0	0	0	0	0	0	0	0	0	0	0	0	0	0	1	0	0	0	0	0	0	0	0
**POU3F3::PXDN**	0	0	0	0	0	0	0	0	0	0	0	0	0	0	0	0	0	0	0	0	0	0	1	0	0	0	0	0
**POU3F3::SPOCK2**	0	0	0	0	0	0	0	0	0	0	0	0	0	0	0	0	0	0	0	1	0	0	0	0	0	0	0	0
**POU3F3::SULF2**	0	0	0	0	0	0	0	0	0	0	0	0	0	0	0	0	0	1	0	0	0	0	0	0	0	0	0	0
**POU3F4::ADAMTS17**	0	0	0	0	0	0	0	0	0	0	0	0	0	0	0	0	1	0	0	0	0	0	0	0	0	0	0	0
**POU4F2::NTNG2**	0	0	0	0	0	0	0	0	0	0	0	0	0	0	0	0	0	0	0	0	0	0	0	1	0	0	0	0
**POU5F1::FGF4**	0	0	0	0	0	0	0	0	0	0	0	0	0	0	0	0	0	0	0	0	0	0	0	0	1	0	0	0
**POU6F2::ADAMTS17**	0	0	0	0	0	0	0	0	0	0	0	0	0	0	0	0	1	0	0	0	0	0	0	0	0	0	0	0
**POU6F2::IGFBP2**	0	0	0	0	0	0	0	0	0	0	0	0	0	0	0	0	1	0	0	0	0	0	0	0	0	0	0	0
**POU6F2::NELL2**	0	0	0	0	0	0	0	0	1	0	0	0	0	0	0	0	1	0	0	0	0	0	0	0	0	0	0	0
**POU6F2::XCL1**	0	0	0	0	0	0	0	0	0	0	0	0	0	0	0	0	0	0	0	0	0	0	0	0	0	0	0	0
**PPARA::PAPLN**	0	0	0	0	0	0	0	0	0	0	0	0	0	0	0	1	0	0	0	0	0	0	0	0	0	0	0	0
**PPARA::PLXNB2**	0	0	0	1	0	0	0	0	1	0	0	0	0	0	0	0	0	0	0	0	0	0	0	0	0	0	0	0
**PPARA::SEMA4A**	0	0	0	1	0	0	0	0	0	0	0	0	0	0	0	0	0	0	0	0	0	0	0	0	0	0	0	0
**PPARG::ADAMTS7**	0	1	0	0	0	0	0	0	0	0	0	0	0	0	0	0	0	0	0	0	0	0	0	0	0	0	0	0
**PPARG::ANGPT1**	0	0	0	0	0	0	0	0	0	1	0	0	1	0	0	0	0	0	0	0	0	0	0	0	0	0	0	0
**PPARG::CILP2**	0	1	0	0	0	0	0	0	0	0	0	0	0	0	0	0	0	0	0	0	0	0	0	0	0	0	0	0
**PPARG::HPSE**	0	0	0	0	0	0	0	1	0	0	0	0	0	0	0	0	0	0	0	0	0	0	0	0	0	0	0	0
**PPARG::IGFBP3**	0	1	0	0	0	0	0	0	0	0	0	0	0	0	0	0	0	0	0	0	0	0	0	0	0	0	0	0
**PPARG::TINAGL1**	0	1	0	0	0	0	0	0	0	0	0	0	0	0	0	0	0	0	0	0	0	0	0	0	0	0	0	0
**PRDM1::A2M**	0	0	0	0	0	0	0	1	0	0	0	0	0	0	0	0	0	0	0	0	0	0	0	0	0	0	0	0
**PRDM1::ADAMTS9**	0	0	0	0	0	0	0	0	0	0	0	0	0	0	0	0	0	0	0	0	0	0	0	0	0	1	0	0
**PRDM1::BMP2**	0	0	0	0	0	0	0	0	1	0	0	0	0	0	0	0	0	0	0	0	0	0	0	0	0	0	0	0
**PRDM1::CCL22**	0	0	0	1	0	0	1	0	0	0	0	0	0	0	0	0	0	0	0	0	0	0	0	0	0	0	0	0
**PRDM1::CLEC2D**	0	0	1	0	0	0	0	0	0	0	0	0	0	0	0	0	0	0	0	0	0	0	0	0	0	0	0	0
**PRDM1::CLEC7A**	0	0	1	1	0	0	0	0	0	0	0	0	0	0	0	0	0	0	0	0	0	0	0	1	0	0	0	0
**PRDM1::COL17A1**	0	0	0	1	0	0	0	0	1	0	0	0	0	0	0	0	0	0	0	0	0	0	0	0	0	0	0	0
**PRDM1::COL5A2**	0	0	0	0	0	0	0	0	0	0	0	0	0	0	0	0	1	0	0	0	0	0	0	0	0	0	0	0
**PRDM1::COL6A3**	0	0	0	0	0	0	0	0	0	0	0	0	0	0	0	1	0	0	0	0	0	0	0	0	0	0	0	0
**PRDM1::CRLF3**	0	0	0	1	0	0	0	0	0	0	0	0	0	0	0	0	0	0	0	0	0	0	0	0	0	0	0	0
**PRDM1::CST7**	0	0	0	0	0	0	0	0	0	0	0	0	0	0	0	0	0	1	0	0	0	0	0	0	0	0	0	0

**PRDM1::CXCL10**	0	0	1	0	0	0	0	0	0	0	0	0	0	0	0	0	0	0	0	0	0	0	0	0	0	0	0	0
**PRDM1::EBI3**	0	0	0	0	0	0	0	0	0	0	0	1	0	0	0	0	0	0	0	0	0	0	0	0	0	0	0	0
**PRDM1::EGFL6**	0	0	0	0	0	0	0	0	1	0	0	0	0	0	0	0	0	0	0	0	0	0	0	0	0	0	0	0
**PRDM1::FGF1**	0	0	0	0	0	0	0	0	0	0	0	0	0	0	0	0	0	0	0	0	0	0	0	0	0	1	0	0
**PRDM1::HAPLN3**	0	0	0	0	0	0	0	0	0	0	0	0	0	0	0	0	0	0	0	0	0	0	1	0	0	0	0	0
**PRDM1::HPSE**	0	0	0	0	0	0	1	0	0	0	0	0	0	0	0	0	0	0	0	0	0	0	0	0	0	0	0	0
**PRDM1::LAMA4**	0	0	0	0	0	0	0	1	0	0	1	0	0	0	0	0	0	0	0	0	0	0	0	0	0	0	0	0
**PRDM1::PAMR1**	0	0	0	0	0	0	0	0	0	0	1	0	0	0	0	0	0	0	0	0	0	0	0	0	0	0	0	0
**PRDM1::PLAU**	0	1	1	0	0	0	0	0	0	0	0	0	0	0	0	0	0	0	0	0	0	0	0	0	0	1	0	0
**PRDM1::PLXNC1**	0	0	1	0	0	0	0	0	0	0	0	0	0	0	0	0	0	0	0	0	0	0	1	0	0	1	0	0
**PRDM1::PODNL1**	0	0	0	0	0	0	1	0	0	0	0	0	0	0	0	0	0	0	0	0	0	0	0	0	0	0	0	0
**PRDM1::SDC1**	0	0	0	0	0	0	0	0	1	0	0	0	0	0	0	0	0	0	0	0	0	0	0	1	0	0	0	0
**PRDM1::SPARC**	0	0	0	0	0	0	0	0	0	0	1	0	0	0	0	0	0	0	0	0	0	0	0	0	0	0	0	0
**PRDM1::ST14**	0	0	0	0	0	0	0	0	0	0	0	0	0	0	0	0	0	0	0	0	0	0	0	1	0	0	0	0
**PRDM1::WISP1**	0	0	1	0	0	0	0	0	0	0	0	0	0	0	0	1	0	0	0	0	0	0	0	0	0	0	0	0
**PRDM1::WNT5A**	0	0	0	0	0	0	0	0	0	0	0	0	0	0	0	0	0	0	0	0	0	0	0	1	0	0	0	0
**PRDM1::XCL2**	0	0	0	0	0	0	0	0	0	0	0	0	1	0	0	0	0	0	0	0	0	0	0	0	0	0	0	0
**PRRX1::ABI3BP**	0	0	0	0	0	0	0	0	0	0	0	0	0	1	0	0	0	0	0	0	0	0	0	0	0	0	0	0
**PRRX1::ADAMTS10**	0	0	0	0	0	0	0	0	0	0	0	0	0	0	1	0	0	0	0	0	0	0	0	0	0	0	0	0
**PRRX1::ASPN**	0	0	0	0	0	0	0	0	0	0	0	0	0	0	0	1	0	0	0	0	0	0	0	0	0	0	0	0
**PRRX1::FBN2**	0	1	0	0	0	0	0	0	0	0	0	0	0	0	0	0	0	0	0	0	0	0	0	0	0	0	0	0
**PRRX1::MMP1**	0	0	0	0	0	1	0	0	0	0	0	0	0	0	0	0	0	0	0	0	0	0	0	0	0	0	0	0
**PRRX1::MMP14**	0	1	0	0	0	0	0	0	0	0	0	0	0	0	0	0	0	0	0	0	0	0	0	0	0	0	0	0
**PRRX1::MMP7**	0	0	0	0	0	1	0	0	0	0	0	0	0	0	0	0	0	0	0	0	0	0	0	0	0	0	0	0
**PRRX1::PCOLCE**	0	0	0	0	0	0	0	0	0	0	0	0	0	0	0	1	0	0	0	0	0	0	0	0	0	0	0	0
**PRRX2::ADAMTSL5**	0	0	0	0	0	0	0	0	0	0	0	0	0	0	0	0	1	0	0	0	0	0	0	0	0	0	0	0
**PRRX2::ANGPTL4**	0	0	0	0	0	0	0	0	0	0	0	0	0	0	0	0	0	0	0	0	0	0	0	0	0	1	0	0
**PRRX2::C1QL1**	0	0	0	0	0	0	0	0	0	0	0	0	0	0	0	0	0	0	0	0	0	0	0	0	0	0	0	1
**PRRX2::CRLF1**	0	0	0	0	0	0	0	0	0	0	0	0	0	0	0	0	0	1	0	0	0	0	0	0	0	0	0	0
**PRRX2::FBLN2**	0	0	0	0	0	0	0	0	0	0	0	0	0	0	0	0	0	0	0	0	0	0	0	0	0	0	0	1
**PRRX2::IGFBP6**	0	0	0	0	0	0	0	0	0	0	0	0	0	0	0	0	0	0	0	0	0	0	0	0	0	1	0	0
**PRRX2::MMP14**	0	1	0	0	0	0	0	0	0	0	0	0	0	0	0	0	0	0	0	0	0	0	0	0	0	0	0	0
**PRRX2::OGFOD1**	0	0	0	0	0	0	0	0	0	0	0	0	0	0	0	0	1	0	0	0	0	0	0	0	0	0	0	0
**PRRX2::PODNL1**	0	1	1	0	0	0	1	0	0	0	0	0	0	0	0	1	0	0	0	0	0	0	0	0	0	0	0	0
**PRRX2::WNT2B**	0	0	0	0	0	0	0	0	0	0	0	0	0	0	0	0	0	0	0	0	0	0	0	0	1	0	0	0
**RARA::AGRN**	0	0	0	0	0	0	0	0	0	0	1	0	0	0	0	0	0	0	0	0	0	0	0	0	0	0	0	0
**RARA::ANGPTL2**	1	0	0	0	0	0	0	1	0	0	0	0	0	0	0	0	0	0	0	0	0	0	0	0	0	0	0	0
**RARA::ANGPTL4**	0	0	0	0	0	0	0	0	0	0	0	0	0	0	0	1	0	0	0	0	0	0	0	0	0	0	0	0
**RARA::BMP1**	0	0	0	0	0	0	0	0	0	0	0	1	0	0	0	0	0	0	0	0	0	0	0	0	0	0	0	0
**RARA::C1QTNF3**	0	0	0	0	0	0	0	0	0	0	0	0	0	0	0	0	0	0	0	0	0	0	0	0	0	0	0	1
**RARA::CCL7**	0	0	0	0	0	0	0	0	0	0	0	0	0	0	0	0	0	0	0	0	0	0	0	0	0	0	0	1
**RARA::CHRD**	0	0	1	0	0	0	0	0	0	0	0	0	0	0	0	0	0	0	0	0	0	0	0	0	0	0	0	0
**RARA::CILP2**	0	0	0	0	0	0	0	0	0	0	0	0	0	0	0	0	0	1	0	0	0	0	0	0	0	0	0	0
**RARA::COL8A1**	0	0	0	0	0	0	0	0	0	0	0	0	0	0	0	0	0	0	0	0	1	0	0	0	0	0	0	0
**RARA::CSPG4**	0	0	0	0	0	0	0	1	1	0	0	0	0	0	0	0	0	0	0	0	0	0	0	0	0	0	0	0
**RARA::HTRA3**	0	0	0	0	0	0	0	0	0	0	0	0	0	0	0	0	0	0	0	0	0	0	0	1	0	0	0	0

**RARA::IGFBP4**	0	0	0	0	0	0	0	0	0	0	1	0	0	0	0	0	0	0	0	0	0	0	0	0	0	0	0	0
**RARA::LGALS8**	0	0	1	0	0	0	0	0	0	0	0	0	0	0	0	0	0	0	0	0	0	0	0	0	0	0	0	0
**RARA::LOXL3**	0	0	0	0	0	0	0	0	0	0	0	0	0	0	0	0	0	0	0	1	0	0	0	0	0	0	0	0
**RARA::SDC1**	0	0	0	0	0	0	0	0	0	0	0	0	0	0	0	1	0	0	0	0	0	0	0	0	0	0	0	0
**RARA::SEMA3F**	0	0	1	0	0	0	0	0	0	0	0	0	0	0	0	0	0	0	0	0	0	0	0	0	0	0	0	0
**RARA::SEMA4B**	0	0	0	0	0	0	0	0	0	0	0	0	0	0	0	1	0	0	0	0	0	0	0	0	0	0	0	0
**RARA::SERPINE1**	0	0	0	0	0	0	0	0	1	0	0	0	0	0	0	0	0	0	0	0	0	0	0	0	0	0	0	0
**RARA::TGM2**	0	0	0	0	0	0	0	0	0	0	0	0	0	0	0	0	0	0	0	0	1	0	0	0	0	0	0	0
**RARA::TNC**	0	0	0	0	0	0	0	0	1	0	0	0	0	0	0	0	0	0	0	0	0	0	0	0	0	0	0	0
**RARA::WNT9A**	0	0	1	0	0	0	0	0	0	0	0	0	0	0	0	0	0	0	0	0	0	0	0	0	0	0	0	0
**RARA::VWA1**	0	0	0	0	0	0	0	0	0	0	0	0	0	0	0	1	0	0	0	0	0	0	0	0	0	0	0	0
**RARA::ZFP91**	0	0	0	0	0	0	0	0	0	0	0	0	1	0	0	0	0	0	0	0	0	0	0	0	0	0	0	0
**RARB::COL13A1**	0	0	0	0	0	0	0	0	1	0	0	0	0	0	0	0	0	0	0	0	0	0	0	0	0	0	0	0
**RARB::COL8A1**	0	0	0	0	0	0	1	0	0	0	0	0	0	0	0	0	0	0	0	0	0	0	0	0	0	0	0	0
**RARB::CST2**	0	0	0	0	0	0	1	0	0	0	0	0	0	0	0	0	0	0	0	0	0	0	0	0	0	0	0	0
**RARB::ESM1**	0	0	0	0	0	0	0	0	0	0	0	0	0	0	0	0	0	0	0	1	0	0	0	0	0	0	0	0
**RARB::FGF1**	0	0	0	0	0	0	0	0	0	0	0	0	0	0	0	0	0	0	0	1	0	0	0	0	0	0	0	0
**RARB::LOXL1**	0	0	0	0	0	0	0	0	1	0	0	0	0	0	0	0	0	0	0	0	0	0	0	0	0	0	0	0
**RARB::MMRN2**	1	0	0	0	0	0	0	0	0	0	0	0	0	0	0	0	0	0	0	0	0	0	0	0	0	0	0	0
**RARB::PAMR1**	0	0	0	0	0	0	0	0	0	0	1	0	0	0	0	0	0	0	0	0	0	0	0	0	0	0	0	0
**RARB::SRPX**	1	0	0	0	0	0	0	0	0	0	0	0	0	0	0	0	0	0	0	0	0	0	0	0	0	0	0	0
**RARB::WNT5A**	0	0	0	0	0	0	1	0	0	0	0	0	0	0	0	0	1	0	0	0	0	0	0	0	0	0	0	0
**RARG::ADAMTSL5**	0	0	0	0	0	0	0	0	0	0	0	0	0	0	0	0	0	0	0	0	0	1	0	0	0	0	0	0
**RARG::CST6**	0	0	0	0	0	0	0	0	0	0	0	0	0	0	0	0	0	0	0	0	0	0	0	0	0	1	0	0
**RARG::CSTA**	0	0	0	1	0	0	0	0	0	0	0	0	0	0	0	0	1	0	0	0	0	0	0	0	0	0	0	0
**RARG::ELN**	0	0	0	0	0	0	0	0	0	0	0	0	0	0	0	0	0	0	0	1	0	0	0	0	0	0	0	0
**RARG::LAMA5**	0	0	0	0	0	0	0	0	0	0	0	0	0	0	0	0	0	0	0	0	0	0	0	0	0	0	1	0
**RARG::LGI4**	0	0	0	0	0	0	0	0	0	0	1	0	0	0	0	0	0	0	0	0	0	0	0	0	0	0	0	0
**RARG::LOXL1**	0	0	0	0	0	0	0	0	0	0	0	0	0	0	0	0	1	1	0	0	0	0	0	0	1	0	0	0
**RARG::LOXL3**	0	0	0	0	0	0	0	1	0	0	0	0	0	0	0	0	0	0	0	1	0	0	0	0	0	0	0	0
**RARG::MMP14**	0	0	0	0	0	0	0	0	0	0	0	0	0	0	0	0	0	0	0	0	0	0	0	1	0	0	0	0
**RARG::PLXNA3**	0	0	0	0	0	0	0	0	0	0	0	0	0	0	0	0	0	0	0	0	0	0	0	0	0	1	0	0
**RARG::PLXNB2**	0	1	0	0	0	0	0	0	1	0	0	0	0	0	0	0	0	0	0	0	0	0	0	0	0	0	0	0
**RARG::PLXND1**	0	0	0	0	0	0	0	0	0	0	1	0	0	0	0	0	0	0	0	0	0	0	0	0	0	1	0	0
**RARG::PODNL1**	0	0	0	0	0	1	0	0	0	0	0	0	0	0	0	0	0	0	0	0	0	0	0	0	0	0	0	0
**RARG::SDC1**	0	1	0	0	0	0	0	0	0	0	0	0	0	0	0	0	0	0	0	0	0	0	0	0	0	0	0	0
**RARG::SEMA3F**	0	0	0	1	0	0	0	0	0	0	0	0	0	0	0	0	0	0	0	0	0	0	0	0	0	0	0	0
**RARG::SEMA4A**	0	1	0	0	0	0	0	0	0	0	0	0	0	0	0	0	0	0	0	0	0	0	0	0	0	0	0	0
**RARG::SERPINB8**	0	0	0	1	0	0	0	0	0	0	0	0	0	0	0	0	0	0	0	0	0	0	0	0	0	0	0	0
**RARG::SPOCK1**	1	0	0	0	0	0	0	0	0	0	0	0	0	0	0	0	0	0	0	0	0	0	0	0	0	0	0	0
**RARG::ST14**	0	0	0	0	0	0	0	0	0	0	0	0	0	0	0	0	1	0	0	0	0	0	0	0	0	0	0	0
**RARG::THBS3**	0	0	0	0	0	0	0	0	0	0	0	0	0	0	0	0	0	0	0	0	0	0	0	0	0	0	0	1
**RARG::WNT5A**	0	0	0	0	0	0	0	0	0	0	0	0	0	0	0	0	0	0	0	0	0	0	0	0	0	1	0	0
**RBPJ::LGALS1**	0	0	0	0	0	0	0	0	0	0	0	0	0	0	0	0	0	0	0	0	0	0	1	0	0	0	0	0
**RBPJ::LGI2**	0	0	0	0	0	0	0	0	0	0	0	0	0	0	0	0	1	0	0	0	0	0	0	0	0	0	0	0
**RBPJ::NELL2**	0	0	0	0	0	0	0	0	0	0	0	0	0	0	0	0	0	0	0	1	0	0	0	0	0	0	0	0

**RBPJ::SEMA3C**	0	0	0	0	0	0	0	0	0	1	0	0	0	0	0	0	0	0	0	0	0	0	0	0	0	0	0	0
**RBPJ::XCL1**	0	0	0	0	0	0	1	0	0	0	0	0	0	0	0	0	0	0	0	0	0	0	0	0	0	0	0	0
**REL::CLEC7A**	0	0	0	0	0	0	0	0	0	0	1	0	0	0	0	0	0	0	0	0	0	0	0	0	0	0	0	0
**REL::RSPO2**	0	0	0	0	0	0	0	0	0	0	0	0	0	0	0	0	0	0	0	0	0	0	0	0	0	0	0	1
**REL::SEMA4D**	0	0	0	0	0	0	0	0	0	0	0	0	0	0	0	0	0	0	0	0	0	0	0	0	1	0	0	0
**REL::SRGN**	0	0	0	0	0	0	0	0	0	0	1	0	0	0	0	0	0	0	0	0	0	0	0	0	0	0	0	0
**RELA::ANXA2**	0	0	0	0	0	0	0	0	0	0	0	0	0	0	0	0	0	1	0	0	0	0	0	0	0	0	0	0
**RELA::BGN**	0	0	0	0	0	0	0	0	0	0	1	0	0	0	0	1	0	0	0	0	0	0	0	0	0	0	0	0
**RELA::C1QTNF1**	1	0	0	0	0	1	0	0	0	0	0	0	0	0	0	0	0	0	0	0	0	0	0	0	0	0	0	0
**RELA::C1QTNF5**	0	0	0	0	0	0	0	1	0	0	0	0	0	0	0	0	0	0	0	0	0	0	0	0	0	0	0	0
**RELA::EGLN2**	0	0	0	0	0	0	0	0	0	1	0	0	0	0	0	0	0	0	0	0	0	0	0	0	0	0	0	0
**RELA::HPSE**	0	0	0	0	0	0	0	0	0	0	0	0	0	0	0	1	0	0	0	0	0	0	0	0	0	0	0	0
**RELA::LTBP3**	0	0	0	0	1	0	0	0	0	0	0	0	0	1	0	0	0	0	0	0	0	0	0	0	0	1	0	0
**RELA::SERPINE1**	0	0	0	0	0	0	0	0	0	0	1	0	0	0	0	0	0	0	0	0	0	0	0	0	0	0	0	0
**REST::COL4A1**	0	0	0	0	0	0	0	0	0	0	0	0	0	0	0	0	0	0	0	1	0	0	0	0	0	0	0	0
**REST::FBN2**	0	0	0	0	0	0	0	0	0	0	0	0	0	0	0	0	0	0	0	0	0	0	1	0	0	0	0	0
**REST::IMPG1**	0	0	0	0	0	0	0	0	0	0	0	0	0	0	0	0	0	0	0	0	1	0	0	0	0	0	0	0
**REST::LAMA5**	0	0	0	0	0	0	0	0	0	0	0	0	0	0	0	0	0	0	0	0	0	0	0	0	0	0	1	0
**REST::LOXL3**	0	0	0	0	0	0	0	0	0	0	0	0	0	1	0	0	0	0	0	0	0	0	0	0	0	0	0	0
**REST::MFAP2**	0	0	0	0	0	0	0	0	0	0	0	0	0	0	0	0	1	0	0	0	0	0	0	0	0	0	0	0
**REST::MFAP3**	0	0	0	0	0	0	0	0	0	0	0	0	1	0	0	0	0	0	0	0	0	0	0	0	0	0	0	0
**REST::PLXNC1**	1	0	0	0	0	0	0	0	0	0	0	0	0	0	0	0	0	0	0	0	0	0	0	0	0	1	0	0
**REST::SDC1**	0	0	0	0	0	0	0	0	0	0	0	0	0	0	0	0	1	0	0	0	0	0	0	0	0	0	0	0
**REST::TNC**	0	0	0	0	0	0	0	0	0	0	0	0	0	1	0	0	0	0	0	0	0	0	0	0	0	0	0	0
**REST::WNT5A**	0	0	0	0	0	0	0	0	0	0	0	0	0	1	0	0	0	0	0	0	0	0	0	0	0	0	0	0
**RFX1::ADAMTS10**	0	0	0	0	1	0	0	0	0	0	0	0	0	0	0	0	0	0	0	0	0	0	0	0	0	0	0	0
**RFX1::CHRD**	0	0	1	0	0	0	0	0	0	0	0	0	0	0	0	0	0	0	0	0	0	0	0	0	0	0	0	0
**RFX1::CILP2**	0	1	1	0	0	0	0	0	0	0	0	0	0	0	0	0	0	0	0	0	0	0	0	0	0	0	0	0
**RFX1::CTHRC1**	0	0	1	0	0	0	0	0	0	0	0	0	0	0	0	0	0	0	0	0	0	0	0	0	0	0	0	0
**RFX1::HSPG2**	0	0	0	0	0	0	0	0	1	0	0	0	0	0	0	0	0	0	0	0	0	0	0	0	0	0	0	0
**RFX1::LAMA5**	0	0	0	0	0	0	0	0	0	0	1	1	0	0	0	0	0	0	0	0	0	0	0	0	0	1	0	0
**RFX1::LTBP3**	0	0	0	0	0	0	0	0	0	0	0	0	0	1	0	0	0	0	0	0	0	0	0	0	0	1	0	0
**RFX1::MMP17**	0	0	1	0	0	0	0	0	0	0	0	0	0	0	0	0	0	0	0	0	0	0	0	0	0	0	0	0
**RFX1::PCOLCE**	0	0	0	0	0	0	0	0	0	0	0	0	0	0	0	1	0	0	0	0	0	0	0	0	0	0	0	0
**RFX1::SEMA4D**	0	0	0	0	0	0	0	0	0	0	0	0	0	0	0	0	0	0	0	0	0	0	0	1	0	0	0	0
**RFX2::ADAM32**	0	0	0	0	0	0	0	0	0	0	0	0	0	0	0	0	0	0	0	0	0	0	0	0	1	0	0	0
**RFX2::ADAMTS5**	0	0	0	0	0	0	0	0	0	0	1	0	0	0	0	0	0	0	0	0	0	0	0	0	0	0	0	0
**RFX2::ANXA1**	0	0	0	0	0	0	0	0	0	0	0	0	0	0	0	0	0	1	0	0	0	0	0	0	0	0	0	0
**RFX2::BDNF**	0	0	0	0	0	0	0	0	0	0	1	0	0	0	0	0	0	0	0	0	0	0	0	0	0	0	0	0
**RFX2::ECM2**	0	0	0	0	0	0	0	1	0	0	0	0	0	0	0	0	0	0	0	0	0	0	0	0	0	0	0	0
**RFX2::LGALS1**	1	0	0	0	0	0	0	0	0	0	0	0	0	0	0	0	0	0	0	0	0	0	0	0	0	0	0	0
**RFX2::LGI4**	0	0	0	0	0	0	0	0	0	0	1	0	0	0	0	0	0	0	0	0	0	0	0	0	0	0	0	0
**RFX2::LTBP3**	0	0	0	0	1	0	0	0	0	0	0	0	0	0	0	0	0	0	0	0	0	0	0	0	0	0	0	0
**RFX2::MMP17**	0	0	0	0	0	0	0	0	0	0	0	0	0	0	0	0	0	0	0	1	0	0	0	0	0	0	0	0
**RFX2::PLXNB1**	0	0	0	1	0	0	0	0	0	0	0	0	0	0	0	0	0	0	0	0	0	0	0	1	0	0	0	0
**RFX2::SPOCK1**	1	0	0	0	0	0	0	0	0	0	0	0	0	0	0	0	0	0	0	0	0	0	0	0	0	0	0	0

**RFX2::THBS3**	0	0	0	0	0	0	0	0	0	0	0	1	0	0	0	0	0	0	0	0	0	0	0	0	0	0	0	0
**RFX2::TNXB**	0	0	0	0	0	0	0	0	0	0	0	0	0	0	0	0	0	1	0	0	0	0	0	0	0	0	0	0
**RFX3::ADAMTS6**	0	0	0	0	0	1	0	0	0	0	0	0	0	1	0	0	0	0	0	0	0	0	0	0	0	0	0	0
**RFX3::COLEC12**	0	0	0	0	0	0	0	0	0	0	0	0	0	0	0	0	0	0	0	0	1	0	0	0	0	0	0	0
**RFX3::CXCL3**	0	0	0	0	0	0	0	0	1	0	0	0	0	0	0	0	0	0	0	0	0	0	0	0	0	0	0	0
**RFX3::ECM2**	0	0	0	0	0	0	0	0	0	0	0	0	0	0	0	0	0	1	0	0	0	0	0	0	0	0	0	0
**RFX3::P4HA1**	0	0	0	0	0	0	0	0	0	0	0	0	0	0	0	0	0	0	0	0	0	0	0	0	0	0	1	0
**RFX3::PLXNB1**	0	0	0	1	0	0	0	0	0	0	0	0	0	0	0	0	0	0	0	0	0	0	0	0	0	0	1	0
**RFX3::WNT8B**	0	0	0	0	0	1	0	0	0	0	0	0	0	0	0	0	0	0	0	0	0	0	0	0	0	0	0	0
**RFX5::ADAMTS6**	0	0	0	0	0	0	0	1	0	0	0	0	0	1	0	0	0	0	0	0	0	0	0	0	0	0	0	0
**RFX5::CXCL5**	0	0	0	0	0	0	0	0	0	0	0	0	0	0	0	0	0	0	0	0	0	1	0	0	0	0	0	0
**RFX5::LGALS9**	0	0	0	0	1	0	0	0	0	0	0	0	0	0	0	0	0	0	0	0	0	0	0	0	0	0	0	0
**RFX5::SEMA4D**	0	0	0	0	0	0	0	0	0	0	0	0	0	0	0	1	0	0	0	0	0	0	0	0	1	0	0	0
**RFX7::MFAP1**	0	0	0	0	0	0	0	0	0	0	0	0	0	0	0	0	1	0	0	0	0	0	0	0	0	0	0	0
**RFX7::PLXNB1**	0	1	0	0	0	0	0	0	0	0	0	0	0	0	0	0	0	0	0	0	0	0	0	0	0	0	0	0
**RFX7::VWA9**	0	1	0	0	0	0	1	0	0	0	0	0	0	0	0	0	1	0	0	0	0	0	0	1	0	0	0	0
**RORA::ADAM15**	0	0	0	0	0	0	0	0	0	0	0	0	0	0	0	0	0	0	0	0	1	0	0	0	0	0	0	0
**RORA::CD109**	0	0	0	0	0	0	0	0	0	0	0	0	0	0	0	0	1	0	0	0	0	0	0	0	0	0	0	0
**RORA::FMOD**	0	0	0	0	0	0	0	0	0	0	0	0	0	0	0	0	0	1	0	0	0	0	0	0	0	0	0	0
**RORA::HMCN1**	0	0	0	0	0	0	0	0	0	0	1	0	0	0	0	0	0	0	0	0	0	0	0	0	0	0	0	0
**RORA::HYAL3**	0	0	0	0	0	0	0	0	0	0	0	0	0	0	0	0	0	0	0	0	1	0	0	0	0	0	0	0
**RORA::P4HA2**	0	0	0	0	0	0	0	0	0	0	1	0	0	0	0	0	0	0	0	0	0	0	0	0	0	0	0	1
**RORA::PXDN**	0	0	0	0	0	0	0	0	0	0	0	0	0	0	0	0	0	0	0	0	0	0	1	0	0	0	0	0
**RORA::SRPX2**	0	0	0	0	0	0	0	0	0	0	0	0	0	0	0	0	0	0	0	0	0	0	0	1	0	0	0	0
**RREB1::CSF1**	0	0	0	0	0	0	0	0	0	0	0	0	0	1	0	0	0	0	0	0	0	0	0	0	0	0	0	0
**RREB1::EGLN2**	0	0	0	0	0	0	0	0	0	0	0	0	0	0	0	0	0	0	0	0	0	0	0	0	0	0	0	1
**RREB1::EPGN**	0	0	0	1	0	0	0	0	0	0	0	0	0	0	0	0	0	0	0	0	0	0	0	0	0	0	0	0
**RREB1::FGF2**	0	0	0	0	0	0	0	0	0	0	0	0	0	1	0	0	0	0	0	0	0	0	0	0	0	0	0	0
**RREB1::LAMC1**	0	0	0	0	0	0	0	0	0	0	0	0	0	0	0	0	0	0	0	0	0	0	0	0	1	0	0	0
**RREB1::LRG1**	0	0	1	0	0	0	0	0	0	0	0	0	0	0	0	0	0	0	0	0	0	0	0	0	0	0	0	0
**RREB1::NTNG2**	0	0	0	0	0	0	0	0	0	0	0	0	1	0	0	0	0	0	0	0	0	0	0	0	0	0	0	0
**RREB1::PLXNB2**	0	0	0	0	0	0	0	0	0	0	0	0	0	0	0	0	0	0	0	0	0	0	0	1	0	0	0	0
**RREB1::PXDN**	0	0	0	0	0	0	0	0	0	0	0	0	0	0	0	0	0	0	0	0	1	0	0	0	0	0	0	0
**RREB1::SEMA4A**	0	0	0	0	0	0	0	0	0	0	0	0	0	0	0	0	0	0	0	0	1	0	0	0	0	0	0	0
**RREB1::SEMA4B**	0	0	0	0	0	0	1	0	1	0	0	0	0	0	0	0	0	0	0	0	0	0	0	0	0	0	0	0
**RREB1::TINAGL1**	0	1	0	0	0	0	0	0	0	0	0	0	0	0	0	0	0	0	0	0	0	0	0	0	0	0	0	0
**RREB1::VCAN**	0	0	1	0	0	0	0	0	0	0	0	0	0	0	0	0	0	0	0	0	0	0	0	0	0	0	0	0
**RUNX1::A2M**	0	0	0	0	0	0	0	0	0	0	0	0	0	1	0	0	0	0	0	0	0	0	0	0	1	0	0	0
**RUNX1::ANXA5**	0	0	0	0	0	0	0	0	0	0	1	0	0	0	0	0	0	0	0	0	0	0	0	0	0	0	0	0
**RUNX1::BMP4**	0	0	0	0	0	0	0	0	0	0	0	0	0	0	0	0	0	0	0	0	0	1	0	0	0	0	0	0
**RUNX1::CSF1**	0	0	0	0	0	0	0	0	0	0	0	0	0	1	0	0	0	0	0	0	0	0	0	0	0	0	0	0
**RUNX1::FAM20C**	0	0	0	0	0	0	0	1	0	0	0	0	0	0	0	0	0	0	0	0	0	0	0	0	0	0	0	0
**RUNX1::LAMA4**	0	0	0	0	0	0	0	1	0	0	1	0	0	0	0	0	0	0	0	0	0	0	0	0	0	0	0	0
**RUNX1::NTN1**	0	0	0	0	0	0	0	0	0	0	0	0	1	0	0	0	0	0	0	0	0	0	0	0	0	0	0	0
**RUNX1::SERPINB8**	0	0	0	0	0	0	0	0	0	0	0	0	0	0	0	0	0	0	0	0	0	0	0	0	0	1	0	0
**RUNX1::VCAN**	0	0	0	0	0	0	0	0	0	0	1	0	0	0	0	0	0	0	0	0	0	0	0	0	0	0	0	0

**RUNX2::ANXA10**	0	0	0	0	0	0	0	0	0	0	0	0	0	0	0	0	1	0	0	0	0	0	0	0	0	0	0	0
**RUNX2::FBN2**	0	1	0	0	0	0	0	0	0	0	0	0	0	0	0	0	0	0	0	0	0	0	0	0	0	0	0	0
**RUNX2::HTRA3**	0	0	0	0	0	0	0	0	0	0	0	0	0	0	0	0	1	0	0	0	0	0	0	0	0	0	0	0
**RUNX2::MMP13**	0	1	0	0	0	0	0	0	1	0	0	0	0	0	0	0	0	0	0	0	0	0	0	0	0	0	0	0
**RUNX2::PLXNC1**	0	0	0	0	0	0	0	0	0	0	0	0	0	0	0	0	0	0	0	0	0	0	0	0	0	1	0	0
**RUNX2::SEMA3C**	0	0	0	0	0	0	0	0	1	0	0	0	0	0	0	0	0	0	0	0	0	0	0	0	0	0	0	0
**RUNX2::SEMA4A**	0	0	0	0	0	0	0	0	0	0	0	0	1	0	0	0	0	0	0	0	0	0	0	0	0	0	0	0
**RUNX2::SERPINB8**	0	0	0	0	0	0	0	0	0	0	0	0	1	0	0	0	0	0	0	0	0	0	0	0	0	0	0	0
**RUNX3::PLXNC1**	0	0	0	0	0	0	0	0	0	0	0	0	1	0	0	0	0	0	0	0	0	0	0	0	0	0	0	0
**RUNX3::SEMA4D**	0	0	0	0	0	0	0	0	0	0	0	0	0	0	0	1	0	0	0	0	0	0	0	0	0	0	0	0
**RXRA::HPSE**	0	0	0	0	0	0	1	0	0	0	0	0	0	0	0	0	0	0	0	0	0	0	0	0	0	0	0	0
**RXRA::SEMA3F**	0	0	1	0	0	0	0	0	0	0	0	0	0	0	0	0	0	0	0	0	0	0	0	0	0	0	0	0
**RXRA::SEMA4A**	0	0	0	0	0	0	0	0	0	0	0	0	1	0	0	0	0	0	0	0	0	0	0	0	0	0	0	0
**RXRA::WNT11**	0	0	0	0	0	0	0	0	0	0	0	0	0	0	0	0	0	0	0	0	0	0	0	0	1	0	0	0
**RXRB::BGLAP**	0	1	0	0	0	0	0	0	0	0	0	0	0	0	0	0	0	0	0	0	0	0	0	0	0	0	0	0
**RXRB::OGFOD2**	0	0	0	0	0	0	0	0	0	0	0	0	0	0	0	0	0	0	0	0	1	0	0	0	0	0	0	0
**RXRB::PLXNB2**	0	0	0	0	0	0	0	0	0	0	0	0	0	0	0	0	0	0	0	0	1	0	0	0	0	0	0	0
**RXRG::LAMA4**	0	0	0	0	0	0	0	0	0	0	0	0	0	0	0	0	0	0	0	0	0	0	1	0	0	0	0	0
**SHOX2::FBLN7**	0	0	0	0	0	0	0	0	0	0	0	0	0	0	0	0	0	0	0	0	0	0	1	0	0	0	0	0
**SHOX2::SERPINE2**	0	0	0	0	0	0	0	0	0	0	0	0	0	0	0	0	1	0	0	0	0	0	0	0	0	0	0	0
**SMAD3::ADAMTS10**	0	0	0	0	0	0	0	0	0	0	0	0	1	0	0	0	0	0	0	0	0	0	0	0	0	0	0	0
**SMAD3::ANXA2**	0	0	0	1	0	0	1	0	0	0	0	0	0	0	0	0	0	0	0	0	0	0	0	1	0	0	0	0
**SMAD3::IGFBP3**	0	1	0	0	0	0	0	0	0	0	0	0	0	0	0	0	0	0	0	0	0	0	0	0	0	0	0	0
**SMAD3::LAMC2**	0	0	0	0	0	1	0	0	0	0	0	0	0	0	0	0	0	0	0	0	0	1	0	0	0	0	0	0
**SMAD3::MMP13**	0	1	0	0	0	0	0	0	0	0	0	0	0	0	0	0	0	0	0	0	0	0	0	0	0	0	0	0
**SMAD3::NELL2**	0	0	0	0	0	0	0	0	0	0	0	0	0	0	0	0	0	0	0	0	0	0	0	0	0	1	0	0
**SMAD3::NTNG2**	1	0	0	0	0	0	0	0	0	0	0	0	0	0	0	0	0	0	0	0	0	0	0	0	0	0	0	0
**SMAD3::P4HA2**	0	0	0	0	0	0	0	0	0	0	0	0	0	0	0	0	0	0	0	0	0	0	0	0	0	1	0	0
**SMAD3::PLAU**	0	0	0	1	0	0	0	0	0	0	0	0	0	0	0	0	0	0	0	0	0	0	0	0	0	0	0	0
**SMAD3::PLXNB2**	0	1	0	0	0	0	0	0	0	0	0	0	0	0	0	0	0	0	0	0	0	0	0	0	0	0	0	0
**SMAD3::PRSS12**	0	0	0	0	0	0	0	0	0	0	0	0	0	0	0	0	0	0	1	0	0	0	0	0	0	0	0	0
**SMAD3::SDC4**	0	0	1	0	0	0	0	0	0	0	0	0	0	0	0	0	0	0	0	0	0	0	0	1	0	0	0	0
**SMAD3::SEMA4B**	0	0	0	0	0	0	1	0	1	0	0	0	0	0	0	0	0	0	0	0	0	0	0	0	0	0	0	0
**SMAD3::SEMA7A**	1	0	0	0	0	0	0	0	0	0	0	0	0	0	0	1	0	0	0	0	0	0	0	1	0	0	0	0
**SMAD3::SMOC1**	0	0	0	0	0	0	0	0	0	1	0	0	0	0	0	0	0	0	0	0	0	0	0	0	0	0	0	0
**SMAD3::SPOCK2**	0	0	0	0	0	0	0	0	0	0	0	0	0	0	0	0	0	0	0	0	0	0	0	0	0	1	0	0
**SNAI2::BGN**	0	0	0	0	0	0	0	0	0	0	1	0	0	0	0	0	0	0	0	0	0	0	0	0	0	0	0	0
**SNAI2::C1QTNF6**	0	0	1	0	0	0	0	0	0	0	0	0	0	0	0	1	0	0	0	0	0	0	0	0	0	0	0	0
**SNAI2::COL15A1**	0	0	0	0	0	0	0	0	0	0	0	0	0	0	0	0	0	1	0	0	0	0	0	0	0	0	0	0
**SNAI2::COL7A1**	0	0	0	0	0	0	1	0	1	0	0	0	0	0	0	0	0	0	0	0	0	0	0	0	0	0	0	0
**SNAI2::LOX**	0	0	0	0	0	0	0	0	0	0	0	0	0	0	0	0	0	0	0	0	0	0	0	1	0	0	0	0
**SNAI2::TNC**	0	0	0	0	0	0	1	0	0	0	0	0	0	0	0	0	0	0	0	0	0	0	0	0	0	0	0	0
**SNAI2::WISP1**	0	0	0	0	0	0	0	1	0	0	0	0	0	0	0	0	0	0	0	0	0	0	0	0	0	0	0	0
**SOX10::SERPINH1**	0	0	0	0	0	0	0	0	0	0	0	0	0	0	0	0	0	0	0	0	0	0	0	0	1	0	0	0
**SOX11::ANGPT2**	0	0	0	0	0	0	0	0	0	0	0	0	0	0	0	0	0	0	0	0	0	0	0	1	0	0	0	0
**SOX11::IGFBP2**	0	0	0	0	0	0	0	1	0	0	0	0	0	0	0	0	0	0	0	0	0	0	0	0	0	0	0	0

**SOX11::LAMA4**	0	0	0	0	0	0	0	0	0	0	0	0	0	0	0	0	0	0	0	0	0	0	0	0	1	0	0	0
**SOX11::PLAU**	0	0	1	0	0	0	0	0	0	0	0	0	0	0	0	0	0	0	0	0	0	0	0	0	0	0	0	0
**SOX12::PLXNB3**	0	0	0	0	0	0	0	0	1	0	0	0	0	0	0	0	0	0	0	0	0	0	0	0	0	0	0	0
**SOX13::ANXA10**	0	0	0	0	0	0	0	0	1	0	0	0	0	0	0	0	0	0	0	0	0	0	0	0	0	0	0	0
**SOX13::LAMA5**	0	0	0	0	0	0	0	0	1	0	0	0	0	0	0	0	0	0	0	0	0	0	0	0	0	0	0	0
**SOX15::COL7A1**	0	0	0	1	0	0	0	0	0	0	0	0	0	0	0	0	0	0	0	0	0	0	0	0	0	0	0	0
**SOX15::FGF11**	0	0	0	0	0	0	1	0	0	0	0	0	0	0	0	0	0	0	0	0	0	0	0	0	0	0	0	0
**SOX15::SDC1**	0	0	0	0	0	0	0	0	1	0	0	0	0	0	0	0	0	0	0	0	0	0	0	0	0	0	0	0
**SOX15::SEMA4B**	0	0	0	1	0	0	0	0	0	0	0	0	0	0	0	0	0	0	0	0	0	0	0	0	0	0	0	0
**SOX15::WNT3A**	0	0	0	0	0	0	1	0	0	0	0	0	0	0	0	0	0	0	0	0	0	0	0	0	0	0	0	0
**SOX17::LAMA4**	0	0	0	0	0	0	0	0	0	0	0	0	0	0	0	0	0	0	0	0	0	0	1	0	0	0	0	0
**SOX18::ANGPT2**	0	0	0	0	0	0	0	0	0	0	0	0	0	0	0	0	0	0	0	1	0	0	0	0	0	0	0	0
**SOX18::CHRD**	0	0	0	0	0	0	0	0	0	0	0	0	0	0	0	1	0	0	0	0	0	0	0	0	0	0	0	0
**SOX18::EGFL7**	0	0	0	0	0	0	0	0	0	0	1	0	0	0	0	0	0	0	0	0	0	0	0	0	0	0	0	0
**SOX18::PAPLN**	0	0	0	0	0	0	0	0	1	0	0	0	0	0	0	0	0	0	0	0	0	0	0	0	0	0	0	0
**SOX18::THBS3**	0	0	0	0	0	0	0	0	0	0	0	0	0	0	0	0	0	0	0	0	0	0	0	0	0	0	0	1
**SOX2::HPSE**	0	0	0	1	0	0	0	0	0	0	0	0	0	0	0	0	0	0	0	0	0	0	0	0	0	0	0	0
**SOX2::LOX**	0	0	0	0	0	0	0	0	0	0	0	0	0	0	0	0	0	0	0	0	0	0	0	0	0	0	0	1
**SOX2::NTN1**	0	0	0	0	0	0	0	1	0	0	0	0	0	0	0	0	0	0	0	0	0	0	0	0	0	0	0	0
**SOX2::SMOC1**	0	0	0	0	0	0	0	0	0	0	0	0	0	1	0	0	0	0	0	0	0	0	0	0	0	0	0	0
**SOX2::WNT5A**	0	0	0	0	0	0	0	0	0	0	0	0	0	1	0	0	0	0	0	0	0	0	0	0	0	0	0	0
**SOX21::WNT2B**	0	0	0	0	0	0	0	0	1	0	0	0	0	0	0	0	0	0	0	0	0	0	0	0	0	0	0	0
**SOX4::THBS3**	0	0	0	0	0	0	0	0	0	0	0	0	0	0	0	0	0	0	0	1	0	0	0	0	0	0	0	0
**SOX5::COL4A6**	0	0	0	0	0	0	0	0	0	0	0	0	0	0	0	0	1	0	0	0	0	0	0	0	0	0	0	0
**SOX5::PODNL1**	0	0	0	0	0	0	0	0	0	0	0	0	0	0	0	0	1	0	0	0	0	0	0	0	0	0	0	0
**SOX8::CRLF1**	0	0	0	0	0	0	0	0	0	0	0	0	0	0	0	0	0	1	0	0	0	0	0	0	0	0	0	0
**SOX8::MASP1**	0	0	0	0	0	0	0	1	0	0	0	0	0	0	0	0	0	0	0	0	0	0	0	0	0	0	0	0
**SOX8::WNT11**	1	0	0	0	0	0	0	0	0	0	0	0	0	0	0	0	0	0	0	0	0	0	0	0	0	0	0	0
**SOX9::ADAMTS14**	0	0	0	0	0	0	0	0	0	0	0	0	0	0	0	0	0	0	0	0	0	0	0	1	0	0	0	0
**SOX9::CLCF1**	0	0	0	0	0	0	0	0	0	0	0	1	0	0	0	0	0	0	0	0	0	0	0	0	0	0	0	0
**SOX9::LAMA1**	0	0	0	0	0	0	0	0	0	0	0	0	0	0	0	0	1	0	0	0	0	0	0	0	0	0	0	0
**SOX9::LAMC1**	0	0	0	0	1	0	0	0	0	0	0	0	0	0	0	0	0	0	0	0	0	0	0	0	0	0	0	0
**SOX9::MASP1**	0	0	0	0	0	0	0	1	0	0	0	0	0	0	0	0	0	0	0	0	0	0	0	0	0	0	0	0
**SOX9::MMP7**	0	0	0	0	0	0	0	0	1	0	0	0	0	0	0	0	0	0	0	0	0	0	0	0	0	0	0	0
**SOX9::SEMA4A**	0	0	1	0	0	0	0	0	0	0	0	0	0	0	0	0	0	0	0	0	0	0	0	0	0	0	0	0
**SOX9::THBS3**	0	0	0	0	0	0	0	0	0	0	0	1	0	0	0	0	0	0	0	0	0	0	0	0	0	0	0	0
**SP1::ADAM15**	0	1	0	0	0	0	0	0	0	0	0	0	0	0	0	0	0	0	0	0	0	0	0	0	0	0	0	0
**SP1::ADAMTS3**	0	0	0	0	0	0	0	0	0	0	0	1	0	0	0	0	0	0	0	0	0	0	0	0	0	0	0	0
**SP1::CLEC14A**	0	0	0	0	0	0	0	0	0	0	0	0	0	0	0	0	0	0	0	0	0	0	0	0	0	1	0	0
**SP1::CRELD2**	0	0	0	0	0	0	0	0	0	0	0	0	0	0	0	0	0	0	0	0	0	0	0	0	0	0	0	0
**SP1::CRLF3**	0	0	0	1	0	0	0	0	0	0	0	0	0	0	0	0	0	0	0	0	0	0	0	0	0	0	0	0
**SP1::CSF1**	0	0	0	0	0	0	0	0	0	0	1	0	0	0	0	0	0	0	0	0	0	0	0	0	0	0	0	0
**SP1::ELFN2**	0	0	0	0	0	0	0	0	0	0	0	1	0	0	0	0	0	0	0	0	0	0	0	0	0	0	0	0
**SP1::IMPG1**	0	0	0	0	0	0	0	0	0	0	0	0	0	0	0	0	0	0	0	0	0	0	0	1	0	0	0	0
**SP1::LAMC1**	0	0	0	0	0	0	0	0	0	0	0	1	0	0	0	0	0	0	0	0	0	0	0	0	0	0	0	0
**SP1::LAMC2**	0	0	0	0	0	0	0	0	0	0	0	0	0	0	0	0	0	0	0	0	0	1	0	0	0	0	0	0

**SP1::MUC3A**	0	0	0	0	0	0	0	0	0	0	1	1	0	0	0	0	0	0	0	0	0	0	0	0	0	0	0	0
**SP1::NELL2**	0	0	1	0	0	0	0	0	0	0	0	0	0	0	0	0	0	0	0	0	0	0	0	0	0	0	0	0
**SP1::PAPLN**	0	0	0	0	0	0	0	0	0	0	0	1	0	0	0	0	0	0	0	0	0	0	0	0	0	1	0	0
**SP1::PLXNA3**	0	0	0	0	0	0	1	0	0	0	0	0	0	0	0	0	0	0	0	0	0	0	0	0	0	0	0	0
**SP1::PLXNB1**	0	0	0	1	0	0	0	0	0	0	0	0	0	0	0	0	0	0	0	0	0	0	0	0	0	0	0	0
**SP1::SEMA6A**	0	0	0	0	0	0	0	0	0	0	1	1	0	0	0	0	0	0	0	0	0	0	0	0	0	0	0	0
**SP1::SPON2**	0	0	0	0	0	0	0	0	1	0	0	0	0	0	0	0	0	0	0	0	0	0	0	0	0	0	0	0
**SP1::VCAN**	0	0	0	0	0	0	0	1	0	0	0	0	0	0	0	0	0	0	0	0	0	0	0	0	0	0	0	0
**SP1::WNT2B**	0	0	0	0	0	0	0	0	1	0	0	0	0	0	0	0	0	0	0	0	0	0	0	0	0	0	0	0
**SP1::WNT5A**	0	0	0	0	0	0	0	0	0	0	0	1	0	0	0	0	0	0	0	0	0	0	0	0	0	0	0	0
**SP1::WNT9A**	0	0	0	0	0	0	0	0	0	0	0	0	0	0	0	0	0	1	0	0	0	0	0	0	0	0	0	0
**SP3::ADAMTS9**	0	0	0	0	0	0	0	1	0	0	0	0	0	0	0	0	0	0	0	0	0	0	0	0	0	0	0	0
**SP3::COL8A2**	0	0	0	0	0	0	0	0	1	0	0	0	0	0	0	0	0	0	0	0	0	0	0	0	0	0	0	0
**SP3::CRLF3**	0	0	0	1	0	0	0	0	0	0	1	0	0	0	0	0	0	0	0	0	0	0	0	0	0	0	0	0
**SP3::HPSE**	0	0	0	0	0	0	0	0	0	0	0	0	0	0	0	1	0	0	0	0	0	0	0	0	0	0	0	0
**SP3::LAMA5**	0	0	0	0	0	0	0	0	0	0	0	0	0	0	0	0	0	0	0	0	0	0	0	1	0	0	0	0
**SP3::LAMC1**	0	0	0	0	0	0	0	0	0	0	1	1	0	0	0	0	0	0	0	0	0	0	1	0	0	0	0	0
**SP3::LGALS8**	0	0	0	0	0	0	0	0	0	0	0	0	1	0	0	0	0	0	0	0	0	0	0	0	0	0	0	0
**SP3::LMAN1**	0	0	0	0	0	0	0	0	0	0	0	0	0	0	0	1	0	0	0	0	0	0	0	0	0	0	0	0
**SP3::MFAP1**	0	0	0	0	0	0	0	0	0	0	0	0	0	0	0	0	0	0	0	0	0	0	0	0	1	0	0	0
**SP3::NPNT**	0	0	0	0	0	0	0	0	1	0	0	0	0	0	0	0	0	0	0	0	0	0	0	0	0	0	0	0
**SP3::ST14**	0	0	0	0	0	0	0	0	0	0	0	0	0	0	0	1	0	0	0	0	0	0	0	0	0	0	0	0
**SP3::ZFP91**	0	0	0	0	0	0	0	0	0	0	0	0	1	0	0	0	0	0	0	0	0	0	0	0	0	0	0	0
**SP4::ADAMTSL2**	0	0	0	0	0	0	0	0	0	0	0	0	0	0	0	0	0	0	0	0	0	0	0	1	0	0	0	0
**SP4::CXCL9**	0	0	0	0	0	0	0	0	0	0	0	0	0	0	0	0	0	0	0	0	0	0	0	0	1	0	0	0
**SP4::GPC2**	0	0	0	0	0	0	0	0	0	0	0	0	0	1	0	0	0	0	0	0	0	0	0	0	0	0	0	0
**SP4::HTRA1**	0	0	0	0	0	0	0	0	0	0	0	0	0	0	0	0	0	0	0	1	0	0	0	0	0	0	0	0
**SP4::LAMA5**	0	0	0	0	0	0	1	0	0	0	0	0	0	0	0	0	0	0	0	0	0	0	0	0	0	0	0	0
**SP4::LTBP2**	0	0	0	0	0	0	0	0	1	0	0	0	0	0	0	0	0	0	0	0	0	0	0	0	0	0	0	0
**SP4::NID2**	0	0	0	0	0	0	0	0	0	0	0	0	0	0	0	0	0	1	0	0	0	0	0	0	0	0	0	0
**SP4::PLXNC1**	0	0	0	0	0	0	0	0	0	0	0	0	0	0	0	0	0	0	0	1	0	0	0	0	0	0	0	0
**SP4::RSPO2**	0	0	0	0	0	0	0	0	0	0	0	0	0	0	0	0	0	0	0	0	0	0	0	0	0	0	0	1
**SP4::SEMA6C**	0	0	0	0	0	0	0	0	0	0	0	0	0	0	0	0	0	0	0	1	0	0	0	0	0	0	0	0
**SP4::VCAN**	0	0	0	0	0	0	0	0	0	0	0	0	0	1	0	0	0	0	0	0	0	0	0	0	0	0	0	0
**SP8::IGFBP2**	0	0	0	0	0	0	0	0	0	0	0	0	0	0	0	0	1	0	0	0	0	0	0	0	0	0	0	0
**SPDEF::CXCL6**	0	0	0	0	0	0	0	0	0	0	0	0	0	0	0	0	1	0	0	0	0	0	0	0	0	0	0	0
**SPDEF::FNDC1**	0	0	1	0	0	0	0	0	0	0	0	0	0	0	0	0	0	0	0	0	0	0	0	0	0	0	0	0
**SPDEF::MUC1**	0	0	0	0	0	0	0	0	0	0	0	0	0	0	0	0	0	0	0	0	0	0	0	1	0	0	0	0
**SPDEF::RSPO4**	0	0	0	0	0	0	0	0	0	0	0	0	0	0	0	0	0	0	0	0	0	0	0	0	0	0	1	0
**SPDEF::SEMA4B**	0	0	0	0	0	0	0	0	0	0	0	0	0	0	0	1	0	0	0	0	0	0	0	0	0	0	0	0
**SPDEF::SEMA6B**	0	0	0	0	0	0	0	0	0	1	0	0	0	0	0	0	0	0	0	0	0	0	0	0	0	0	0	0
**SPI1::CXCL10**	0	1	0	0	0	0	0	0	0	0	0	0	0	0	0	1	1	0	0	0	0	0	0	0	0	0	0	0
**SPI1::LGALS9**	0	0	0	0	0	0	0	0	0	0	0	0	1	0	0	0	0	0	0	0	0	0	0	0	0	0	0	0
**SREBF1::MUC1**	0	1	1	0	0	0	0	0	0	0	0	0	0	0	0	0	0	0	0	0	0	0	0	0	0	0	0	0
**SREBF1::PLXNB2**	0	1	0	0	0	0	0	0	0	0	0	0	0	0	0	0	0	0	0	0	0	0	0	0	0	0	0	0
**SREBF1::WNT11**	0	0	0	0	0	0	1	0	0	0	0	0	0	0	0	0	0	0	0	0	0	0	0	0	0	0	0	0

**SREBF2::PLXNB2**	0	0	0	0	0	0	0	0	0	0	0	0	0	0	0	0	0	0	0	0	0	0	0	0	0	1	0	0
**SRF::ANGPT2**	0	0	0	0	0	0	1	0	0	0	0	0	0	0	0	0	0	0	0	0	0	0	0	0	0	0	0	0
**STAT5A::A2M**	0	0	0	0	0	0	0	0	0	0	0	0	0	1	0	0	0	0	0	0	0	0	0	0	0	0	0	0
**STAT5A::BMP4**	0	0	0	0	0	0	0	0	0	0	0	0	0	0	0	0	0	0	0	0	0	1	0	0	0	0	0	0
**STAT5A::C1QTNF1**	0	0	0	0	0	1	0	0	0	0	0	0	0	0	0	0	0	0	0	0	0	0	0	0	0	0	0	0
**STAT5A::C1QTNF5**	0	0	0	0	0	0	0	0	0	0	0	1	0	0	0	0	0	0	0	0	0	0	0	0	0	0	0	0
**STAT5A::CCL22**	0	0	0	1	0	0	0	0	0	0	0	0	0	0	0	0	0	0	0	0	0	0	0	0	0	0	0	0
**STAT5A::CCL5**	0	0	0	0	0	0	0	0	1	0	0	0	0	0	0	0	0	0	0	0	0	0	0	0	0	0	0	0
**STAT5A::CLEC4A**	0	0	0	0	0	0	0	0	0	0	0	0	0	0	0	0	0	0	0	0	0	0	0	0	1	0	0	0
**STAT5A::COL8A2**	0	0	0	0	0	0	0	0	1	0	0	0	0	0	0	0	0	0	0	0	0	0	0	0	0	0	0	0
**STAT5A::CXCL11**	0	0	0	0	0	0	1	0	0	0	0	0	0	0	0	0	0	0	0	0	0	1	0	0	0	0	0	0
**STAT5A::CXCL6**	0	0	0	0	0	0	0	0	0	0	0	1	0	0	0	0	0	0	0	0	0	0	0	0	0	0	0	0
**STAT5A::FGL2**	0	0	0	0	0	0	0	0	0	0	0	0	0	0	0	0	0	0	0	0	0	0	0	0	1	0	0	0
**STAT5A::HAPLN3**	0	0	0	0	0	0	0	0	1	0	0	0	0	0	0	1	1	0	0	0	0	0	0	0	0	0	0	1
**STAT5A::HPSE**	0	0	0	0	0	0	0	0	0	0	0	0	0	0	0	1	0	0	0	0	0	0	0	0	0	0	0	0
**STAT5A::HTRA4**	0	0	0	0	0	0	0	0	0	0	0	1	0	0	0	0	0	0	0	0	0	0	0	0	0	0	0	0
**STAT5A::LOXL3**	0	0	0	0	0	0	0	0	0	0	0	0	0	1	0	0	0	0	0	0	0	0	0	0	0	0	0	0
**STAT5A::MMP14**	0	0	0	0	0	0	0	0	0	0	0	0	0	0	0	1	0	0	0	0	0	0	0	0	0	0	0	0
**STAT5A::MMP28**	0	0	0	0	0	0	0	0	1	0	0	0	0	0	0	0	0	0	0	0	0	0	0	0	0	0	0	0
**STAT5A::NTNG2**	0	0	0	0	0	0	0	0	0	0	0	0	1	0	0	0	0	0	0	0	0	0	0	0	0	0	0	0
**STAT5A::OGFOD2**	0	0	0	0	0	0	0	0	0	0	0	1	0	0	0	0	0	0	0	0	0	0	0	0	0	0	0	0
**STAT5A::SERPING1**	0	0	0	0	0	0	0	0	0	0	1	0	0	0	0	0	0	0	0	0	0	0	0	0	0	0	0	0
**TAL1::ADAMTS7**	0	0	0	0	0	0	0	0	0	0	1	0	0	0	0	0	0	0	0	0	0	0	0	1	0	0	0	0
**TAL1::ANGPT2**	0	0	0	0	0	0	0	0	0	0	0	0	0	0	0	0	0	0	0	0	0	0	0	1	0	0	0	0
**TAL1::C1QTNF5**	0	0	0	0	0	0	0	0	0	0	1	0	0	0	0	0	0	0	0	0	0	0	0	0	0	0	0	0
**TAL1::COL4A1**	0	0	0	0	0	1	0	0	0	0	0	0	0	0	0	0	0	0	0	0	0	1	0	0	0	0	0	0
**TAL1::EGFL7**	0	0	0	0	0	0	0	0	0	0	1	0	0	0	0	0	0	0	0	0	0	0	0	0	0	1	0	0
**TAL1::FN1**	0	1	0	0	0	0	0	0	0	0	0	0	0	0	0	0	0	0	0	0	0	0	0	0	0	0	0	0
**TAL1::HPSE**	0	0	0	0	0	0	0	1	0	0	0	0	0	0	0	0	0	0	0	0	0	0	0	0	0	0	0	0
**TAL1::PLXDC1**	0	0	0	0	0	0	1	0	0	0	0	0	0	0	0	0	0	0	0	0	0	0	0	0	0	0	0	0
**TBX1::CLEC11A**	0	0	0	0	0	0	0	0	0	0	0	0	1	0	0	0	0	0	0	0	0	0	0	0	0	0	0	0
**TBX1::CSPG4**	0	0	0	0	0	0	0	0	1	0	0	0	0	0	0	0	0	0	0	0	0	0	0	0	0	0	0	0
**TBX1::MFAP2**	0	0	0	0	0	0	1	0	0	0	0	0	0	0	0	0	0	0	0	0	0	0	0	0	0	0	0	0
**TBX1::NTNG2**	1	0	0	0	0	0	0	0	0	0	0	0	0	0	0	0	0	0	0	0	0	0	0	0	0	0	0	0
**TBX1::SDC1**	0	1	0	0	0	0	0	0	0	0	0	0	0	0	0	0	0	0	0	0	0	0	0	0	0	0	0	0
**TBX15::ADAMTS6**	0	0	0	0	0	1	0	0	0	0	0	0	0	0	0	0	0	0	0	0	0	0	0	0	0	0	0	0
**TBX15::C1QTNF3**	0	0	0	0	0	0	0	0	0	0	0	1	0	0	0	0	0	0	0	0	0	0	0	0	0	0	0	0
**TBX15::HAPLN1**	0	0	0	0	0	0	0	0	0	0	1	0	0	0	0	0	0	0	0	0	0	0	0	0	1	0	0	0
**TBX19::ADAMTS10**	0	0	0	0	0	0	0	0	0	0	1	0	0	0	0	0	0	0	0	0	0	0	0	0	0	0	0	0
**TBX19::CBLN3**	0	0	0	0	0	0	0	0	0	0	1	0	0	0	0	1	0	0	0	0	0	0	0	0	0	0	0	0
**TBX19::PLXNA3**	0	0	0	0	0	0	0	0	0	0	0	0	0	0	0	1	0	0	0	0	0	0	0	0	0	0	0	0
**TBX2::C1QTNF5**	0	0	1	0	0	0	0	0	0	0	0	0	0	0	0	0	0	0	0	0	0	0	0	0	0	0	0	0
**TBX2::PLXDC1**	0	0	0	0	0	0	0	0	0	0	0	0	0	0	0	0	0	0	0	0	0	0	0	1	0	0	0	0
**TBX2::SEMA4C**	0	0	1	0	0	0	0	0	0	0	0	0	0	0	0	0	0	0	0	0	0	0	0	0	0	0	0	0
**TBX21::CXCL9**	0	0	0	0	0	0	0	0	0	0	0	0	0	0	0	0	0	0	0	0	0	0	0	0	1	0	0	0
**TBX21::FASLG**	0	0	0	0	0	0	0	0	0	0	0	0	0	0	0	0	0	0	0	0	0	0	0	1	0	0	0	0

**TBX21::SEMA3F**	0	0	0	1	0	0	0	0	0	0	0	0	0	0	0	0	0	0	0	0	0	0	0	0	0	0	0	0
**TBX4::VWCE**	0	0	0	0	0	0	0	0	0	0	0	0	1	0	0	0	0	0	0	0	0	0	0	0	0	0	0	0
**TCF12::ADAM15**	0	0	1	0	0	0	0	0	0	0	0	0	0	0	0	0	0	0	0	0	0	0	0	0	0	0	0	0
**TCF12::ADAMTS6**	0	0	0	0	0	0	0	0	0	0	0	0	0	1	0	0	0	0	0	0	0	0	0	0	0	0	0	0
**TCF12::BMP2**	0	0	0	0	0	0	0	0	0	0	0	0	0	0	0	0	1	0	0	0	0	0	0	0	0	0	0	0
**TCF12::LOXL1**	0	0	0	0	0	0	0	0	0	0	0	0	0	0	0	0	0	0	0	0	0	0	0	0	0	0	0	0
**TCF12::SEMA4C**	0	0	0	0	0	0	0	0	0	0	0	0	0	0	0	0	0	1	0	0	0	0	0	0	0	0	0	0
**TCF12::VCAN**	0	0	0	0	0	0	0	0	0	0	0	0	0	1	0	0	0	0	0	0	0	0	0	0	0	0	0	0
**TCF21::AGT**	0	0	0	0	0	0	0	0	0	0	0	0	0	0	0	0	0	0	0	0	0	0	0	1	0	0	0	0
**TCF3::ADAMTS7**	0	1	0	0	0	0	0	0	0	0	0	0	0	0	0	0	0	0	0	0	0	0	0	0	0	0	0	0
**TCF3::BMP1**	0	0	0	0	0	0	0	0	0	0	0	1	0	0	0	0	0	0	0	0	0	0	0	0	0	0	0	0
**TCF3::CILP2**	0	0	0	0	0	0	0	0	0	0	0	0	0	0	0	0	0	0	0	0	0	0	0	0	0	1	0	0
**TCF3::COL15A1**	1	0	0	0	0	0	0	0	0	0	0	0	0	0	0	0	0	0	0	0	0	0	0	0	0	0	0	0
**TCF3::CRLF1**	0	0	0	0	0	0	0	0	0	0	0	0	0	0	0	0	0	0	0	0	0	0	0	0	1	0	0	0
**TCF3::GPC2**	0	0	0	0	0	0	0	0	0	0	0	0	0	0	0	0	1	0	0	0	0	0	0	0	0	0	0	0
**TCF3::HYAL3**	0	0	0	0	0	0	0	0	0	0	0	0	1	0	0	0	0	0	0	0	0	0	0	0	0	0	0	0
**TCF3::LAMA5**	0	0	0	0	0	0	0	0	0	0	0	1	0	0	0	0	0	0	0	0	0	0	0	0	0	0	0	0
**TCF3::MFAP2**	0	0	1	0	0	0	0	0	0	0	0	0	0	0	0	0	0	0	0	0	0	0	1	0	0	0	0	0
**TCF3::MMRN2**	1	0	0	0	0	0	0	0	0	0	0	0	0	0	0	0	0	0	0	0	0	0	0	0	0	0	0	0
**TCF3::NID1**	0	0	0	0	0	0	0	1	0	0	0	0	0	0	0	0	0	0	0	0	0	0	0	0	0	0	0	0
**TCF3::OGFOD2**	0	0	0	0	0	0	0	0	0	0	0	0	1	0	0	0	0	0	0	0	0	0	0	0	0	0	0	0
**TCF3::PAPLN**	0	0	0	0	0	0	0	0	0	0	0	0	0	0	0	0	0	0	0	0	0	0	0	0	0	1	0	0
**TCF3::PLXNA3**	0	0	1	0	0	0	0	1	0	0	0	0	0	0	0	1	0	0	0	0	1	0	0	0	0	0	0	0
**TCF3::PLXNB1**	0	1	0	0	0	0	0	0	0	0	0	0	0	0	0	0	0	0	0	0	0	0	0	0	0	0	0	0
**TCF3::PLXNB3**	0	0	0	0	0	0	0	0	0	0	0	0	0	0	0	0	0	0	0	0	1	0	0	0	0	0	0	0
**TCF3::SEMA4C**	0	0	1	0	0	0	0	0	0	0	0	0	0	0	0	0	1	0	0	0	0	0	0	0	0	0	0	0
**TCF3::SEMA4D**	0	0	0	0	0	0	0	0	0	0	0	0	0	0	0	0	0	0	0	0	0	0	0	1	0	0	0	0
**TCF3::SEMA6C**	0	0	0	0	0	0	0	0	0	0	1	0	0	0	0	0	0	0	0	0	0	0	0	0	0	0	0	0
**TCF3::SERPINH1**	1	0	0	0	0	0	0	0	0	0	0	0	0	0	0	0	0	0	0	0	0	0	0	0	0	0	0	0
**TCF3::SMOC1**	0	0	0	0	0	0	0	0	0	1	0	0	0	0	0	0	0	0	0	0	0	0	0	0	0	0	0	0
**TCF3::SRGN**	0	0	0	0	0	0	0	0	0	0	0	0	1	0	0	0	0	0	0	0	0	0	0	0	0	0	0	0
**TCF3::ST14**	0	0	0	0	0	0	0	0	0	0	0	0	0	0	0	0	0	0	0	0	1	0	0	0	0	0	1	0
**TCF3::VWCE**	0	0	0	0	0	0	0	0	0	0	0	0	1	0	0	0	0	0	0	0	0	0	0	0	0	0	0	0
**TCF4::A2M**	0	0	0	0	0	0	0	0	0	0	1	0	0	0	0	0	0	0	0	0	0	0	0	0	0	0	0	0
**TCF4::ADAMTSL2**	0	0	0	0	0	0	0	0	0	0	0	0	0	0	0	0	0	0	0	0	0	0	0	0	0	1	0	0
**TCF4::C1QTNF5**	0	0	1	0	0	0	0	0	0	0	0	0	0	0	0	0	0	0	0	0	0	0	0	0	0	0	0	0
**TCF4::C1QTNF6**	0	0	0	0	0	0	0	0	0	0	0	0	0	0	0	1	0	0	0	0	0	0	0	0	0	0	0	0
**TCF4::CLEC14A**	0	0	0	0	0	0	0	0	0	0	1	0	0	0	0	0	0	0	0	0	0	0	0	0	0	0	0	0
**TCF4::COL15A1**	0	0	0	0	0	0	0	0	0	0	0	0	0	0	0	0	0	0	0	0	0	0	0	1	0	1	0	0
**TCF4::CTHRC1**	0	0	0	0	0	0	0	0	0	0	0	0	0	0	0	0	0	0	0	0	0	0	0	0	0	0	0	1
**TCF4::HSPG2**	0	0	0	0	0	0	0	0	0	0	1	0	0	0	0	0	0	0	0	0	0	0	0	0	0	0	0	0
**TCF4::PAMR1**	0	0	0	0	0	0	0	0	0	0	1	0	0	0	0	0	0	0	0	1	0	0	0	0	0	0	0	0
**TCF4::VCAN**	0	0	0	0	0	0	0	0	0	0	0	0	0	0	0	0	0	0	0	0	0	0	1	0	0	0	0	0
**TCF7::BMP4**	0	0	0	0	0	0	0	0	0	0	0	0	0	0	0	0	0	0	0	0	0	1	0	0	0	0	0	0
**TCF7::CTSV**	0	0	0	0	0	1	0	0	0	0	0	0	0	0	0	0	0	0	0	0	0	0	0	0	0	0	0	0
**TCF7::P4HA2**	0	0	0	0	0	0	0	0	1	0	0	0	0	0	0	0	0	0	0	0	0	0	0	0	0	0	0	0

**TCF7::TGFBI**	0	0	0	0	0	0	0	0	0	0	0	0	0	0	0	0	0	0	0	0	0	1	0	0	0	0	0	0
**TCF7::WISP1**	0	0	0	0	0	0	0	1	0	0	0	0	0	0	0	0	0	0	0	0	0	0	0	0	0	0	0	0
**TCF7L1::ADAMTS17**	0	0	0	0	0	0	0	0	0	0	0	0	0	0	0	0	0	0	0	0	0	0	0	0	0	0	0	1
**TCF7L1::BGN**	0	0	0	0	0	0	0	0	0	0	1	0	0	0	0	0	0	0	0	0	0	0	0	0	0	0	0	0
**TCF7L1::COL17A1**	0	0	0	0	0	0	0	0	0	0	0	0	0	0	0	0	0	0	0	0	0	0	0	0	1	0	0	0
**TCF7L1::EGFL6**	0	0	0	0	0	0	0	0	0	0	0	0	0	0	0	0	0	0	0	0	0	0	0	0	0	0	1	0
**TCF7L1::IGFBP2**	0	0	0	0	0	0	0	0	0	0	0	0	0	0	0	0	0	0	0	0	0	0	0	0	0	0	1	0
**TCF7L1::LTBP2**	0	0	0	0	0	0	0	0	1	0	0	0	0	0	0	0	0	0	0	0	0	0	0	0	0	0	0	0
**TCF7L1::SEMA4F**	0	0	0	0	0	0	0	0	0	0	0	0	0	0	0	0	0	0	0	0	0	0	0	0	0	0	1	0
**TCF7L1::VWCE**	0	0	0	0	0	0	0	0	0	0	0	0	0	1	0	0	0	0	0	0	0	0	0	0	0	0	0	0
**TCF7L2::ADAMTS7**	1	0	0	0	0	0	0	0	0	0	0	0	0	0	0	0	0	0	0	0	0	0	0	0	0	0	0	0
**TCF7L2::LAMA3**	0	0	0	0	0	0	0	0	0	0	0	0	0	0	0	0	0	0	0	0	0	0	0	1	0	0	0	0
**TEAD1::AGRN**	0	0	0	1	0	0	0	0	0	0	0	0	0	0	0	1	0	0	0	0	0	0	0	0	0	0	0	0
**TEAD1::BGLAP**	0	0	0	0	0	0	0	0	0	0	0	0	0	0	0	0	0	0	0	0	0	0	1	0	0	0	0	0
**TEAD1::COL4A1**	0	0	0	0	0	0	0	0	0	0	0	0	0	0	0	0	0	0	0	0	0	0	0	0	0	0	0	0
**TEAD1::COL4A2**	0	0	0	0	0	0	0	0	0	0	0	0	0	0	0	0	0	0	0	0	0	0	0	0	1	0	0	0
**TEAD1::CTHRC1**	0	0	0	0	0	0	0	0	0	0	0	0	0	0	0	0	1	0	0	0	0	0	0	0	0	0	0	0
**TEAD1::EGFL7**	0	0	0	0	0	0	0	0	0	0	0	0	0	0	0	0	0	0	0	0	0	0	0	0	0	1	0	0
**TEAD1::FGF2**	0	0	0	0	0	0	0	0	0	0	0	0	0	0	0	0	0	1	0	1	0	0	0	0	0	1	0	0
**TEAD1::HSPG2**	0	0	0	0	0	0	0	0	0	0	0	0	0	0	0	0	0	0	0	0	0	0	0	1	0	1	0	0
**TEAD1::LAMC1**	0	0	0	0	0	0	0	0	0	0	1	1	0	0	0	0	0	0	0	0	0	0	0	1	0	1	0	0
**TEAD1::LAMC2**	0	0	0	1	0	0	0	0	0	0	0	0	0	0	0	0	0	0	0	0	0	0	0	0	0	0	0	0
**TEAD1::LOXL2**	0	0	1	0	0	0	0	0	0	0	0	0	0	0	0	0	0	0	0	0	0	0	0	0	0	0	0	0
**TEAD1::PLAU**	0	0	0	0	0	0	0	0	0	0	0	0	0	0	0	1	0	0	0	0	0	0	0	0	0	0	0	0
**TEAD1::PLXNB2**	0	0	0	0	0	0	0	0	0	0	0	0	0	0	0	0	0	0	0	0	0	0	0	0	0	1	0	0
**TEAD1::SDC2**	0	0	0	0	0	0	0	0	0	0	0	0	0	0	0	0	0	0	0	0	0	0	0	0	1	0	0	0
**TEAD1::TNC**	0	0	0	0	0	0	0	0	1	0	0	0	0	0	0	1	0	0	0	0	0	0	0	0	0	0	0	0
**TEAD1::VCAN**	0	0	0	0	0	0	1	0	0	0	0	0	0	0	0	0	0	0	0	0	0	0	0	0	0	0	0	0
**TEAD1::VWA7**	0	0	0	0	0	0	0	0	0	0	0	0	0	0	0	0	0	0	0	0	0	0	0	0	0	1	0	0
**TEAD3::ADAMTS17**	0	0	0	0	0	0	0	0	0	0	0	0	0	0	0	0	1	0	0	0	0	0	0	0	0	0	0	0
**TEAD3::C1QTNF1**	1	0	0	0	0	0	0	0	0	0	0	0	0	0	0	0	0	0	0	0	0	0	0	0	0	0	0	0
**TEAD3::COL13A1**	0	0	0	0	0	0	0	0	0	0	0	0	0	0	0	0	0	0	0	0	0	0	0	0	1	0	0	0
**TEAD3::CSF1**	0	0	0	0	0	0	0	0	0	0	1	0	0	0	0	0	0	0	0	0	0	0	0	0	0	0	0	0
**TEAD3::EFEMP2**	0	0	0	0	0	0	0	1	0	0	0	0	0	0	0	0	0	0	0	0	0	0	0	0	0	0	0	0
**TEAD3::HSPG2**	0	0	0	0	0	0	0	0	0	0	0	0	0	0	0	0	0	0	0	0	0	0	0	1	0	0	0	0
**TEAD3::LAMC1**	0	0	0	0	1	0	0	0	0	0	0	0	0	0	0	0	0	0	0	0	0	0	0	0	0	0	0	0
**TEAD3::MMP3**	0	0	1	0	0	0	0	0	0	0	0	0	0	0	0	0	0	0	1	0	0	0	0	0	0	0	0	0
**TEAD3::SPOCK2**	0	0	0	0	0	0	0	0	0	0	0	0	0	0	0	0	0	0	0	0	0	0	0	0	0	1	0	0
**TEAD3::TINAGL1**	0	1	0	0	0	0	0	0	0	0	0	0	0	0	0	0	0	0	0	0	0	0	0	0	0	0	0	0
**TEAD3::WNT11**	0	0	0	0	0	0	0	0	0	0	0	0	0	0	0	0	0	0	0	0	0	0	0	0	1	0	0	0
**TEAD3::VWA1**	0	0	0	0	0	0	0	0	0	0	0	0	0	0	0	1	0	0	0	0	0	0	0	1	0	0	0	0
**TEAD4::ADAM15**	0	1	1	0	0	0	0	0	0	0	0	0	0	0	0	0	0	0	0	0	0	0	0	0	0	0	0	0
**TEAD4::ADAMTSL5**	0	0	0	0	0	0	0	0	0	0	0	0	0	0	0	0	0	0	0	0	0	1	0	0	0	0	0	0
**TEAD4::ANXA11**	0	0	0	0	1	0	0	0	0	0	0	0	0	0	0	0	0	0	0	0	0	0	0	0	0	0	0	0
**TEAD4::COL4A1**	0	0	0	0	0	1	0	0	0	0	0	0	0	0	0	0	0	0	0	1	0	0	0	0	0	0	0	0
**TEAD4::FNDC1**	0	0	1	0	0	0	0	0	0	0	0	0	0	0	0	0	0	0	0	0	0	0	0	0	0	0	0	0

**TEAD4::LAMC2**	0	1	0	0	1	0	0	0	0	0	0	0	0	0	0	0	0	0	0	0	0	0	0	0	0	0	0	0
**TEAD4::LGALS1**	0	0	0	0	0	0	0	0	0	0	0	0	0	0	0	0	0	0	0	0	0	0	1	0	0	0	0	0
**TEAD4::LGALS9**	0	0	0	0	1	0	0	0	0	0	0	0	0	0	0	0	0	0	0	0	0	0	0	0	0	0	0	0
**TEAD4::LOXL2**	0	0	0	0	0	0	0	0	0	0	0	0	0	0	0	0	0	0	0	0	0	1	0	0	0	0	0	0
**TEAD4::MFAP2**	0	1	0	0	0	0	0	0	0	0	0	0	0	0	0	0	0	0	0	0	0	0	0	0	0	0	0	0
**TEAD4::PLAU**	0	1	0	0	0	0	0	0	0	0	0	0	0	0	0	0	0	0	0	0	0	0	0	0	0	0	0	0
**TEAD4::SERPINA3**	0	0	0	0	0	0	0	1	0	0	0	0	0	0	0	0	0	0	0	0	0	0	0	0	0	0	0	0
**TEAD4::SERPINA5**	1	0	0	0	0	0	0	0	0	0	0	0	0	0	0	0	0	0	0	0	0	0	0	0	0	0	0	0
**TEAD4::SERPINE1**	0	0	0	0	0	0	0	0	0	0	1	0	0	0	0	0	0	0	0	0	0	0	0	1	0	0	0	0
**TEAD4::SERPINH1**	0	0	0	0	0	0	1	0	0	0	0	0	0	0	0	0	0	0	0	0	0	0	0	0	0	0	0	0
**TEAD4::VWCE**	0	0	0	0	0	0	0	0	0	0	0	0	1	0	0	0	0	0	0	0	0	0	0	0	0	0	0	0
**TEAD4::ZP3**	0	1	0	0	0	0	0	0	0	0	0	0	0	0	0	0	0	0	0	0	0	0	0	0	0	0	0	0
**TFAP2A::ADAMTSL5**	0	0	0	0	0	0	0	0	0	0	0	0	0	0	0	1	1	0	0	0	0	0	0	0	0	0	0	0
**TFAP2A::ANXA1**	0	0	0	1	0	0	0	0	0	0	0	0	0	0	0	0	0	0	0	0	0	0	0	0	0	0	0	0
**TFAP2A::ANXA2**	0	0	0	0	0	0	0	0	0	0	0	0	0	0	0	0	0	0	0	0	0	0	0	1	0	0	0	0
**TFAP2A::ANXA5**	0	0	0	0	0	0	0	0	0	0	0	0	0	0	0	0	0	0	0	0	1	0	0	0	0	0	0	0
**TFAP2A::C1QTNF6**	0	0	0	0	0	0	0	0	0	0	0	0	0	0	0	0	1	0	0	0	0	0	0	0	0	0	0	0
**TFAP2A::COL13A1**	0	0	0	0	0	0	0	0	1	0	0	0	0	0	0	0	0	0	0	0	0	0	0	0	0	0	0	0
**TFAP2A::COL5A2**	0	0	0	0	0	0	0	0	0	0	0	0	0	0	0	0	1	0	0	0	0	0	0	0	0	0	0	0
**TFAP2A::FGF11**	0	0	0	0	0	0	0	0	0	0	0	0	0	0	0	0	1	0	0	0	0	0	0	0	0	0	0	0
**TFAP2A::GREM1**	0	0	0	0	0	0	0	0	0	0	0	0	0	0	0	0	1	0	0	0	0	0	0	0	0	0	0	0
**TFAP2A::LAMC2**	0	1	0	0	0	0	0	0	0	0	0	0	0	0	0	0	0	0	0	0	0	0	0	0	0	0	0	0
**TFAP2A::MMP17**	0	0	0	0	0	0	0	0	0	0	0	0	0	0	0	0	0	0	0	1	0	0	0	0	0	0	0	0
**TFAP2A::MUC1**	0	0	1	0	0	0	0	0	0	0	0	0	0	0	0	0	0	0	0	0	0	0	0	1	0	0	0	0
**TFAP2A::P4HA3**	0	0	0	0	0	0	0	0	0	0	0	0	0	0	0	0	0	0	0	0	1	0	0	0	0	0	0	0
**TFAP2A::POSTN**	0	0	1	0	0	0	0	0	0	0	0	0	0	0	0	0	0	0	0	0	0	0	0	0	0	0	0	0
**TFAP2A::SDC1**	0	0	0	0	0	0	1	0	0	0	0	0	0	0	0	0	0	0	0	0	0	0	0	0	0	0	0	0
**TFAP2A::SEMA3F**	0	1	1	1	0	0	0	0	0	0	0	0	0	0	0	0	0	0	0	0	0	0	0	0	0	0	0	0
**TFAP2A::SEMA4A**	0	0	1	0	0	0	0	0	0	0	0	0	0	0	0	0	0	0	0	0	0	0	0	0	0	0	0	0
**TFAP2A::SEMA4B**	0	0	0	0	0	0	0	0	0	0	0	0	0	0	0	1	0	0	0	0	0	0	0	0	0	0	0	0
**TFAP2A::SEMA4C**	0	0	1	0	0	0	0	0	0	0	0	0	0	0	0	0	1	0	0	0	0	0	0	0	0	0	0	0
**TFAP2B::ELN**	0	0	0	0	0	0	0	0	0	0	0	0	0	0	0	0	0	0	0	1	0	0	0	0	0	0	0	0
**TFAP2B::PLXNC1**	0	0	0	0	0	0	0	0	0	0	0	0	0	0	0	0	0	0	0	1	0	0	0	0	0	0	0	0
**TFAP2B::PRSS3**	0	0	0	0	0	0	0	0	0	0	0	0	0	0	0	0	0	0	0	1	0	0	0	0	0	0	0	0
**TFAP2B::SLIT3**	0	0	0	0	0	0	0	0	0	0	0	0	0	0	0	0	0	0	0	1	0	0	0	0	0	0	0	0
**TFAP2C::ANXA1**	0	0	0	0	0	0	0	0	0	0	0	0	0	0	0	0	0	0	0	0	0	0	0	0	0	1	0	0
**TFAP2C::COL8A2**	0	0	0	0	0	0	0	0	0	0	0	0	0	0	0	0	0	0	0	0	0	0	0	0	0	1	0	0
**TFAP2C::LOXL3**	0	0	0	0	0	0	0	0	0	0	0	0	0	0	0	0	0	0	0	1	0	0	0	0	0	0	0	0
**TFAP2C::PAPPA**	0	0	0	0	0	0	0	0	0	0	0	0	0	0	0	0	0	0	0	0	0	0	0	0	0	1	0	0
**TFAP2C::PLXNA3**	0	0	0	0	0	0	0	0	0	0	0	0	0	0	0	0	0	0	0	0	1	0	0	0	0	0	0	0
**TFAP2C::SDC1**	0	0	1	0	0	0	0	0	0	0	0	0	0	0	0	0	0	0	0	0	0	0	0	0	0	0	0	0
**TFAP2C::SDC4**	0	0	1	1	0	0	0	0	0	0	0	0	0	0	0	0	0	0	0	0	0	0	0	0	0	0	0	0
**TFAP2C::SEMA4A**	0	1	0	1	0	0	0	0	0	0	0	0	0	0	0	0	0	0	0	0	0	0	0	0	0	0	0	0
**TFAP2C::TSKU**	0	0	0	0	0	0	0	0	1	0	0	0	0	0	0	0	0	0	0	0	0	0	0	0	0	0	0	0
**TFAP2C::WNT5A**	0	0	0	0	0	0	0	0	0	0	0	0	0	0	0	0	1	0	0	0	0	0	0	0	0	1	0	0
**TFAP4::ADAMTSL4**	0	0	0	0	0	0	0	0	0	0	1	0	0	0	0	0	0	0	0	0	0	0	0	0	0	0	0	0

**TFAP4::ANGPTL2**	0	0	0	0	0	0	0	0	0	0	0	0	0	1	0	0	0	0	0	0	0	0	0	0	0	0	0	0
**TFAP4::COL18A1**	0	0	0	0	0	0	0	0	0	0	0	0	0	0	0	0	0	0	0	0	0	0	0	0	0	0	1	0
**TFAP4::COL27A1**	1	0	0	0	0	0	0	0	0	0	0	0	0	0	0	0	0	0	0	0	0	0	0	0	0	0	0	0
**TFAP4::CRLF3**	0	1	0	0	0	0	0	0	0	0	0	0	0	0	0	0	0	0	0	0	0	0	0	0	0	0	0	0
**TFAP4::CXCL3**	0	0	0	0	0	1	0	0	0	0	0	0	0	0	0	0	0	0	0	0	0	0	0	0	0	0	0	0
**TFAP4::GAS6**	0	0	0	0	0	0	0	0	0	1	0	0	0	0	0	0	0	0	0	0	0	0	0	0	0	0	0	0
**TFAP4::GPC2**	0	1	0	0	0	0	0	1	0	0	0	0	0	0	0	0	1	0	0	0	0	0	0	0	0	0	0	0
**TFAP4::HYAL3**	0	0	0	0	0	0	0	0	0	0	0	0	0	0	0	0	0	0	0	0	1	0	0	0	0	0	0	0
**TFAP4::LOXL2**	0	0	0	0	0	0	0	0	0	0	1	0	0	0	0	0	0	0	0	0	0	0	0	0	0	0	0	0
**TFAP4::MUC1**	0	1	0	0	0	0	0	0	0	0	0	0	0	0	0	0	0	0	0	0	0	0	0	0	0	0	0	0
**TFAP4::NTN3**	0	0	0	0	0	0	0	0	0	0	0	0	0	0	0	0	1	0	0	0	0	0	0	0	0	0	0	1
**TFAP4::OGFOD2**	0	1	0	0	0	0	0	0	0	1	0	1	0	0	0	0	0	0	0	0	1	0	0	0	0	0	0	0
**TFAP4::PLOD1**	0	0	0	0	0	0	0	0	0	0	1	0	0	0	0	0	0	0	0	0	0	0	0	0	0	0	0	0
**TFAP4::PODN**	0	0	0	0	0	0	0	0	0	0	0	0	0	1	0	0	0	0	0	0	0	0	0	0	0	0	0	0
**TFAP4::WNT11**	1	0	0	0	0	0	0	0	0	0	0	0	0	0	0	0	0	0	0	0	0	0	0	0	0	0	0	0
**TFAP4::WNT8B**	0	0	0	0	0	1	0	0	0	0	0	0	0	0	0	0	0	0	0	0	0	0	0	0	0	0	0	0
**TFAP4::ZP3**	0	0	0	0	0	0	0	0	0	0	0	0	0	0	0	0	0	0	0	0	0	1	0	0	0	0	0	0
**TFCP2::BTC**	0	0	0	0	0	0	0	0	0	0	0	0	0	0	0	0	0	0	1	0	0	0	0	0	0	0	0	0
**TFCP2::SEMA4F**	0	0	0	0	0	0	0	0	0	0	0	0	0	0	0	0	0	0	0	1	0	0	0	0	0	0	0	0
**TFEB::EGLN2**	0	0	0	0	0	0	0	0	0	1	0	0	0	0	0	0	0	0	0	0	0	0	0	0	0	0	0	0
**TFEB::SEMA4A**	0	0	0	0	0	0	0	0	0	0	0	0	0	0	0	0	0	0	0	0	0	0	0	0	1	0	0	0
**TP53::ELN**	0	0	0	0	0	0	0	1	0	0	0	0	0	0	0	0	0	0	0	0	0	0	0	0	0	0	0	0
**TP53::GAS6**	0	0	0	0	0	0	0	0	0	0	0	0	0	0	0	0	0	0	0	1	0	0	0	0	0	0	0	0
**TP53::MUC2**	0	0	0	0	0	0	0	0	0	0	0	0	0	0	0	0	0	0	0	0	1	0	0	0	0	0	0	0
**TP53::THBS3**	0	0	0	0	1	0	0	0	0	0	0	0	0	0	0	0	0	0	0	0	0	0	0	0	0	0	0	0
**TP53::VWCE**	0	0	0	0	0	0	0	0	0	0	1	0	0	0	0	0	0	0	0	0	0	0	0	0	0	0	0	0
**TTF1::CRLF3**	0	0	0	0	0	0	0	0	0	0	0	0	0	0	0	0	0	0	0	0	0	0	0	1	0	0	0	0
**TTF1::IMPG1**	0	0	0	0	0	0	0	0	0	0	0	0	0	0	0	0	0	0	0	0	1	0	0	0	0	0	0	0
**TTF1::OGFOD1**	0	0	0	0	0	0	0	0	0	0	0	0	0	0	0	0	0	0	0	0	0	0	0	1	0	0	0	0
**USF1::CRELD2**	0	0	0	0	0	0	0	0	0	0	0	0	0	0	1	0	0	0	0	0	0	0	0	0	0	0	0	0
**USF1::EGLN2**	0	0	1	0	0	0	0	0	0	0	0	0	0	0	1	0	0	0	0	0	0	0	0	0	0	0	0	0
**USF1::SEMA4A**	0	0	1	1	0	0	0	0	0	0	0	0	0	0	0	0	0	0	0	0	0	0	0	0	0	0	0	0
**VAX2::ADAMTS17**	0	0	0	0	0	0	0	0	0	0	0	0	0	0	0	0	1	0	0	0	0	0	0	0	0	0	0	0
**VAX2::C1QL1**	0	0	0	0	0	0	0	1	0	0	0	0	0	0	1	0	0	0	0	0	0	0	0	0	0	0	0	0
**VAX2::EFEMP2**	0	0	0	0	0	0	0	0	0	0	0	0	0	0	0	0	0	1	0	0	0	0	0	0	0	0	0	0
**VAX2::GPC2**	0	0	0	0	0	0	0	0	0	0	0	0	0	0	1	0	0	0	0	0	0	0	0	0	0	0	0	0
**VAX2::MMP14**	0	0	0	0	0	0	0	0	0	0	0	0	0	0	0	0	0	1	0	0	0	0	0	0	0	0	0	0
**VDR::COL24A1**	0	0	0	0	0	0	0	0	0	0	0	0	0	0	0	1	0	0	0	0	0	0	0	0	0	0	0	0
**VDR::COL7A1**	0	1	0	0	0	0	0	0	0	0	0	0	0	0	0	0	0	0	0	0	0	0	0	0	0	0	0	0
**VDR::CSF1**	0	0	0	0	0	0	0	1	0	0	0	0	0	0	0	0	0	0	0	0	0	0	0	0	0	0	0	0
**VDR::ECM1**	0	0	0	0	0	0	0	0	0	0	0	0	0	0	0	0	0	0	0	0	0	0	0	0	0	1	0	0
**VDR::F13A1**	0	0	0	0	0	0	0	1	0	0	0	0	0	0	0	0	0	0	0	0	0	0	0	0	0	0	0	0
**VDR::FGF13**	0	0	0	0	0	0	0	0	0	0	0	0	0	0	0	0	0	0	0	0	0	0	0	0	1	0	0	0
**VDR::HAPLN3**	0	0	0	0	0	0	0	0	0	0	0	0	0	0	0	0	0	0	0	0	0	0	1	0	0	0	0	0
**VDR::MMP13**	0	1	0	0	0	0	0	0	0	0	0	0	0	0	0	0	0	0	0	0	0	0	0	0	0	0	0	0
**VDR::PLXNB1**	0	0	0	0	0	0	0	0	0	0	0	0	0	0	0	0	0	0	0	0	0	0	0	0	0	1	0	0

VDR::SEMA4A	0	1	1	0	0	0	0	0	0	0	0	0	0	0	0	0	0	0	0	0	0	0	0	0	0	0	0	0
VDR::SERPINB8	0	0	0	0	0	0	0	0	0	0	0	0	0	0	0	0	0	0	0	0	0	0	0	0	0	1	0	0
VDR::ST14	0	1	1	1	0	0	1	0	0	0	0	0	0	0	0	0	0	0	0	0	0	0	0	1	0	0	0	0
VDR::TINAGL1	0	0	0	0	1	0	0	0	1	0	0	0	0	0	0	0	0	0	0	0	0	0	0	0	0	0	0	0
VDR::VCAN	0	0	0	0	0	0	0	0	0	0	0	1	0	0	0	0	0	0	0	0	0	0	0	0	0	0	0	0
WT1::FMOD	0	0	0	0	0	0	0	0	0	0	0	0	0	0	0	0	0	1	0	0	0	0	0	0	0	0	0	0
WT1::SULF1	0	0	0	0	0	0	0	0	0	0	0	0	0	0	0	0	0	1	0	0	0	0	0	0	0	0	0	0
XBP1::ANXA9	0	0	1	0	0	0	0	0	0	0	0	0	0	0	0	0	0	0	0	0	0	0	0	0	0	0	0	0
XBP1::CCL2	0	0	0	0	0	0	0	0	0	0	0	0	0	0	0	0	0	1	0	0	0	0	0	0	0	0	0	0
XBP1::CCL24	0	0	0	0	0	1	0	0	0	0	0	0	0	0	0	0	0	0	0	0	0	1	0	0	1	0	0	0
XBP1::CHRD	0	0	1	0	0	0	0	0	0	0	0	0	0	0	0	0	0	0	0	0	0	0	0	0	0	0	0	0
XBP1::CRELD2	0	0	0	0	0	0	0	0	0	0	0	0	0	0	0	1	0	0	0	0	0	0	0	0	0	0	0	0
XBP1::EGF	0	0	0	0	0	0	0	0	0	0	0	0	0	0	0	0	0	0	0	0	1	0	0	0	0	0	0	0
XBP1::FMOD	0	0	0	0	0	0	0	0	0	0	0	0	0	0	0	0	0	0	0	0	1	0	0	0	0	0	0	0
XBP1::LGALS1	0	0	0	0	0	0	0	1	0	0	0	0	0	0	0	0	0	0	0	0	0	0	0	0	0	0	0	0
XBP1::LRG1	0	0	1	0	0	0	0	0	0	0	0	0	0	0	0	0	0	0	0	0	0	0	0	0	0	0	0	0
XBP1::MFAP2	0	0	1	0	0	0	0	0	0	0	0	0	0	0	0	0	0	0	0	0	0	0	0	0	0	0	0	0
XBP1::SDC4	0	0	1	0	0	0	0	0	0	0	0	0	0	0	0	0	0	0	0	0	0	0	0	0	0	0	0	0
XBP1::SERPINH1	0	0	1	0	0	0	0	0	0	0	0	0	0	0	0	0	0	0	0	0	0	0	0	0	0	0	0	0
XBP1::WNT11	0	0	0	0	0	1	0	0	0	0	0	0	0	0	0	0	0	0	0	0	0	0	0	0	0	0	0	0
YY1::LGALS9	0	0	0	0	0	0	0	0	0	0	0	0	0	0	0	0	0	0	0	0	0	0	0	0	0	0	1	0
YY1::P4HTM	0	0	0	0	0	0	0	0	0	0	0	0	0	0	0	0	0	0	0	0	0	0	0	0	0	1	0	0
YY1::WNT2B	0	0	0	0	0	0	0	0	1	0	0	0	0	0	0	0	0	0	0	0	0	0	0	0	0	0	0	0
ZBTB33::ANGPT1	0	0	0	0	0	0	0	0	0	1	0	0	0	0	0	0	0	0	0	0	0	0	0	0	0	0	0	0
ZBTB33::CXCL5	0	0	0	0	0	0	0	0	0	0	0	0	0	0	0	0	0	0	0	0	0	0	0	0	0	0	1	0
ZBTB33::PLXNA3	0	0	0	0	0	0	0	0	0	0	0	0	0	0	0	0	0	0	0	0	0	0	0	0	0	1	0	0
ZBTB49::ADAMTS7	1	0	0	0	0	0	0	0	0	0	0	0	0	0	0	0	0	0	0	0	0	0	1	0	0	0	0	0
ZBTB6::LMAN1	0	0	1	0	0	0	0	0	0	0	0	0	0	0	0	0	0	0	0	0	0	0	0	0	0	0	0	0
ZBTB6::PLXNC1	0	0	1	0	0	0	0	0	0	0	0	0	0	0	0	0	0	0	0	0	0	0	0	0	0	0	0	0
ZBTB7A::ADAMTSL5	0	0	0	0	0	0	0	0	0	0	0	0	0	0	0	1	1	0	0	0	0	0	0	0	0	0	0	0
ZBTB7A::CBLN3	0	0	0	0	0	0	0	0	0	0	0	0	0	0	0	0	0	0	0	0	1	0	0	0	0	0	0	0
ZBTB7A::CILP2	0	1	0	0	0	0	0	0	0	0	0	0	0	0	0	0	0	0	0	0	0	0	0	0	0	0	0	0
ZBTB7A::GPC2	0	0	0	0	0	0	0	0	0	0	0	0	0	0	0	0	1	0	0	0	0	0	0	0	0	0	1	0
ZBTB7A::LAMA5	0	0	0	0	0	0	0	0	0	1	0	0	0	0	0	0	0	0	0	0	0	0	0	0	0	0	0	0
ZBTB7A::MMP17	0	0	1	0	0	0	0	0	0	0	0	0	0	0	0	0	0	0	0	0	0	0	0	0	0	0	0	0
ZBTB7A::NTN3	0	0	0	0	0	0	0	0	0	0	0	0	0	0	0	0	0	0	0	1	0	0	0	0	0	0	0	0
ZBTB7A::PLOD2	0	0	0	0	0	0	0	0	0	0	0	0	0	0	0	0	1	0	0	0	0	0	0	0	0	0	0	0
ZBTB7A::PRSS1	0	0	0	0	0	0	0	0	0	0	0	0	0	0	0	0	0	0	0	0	0	0	0	0	0	0	1	0
ZBTB7B::ADAM15	0	1	1	0	0	0	0	0	0	0	0	0	0	0	0	1	0	1	1	0	1	0	0	0	0	0	0	0
ZBTB7B::COL17A1	0	0	0	1	0	0	0	0	0	0	0	0	0	0	0	0	0	0	0	0	0	0	0	0	0	0	0	0
ZBTB7B::ESM1	0	0	0	0	0	0	0	0	0	0	0	0	0	0	0	0	0	0	0	0	0	0	0	0	0	0	1	0
ZBTB7B::FAM20B	0	0	0	0	0	0	0	0	0	0	0	0	0	0	0	0	1	0	0	0	0	0	0	0	0	0	0	0
ZBTB7B::LAMC1	0	0	0	0	0	0	0	0	0	0	0	0	0	0	0	0	0	0	0	0	0	0	1	0	0	1	0	0
ZBTB7B::LRG1	0	0	1	0	0	0	0	0	0	0	0	0	0	0	0	0	0	0	0	0	0	0	0	0	0	0	0	0
ZBTB7B::MUC1	0	1	1	0	0	0	0	0	0	0	0	0	0	0	0	0	0	0	1	0	0	0	0	0	0	0	1	0
ZBTB7B::P4HTM	0	0	0	0	0	0	0	0	0	0	0	0	0	0	0	0	0	0	0	0	0	0	0	0	0	1	0	0

**ZBTB7B::PLOD1**	0	0	0	0	0	0	0	0	0	0	1	0	0	0	0	0	0	0	0	0	0	0	0	0	0	0	0	0
**ZBTB7B::PLXNB3**	0	0	0	0	0	0	0	0	0	0	0	0	0	0	0	0	1	0	0	0	0	0	0	0	0	0	0	0
**ZBTB7B::SDC1**	0	0	0	0	0	0	0	0	0	0	0	0	0	0	0	0	0	0	0	0	0	0	0	1	0	0	0	0
**ZBTB7B::SEMA4A**	0	1	0	0	0	0	0	0	0	0	0	0	0	0	0	0	0	0	0	0	0	0	0	0	0	0	0	0
**ZBTB7B::ST14**	0	0	0	0	0	0	0	0	0	0	0	0	0	0	0	0	1	0	0	0	1	0	0	0	0	0	0	0
**ZBTB7C::C1QTNF6**	0	0	0	0	0	0	0	0	0	0	0	0	0	0	0	0	1	0	0	0	0	0	0	0	0	0	0	0
**ZBTB7C::ST14**	0	0	0	0	0	0	0	0	0	0	0	0	0	0	0	0	0	0	0	0	0	0	0	0	0	1	0	0
**ZEB1::ADAMTS14**	0	0	0	0	0	0	0	0	1	0	0	0	0	0	0	0	0	0	0	0	0	0	0	0	0	0	0	0
**ZEB1::AEBP1**	0	0	1	0	0	0	0	0	0	0	0	0	0	0	0	0	0	0	0	0	0	0	0	0	0	0	0	0
**ZEB1::ANGPTL2**	0	0	0	0	0	0	0	0	0	0	1	0	0	0	0	0	0	0	0	0	0	0	0	0	0	0	0	0
**ZEB1::COL1A1**	0	0	1	0	0	0	0	0	0	0	0	0	0	0	0	0	0	0	0	0	0	0	0	0	0	0	0	0
**ZEB1::COL6A3**	0	0	1	0	0	1	1	0	1	0	0	0	0	0	0	0	0	0	0	0	0	0	0	0	0	0	0	0
**ZEB1::COL8A1**	0	0	0	0	0	0	0	0	0	0	0	0	0	0	0	0	0	0	0	0	1	0	0	0	0	0	0	0
**ZEB1::FGF2**	0	0	0	0	0	0	0	0	0	0	0	0	0	0	0	0	0	0	0	1	0	0	0	0	0	0	0	0
**ZEB1::IGFBP7**	0	0	0	0	0	0	0	0	0	0	0	0	0	0	0	0	0	0	0	0	0	0	0	1	0	0	0	0
**ZEB1::LAMA4**	0	0	0	0	0	0	0	0	0	0	0	0	0	0	0	0	0	0	0	0	0	0	1	0	0	0	0	0
**ZEB1::MMP11**	0	0	0	1	0	0	0	0	0	0	0	0	0	0	0	0	0	0	0	0	0	0	0	0	0	0	0	0
**ZEB1::MMP19**	0	0	0	0	0	0	0	0	1	0	0	0	0	0	0	0	0	0	0	0	0	0	0	0	0	0	0	0
**ZEB1::NTNG2**	0	0	0	0	0	0	0	0	0	0	0	0	1	0	0	0	0	0	0	0	0	0	0	0	0	0	0	0
**ZEB1::P4HA3**	0	0	0	0	0	0	0	0	0	0	0	0	0	0	0	0	0	0	0	0	1	0	0	0	0	0	0	0
**ZEB1::SEMA6D**	0	0	0	0	0	0	0	0	0	0	0	0	0	0	0	0	0	0	0	1	0	0	0	0	0	0	0	0
**ZEB1::SEMA7A**	0	0	0	0	0	0	0	0	0	0	0	0	0	0	0	1	0	0	0	0	0	0	0	0	0	0	0	0
**ZEB1::ST14**	0	0	1	0	0	0	0	0	0	0	0	0	0	0	0	0	1	0	0	0	0	0	0	0	0	0	0	0
**ZEB1::THBS1**	0	0	0	0	0	0	0	0	0	0	0	0	0	0	0	0	0	0	0	0	0	0	0	1	0	0	0	0
**ZFX::PLXNA2**	0	0	0	0	0	0	0	0	0	0	1	0	0	0	0	0	0	0	0	0	0	0	0	0	0	0	0	0
**ZFX::PLXNA3**	0	0	0	0	0	0	0	0	0	0	0	0	0	0	0	0	0	0	0	0	0	0	0	1	0	0	0	0
**ZIC2::ADAMTSL5**	0	0	0	0	0	0	0	0	0	0	0	0	0	0	0	1	0	0	0	0	0	0	0	0	0	0	0	0
**ZIC2::C1QTNF6**	0	0	0	0	0	0	0	0	1	0	0	0	0	0	0	0	0	0	0	0	0	0	0	0	0	0	0	0
**ZIC2::CTHRC1**	0	0	0	0	0	0	0	0	1	0	0	0	0	0	0	0	0	0	0	0	0	0	0	0	0	0	0	0
**ZIC3::LAMA1**	0	0	0	0	0	0	0	0	0	0	0	0	0	0	0	0	1	0	0	0	0	0	0	0	0	0	0	0
**ZNF143::COL15A1**	1	0	0	0	0	0	0	0	0	0	0	0	0	0	0	0	0	0	0	0	0	0	0	0	0	0	0	0
**ZNF143::CRLF3**	0	0	0	0	0	0	0	0	1	0	0	0	0	0	0	0	0	0	0	0	0	0	0	0	0	0	0	0
**ZNF143::CXCL8**	0	0	0	0	0	0	0	0	1	0	0	0	0	0	0	0	0	0	0	0	0	0	0	0	0	0	0	0
**ZNF143::NELL2**	0	0	0	0	0	0	0	0	0	0	0	0	0	0	0	0	0	0	0	0	0	0	0	0	0	1	0	0
**ZNF143::OGFOD1**	0	0	0	0	0	0	0	0	0	0	0	0	0	0	0	0	0	0	0	0	0	0	0	1	0	0	0	0
**ZNF219::ANXA11**	0	0	0	0	0	0	0	0	0	0	0	0	0	0	0	0	0	1	0	0	0	0	0	0	0	0	0	0
**ZNF219::C1QL1**	0	0	0	0	0	0	0	1	0	0	0	0	0	0	0	0	0	0	0	0	0	0	0	0	0	0	0	0
**ZNF219::C1QTNF5**	0	0	1	0	0	0	0	0	0	0	1	0	0	0	0	0	0	0	0	0	0	0	0	0	0	0	0	0
**ZNF219::CRELD2**	0	0	0	0	0	0	0	0	0	0	0	0	0	0	1	0	0	0	0	0	0	0	0	0	0	0	0	0
**ZNF219::EFEMP2**	0	0	0	0	0	0	0	0	0	0	1	0	0	0	0	0	0	0	0	0	0	0	0	0	0	0	0	0
**ZNF219::EGLN2**	0	0	0	0	0	0	0	0	0	1	0	0	0	0	1	0	0	0	0	0	0	0	0	0	0	0	0	0
**ZNF219::EMILIN1**	0	0	1	0	0	0	0	0	0	0	0	0	0	0	0	0	0	0	0	0	0	0	0	0	0	0	0	0
**ZNF219::FGF14**	0	0	0	0	0	0	0	0	0	1	0	0	0	0	0	0	0	0	0	0	0	0	0	0	0	0	0	0
**ZNF219::GPC2**	0	0	0	1	0	0	0	0	0	0	0	0	0	0	0	0	0	0	0	0	0	0	0	0	0	0	0	0
**ZNF219::LOXL1**	0	0	1	0	0	0	0	0	0	0	0	0	0	0	0	0	0	0	0	0	0	0	0	0	0	0	0	0
**ZNF219::LTBP3**	0	0	0	0	0	0	0	0	0	0	0	0	0	1	0	0	0	0	0	0	0	0	0	0	0	0	0	0

**ZNF219::MMP15**	0	0	0	0	0	0	0	0	0	1	0	0	0	0	0	0	0	0	0	0	0	0	0	0	0	0	0	0
**ZNF219::OGFOD2**	0	0	0	0	0	0	0	0	0	1	0	1	0	0	0	0	0	0	0	0	0	0	0	0	0	0	0	0
**ZNF219::PODNL1**	0	0	0	1	0	0	0	0	0	0	0	0	0	0	0	0	0	0	0	0	0	0	0	0	0	0	0	0
**ZNF219::SEMA6B**	0	0	0	0	0	0	0	0	0	0	1	0	0	0	0	0	0	0	0	0	0	0	0	0	0	0	0	0
**ZNF219::TNXB**	1	0	0	0	0	0	0	0	0	0	0	0	0	0	0	0	0	0	0	0	0	0	0	0	0	0	0	0
**ZNF281::COL24A1**	0	0	0	0	0	0	0	0	1	0	0	0	0	0	0	0	0	0	0	0	0	0	0	0	0	0	0	0
**ZNF281::DMP1**	0	0	0	0	0	0	0	0	0	0	0	0	0	0	0	0	0	0	0	0	0	0	0	1	0	0	0	0
**ZNF281::EFEMP2**	0	0	0	0	0	0	0	1	0	0	0	0	0	0	0	0	0	0	0	0	0	0	0	0	0	0	0	0
**ZNF281::LGALS8**	0	0	1	0	0	0	0	0	0	0	0	0	0	0	0	0	0	0	0	0	0	0	0	0	0	0	0	0
**ZNF281::SEMA3F**	0	0	1	0	0	0	0	0	0	0	0	0	0	0	0	0	0	0	0	0	0	0	0	0	0	0	0	0
**ZNF281::SERPINH1**	0	0	0	0	0	0	1	0	0	0	0	0	0	0	0	0	1	0	0	0	0	0	0	0	0	0	0	0
**ZNF281::TGFBI**	0	0	0	0	0	0	0	0	0	0	0	0	0	0	0	0	0	0	0	0	0	1	0	0	0	0	0	0
**ZNF35::ADAMTS6**	0	0	0	0	0	0	0	0	0	0	0	0	0	0	0	0	0	0	0	0	0	0	1	0	0	0	0	0
**ZNF35::ADAMTS9**	0	0	0	0	0	0	0	0	0	0	0	0	0	0	0	0	0	0	0	1	0	0	0	0	0	0	0	0
**ZNF35::COL15A1**	1	0	0	0	0	0	0	0	0	0	0	0	0	0	0	0	0	0	0	0	0	0	0	0	0	0	0	0
**ZNF35::TGFBI**	0	0	0	0	0	1	0	0	0	0	0	0	0	0	0	0	0	0	0	0	0	0	0	0	0	0	0	0
**ZNF35::TNXB**	1	0	0	0	0	0	0	0	0	0	0	0	0	0	0	0	0	0	0	0	0	0	0	0	0	0	0	0
**ZNF350::FMOD**	0	0	0	0	0	0	0	0	0	0	0	0	0	0	0	0	0	0	0	0	1	0	0	0	0	0	0	0
**ZNF410::ANXA4**	0	0	0	0	0	0	0	0	0	0	0	0	0	0	0	0	0	0	0	0	0	1	0	0	0	0	0	0
**ZNF410::COL13A1**	0	0	0	0	0	0	0	0	0	0	0	0	0	0	0	0	0	1	0	0	0	0	0	0	0	0	0	0
**ZNF410::FBLN5**	0	0	0	0	0	0	0	0	0	0	0	0	0	0	0	0	0	1	0	0	0	0	0	0	0	0	0	0
**ZNF423::ADAMTS7**	0	1	0	0	0	0	0	0	0	0	0	0	0	0	0	0	0	0	0	0	0	0	0	0	0	0	0	0
**ZNF423::COL15A1**	0	0	0	0	0	0	0	0	0	0	0	0	0	0	0	0	0	0	0	0	0	0	0	0	0	1	0	0
**ZNF423::PLXNA2**	0	0	0	0	0	0	0	0	0	0	0	0	0	0	0	0	0	0	0	0	0	0	0	0	0	1	0	0
**ZNF423::PLXNB1**	0	1	0	0	0	0	0	0	0	0	0	0	0	0	0	0	0	0	0	0	0	0	0	0	0	0	0	0
**ZNF423::SPON2**	0	0	0	0	0	0	0	0	1	0	0	0	0	0	0	0	0	0	0	0	0	0	0	0	0	0	0	0
**ZNF423::ZP3**	0	1	0	0	0	0	0	0	0	0	0	0	0	0	0	0	0	0	0	0	0	0	0	0	0	0	0	0
**ZNF628::ADAMTS7**	0	0	0	0	0	0	0	0	0	0	0	0	0	0	0	0	0	0	0	0	0	0	0	1	0	0	0	0
**ZNF628::AGRN**	0	0	0	0	0	0	0	0	0	0	1	0	0	0	0	0	0	0	0	0	0	0	0	0	0	0	0	0
**ZNF628::EGLN2**	0	0	1	0	0	0	0	0	0	0	0	0	0	0	1	0	0	1	0	0	0	0	0	0	1	0	0	1
**ZNF652::C1QTNF5**	0	0	0	0	0	0	0	0	0	0	0	0	0	1	0	0	0	0	0	0	0	0	0	0	0	0	0	0
**ZNF652::LAMA5**	0	0	0	0	0	0	0	0	0	0	0	0	0	0	0	0	1	0	0	0	0	0	0	0	0	0	0	0
**ZNF652::NPNT**	0	0	1	0	0	0	0	0	0	0	0	0	0	0	0	0	0	0	0	0	0	0	0	0	0	0	0	0
**ZNF652::WNT5A**	0	0	0	0	0	0	0	0	0	0	0	1	0	0	0	0	0	0	0	0	0	0	0	0	0	0	0	0
**ZNF691::BGLAP**	0	1	0	0	0	0	0	0	0	0	0	0	0	0	0	0	0	0	0	1	0	0	0	0	0	0	0	0
**ZNF691::ZP3**	0	1	0	0	0	0	0	0	0	0	0	0	0	0	0	0	0	0	0	0	0	0	0	0	0	0	0	0
**ZNF713::ADAMTS6**	0	0	0	0	0	1	0	0	0	0	0	0	0	1	0	0	0	0	0	0	0	0	0	1	0	0	0	0
**ZNF713::CRLF3**	0	0	0	0	0	0	0	0	0	0	0	0	1	0	0	0	0	0	0	0	0	0	0	0	0	0	0	0
**ZNF713::MASP1**	0	0	0	0	0	0	0	1	0	0	0	0	0	0	0	0	0	0	0	0	0	0	0	0	0	0	0	0
**ZNF75A::NELL2**	0	0	1	0	0	0	0	0	0	0	0	0	0	0	0	0	0	0	0	0	0	0	0	0	0	0	0	0
**ZNF75A::THBS3**	0	0	0	0	0	0	0	0	0	0	0	1	0	0	0	0	0	0	0	0	0	0	0	0	0	0	0	0
**ZNF784::LTBP3**	0	0	0	0	0	0	0	0	0	0	0	0	0	1	0	0	0	0	0	0	0	0	0	0	0	0	0	0
**ZNF784::SEMA6C**	0	0	0	0	0	0	0	0	0	0	1	0	0	0	0	0	0	0	0	0	0	0	0	0	0	0	0	0
**ZNF784::ST14**	0	0	0	0	0	0	0	0	0	1	0	0	0	0	0	0	0	0	0	0	0	0	0	0	0	0	0	0
**ZSCAN16::ANXA5**	0	0	0	0	0	0	0	0	0	0	0	0	0	0	0	0	0	1	0	0	0	0	0	0	0	0	0	0
**ZSCAN16::HYAL3**	0	0	0	0	0	0	0	0	0	0	0	0	0	0	0	0	0	0	0	0	0	0	0	0	0	0	1	0

**ZSCAN16::LAMC2**	0	1	0	0	0	0	0	0	0	0	0	0	0	0	0	0	0	0	0	0	0	0	0	0	0	0	0	0
**ZSCAN16::SEMA3F**	0	1	0	0	0	0	0	0	0	0	0	0	0	0	0	0	0	0	0	0	0	0	0	0	0	0	0	0
**ZSCAN16::ST14**	0	1	0	0	0	0	0	0	0	0	0	0	0	0	0	0	0	0	0	0	0	0	0	0	0	0	0	0
**ZSCAN16::VWCE**	0	0	0	0	0	0	0	0	0	0	0	0	0	1	0	0	0	0	0	0	0	0	0	0	0	0	0	0

Numbers in the table report whether a module is active (1) or not (0) in any given tumor type. Modules are named as TF::matrisome target.

Supplemental Table 7. Prognostic matrisome modules

Tumor	Transcription factor	Matrisome gene target	*P* value	Better prognosis	Worse prognosis
**ACC**	TCF3	SERPINH1	5.28988E-06	module-low	module-high
**ACC**	TCF7L2	ADAMTS7	3.07808E-05	module-low	module-high
**ACC**	SMAD3	NTNG2	0.000135261	module-low	module-high
**ACC**	ZBTB49	ADAMTS7	0.000294777	module-low	module-high
**ACC**	SMAD3	SEMA7A	0.000501965	module-low	module-high
**ACC**	TBX1	NTNG2	0.000531329	module-low	module-high
**ACC**	TCF3	COL15A1	0.00123809	module-low	module-high
**ACC**	RARB	MMRN2	0.00263679	module-high	module-low
**ACC**	SOX8	WNT11	0.014516513	module-low	module-high
**ACC**	ZNF35	TNXB	0.03487466	module-low	module-high
**ACC**	ZNF143	COL15A1	0.045534996	module-low	module-high
**BLCA**	NR2F6	OGFOD2	0.001649254	module-high	module-low
**BLCA**	NR2F6	PLXNB2	0.004526848	module-high	module-low
**BLCA**	NR2F6	ST14	0.002371232	module-high	module-low
**BLCA**	NR6A1	BGLAP	0.048204052	module-high	module-low
**BLCA**	PPARG	ADAMTS7	0.000545996	module-high	module-low
**BLCA**	PPARG	IGFBP3	0.006882439	module-high	module-low
**BLCA**	PPARG	TINAGL1	0.008805562	module-high	module-low
**BLCA**	PRRX1	FBN2	0.035846959	module-low	module-high
**BLCA**	PRRX1	MMP14	0.019172173	module-low	module-high
**BLCA**	RARG	PLXNB2	0.021044962	module-high	module-low
**BLCA**	RFX7	PLXNB1	0.0277367	module-high	module-low
**BLCA**	RUNX2	FBN2	0.025574572	module-low	module-high
**BLCA**	SMAD3	IGFBP3	0.041645963	module-high	module-low
**BLCA**	TBX1	SDC1	0.018132999	module-high	module-low
**BLCA**	TCF3	PLXNB1	0.010507094	module-high	module-low
**BLCA**	TEAD4	ADAM15	0.037885549	module-low	module-high
**BLCA**	TEAD4	PLAU	0.024111034	module-low	module-high
**BLCA**	TFAP4	GPC2	0.023439704	module-high	module-low
**BLCA**	ZNF691	BGLAP	0.016859878	module-high	module-low
**BLCA**	ZSCAN16	SEMA3F	2.08E-05	module-high	module-low
**BLCA**	ZSCAN16	ST14	0.000714791	module-high	module-low
**BRCA**	NR2F2	LRG1	0.001160383	module-high	module-low
**BRCA**	PRDM1	CLEC2D	0.01246197	module-high	module-low
**BRCA**	RARA	CHRD	0.019458371	module-high	module-low
**BRCA**	RARA	SEMA3F	0.001561244	module-high	module-low
**BRCA**	RFX1	CILP2	0.001738291	module-high	module-low
**BRCA**	RFX1	MMP17	0.046825623	module-high	module-low

**BRCA**	RREB1	LRG1	0.007878654	module-high	module-low
**BRCA**	RXRA	SEMA3F	0.049877246	module-high	module-low
**BRCA**	SP1	NELL2	0.017276383	module-high	module-low
**BRCA**	SREBF1	MUC1	0.00278415	module-high	module-low
**BRCA**	TEAD1	LOXL2	0.015085869	module-low	module-high
**BRCA**	TFAP2A	MUC1	0.005754719	module-high	module-low
**BRCA**	USF1	SEMA4A	0.014215827	module-high	module-low
**BRCA**	XBP1	ANXA9	0.003624732	module-high	module-low
**BRCA**	XBP1	CHRD	0.007690921	module-high	module-low
**BRCA**	XBP1	LRG1	0.001319192	module-high	module-low
**BRCA**	XBP1	MFAP2	0.017658529	module-high	module-low
**BRCA**	XBP1	SERPINH1	0.044552714	module-high	module-low
**BRCA**	ZBTB7A	MMP17	0.027785497	module-high	module-low
**BRCA**	ZBTB7B	LRG1	0.000183295	module-high	module-low
**BRCA**	ZBTB7B	MUC1	0.014483349	module-high	module-low
**BRCA**	ZEB1	ST14	0.01551516	module-low	module-high
**BRCA**	ZNF219	LOXL1	0.006749677	module-high	module-low
**BRCA**	ZNF281	SEMA3F	0.007312899	module-high	module-low
**BRCA**	ZNF628	EGLN2	0.01232958	module-high	module-low
**BRCA**	ZNF652	NPNT	0.001214069	module-high	module-low
**BRCA**	ZNF75A	NELL2	0.035412353	module-high	module-low
**CESC**	BACH1	CXCL8	0.003311276	module-low	module-high
**CESC**	NFE2	CXCL1	0.027222979	module-low	module-high
**CESC**	RFX2	PLXNB1	0.046491876	module-high	module-low
**CESC**	SOX2	HPSE	0.015019977	module-high	module-low
**CESC**	STAT5A	CCL22	0.048813656	module-high	module-low
**CESC**	TEAD1	LAMC2	0.014089816	module-low	module-high
**CESC**	ZNF219	PODNL1	0.011662186	module-low	module-high
**COAD**	RELA	C1QTNF1	0.003342958	module-low	module-high
**COAD**	RFX3	ADAMTS6	0.027789537	module-low	module-high
**COAD**	ZEB1	COL6A3	0.042602017	module-low	module-high
**COAD**	ZNF713	ADAMTS6	0.028415083	module-low	module-high
**GBM**	VDR	CSF1	0.012676998	module-low	module-high
**GBM**	VDR	F13A1	0.040482665	module-low	module-high
**GBM**	ZNF281	EFEMP2	0.024230538	module-low	module-high
**HNSC**	RARA	SERPINE1	0.035066904	module-low	module-high
**HNSC**	RUNX2	SEMA3C	0.029994943	module-low	module-high
**HNSC**	SOX18	PAPLN	0.02213199	module-high	module-low
**HNSC**	SP3	COL8A2	0.025686302	module-high	module-low
**HNSC**	STAT5A	CCL5	0.007139558	module-high	module-low
**HNSC**	STAT5A	COL8A2	0.001784191	module-high	module-low
**HNSC**	STAT5A	HAPLN3	0.018176652	module-high	module-low
**HNSC**	STAT5A	MMP28	0.038219845	module-high	module-low
**HNSC**	VDR	TINAGL1	0.013020321	module-low	module-high

**HNSC**	ZEB1	MMP19	0.028886024	module-high	module-low
**KICH**	TFEB	EGLN2	0.00789214	module-high	module-low
**KICH**	ZBTB7A	LAMA5	0.012130945	module-high	module-low
**KICH**	ZNF219	OGFOD2	0.028149292	module-high	module-low
**KICH**	ZNF219	EGLN2	0.006278103	module-high	module-low
**KIRC**	ATF3	CXCL2	0.005604564	module-low	module-high
**KIRC**	NFKB2	EBI3	0.002623082	module-low	module-high
**KIRC**	NFKB2	P4HA1	0.046885302	module-low	module-high
**KIRC**	PAX2	CX3CL1	0.002931256	module-high	module-low
**KIRC**	POU2F1	LGALS8	0.01888582	module-low	module-high
**KIRC**	POU3F1	CSF1	0.03447886	module-low	module-high
**KIRC**	RARG	LGI4	0.002039085	module-low	module-high
**KIRC**	RELA	SERPINE1	0.000318604	module-low	module-high
**KIRC**	RFX1	LAMA5	0.008127486	module-low	module-high
**KIRC**	RORA	HMCN1	8.86E-06	module-high	module-low
**KIRC**	RORA	P4HA2	0.045830965	module-high	module-low
**KIRC**	RUNX1	ANXA5	1.17E-06	module-low	module-high
**KIRC**	RUNX1	LAMA4	0.003931839	module-low	module-high
**KIRC**	RUNX1	VCAN	0.000227449	module-low	module-high
**KIRC**	SP1	CSF1	0.004572017	module-low	module-high
**KIRC**	SP1	MUC3A	0.009720805	module-low	module-high
**KIRC**	STAT5A	SERPING1	0.002767937	module-low	module-high
**KIRC**	TBX19	ADAMTS10	0.009062649	module-low	module-high
**KIRC**	TBX19	CBLN3	0.000376126	module-low	module-high
**KIRC**	TCF4	A2M	0.012738597	module-high	module-low
**KIRC**	TCF4	CLEC14A	0.022825311	module-high	module-low
**KIRC**	TCF4	HSPG2	0.003139282	module-high	module-low
**KIRC**	TEAD3	CSF1	1.06E-08	module-low	module-high
**KIRC**	TEAD4	SERPINE1	0.000111154	module-low	module-high
**KIRC**	TFAP4	ADAMTSL4	2.06E-05	module-low	module-high
**KIRC**	TFAP4	LOXL2	0.010515427	module-low	module-high
**KIRC**	TFAP4	PLOD1	0.001027241	module-low	module-high
**KIRC**	ZFX	PLXNA2	0.001397604	module-high	module-low
**KIRP**	PAX2	CX3CL1	0.006707384	module-high	module-low
**KIRP**	POU3F3	CX3CL1	0.030862625	module-high	module-low
**KIRP**	SP1	PAPLN	0.028037801	module-high	module-low
**KIRP**	STAT5A	HTRA4	0.009502427	module-high	module-low
**LAML**	RREB1	NTNG2	0.015116527	module-high	module-low
**LAML**	RUNX2	SERPINB8	0.022606921	module-high	module-low
**LAML**	RXRA	SEMA4A	0.045547267	module-low	module-high
**LAML**	SP3	ZFP91	0.034682139	module-high	module-low
**LAML**	TBX1	CLEC11A	0.00045927	module-high	module-low
**LAML**	TEAD4	VWCE	0.023116405	module-high	module-low
**LAML**	ZEB1	NTNG2	0.048046253	module-high	module-low

**LAML**	ZNF713	CRLF3	0.020548102	module-high	module-low
**LGG**	BACH1	A2M	0.015715357	module-low	module-high
**LGG**	NFKB1	LTBP3	0.029222746	module-low	module-high
**LGG**	NR3C1	FGF2	0.026140605	module-high	module-low
**LGG**	NR3C1	VCAN	0.000349736	module-high	module-low
**LGG**	PAX6	MASP1	0.03847823	module-high	module-low
**LGG**	REST	LOXL3	9.91E-09	module-low	module-high
**LGG**	REST	TNC	1.01E-07	module-low	module-high
**LGG**	REST	WNT5A	1.94E-05	module-low	module-high
**LGG**	RFX1	LTBP3	0.012040946	module-low	module-high
**LGG**	RFX5	ADAMTS6	0.011068769	module-high	module-low
**LGG**	RUNX1	A2M	0.000141338	module-low	module-high
**LGG**	RUNX1	CSF1	8.31E-05	module-low	module-high
**LGG**	RUNX1	NTN1	0.002190275	module-low	module-high
**LGG**	SOX2	SMOC1	1.93E-05	module-high	module-low
**LGG**	STAT5A	A2M	0.000381721	module-low	module-high
**LGG**	STAT5A	LOXL3	7.51E-05	module-low	module-high
**LGG**	TCF12	ADAMTS6	0.001553505	module-high	module-low
**LGG**	TCF12	BMP2	3.90E-08	module-high	module-low
**LGG**	TCF12	VCAN	7.78E-06	module-high	module-low
**LGG**	TFAP4	ANGPTL2	3.04E-09	module-high	module-low
**LGG**	TFAP4	PODN	0.00295398	module-high	module-low
**LGG**	VAX2	C1QL1	2.01E-05	module-high	module-low
**LGG**	VAX2	GPC2	0.012321136	module-high	module-low
**LGG**	ZNF652	C1QTNF5	0.000131345	module-high	module-low
**LGG**	ZNF713	ADAMTS6	0.044244092	module-high	module-low
**LGG**	ZNF784	LTBP3	0.032087461	module-low	module-high
**LUAD**	NFAT5	CLEC2D	0.019561203	module-high	module-low
**LUAD**	NR2F6	KAZALD1	0.018775607	module-low	module-high
**LUAD**	PITX1	ANXA10	0.015047999	module-low	module-high
**LUAD**	PLAG1	CRLF1	0.020508454	module-low	module-high
**LUAD**	PPARA	PAPLN	0.012858276	module-low	module-high
**LUAD**	PRRX2	PODNL1	0.024020211	module-high	module-low
**LUAD**	RARA	ANGPTL4	0.012594531	module-low	module-high
**LUAD**	RARA	SDC1	0.032133692	module-high	module-low
**LUAD**	RARA	VWA1	0.019394745	module-high	module-low
**LUAD**	RELA	HPSE	0.004126654	module-low	module-high
**LUAD**	SP3	HPSE	0.009479946	module-low	module-high
**LUAD**	STAT5A	HAPLN3	0.031420021	module-low	module-high
**LUAD**	TBX19	CBLN3	0.028934965	module-high	module-low
**LUAD**	TBX19	PLXNA3	0.014336801	module-high	module-low
**LUAD**	TFAP2A	ADAMTSL5	0.001157149	module-low	module-high
**LUAD**	TFAP2A	SEMA4B	4.56E-05	module-low	module-high
**LUAD**	VDR	COL24A1	0.015345863	module-high	module-low

**LUAD**	ZIC2	ADAMTSL5	0.002412552	module-low	module-high
**LUSC**	PGR	VCAN	0.022504219	module-low	module-high
**LUSC**	POU6F2	ADAMTS17	0.035313357	module-high	module-low
**LUSC**	POU6F2	NELL2	0.028833279	module-high	module-low
**LUSC**	RORA	CD109	0.037308447	module-high	module-low
**LUSC**	TCF3	GPC2	0.047962331	module-high	module-low
**LUSC**	TEAD3	ADAMTS17	0.000565815	module-high	module-low
**LUSC**	TFAP2A	GREM1	0.038672636	module-low	module-high
**LUSC**	TFAP2C	WNT5A	0.014194057	module-high	module-low
**LUSC**	VAX2	ADAMTS17	0.027206135	module-high	module-low
**LUSC**	ZIC3	LAMA1	0.018094127	module-high	module-low
**MESO**	NR2F6	SERPINH1	1.86E-05	module-low	module-high
**MESO**	SNAI2	COL15A1	7.26E-05	module-low	module-high
**MESO**	NR2F1	CXCL6	0.00011213	module-high	module-low
**MESO**	ZNF219	ANXA11	0.001054055	module-high	module-low
**MESO**	RARG	LOXL1	0.001107784	module-low	module-high
**MESO**	VAX2	MMP14	0.001766403	module-low	module-high
**MESO**	VAX2	EFEMP2	0.007231699	module-low	module-high
**MESO**	WT1	FMOD	0.010194241	module-high	module-low
**MESO**	WT1	SULF1	0.012273306	module-high	module-low
**MESO**	XBP1	CCL2	0.014433129	module-high	module-low
**MESO**	PDX1	COL11A2	0.015279822	module-low	module-high
**MESO**	SP4	NID2	0.015342693	module-low	module-high
**MESO**	PBX1	LAMA4	0.018139411	module-high	module-low
**MESO**	NFIB	EGFLAM	0.020564475	module-low	module-high
**MESO**	OSR1	SEMA5A	0.035720984	module-low	module-high
**PAAD**	NFAT5	LAMA3	0.001192108	module-low	module-high
**PAAD**	NKX2-8	EREG	0.02226153	module-low	module-high
**PAAD**	SMAD3	PRSS12	0.007529121	module-low	module-high
**PAAD**	TEAD3	MMP3	0.002817375	module-low	module-high
**PRAD**	NR2F6	BGLAP	0.037671358	module-low	module-high
**PRAD**	REST	IMPG1	0.021579481	module-low	module-high
**SARC**	NFKB2	HAPLN3	0.020259306	module-high	module-low
**SARC**	NKX6-1	LOXL2	0.00217866	module-low	module-high
**SARC**	NR2F6	SERPINH1	0.015464811	module-low	module-high
**SARC**	POU3F3	PXDN	0.025713805	module-low	module-high
**SARC**	REST	FBN2	0.002778349	module-low	module-high
**SARC**	RORA	PXDN	0.00232826	module-low	module-high
**SARC**	SOX17	LAMA4	0.036000281	module-high	module-low
**SARC**	TCF3	MFAP2	0.007394702	module-low	module-high
**STAD**	POU2F2	NTNG2	0.028992554	module-low	module-high
**STAD**	RARA	HTRA3	0.036741491	module-low	module-high
**STAD**	RORA	SRPX2	0.00152623	module-low	module-high
**STAD**	RREB1	PLXNB2	0.037169493	module-high	module-low

**STAD**	SNAI2	LOX	0.002392283	module-low	module-high
**STAD**	TAL1	ADAMTS7	0.010536732	module-low	module-high
**STAD**	TAL1	ANGPT2	0.001790626	module-low	module-high
**STAD**	TCF21	AGT	0.03075979	module-low	module-high
**STAD**	TCF3	SEMA4D	0.01999062	module-high	module-low
**STAD**	TCF4	COL15A1	0.024149113	module-low	module-high
**STAD**	TEAD1	HSPG2	0.030284765	module-low	module-high
**STAD**	TEAD4	SERPINE1	0.01340268	module-low	module-high
**STAD**	ZBTB7B	SDC1	0.036447193	module-high	module-low
**STAD**	ZEB1	IGFBP7	0.000154535	module-low	module-high
**STAD**	ZEB1	THBS1	0.004867948	module-low	module-high
**STAD**	ZNF143	OGFOD1	0.009753086	module-low	module-high
**STAD**	ZNF713	ADAMTS6	0.003522014	module-low	module-high
**THCA**	POU2F3	VWA5A	0.013279907	module-high	module-low
**THCA**	PRDM1	ADAMTS9	0.006701597	module-high	module-low
**THCA**	PRDM1	FGF1	0.034147628	module-high	module-low
**THCA**	RARG	CST6	0.042919101	module-high	module-low
**THCA**	REST	PLXNC1	0.034150661	module-high	module-low
**THCA**	TCF4	COL15A1	0.013687991	module-low	module-high
**THCA**	TFAP2C	ANXA1	0.041003381	module-high	module-low
**THCA**	TFAP2C	COL8A2	0.036689361	module-high	module-low
**THCA**	ZNF143	NELL2	0.013923797	module-high	module-low
**UCEC**	NR2F6	CILP2	0.000884206	module-low	module-high
**UCEC**	ZBTB7A	PRSS1	0.002737465	module-low	module-high
**UCEC**	ZBTB7B	ESM1	0.003827208	module-low	module-high
**UCS**	RARA	CCL7	0.010641011	module-high	module-low
**UCS**	TCF7L1	ADAMTS17	0.022391296	module-high	module-low

P values are calculated for univariate survival curves (Kaplan-Meier, Log-rank test). Patients in each cohort are divided at the average expression of the module (average of TF and target expression values) into module-high and -low group (above and below the average module activity, respectively) and compared. Association of a group with better (longer overall survival) or worse (shorter overall survival) prognosis is reported.

Supplemental Table 8. Drivers of matrisome modules

Driver	ACC	BLCA	BRCA	CESC	CHOL	COAD	ESCA	GBM	HNSC	KICH	KIRC	KIRP	LAML	LGG	LIHC	LUAD	LUSC	MESO	PAAD	PCPG	PRAD	READ	SARC	STAD	TGCT	THCA	UCEC	UCS	In tumors	N tot int	N avg int
EP300	9	25	33	34	4	6	10	15	54	6	30	18	16	14	2	24	20	9	3	8	15	6	8	23	10	23	16	6	28	447	15.964
TP53	10	19	32	22	3	14	18	18	28	16	20	10	14	12	0	24	34	12	2	12	18	6	8	22	3	21	16	4	27	418	14.929
CREBBP	7	18	17	10	3	6	8	15	19	5	21	12	16	9	0	13	8	7	0	9	8	3	6	13	8	17	6	3	26	267	9.536
NCOR1	4	21	12	11	5	3	4	12	11	4	13	9	6	13	0	8	11	5	0	8	7	5	3	7	4	16	6	4	26	212	7.571
RB1	3	17	14	10	2	0	4	16	10	3	9	11	9	0	0	6	28	0	0	3	7	2	4	7	0	10	12	0	21	187	6.679
SMARCA4	3	8	9	7	0	4	9	7	12	4	13	11	7	11	2	10	7	4	0	6	4	5	5	6	5	7	2	0	25	168	6.000
ARID1A	8	10	5	12	2	8	10	6	7	4	11	2	0	6	0	5	14	0	2	7	5	6	0	10	0	7	4	10	23	161	5.750
CTNNB1	6	12	10	9	0	0	6	3	8	3	8	7	3	5	0	6	8	4	0	2	0	3	0	9	3	10	8	3	22	136	4.857
SMAD4	3	11	7	5	2	4	6	0	10	3	6	8	0	5	0	6	0	4	2	0	0	4	0	12	0	14	3	0	19	115	4.107
NSD1	2	12	8	0	2	0	0	6	10	2	8	0	4	0	0	9	0	5	0	3	10	0	3	5	4	7	2	3	19	105	3.750
NOTCH1	3	6	6	6	2	0	6	3	10	3	6	0	0	20	0	6	4	4	0	0	0	2	0	5	0	10	0	0	17	102	3.643
FBXW7	0	4	0	0	2	0	6	3	20	0	4	3	5	4	0	0	14	0	0	2	0	0	2	0	0	3	6	0	14	78	2.786
BRCA2	2	5	0	6	0	3	4	0	8	0	5	12	0	3	0	4	0	0	0	0	3	4	0	5	0	7	0	0	14	71	2.536
BCOR	3	4	5	0	0	0	0	3	0	3	5	9	4	0	0	0	5	0	0	2	4	0	0	0	0	4	6	0	13	57	2.036
GATA3	2	0	4	3	0	0	5	0	5	0	7	7	0	0	0	0	0	0	0	2	0	2	0	0	0	3	0	0	10	40	1.429
CHD4	0	0	5	3	0	0	4	4	0	5	8	0	0	3	2	0	0	0	0	0	0	0	0	0	0	0	0	2	9	36	1.286
EGFR	0	4	0	0	0	0	0	4	4	2	0	4	0	8	0	6	0	0	0	2	0	2	0	0	0	0	0	0	9	36	1.286
CDKN2A	0	0	0	0	2	0	0	3	8	0	6	7	0	0	0	0	0	0	0	0	0	0	0	0	0	4	0	0	6	30	1.071
PIK3CA	2	3	0	4	0	0	4	0	0	0	0	0	0	0	0	0	0	0	0	0	0	2	0	6	0	3	0	0	7	24	0.857
ATM	0	0	5	0	2	0	0	0	0	0	4	0	0	0	0	6	0	0	0	3	3	0	0	0	0	0	0	0	6	23	0.821
NFE2L2	0	0	0	3	0	0	0	0	6	0	0	2	0	0	0	0	0	0	0	0	0	0	0	0	0	3	8	0	5	22	0.786
RUNX1	0	0	13	0	0	0	0	0	0	0	0	0	8	0	0	0	0	0	0	0	0	0	0	0	0	0	0	0	2	21	0.750
VHL	0	0	0	0	0	0	0	0	0	0	8	7	0	0	0	0	0	3	0	0	0	0	0	0	0	0	0	0	3	18	0.643
RXRA	0	17	0	0	0	0	0	0	0	0	0	0	0	0	0	0	0	0	0	0	0	0	0	0	0	0	0	0	1	17	0.607
CHD8	3	3	0	4	0	0	0	0	0	0	0	0	0	0	2	0	0	0	0	0	0	0	0	0	0	4	0	0	5	16	0.571
SETD2	2	3	0	3	0	0	0	0	0	0	0	0	0	0	0	0	0	0	0	0	0	0	0	0	0	3	0	0	4	11	0.393
USP9X	2	3	0	3	0	0	0	0	0	0	0	0	0	0	0	0	0	0	0	0	0	0	0	0	0	3	0	0	4	11	0.393
APC	0	0	5	0	0	0	0	0	0	0	0	0	0	0	0	0	6	0	0	0	0	0	0	0	0	0	0	0	2	11	0.393
KLF5	0	11	0	0	0	0	0	0	0	0	0	0	0	0	0	0	0	0	0	0	0	0	0	0	0	0	0	0	1	11	0.393
KMT2A	0	0	0	0	0	0	0	3	0	0	4	0	0	3	0	0	0	0	0	0	0	0	0	0	0	0	0	0	3	10	0.357
ARID2	2	4	0	0	0	0	0	0	0	2	0	0	0	0	0	0	0	0	0	0	0	0	0	0	0	0	0	0	3	8	0.286
KMT2B	2	3	0	0	0	0	0	0	0	0	0	0	0	0	0	0	0	0	0	0	0	0	0	0	0	3	0	0	3	8	0.286
TLR4	2	3	0	0	0	0	0	0	0	0	0	0	0	0	0	0	0	0	0	0	0	0	0	0	0	3	0	0	3	8	0.286
KEAP1	0	0	0	0	0	0	0	0	0	0	0	0	0	0	0	4	4	0	0	0	0	0	0	0	0	0	0	0	2	8	0.286
CDKN1A	0	7	0	0	0	0	0	0	0	0	0	0	0	0	0	0	0	0	0	0	0	0	0	0	0	0	0	0	1	7	0.250
MEN1	7	0	0	0	0	0	0	0	0	0	0	0	0	0	0	0	0	0	0	0	0	0	0	0	0	0	0	0	1	7	0.250
AJUBA	0	0	0	0	0	0	0	0	4	0	0	0	0	0	0	0	0	0	0	0	0	0	0	0	0	0	0	0	1	4	0.143
SIN3A	0	0	0	0	0	0	0	0	0	0	0	0	0	0	0	0	0	0	0	0	0	0	0	0	0	0	4	0	1	4	0.143
CTCF	0	0	0	0	0	0	0	0	0	0	0	0	0	0	0	0	0	0	0	0	0	0	0	0	0	0	3	0	1	3	0.107
KANSL1	0	3	0	0	0	0	0	0	0	0	0	0	0	0	0	0	0	0	0	0	0	0	0	0	0	0	0	0	1	3	0.107
N drivers/tumor	22	26	17	18	12	8	15	16	18	15	20	17	11	14	4	15	13	10	4	14	11	14	8	13	7	23	15	8			
N tum int	87	236	190	155	31	48	104	121	234	65	196	139	92	116	8	137	163	57	9	69	84	52	39	130	37	185	102	35			

Drivers identified in the study and number of TF they interact with in any given cancer. The table also reports the following Pan-Cancer and tumor specific analyses (bold red): number of tumors in which the driver is active ("In tumors"); total number of TFs regulated by the driver Pan-Cancer ("N tot int"); average number of TFs regulated by the driver Pan-Cancer ("N avg int"); number of active drivers in any tumor ("N drivers/tumor"); number of TFs regulated by the driver in any given cancer ("N tum int").

Supplemental Table 9. KEGG Pathways enrichment of matrisome regulators (drivers + TFs)

Pathway	ACC	BLCA	BRCA	CESC	CHOL	COAD	ESCA	GBM	HNSC	KICH	KIRC	KIRP	LAML	LGG	LUAD	LUSC	MESO	PAAD	PCPG	PRAD	READ	SARC	STAD	TGCT	THCA	UCEC	UCS	In tumors
Adherens junction	12.28	10.90	10.33	11.51	9.85	11.80	14.40	14.29	17.19	12.86	17.06	12.56	11.10	15.41	14.94	13.65	12.19	11.70	11.58	11.07	12.75	6.02	13.34	11.27	7.92	15.61	12.83	**27**
Cell cycle	15.93	17.26	13.36	15.06	21.46	15.38	14.40	17.18	13.68	16.63	16.61	13.03	11.42	15.51	14.94	11.16	15.98	9.76	18.02	17.52	13.24	10.89	13.28	9.00	13.89	12.76	4.86	**27**
FoxO signaling pathway	9.88	14.24	7.95	11.94	14.31	12.28	11.52	11.28	10.97	13.42	10.80	7.31	11.42	9.56	14.94	8.50	6.92	9.76	14.99	11.32	10.37	4.57	7.83	4.00	8.46	4.34	4.86	**27**
MicroRNAs in cancer	15.08	22.06	14.92	22.96	23.67	20.82	19.10	18.92	21.33	18.89	21.15	12.49	19.06	17.74	23.42	12.81	15.17	12.51	30.31	18.96	15.46	12.90	17.38	8.49	15.65	9.12	6.92	**27**
Wnt signaling pathway	15.39	10.64	10.09	11.45	10.56	11.77	10.94	10.77	12.99	12.88	15.83	9.69	10.95	14.95	11.32	7.98	9.23	16.14	11.47	10.94	12.75	7.06	12.61	8.63	10.19	12.34	12.96	**27**
Notch signaling pathway	6.90	6.04	5.69	6.51	7.40	6.54	9.48	6.04	5.92	10.78	5.75	7.03	6.10	6.71	6.63	9.06	6.75	8.77	9.76	0.00	7.01	10.97	5.79	6.34	6.20	6.81	3.94	**26**
TGF-beta signaling pathway	5.74	7.48	7.10	8.08	6.19	5.39	10.75	7.56	7.28	5.90	7.21	8.86	4.90	5.60	8.14	4.49	8.42	7.73	4.99	4.70	12.32	3.11	7.04	0.00	7.58	5.60	3.26	**26**
Transcriptional misregulation in cancer	14.22	23.45	22.15	10.42	12.75	10.73	12.41	27.33	20.15	11.88	26.23	11.77	27.84	13.69	18.83	17.55	20.32	0.00	13.12	18.02	11.83	12.30	16.57	16.15	20.51	8.60	6.74	**26**
HIF-1 signaling pathway	10.52	18.27	8.56	16.00	15.03	12.97	12.23	12.12	14.64	14.10	11.62	7.86	9.30	10.10	15.83	3.88	10.41	10.24	9.76	9.40	14.01	0.00	11.34	7.01	14.83	0.00	5.24	**25**
Proteoglycans in cancer	7.37	10.55	3.70	6.68	3.54	9.23	6.06	6.04	5.77	10.46	5.55	5.26	6.12	4.97	6.70	3.88	0.00	7.91	6.48	6.10	10.37	0.00	5.59	0.00	4.16	0.00	5.75	**23**
p53 signaling pathway	6.09	8.02	4.87	5.62	13.68	5.77	5.23	11.20	5.07	6.21	10.80	3.09	0.00	5.93	5.77	0.00	5.97	0.00	11.76	11.21	3.31	3.32	4.95	0.00	5.29	0.00	0.00	**21**
Apoptosis	8.34	7.18	4.29	7.84	5.97	7.93	4.67	4.53	6.97	8.77	4.28	0.00	4.70	8.09	10.70	0.00	8.23	4.14	0.00	0.00	0.00	3.00	6.78	9.72	9.72	0.00	0.00	**20**
Hippo signaling pathway	6.46	3.24	4.96	3.71	0.00	3.84	5.38	7.71	7.37	0.00	7.29	0.00	3.26	6.26	3.71	0.00	6.29	3.26	3.38	0.00	4.10	0.00	7.10	0.00	0.00	3.82	4.36	**19**
PI3K-Akt signaling pathway	3.75	7.96	0.00	4.97	6.59	5.20	4.52	4.36	4.17	5.97	3.90	0.00	4.56	3.51	8.88	0.00	0.00	4.01	4.72	0.00	3.84	0.00	0.00	0.00	4.37	0.00	0.00	**17**
Jak-STAT signaling pathway	3.93	3.13	0.00	5.68	0.00	5.85	5.23	0.00	4.99	4.13	0.00	3.85	5.28	3.72	5.79	0.00	0.00	5.84	0.00	0.00	6.43	0.00	0.00	3.39	3.20	0.00	4.29	**16**
ErbB signaling pathway	0.00	4.59	0.00	5.05	3.13	5.20	4.68	0.00	4.53	3.13	4.28	0.00	4.71	0.00	5.20	0.00	0.00	0.00	4.83	0.00	5.54	0.00	0.00	0.00	0.00	0.00	3.14	**13**
Neurotrophin signaling pathway	4.71	3.85	0.00	4.37	0.00	4.46	0.00	0.00	3.78	4.92	0.00	0.00	0.00	4.50	6.73	0.00	4.61	3.68	0.00	0.00	0.00	0.00	3.53	0.00	6.22	0.00	0.00	**12**
MAPK signaling pathway	0.00	0.00	0.00	0.00	3.20	4.40	3.73	3.59	3.53	3.09	4.98	0.00	0.00	6.52	6.12	0.00	0.00	0.00	0.00	0.00	0.00	0.00	0.00	0.00	3.70	0.00	0.00	**10**
Prolactin signaling pathway	3.21	0.00	0.00	5.51	0.00	5.67	0.00	0.00	4.99	3.42	0.00	0.00	0.00	5.85	8.55	0.00	3.24	0.00	0.00	0.00	3.25	0.00	0.00	0.00	5.25	0.00	0.00	**10**
Toll-like receptor signaling pathway	5.02	4.15	0.00	4.64	0.00	4.74	0.00	0.00	4.09	5.23	0.00	0.00	0.00	0.00	7.16	0.00	0.00	3.84	0.00	0.00	0.00	0.00	3.83	0.00	6.64	0.00	0.00	**10**
Focal adhesion	0.00	0.00	0.00	3.01	0.00	3.10	0.00	0.00	0.00	3.50	0.00	0.00	0.00	0.00	3.00	4.64	3.36	0.00	4.64	0.00	3.36	0.00	0.00	0.00	0.00	0.00	0.00	**8**
Estrogen signaling pathway	0.00	4.33	0.00	4.81	0.00	0.00	4.47	0.00	4.26	0.00	0.00	0.00	0.00	0.00	0.00	0.00	0.00	0.00	0.00	0.00	5.26	0.00	0.00	0.00	0.00	0.00	0.00	**5**
NOD-like receptor signaling pathway	0.00	0.00	0.00	0.00	0.00	0.00	0.00	0.00	0.00	0.00	0.00	0.00	0.00	3.38	3.35	0.00	3.45	0.00	0.00	0.00	0.00	0.00	0.00	0.00	3.07	0.00	0.00	**4**
Rap1 signaling pathway	0.00	0.00	0.00	3.00	0.00	3.10	0.00	0.00	0.00	3.50	0.00	0.00	0.00	0.00	0.00	0.00	0.00	0.00	0.00	0.00	3.36	0.00	0.00	0.00	0.00	0.00	0.00	**4**
TNF signaling pathway	0.00	0.00	0.00	4.56	0.00	0.00	0.00	0.00	0.00	0.00	0.00	0.00	0.00	0.00	4.60	0.00	4.78	0.00	0.00	0.00	0.00	0.00	0.00	0.00	4.15	0.00	0.00	**4**
Ras signaling pathway	0.00	0.00	0.00	0.00	0.00	0.00	0.00	0.00	0.00	3.40	0.00	0.00	0.00	3.02	4.68	0.00	0.00	0.00	0.00	0.00	0.00	0.00	0.00	0.00	0.00	0.00	0.00	**3**
mTOR signaling pathway	0.00	0.00	0.00	0.00	3.65	0.00	0.00	0.00	0.00	0.00	0.00	0.00	0.00	0.00	3.28	0.00	0.00	0.00	0.00	0.00	0.00	0.00	0.00	0.00	0.00	0.00	0.00	**2**
T cell receptor signaling pathway	0.00	0.00	0.00	0.00	0.00	0.00	0.00	0.00	0.00	0.00	0.00	0.00	0.00	0.00	4.77	0.00	0.00	0.00	0.00	0.00	0.00	0.00	0.00	0.00	4.34	0.00	0.00	**2**
AMPK signaling pathway	0.00	3.77	0.00	0.00	0.00	0.00	0.00	0.00	0.00	0.00	0.00	0.00	0.00	0.00	0.00	0.00	0.00	0.00	0.00	0.00	0.00	0.00	0.00	0.00	0.00	0.00	0.00	**1**
Oxytocin signaling pathway	0.00	3.15	0.00	0.00	0.00	0.00	0.00	0.00	0.00	0.00	0.00	0.00	0.00	0.00	0.00	0.00	0.00	0.00	0.00	0.00	0.00	0.00	0.00	0.00	0.00	0.00	0.00	**1**
Tight junction	0.00	0.00	0.00	0.00	0.00	0.00	0.00	0.00	0.00	0.00	0.00	0.00	0.00	0.00	0.00	0.00	0.00	0.00	3.72	0.00	0.00	0.00	0.00	0.00	0.00	0.00	0.00	**1**

KEGG pathways enrichment in drivers + TF for any given cancer type. Data are reported as (-Log$_{10}$(FDR)). The table also reports the following Pan-Cancer analysis (bold red): number of tumors in which the pathway is enriched ("In tumors")

Supplemental Table 10. Druggability of the cancer matrisome

drug name	tumor type	mechanism	use	molecular target
THIOTEPA	ACC	ctx	off label	specific master
CAPECITABINE	ACC	ctx	off label	specific master
CYCLOPHOSPHAMIDE	ACC	ctx	off label	specific master
DOCETAXEL	ACC	ctx	off label	specific master
EPIRUBICIN	ACC	ctx	off label	specific master
EVEROLIMUS	ACC	tgt	off label	specific master
GEMCITABINE	ACC	ctx	off label	specific master
LETROZOLE	ACC	tgt	off label	specific master
PACLITAXEL	ACC	ctx	off label	specific master
BEVACIZUMAB	ACC	tgt	off label	specific master
CETUXIMAB	ACC	tgt	off label	specific master
IRINOTECAN	ACC	ctx	off label	specific master
OXALIPLATIN	ACC	ctx	off label	specific master
PANITUMUMAB	ACC	tgt	off label	specific master
TEMOZOLOMIDE	ACC	ctx	off label	specific master
TEMSIROLIMUS	ACC	tgt	off label	specific master
CRIZOTINIB	ACC	tgt	off label	specific master
ETOPOSIDE	ACC	ctx	off label	specific master
CARBOPLATIN	ACC	ctx	off label	specific master
OLAPARIB	ACC	tgt	off label	specific master
MITOMYCIN	ACC	ctx	off label	specific master
DABRAFENIB	ACC	tgt	off label	specific master
PEMBROLIZUMAB	ACC	tgt	off label	specific master
TRAMETINIB	ACC	tgt	off label	specific master
VEMURAFENIB	ACC	tgt	off label	specific master
IFOSFAMIDE	ACC	ctx	off label	specific master
LEUPROLIDE ACETATE	ACC	tgt	off label	transcription factor
THIOTEPA	BLCA	ctx	on label	specific master
CAPECITABINE	BLCA	ctx	off label	specific master
CYCLOPHOSPHAMIDE	BLCA	ctx	off label	specific master
DOCETAXEL	BLCA	ctx	off label	specific master
EPIRUBICIN	BLCA	ctx	off label	specific master
EVEROLIMUS	BLCA	tgt	off label	specific master
FLUOROURACIL	BLCA	ctx	off label	specific master
FULVESTRANT	BLCA	tgt	off label	specific master
GEMCITABINE	BLCA	ctx	off label	specific master
LETROZOLE	BLCA	tgt	off label	specific master

METHOTREXATE	BLCA	ctx	off label	specific master
PACLITAXEL	BLCA	ctx	off label	specific master
PERTUZUMAB	BLCA	tgt	off label	specific master
THIOTEPA	BLCA	ctx	off label	specific master
TRASTUZUMAB	BLCA	tgt	off label	specific master
BEVACIZUMAB	BLCA	tgt	off label	specific master
CETUXIMAB	BLCA	tgt	off label	specific master
IRINOTECAN	BLCA	ctx	off label	specific master
OXALIPLATIN	BLCA	ctx	off label	specific master
PANITUMUMAB	BLCA	tgt	off label	specific master
REGORAFENIB	BLCA	tgt	off label	specific master
TEMOZOLOMIDE	BLCA	ctx	off label	specific master
TEMSIROLIMUS	BLCA	tgt	off label	specific master
CRIZOTINIB	BLCA	tgt	off label	specific master
ETOPOSIDE	BLCA	ctx	off label	specific master
CARBOPLATIN	BLCA	ctx	off label	specific master
MELPHALAN	BLCA	ctx	off label	specific master
OLAPARIB	BLCA	tgt	off label	specific master
MITOMYCIN	BLCA	ctx	off label	specific master
FLUTAMIDE	BLCA	tgt	off label	specific master
DABRAFENIB	BLCA	tgt	off label	specific master
PEMBROLIZUMAB	BLCA	tgt	off label	specific master
TRAMETINIB	BLCA	tgt	off label	specific master
VEMURAFENIB	BLCA	tgt	off label	specific master
FLOXURIDINE	BLCA	ctx	off label	specific master
IFOSFAMIDE	BLCA	ctx	off label	specific master
CABOZANTINIB	BLCA	tgt	off label	specific master
VANDETANIB	BLCA	tgt	off label	specific master
BICALUTAMIDE	BLCA	tgt	off label	transcription factor
LEUPROLIDE ACETATE	BLCA	tgt	off label	transcription factor
OXALIPLATIN	BLCA	ctx	off label	matrisome
THIOTEPA	BRCA	ctx	off label	specific master
CAPECITABINE	BRCA	ctx	on label	specific master
CYCLOPHOSPHAMIDE	BRCA	ctx	on label	specific master
DOCETAXEL	BRCA	ctx	on label	specific master
EPIRUBICIN	BRCA	ctx	on label	specific master
EVEROLIMUS	BRCA	tgt	on label	specific master
FLUOROURACIL	BRCA	ctx	on label	specific master
FULVESTRANT	BRCA	tgt	on label	specific master
GEMCITABINE	BRCA	ctx	on label	specific master
LETROZOLE	BRCA	tgt	on label	specific master

PACLITAXEL	BRCA	ctx	on label	specific master
PERTUZUMAB	BRCA	tgt	on label	specific master
THIOTEPA	BRCA	ctx	on label	specific master
TRASTUZUMAB	BRCA	tgt	on label	specific master
BEVACIZUMAB	BRCA	tgt	off label	specific master
CAPECITABINE	BRCA	ctx	off label	specific master
CETUXIMAB	BRCA	tgt	off label	specific master
FLUOROURACIL	BRCA	ctx	off label	specific master
IRINOTECAN	BRCA	ctx	off label	specific master
OXALIPLATIN	BRCA	ctx	off label	specific master
PANITUMUMAB	BRCA	tgt	off label	specific master
REGORAFENIB	BRCA	tgt	off label	specific master
CYCLOPHOSPHAMIDE	BRCA	ctx	off label	specific master
DOCETAXEL	BRCA	ctx	off label	specific master
EVEROLIMUS	BRCA	tgt	off label	specific master
TEMOZOLOMIDE	BRCA	ctx	off label	specific master
TEMSIROLIMUS	BRCA	tgt	off label	specific master
CRIZOTINIB	BRCA	tgt	off label	specific master
ETOPOSIDE	BRCA	ctx	off label	specific master
GEMCITABINE	BRCA	ctx	off label	specific master
PACLITAXEL	BRCA	ctx	off label	specific master
CARBOPLATIN	BRCA	ctx	off label	specific master
OLAPARIB	BRCA	tgt	off label	specific master
MITOMYCIN	BRCA	ctx	off label	specific master
DABRAFENIB	BRCA	tgt	off label	specific master
PEMBROLIZUMAB	BRCA	tgt	off label	specific master
TRAMETINIB	BRCA	tgt	off label	specific master
VEMURAFENIB	BRCA	tgt	off label	specific master
FLOXURIDINE	BRCA	ctx	off label	specific master
TRASTUZUMAB	BRCA	tgt	off label	specific master
IFOSFAMIDE	BRCA	ctx	off label	specific master
CABOZANTINIB	BRCA	tgt	off label	specific master
CETUXIMAB	BRCA	tgt	off label	transcription factor
BICALUTAMIDE	BRCA	tgt	off label	transcription factor
LEUPROLIDE ACETATE	BRCA	tgt	off label	transcription factor
THIOTEPA	CESC	ctx	off label	specific master
CAPECITABINE	CESC	ctx	off label	specific master
CYCLOPHOSPHAMIDE	CESC	ctx	off label	specific master
DOCETAXEL	CESC	ctx	off label	specific master
EPIRUBICIN	CESC	ctx	off label	specific master
EVEROLIMUS	CESC	tgt	off label	specific master

FLUOROURACIL	CESC	ctx	off label	specific master
FULVESTRANT	CESC	tgt	off label	specific master
GEMCITABINE	CESC	ctx	off label	specific master
LETROZOLE	CESC	tgt	off label	specific master
METHOTREXATE	CESC	ctx	off label	specific master
PACLITAXEL	CESC	ctx	off label	specific master
PERTUZUMAB	CESC	tgt	off label	specific master
TRASTUZUMAB	CESC	tgt	off label	specific master
BEVACIZUMAB	CESC	tgt	off label	specific master
CETUXIMAB	CESC	tgt	off label	specific master
IRINOTECAN	CESC	ctx	off label	specific master
OXALIPLATIN	CESC	ctx	off label	specific master
PANITUMUMAB	CESC	tgt	off label	specific master
REGORAFENIB	CESC	tgt	off label	specific master
TEMOZOLOMIDE	CESC	ctx	off label	specific master
TEMSIROLIMUS	CESC	tgt	off label	specific master
CRIZOTINIB	CESC	tgt	off label	specific master
ETOPOSIDE	CESC	ctx	off label	specific master
CARBOPLATIN	CESC	ctx	off label	specific master
OLAPARIB	CESC	tgt	off label	specific master
MITOMYCIN	CESC	ctx	off label	specific master
DABRAFENIB	CESC	tgt	off label	specific master
PEMBROLIZUMAB	CESC	tgt	off label	specific master
TRAMETINIB	CESC	tgt	off label	specific master
VEMURAFENIB	CESC	tgt	off label	specific master
FLOXURIDINE	CESC	ctx	off label	specific master
IFOSFAMIDE	CESC	ctx	off label	specific master
CABOZANTINIB	CESC	tgt	off label	specific master
BICALUTAMIDE	CESC	tgt	off label	transcription factor
LEUPROLIDE ACETATE	CESC	tgt	off label	transcription factor
PACLITAXEL	CESC	ctx	off label	matrisome
TRASTUZUMAB	CESC	tgt	off label	matrisome
CETUXIMAB	CESC	tgt	off label	matrisome
CARMUSTINE	CESC	ctx	off label	matrisome
GEFITINIB	CESC	tgt	off label	matrisome
DACARBAZINE	CESC	ctx	off label	matrisome
TRASTUZUMAB	CHOL	tgt	off label	specific master
THIOTEPA	CHOL	ctx	off label	transcription factor
CAPECITABINE	CHOL	ctx	off label	transcription factor
CYCLOPHOSPHAMIDE	CHOL	ctx	off label	transcription factor
DOCETAXEL	CHOL	ctx	off label	transcription factor

EPIRUBICIN	CHOL	ctx	off label	transcription factor
GEMCITABINE	CHOL	ctx	off label	transcription factor
PACLITAXEL	CHOL	ctx	off label	transcription factor
BEVACIZUMAB	CHOL	tgt	off label	transcription factor
CETUXIMAB	CHOL	tgt	off label	transcription factor
IRINOTECAN	CHOL	ctx	off label	transcription factor
OXALIPLATIN	CHOL	ctx	off label	transcription factor
PANITUMUMAB	CHOL	tgt	off label	transcription factor
TEMOZOLOMIDE	CHOL	ctx	off label	transcription factor
CRIZOTINIB	CHOL	tgt	off label	transcription factor
ETOPOSIDE	CHOL	ctx	off label	transcription factor
CARBOPLATIN	CHOL	ctx	off label	transcription factor
OLAPARIB	CHOL	tgt	off label	transcription factor
MITOMYCIN	CHOL	ctx	off label	transcription factor
DABRAFENIB	CHOL	tgt	off label	transcription factor
PEMBROLIZUMAB	CHOL	tgt	off label	transcription factor
TRAMETINIB	CHOL	tgt	off label	transcription factor
VEMURAFENIB	CHOL	tgt	off label	transcription factor
IFOSFAMIDE	CHOL	ctx	off label	transcription factor
THIOTEPA	COADREAD	ctx	off label	specific master
CAPECITABINE	COADREAD	ctx	off label	specific master
CYCLOPHOSPHAMIDE	COADREAD	ctx	off label	specific master
DOCETAXEL	COADREAD	ctx	off label	specific master
EPIRUBICIN	COADREAD	ctx	off label	specific master
EVEROLIMUS	COADREAD	tgt	off label	specific master
FLUOROURACIL	COADREAD	ctx	off label	specific master
FULVESTRANT	COADREAD	tgt	off label	specific master
GEMCITABINE	COADREAD	ctx	off label	specific master
LETROZOLE	COADREAD	tgt	off label	specific master
PACLITAXEL	COADREAD	ctx	off label	specific master
PERTUZUMAB	COADREAD	tgt	off label	specific master
TRASTUZUMAB	COADREAD	tgt	off label	specific master
BEVACIZUMAB	COADREAD	tgt	on label	specific master
CAPECITABINE	COADREAD	ctx	on label	specific master
CETUXIMAB	COADREAD	tgt	on label	specific master
FLUOROURACIL	COADREAD	ctx	on label	specific master
IRINOTECAN	COADREAD	ctx	on label	specific master
OXALIPLATIN	COADREAD	ctx	on label	specific master
PANITUMUMAB	COADREAD	tgt	on label	specific master
REGORAFENIB	COADREAD	tgt	on label	specific master
BEVACIZUMAB	COADREAD	tgt	off label	specific master

TEMOZOLOMIDE	COADREAD	ctx	off label	specific master
CETUXIMAB	COADREAD	tgt	off label	specific master
TEMSIROLIMUS	COADREAD	tgt	off label	specific master
CRIZOTINIB	COADREAD	tgt	off label	specific master
ETOPOSIDE	COADREAD	ctx	off label	specific master
CARBOPLATIN	COADREAD	ctx	off label	specific master
OLAPARIB	COADREAD	tgt	off label	specific master
MITOMYCIN	COADREAD	ctx	off label	specific master
DABRAFENIB	COADREAD	tgt	off label	specific master
PEMBROLIZUMAB	COADREAD	tgt	off label	specific master
TRAMETINIB	COADREAD	tgt	off label	specific master
VEMURAFENIB	COADREAD	tgt	off label	specific master
FLOXURIDINE	COADREAD	ctx	off label	specific master
REGORAFENIB	COADREAD	tgt	off label	specific master
IFOSFAMIDE	COADREAD	ctx	off label	specific master
CABOZANTINIB	COADREAD	tgt	off label	specific master
LEUPROLIDE ACETATE	COADREAD	tgt	off label	transcription factor
THIOTEPA	ESCA	ctx	off label	specific master
CAPECITABINE	ESCA	ctx	off label	specific master
CYCLOPHOSPHAMIDE	ESCA	ctx	off label	specific master
DOCETAXEL	ESCA	ctx	off label	specific master
EPIRUBICIN	ESCA	ctx	off label	specific master
EVEROLIMUS	ESCA	tgt	off label	specific master
FLUOROURACIL	ESCA	ctx	off label	specific master
FULVESTRANT	ESCA	tgt	off label	specific master
GEMCITABINE	ESCA	ctx	off label	specific master
LETROZOLE	ESCA	tgt	off label	specific master
METHOTREXATE	ESCA	ctx	off label	specific master
PACLITAXEL	ESCA	ctx	off label	specific master
PERTUZUMAB	ESCA	tgt	off label	specific master
TRASTUZUMAB	ESCA	tgt	off label	specific master
BEVACIZUMAB	ESCA	tgt	off label	specific master
CETUXIMAB	ESCA	tgt	off label	specific master
IRINOTECAN	ESCA	ctx	off label	specific master
OXALIPLATIN	ESCA	ctx	off label	specific master
PANITUMUMAB	ESCA	tgt	off label	specific master
REGORAFENIB	ESCA	tgt	off label	specific master
TEMOZOLOMIDE	ESCA	ctx	off label	specific master
TEMSIROLIMUS	ESCA	tgt	off label	specific master
CRIZOTINIB	ESCA	tgt	off label	specific master
ETOPOSIDE	ESCA	ctx	off label	specific master

CARBOPLATIN	ESCA	ctx	off label	specific master
OLAPARIB	ESCA	tgt	off label	specific master
MITOMYCIN	ESCA	ctx	off label	specific master
DABRAFENIB	ESCA	tgt	off label	specific master
PEMBROLIZUMAB	ESCA	tgt	off label	specific master
TRAMETINIB	ESCA	tgt	off label	specific master
VEMURAFENIB	ESCA	tgt	off label	specific master
FLOXURIDINE	ESCA	ctx	off label	specific master
IFOSFAMIDE	ESCA	ctx	off label	specific master
CABOZANTINIB	ESCA	tgt	off label	specific master
BICALUTAMIDE	ESCA	tgt	off label	transcription factor
LEUPROLIDE ACETATE	ESCA	tgt	off label	transcription factor
EPIRUBICIN	ESCA	ctx	off label	matrisome
THIOTEPA	GBM	ctx	off label	specific master
CAPECITABINE	GBM	ctx	off label	specific master
CYCLOPHOSPHAMIDE	GBM	ctx	off label	specific master
DOCETAXEL	GBM	ctx	off label	specific master
EPIRUBICIN	GBM	ctx	off label	specific master
EVEROLIMUS	GBM	tgt	off label	specific master
FULVESTRANT	GBM	tgt	off label	specific master
GEMCITABINE	GBM	ctx	off label	specific master
LAPATINIB DITOSYLATE	GBM	tgt	off label	specific master
METHOTREXATE	GBM	ctx	off label	specific master
PACLITAXEL	GBM	ctx	off label	specific master
TRASTUZUMAB	GBM	tgt	off label	specific master
BEVACIZUMAB	GBM	tgt	off label	specific master
CETUXIMAB	GBM	tgt	off label	specific master
IRINOTECAN	GBM	ctx	off label	specific master
OXALIPLATIN	GBM	ctx	off label	specific master
PANITUMUMAB	GBM	tgt	off label	specific master
REGORAFENIB	GBM	tgt	off label	specific master
BEVACIZUMAB	GBM	tgt	on label	specific master
CYCLOPHOSPHAMIDE	GBM	ctx	on label	specific master
DOCETAXEL	GBM	ctx	on label	specific master
EVEROLIMUS	GBM	tgt	on label	specific master
LOMUSTINE	GBM	ctx	on label	specific master
TEMOZOLOMIDE	GBM	ctx	on label	specific master
TEMSIROLIMUS	GBM	tgt	off label	specific master
LOMUSTINE	GBM	ctx	off label	specific master
TEMOZOLOMIDE	GBM	ctx	off label	specific master
AFATINIB	GBM	tgt	off label	specific master

CRIZOTINIB	GBM	tgt	off label	specific master
ERLOTINIB HYDROCHLORIDE	GBM	tgt	off label	specific master
ETOPOSIDE	GBM	ctx	off label	specific master
GEFITINIB	GBM	tgt	off label	specific master
CARBOPLATIN	GBM	ctx	off label	specific master
OLAPARIB	GBM	tgt	off label	specific master
MITOMYCIN	GBM	ctx	off label	specific master
ENZALUTAMIDE	GBM	tgt	off label	specific master
DABRAFENIB	GBM	tgt	off label	specific master
NIVOLUMAB	GBM	tgt	off label	specific master
PEMBROLIZUMAB	GBM	tgt	off label	specific master
TRAMETINIB	GBM	tgt	off label	specific master
VEMURAFENIB	GBM	tgt	off label	specific master
IFOSFAMIDE	GBM	ctx	off label	specific master
CABOZANTINIB	GBM	tgt	off label	specific master
VANDETANIB	GBM	tgt	off label	specific master
THIOTEPA	GBM	ctx	off label	transcription factor
CAPECITABINE	GBM	ctx	off label	transcription factor
CYCLOPHOSPHAMIDE	GBM	ctx	off label	transcription factor
DOCETAXEL	GBM	ctx	off label	transcription factor
EPIRUBICIN	GBM	ctx	off label	transcription factor
GEMCITABINE	GBM	ctx	off label	transcription factor
PACLITAXEL	GBM	ctx	off label	transcription factor
BEVACIZUMAB	GBM	tgt	off label	transcription factor
CETUXIMAB	GBM	tgt	off label	transcription factor
IRINOTECAN	GBM	ctx	off label	transcription factor
OXALIPLATIN	GBM	ctx	off label	transcription factor
PANITUMUMAB	GBM	tgt	off label	transcription factor
BEVACIZUMAB	GBM	tgt	on label	transcription factor
CYCLOPHOSPHAMIDE	GBM	ctx	on label	transcription factor
DOCETAXEL	GBM	ctx	on label	transcription factor
TEMOZOLOMIDE	GBM	ctx	on label	transcription factor
TEMOZOLOMIDE	GBM	ctx	off label	transcription factor
CRIZOTINIB	GBM	tgt	off label	transcription factor
ETOPOSIDE	GBM	ctx	off label	transcription factor
CARBOPLATIN	GBM	ctx	off label	transcription factor
OLAPARIB	GBM	tgt	off label	transcription factor
MITOMYCIN	GBM	ctx	off label	transcription factor
BICALUTAMIDE	GBM	tgt	off label	transcription factor
DABRAFENIB	GBM	tgt	off label	transcription factor
PEMBROLIZUMAB	GBM	tgt	off label	transcription factor

TRAMETINIB	GBM	tgt	off label	transcription factor
VEMURAFENIB	GBM	tgt	off label	transcription factor
IFOSFAMIDE	GBM	ctx	off label	transcription factor
EPIRUBICIN	GBM	ctx	off label	matrisome
AXITINIB	GBM	tgt	off label	matrisome
DACTINOMYCIN	GBM	ctx	off label	matrisome
THIOTEPA	HNSC	ctx	off label	specific master
CAPECITABINE	HNSC	ctx	off label	specific master
CYCLOPHOSPHAMIDE	HNSC	ctx	off label	specific master
DOCETAXEL	HNSC	ctx	off label	specific master
EPIRUBICIN	HNSC	ctx	off label	specific master
EVEROLIMUS	HNSC	tgt	off label	specific master
FLUOROURACIL	HNSC	ctx	off label	specific master
FULVESTRANT	HNSC	tgt	off label	specific master
GEMCITABINE	HNSC	ctx	off label	specific master
LETROZOLE	HNSC	tgt	off label	specific master
METHOTREXATE	HNSC	ctx	off label	specific master
PACLITAXEL	HNSC	ctx	off label	specific master
PERTUZUMAB	HNSC	tgt	off label	specific master
TRASTUZUMAB	HNSC	tgt	off label	specific master
BEVACIZUMAB	HNSC	tgt	off label	specific master
CETUXIMAB	HNSC	tgt	off label	specific master
IRINOTECAN	HNSC	ctx	off label	specific master
OXALIPLATIN	HNSC	ctx	off label	specific master
PANITUMUMAB	HNSC	tgt	off label	specific master
REGORAFENIB	HNSC	tgt	off label	specific master
TEMOZOLOMIDE	HNSC	ctx	off label	specific master
CETUXIMAB	HNSC	tgt	on label	specific master
DOCETAXEL	HNSC	ctx	on label	specific master
METHOTREXATE	HNSC	ctx	on label	specific master
TEMSIROLIMUS	HNSC	tgt	off label	specific master
CRIZOTINIB	HNSC	tgt	off label	specific master
ETOPOSIDE	HNSC	ctx	off label	specific master
CARBOPLATIN	HNSC	ctx	off label	specific master
OLAPARIB	HNSC	tgt	off label	specific master
MITOMYCIN	HNSC	ctx	off label	specific master
FLUTAMIDE	HNSC	tgt	off label	specific master
DABRAFENIB	HNSC	tgt	off label	specific master
PEMBROLIZUMAB	HNSC	tgt	off label	specific master
TRAMETINIB	HNSC	tgt	off label	specific master
VEMURAFENIB	HNSC	tgt	off label	specific master

FLOXURIDINE	HNSC	ctx	off label	specific master
IFOSFAMIDE	HNSC	ctx	off label	specific master
CABOZANTINIB	HNSC	tgt	off label	specific master
PACLITAXEL	HNSC	ctx	off label	transcription factor
CETUXIMAB	HNSC	tgt	off label	transcription factor
CETUXIMAB	HNSC	tgt	on label	transcription factor
BICALUTAMIDE	HNSC	tgt	off label	transcription factor
LEUPROLIDE ACETATE	HNSC	tgt	off label	transcription factor
EPIRUBICIN	HNSC	ctx	off label	matrisome
THIOTEPA	KICH	ctx	off label	specific master
CAPECITABINE	KICH	ctx	off label	specific master
CYCLOPHOSPHAMIDE	KICH	ctx	off label	specific master
DOCETAXEL	KICH	ctx	off label	specific master
EPIRUBICIN	KICH	ctx	off label	specific master
GEMCITABINE	KICH	ctx	off label	specific master
PACLITAXEL	KICH	ctx	off label	specific master
BEVACIZUMAB	KICH	tgt	off label	specific master
CETUXIMAB	KICH	tgt	off label	specific master
IRINOTECAN	KICH	ctx	off label	specific master
OXALIPLATIN	KICH	ctx	off label	specific master
PANITUMUMAB	KICH	tgt	off label	specific master
TEMOZOLOMIDE	KICH	ctx	off label	specific master
BEVACIZUMAB	KICH	tgt	on label	specific master
CRIZOTINIB	KICH	tgt	off label	specific master
ETOPOSIDE	KICH	ctx	off label	specific master
CARBOPLATIN	KICH	ctx	off label	specific master
OLAPARIB	KICH	tgt	off label	specific master
MITOMYCIN	KICH	ctx	off label	specific master
DABRAFENIB	KICH	tgt	off label	specific master
PEMBROLIZUMAB	KICH	tgt	off label	specific master
TRAMETINIB	KICH	tgt	off label	specific master
VEMURAFENIB	KICH	tgt	off label	specific master
IFOSFAMIDE	KICH	ctx	off label	specific master
METHOTREXATE	KICH	ctx	off label	transcription factor
LEUPROLIDE ACETATE	KICH	tgt	off label	transcription factor
DOCETAXEL	KICH	ctx	off label	matrisome
EVEROLIMUS	KIRC	tgt	off label	specific master
BEVACIZUMAB	KIRC	tgt	off label	specific master
REGORAFENIB	KIRC	tgt	off label	specific master
AXITINIB	KIRC	tgt	off label	specific master
TEMSIROLIMUS	KIRC	tgt	off label	specific master

AXITINIB	KIRC	tgt	on label	specific master
BEVACIZUMAB	KIRC	tgt	on label	specific master
EVEROLIMUS	KIRC	tgt	on label	specific master
TEMSIROLIMUS	KIRC	tgt	on label	specific master
OLAPARIB	KIRC	tgt	off label	specific master
TRAMETINIB	KIRC	tgt	off label	specific master
THIOTEPA	KIRC	ctx	off label	transcription factor
CAPECITABINE	KIRC	ctx	off label	transcription factor
CYCLOPHOSPHAMIDE	KIRC	ctx	off label	transcription factor
DOCETAXEL	KIRC	ctx	off label	transcription factor
EPIRUBICIN	KIRC	ctx	off label	transcription factor
GEMCITABINE	KIRC	ctx	off label	transcription factor
PACLITAXEL	KIRC	ctx	off label	transcription factor
BEVACIZUMAB	KIRC	tgt	off label	transcription factor
CETUXIMAB	KIRC	tgt	off label	transcription factor
IRINOTECAN	KIRC	ctx	off label	transcription factor
OXALIPLATIN	KIRC	ctx	off label	transcription factor
PANITUMUMAB	KIRC	tgt	off label	transcription factor
TEMOZOLOMIDE	KIRC	ctx	off label	transcription factor
BEVACIZUMAB	KIRC	tgt	on label	transcription factor
CRIZOTINIB	KIRC	tgt	off label	transcription factor
ETOPOSIDE	KIRC	ctx	off label	transcription factor
CARBOPLATIN	KIRC	ctx	off label	transcription factor
OLAPARIB	KIRC	tgt	off label	transcription factor
MITOMYCIN	KIRC	ctx	off label	transcription factor
DABRAFENIB	KIRC	tgt	off label	transcription factor
PEMBROLIZUMAB	KIRC	tgt	off label	transcription factor
TRAMETINIB	KIRC	tgt	off label	transcription factor
VEMURAFENIB	KIRC	tgt	off label	transcription factor
IFOSFAMIDE	KIRC	ctx	off label	transcription factor
AXITINIB	KIRC	tgt	off label	matrisome
DACTINOMYCIN	KIRC	ctx	off label	matrisome
AXITINIB	KIRC	tgt	on label	matrisome
DACTINOMYCIN	KIRC	ctx	on label	matrisome
THIOTEPA	LAML	ctx	off label	specific master
CAPECITABINE	LAML	ctx	off label	specific master
CYCLOPHOSPHAMIDE	LAML	ctx	off label	specific master
DOCETAXEL	LAML	ctx	off label	specific master
EPIRUBICIN	LAML	ctx	off label	specific master
GEMCITABINE	LAML	ctx	off label	specific master
PACLITAXEL	LAML	ctx	off label	specific master

BEVACIZUMAB	LAML	tgt	off label	specific master
CETUXIMAB	LAML	tgt	off label	specific master
IRINOTECAN	LAML	ctx	off label	specific master
OXALIPLATIN	LAML	ctx	off label	specific master
PANITUMUMAB	LAML	tgt	off label	specific master
TEMOZOLOMIDE	LAML	ctx	off label	specific master
CERITINIB	LAML	tgt	off label	specific master
CRIZOTINIB	LAML	tgt	off label	specific master
ETOPOSIDE	LAML	ctx	off label	specific master
CARBOPLATIN	LAML	ctx	off label	specific master
OLAPARIB	LAML	tgt	off label	specific master
MITOMYCIN	LAML	ctx	off label	specific master
DABRAFENIB	LAML	tgt	off label	specific master
PEMBROLIZUMAB	LAML	tgt	off label	specific master
TRAMETINIB	LAML	tgt	off label	specific master
VEMURAFENIB	LAML	tgt	off label	specific master
IFOSFAMIDE	LAML	ctx	off label	specific master
CETUXIMAB	LAML	tgt	off label	transcription factor
BICALUTAMIDE	LAML	tgt	off label	transcription factor
LEUPROLIDE ACETATE	LAML	tgt	off label	transcription factor
THIOTEPA	LGG	ctx	off label	specific master
CAPECITABINE	LGG	ctx	off label	specific master
CYCLOPHOSPHAMIDE	LGG	ctx	off label	specific master
DOCETAXEL	LGG	ctx	off label	specific master
EPIRUBICIN	LGG	ctx	off label	specific master
EVEROLIMUS	LGG	tgt	off label	specific master
GEMCITABINE	LGG	ctx	off label	specific master
LAPATINIB DITOSYLATE	LGG	tgt	off label	specific master
METHOTREXATE	LGG	ctx	off label	specific master
PACLITAXEL	LGG	ctx	off label	specific master
TRASTUZUMAB	LGG	tgt	off label	specific master
BEVACIZUMAB	LGG	tgt	off label	specific master
CETUXIMAB	LGG	tgt	off label	specific master
IRINOTECAN	LGG	ctx	off label	specific master
OXALIPLATIN	LGG	ctx	off label	specific master
PANITUMUMAB	LGG	tgt	off label	specific master
REGORAFENIB	LGG	tgt	off label	specific master
TEMOZOLOMIDE	LGG	ctx	off label	specific master
TEMSIROLIMUS	LGG	tgt	off label	specific master
BEVACIZUMAB	LGG	tgt	on label	specific master
CYCLOPHOSPHAMIDE	LGG	ctx	on label	specific master

DOCETAXEL	LGG	ctx	on label	specific master
EVEROLIMUS	LGG	tgt	on label	specific master
TEMOZOLOMIDE	LGG	ctx	on label	specific master
AFATINIB	LGG	tgt	off label	specific master
CRIZOTINIB	LGG	tgt	off label	specific master
ERLOTINIB HYDROCHLORIDE	LGG	tgt	off label	specific master
ETOPOSIDE	LGG	ctx	off label	specific master
GEFITINIB	LGG	tgt	off label	specific master
CARBOPLATIN	LGG	ctx	off label	specific master
OLAPARIB	LGG	tgt	off label	specific master
MITOMYCIN	LGG	ctx	off label	specific master
DABRAFENIB	LGG	tgt	off label	specific master
NIVOLUMAB	LGG	tgt	off label	specific master
PEMBROLIZUMAB	LGG	tgt	off label	specific master
TRAMETINIB	LGG	tgt	off label	specific master
VEMURAFENIB	LGG	tgt	off label	specific master
IFOSFAMIDE	LGG	ctx	off label	specific master
CABOZANTINIB	LGG	tgt	off label	specific master
VANDETANIB	LGG	tgt	off label	specific master
METHOTREXATE	LGG	ctx	off label	transcription factor
FULVESTRANT	LGG	tgt	off label	matrisome
AXITINIB	LGG	tgt	off label	matrisome
DACTINOMYCIN	LGG	ctx	off label	matrisome
THIOTEPA	LUAD	ctx	off label	specific master
CAPECITABINE	LUAD	ctx	off label	specific master
CYCLOPHOSPHAMIDE	LUAD	ctx	off label	specific master
DOCETAXEL	LUAD	ctx	off label	specific master
EPIRUBICIN	LUAD	ctx	off label	specific master
EVEROLIMUS	LUAD	tgt	off label	specific master
GEMCITABINE	LUAD	ctx	off label	specific master
LAPATINIB DITOSYLATE	LUAD	tgt	off label	specific master
PACLITAXEL	LUAD	ctx	off label	specific master
TRASTUZUMAB	LUAD	tgt	off label	specific master
BEVACIZUMAB	LUAD	tgt	off label	specific master
CETUXIMAB	LUAD	tgt	off label	specific master
IRINOTECAN	LUAD	ctx	off label	specific master
OXALIPLATIN	LUAD	ctx	off label	specific master
PANITUMUMAB	LUAD	tgt	off label	specific master
REGORAFENIB	LUAD	tgt	off label	specific master
TEMOZOLOMIDE	LUAD	ctx	off label	specific master
TEMSIROLIMUS	LUAD	tgt	off label	specific master

AFATINIB	LUAD	tgt	on label	specific master
BEVACIZUMAB	LUAD	tgt	on label	specific master
CRIZOTINIB	LUAD	tgt	on label	specific master
DOCETAXEL	LUAD	ctx	on label	specific master
ERLOTINIB HYDROCHLORIDE	LUAD	tgt	on label	specific master
ETOPOSIDE	LUAD	ctx	on label	specific master
GEFITINIB	LUAD	tgt	on label	specific master
GEMCITABINE	LUAD	ctx	on label	specific master
PACLITAXEL	LUAD	ctx	on label	specific master
AFATINIB	LUAD	tgt	off label	specific master
CRIZOTINIB	LUAD	tgt	off label	specific master
ERLOTINIB HYDROCHLORIDE	LUAD	tgt	off label	specific master
ETOPOSIDE	LUAD	ctx	off label	specific master
GEFITINIB	LUAD	tgt	off label	specific master
CARBOPLATIN	LUAD	ctx	off label	specific master
OLAPARIB	LUAD	tgt	off label	specific master
MITOMYCIN	LUAD	ctx	off label	specific master
DABRAFENIB	LUAD	tgt	off label	specific master
NIVOLUMAB	LUAD	tgt	off label	specific master
PEMBROLIZUMAB	LUAD	tgt	off label	specific master
TRAMETINIB	LUAD	tgt	off label	specific master
VEMURAFENIB	LUAD	tgt	off label	specific master
IFOSFAMIDE	LUAD	ctx	off label	specific master
CABOZANTINIB	LUAD	tgt	off label	specific master
VANDETANIB	LUAD	tgt	off label	specific master
BICALUTAMIDE	LUAD	tgt	off label	transcription factor
LEUPROLIDE ACETATE	LUAD	tgt	off label	transcription factor
THIOTEPA	LUSC	ctx	off label	specific master
CAPECITABINE	LUSC	ctx	off label	specific master
CYCLOPHOSPHAMIDE	LUSC	ctx	off label	specific master
DOCETAXEL	LUSC	ctx	off label	specific master
EPIRUBICIN	LUSC	ctx	off label	specific master
EVEROLIMUS	LUSC	tgt	off label	specific master
GEMCITABINE	LUSC	ctx	off label	specific master
METHOTREXATE	LUSC	ctx	off label	specific master
PACLITAXEL	LUSC	ctx	off label	specific master
TRASTUZUMAB	LUSC	tgt	off label	specific master
BEVACIZUMAB	LUSC	tgt	off label	specific master
CETUXIMAB	LUSC	tgt	off label	specific master
IRINOTECAN	LUSC	ctx	off label	specific master
OXALIPLATIN	LUSC	ctx	off label	specific master

PANITUMUMAB	LUSC	tgt	off label	specific master
REGORAFENIB	LUSC	tgt	off label	specific master
TEMOZOLOMIDE	LUSC	ctx	off label	specific master
TEMSIROLIMUS	LUSC	tgt	off label	specific master
CRIZOTINIB	LUSC	tgt	off label	specific master
ETOPOSIDE	LUSC	ctx	off label	specific master
BEVACIZUMAB	LUSC	tgt	on label	specific master
CRIZOTINIB	LUSC	tgt	on label	specific master
DOCETAXEL	LUSC	ctx	on label	specific master
ETOPOSIDE	LUSC	ctx	on label	specific master
GEMCITABINE	LUSC	ctx	on label	specific master
METHOTREXATE	LUSC	ctx	on label	specific master
PACLITAXEL	LUSC	ctx	on label	specific master
CARBOPLATIN	LUSC	ctx	off label	specific master
OLAPARIB	LUSC	tgt	off label	specific master
MITOMYCIN	LUSC	ctx	off label	specific master
FLUTAMIDE	LUSC	tgt	off label	specific master
DABRAFENIB	LUSC	tgt	off label	specific master
PEMBROLIZUMAB	LUSC	tgt	off label	specific master
TRAMETINIB	LUSC	tgt	off label	specific master
VEMURAFENIB	LUSC	tgt	off label	specific master
IFOSFAMIDE	LUSC	ctx	off label	specific master
ANASTROZOLE	LUSC	tgt	off label	transcription factor
LETROZOLE	LUSC	tgt	off label	transcription factor
TOREMIFENE	LUSC	tgt	off label	transcription factor
TRASTUZUMAB	LUSC	tgt	off label	transcription factor
FLUTAMIDE	LUSC	tgt	off label	transcription factor
OXALIPLATIN	LUSC	ctx	off label	matrisome
THIOTEPA	MESO	ctx	off label	specific master
CAPECITABINE	MESO	ctx	off label	specific master
CYCLOPHOSPHAMIDE	MESO	ctx	off label	specific master
DOCETAXEL	MESO	ctx	off label	specific master
EPIRUBICIN	MESO	ctx	off label	specific master
EVEROLIMUS	MESO	tgt	off label	specific master
GEMCITABINE	MESO	ctx	off label	specific master
PACLITAXEL	MESO	ctx	off label	specific master
BEVACIZUMAB	MESO	tgt	off label	specific master
CETUXIMAB	MESO	tgt	off label	specific master
IRINOTECAN	MESO	ctx	off label	specific master
OXALIPLATIN	MESO	ctx	off label	specific master
PANITUMUMAB	MESO	tgt	off label	specific master

TEMOZOLOMIDE	MESO	ctx	off label	specific master
TEMSIROLIMUS	MESO	tgt	off label	specific master
CRIZOTINIB	MESO	tgt	off label	specific master
ETOPOSIDE	MESO	ctx	off label	specific master
CARBOPLATIN	MESO	ctx	off label	specific master
OLAPARIB	MESO	tgt	off label	specific master
MITOMYCIN	MESO	ctx	off label	specific master
DABRAFENIB	MESO	tgt	off label	specific master
PEMBROLIZUMAB	MESO	tgt	off label	specific master
TRAMETINIB	MESO	tgt	off label	specific master
VEMURAFENIB	MESO	tgt	off label	specific master
IFOSFAMIDE	MESO	ctx	off label	specific master
THIOTEPA	PAAD	ctx	off label	specific master
CAPECITABINE	PAAD	ctx	off label	specific master
CYCLOPHOSPHAMIDE	PAAD	ctx	off label	specific master
DOCETAXEL	PAAD	ctx	off label	specific master
EPIRUBICIN	PAAD	ctx	off label	specific master
FLUOROURACIL	PAAD	ctx	off label	specific master
GEMCITABINE	PAAD	ctx	off label	specific master
PACLITAXEL	PAAD	ctx	off label	specific master
TRASTUZUMAB	PAAD	tgt	off label	specific master
BEVACIZUMAB	PAAD	tgt	off label	specific master
CETUXIMAB	PAAD	tgt	off label	specific master
IRINOTECAN	PAAD	ctx	off label	specific master
OXALIPLATIN	PAAD	ctx	off label	specific master
PANITUMUMAB	PAAD	tgt	off label	specific master
TEMOZOLOMIDE	PAAD	ctx	off label	specific master
CRIZOTINIB	PAAD	tgt	off label	specific master
ETOPOSIDE	PAAD	ctx	off label	specific master
CARBOPLATIN	PAAD	ctx	off label	specific master
OLAPARIB	PAAD	tgt	off label	specific master
FLUOROURACIL	PAAD	ctx	on label	specific master
GEMCITABINE	PAAD	ctx	on label	specific master
MITOMYCIN	PAAD	ctx	on label	specific master
PACLITAXEL	PAAD	ctx	on label	specific master
DABRAFENIB	PAAD	tgt	off label	specific master
PEMBROLIZUMAB	PAAD	tgt	off label	specific master
TRAMETINIB	PAAD	tgt	off label	specific master
VEMURAFENIB	PAAD	tgt	off label	specific master
MITOMYCIN	PAAD	ctx	off label	specific master
IFOSFAMIDE	PAAD	ctx	off label	specific master

LEUPROLIDE ACETATE	PAAD	tgt	off label	transcription factor
DOCETAXEL	PCPG	ctx	off label	specific master
EVEROLIMUS	PCPG	tgt	off label	specific master
GEMCITABINE	PCPG	ctx	off label	specific master
BEVACIZUMAB	PCPG	tgt	off label	specific master
CETUXIMAB	PCPG	tgt	off label	specific master
PANITUMUMAB	PCPG	tgt	off label	specific master
HYDROXYUREA	PCPG	tgt	off label	specific master
TRAMETINIB	PCPG	tgt	off label	specific master
CABOZANTINIB	PCPG	tgt	off label	specific master
THIOTEPA	PCPG	ctx	off label	transcription factor
CAPECITABINE	PCPG	ctx	off label	transcription factor
CYCLOPHOSPHAMIDE	PCPG	ctx	off label	transcription factor
DOCETAXEL	PCPG	ctx	off label	transcription factor
EPIRUBICIN	PCPG	ctx	off label	transcription factor
GEMCITABINE	PCPG	ctx	off label	transcription factor
PACLITAXEL	PCPG	ctx	off label	transcription factor
BEVACIZUMAB	PCPG	tgt	off label	transcription factor
CETUXIMAB	PCPG	tgt	off label	transcription factor
IRINOTECAN	PCPG	ctx	off label	transcription factor
OXALIPLATIN	PCPG	ctx	off label	transcription factor
PANITUMUMAB	PCPG	tgt	off label	transcription factor
TEMOZOLOMIDE	PCPG	ctx	off label	transcription factor
CRIZOTINIB	PCPG	tgt	off label	transcription factor
ETOPOSIDE	PCPG	ctx	off label	transcription factor
CARBOPLATIN	PCPG	ctx	off label	transcription factor
OLAPARIB	PCPG	tgt	off label	transcription factor
MITOMYCIN	PCPG	ctx	off label	transcription factor
DABRAFENIB	PCPG	tgt	off label	transcription factor
PEMBROLIZUMAB	PCPG	tgt	off label	transcription factor
TRAMETINIB	PCPG	tgt	off label	transcription factor
VEMURAFENIB	PCPG	tgt	off label	transcription factor
IFOSFAMIDE	PCPG	ctx	off label	transcription factor
DOCETAXEL	PCPG	ctx	off label	matrisome
THIOTEPA	PRAD	ctx	off label	specific master
CAPECITABINE	PRAD	ctx	off label	specific master
CYCLOPHOSPHAMIDE	PRAD	ctx	off label	specific master
DOCETAXEL	PRAD	ctx	off label	specific master
EPIRUBICIN	PRAD	ctx	off label	specific master
GEMCITABINE	PRAD	ctx	off label	specific master
PACLITAXEL	PRAD	ctx	off label	specific master

BEVACIZUMAB	PRAD	tgt	off label	specific master
CETUXIMAB	PRAD	tgt	off label	specific master
IRINOTECAN	PRAD	ctx	off label	specific master
OXALIPLATIN	PRAD	ctx	off label	specific master
PANITUMUMAB	PRAD	tgt	off label	specific master
TEMOZOLOMIDE	PRAD	ctx	off label	specific master
CRIZOTINIB	PRAD	tgt	off label	specific master
ETOPOSIDE	PRAD	ctx	off label	specific master
CARBOPLATIN	PRAD	ctx	off label	specific master
OLAPARIB	PRAD	tgt	off label	specific master
MITOMYCIN	PRAD	ctx	off label	specific master
DOCETAXEL	PRAD	ctx	on label	specific master
DABRAFENIB	PRAD	tgt	off label	specific master
PEMBROLIZUMAB	PRAD	tgt	off label	specific master
TRAMETINIB	PRAD	tgt	off label	specific master
VEMURAFENIB	PRAD	tgt	off label	specific master
IFOSFAMIDE	PRAD	ctx	off label	specific master
THIOTEPA	PRAD	ctx	off label	transcription factor
CAPECITABINE	PRAD	ctx	off label	transcription factor
CYCLOPHOSPHAMIDE	PRAD	ctx	off label	transcription factor
DOCETAXEL	PRAD	ctx	off label	transcription factor
EPIRUBICIN	PRAD	ctx	off label	transcription factor
GEMCITABINE	PRAD	ctx	off label	transcription factor
PACLITAXEL	PRAD	ctx	off label	transcription factor
BEVACIZUMAB	PRAD	tgt	off label	transcription factor
CETUXIMAB	PRAD	tgt	off label	transcription factor
IRINOTECAN	PRAD	ctx	off label	transcription factor
OXALIPLATIN	PRAD	ctx	off label	transcription factor
PANITUMUMAB	PRAD	tgt	off label	transcription factor
TEMOZOLOMIDE	PRAD	ctx	off label	transcription factor
CRIZOTINIB	PRAD	tgt	off label	transcription factor
ETOPOSIDE	PRAD	ctx	off label	transcription factor
CARBOPLATIN	PRAD	ctx	off label	transcription factor
OLAPARIB	PRAD	tgt	off label	transcription factor
MITOMYCIN	PRAD	ctx	off label	transcription factor
DOCETAXEL	PRAD	ctx	on label	transcription factor
DABRAFENIB	PRAD	tgt	off label	transcription factor
PEMBROLIZUMAB	PRAD	tgt	off label	transcription factor
TRAMETINIB	PRAD	tgt	off label	transcription factor
VEMURAFENIB	PRAD	tgt	off label	transcription factor
IFOSFAMIDE	PRAD	ctx	off label	transcription factor

FLUOROURACIL	PRAD	ctx	off label	matrisome
CETUXIMAB	PRAD	tgt	off label	matrisome
THIOTEPA	SARC	ctx	off label	specific master
CAPECITABINE	SARC	ctx	off label	specific master
CYCLOPHOSPHAMIDE	SARC	ctx	off label	specific master
DOCETAXEL	SARC	ctx	off label	specific master
EPIRUBICIN	SARC	ctx	off label	specific master
EVEROLIMUS	SARC	tgt	off label	specific master
GEMCITABINE	SARC	ctx	off label	specific master
METHOTREXATE	SARC	ctx	off label	specific master
PACLITAXEL	SARC	ctx	off label	specific master
BEVACIZUMAB	SARC	tgt	off label	specific master
CETUXIMAB	SARC	tgt	off label	specific master
IRINOTECAN	SARC	ctx	off label	specific master
OXALIPLATIN	SARC	ctx	off label	specific master
PANITUMUMAB	SARC	tgt	off label	specific master
TEMOZOLOMIDE	SARC	ctx	off label	specific master
CRIZOTINIB	SARC	tgt	off label	specific master
ETOPOSIDE	SARC	ctx	off label	specific master
CARBOPLATIN	SARC	ctx	off label	specific master
OLAPARIB	SARC	tgt	off label	specific master
MITOMYCIN	SARC	ctx	off label	specific master
METHOTREXATE	SARC	ctx	on label	specific master
PACLITAXEL	SARC	ctx	on label	specific master
DABRAFENIB	SARC	tgt	off label	specific master
PEMBROLIZUMAB	SARC	tgt	off label	specific master
TRAMETINIB	SARC	tgt	off label	specific master
VEMURAFENIB	SARC	tgt	off label	specific master
IFOSFAMIDE	SARC	ctx	off label	specific master
BICALUTAMIDE	SARC	tgt	off label	transcription factor
THIOTEPA	STAD	ctx	off label	specific master
CAPECITABINE	STAD	ctx	off label	specific master
CYCLOPHOSPHAMIDE	STAD	ctx	off label	specific master
DOCETAXEL	STAD	ctx	off label	specific master
EPIRUBICIN	STAD	ctx	off label	specific master
EVEROLIMUS	STAD	tgt	off label	specific master
FLUOROURACIL	STAD	ctx	off label	specific master
FULVESTRANT	STAD	tgt	off label	specific master
GEMCITABINE	STAD	ctx	off label	specific master
LETROZOLE	STAD	tgt	off label	specific master
PACLITAXEL	STAD	ctx	off label	specific master

PERTUZUMAB	STAD	tgt	off label	specific master
TRASTUZUMAB	STAD	tgt	off label	specific master
BEVACIZUMAB	STAD	tgt	off label	specific master
CETUXIMAB	STAD	tgt	off label	specific master
IRINOTECAN	STAD	ctx	off label	specific master
LEUCOVORIN	STAD	ctx	off label	specific master
OXALIPLATIN	STAD	ctx	off label	specific master
PANITUMUMAB	STAD	tgt	off label	specific master
REGORAFENIB	STAD	tgt	off label	specific master
TEMOZOLOMIDE	STAD	ctx	off label	specific master
TEMSIROLIMUS	STAD	tgt	off label	specific master
AFATINIB	STAD	tgt	off label	specific master
CRIZOTINIB	STAD	tgt	off label	specific master
ETOPOSIDE	STAD	ctx	off label	specific master
GEFITINIB	STAD	tgt	off label	specific master
CARBOPLATIN	STAD	ctx	off label	specific master
OLAPARIB	STAD	tgt	off label	specific master
MITOMYCIN	STAD	ctx	off label	specific master
DABRAFENIB	STAD	tgt	off label	specific master
PEMBROLIZUMAB	STAD	tgt	off label	specific master
TRAMETINIB	STAD	tgt	off label	specific master
VEMURAFENIB	STAD	tgt	off label	specific master
DOCETAXEL	STAD	ctx	on label	specific master
FLOXURIDINE	STAD	ctx	on label	specific master
FLUOROURACIL	STAD	ctx	on label	specific master
MITOMYCIN	STAD	ctx	on label	specific master
REGORAFENIB	STAD	tgt	on label	specific master
TRASTUZUMAB	STAD	tgt	on label	specific master
IFOSFAMIDE	STAD	ctx	off label	specific master
CABOZANTINIB	STAD	tgt	off label	specific master
VANDETANIB	STAD	tgt	off label	specific master
CETUXIMAB	STAD	tgt	off label	transcription factor
BICALUTAMIDE	STAD	tgt	off label	transcription factor
LEUPROLIDE ACETATE	STAD	tgt	off label	transcription factor
THIOTEPA	UCEC	ctx	off label	specific master
CAPECITABINE	UCEC	ctx	off label	specific master
CYCLOPHOSPHAMIDE	UCEC	ctx	off label	specific master
DOCETAXEL	UCEC	ctx	off label	specific master
EPIRUBICIN	UCEC	ctx	off label	specific master
EVEROLIMUS	UCEC	tgt	off label	specific master
GEMCITABINE	UCEC	ctx	off label	specific master

LETROZOLE	UCEC	tgt	off label	specific master
METHOTREXATE	UCEC	ctx	off label	specific master
PACLITAXEL	UCEC	ctx	off label	specific master
TRASTUZUMAB	UCEC	tgt	off label	specific master
BEVACIZUMAB	UCEC	tgt	off label	specific master
CETUXIMAB	UCEC	tgt	off label	specific master
IRINOTECAN	UCEC	ctx	off label	specific master
OXALIPLATIN	UCEC	ctx	off label	specific master
PANITUMUMAB	UCEC	tgt	off label	specific master
REGORAFENIB	UCEC	tgt	off label	specific master
TEMOZOLOMIDE	UCEC	ctx	off label	specific master
TEMSIROLIMUS	UCEC	tgt	off label	specific master
CRIZOTINIB	UCEC	tgt	off label	specific master
ETOPOSIDE	UCEC	ctx	off label	specific master
CARBOPLATIN	UCEC	ctx	off label	specific master
OLAPARIB	UCEC	tgt	off label	specific master
MITOMYCIN	UCEC	ctx	off label	specific master
FLUTAMIDE	UCEC	tgt	off label	specific master
DABRAFENIB	UCEC	tgt	off label	specific master
PEMBROLIZUMAB	UCEC	tgt	off label	specific master
TRAMETINIB	UCEC	tgt	off label	specific master
VEMURAFENIB	UCEC	tgt	off label	specific master
IFOSFAMIDE	UCEC	ctx	off label	specific master
PACLITAXEL	UCEC	ctx	off label	transcription factor
THIOTEPA	UCS	ctx	off label	specific master
CAPECITABINE	UCS	ctx	off label	specific master
CYCLOPHOSPHAMIDE	UCS	ctx	off label	specific master
DOCETAXEL	UCS	ctx	off label	specific master
EPIRUBICIN	UCS	ctx	off label	specific master
GEMCITABINE	UCS	ctx	off label	specific master
PACLITAXEL	UCS	ctx	off label	specific master
TRASTUZUMAB	UCS	tgt	off label	specific master
BEVACIZUMAB	UCS	tgt	off label	specific master
CETUXIMAB	UCS	tgt	off label	specific master
IRINOTECAN	UCS	ctx	off label	specific master
OXALIPLATIN	UCS	ctx	off label	specific master
PANITUMUMAB	UCS	tgt	off label	specific master
TEMOZOLOMIDE	UCS	ctx	off label	specific master
CRIZOTINIB	UCS	tgt	off label	specific master
ETOPOSIDE	UCS	ctx	off label	specific master
CARBOPLATIN	UCS	ctx	off label	specific master

OLAPARIB	UCS	tgt	off label	specific master
MITOMYCIN	UCS	ctx	off label	specific master
DABRAFENIB	UCS	tgt	off label	specific master
PEMBROLIZUMAB	UCS	tgt	off label	specific master
TRAMETINIB	UCS	tgt	off label	specific master
VEMURAFENIB	UCS	tgt	off label	specific master
IFOSFAMIDE	UCS	ctx	off label	specific master
CETUXIMAB	UCS	tgt	off label	transcription factor
THIOTEPA	PANCAN	ctx	off label	general master
CAPECITABINE	PANCAN	ctx	off label	general master
CYCLOPHOSPHAMIDE	PANCAN	ctx	off label	general master
DOCETAXEL	PANCAN	ctx	off label	general master
EPIRUBICIN	PANCAN	ctx	off label	general master
EVEROLIMUS	PANCAN	tgt	off label	general master
FLUOROURACIL	PANCAN	ctx	off label	general master
FULVESTRANT	PANCAN	tgt	off label	general master
GEMCITABINE	PANCAN	ctx	off label	general master
LETROZOLE	PANCAN	tgt	off label	general master
METHOTREXATE	PANCAN	ctx	off label	general master
PACLITAXEL	PANCAN	ctx	off label	general master
PERTUZUMAB	PANCAN	tgt	off label	general master
TRASTUZUMAB	PANCAN	tgt	off label	general master
BEVACIZUMAB	PANCAN	tgt	off label	general master
CETUXIMAB	PANCAN	tgt	off label	general master
IRINOTECAN	PANCAN	ctx	off label	general master
OXALIPLATIN	PANCAN	ctx	off label	general master
PANITUMUMAB	PANCAN	tgt	off label	general master
REGORAFENIB	PANCAN	tgt	off label	general master
TEMOZOLOMIDE	PANCAN	ctx	off label	general master
TEMSIROLIMUS	PANCAN	tgt	off label	general master
CRIZOTINIB	PANCAN	tgt	off label	general master
ETOPOSIDE	PANCAN	ctx	off label	general master
CARBOPLATIN	PANCAN	ctx	off label	general master
OLAPARIB	PANCAN	tgt	off label	general master
MITOMYCIN	PANCAN	ctx	off label	general master
DABRAFENIB	PANCAN	tgt	off label	general master
PEMBROLIZUMAB	PANCAN	tgt	off label	general master
TRAMETINIB	PANCAN	tgt	off label	general master
VEMURAFENIB	PANCAN	tgt	off label	general master
FLOXURIDINE	PANCAN	ctx	off label	general master
IFOSFAMIDE	PANCAN	ctx	off label	general master

CABOZANTINIB	PANCAN	tgt	off label	general master
VANDETANIB	PANCAN	tgt	off label	general master
LAPATINIB DITOSYLATE	PANCAN	tgt	off label	general master
LEUCOVORIN	PANCAN	ctx	off label	general master
AXITINIB	PANCAN	tgt	off label	general master
AFATINIB	PANCAN	tgt	off label	general master
CERITINIB	PANCAN	tgt	off label	general master
ERLOTINIB HYDROCHLORIDE	PANCAN	tgt	off label	general master
GEFITINIB	PANCAN	tgt	off label	general master
FLUTAMIDE	PANCAN	tgt	off label	general master
NIVOLUMAB	PANCAN	tgt	off label	general master
LOMUSTINE	PANCAN	ctx	off label	general master
ENZALUTAMIDE	PANCAN	tgt	off label	general master
BICALUTAMIDE	PANCAN	tgt	off label	general master

Drugs targeting the cancer matrisome. Results are divided by tumor type, gross mechanism of action (ctx, cytotoxic drug; tgt, targeted drug), potential use (on or off label) and molecular target (Pan-Cancer – “general” or cancer-specific – “specific” – master regulators, transcription factors and matrisome molecules). Drugs with Pan-Cancer activity are reported in red.

Supplemental Table 11. List of FANTOM5 samples

**Sample ID**	Tumor(s)
CNhs11893	ACC
CNhs12848	BLCA
CNhs11256	BLCA
CNhs10733	BLCA
CNhs10735	BLCA
CNhs11261	BLCA
CNhs11844	BRCA
CNhs11943	BRCA
CNhs10736	BRCA
CNhs11100	BRCA
CNhs11259	BRCA
CNhs10748	CESC
CNhs12325	CESC
CNhs12326	CESC
CNhs12327	CESC
CNhs11825	CESC
CNhs11885	CESC
CNhs10750	CHOL
CNhs11265	CHOL
CNhs11283	CHOL
CNhs11280	COAD; READ
CNhs10737	COAD; READ
CNhs10734	COAD; READ
CNhs11255	COAD; READ
CNhs11252	ESCA
CNhs11889	HNSC
CNhs10732	HNSC
CNhs10752	HNSC
CNhs11287	HNSC
CNhs11717	HNSC
CNhs11810	HNSC
CNhs11849	HNSC
CNhs11892	KICH; KIRP; KIRC
CNhs11728	KICH; KIRP; KIRC
CNhs10729	KICH; KIRP; KIRC
CNhs11257	KICH; KIRP; KIRC

CNhs13053	LAML
CNhs13054	LAML
CNhs13502	LAML
CNhs13052	LAML
CNhs11864	LAML
CNhs13055	LAML
CNhs13503	LAML
CNhs13504	LAML
CNhs13056	LAML
CNhs13057	LAML
CNhs13050	LAML
CNhs13051	LAML
CNhs10722	LAML
CNhs10723	LAML
CNhs10724	LAML
CNhs13058	LAML
CNhs13059	LAML
CNhs13060	LAML
CNhs13505	LAML
CNhs13049	LAML
CNhs11888	LAML
CNhs11843	LAML
CNhs10742	LGG; GBM
CNhs11846	LGG; GBM
CNhs11185	LGG; GBM
CNhs11248	LGG; GBM
CNhs11272	LGG; GBM
CNhs10731	LGG; GBM
CNhs12805	LGG; GBM
CNhs11861	LGG; GBM
CNhs11276	LGG; GBM
CNhs11284	LGG; GBM
CNhs11818	LGG; GBM
CNhs11811	LGG; GBM
CNhs11868	LIHC
CNhs11742	LIHC
CNhs12328	LIHC
CNhs12329	LIHC
CNhs12330	LIHC
CNhs11271	LIHC
CNhs11933	LIHC

CNhs11838	LUAD; LUSC
CNhs11862	LUAD; LUSC
CNhs11840	LUAD; LUSC
CNhs11841	LUAD; LUSC
CNhs11834	LUAD; LUSC
CNhs11274	LUAD; LUSC
CNhs10751	LUAD; LUSC
CNhs11277	LUAD; LUSC
CNhs12806	LUAD; LUSC
CNhs11275	LUAD; LUSC
CNhs10726	LUAD; LUSC
CNhs10625	LUAD; LUSC
CNhs11680	LUAD; LUSC
CNhs11786	LUAD; LUSC
CNhs12808	LUAD; LUSC
CNhs11285	LUAD; LUSC
CNhs12809	LUAD; LUSC
CNhs11812	LUAD; LUSC
CNhs11273	LUAD; LUSC
CNhs11263	MESO
CNhs11264	MESO
CNhs13066	MESO
CNhs13067	MESO
CNhs13068	MESO
CNhs13069	MESO
CNhs13070	MESO
CNhs13072	MESO
CNhs13073	MESO
CNhs13063	MESO
CNhs13062	MESO
CNhs13064	MESO
CNhs13061	MESO
CNhs13074	MESO
CNhs13075	MESO
CNhs11832	PAAD
CNhs11883	PAAD
CNhs11866	PCPG
CNhs11744	PCPG
CNhs11753	PCPG
CNhs11853	PCPG
CNhs11854	PCPG

CNhs11830	PCPG
CNhs11183	PCPG
CNhs11245	PCPG
CNhs10628	PRAD
CNhs11260	PRAD
CNhs11243	PRAD
CNhs11247	SARC
CNhs10728	SARC
CNhs11860	SARC
CNhs11842	SARC
CNhs11848	SARC
CNhs11722	SARC
CNhs11723	SARC
CNhs11724	SARC
CNhs11870	SARC
CNhs11851	SARC
CNhs11729	SARC
CNhs11821	SARC
CNhs11835	SARC
CNhs11279	SARC
CNhs11290	SARC
CNhs11877	SARC
CNhs11269	SARC
CNhs11857	SARC
CNhs11244	SARC
CNhs11281	SKCM
CNhs11254	SKCM
CNhs11882	STAD
CNhs11737	STAD
CNhs11819	STAD
CNhs11286	STAD
CNhs11738	STAD
CNhs10753	STAD
CNhs11270	STAD
CNhs11736	STAD
CNhs11734	STAD
CNhs11884	TGCT
CNhs11876	TGCT
CNhs12351	TGCT
CNhs12362	TGCT
CNhs11726	TGCT

CNhs10745	THCA
CNhs11716	THCA
CNhs11739	THCA
CNhs11872	THCA
CNhs11725	UCS; UCEC
CNhs11747	UCS; UCEC
CNhs11288	UCS; UCEC
CNhs11289	UCS; UCEC
CNhs11930	UCS; UCEC
CNhs11266	UCS; UCEC
CNhs11249	UCS; UCEC
CNhs11748	UCS; UCEC
CNhs11824	UCS; UCEC
CNhs11944	UCS; UCEC

Supplemental Table 12. Matching GTEx-TCGA samples

GTEx	TCGA
Adipose Tissue	SARC
Adrenal Gland	ACC
Bladder	BLCA
Blood	LAML
Bone Marrow	LAML
Brain	GBM; LGG
Breast	BRCA
Cervix Uteri	UCEC; UCS
Colon	COAD; READ
Esophagus	ESCA
Kidney	KICH; KIRC; KIRP
Liver	LIHC
Lung	LUAD; LUSC
Muscle	SARC
Nerve	PCPG
Ovary	OV
Pancreas	PAAD
Prostate	PRAD
Salivary Gland	HNSC
Small Intestine	COAD; READ
Stomach	STAD
Testis	TGCT
Thyroid	THCA
Uterus	UCEC; UCS

Supplemental Table 13. Cross-validation cohorts from Oncomine

**Cohort and test (relative publication follows)**	Cluster
1. Diffuse Gastric Adenocarcinoma vs. Normal	Digestive
Cho Gastric, Clin Cancer Res, 2011	Digestive
2. Gastric Adenocarcinoma vs. Normal	Digestive
Cho Gastric, Clin Cancer Res, 2011	Digestive
3. Gastric Intestinal Type Adenocarcinoma vs. Normal	Digestive
Cho Gastric, Clin Cancer Res, 2011	Digestive
4. Gastric Mixed Adenocarcinoma vs. Normal	Digestive
Cho Gastric, Clin Cancer Res, 2011	Digestive
5. Gastric Cancer vs. Normal	Digestive
Cui Gastric, Nucleic Acids Res, 2011	Digestive
6. Diffuse Gastric Adenocarcinoma vs. Normal	Digestive
Deng Gastric, Gut, 2012	Digestive
7. Gastric Adenocarcinoma vs. Normal	Digestive
Deng Gastric, Gut, 2012	Digestive
8. Gastric Cancer vs. Normal	Digestive
Deng Gastric, Gut, 2012	Digestive
9. Gastric Intestinal Type Adenocarcinoma vs. Normal	Digestive
Deng Gastric, Gut, 2012	Digestive
10. Gastric Mixed Adenocarcinoma vs. Normal	Digestive
Deng Gastric, Gut, 2012	Digestive
11. Diffuse Gastric Adenocarcinoma vs. Normal	Digestive
DErrico Gastric, Eur J Cancer, 2009	Digestive
12. Gastric Intestinal Type Adenocarcinoma vs. Normal	Digestive
DErrico Gastric, Eur J Cancer, 2009	Digestive
13. Gastric Mixed Adenocarcinoma vs. Normal	Digestive
DErrico Gastric, Eur J Cancer, 2009	Digestive
14. Rectal Adenocarcinoma vs. Normal	Digestive
Gaedcke Colorectal, Genes Chromosomes Cancer, 2010	Digestive
15. Colorectal Carcinoma vs. Normal	Digestive
Hong Colorectal, Clin Exp Metastasis, 2010	Digestive
16. Esophageal Squamous Cell Carcinoma vs. Normal	Digestive
Hu Esophagus, BMC Genomics, 2010	Digestive
17. Esophageal Squamous Cell Carcinoma vs. Normal	Digestive
Hu Esophagus 2, Cancer Res, 2009	Digestive
18. Cecum Adenocarcinoma vs. Normal	Digestive
Kaiser Colon, Genome Biol, 2007	Digestive
19. Colon Adenocarcinoma vs. Normal	Digestive

Kaiser Colon, Genome Biol, 2007	Digestive
20. Colon Mucinous Adenocarcinoma vs. Normal	Digestive
Kaiser Colon, Genome Biol, 2007	Digestive
21. Rectal Adenocarcinoma vs. Normal	Digestive
Kaiser Colon, Genome Biol, 2007	Digestive
22. Rectal Mucinous Adenocarcinoma vs. Normal	Digestive
Kaiser Colon, Genome Biol, 2007	Digestive
23. Rectosigmoid Adenocarcinoma vs. Normal	Digestive
Kaiser Colon, Genome Biol, 2007	Digestive
24. Barrett's Esophagus vs. Normal	Digestive
Kim Esophagus, PLoS One, 2010	Digestive
25. Esophageal Adenocarcinoma vs. Normal	Digestive
Kim Esophagus, PLoS One, 2010	Digestive
26. Esophageal Adenocarcinoma vs. Normal	Digestive
Kimchi Esophagus, Cancer Res, 2005	Digestive
27. Colon Adenocarcinoma vs. Normal	Digestive
Kurashina Colon, Cancer Sci, 2008	Digestive
28. Colon Mucinous Adenocarcinoma vs. Normal	Digestive
Kurashina Colon, Cancer Sci, 2008	Digestive
29. Rectal Adenocarcinoma vs. Normal	Digestive
Kurashina Colon, Cancer Sci, 2008	Digestive
30. Colon Adenocarcinoma vs. Normal	Digestive
Notterman Colon, Cancer Res, 2001	Digestive
31. Colon Adenoma vs. Normal	Digestive
Sabates-Bellver Colon, Mol Cancer Res, 2007	Digestive
32. Rectal Adenoma vs. Normal	Digestive
Sabates-Bellver Colon, Mol Cancer Res, 2007	Digestive
33. Colorectal Adenocarcinoma vs. Normal	Digestive
Skrzypczak Colorectal, PLoS One, 2010	Digestive
34. Colorectal Carcinoma vs. Normal	Digestive
Skrzypczak Colorectal, PLoS One, 2010	Digestive
35. Colon Adenoma Epithelia vs. Normal	Digestive
Skrzypczak Colorectal 2, PLoS One, 2010	Digestive
36. Colon Adenoma vs. Normal	Digestive
Skrzypczak Colorectal 2, PLoS One, 2010	Digestive
37. Colon Carcinoma Epithelia vs. Normal	Digestive
Skrzypczak Colorectal 2, PLoS One, 2010	Digestive
38. Colon Carcinoma vs. Normal	Digestive
Skrzypczak Colorectal 2, PLoS One, 2010	Digestive
39. Esophageal Squamous Cell Carcinoma vs. Normal	Digestive
Su Esophagus 2, Clin Cancer Res, 2011	Digestive

40. Rectal Adenocarcinoma vs. Normal	Digestive
TCGA Colorectal, No Associated Paper, 2011	Digestive
41. Rectosigmoid Adenocarcinoma vs. Normal	Digestive
TCGA Colorectal, No Associated Paper, 2011	Digestive
42. Cecum Adenocarcinoma vs. Normal	Digestive
TCGA Colorectal 2, No Associated Paper, 2011	Digestive
43. Colon Adenocarcinoma vs. Normal	Digestive
TCGA Colorectal 2, No Associated Paper, 2011	Digestive
44. Colon Mucinous Adenocarcinoma vs. Normal	Digestive
TCGA Colorectal 2, No Associated Paper, 2011	Digestive
45. Rectal Adenocarcinoma vs. Normal	Digestive
TCGA Colorectal 2, No Associated Paper, 2011	Digestive
46. Rectal Mucinous Adenocarcinoma vs. Normal	Digestive
TCGA Colorectal 2, No Associated Paper, 2011	Digestive
47. Rectosigmoid Adenocarcinoma vs. Normal	Digestive
TCGA Colorectal 2, No Associated Paper, 2011	Digestive
48. Diffuse Gastric Adenocarcinoma vs. Normal	Digestive
TCGA Gastric, No Associated Paper, 2013	Digestive
49. Gastric Adenocarcinoma vs. Normal	Digestive
TCGA Gastric, No Associated Paper, 2013	Digestive
50. Gastric Intestinal Type Adenocarcinoma vs. Normal	Digestive
TCGA Gastric, No Associated Paper, 2013	Digestive
51. Gastric Papillary Adenocarcinoma vs. Normal	Digestive
TCGA Gastric, No Associated Paper, 2013	Digestive
52. Gastric Tubular Adenocarcinoma vs. Normal	Digestive
TCGA Gastric, No Associated Paper, 2013	Digestive
53. Mucinous Gastric Adenocarcinoma vs. Normal	Digestive
TCGA Gastric, No Associated Paper, 2013	Digestive
54. Gastric Cancer vs. Normal	Digestive
Wang Gastric, Med Oncol, 2010	Digestive
Lung Adenocarcinoma vs. Normal	Lung
Beer Lung, Nat Med, 2002	Lung
2. Large Cell Lung Carcinoma vs. Normal	Lung
Hou Lung, PLoS One, 2010	Lung
3. Lung Adenocarcinoma vs. Normal	Lung
Hou Lung, PLoS One, 2010	Lung
4. Squamous Cell Lung Carcinoma vs. Normal	Lung
Hou Lung, PLoS One, 2010	Lung
5. Lung Adenocarcinoma vs. Normal	Lung
Landi Lung, PLoS ONE, 2008	Lung
6. Lung Adenocarcinoma vs. Normal	Lung

Okayama Lung, Cancer Res, 2012	Lung
7. Lung Adenocarcinoma vs. Normal	Lung
Selamat Lung, Genome Res, 2012	Lung
8. Lung Adenocarcinoma vs. Normal	Lung
Su Lung, BMC Genomics, 2007	Lung
9. Acinar Lung Adenocarcinoma vs. Normal	Lung
TCGA Lung 2, No Associated Paper, 2012	Lung
10. Lung Adenocarcinoma vs. Normal	Lung
TCGA Lung 2, No Associated Paper, 2012	Lung
11. Lung Adenocarcinoma, Mixed Subtype vs. Normal	Lung
TCGA Lung 2, No Associated Paper, 2012	Lung
12. Lung Mucinous Adenocarcinoma vs. Normal	Lung
TCGA Lung 2, No Associated Paper, 2012	Lung
13. Micropapillary Lung Adenocarcinoma vs. Normal	Lung
TCGA Lung 2, No Associated Paper, 2012	Lung
14. Mucinous Bronchioloalveolar Carcinoma vs. Normal	Lung
TCGA Lung 2, No Associated Paper, 2012	Lung
15. Non-Mucinous Bronchioloalveolar Carcinoma vs. Normal	Lung
TCGA Lung 2, No Associated Paper, 2012	Lung
16. Squamous Cell Lung Carcinoma vs. Normal	Lung
TCGA Lung 2, No Associated Paper, 2012	Lung
17. Squamous Cell Lung Carcinoma, Basaloid Variant vs. Normal	Lung
TCGA Lung 2, No Associated Paper, 2012	Lung
18. Squamous Cell Lung Carcinoma vs. Normal	Lung
Wachi Lung, Bioinformatics, 2005	Lung
19. Lung Adenocarcinoma vs. Normal	Lung
Weiss Lung, Sci Transl Med, 2010	Lung
20. Squamous Cell Lung Carcinoma vs. Normal	Lung
Weiss Lung, Sci Transl Med, 2010	Lung
2. Anaplastic Astrocytoma vs. Normal	Brain
Beroukhim Brain, Proc Natl Acad Sci U S A, 2007	Brain
3. Anaplastic Oligodendroglioma vs. Normal	Brain
Beroukhim Brain, Proc Natl Acad Sci U S A, 2007	Brain
4. Primary Glioblastoma vs. Normal	Brain
Beroukhim Brain, Proc Natl Acad Sci U S A, 2007	Brain
5. Secondary Glioblastoma vs. Normal	Brain
Beroukhim Brain, Proc Natl Acad Sci U S A, 2007	Brain
6. Anaplastic Oligoastrocytoma vs. Normal	Brain
Bredel Brain 2, Cancer Res, 2005	Brain
7. Anaplastic Oligodendroglioma vs. Normal	Brain
Bredel Brain 2, Cancer Res, 2005	Brain

8. Glioblastoma vs. Normal	Brain
Bredel Brain 2, Cancer Res, 2005	Brain
9. Oligodendroglioma vs. Normal	Brain
Bredel Brain 2, Cancer Res, 2005	Brain
10. Anaplastic Oligoastrocytoma vs. Normal	Brain
French Brain, Cancer Res, 2006	Brain
11. Anaplastic Oligodendroglioma vs. Normal	Brain
French Brain, Cancer Res, 2006	Brain
12. Pilocytic Astrocytoma vs. Normal	Brain
Gutmann Brain, Cancer Res, 2002	Brain
17. Glioblastoma vs. Normal	Brain
Lee Brain, Cancer Cell, 2006	Brain
18. Glioblastoma vs. Normal	Brain
Liang Brain, Proc Natl Acad Sci U S A, 2005	Brain
19. Oligoastrocytoma vs. Normal	Brain
Liang Brain, Proc Natl Acad Sci U S A, 2005	Brain
20. Glioblastoma vs. Normal	Brain
Murat Brain, J Clin Oncol, 2008	Brain
23. Astrocytoma vs. Normal	Brain
Shai Brain, Oncogene, 2003	Brain
24. Glioblastoma vs. Normal	Brain
Shai Brain, Oncogene, 2003	Brain
25. Oligodendroglioma vs. Normal	Brain
Shai Brain, Oncogene, 2003	Brain
27. Anaplastic Astrocytoma vs. Normal	Brain
Sun Brain, Cancer Cell, 2006	Brain
28. Diffuse Astrocytoma vs. Normal	Brain
Sun Brain, Cancer Cell, 2006	Brain
29. Glioblastoma vs. Normal	Brain
Sun Brain, Cancer Cell, 2006	Brain
30. Oligodendroglioma vs. Normal	Brain
Sun Brain, Cancer Cell, 2006	Brain
31. Brain Glioblastoma vs. Normal	Brain
TCGA Brain, No Associated Paper, 2013	Brain
32. Glioblastoma vs. Normal	Brain
TCGA Brain, No Associated Paper, 2013	Brain
33. Brain Astrocytoma vs. Normal	Brain
TCGA Brain 2, No Associated Paper, 2013	Brain
34. Brain Glioblastoma vs. Normal	Brain
TCGA Brain 2, No Associated Paper, 2013	Brain
35. Brain Oligodendroglioma vs. Normal	Brain

TCGA Brain 2, No Associated Paper, 2013	Brain
36. Glioblastoma vs. Normal	Brain
TCGA Brain 2, No Associated Paper, 2013	Brain
37. Malignant Glioma, NOS vs. Normal	Brain
TCGA Brain 2, No Associated Paper, 2013	Brain
38. Oligoastrocytoma vs. Normal	Brain
TCGA Brain 2, No Associated Paper, 2013	Brain
1. Cervical Squamous Cell Carcinoma vs. Normal	Ovary-Uterine
Biewenga Cervix, Gynecol Oncol, 2008	Ovary-Uterine
2. Ovarian Clear Cell Adenocarcinoma vs. Normal	Ovary-Uterine
Lu Ovarian, Clin Cancer Res, 2004	Ovary-Uterine
3. Ovarian Endometrioid Adenocarcinoma vs. Normal	Ovary-Uterine
Lu Ovarian, Clin Cancer Res, 2004	Ovary-Uterine
4. Ovarian Mucinous Adenocarcinoma vs. Normal	Ovary-Uterine
Lu Ovarian, Clin Cancer Res, 2004	Ovary-Uterine
5. Ovarian Serous Adenocarcinoma vs. Normal	Ovary-Uterine
Lu Ovarian, Clin Cancer Res, 2004	Ovary-Uterine
6. Cervical Cancer vs. Normal	Ovary-Uterine
Pyeon Multi-cancer, Cancer Res, 2007	Ovary-Uterine
7. Cervical Adenocarcinoma vs. Normal	Ovary-Uterine
Scotto Cervix, Genes Chromosomes Cancer, 2008	Ovary-Uterine
8. Cervical Squamous Cell Carcinoma vs. Normal	Ovary-Uterine
Scotto Cervix, Genes Chromosomes Cancer, 2008	Ovary-Uterine
9. Cervical Keratinizing Squamous Cell Carcinoma vs. Normal	Ovary-Uterine
TCGA Cervix, No Associated Paper, 2012	Ovary-Uterine
10. Cervical Non-Keratinizing Squamous Cell Carcinoma vs. Normal	Ovary-Uterine
TCGA Cervix, No Associated Paper, 2012	Ovary-Uterine
11. Cervical Squamous Cell Carcinoma vs. Normal	Ovary-Uterine
TCGA Cervix, No Associated Paper, 2012	Ovary-Uterine
12. Ovarian Serous Cystadenocarcinoma vs. Normal	Ovary-Uterine
TCGA Ovarian 2, No Associated Paper, 2013	Ovary-Uterine
13. Ovarian Serous Adenocarcinoma vs. Normal	Ovary-Uterine
Yoshihara Ovarian, Cancer Sci, 2009	Ovary-Uterine

Supplemental Table 14. Nomenclature match – The Cancer Genome Atlas (TCGA) and The Human Protein Atlas (THPA)

TCGA nomenclature	THPA nomenclature
BLCA	urothelial cancer
BRCA	breast cancer
CESC	cervical cancer
COAD	colorectal cancer
HNSC	head and neck cancer
KICH	renal cancer
KIRC	renal cancer
KIRP	renal cancer
GBM	glioma
LGG	glioma
LIHC	liver cancer
LUAD	lung cancer
LUSC	lung cancer
OV	ovarian cancer
PAAD	pancreatic cancer
PRAD	prostate cancer
READ	colorectal cancer
SKCM	melanoma
STAD	stomach cancer
TGCT	testis cancer
THCA	thyroid cancer
UCEC	endometrial cancer
